# Downregulation of miR-181b-5p Inhibits the Viability, Migration, and Glycolysis of Gallbladder Cancer by Upregulating PDHX Under Hypoxia

**DOI:** 10.3389/fonc.2021.683725

**Published:** 2021-08-16

**Authors:** Yiyu Qin, Yongliang Zheng, Cheng Huang, Yuanyuan Li, Min Gu, Qin Wu

**Affiliations:** ^1^Clinical Medical College, Jiangsu Vocational College of Medicine, Yancheng, China; ^2^Rehabilitation College, Jiangsu Vocational College of Medicine, Yancheng, China

**Keywords:** gallbladder cancer, miR-181b-5p, hypoxia, viability, pyruvate dehydrogenase complex component X, glycolysis

## Abstract

**Background:**

Gallbladder cancer (GBC) is a malignant cancer with poor prognosis. Evidences have shown that miRNAs are closely related to the occurrence of GBC; thus, we aimed to explore miRNAs, which plays an important role in the occurrence and development of GBC.

**Methods:**

Microarray analysis was performed to investigate the differentially expressed miRNAs between five non-neoplastic gallbladder tissues (normal tissues) and five gallbladder tumor tissues (tumor tissues). RT-qPCR was performed to detect the level of miR-181b-5p in cells, and CCK-8 was performed to detect cell viability. Then, glucose assay kit or lactic acid assay kit was performed to detect the level of glucose consumption or lactate production. Next, transwell and wound healing assays were used to assess cell migration. In addition, dual-luciferase reporter assay was used to verify the relationship between miR-181b-5p and PDHX. At last, Western blotting was performed to determine the protein level of PDHX.

**Results:**

Microarray analysis suggested miR-181b-5p was significantly upregulated in GBC tumor tissue. KEGG analysis for the protein targets of miR-181b-5p indicates a close relationship existed between miR-181b-5p and glycolysis. In addition, the level of miR-181b-5p was notably increased in GBC-SD or G415 cells, compared with HIBEpiC cells. GBC cell viability was significantly decreased under hypoxia, and these decreases were exacerbated by miR-181b-5p antagomir. Moreover, glucose consumption or lactate production of GBC cells was significantly upregulated under hypoxia, whereas these increases were completely revered by miR-181b-5p antagomir. Further investigation revealed that PDHX was a direct target of miR-181b-5p.

**Conclusion:**

In this study, downregulation of miR-181b-5p inhibits the viability, migration, and glycolysis of GBC by upregulating PDHX under hypoxia. This finding suggested that miR-181b-5p might be considered as a novel therapeutic target for the treatment of GBC.

## Introduction

Gallbladder carcinoma (GBC) is a malignant tumor originating from the epithelial cells of gallbladder mucosa (1, 2). In addition, GBC is the most common malignancy of biliary tract, accounting for more than 70% of biliary tract malignancy (3). The main causes of GBC include gallbladder stones, specific types of gallbladder lesions, bacterial infections, and genetic factors (4). GBC can be divided into five stages, according to the severity of the patient’s condition (5). Treatment can be divided into drug therapy, surgery, radiation therapy, and chemical therapy based on the stage of GBC (6). However, most patients with GBC were diagnosed at a late stage and had a poor prognosis (7). Piehler et al. reported that the 5-year survival rate for patients with GBC is only 4.1% (8, 9). Despite people having made a lot of efforts in the study of GBC, effective treatments for GBC are still limited.

MicroRNAs (miRNAs) are a kind of endogenous, single-stranded small RNA with 20 to 24 nucleotides in length (10). MiRNA exists widely in higher eukaryotes and plays an important role in regulating gene expression (11). From the perspective of biological mechanism, the expression of miRNA is always changing with the generation and decay of tumor cells; thus, the level of each miRNA represents the information of health or disease in the human body at a certain moment (12).

It has been reported that miRNAs are closely related to the occurrence of GBC (13, 14). Li et al. indicated that specific miRNAs are involved in the development and progression of GBC by regulating cell proliferation and metastasis (13). Liu et al. found that the expression of miR-30d-5p was downregulated in GBC tissues and cell lines (14). In this study, we aimed to explore miRNAs, which plays an important role in the occurrence and development of GBC.

## Materials and Methods

### Patients and Samples

GBC tissues and adjacent tissues samples were obtained from 10 patients with GBC in Xinhua Hospital Affiliated to Shanghai Jiao Tong University School of Medicine. The samples were snap-froze in liquid nitrogen and immediately stored at −80°C for further analysis. Microarray was used to analyze the differentially expressed genes (DEGs) in five pairs of GBC tissues and adjacent tissues. In addition, RT-qPCR was used to confirm the DEGs levels in another five pairs of GBC tissues and adjacent tissues. These studies were approved by the ethics committee of Xinhua Hospital Affiliated to Shanghai Jiao Tong University School of Medicine, and the informed consent form was signed by each participant.

### Microarray and DEGs Analysis

Total RNA was isolated from tissues using the Trizol reagent (ELK Biotechnology, Wuhan, China). Nanodrop 2000 spectrophotometer (Thermo Fisher Scientific, Waltham, MA, USA) was used to qualify the concentration of RNAs. Affymetrix Expression Console Software was used for microarray analysis. DESeq2 was used for differentially expressed gene analysis (15). DEGs were identified with p ≤ 0.05 and AveExpr ≥ 3.

### KEGG Analysis for the Protein Targets of DEGs

The protein targets of DEGs were analyzed with miRTarBase. ClusterProfiler software was used for KEGG (Kyoto Encyclopedia of Genes and Genomes) analysis. KEGG database (KEGG, http://www.genome.jp/kegg/) was used to determine the potential pathways of the protein targets of DEGs.

### Cell Culture

Human intrahepatic biliary epithelial cells (HIBEpiC) were provided by ScienCell (Carlsbad, CA, USA); the gallbladder carcinoma cell lines GBC-SD and G415 were provided with Cell Bank of the Chinese Academy of Science (Shanghai, China) and RIKEN Cell Engineering Division-Cell Bank (Tokyo, Japan), respectively. Cells were cultured in DMEM (FBS (10%) + penicillin (100 U/ml) + streptomycin (100 mg/ml) (Thermo Fisher Scientific)) at 37°C, 5% CO_2_. For hypoxia, cells were cultured in a hypoxia chamber (MiniGalaxy A, RS Biotech, Irvine, Scotland) with 1% O_2_.

### Cell Transfection

MiR-181b-5p agomir, miR-181b-5p antagomir, and negative control (NC) were purchased from GenePharma (Shanghai, China). MiR-181b-5p agomir (10 nM), miR-181b-5p antagomir (10 nM), or NC was transfected into GBC-SD or G415 cells with Lipofectamine^®^ 2000 (Thermo Fisher Scientific).

SiRNA against PDHX (si PDHX-1, si PDHX-2, and siRNA-ctrl, 10 μM) were purchased from RiboBio (Guangzhou, China). Si PDHX-1, si PDHX-2, or siRNA-ctrl was transfected into GBC-SD or G415 cells with Lipofectamine^®^ 2000. The sequences of si PDHX-1, si PDHX-2, and siRNA-ctrl were presented as follows: si PDHX-1, 5′-CAACCCAATGCAGTGGGCACATTCA-3′; si PDHX-2, 5′-CAGCAGCTGTTACCCTTAAACAAAT-3′; siRNA-ctrl, 5′-CAAACTACGGAGTGGACACTCCTCA-3′.

### Reverse Transcription-Quantitative Polymerase Chain Reaction

Total RNA was isolated from cells using the Trizol reagent (ELK Biotechnology, Wuhan, China). Next, RNA samples were reversed transcribed into cDNA using an EntiLink™ 1st-Strand cDNA Synthesis Kit (ELK Biotechnology). Then, RT-qPCR was carried out using the EnTurbo™ SYBR Green PCR SuperMix (ELK Biotechnology) on the StepOne™ Real-Time PCR System (Thermo Fisher Scientific). The 2^−ΔΔCT^ method was used for data analysis. The U6 gene worked as an internal control. The primers were as follows: U6, forward, 5′-CTCGCTTCGGCAGCACAT-3′, reverse, 5′-AACGCTTCACGAATTTGCGT-3′; hsa-miR-181b-5p, forward, 5′-TCGGTGGGTCTCAACTGAATT-3′, reverse, 5′-CTCAACTGGTGTCGTGGAGTC-3′. PDHX, forward, 5′-AAGATTACCGAC TCCAGACCAA-3′, reverse: 5′-TGTCCAGGAGTTGATACTGCTG-3′. Actin, forward, 5′-GTCCACCGCAAATGCTTCTA-3′, reverse: 5′-TGCTGTCACCTTCACCGTTC-3′.

### Cell Counting Kit-8 Assay

GBC and G415 cells (5 × 10^3^ cells per well) were plated into 96-well plates. Next, cells were incubated at 37°C overnight. Next, 10 μl of CCK-8 (Dojindo, Kumamoto, Japan) reagent was added into each well. Then, the cells were incubated with CCK-8 reagent at 37°C for 2 h. Finally, the absorbance of cells was measured at 450 nm using a microplate reader (Bio-Rad, Hercules, CA, USA).

### Glucose and Lactic Acid Assay

GBC and G415 cells were plated onto six-well plates overnight and then maintained in normoxia or hypoxia condition for 48 h. After that, the level of glucose consumption or lactate production in the supernatant was detected using Glucose Assay Kit or Lactic Acid assay kit (Nanjing Jiancheng Company, Nanjing, China).

### Transwell Assay

Transwell and matrigel-coated transwell (8 μm pore size, Corning, USA) were used to detect GBC cells migration and invasion ability, respectively. The upper chamber was added with GBC cells suspended in 100 µl of FBS-free medium. The lower chamber was added with 600 μl of DMEM medium supplemented with 10% FBS. After 24 h of incubation, cells that migrated or invaded through the transwell membrane were fixed with 4% paraformaldehyde. Then, the cells were stained with 0.1% crystal violet for 10 min and imaged using a fluorescence microscope.

### Wound Healing

GBC cells (5 × 10^5^/cell) were plated into six-well plate. When the cells formed a fused monolayer, scratches were made with sterilized 10 L pipette tips. Next, images were captured at 48 h using a microscope after scratching.

### Dual-Luciferase Reporter Assay

The pGL6-miR–based luciferase reporter plasmids (Beyotime, Beijing, China) containing wild‐type PDHX 3′UTR (pGL6 3′UTR‐wt) or PDHX mutated (pGL6 3′UTR‐mut) at the putative miR‐181b-5p binding sites were designed. Next, pGL6 3′UTR‐wt or pGL6 3′UTR‐mut, together with either agomir-control or miR‐181b-5p agomir were transfected into GBC cells with Lipofectamine^®^ 2000 for 48 h. Finally, luciferase activity in cell lysates was detected with the Dual Luciferase Reporter Assay System (Beyotime). The renilla luciferase activity worked as an internal control.

### Western Blot Assay

Total protein was isolated from cells using Protein Lysis Buffer (Beyotime, Shanghai, China). Equivalent amounts of proteins (25 μg) were separated by 10% sodium dodecyl sulfate-polyacrylamide gel electrophoresis (SDS-PAGE). Then, the proteins were transferred to the polyvinylidene fluoride (PVDF) membranes. After blocking with 3% skim milk, the membranes were incubated with primary antibodies at 4°C overnight. Next, the membranes were incubated with secondary antibody (Abcam; 1:5000) for 1 h at room temperature. The efficient chemiluminescence (ECL) kit (Thermo Fisher Scientific) was used to detect the protein bands. The primary antibodies used in the present study were as follows: anti-PDHX (1:1000), HIF-1α (1: 1000), anti-β-actin (1:1000). The β-actin worked as an internal control.

### JC-1 Fluorescence Assay

The mitochondrial membrane potential (MMP) was determined using JC-1 staining assay. GBC and G415 cells were incubated with JC-1 reagent for 20 min in the dark. Subsequently, images were taken under a fluorescence microscope.

### Measurement of ATP Concentrations

The ATP concentrations GBC and G415 cells were detected using the ATP Assay Kit (Beyotime, Bejing, China) according to the manufacturer’s protocol. A luminometer (Victor X3, multilabel counter, Perkin Elmer) was used to measure the emitted light.

### Statistical Analysis

All data were expressed as the mean ± SD of at least three repeated experiments. ANOVA, followed by Tukey’s tests, was used to compare the differences between multiple groups (GraphPad Prism, La Jolla, CA, USA). In the study, *P < 0.05 was considered to indicate a statistically significant result.

## Results

### DEGs Profiling in GBC Tissues

**Figure 1:**
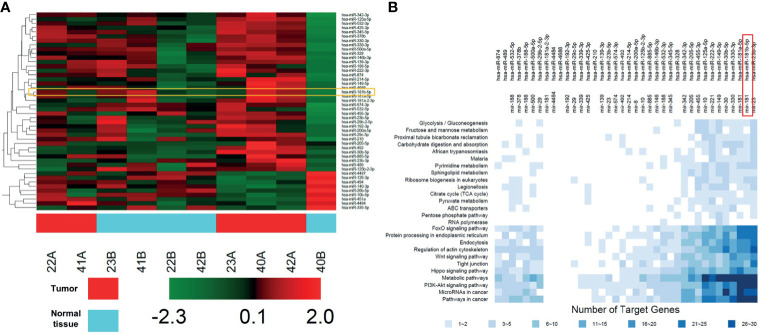
DEGs profiling in GBC tissues. **(A)** Heat map showing these differentially expressed miRNAs in five non-neoplastic gallbladder tissues (normal tissues) and five gallbladder tumor tissues (tumor tissues). Red represents upregulated genes, green represents downregulated genes. **(B)** KEGG analysis of protein targets of these DEGs.

Microarray was firstly performed to explore DEGs profiling between five non-neoplastic gallbladder tissues (normal tissues) and five gallbladder tumor tissues (tumor tissues). These differentially expressed miRNAs were presented in Heatmap (**Figure 1A**). Next, KEGG analysis was performed to investigate the enrichment function and pathways of the target genes of DEGs (**Figure 1B** and **Supplementary Figure 1**). KEGG analysis revealed that the upregulated targets were mostly involved in “glycolysis”, “gluconeogenesis”, and “metabolic pathways” (**Figure 1B**).

### The Expression Level of miR-181b-5p Is Up-Regulated in GBC

**Figure 2:**
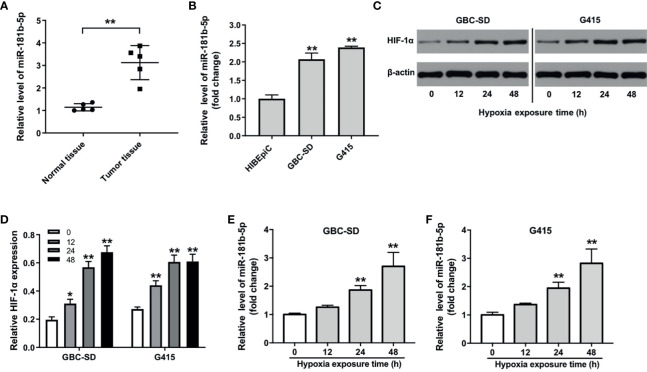
The expression level of miR-181b-5p is upregulated in GBC. **(A)** PCR was performed to measure the level of miR-181b-5p in five non-neoplastic gallbladder tissues (normal tissues) and five gallbladder tumor tissues (tumor tissues). **(B)** RT-qPCR was performed to measure the level of miR-181b-5p in HIBEpiC, GBC-SD or G415 cells. **(C, D)** GBC-SD or G415 cells was exposed to hypoxia (a humidified atmosphere of 1% O_2_) for 0, 12, 24, 48 h Western blot was performed to measure the expression of HIF-1α in GBC-SD or G415 cells. **(E, F)** GBC-SD or G415 cells was exposed to hypoxia (a humidified atmosphere of 1% O_2_) for 0, 12, 24, 48 h RT-qPCR was performed to measure the level of miR-181b-5p in GBC-SD or G415 cells. ^*^P < 0.05, ^**^P < 0.01 compared with control group, n = 3.

As shown in Heatmap and KEGG analysis results, miR-181b-5p was one of the remarkably upregulated miRNAs in GBC. Because miR-181b-5p was reported to be closely related to the occurrence and development of GBC (16), miR-181b-5p was selected for further investigation. We firstly confirm the expression of miR-181b-5p in GBC cancer using with RT-qPCR. As indicated in **Figure 2A**, compared with normal tissues, miR-181b-5p level was found to be highly expressed in tumor tissues. Consistently, the level of miR-181b-5p was notably upregulated in GBC cells, compared with HIBEpiC cells (**Figure 2B**). Microarray and KEGG analysis revealed that miR-181b-5p was mostly involved in “glycolysis”. In addition, more and more evidence shows that hypoxic microenvironment is prevalent in human tumors (17). Hypoxic microenvironment is a common feature of all solid tumors and an important marker of tumor microenvironment (18). To explore the role of miR-181b-5p in GBC, GBC-SD or G415 cells were exposed to hypoxia (a humidified atmosphere of 1% O_2_) for 0, 12, 24, 48 h. The results of Western blot assay showed that HIF-1α protein level was increased in GBC cells under hypoxia in a time-dependent manner (**Figures 2C, D**). Moreover, RT-qPCR analysis showed that hypoxia increased the expression of miR‐181b-5p in GBC cells in a time-dependent manner (**Figures 2E, F**). These results indicated that the level of miR-181b-5p was notably upregulated in GBC.

### Downregulation of miR-181b-5p Inhibits Cell Viability and Glycolysis in GBC Under Hypoxia

**Figure 3:**
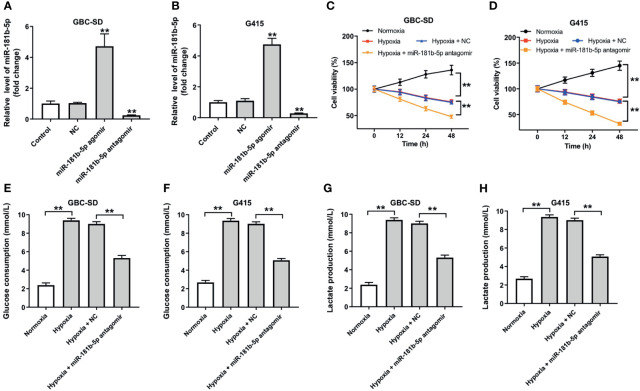
Downregulation of miR-181b-5p inhibits cell viability and glycolysis in GBC under hypoxia. **(A, B)** GBC cells were transfected with miR-181b-5p agomir, antagomir, or negative control for 24 h RT-qPCR was performed to measure the level of miR-181b-5p in GBC-SD or in G415 cells. GBC cells were treated with hypoxia or hypoxia plus miR-181-5p antagomir **(C, D)** CCK-8 assay was used to detect GBC-SD or G415 cells viability. **(E–H)** Glucose Assay Kit or Lactic Acid assay kit was performed to detect the level of glucose consumption or lactate production in the cell supernatant. ^**^P < 0.01 compared with control group, n = 3.

To explore the role of miR-181b-5p in GBC, miR-181b-5p agomir and miR-181b-5p antagomir were transfected into GBC cells. RT-qPCR result indicated that miR-181b-5p agomir notably increased the expression of miR-181b-5p in GBC cells; in contrast, miR-181b-5p antagomir decreased the expression of miR-181b-5p (**Figures 3A, B**). In addition, cell viability was significantly decreased in GBC cells under hypoxia, and this phenomenon was exacerbated by miR-181b-5p antagomir (**Figures 3C, D**). According to the KEGG analysis, miR-181b-5p has been demonstrated to have a close relationship with glucose metabolism; thus, we explored if miR-181b-5p could regulate glucose consumption or lactate production of GBC cells under hypoxia condition. As indicated in **Figures 3E–H**, glucose consumption and lactate production were significantly upregulated under hypoxia, whereas these increases were completely revered by miR-181b-5p antagomir. Taken together, downregulation of miR-181b-5p significantly inhibited cell viability and glycolysis in GBC under hypoxia.

### Downregulation of miR-181b-5p Inhibits the Migration of GBC Under Hypoxia

**Figure 4:**
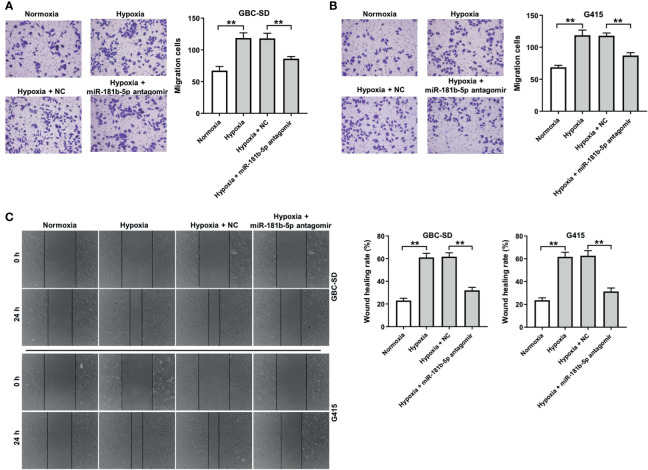
Downregulation of miR-181b-5p inhibits the migration of GBC under hypoxia. GBC cells were treated with hypoxia or hypoxia plus miR-181-5p antagomir for 24 h **(A, B)** Migrated cells were stained with 0.1% crystal violet and counted at three random fields. **(C)** The ability of cell migration was detected with wound healing assay. ^**^P < 0.01, n = 3.

To investigate the effect of miR-181b-5p on the migration ability of GBC cells under hypoxia, transwell assay was conducted. The result of transwell indicated that hypoxia significantly promoted cells migration ability, whereas miR-181b-5p antagomir notably reversed this phenomenon (**Figures 4A, B**). Consistently, the data of wound healing assay indicating hypoxia-induced cell migration were completely reversed by miR-181b-5p antagomir (**Figure 4C**). These results suggested that downregulation of miR-181b-5p could inhibit the migration of GBC under hypoxia.

### PDHX Is a Direct Target of miR-181b-5p

**Figure 5:**
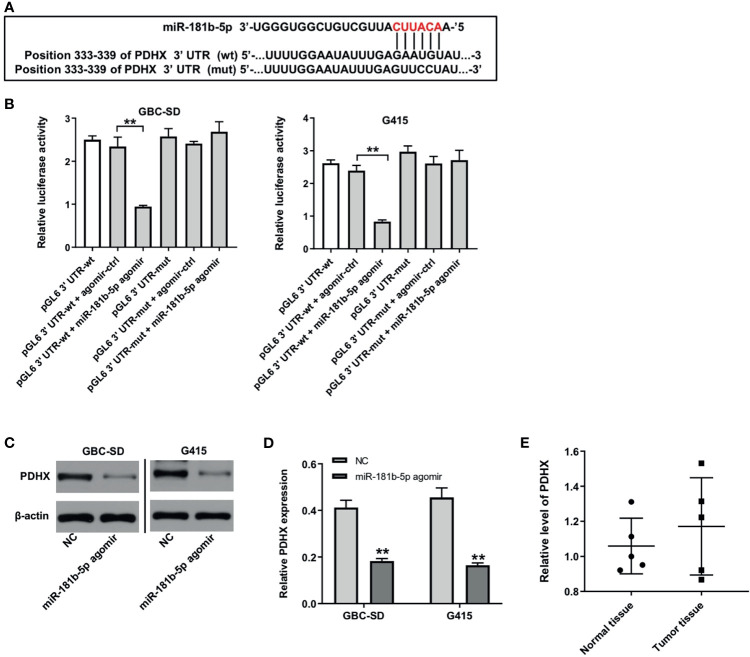
PDHX is a direct target of miR-181b-5p. **(A)** Sequence alignment of miR-181b-5p with the binding sites within the wt or mut regions of PDHX. **(B)** Dual-luciferase reporter assay was used to verify the relationship between miR-181b-5p and PDHX. **(C, D)** Western blotting was used to determine the level of PDHX. **(E)** RT-qPCR was performed to measure the level of PDHX in five non-neoplastic gallbladder tissues (normal tissues) and five gallbladder tumor tissues (tumor tissues). ^**^P < 0.01 compared with control group, n = 3.

There online bioinformatics tools Targetscan (http://www.targetscan.org/vert_72/), miRDB (http://www.mirdb.org), and miRWalk (http://zmf.umm.uni-heidelberg.de/apps/zmf/mirwalk/micrornapredictedtarget.html) were used to predict the target genes of miR-181b-5p. These three online bioinformatics databases commonly suggested pyruvate dehydrogenase complex (PDHX), which might be a potential target of miR-181b-5p (**Figure 5A**). Next, dual luciferase reporter assay revealed that miR-181b-5p agomir reduced the luciferase activity of GBC cells with PDHX-wt, but it did not affect the luciferase activity of cells with PDHX-mut (**Figure 5B**). In addition, Western blot results indicated that miR-181b-5p agomir notably downregulated the level of PDHX in GBC cells (**Figures 5C, D**). Moreover, the level of PDHX was slightly upregulated in GBC tumor tissues, compared with normal tissues (**Figure 5E**). Meanwhile, the expression of PDHX was slightly downregulated in HIBEpiC cells under hypoxic condition compared with under normoxic condition (**Supplementary Figures 2A, B**). All these results illustrated that PDHX was a direct target of miR-181b-5p.

### Downregulation of miR-181b-5p Inhibits the Viability, Migration, and Glycolysis of GBC by Upregulating PDHX Under Hypoxia

**Figure 6:**
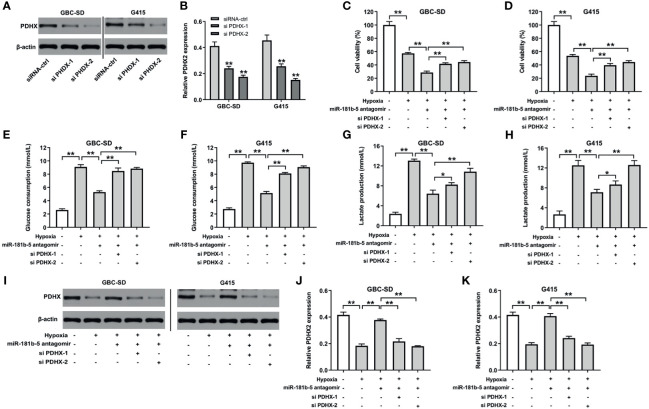
Downregulation of miR-181b-5p inhibits the viability, migration, and glycolysis of GBC by upregulating PDHX under hypoxia. **(A, B)** Western blotting was used to determine the level of PDHX in GBC cells. **(C, D)** CCK-8 assay was used to detect GBC cell viability. **(E–H)** Glucose Assay Kit or Lactic Acid assay kit was performed to detect the level of glucose consumption or lactate production in the supernatant. **(I–K)** Western blotting was used to determine the level of PDHX. *P < 0.05, **P < 0.01 compared with control group, n = 3.

To further explore the mechanism by which miR-181b-5p regulated the progression of GBC, Western blot and CCK8 assays were conducted. The results indicated that the expression of PDHX was notably downregulated in GBC cells after transfection with si PDHX-1 or si PDHX-2 (**Figures 6A, B**). In addition, miR-181b-5p antagomir markedly decreased the viability of GBC cells under hypoxia; however, this phenomenon was reversed by PDHX-1 siRNAs (**Figures 6C, D**). Moreover, miR-181b-5p antagomir significantly decreased glucose consumption or lactate production in GBC cells under hypoxia, whereas these phenomena were reversed by PDHX-1 siRNAs as well (**Figures 6E–H**). Meanwhile, the expression of PDHX was significantly downregulated in GBC-SD and G415 cells under hypoxic condition compared with under normoxic condition (**Figures 6I–K**). In contrast, miR-181b-5p antagomir obviously increased the expression of PDHX in GBC cells under hypoxia; however, these increases were notably reversed in the presence of PDHX-1 siRNAs (**Figures 6I–K**). Collectively, miR-181b-5p antagomir could inhibit the viability, migration, and glycolysis of GBC by upregulating PDHX under hypoxia.

**Figure 7:**
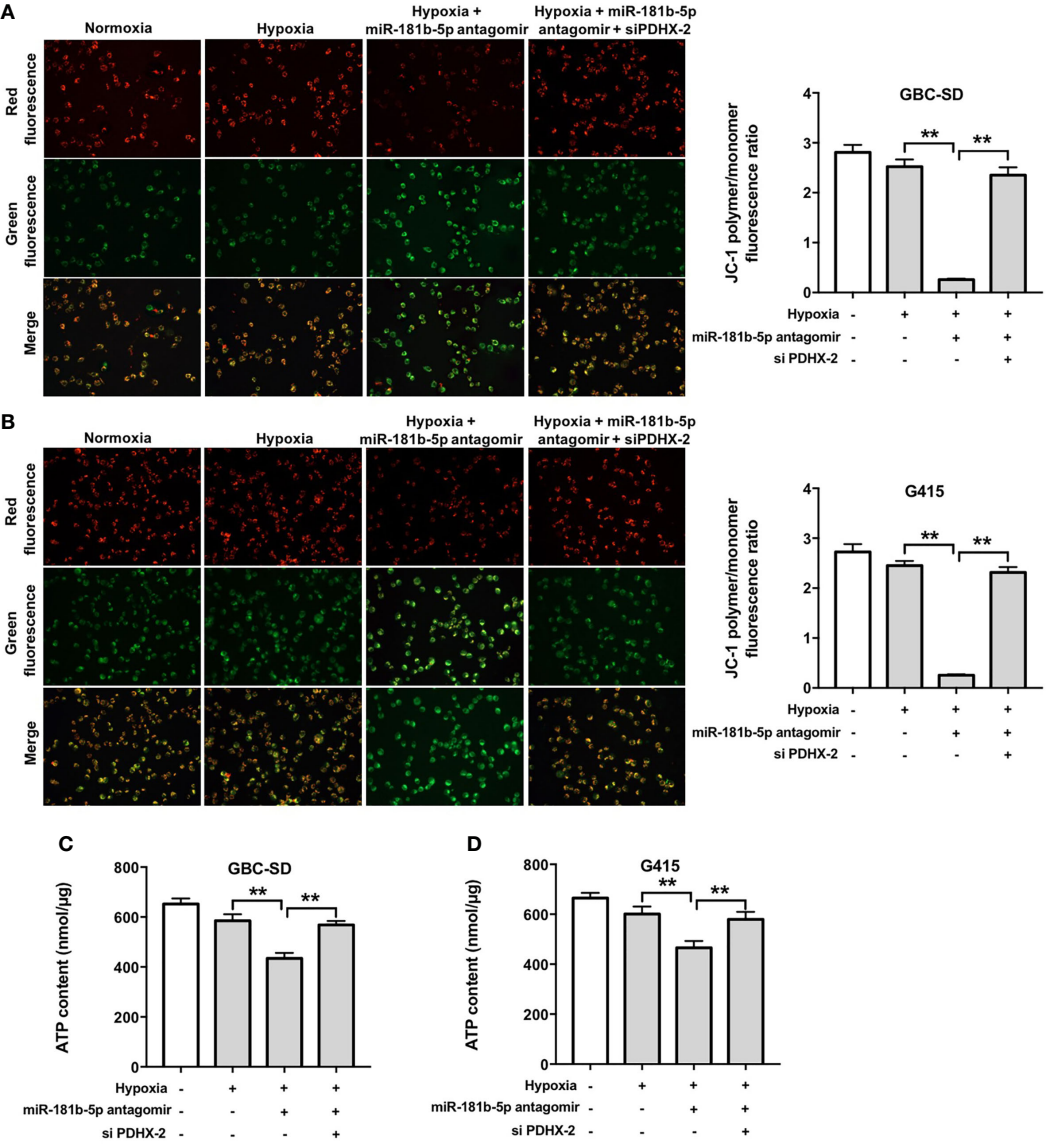
Downregulation of miR-181b-5p impairs the mitochondrial function in GBC cells under hypoxia by upregulating PDHX. **(A, B)** JC-1 staining assay was used to study the change in MMP in GBC cells. **(C, D)** ATP level in GBC cells were detected. ^**^P < 0.01.

Furthermore, as shown in **Figures 7A–D**, hypoxia slightly reduced MMP and ATP levels in GBC cells compared with normoxia group. Significantly, miR-181b-5p antagomir reduced MMP and ATP levels in GBC cells under hypoxia; however, these changes were markedly reversed in the presence of PDHX-1 siRNAs. These data indicated that miR-181b-5p antagomir could impair the mitochondrial function in GBC cells under hypoxia by upregulating PDHX.

## Discussion

In recent years, GBC has become one of the major diseases harmful to human health, which brings great pain and trouble to human beings (19). MiRNAs emerge as promising biomarkers and therapeutic targets for the treatment of GBC (20). In this study, we identified dysregulated miRNAs in GBC tissues using with Microarray and KEGG analysis. Highly upregulated miRNAs were screened out, including miR-181b-5p. Evidences have shown that miR-181b is a tumor promoter in various human cancers, such as colon cancer and breast cancer (21, 22). In addition, Zheng et al. found that the level of miR-181b was increased in doxorubicin-resistant breast cancer cells, and downregulation of miR-181b could enhance the sensitivity of breast cancer cells to doxorubicin (23). We found that miR-181b-5p level was increased in GBC cells and further increased after the cells exposed to hypoxia. These data suggested that miR-181b might act as an important factor in human cancers that modifies various biological processes, such as hypoxia and chemoresistance.

Interestingly, KEGG analysis results showed that miR-181b-5p was extensively involved in cell glycolysis. Evidence in literature has shown that metabolism of tumor cells depends on glycolysis and oxidative phosphorylation (20). Hypoxia is an important feature of cancer, which affects the migration, development, and metabolism of cancer (24). Through aerobic glycolysis (Warburg effect), the adaptability of tumor cells to the microenvironment *in vivo* is enhanced, and the proliferation of tumor cells is promoted (25, 26). Ren et al. indicated that the up-regulated expression of miR-200b and miR-200c could inhibit the glycolysis, migration, and invasion of breast cancer cells under hypoxia (24). Similar to these studies, we found that the downregulation of miR-181b-5p inhibited the viability, migration, and glycolysis of GBC cells under hypoxia.

It has been reported that the expression of miRNAs is inversely associated with the level of its targeting protein PDHX (27). PDHX has been found to be involved in altered glucose metabolism in tumors (28, 29). It has been reported that overexpression of miR-26a could improve the accumulation of pyruvate in HCT116 cells by inhibiting PDHX expression (30). Wang et al. indicated that miR-26a regulated the glucose metabolism pathway through PDHX pathway (31). Consistent with previous studies results, downregulation of miR-181b-5p inhibited the glycolysis of GBC cells under hypoxia by upregulating PDHX. In addition, Tang et al. found that pyruvate dehydrogenase B could promote the migration of the nasopharyngeal carcinoma cells (32), suggesting that pyruvate dehydrogenase might affect the migration of tumor cells. Thus, in the future, we aimed to investigate whether miR-181b-5p could promote tumor cell migration *via* targeting PDHX.

It has been shown that mitochondria are responsible for energy (ATP) production by regulating energy metabolism (33). The mitochondrial PDHX has been found to participated in cellular energy metabolism *via* converting pyruvate into acetyl-CoA and connecting glycolysis to the tricarboxylic acid cycle (29, 34). Thus, we investigated whether miR-181b-5p could affect mitochondrial function in GBC cells under hypoxia *via* targeting PDHX. We found that miR-181b-5p antagomir significantly reduced MMP and ATP levels in GBC cells under hypoxia; however, these changes were markedly reversed in the presence of PDHX-1 siRNAs. These data indicated that miR-181b-5p antagomir could impair the mitochondrial function in GBC cells under hypoxia. Porcelli et al. found that mitochondrial impairment may induce destabilization of HIF-1a and then lead to the suppression of tumor growth (35). In this study, we found that miR-181b-5p antagomir reduced the viability of GBC cells during hypoxia. These results suggested that miR-181b-5p antagomir could impair the mitochondrial function in GBC cells under hypoxia, and eventually suppress GBC cell proliferation.

Of course, there are some limitations in this study. For example, other targets regulated by miR-181b-5p remain unclear; in this paper, the mechanism research is not in-depth enough. Therefore, more research is needed in the future. In addition, evidence has shown that hypoxia could regulate miRNAs expression in human cancers *via* a HIF-dependent or a HIF-independent pathway (36). He et al. found that HIF-1α could upregulate the level of miR-224 in gastric cancer, suggesting that the induction of miR-224 by hypoxia is an HIF-1α–dependent manner (37). In contrast, Xu et al. found that HIF-1α knockdown could not decrease the level of miR-181b in retinoblastoma, indicating that induction of miR-181b by hypoxia is HIF-1α–independent manner (38). In addition to HIF-1, other genes may be involved in the adaptation of cancer cells to the hypoxic environments (36). Polytarchou et al. found that the induction of miR-21 by hypoxia is an Akt2-dependent manner (39). These data indicated that other genes may be activated by hypoxia and promote cancer progression *via* induction of miR-181b-5p. Thus, further studies are needed to investigate the molecular mechanism of miR-181b-5p in GBC cells exposed to hypoxia. Meanwhile, further study is needed to evaluate the diagnostic value of miR-181b-5p in GBC by using a large number of clinical specimens.

All in all, our findings suggest that miR-181b-5p might serve as a novel potential prognostic biomarker or molecular therapeutic target for the treatment of GBC and inspire further research on it.

## Data Availability Statement

The data presented in the study are deposited in the GEO repository, accession number GSE62335.

## Ethics Statement

The studies involving human participants were reviewed and approved by Ethics Committee of Xinhua Hospital Affiliated to Shanghai Jiao Tong University School of Medicine. The patients/participants provided their written informed consent to participate in this study.

## Author Contributions

YQ made major contributions to the conception, design, and manuscript drafting of this study. YZ, CH, YL, MG, and QW were responsible for data acquisition, data analysis, data interpretation, and manuscript revision. YQ made substantial contributions to conception and design of the study and revised the manuscript critically for important intellectual content. All authors contributed to the article and approved the submitted version.

## Conflict of Interest

The authors declare that the research was conducted in the absence of any commercial or financial relationships that could be construed as a potential conflict of interest.

## Publisher’s Note

All claims expressed in this article are solely those of the authors and do not necessarily represent those of their affiliated organizations, or those of the publisher, the editors and the reviewers. Any product that may be evaluated in this article, or claim that may be made by its manufacturer, is not guaranteed or endorsed by the publisher.

## Supplementary Material

Supplementary Figure 1 KEGG analysis of protein targets of DEGs. KEGG analysis of protein targets of miR-126-3p, miR-451a, miR-4487, miR-140-3p, miR-494, miR-10b-5p, miR-26b-5p, miR-335-5p.

Supplementary Figure 2 The expression of PDHX in HIBEpiC cells under hypoxic condition. **(A, B)** HIBEpiC cells were treated with hypoxia or hypoxia. Western blot was performed to measure the expression of PDHX in HIBEpiC cells.
