# Supplementary figures and images for: Downregulation of miR-181b-5p Inhibits the Viability, Migration, and Glycolysis of Gallbladder Cancer by Upregulating PDHX Under Hypoxia
Source: Front Oncol. 2021 Aug 16;11:683725. doi: 10.3389/fonc.2021.683725 (PMC8415503; doi:10.3389/fonc.2021.683725)

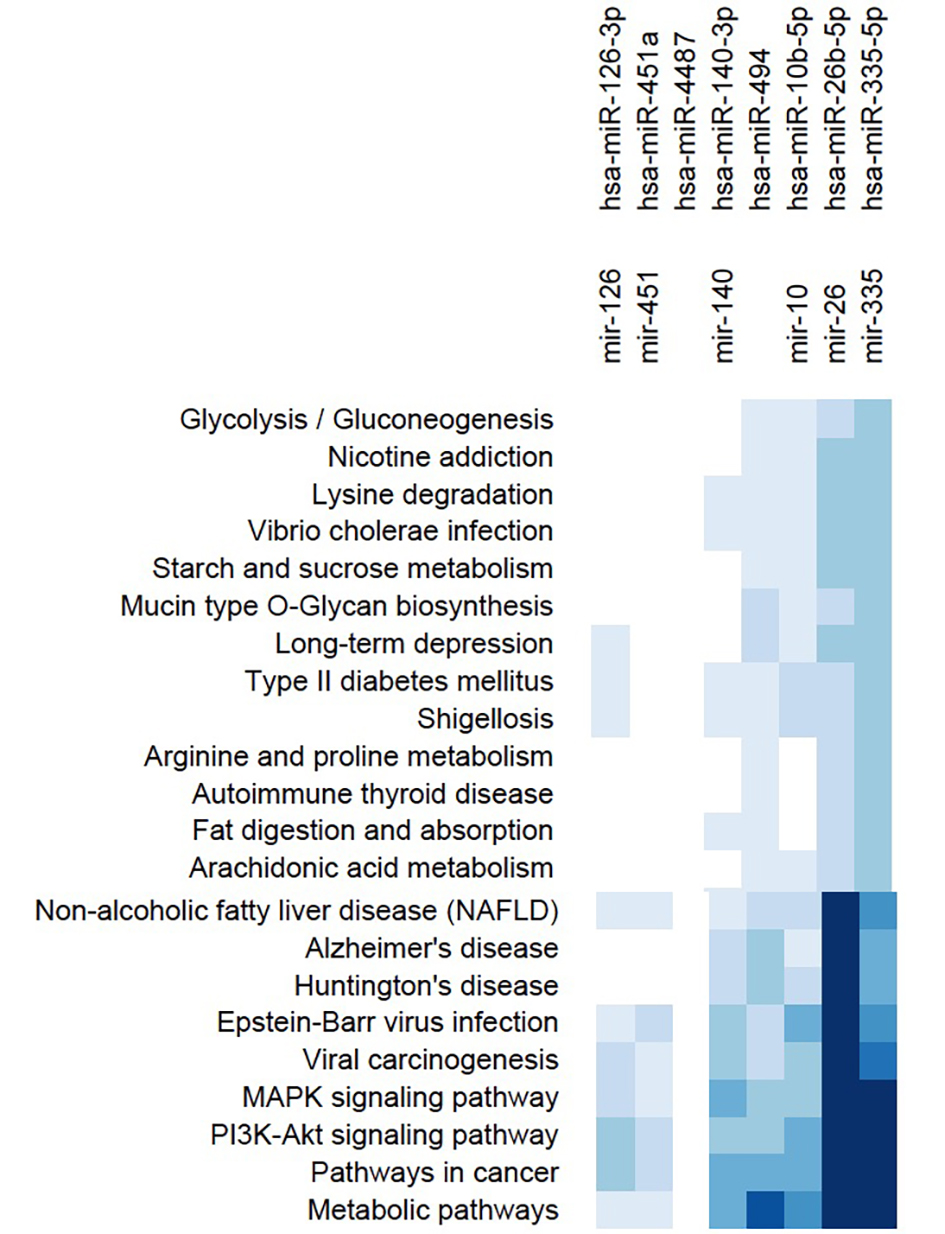

Supplement: Supplementary Figure 1 — KEGG analysis of protein targets of DEGs. KEGG analysis of protein targets of miR-126-3p, miR-451a, miR-4487, miR-140-3p, miR-494, miR-10b-5p, miR-26b-5p, miR-335-5p. [file Image_1.jpeg]

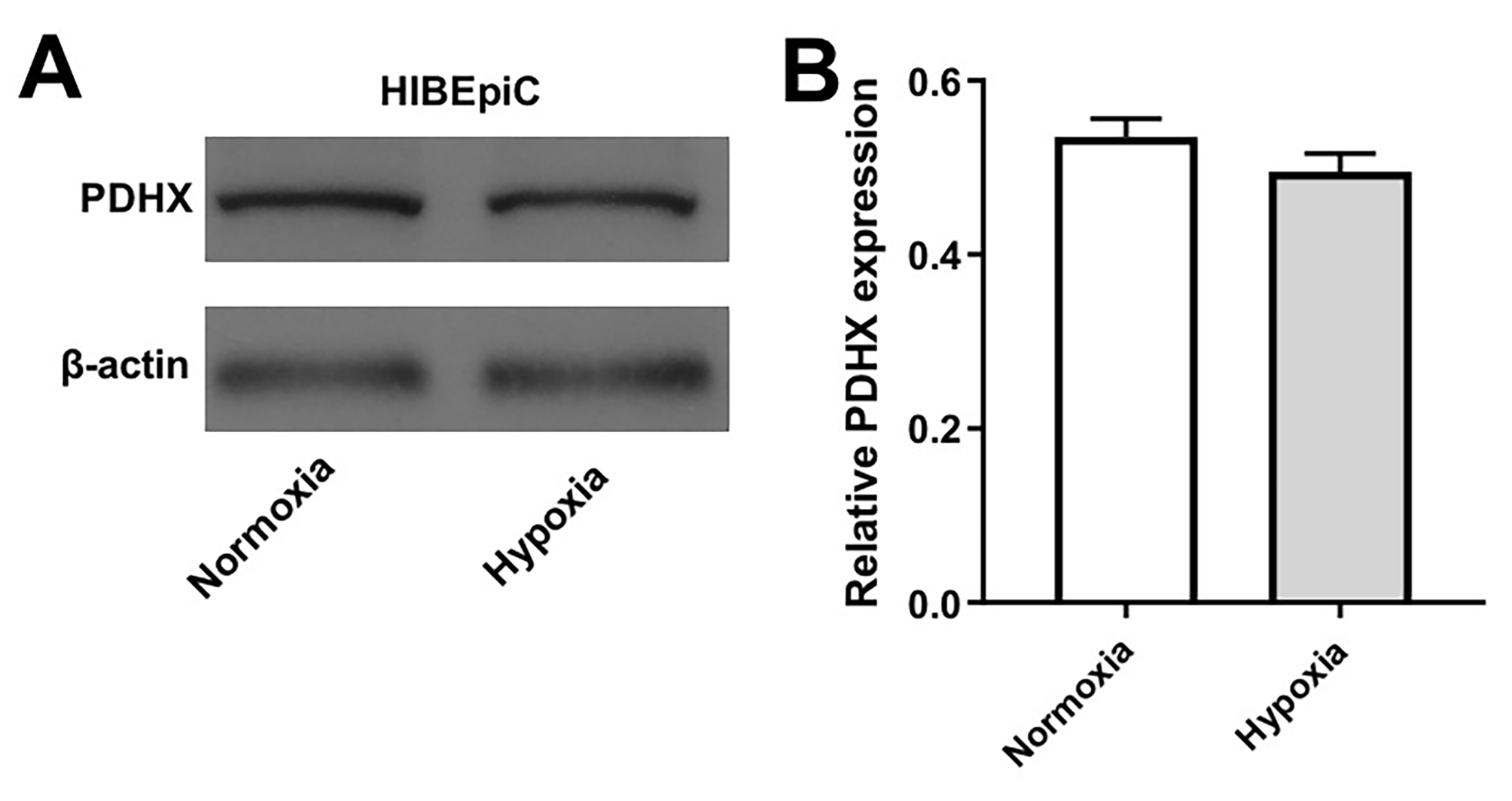

Supplement: Supplementary Figure 2 — The expression of PDHX in HIBEpiC cells under hypoxic condition. (A, B) HIBEpiC cells were treated with hypoxia or hypoxia. Western blot was performed to measure the expression of PDHX in HIBEpiC cells. [file Image_2.jpeg]

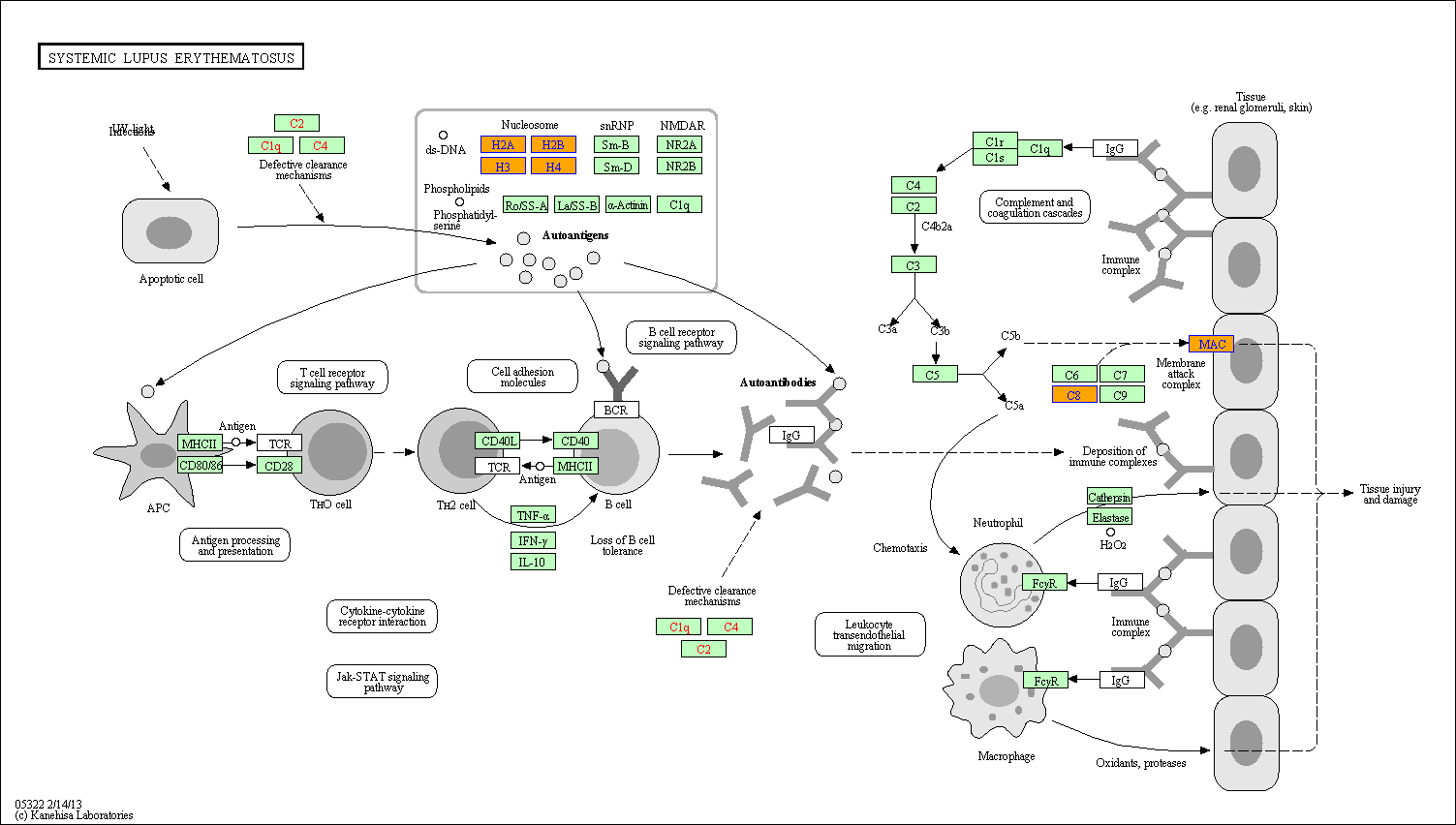

Supplement: Supplementary file 3 [file DataSheet_1.zip › RNA seq raw data/HuGene 2.0 ST Data/Pathway Analysis/A vs B_up/hsa05322.png]

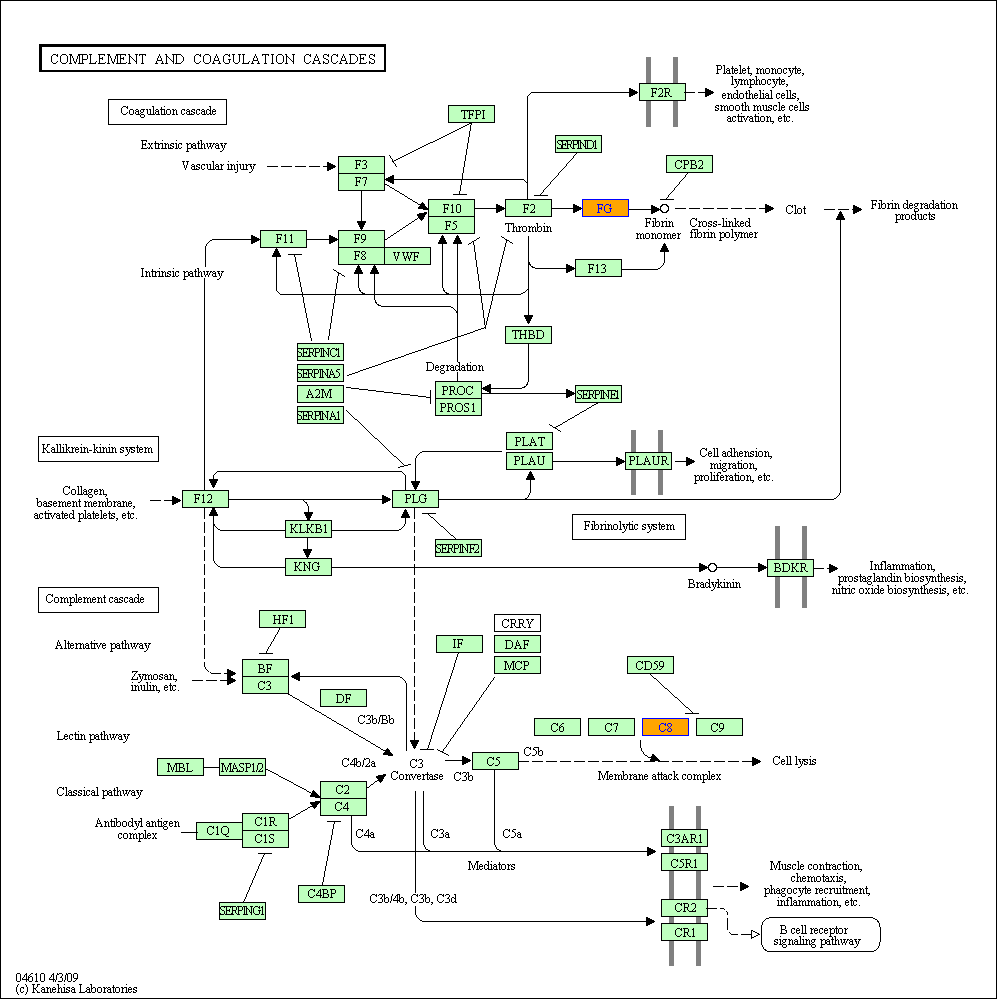

Supplement: Supplementary file 3 [file DataSheet_1.zip › RNA seq raw data/HuGene 2.0 ST Data/Pathway Analysis/A vs B_up/hsa04610.png]

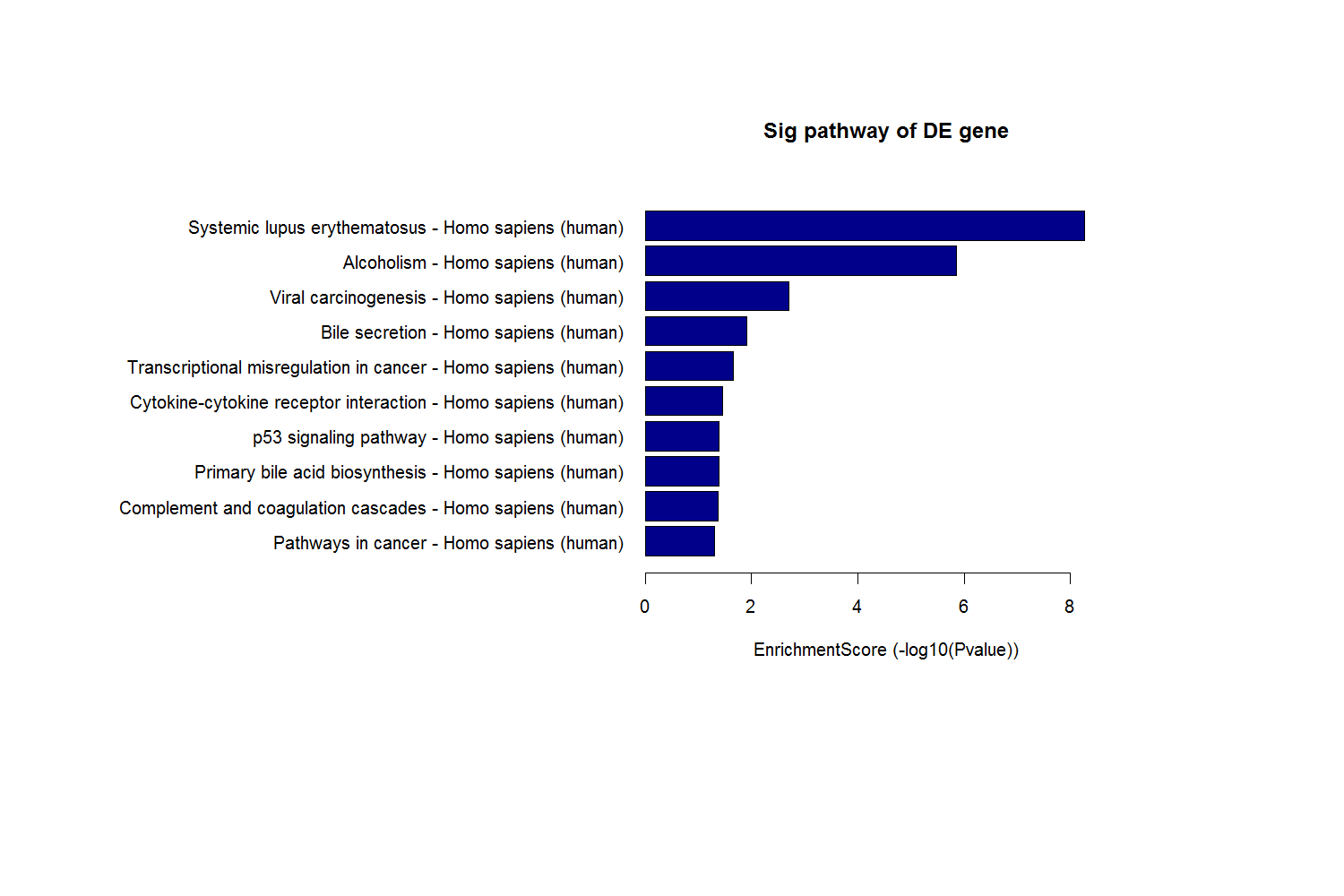

Supplement: Supplementary file 3 [file DataSheet_1.zip › RNA seq raw data/HuGene 2.0 ST Data/Pathway Analysis/A vs B_up/hsa_EnrichmentScore.png]

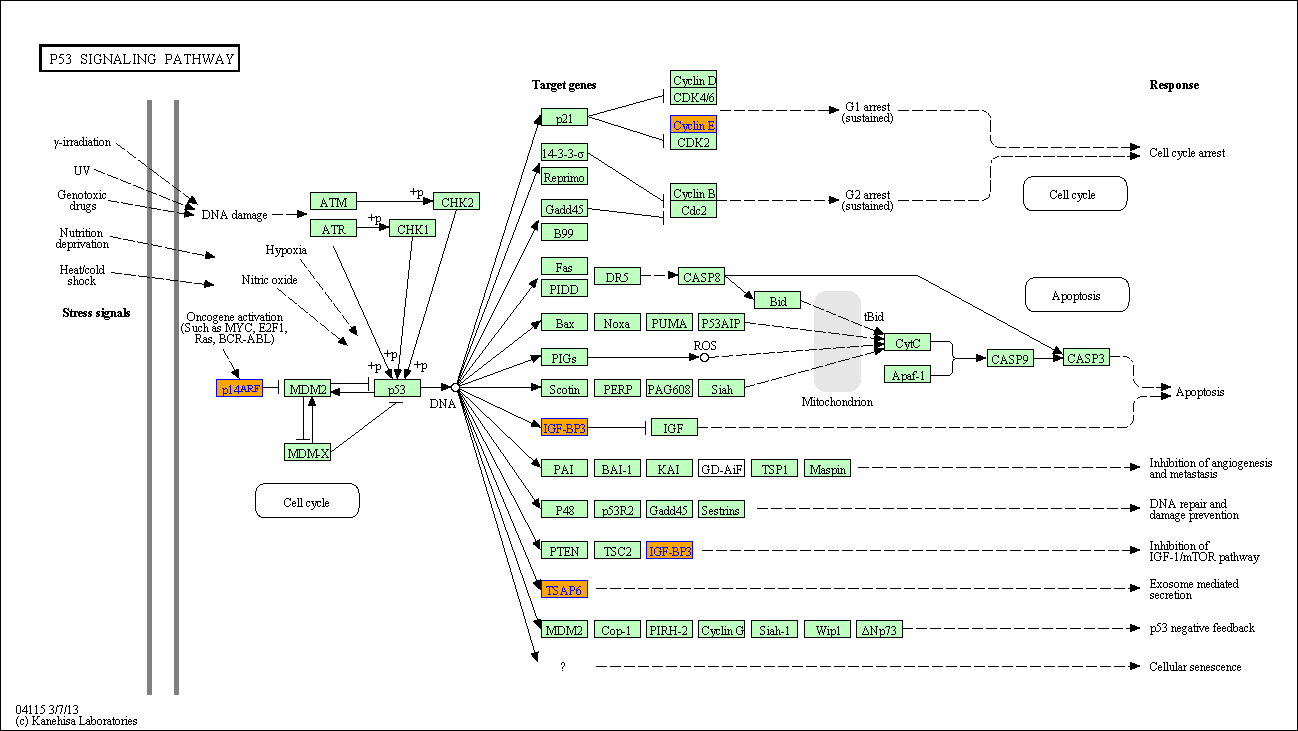

Supplement: Supplementary file 3 [file DataSheet_1.zip › RNA seq raw data/HuGene 2.0 ST Data/Pathway Analysis/A vs B_up/hsa04115.png]

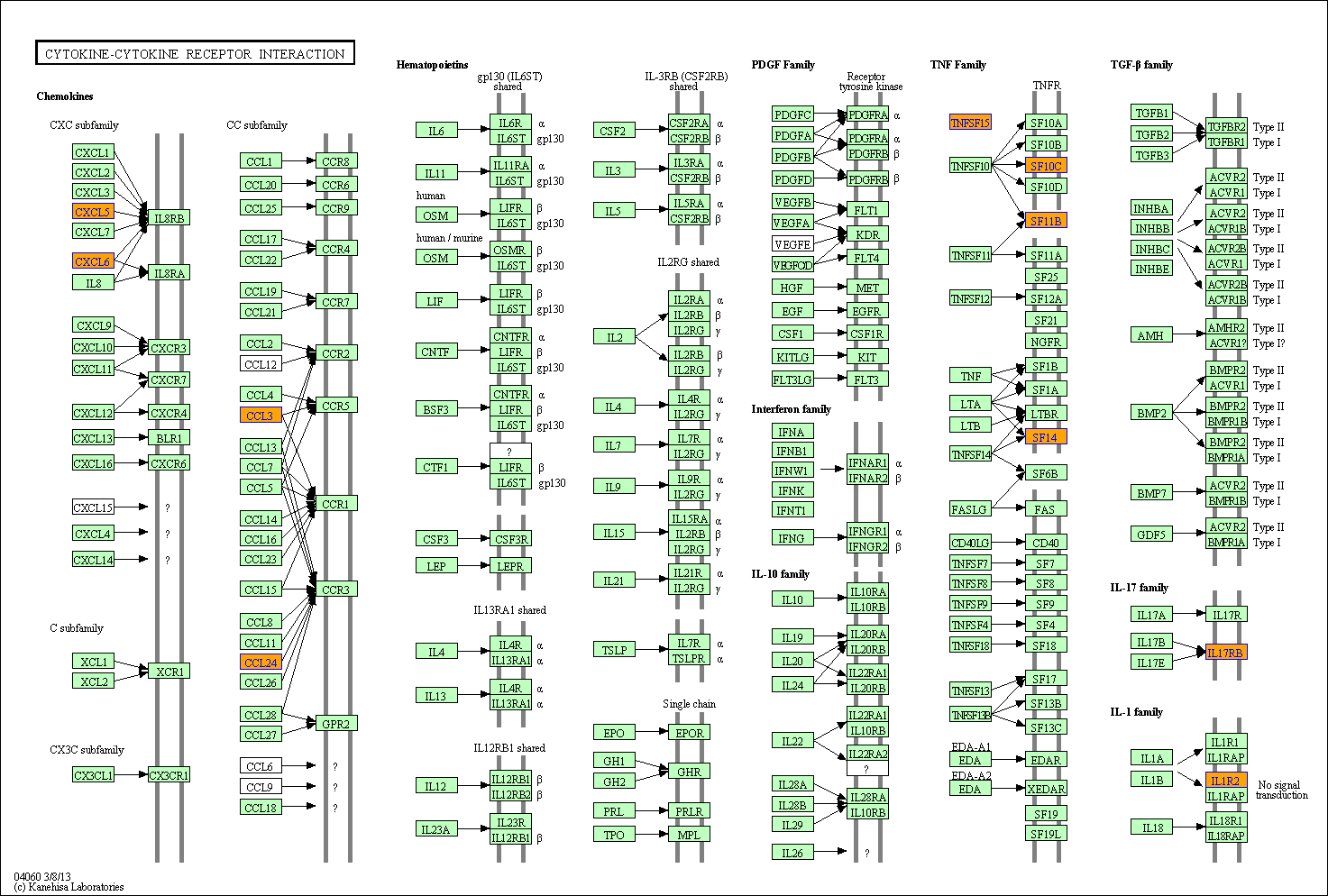

Supplement: Supplementary file 3 [file DataSheet_1.zip › RNA seq raw data/HuGene 2.0 ST Data/Pathway Analysis/A vs B_up/hsa04060.png]

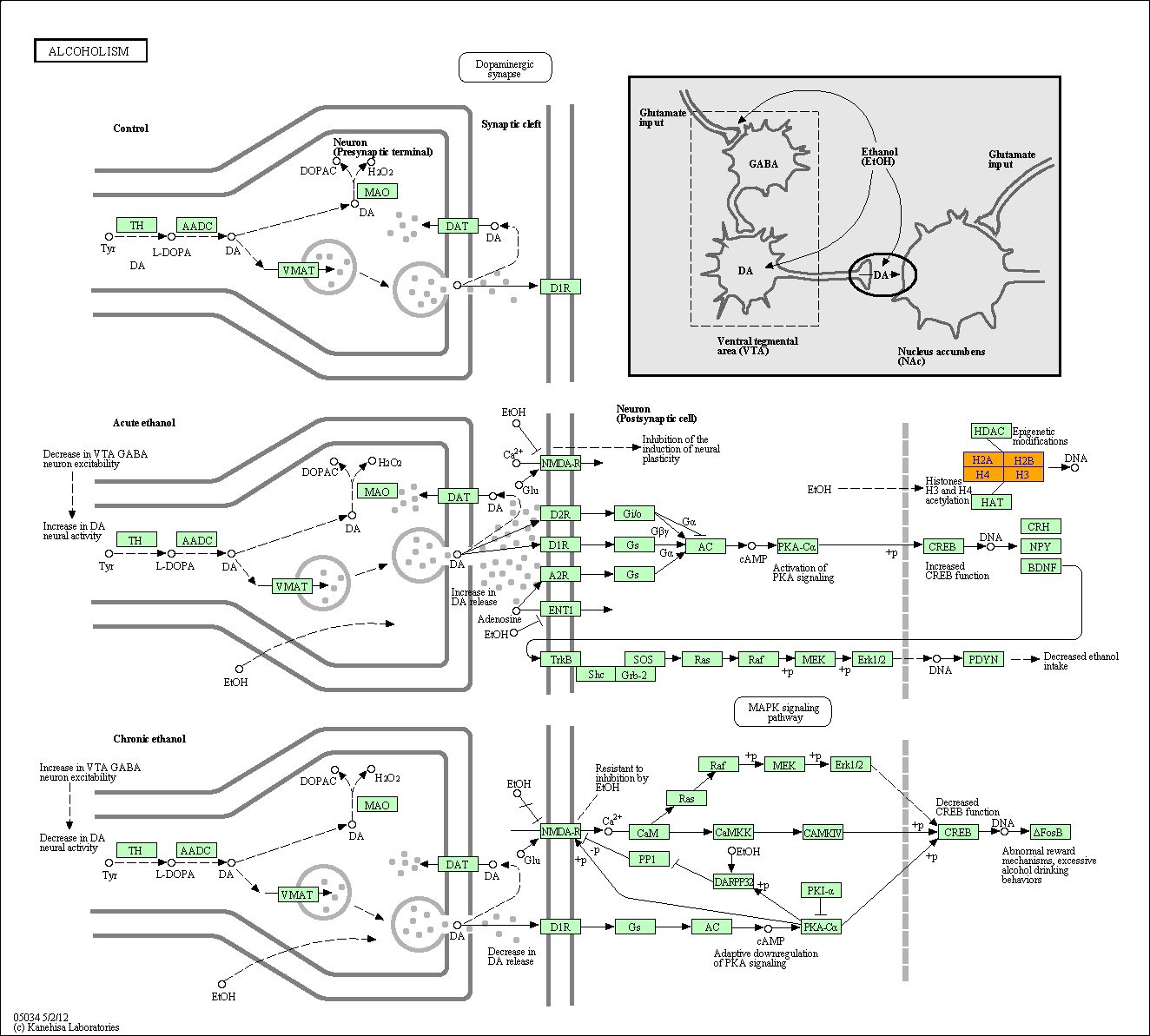

Supplement: Supplementary file 3 [file DataSheet_1.zip › RNA seq raw data/HuGene 2.0 ST Data/Pathway Analysis/A vs B_up/hsa05034.png]

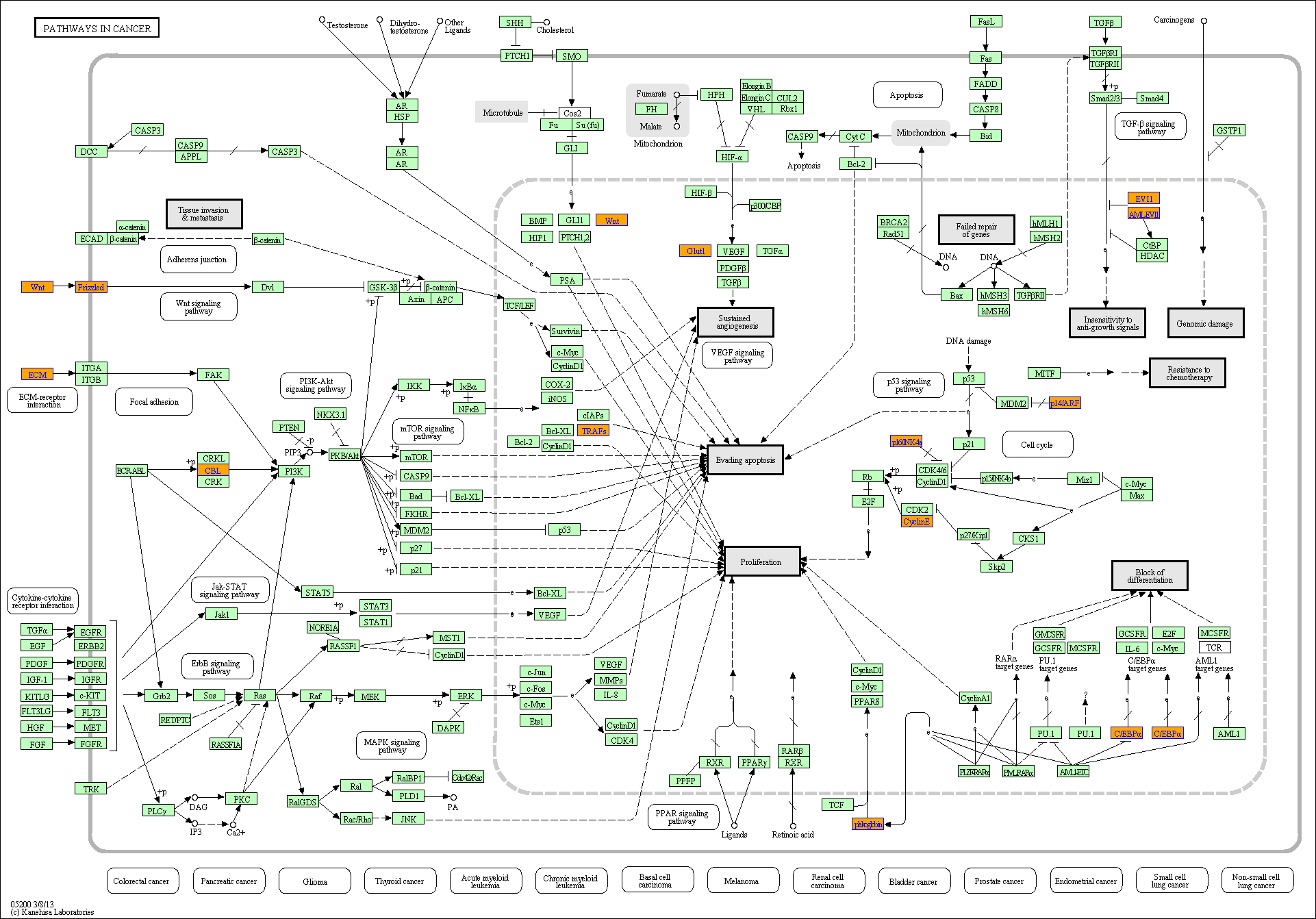

Supplement: Supplementary file 3 [file DataSheet_1.zip › RNA seq raw data/HuGene 2.0 ST Data/Pathway Analysis/A vs B_up/hsa05200.png]

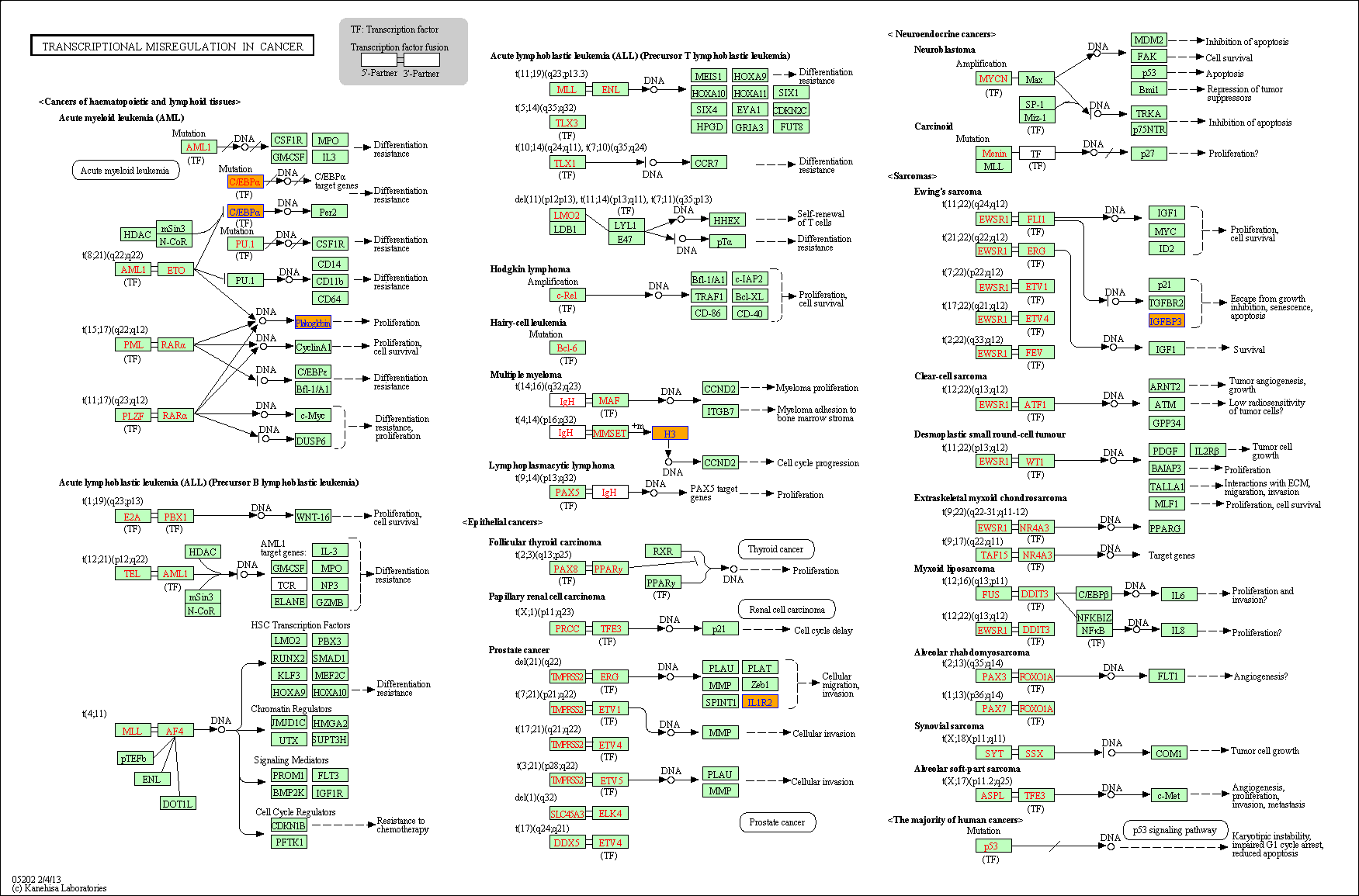

Supplement: Supplementary file 3 [file DataSheet_1.zip › RNA seq raw data/HuGene 2.0 ST Data/Pathway Analysis/A vs B_up/hsa05202.png]

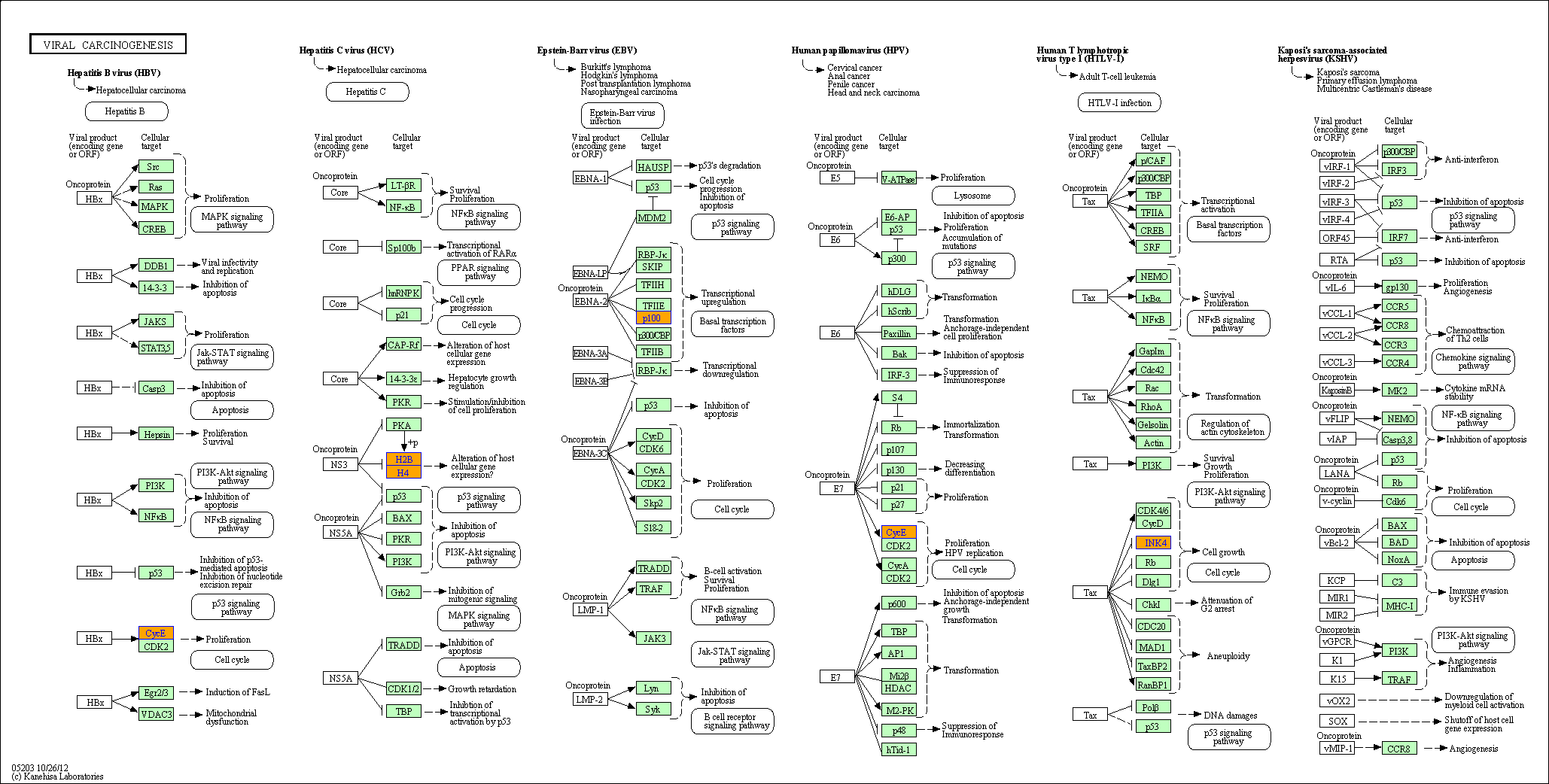

Supplement: Supplementary file 3 [file DataSheet_1.zip › RNA seq raw data/HuGene 2.0 ST Data/Pathway Analysis/A vs B_up/hsa05203.png]

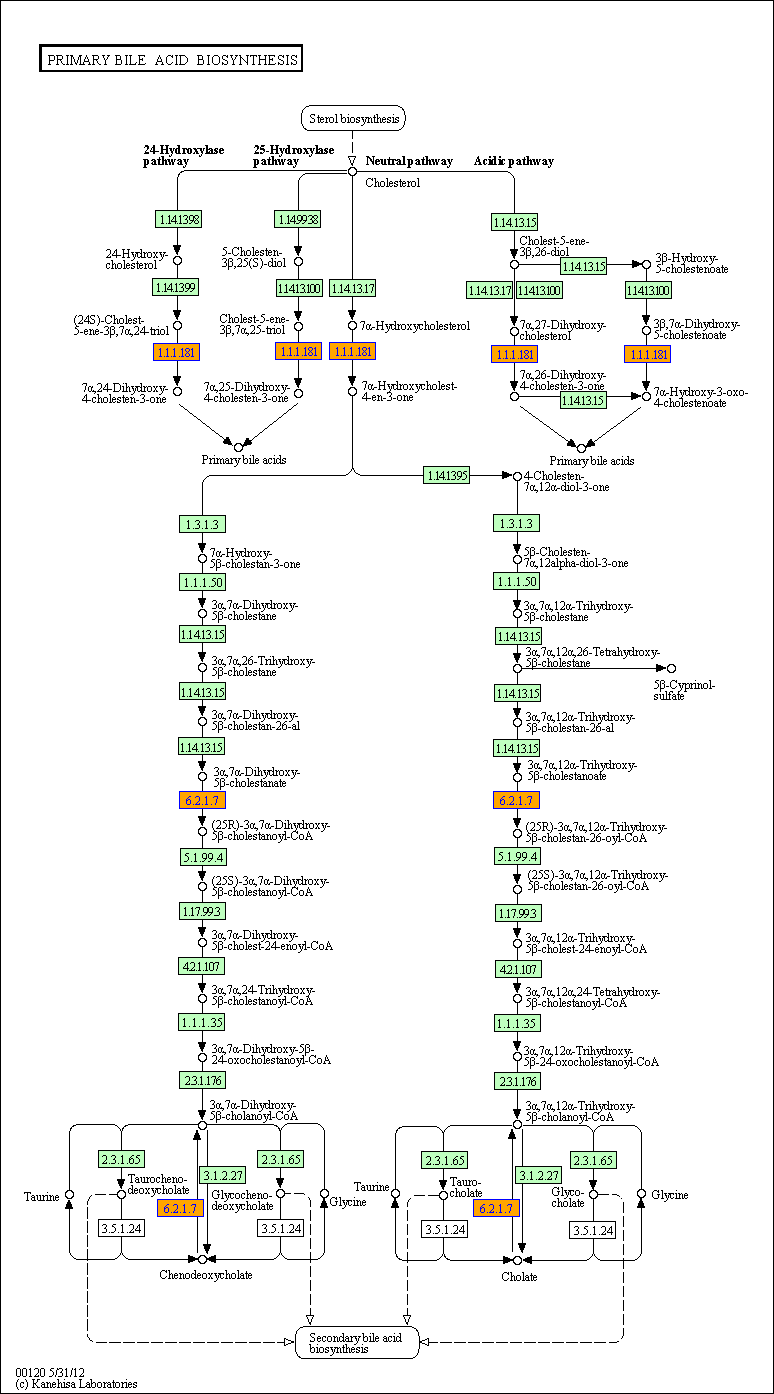

Supplement: Supplementary file 3 [file DataSheet_1.zip › RNA seq raw data/HuGene 2.0 ST Data/Pathway Analysis/A vs B_up/hsa00120.png]

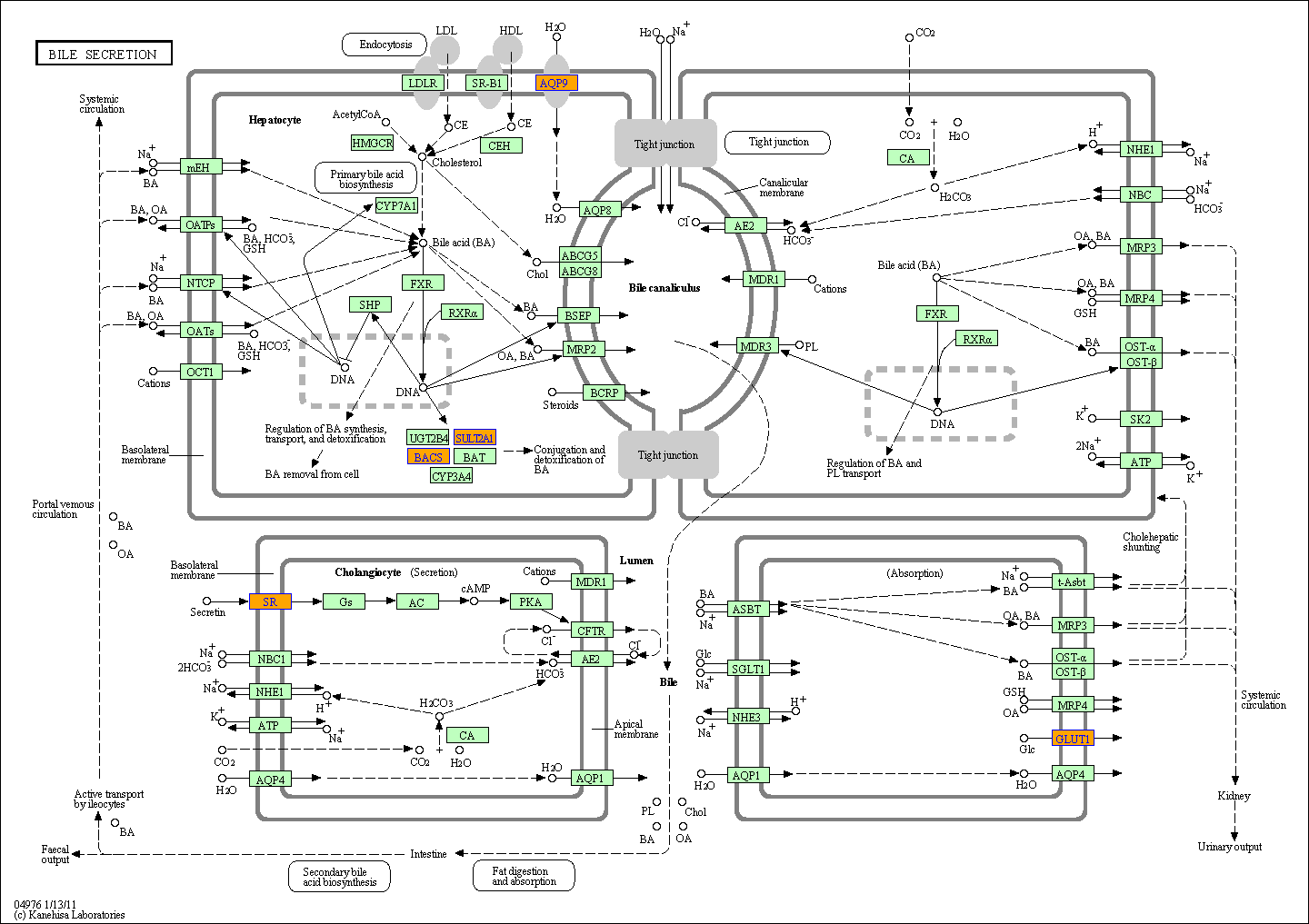

Supplement: Supplementary file 3 [file DataSheet_1.zip › RNA seq raw data/HuGene 2.0 ST Data/Pathway Analysis/A vs B_up/hsa04976.png]

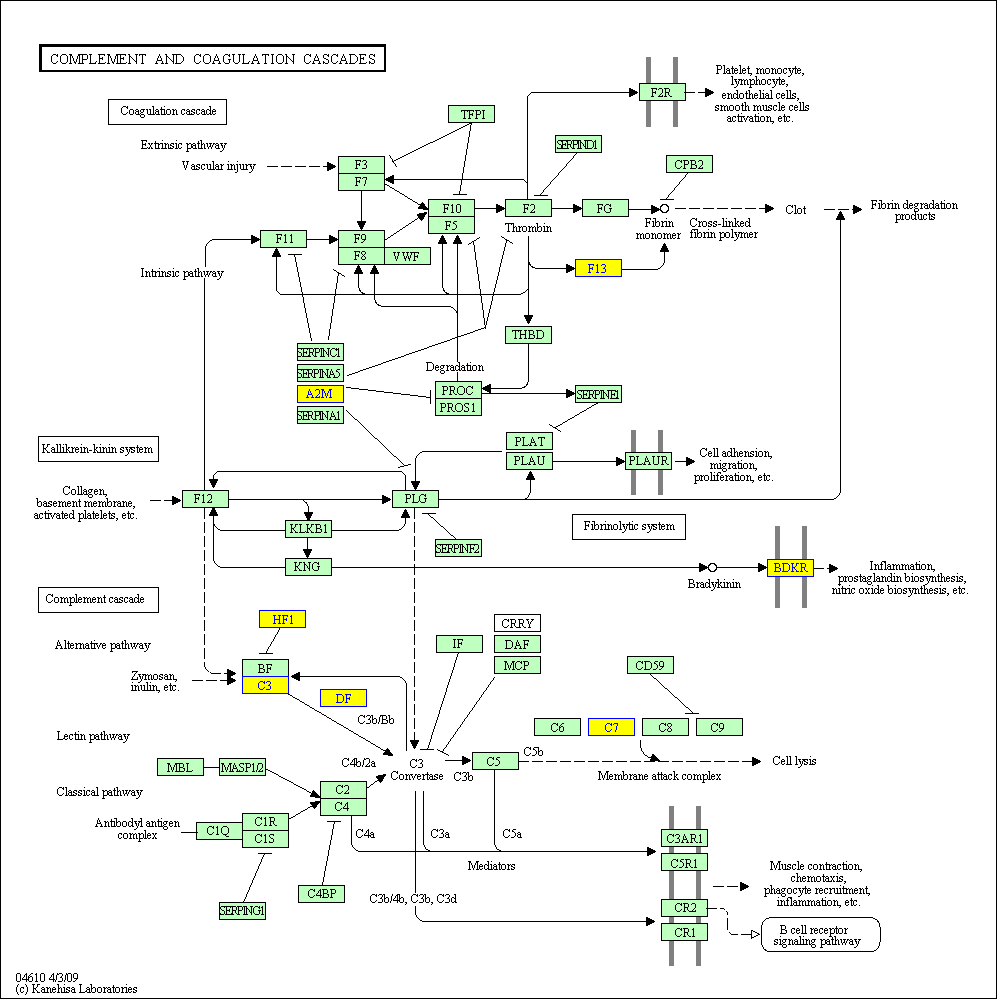

Supplement: Supplementary file 3 [file DataSheet_1.zip › RNA seq raw data/HuGene 2.0 ST Data/Pathway Analysis/A vs B_down/hsa04610.png]

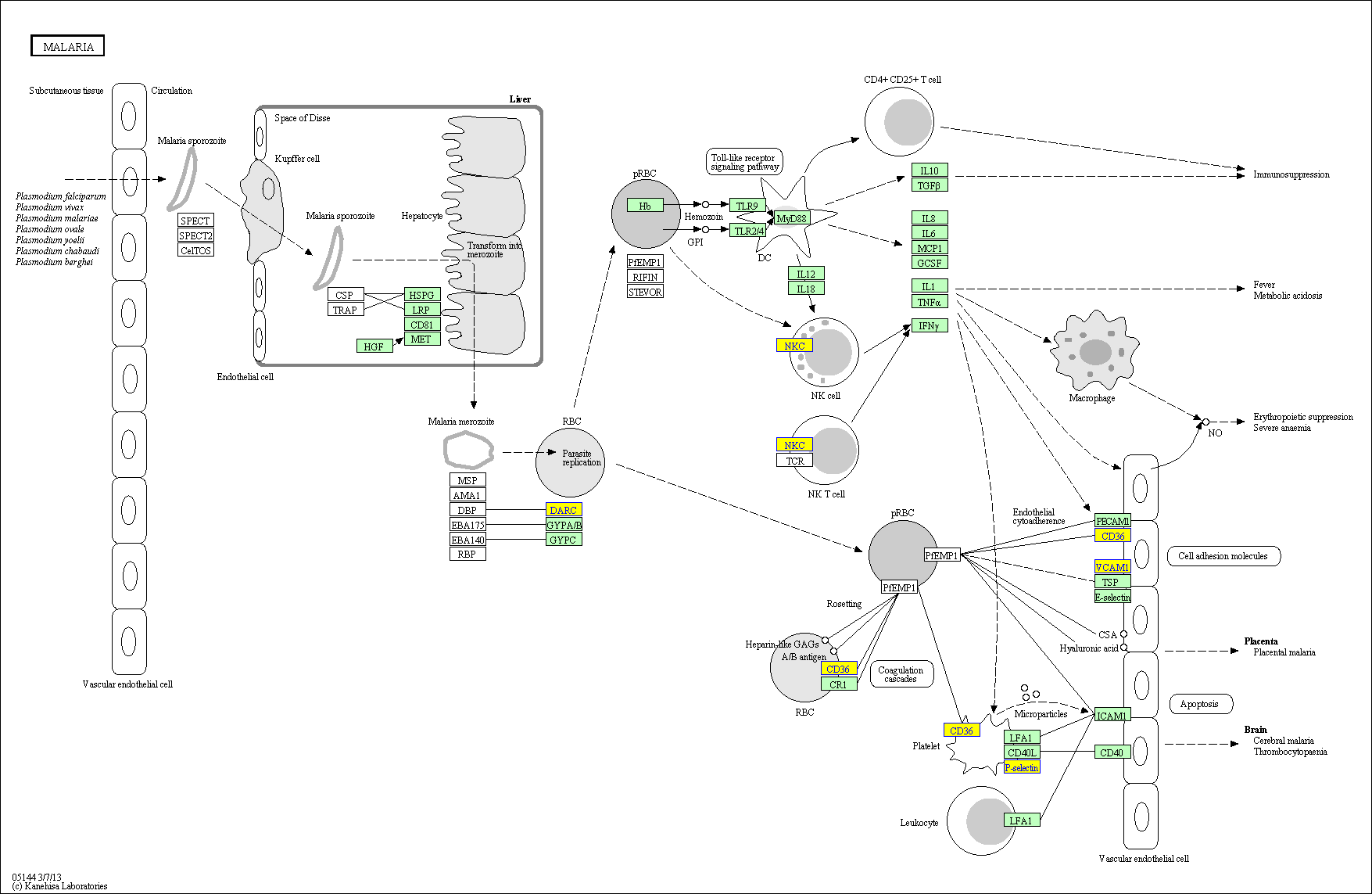

Supplement: Supplementary file 3 [file DataSheet_1.zip › RNA seq raw data/HuGene 2.0 ST Data/Pathway Analysis/A vs B_down/hsa05144.png]

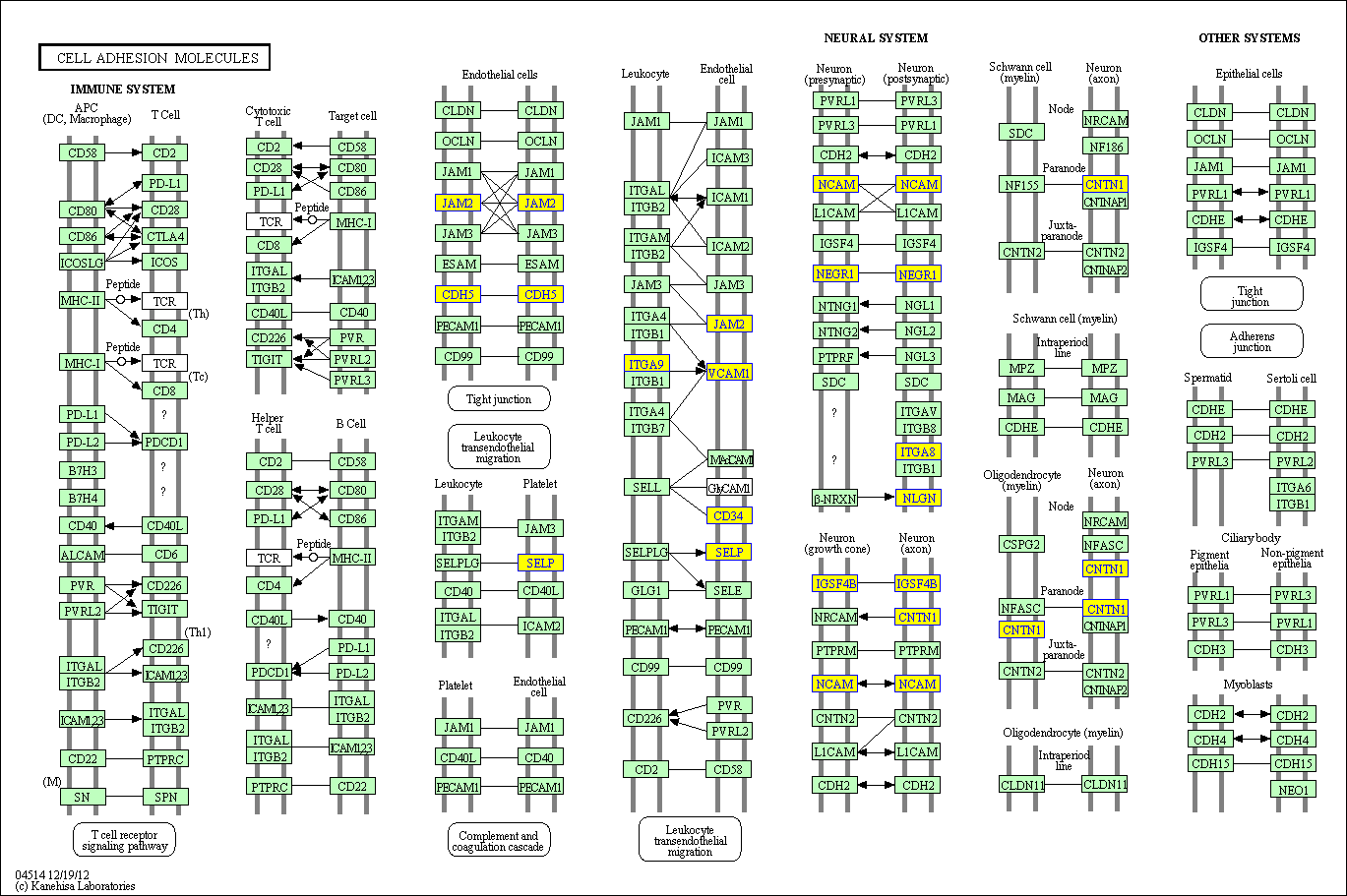

Supplement: Supplementary file 3 [file DataSheet_1.zip › RNA seq raw data/HuGene 2.0 ST Data/Pathway Analysis/A vs B_down/hsa04514.png]

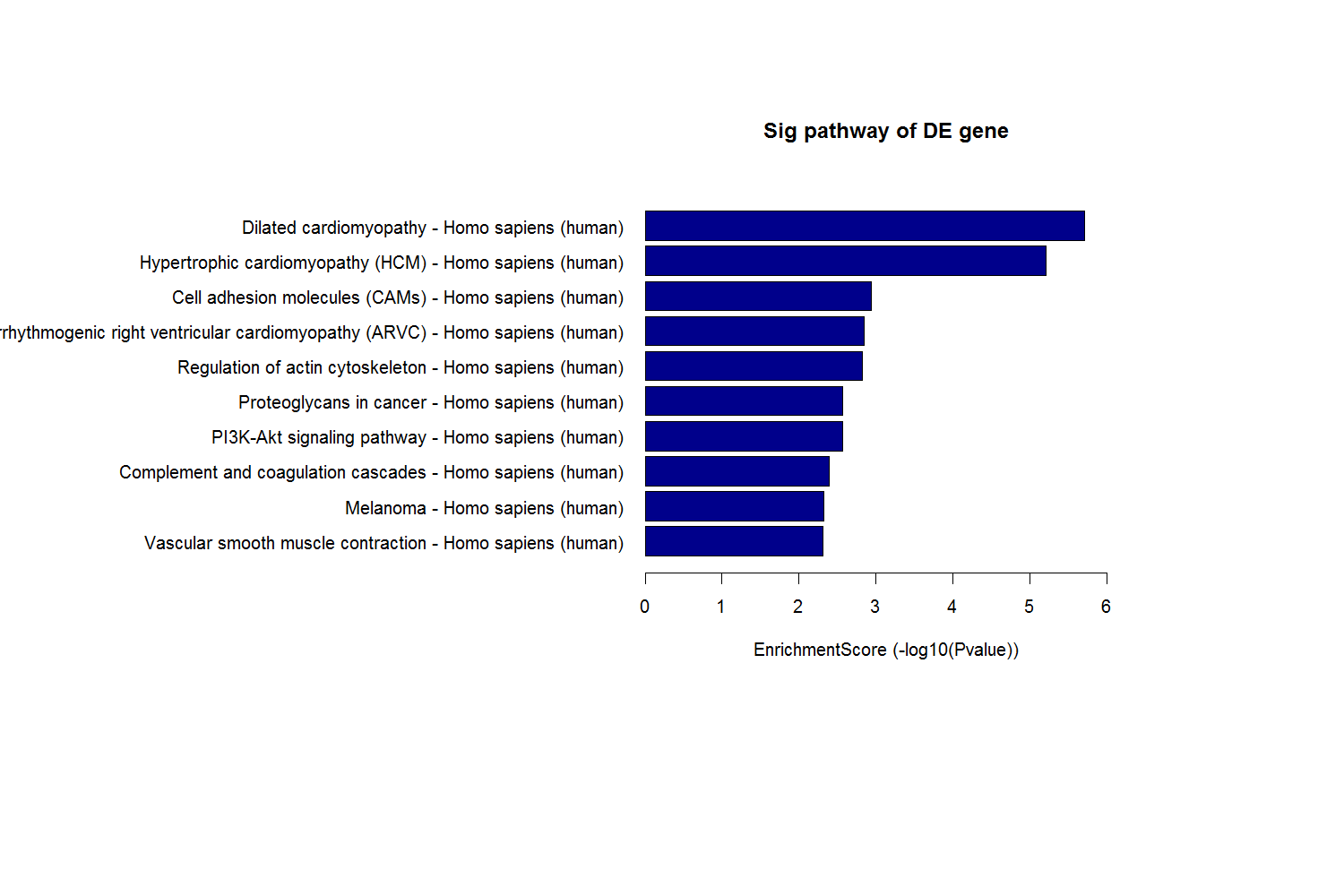

Supplement: Supplementary file 3 [file DataSheet_1.zip › RNA seq raw data/HuGene 2.0 ST Data/Pathway Analysis/A vs B_down/hsa_EnrichmentScore.png]

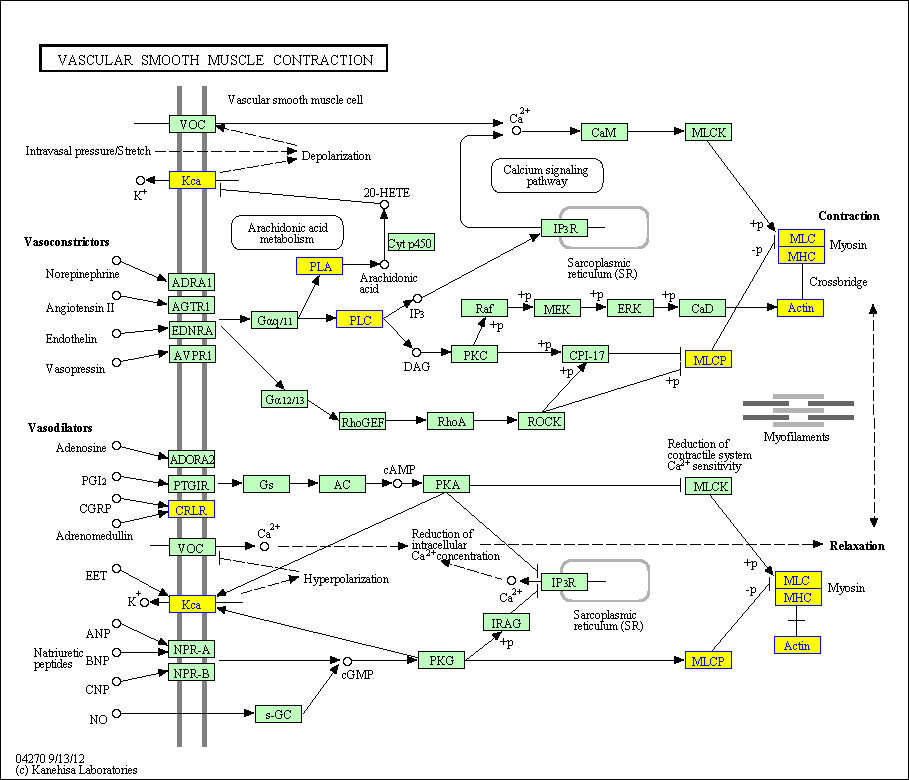

Supplement: Supplementary file 3 [file DataSheet_1.zip › RNA seq raw data/HuGene 2.0 ST Data/Pathway Analysis/A vs B_down/hsa04270.png]

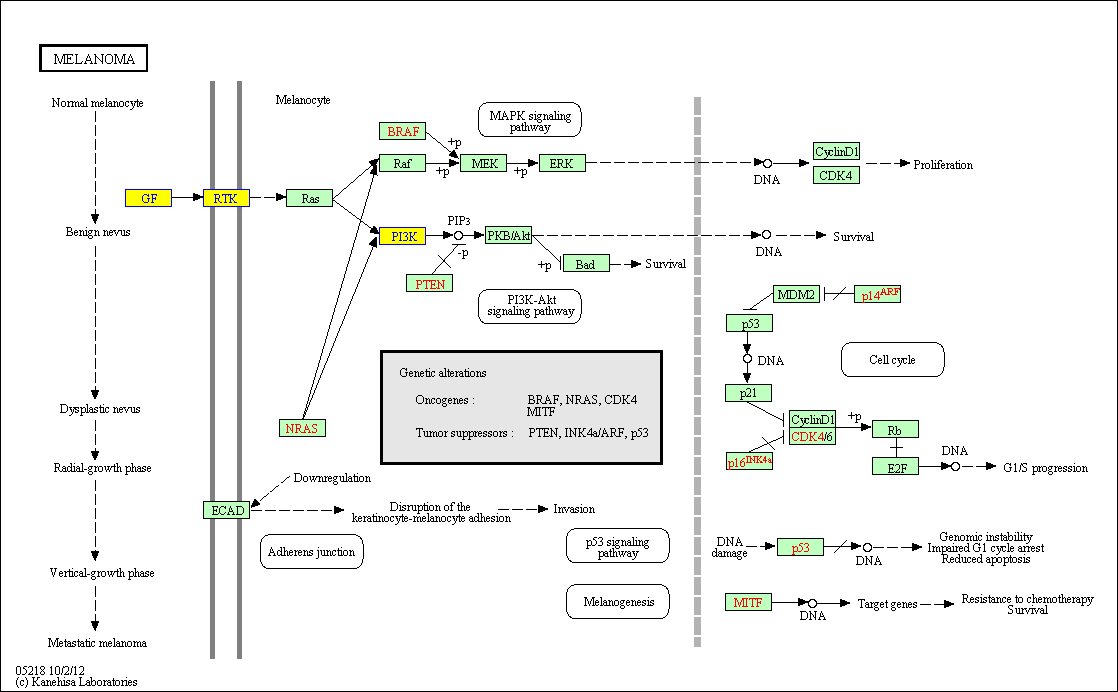

Supplement: Supplementary file 3 [file DataSheet_1.zip › RNA seq raw data/HuGene 2.0 ST Data/Pathway Analysis/A vs B_down/hsa05218.png]

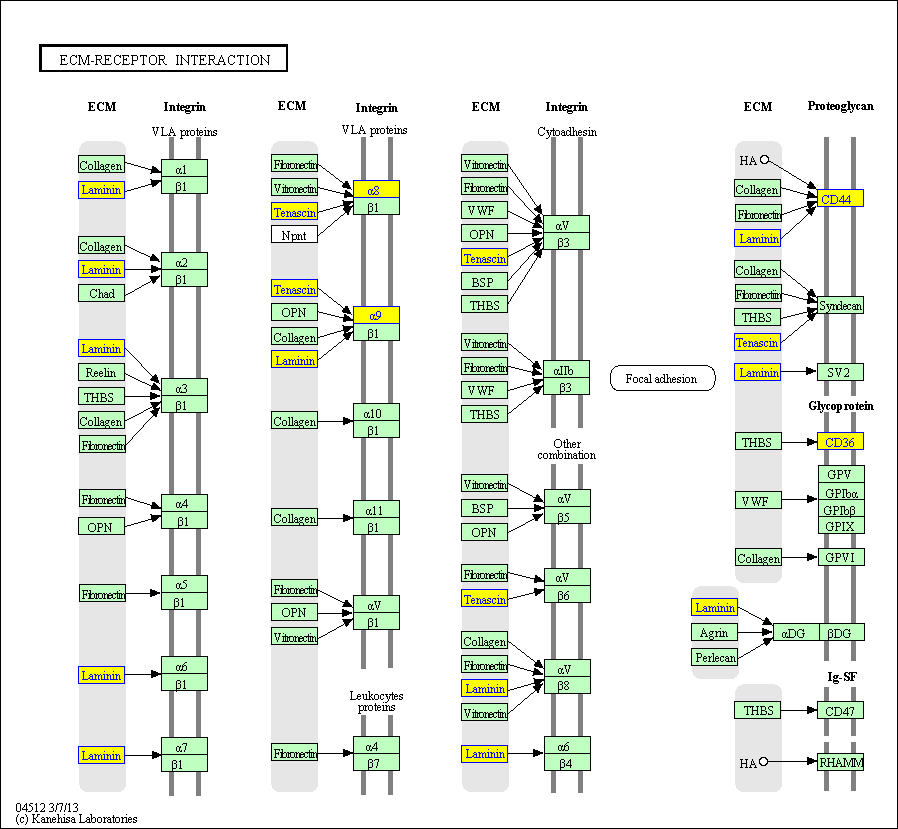

Supplement: Supplementary file 3 [file DataSheet_1.zip › RNA seq raw data/HuGene 2.0 ST Data/Pathway Analysis/A vs B_down/hsa04512.png]

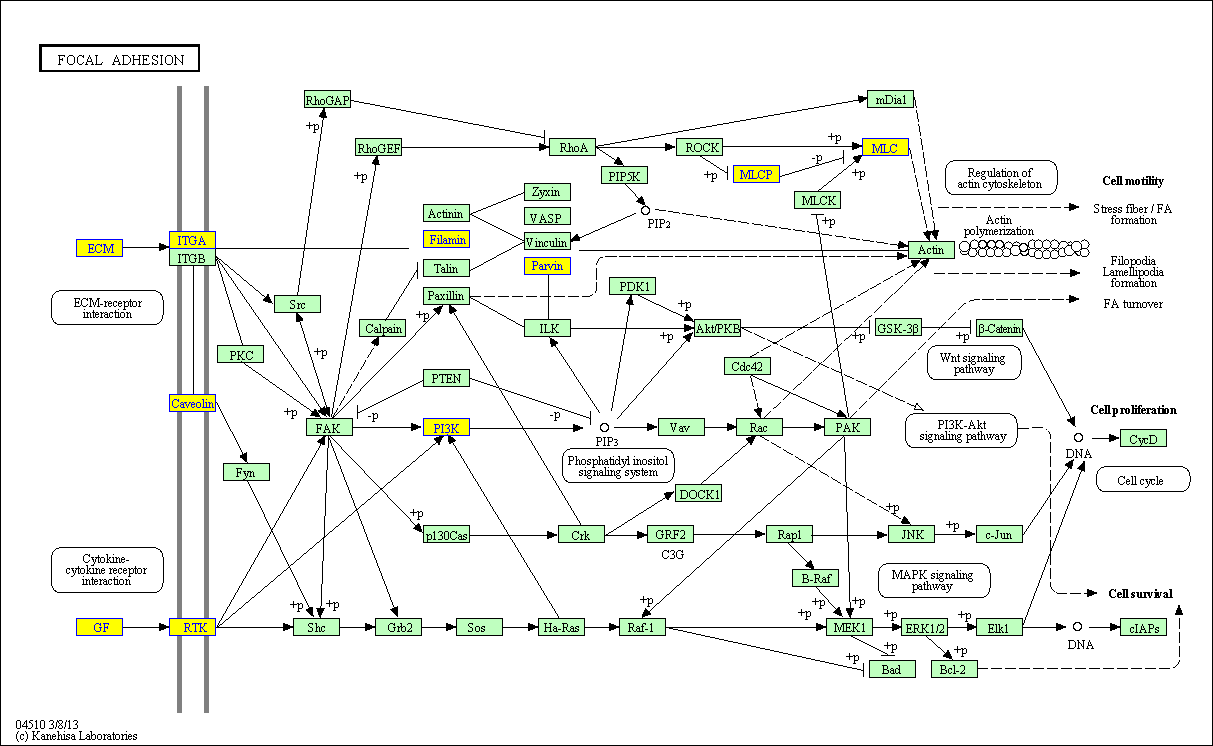

Supplement: Supplementary file 3 [file DataSheet_1.zip › RNA seq raw data/HuGene 2.0 ST Data/Pathway Analysis/A vs B_down/hsa04510.png]

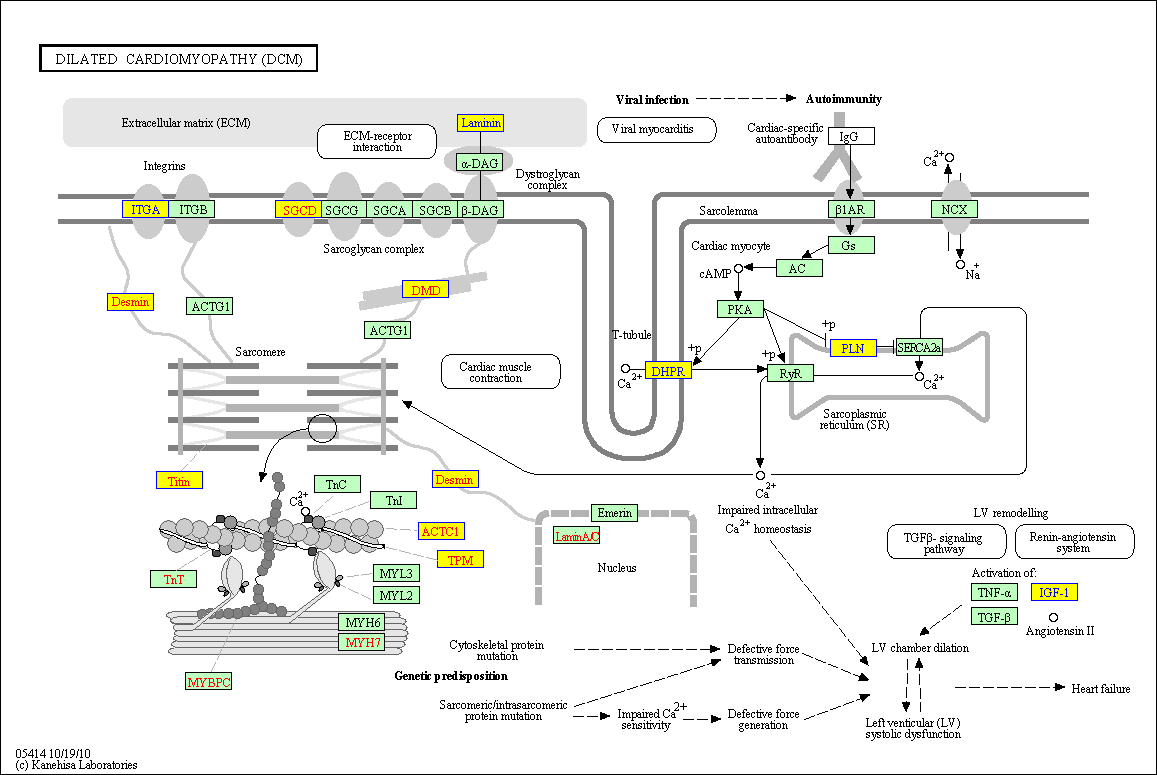

Supplement: Supplementary file 3 [file DataSheet_1.zip › RNA seq raw data/HuGene 2.0 ST Data/Pathway Analysis/A vs B_down/hsa05414.png]

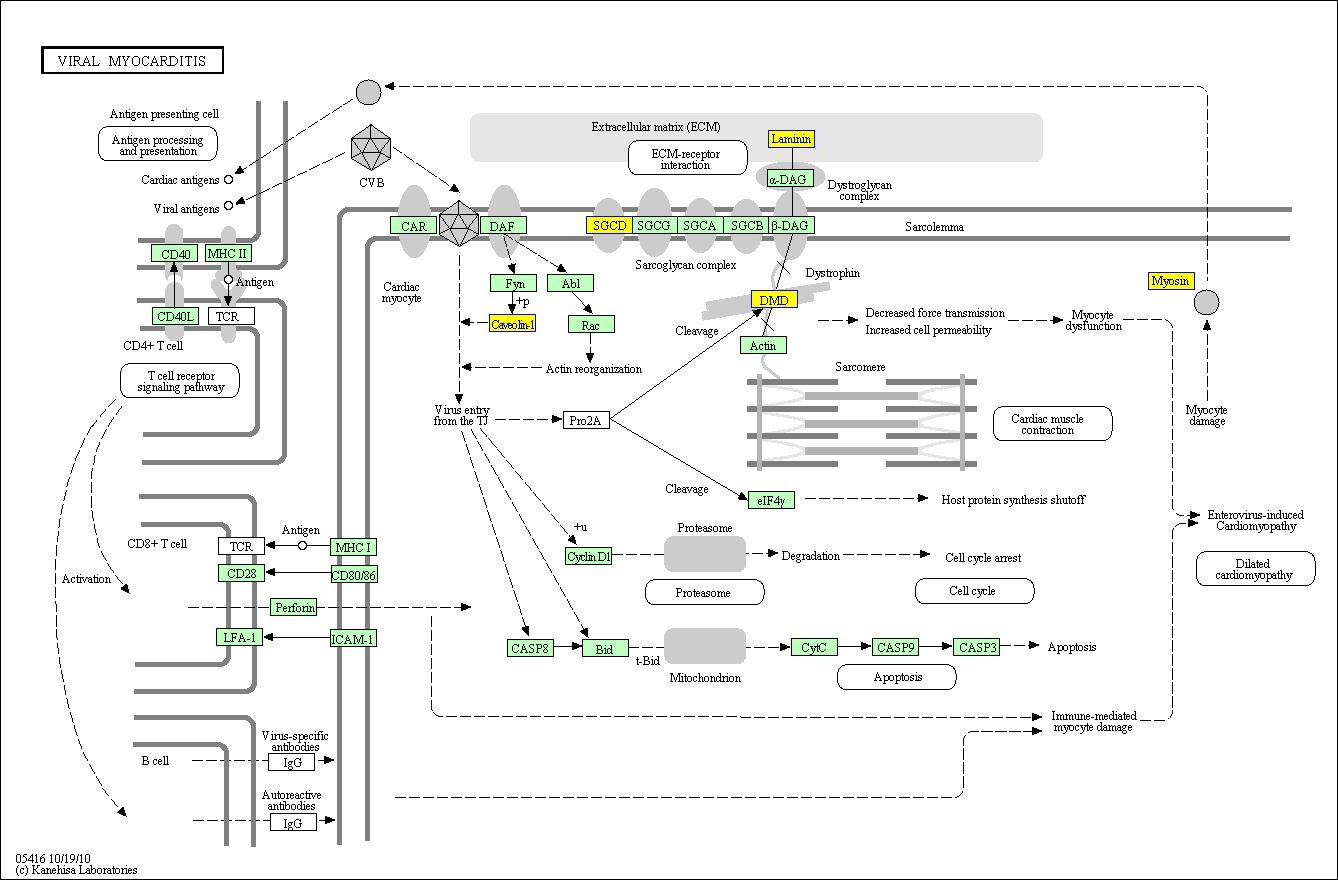

Supplement: Supplementary file 3 [file DataSheet_1.zip › RNA seq raw data/HuGene 2.0 ST Data/Pathway Analysis/A vs B_down/hsa05416.png]

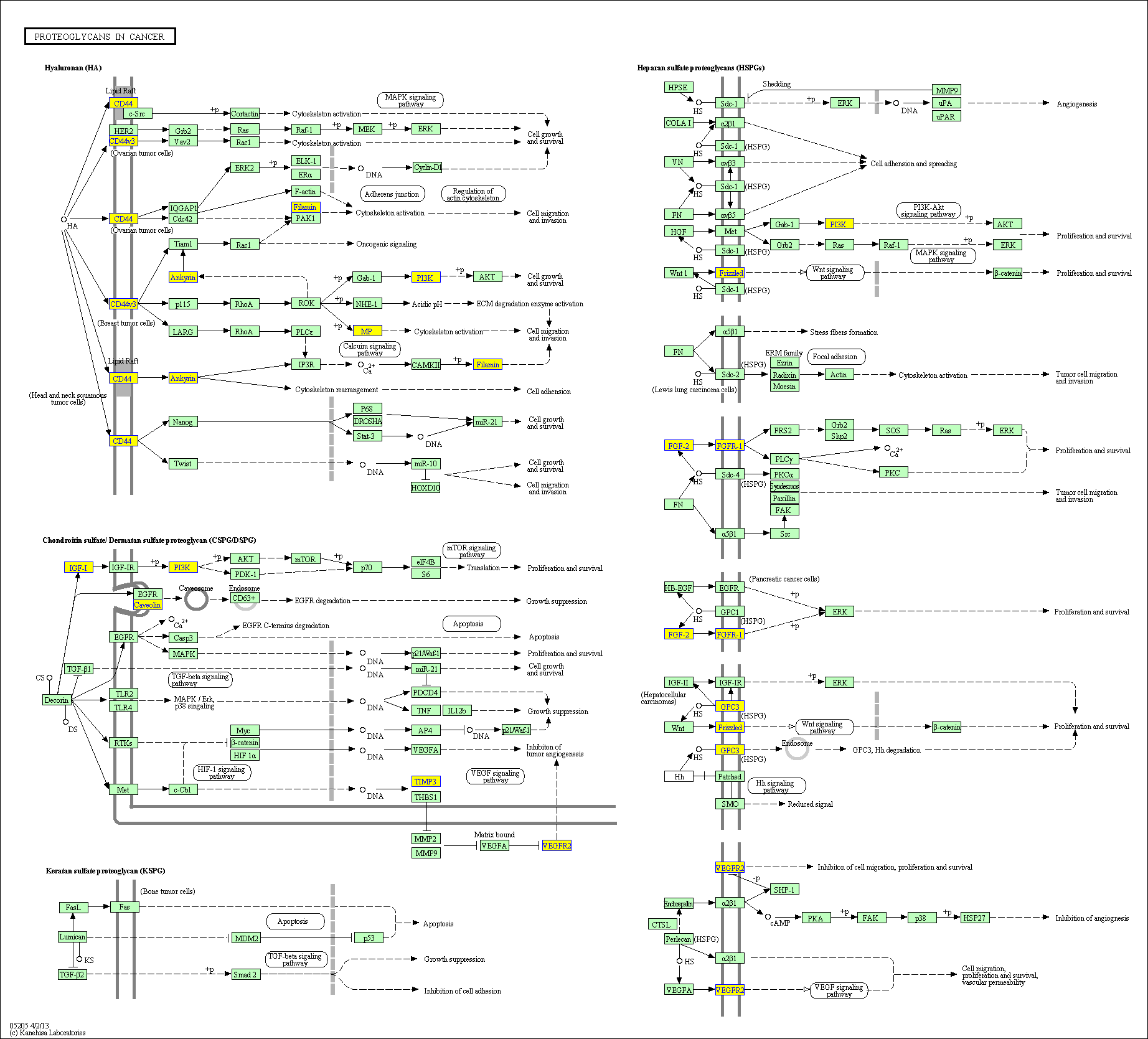

Supplement: Supplementary file 3 [file DataSheet_1.zip › RNA seq raw data/HuGene 2.0 ST Data/Pathway Analysis/A vs B_down/hsa05205.png]

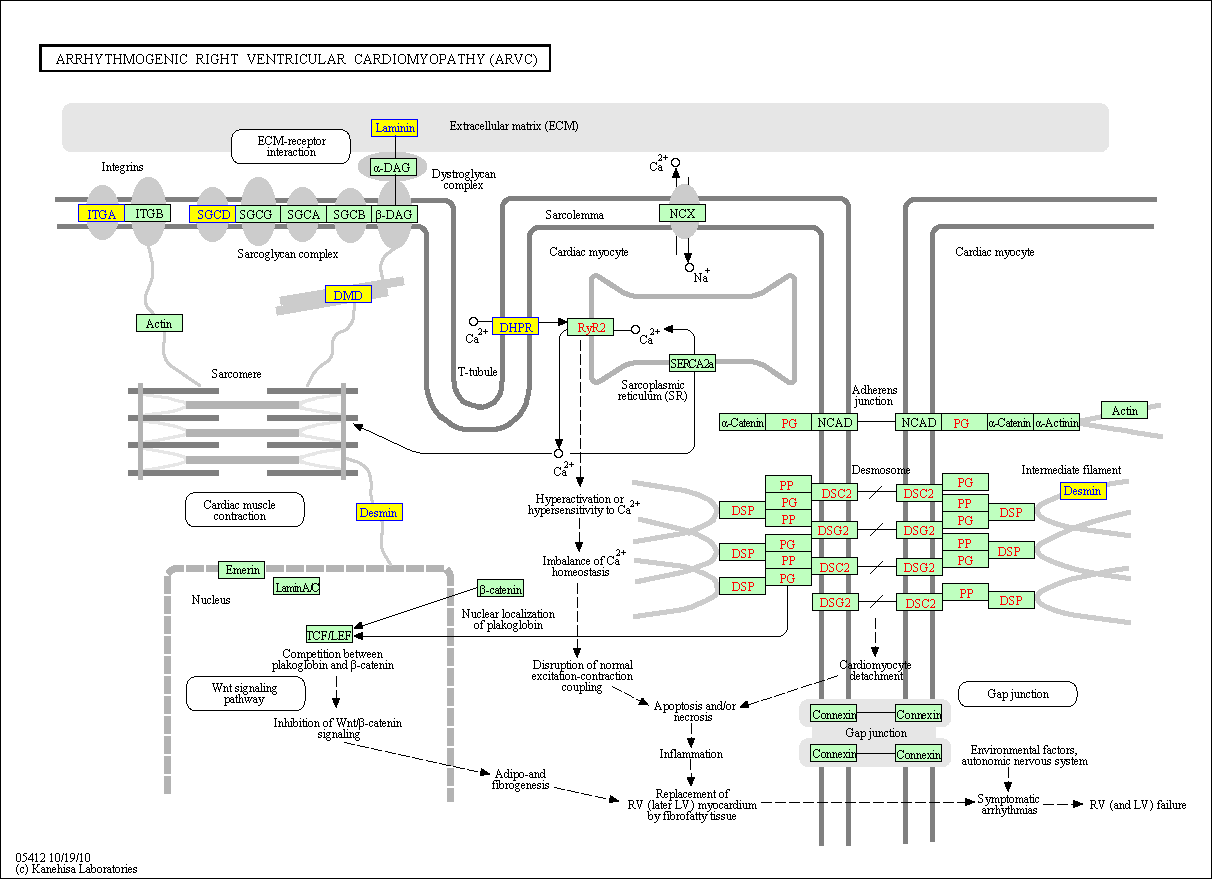

Supplement: Supplementary file 3 [file DataSheet_1.zip › RNA seq raw data/HuGene 2.0 ST Data/Pathway Analysis/A vs B_down/hsa05412.png]

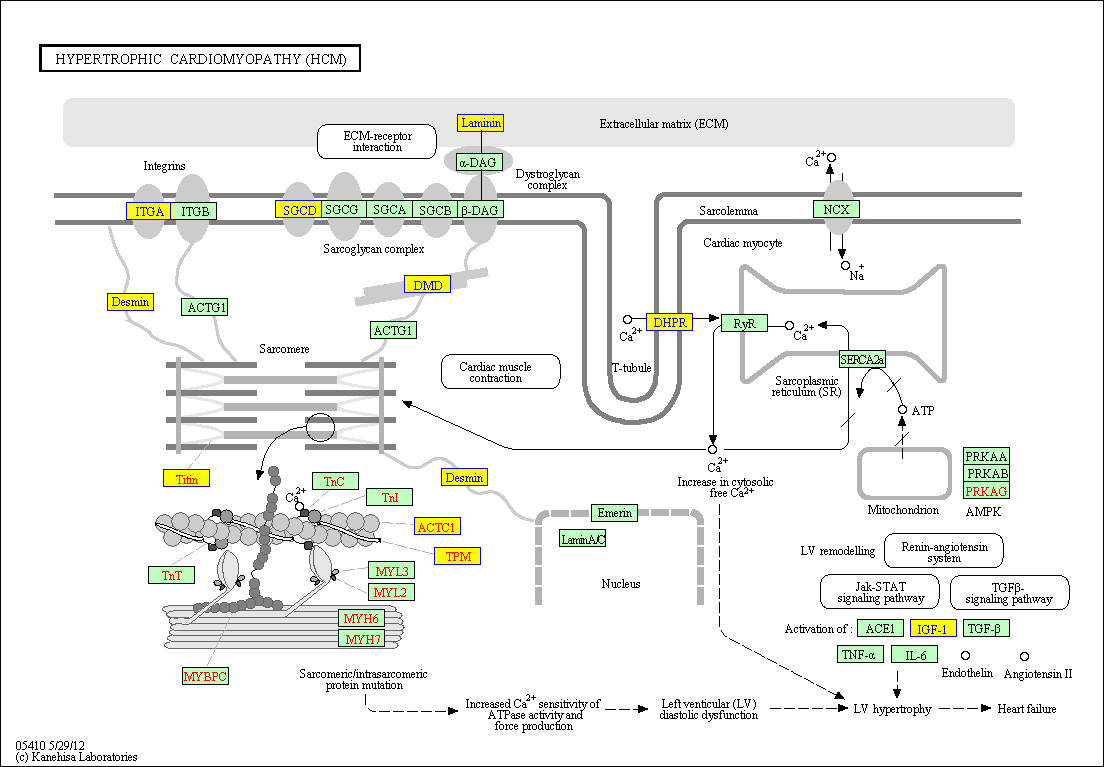

Supplement: Supplementary file 3 [file DataSheet_1.zip › RNA seq raw data/HuGene 2.0 ST Data/Pathway Analysis/A vs B_down/hsa05410.png]

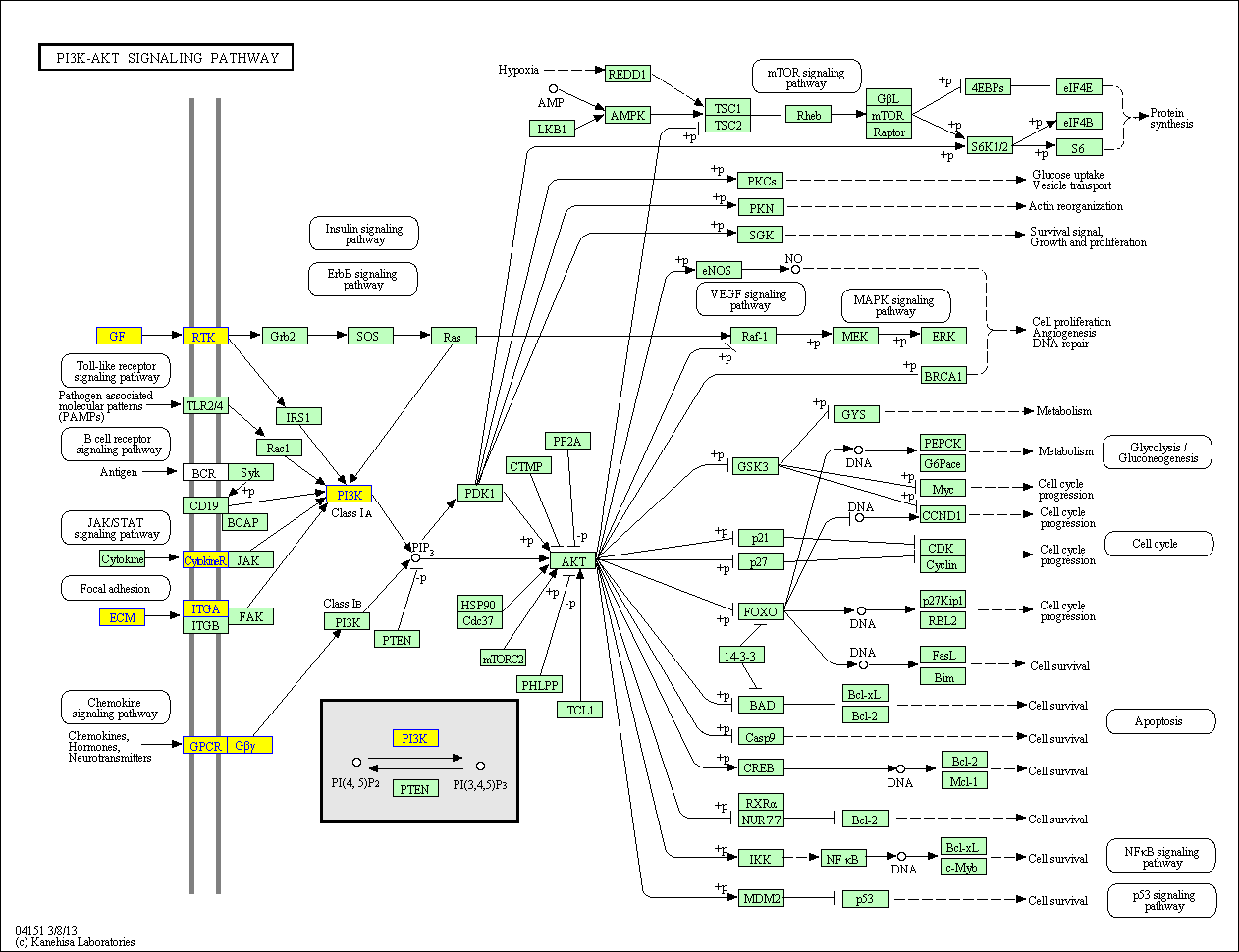

Supplement: Supplementary file 3 [file DataSheet_1.zip › RNA seq raw data/HuGene 2.0 ST Data/Pathway Analysis/A vs B_down/hsa04151.png]

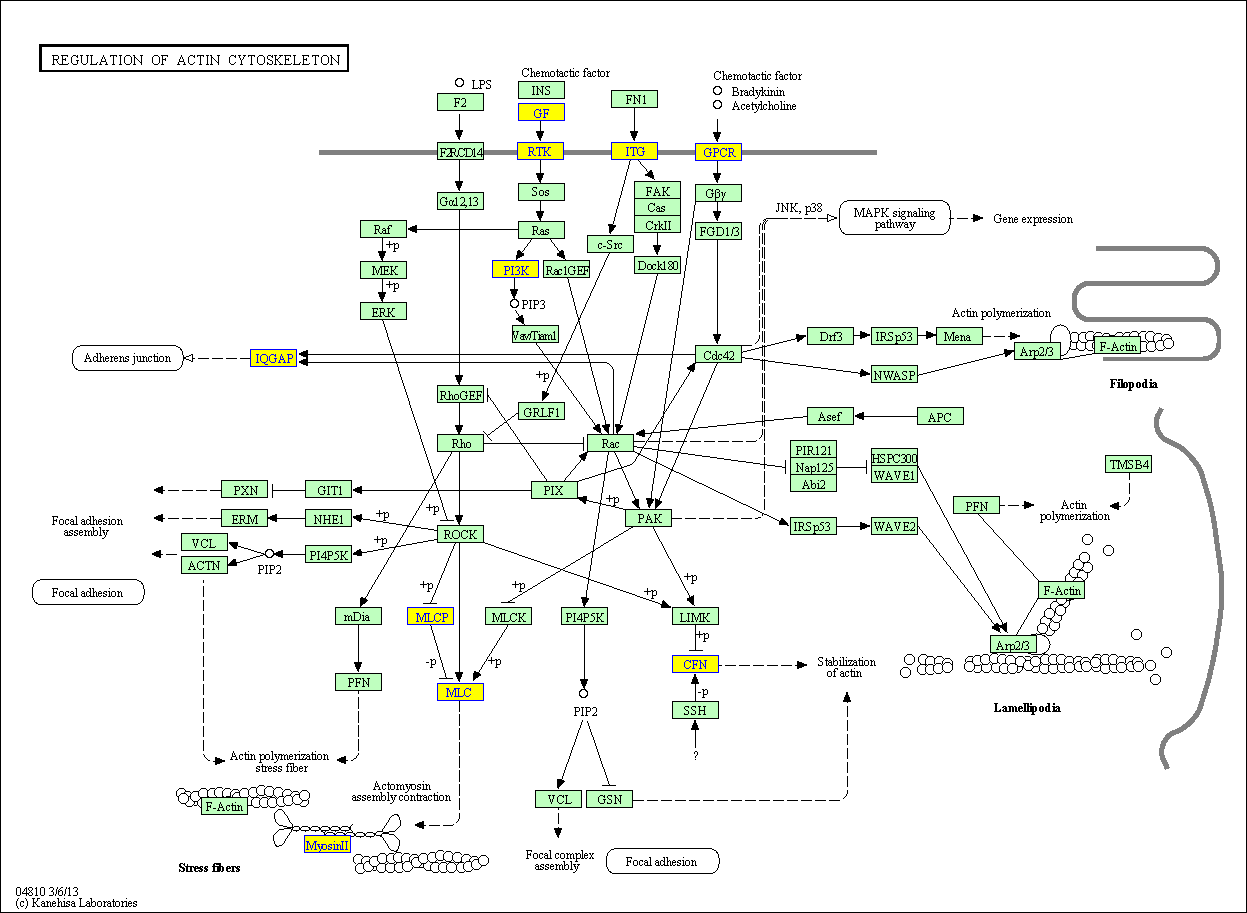

Supplement: Supplementary file 3 [file DataSheet_1.zip › RNA seq raw data/HuGene 2.0 ST Data/Pathway Analysis/A vs B_down/hsa04810.png]

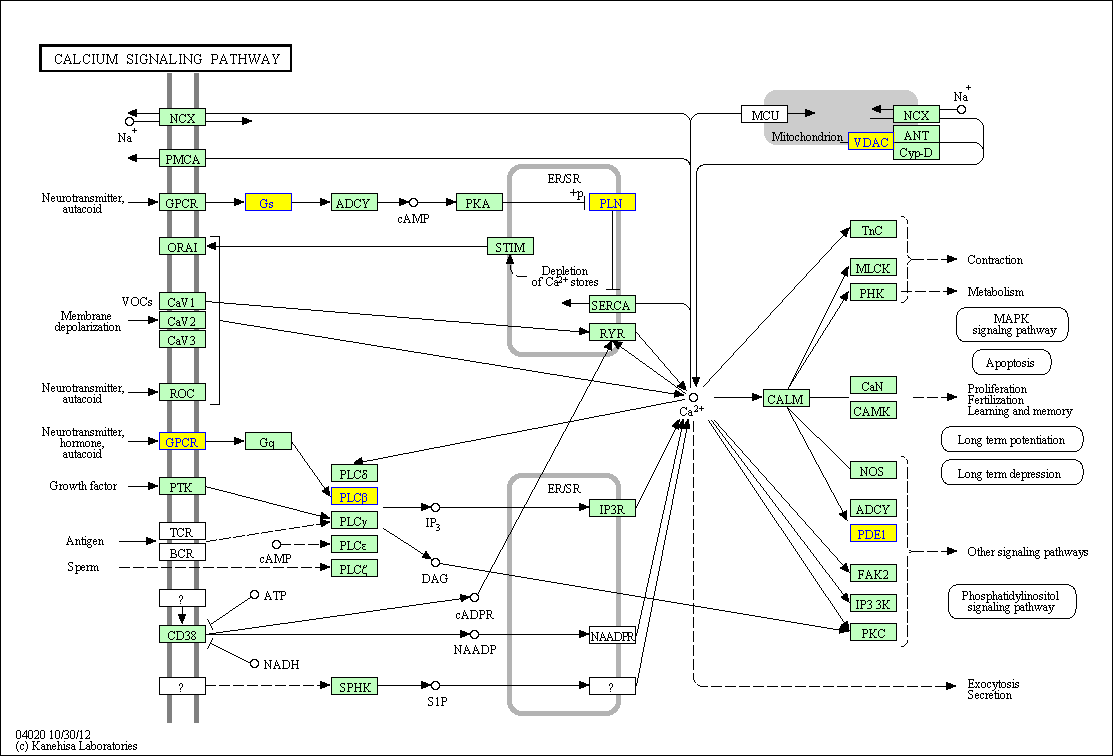

Supplement: Supplementary file 3 [file DataSheet_1.zip › RNA seq raw data/HuGene 2.0 ST Data/Pathway Analysis/A vs B_down/hsa04020.png]

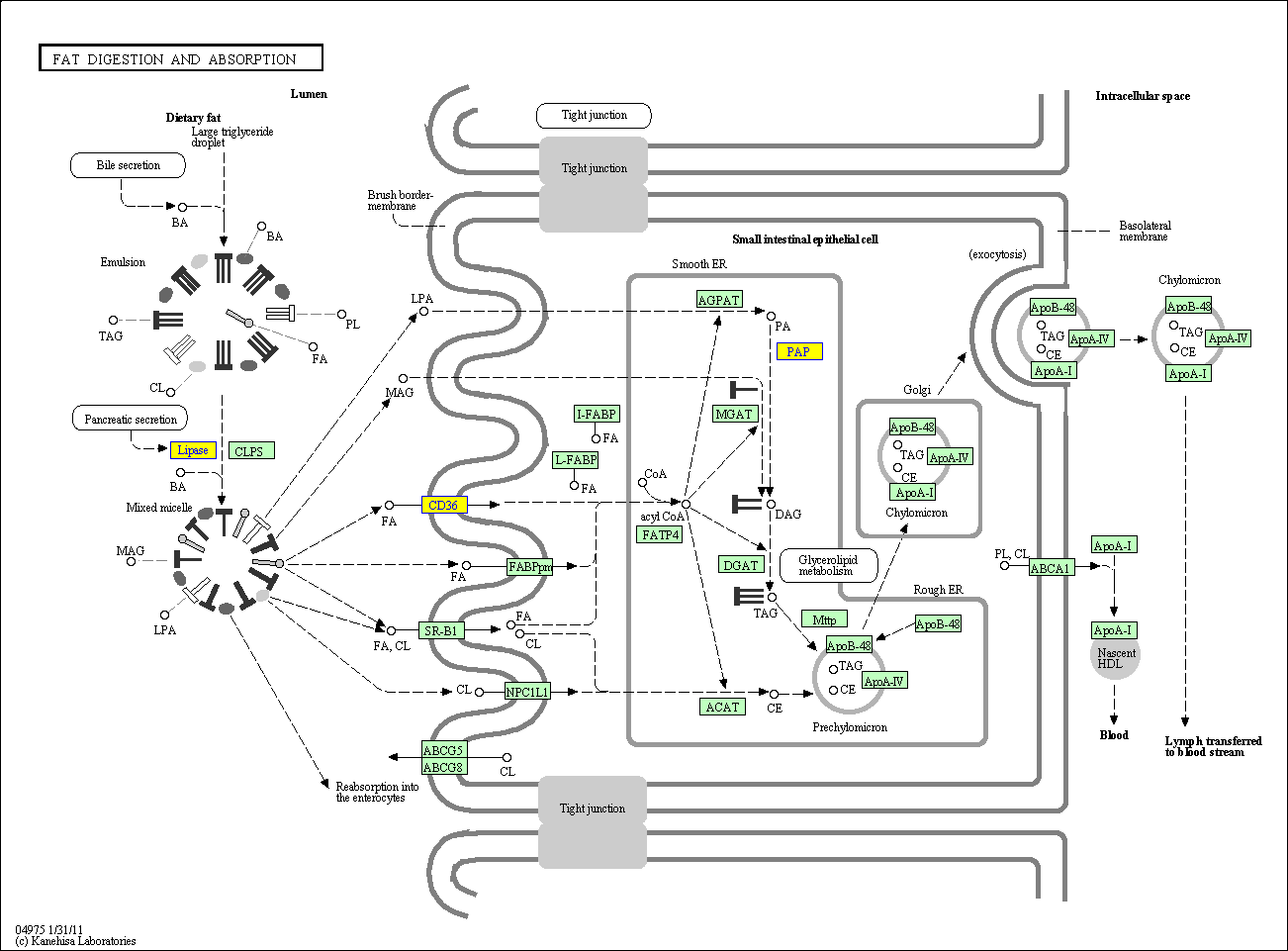

Supplement: Supplementary file 3 [file DataSheet_1.zip › RNA seq raw data/HuGene 2.0 ST Data/Pathway Analysis/A vs B_down/hsa04975.png]

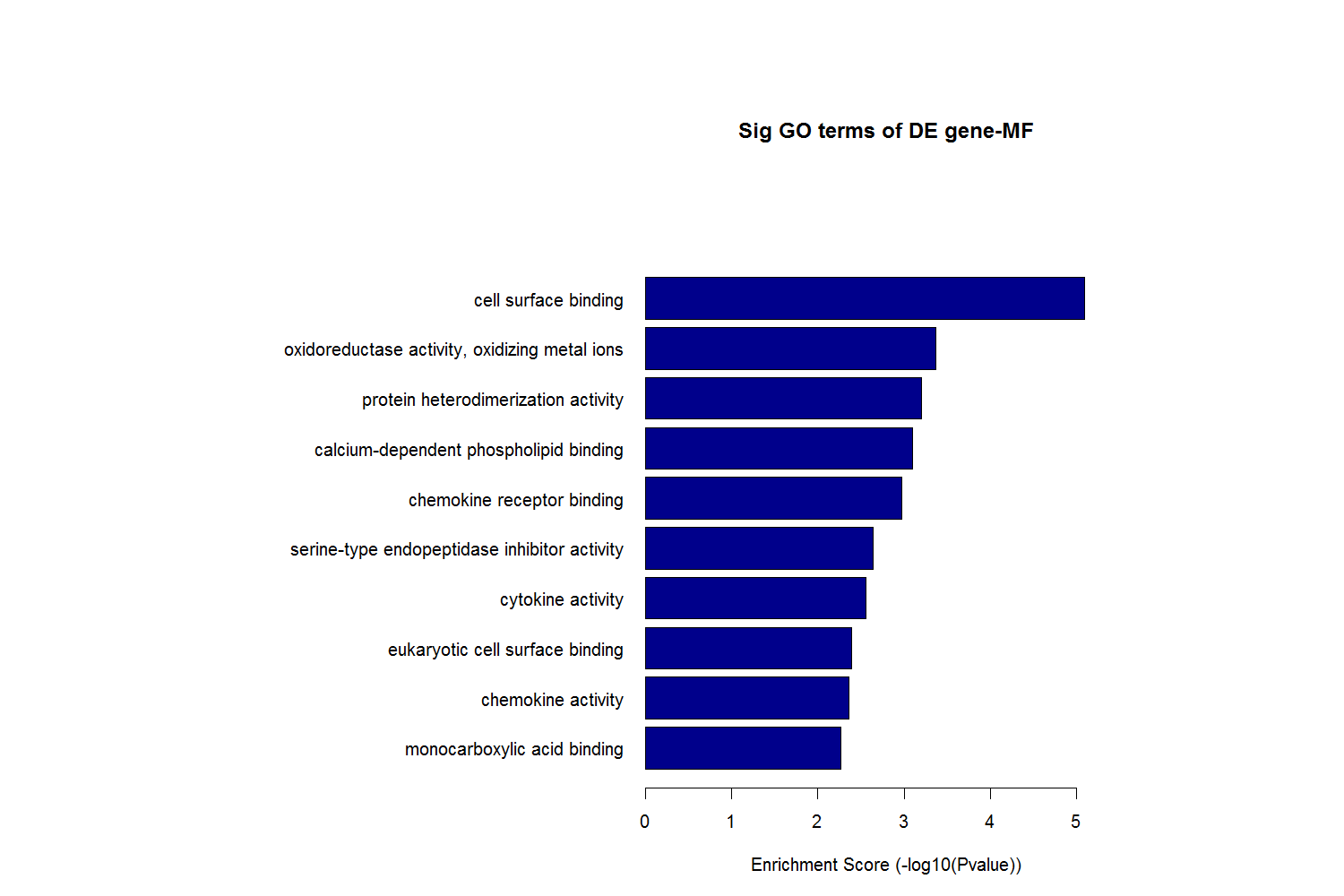

Supplement: Supplementary file 3 [file DataSheet_1.zip › RNA seq raw data/HuGene 2.0 ST Data/GO Analysis/A vs B_up/MF_EnrichmentScore.png]

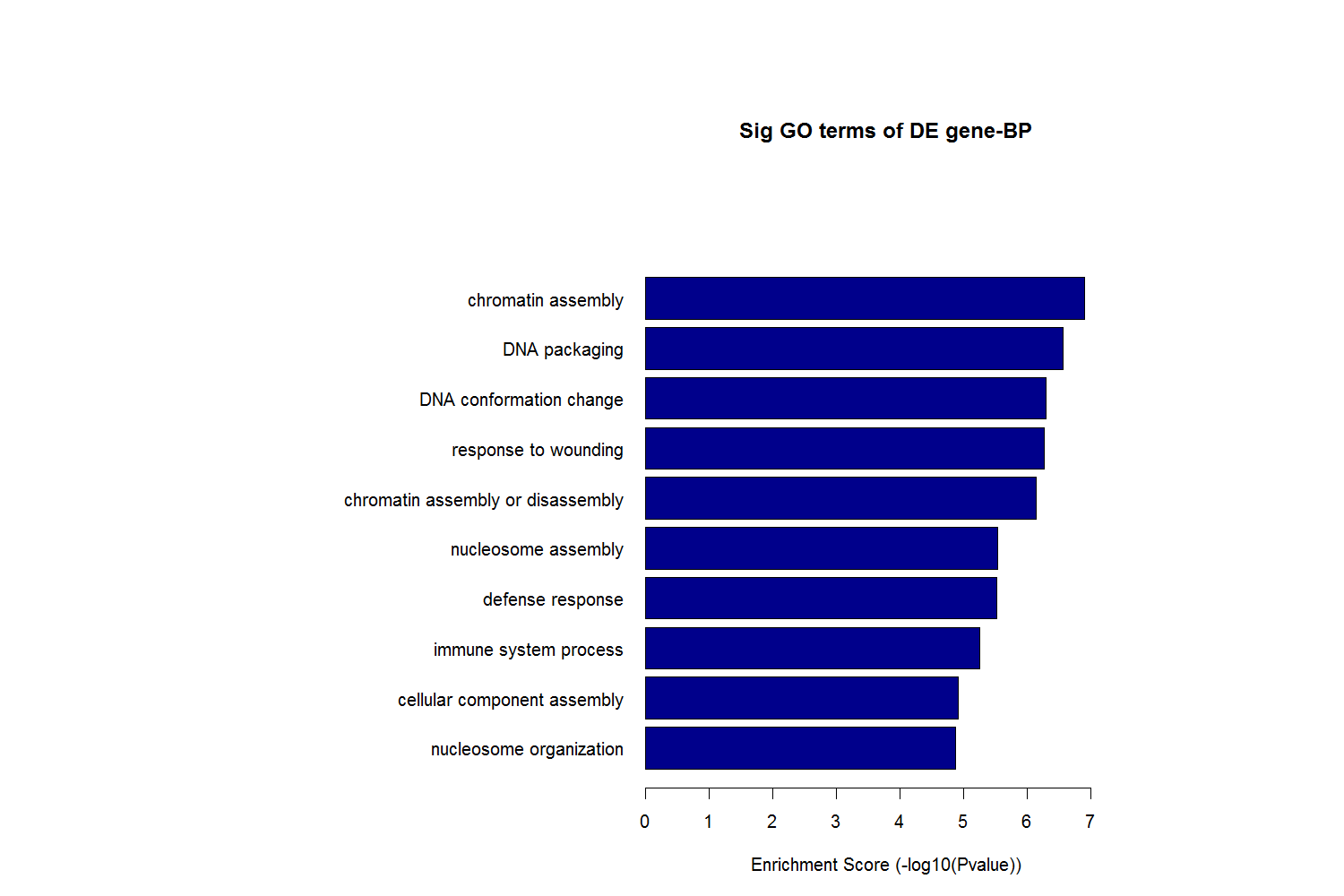

Supplement: Supplementary file 3 [file DataSheet_1.zip › RNA seq raw data/HuGene 2.0 ST Data/GO Analysis/A vs B_up/BP_EnrichmentScore.png]

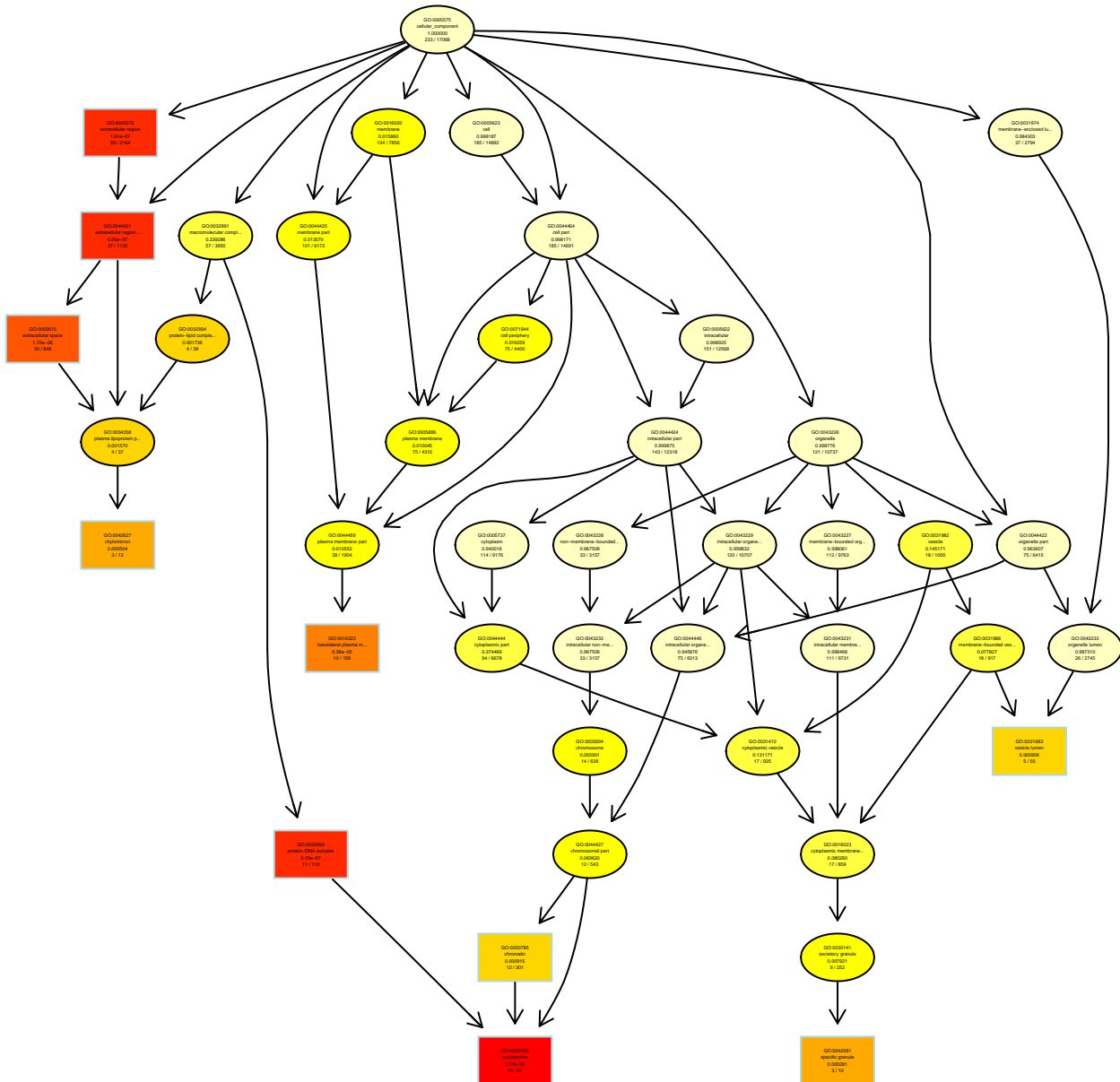

Supplement: Supplementary file 3 [file DataSheet_1.zip › RNA seq raw data/HuGene 2.0 ST Data/GO Analysis/A vs B_up/CC_Pvalue_tree.pdf]

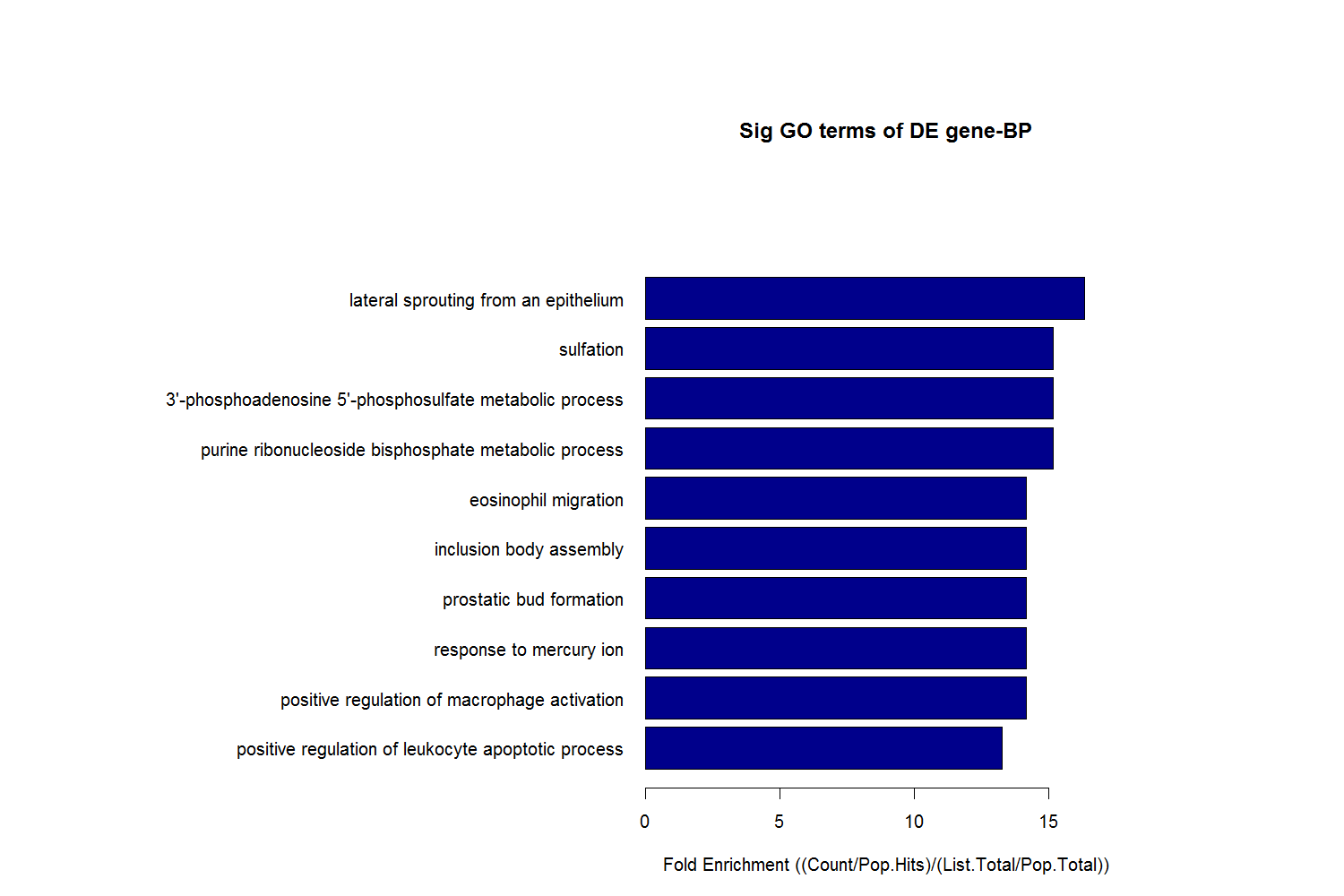

Supplement: Supplementary file 3 [file DataSheet_1.zip › RNA seq raw data/HuGene 2.0 ST Data/GO Analysis/A vs B_up/BP_FoldEnrichment.png]

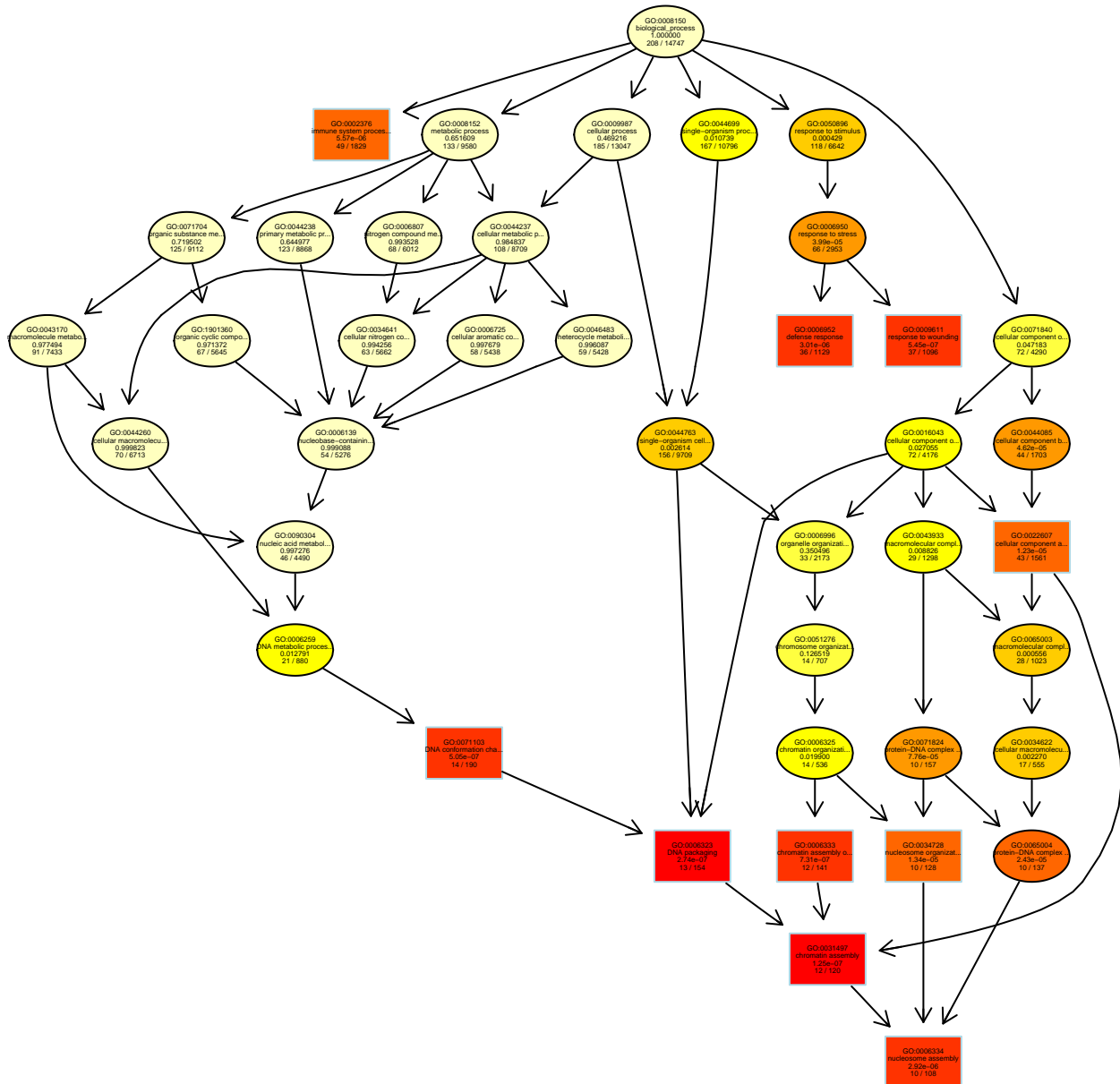

Supplement: Supplementary file 3 [file DataSheet_1.zip › RNA seq raw data/HuGene 2.0 ST Data/GO Analysis/A vs B_up/BP_Pvalue_tree.pdf]

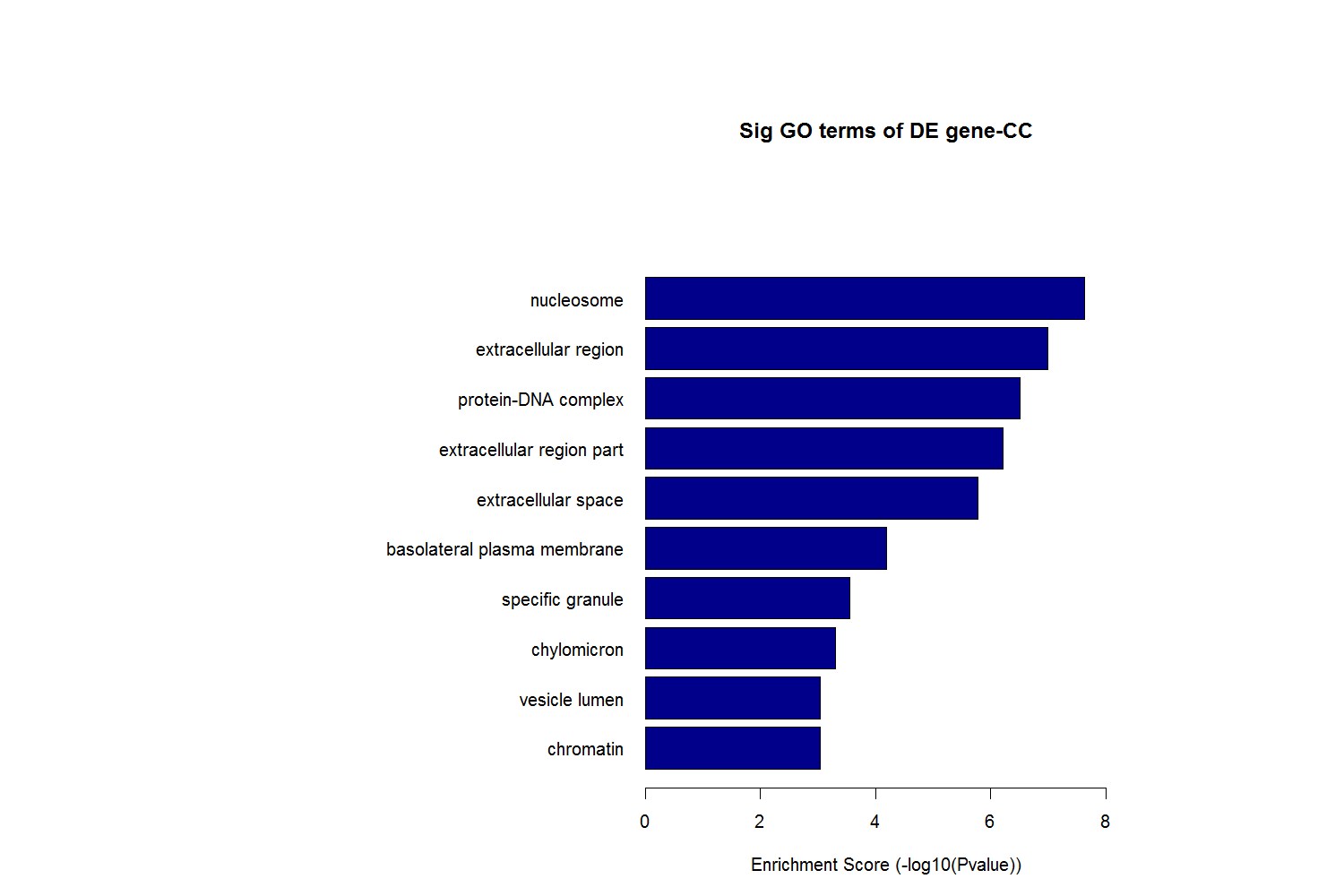

Supplement: Supplementary file 3 [file DataSheet_1.zip › RNA seq raw data/HuGene 2.0 ST Data/GO Analysis/A vs B_up/CC_EnrichmentScore.png]

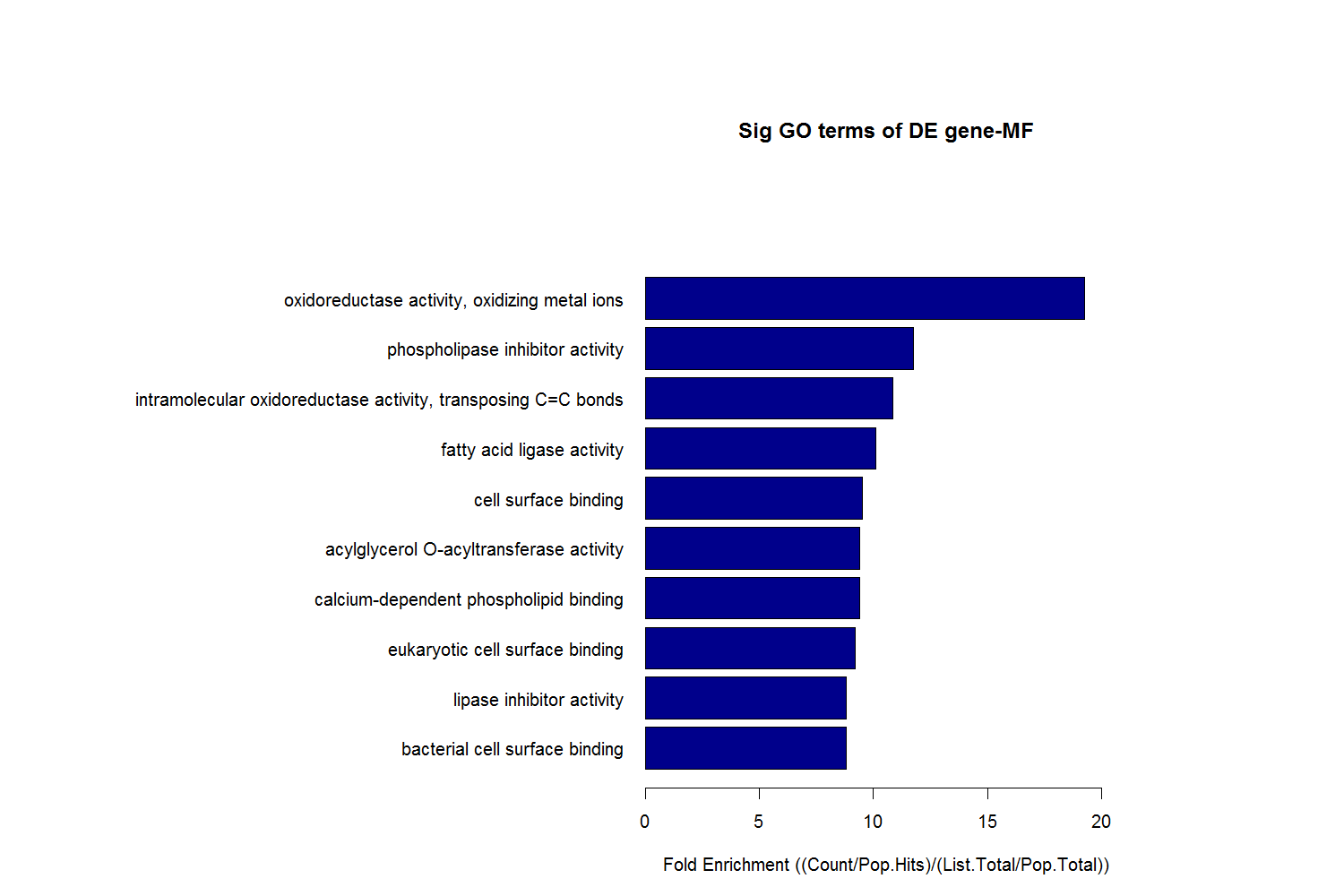

Supplement: Supplementary file 3 [file DataSheet_1.zip › RNA seq raw data/HuGene 2.0 ST Data/GO Analysis/A vs B_up/MF_FoldEnrichment.png]

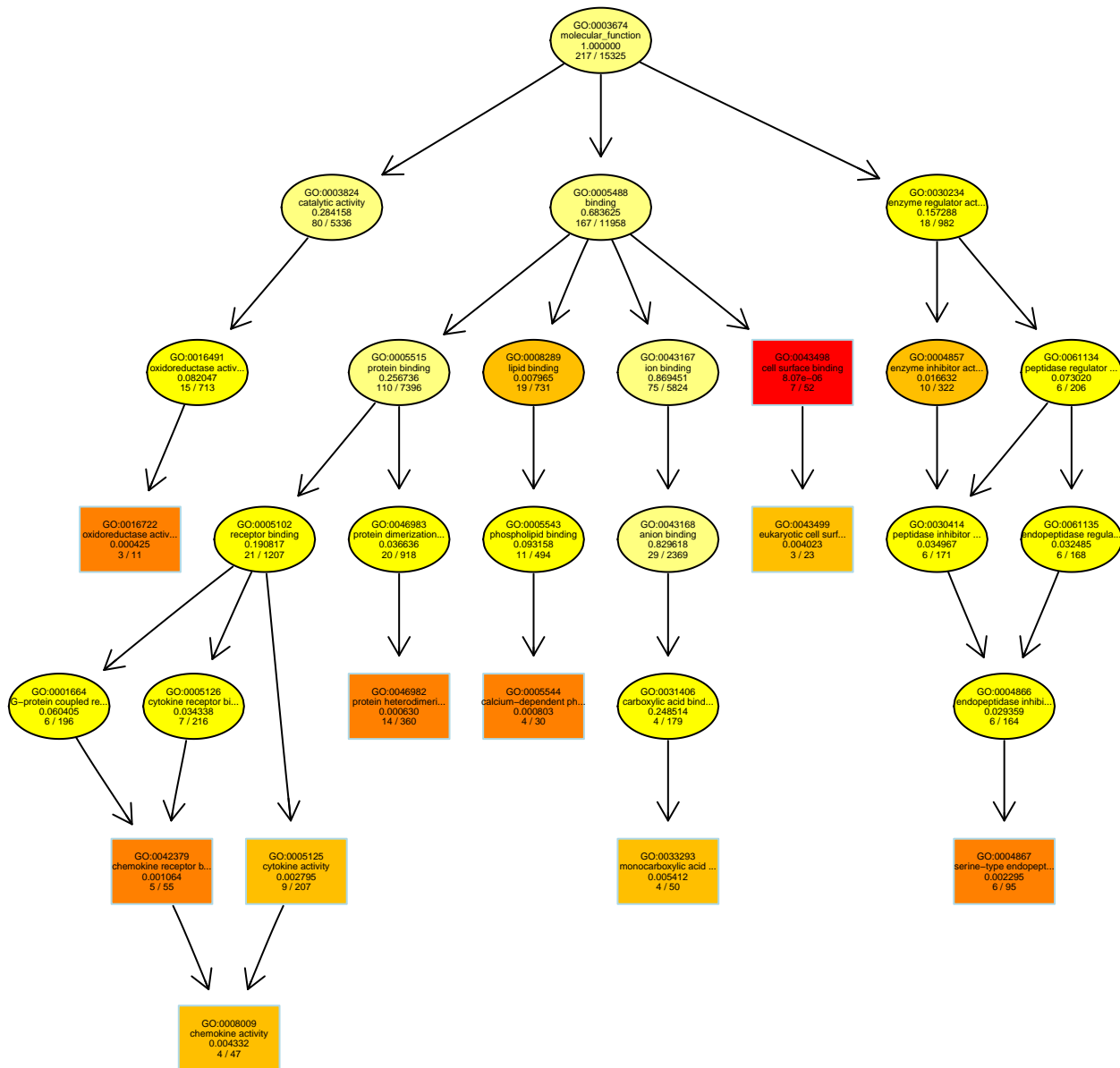

Supplement: Supplementary file 3 [file DataSheet_1.zip › RNA seq raw data/HuGene 2.0 ST Data/GO Analysis/A vs B_up/MF_Pvalue_tree.pdf]

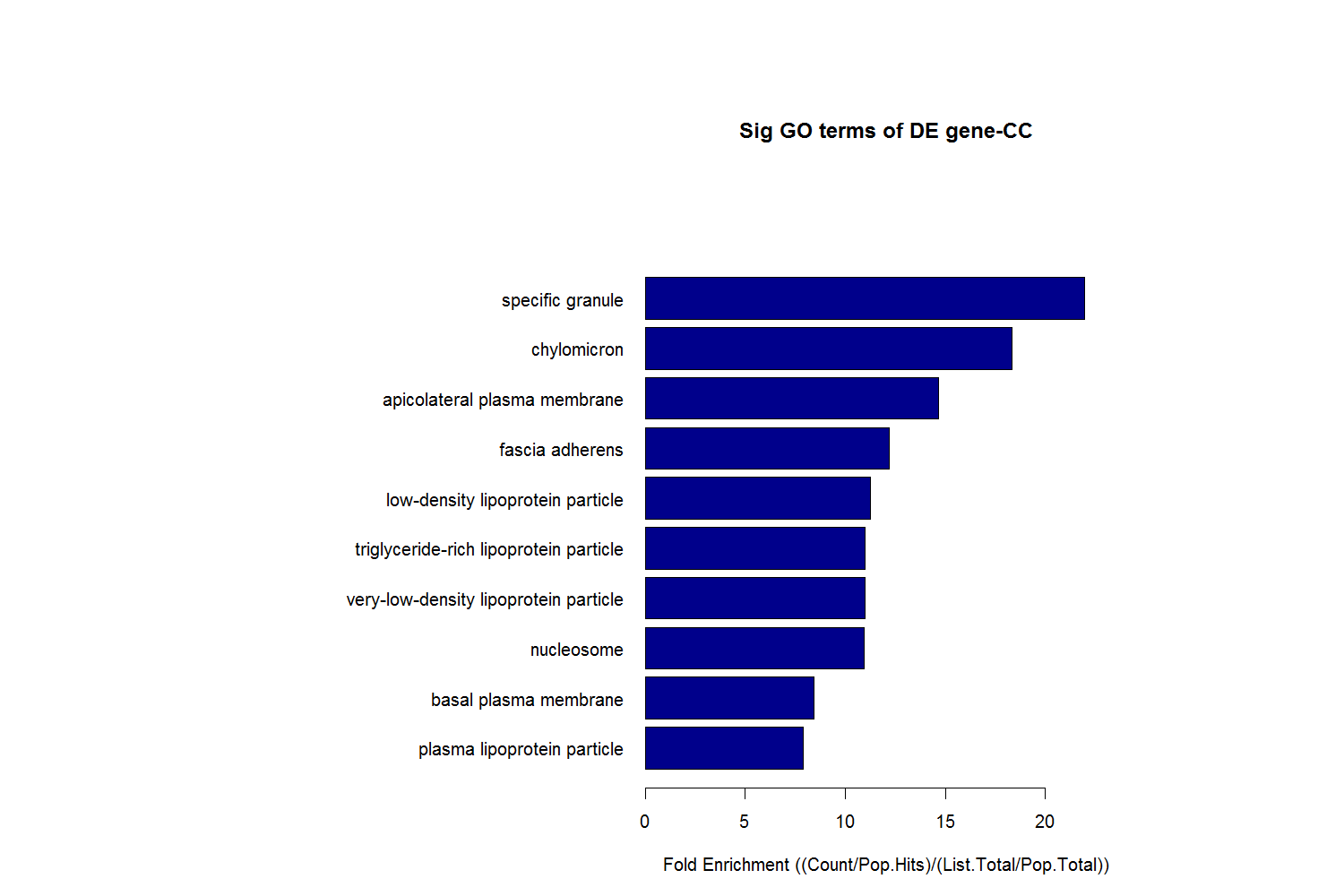

Supplement: Supplementary file 3 [file DataSheet_1.zip › RNA seq raw data/HuGene 2.0 ST Data/GO Analysis/A vs B_up/CC_FoldEnrichment.png]

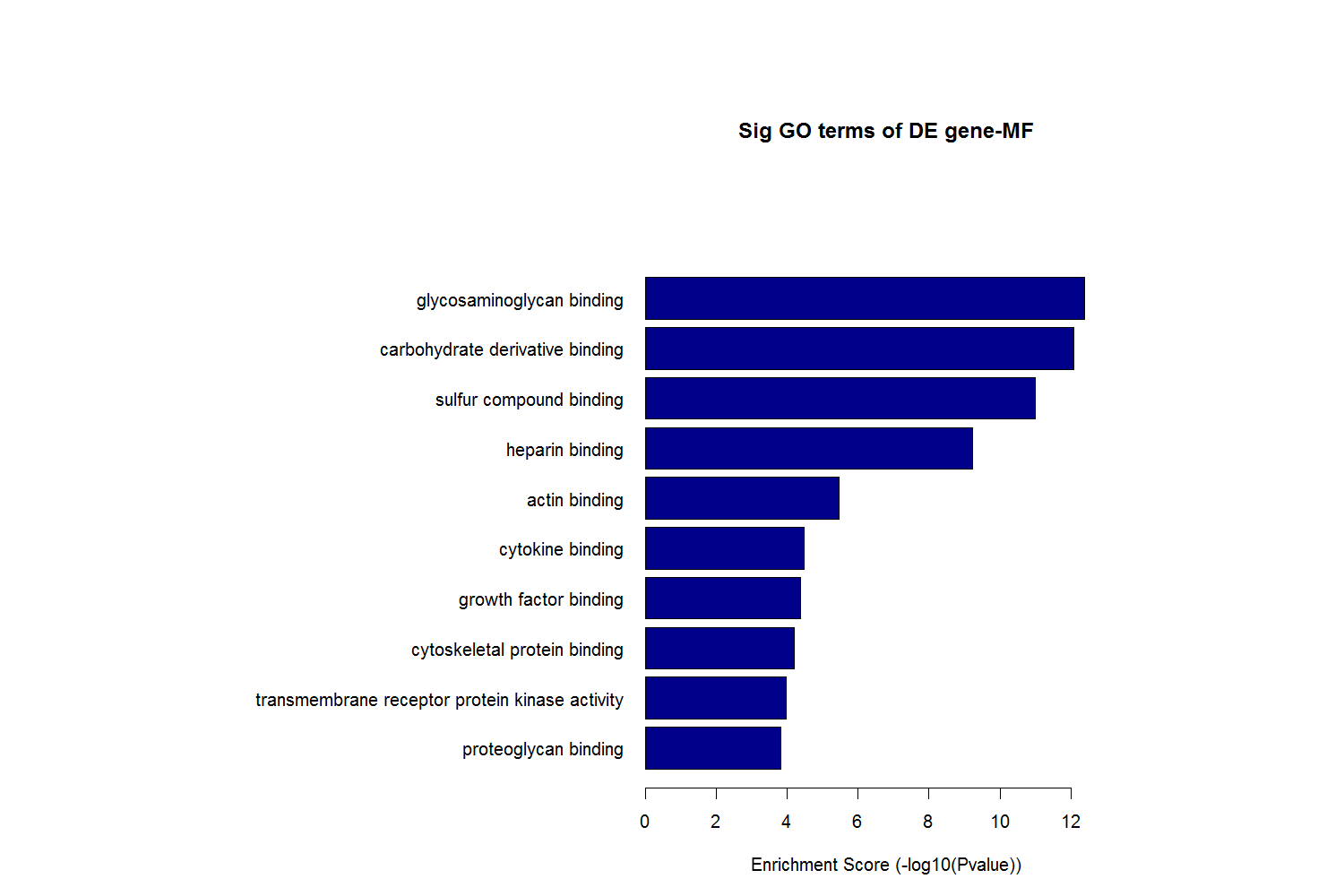

Supplement: Supplementary file 3 [file DataSheet_1.zip › RNA seq raw data/HuGene 2.0 ST Data/GO Analysis/A vs B_down/MF_EnrichmentScore.png]

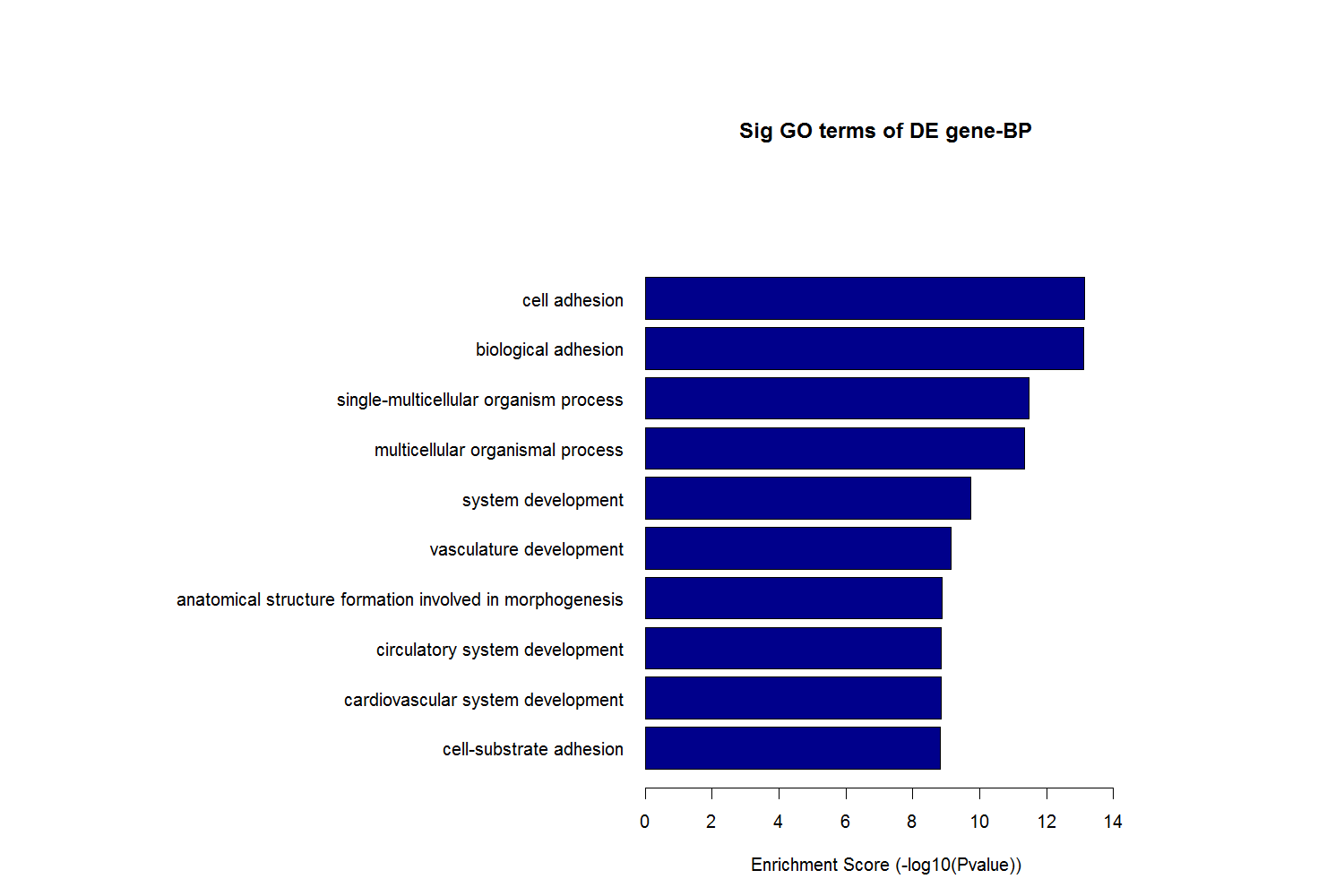

Supplement: Supplementary file 3 [file DataSheet_1.zip › RNA seq raw data/HuGene 2.0 ST Data/GO Analysis/A vs B_down/BP_EnrichmentScore.png]

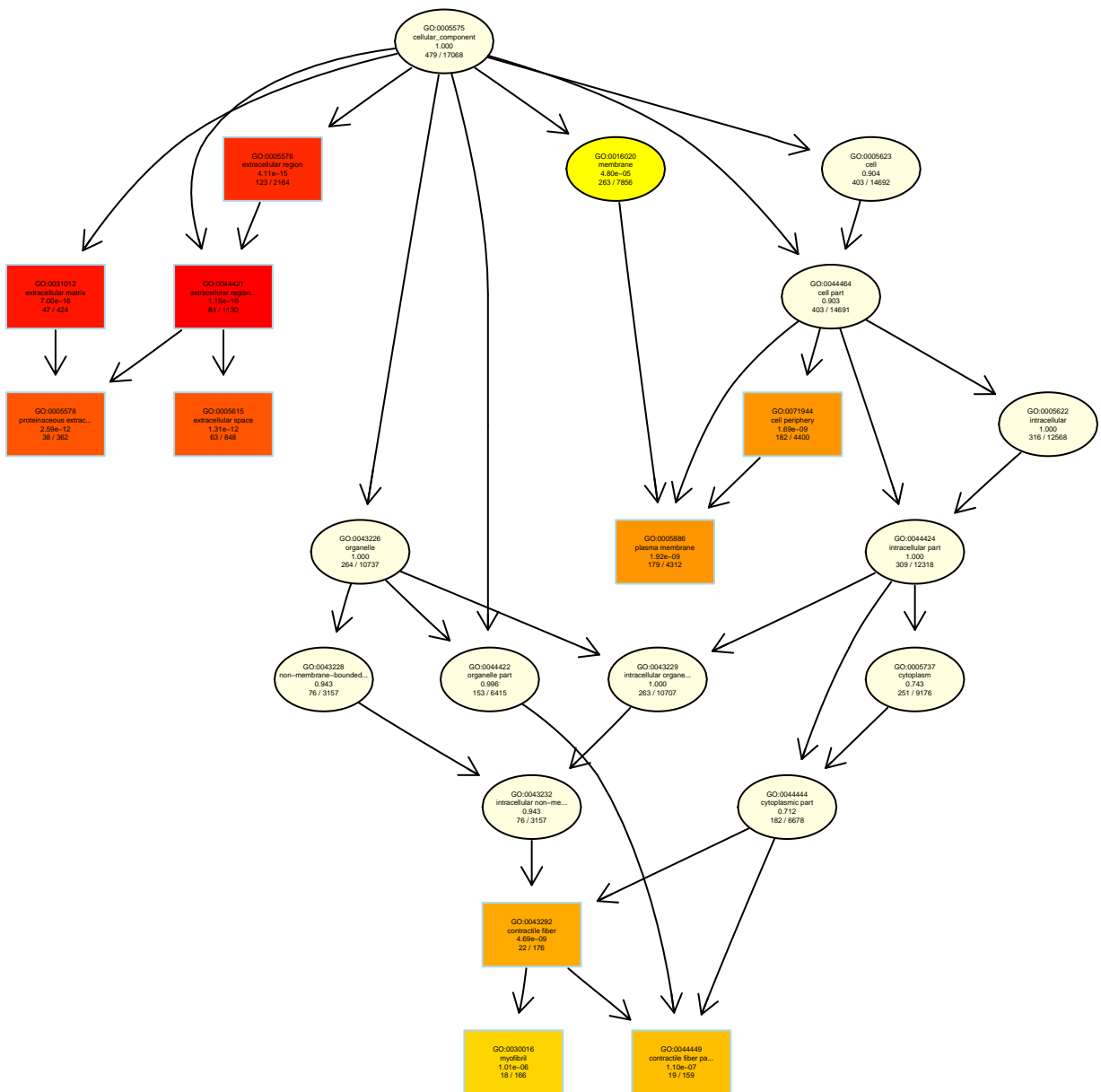

Supplement: Supplementary file 3 [file DataSheet_1.zip › RNA seq raw data/HuGene 2.0 ST Data/GO Analysis/A vs B_down/CC_Pvalue_tree.pdf]

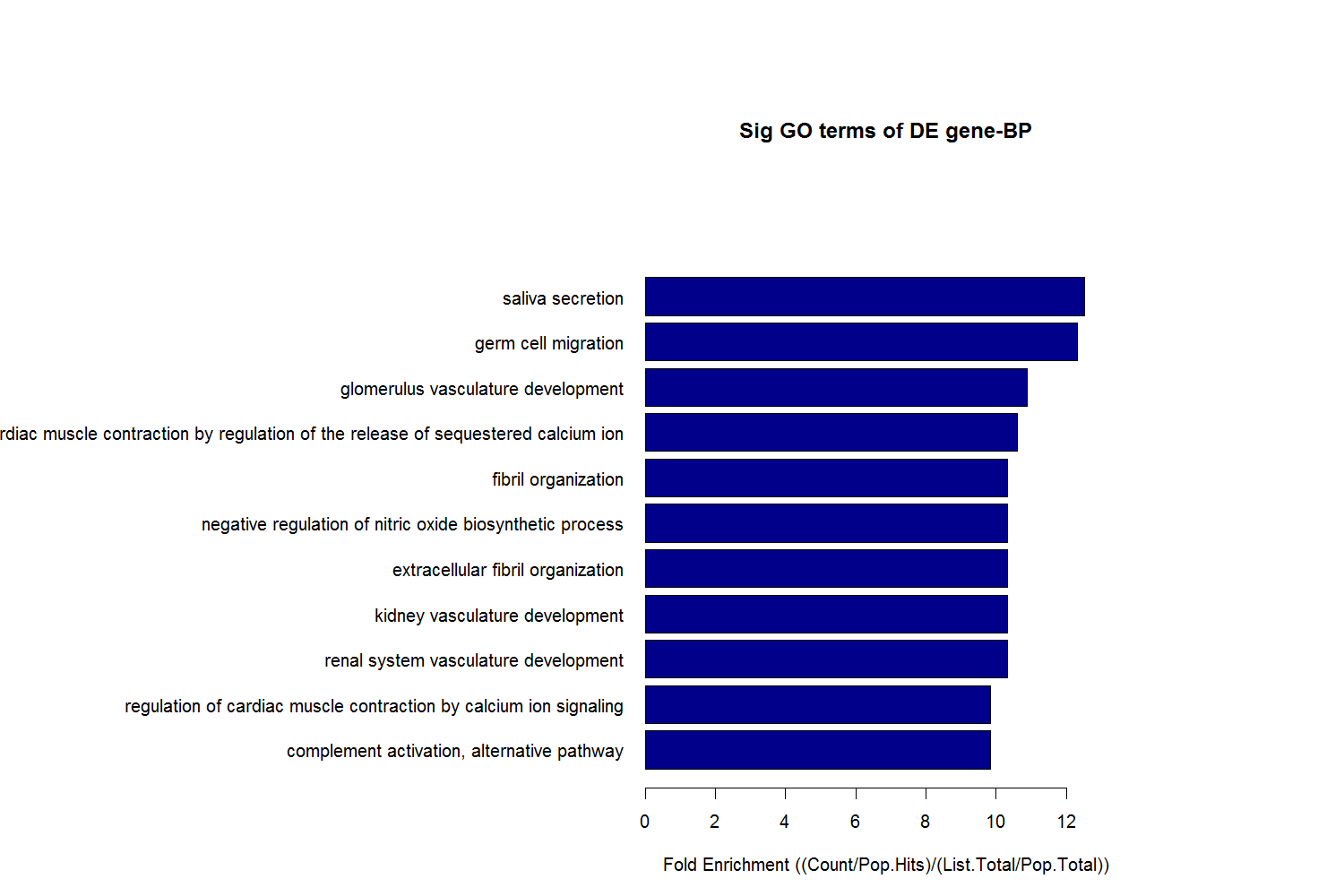

Supplement: Supplementary file 3 [file DataSheet_1.zip › RNA seq raw data/HuGene 2.0 ST Data/GO Analysis/A vs B_down/BP_FoldEnrichment.png]

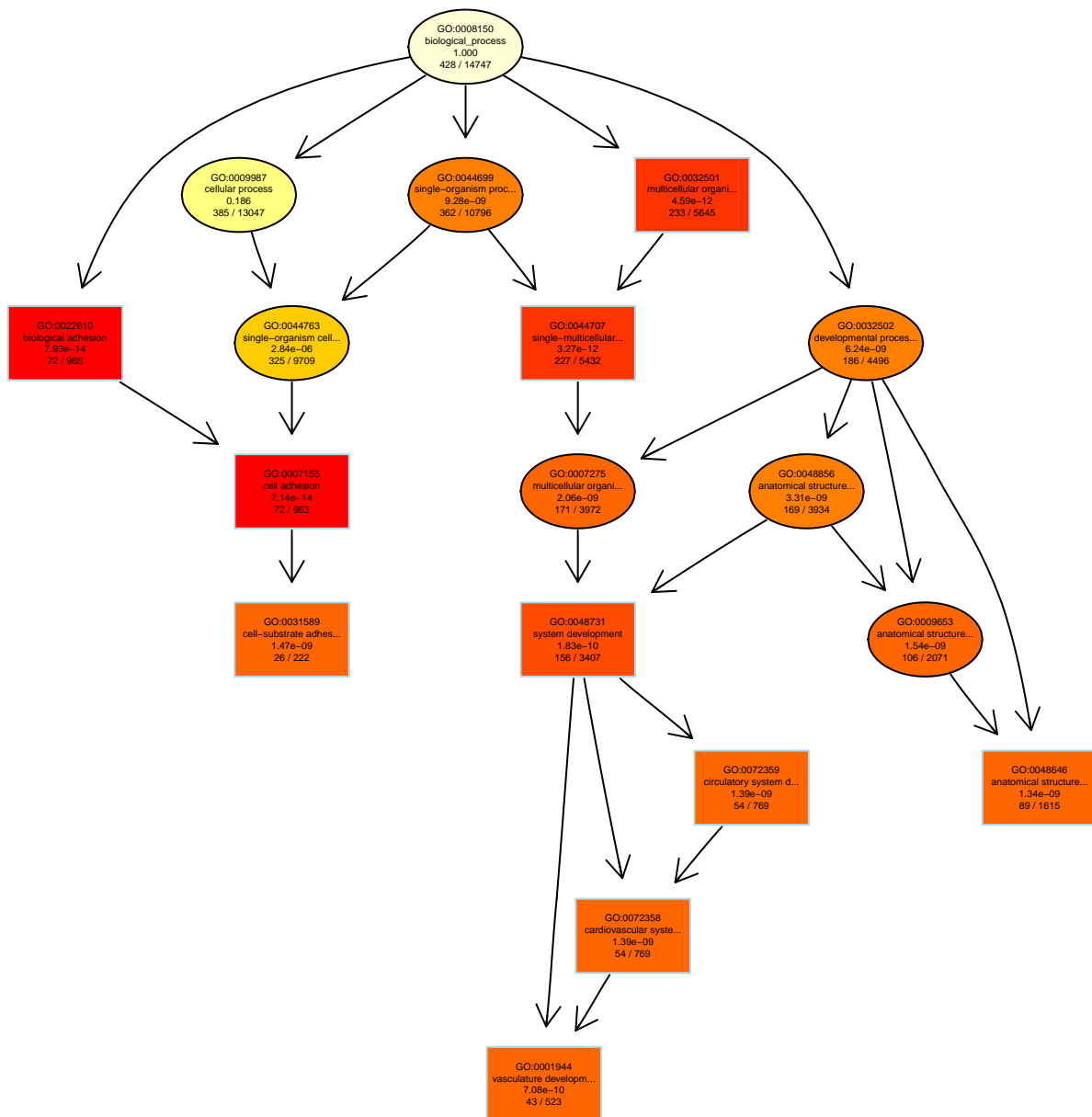

Supplement: Supplementary file 3 [file DataSheet_1.zip › RNA seq raw data/HuGene 2.0 ST Data/GO Analysis/A vs B_down/BP_Pvalue_tree.pdf]

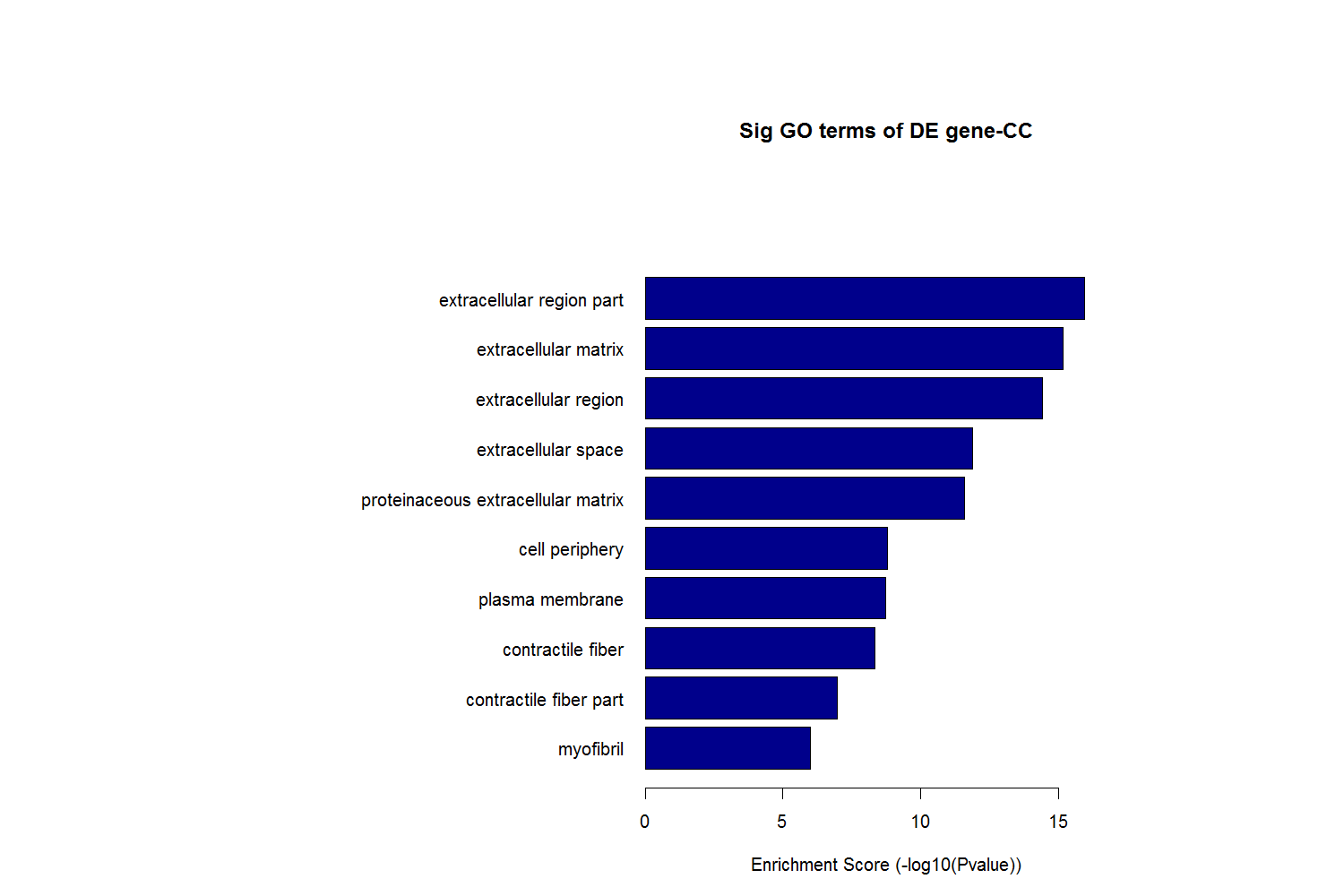

Supplement: Supplementary file 3 [file DataSheet_1.zip › RNA seq raw data/HuGene 2.0 ST Data/GO Analysis/A vs B_down/CC_EnrichmentScore.png]

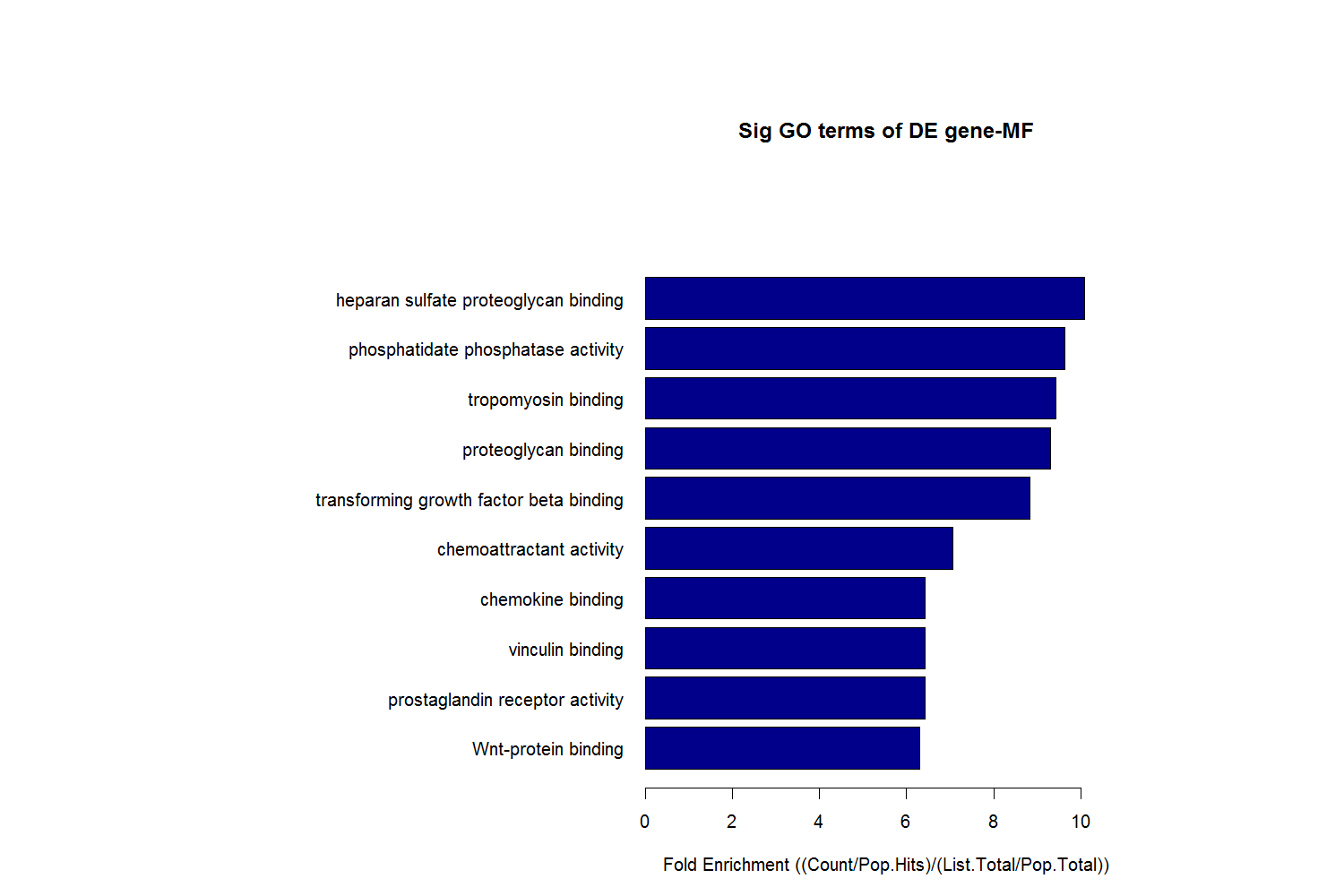

Supplement: Supplementary file 3 [file DataSheet_1.zip › RNA seq raw data/HuGene 2.0 ST Data/GO Analysis/A vs B_down/MF_FoldEnrichment.png]

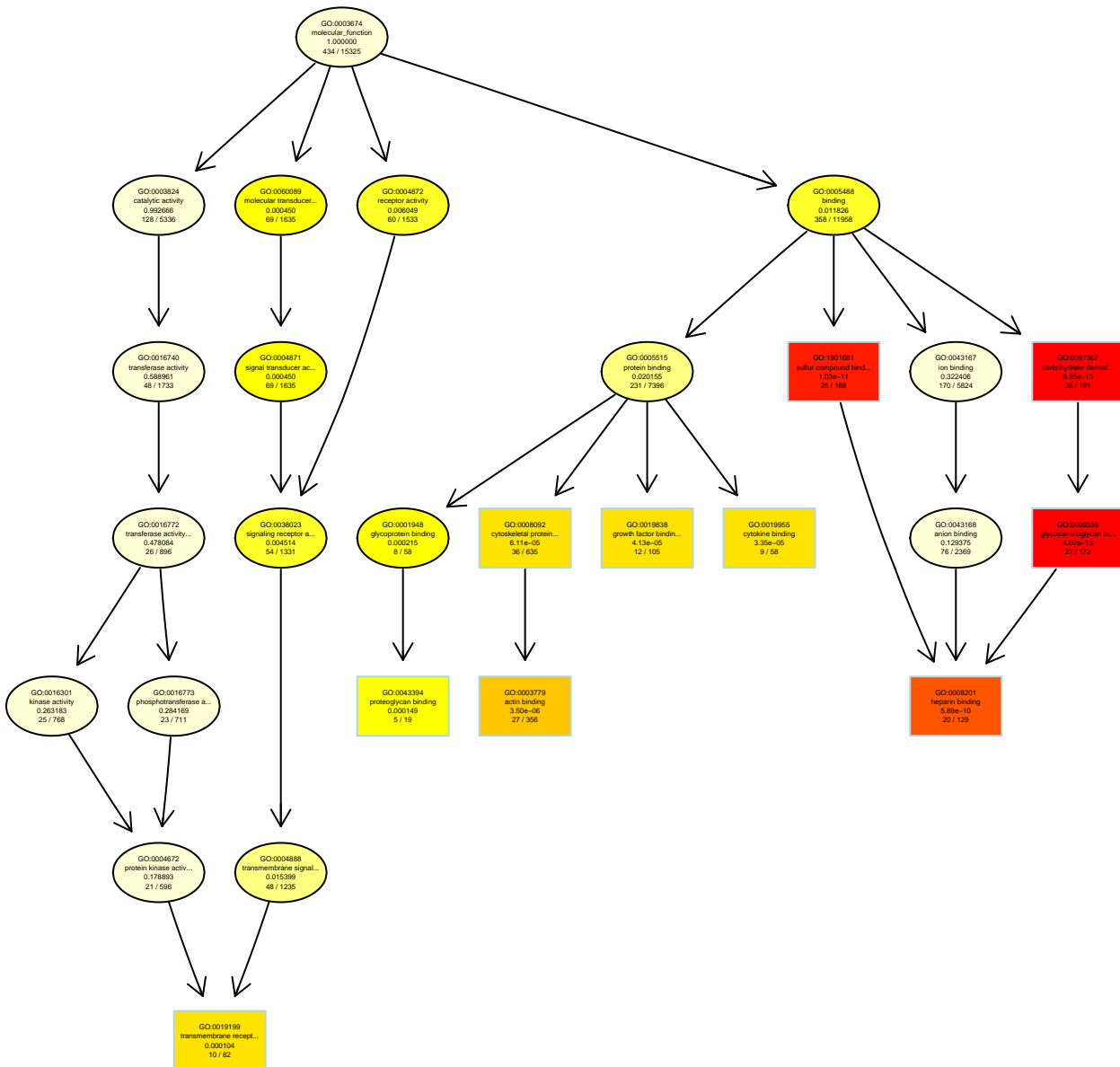

Supplement: Supplementary file 3 [file DataSheet_1.zip › RNA seq raw data/HuGene 2.0 ST Data/GO Analysis/A vs B_down/MF_Pvalue_tree.pdf]

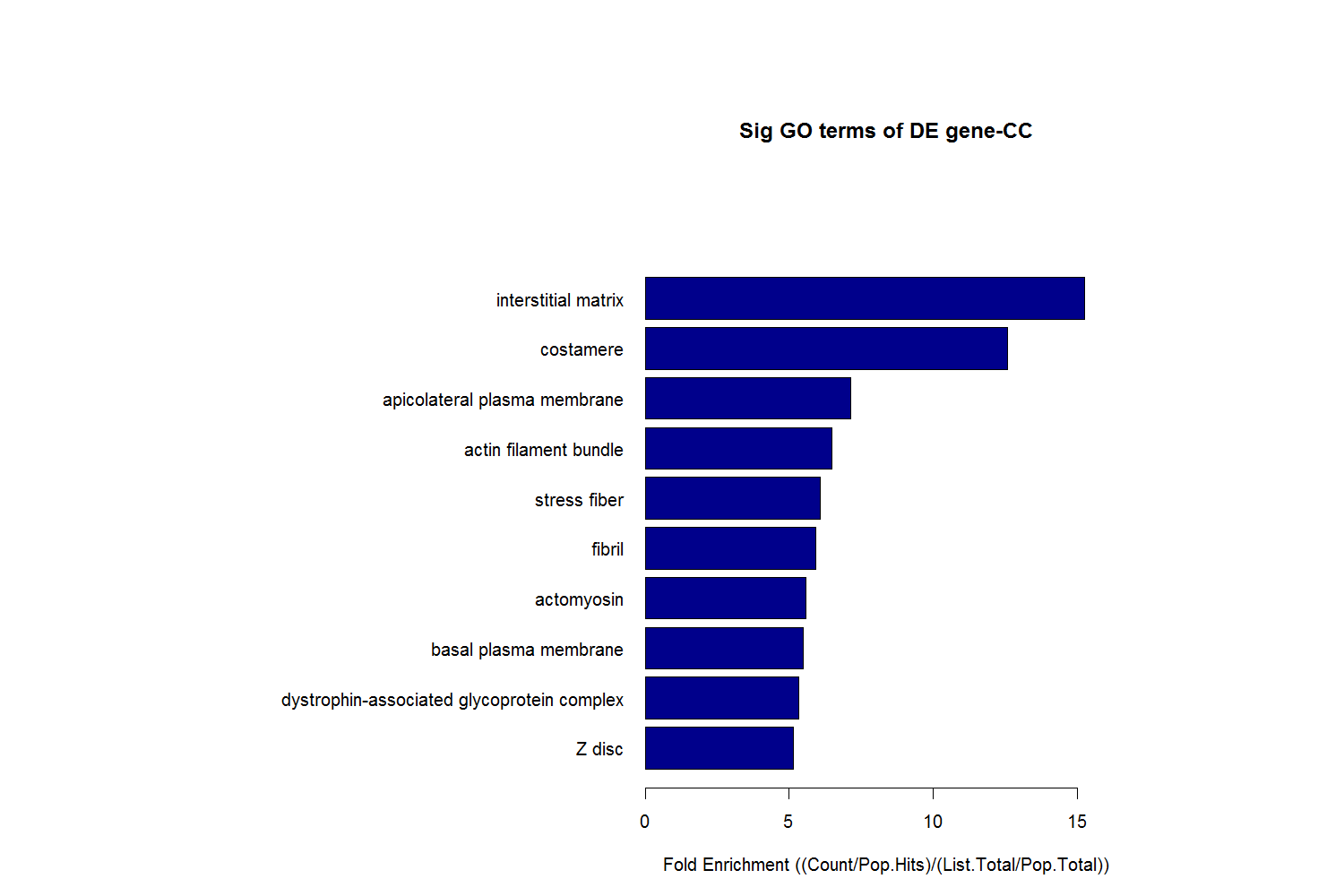

Supplement: Supplementary file 3 [file DataSheet_1.zip › RNA seq raw data/HuGene 2.0 ST Data/GO Analysis/A vs B_down/CC_FoldEnrichment.png]

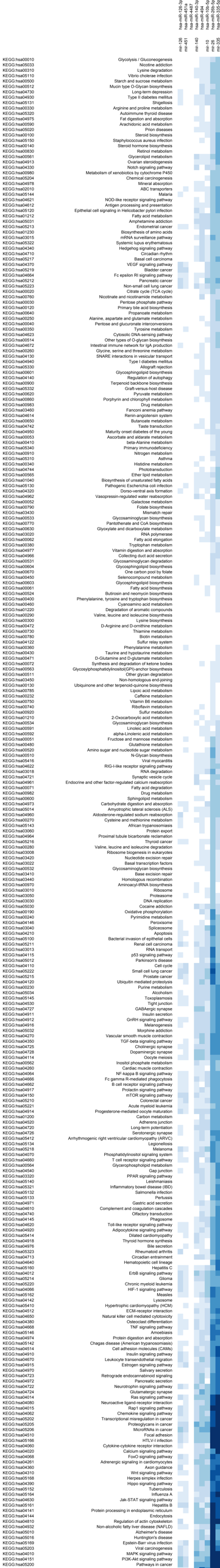

Number of Target Genes

Supplement: Supplementary file 3 [file DataSheet_1.zip › RNA seq raw data/mirPath Analysis/A vs B_dn miRNA up Gene/Heatmap/Biclustering.pdf]

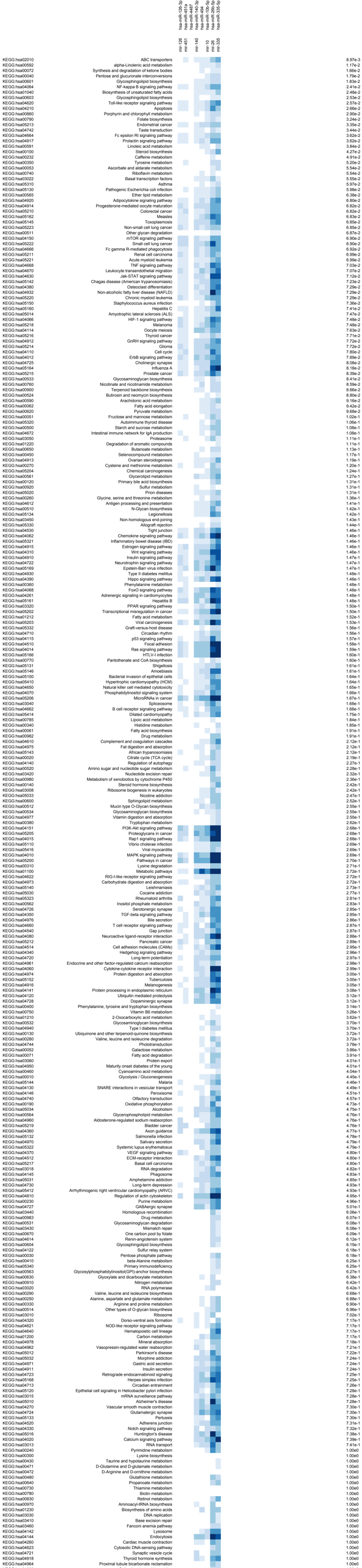

Supplement: Supplementary file 3 [file DataSheet_1.zip › RNA seq raw data/mirPath Analysis/A vs B_dn miRNA up Gene/Heatmap/OrderedByFisherExact.pdf]

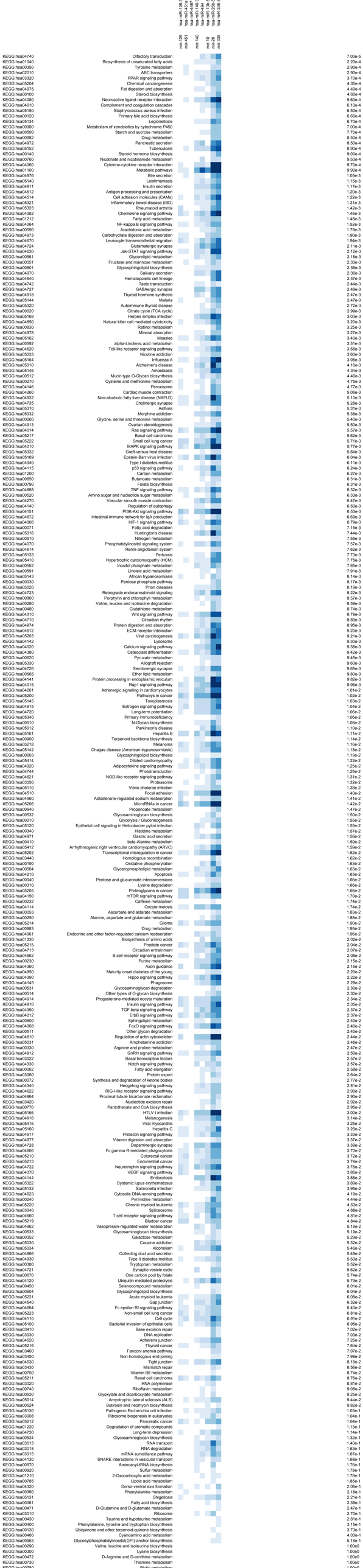

Supplement: Supplementary file 3 [file DataSheet_1.zip › RNA seq raw data/mirPath Analysis/A vs B_dn miRNA up Gene/Heatmap/OrderedByRandomSampling.pdf]

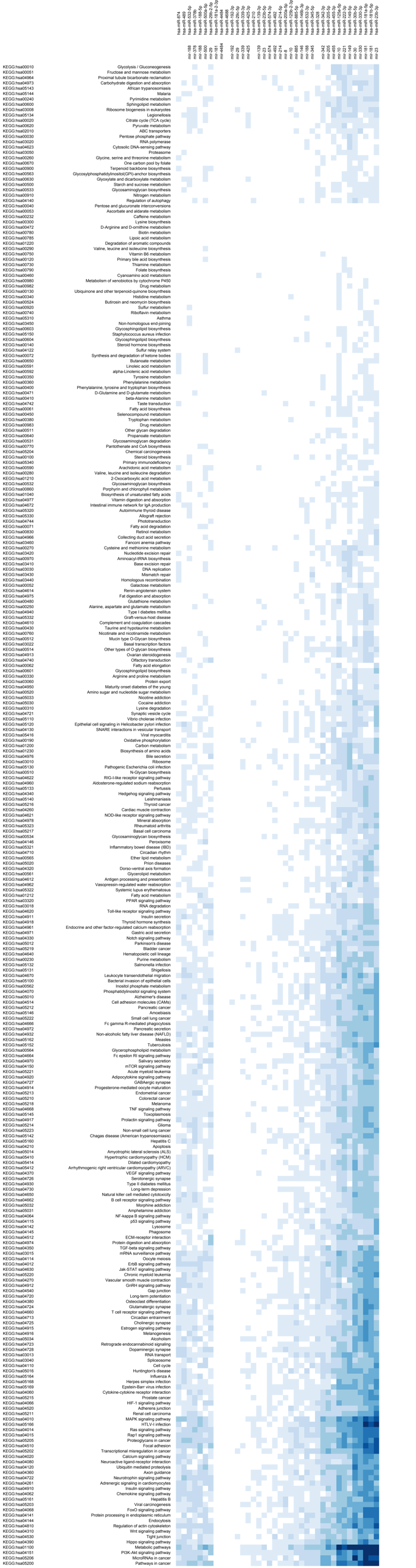

Supplement: Supplementary file 3 [file DataSheet_1.zip › RNA seq raw data/mirPath Analysis/A vs B_up miRNA dn Gene/Heatmap/Biclustering.pdf]

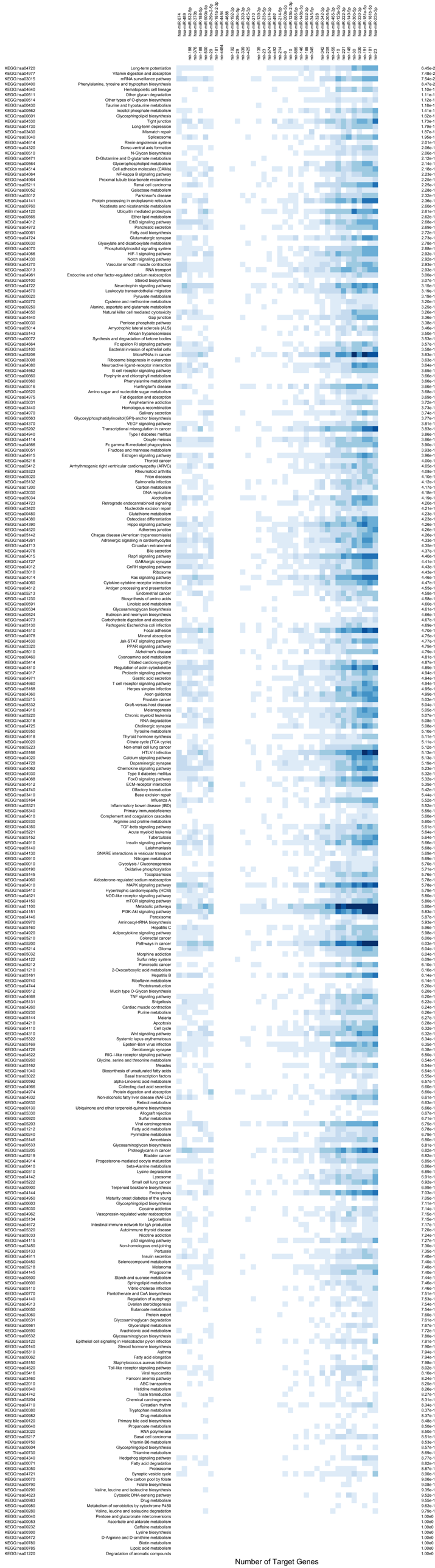

Supplement: Supplementary file 3 [file DataSheet_1.zip › RNA seq raw data/mirPath Analysis/A vs B_up miRNA dn Gene/Heatmap/OrderedByRandomSampling.pdf]

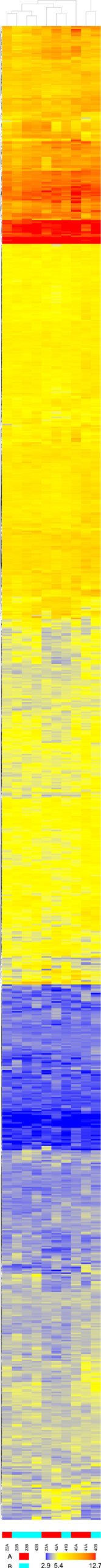

Supplement: Supplementary file 3 [file DataSheet_1.zip › RNA seq raw data/Heatmap/NoRowNormalization/AllsnoRNA.pdf]

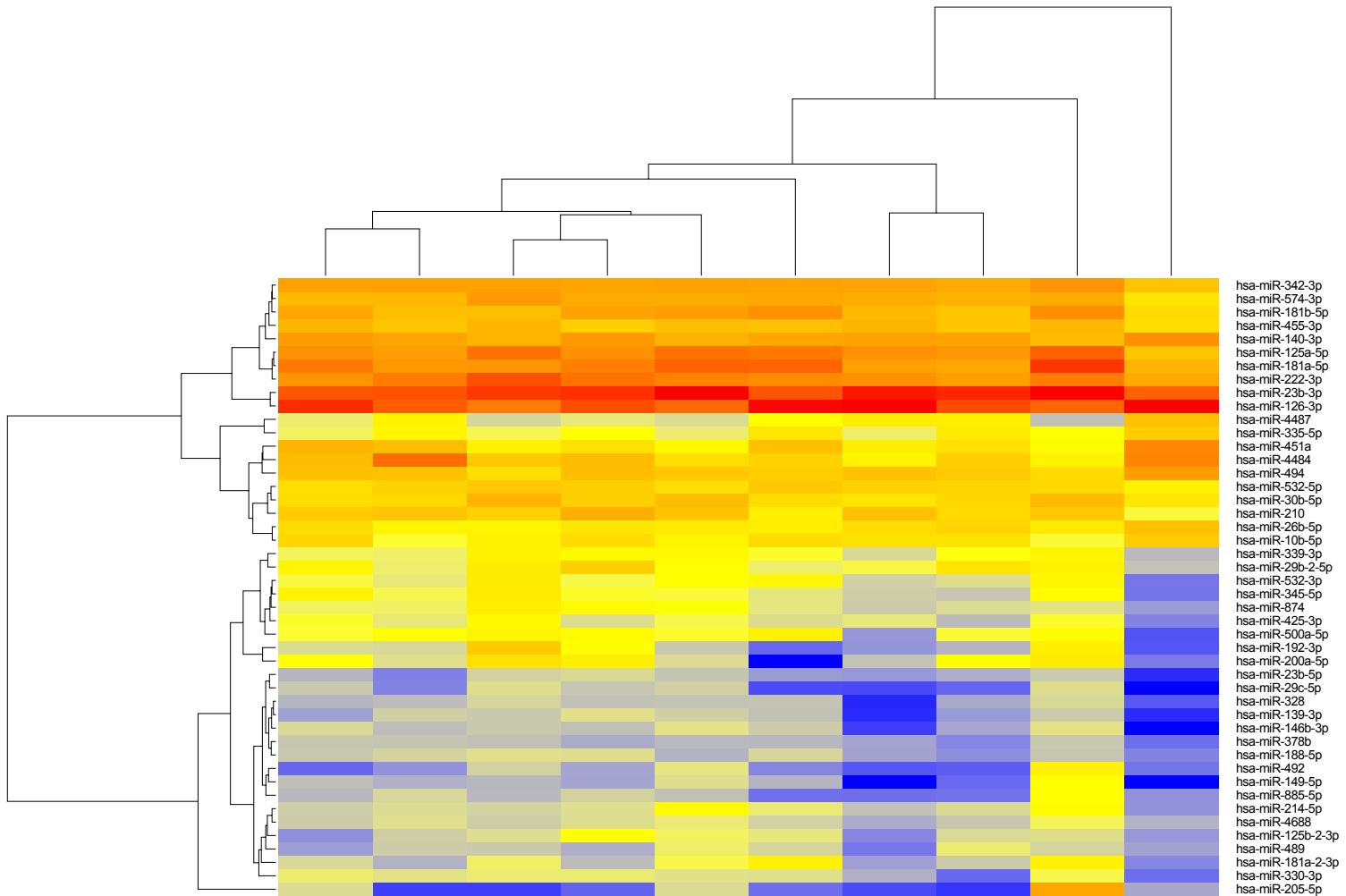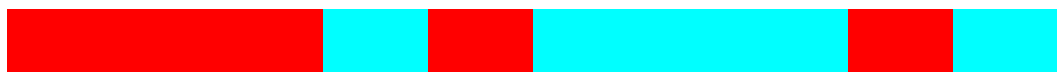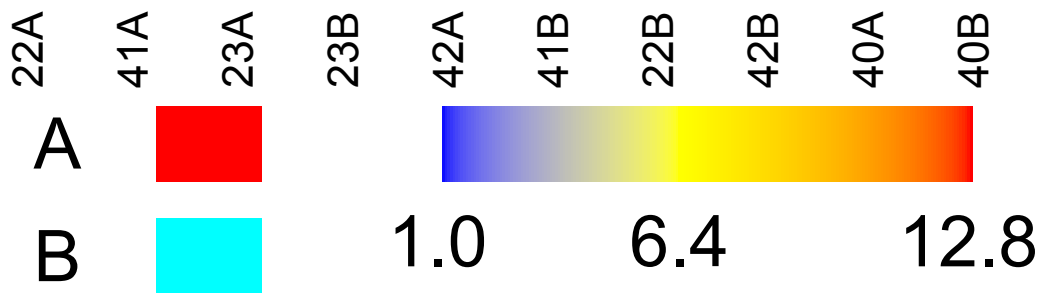

Supplement: Supplementary file 3 [file DataSheet_1.zip › RNA seq raw data/Heatmap/NoRowNormalization/diffMiRNA.pdf]

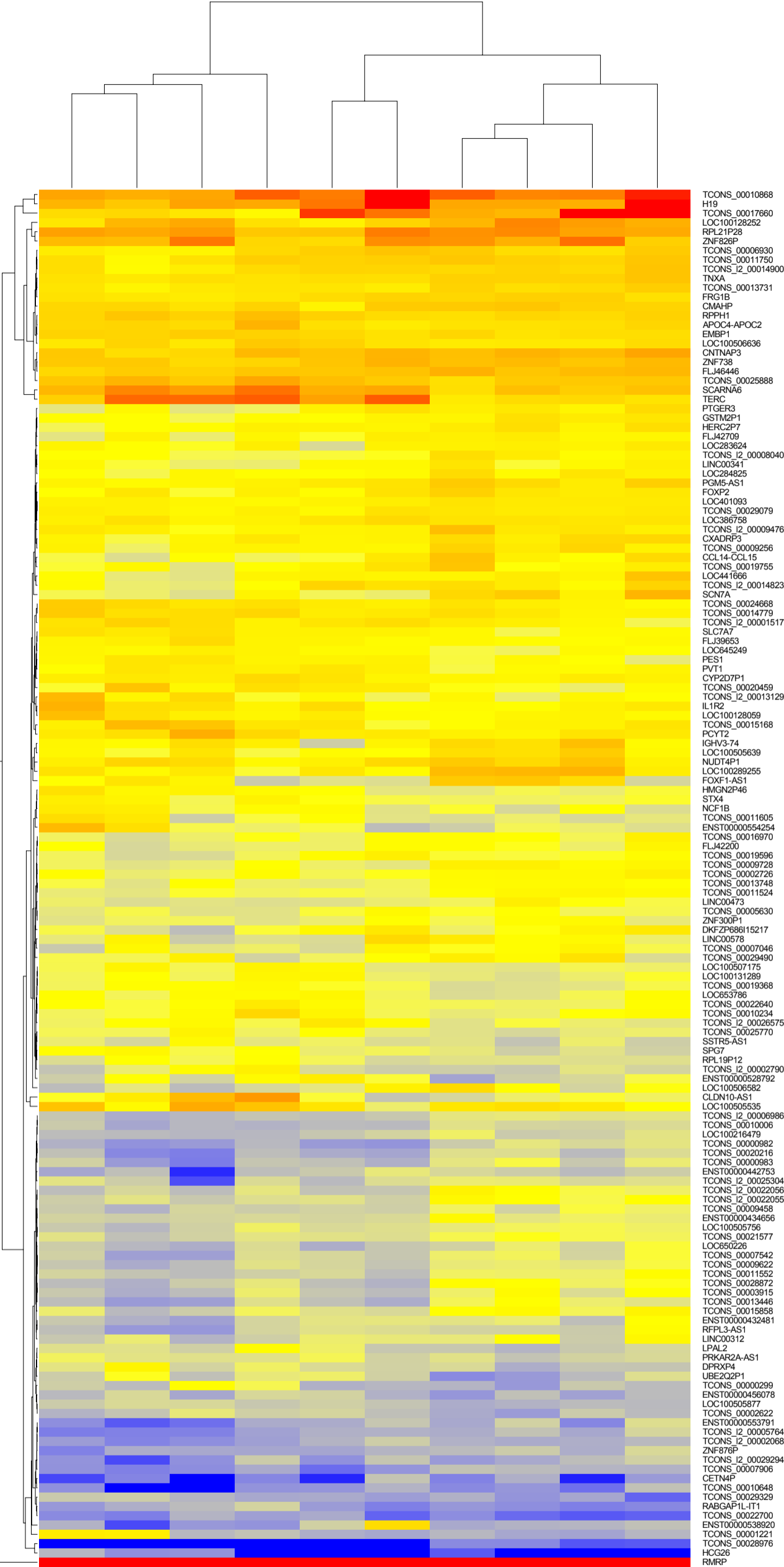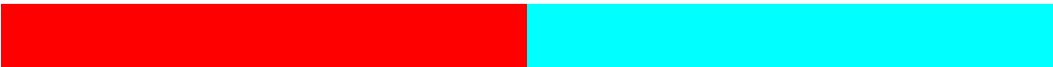

22A

42A

23A

40A

41A

41B

22B

42B

23B

40B

A

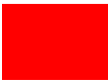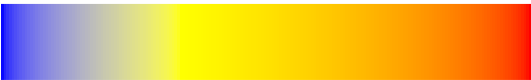

B

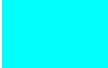

3.8

6.1

10.6

Supplement: Supplementary file 3 [file DataSheet_1.zip › RNA seq raw data/Heatmap/NoRowNormalization/diffLncRNA.pdf]

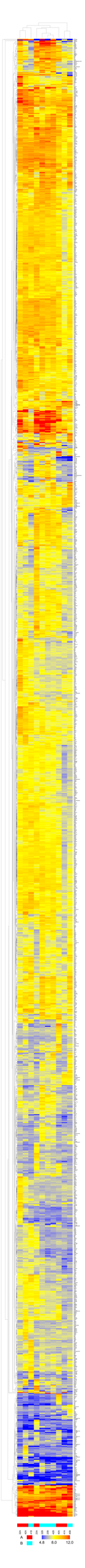

Supplement: Supplementary file 3 [file DataSheet_1.zip › RNA seq raw data/Heatmap/NoRowNormalization/varTop2000Coding.pdf]

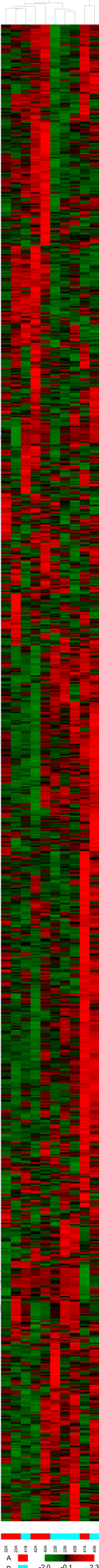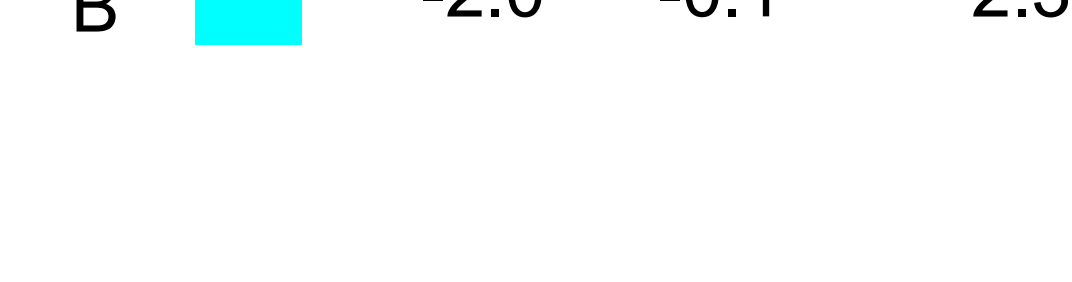

Supplement: Supplementary file 3 [file DataSheet_1.zip › RNA seq raw data/Heatmap/RowNormalization/AllsnoRNA.pdf]

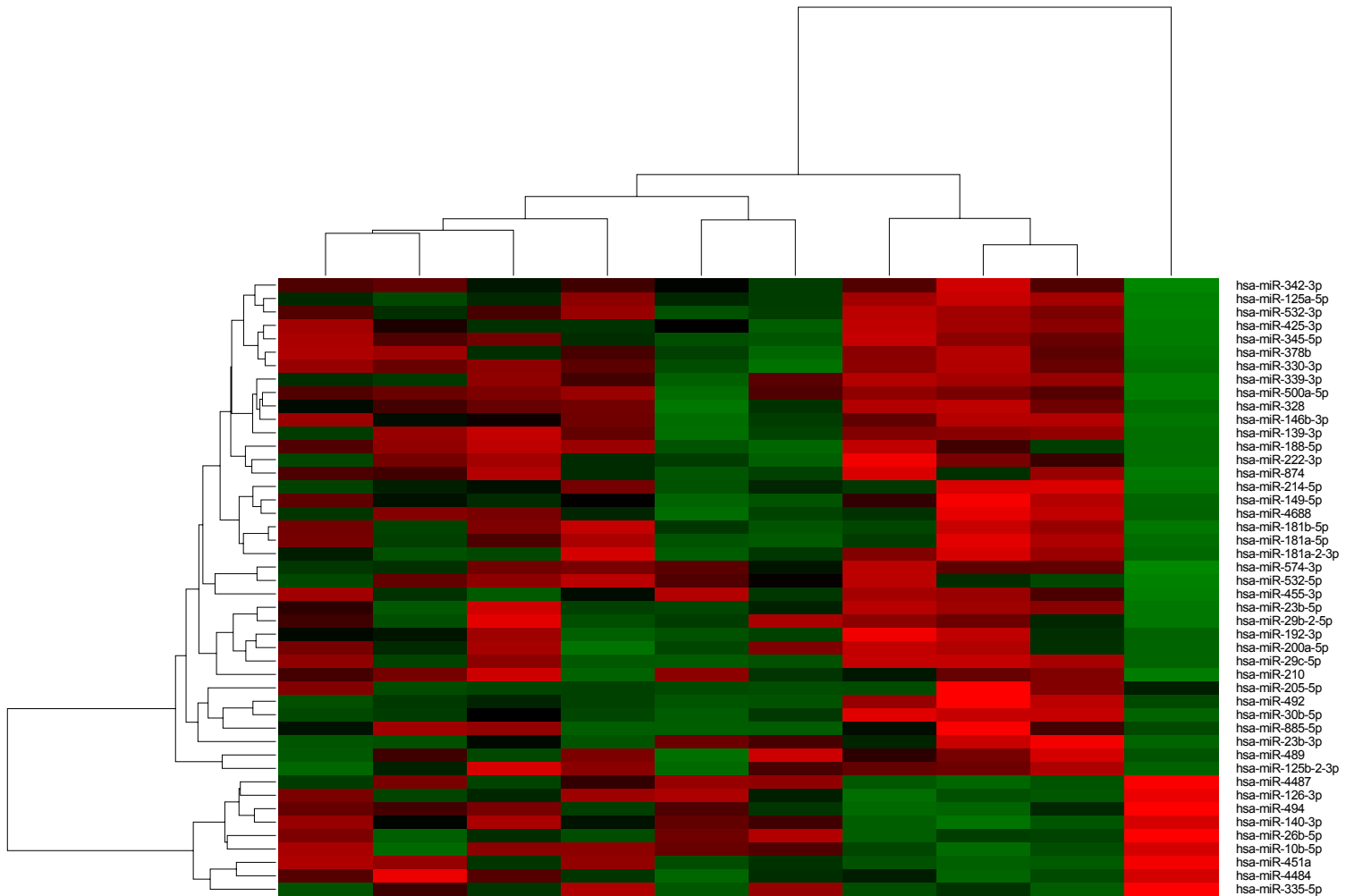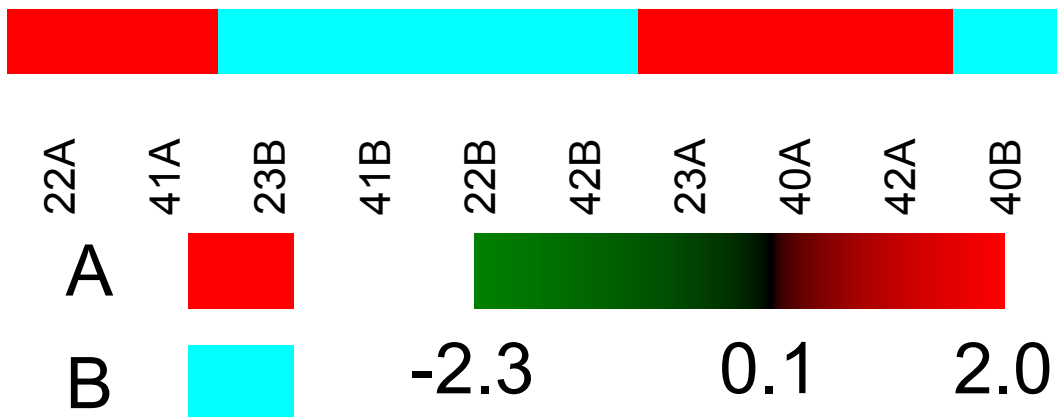

Supplement: Supplementary file 3 [file DataSheet_1.zip › RNA seq raw data/Heatmap/RowNormalization/diffMiRNA.pdf]

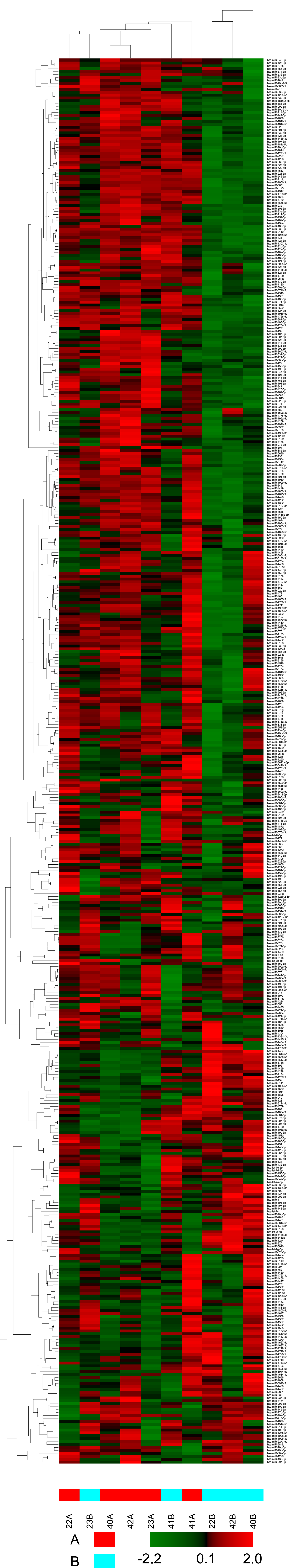

Supplement: Supplementary file 3 [file DataSheet_1.zip › RNA seq raw data/Heatmap/RowNormalization/AllmiRNA.pdf]

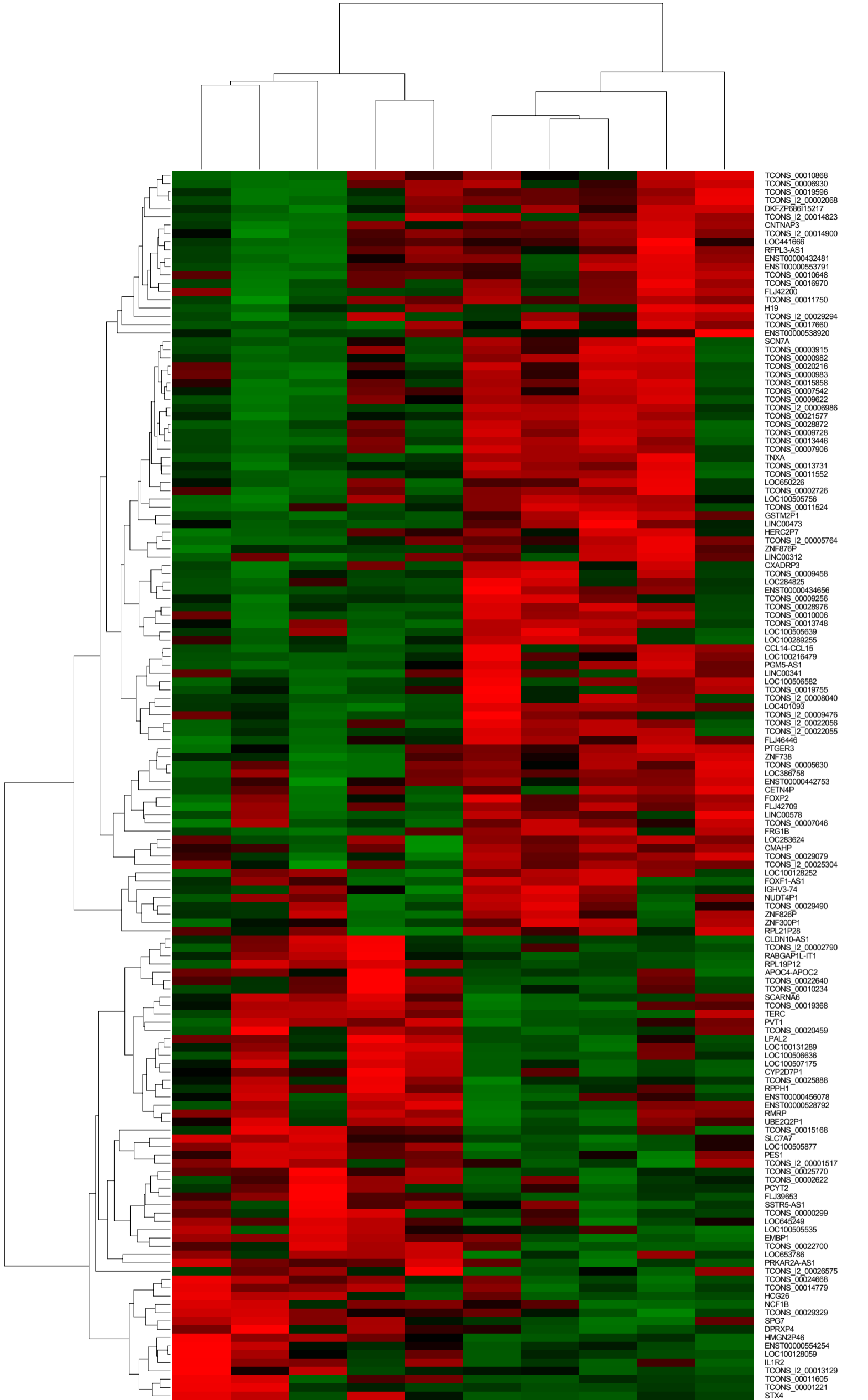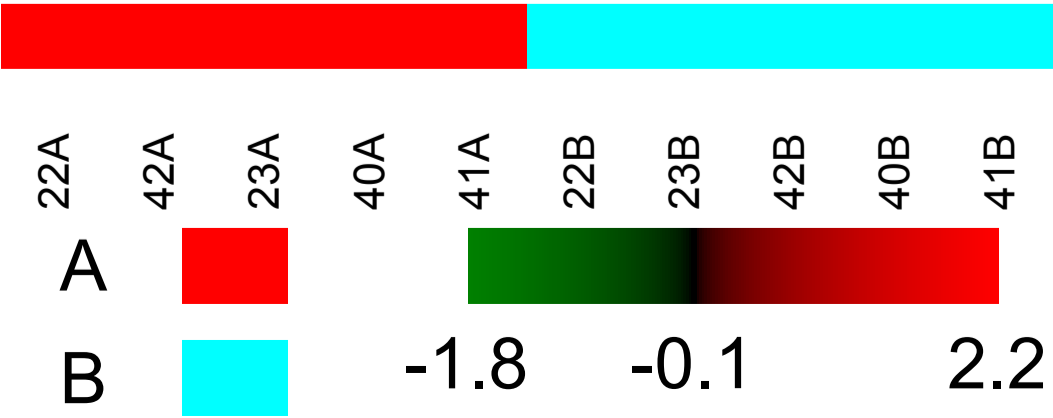

Supplement: Supplementary file 3 [file DataSheet_1.zip › RNA seq raw data/Heatmap/RowNormalization/diffLncRNA.pdf]

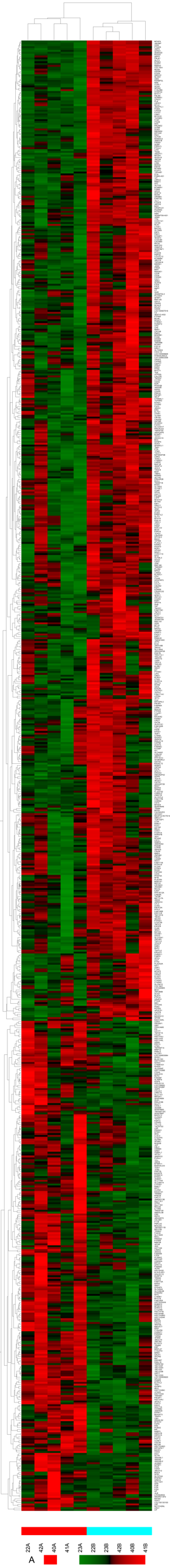

Supplement: Supplementary file 3 [file DataSheet_1.zip › RNA seq raw data/Heatmap/RowNormalization/diffCoding.pdf]

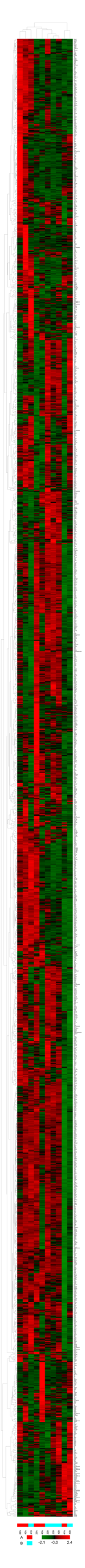

Supplement: Supplementary file 3 [file DataSheet_1.zip › RNA seq raw data/Heatmap/RowNormalization/varTop2000Coding.pdf]
