# Supplementary material for: Downregulation of miR-181b-5p Inhibits the Viability, Migration, and Glycolysis of Gallbladder Cancer by Upregulating PDHX Under Hypoxia
Source: Front Oncol. 2021 Aug 16;11:683725. doi: 10.3389/fonc.2021.683725 (PMC8415503; doi:10.3389/fonc.2021.683725)
Supplement: Supplementary file 3 [file DataSheet_1.zip › RNA seq raw data/tfbsEnrich/A vs B_dn RefSeq/Enrichment.pdf]

TF Name	p-value	q-value	GeneSymbol(s)	Family
FOXD3	0.00000	0.00000	FOXD3	Fork head
SRY	0.00000	0.00000	SRY	HMG
MEF-2A	0.00019	0.00445	MEF2A	Mef2
FOXF2	0.00026	0.00525	FOXF2	Fork head
POU3F2	0.00032	0.00525	POU3F2	POU
POU3F2 (N-Oct-5a)	0.00037	0.00536	POU3F2	POU
NF-AT1	0.00045	0.00536	NFATC2	RHD
Nkx6-1	0.00048	0.00536	NKX6-1	Homeobox
NF-AT2	0.00049	0.00536	NFATC1	RHD
POU3F2 (N-Oct-5b)	0.00062	0.00636	POU3F2	POU
TGIF	0.00149	0.01358	TGIF1	Homeobox
E4BP4	0.00265	0.02287	NFIL3	bZIP
Nkx2-2	0.00462	0.03537	NKX2-2	Homeobox
FOXL1	0.00469	0.03537	FOXL1	Fork head
GATA-1	0.00486	0.03537	GATA1	zf-GATA
LUN-1	0.00496	0.03537	TOPORS	
HOXA9B	0.00584	0.03854	HOXA9	Homeobox
FAC1	0.00589	0.03854	BPTF	
Pbx1a	0.00611	0.03854	PBX1	Homeobox
Meis-1	0.00648	0.03936	MEIS1	Homeobox
HNF-1A	0.00686	0.04018	HNF1A	Homeobox
ATF-2	0.00842	0.04762	ATF2	bZIP
FOXO4	0.00914	0.04997	FOXO4	Fork head
Cdc5	0.00960	0.05079	CDC5L	MYB
Cart-1	0.01064	0.05453	ALX1	Homeobox
FOXC1	0.01103	0.05482	FOXC1	Fork head
c-Jun	0.01388	0.06695	JUN	bZIP
SRF	0.01500	0.07029	SRF	MADS-box
C/EBPbeta	0.02538	0.11562	CEBPB	C/EBP
ISGF-3	0.03239	0.14357	STAT1, STAT2, IRF9	
RSRFC4	0.03519	0.15187	MEF2A	Mef2
Nkx3-1 v1	0.03995	0.16041	NKX3-1	Homeobox
Nkx3-1 v4	0.04096	0.16041	NKX3-1	Homeobox
Nkx3-1	0.04146	0.16041	NKX3-1	Homeobox
Nkx3-1 v2	0.04165	0.16041	NKX3-1	Homeobox
Nkx3-1 v3	0.04206	0.16041	NKX3-1	Homeobox
TBP	0.04869	0.18148	TBP	
RORalpha1	0.04997	0.18211	RORA	RAR-related orphan receptor
