# Supplementary material for: Downregulation of miR-181b-5p Inhibits the Viability, Migration, and Glycolysis of Gallbladder Cancer by Upregulating PDHX Under Hypoxia
Source: Front Oncol. 2021 Aug 16;11:683725. doi: 10.3389/fonc.2021.683725 (PMC8415503; doi:10.3389/fonc.2021.683725)
Supplement: Supplementary file 3 [file DataSheet_1.zip › RNA seq raw data/tfbsEnrich/A vs B_up RefSeq/Enrichment.pdf]

TF Name	p-value	q-value	GeneSymbol(s)	Family
AP-1	0.00011	0.00671	FOS, FOSL1, JUN, JUNB, JUND	
PPAR-gamma2	0.00011	0.00671	PPARG	PPAR receptor
PPAR-gamma1	0.00017	0.00671	PPARG	PPAR receptor
POU3F2 (N-Oct-5b)	0.00029	0.00671	POU3F2	POU
oct-B2	0.00038	0.00671	HOXB1	Homeobox
POU2F2 (Oct-2.1)	0.00038	0.00671	POU2F2	POU
POU3F2 (N-Oct-5a)	0.00038	0.00671	POU3F2	POU
Oct-B1	0.00041	0.00671	HOXB1	Homeobox
POU2F2	0.00042	0.00671	POU2F2	POU
POU2F2B	0.00042	0.00671	POU2F2	POU
POU2F2C	0.00045	0.00671	POU2F2	POU
oct-B3	0.00053	0.00724	HOXB1	Homeobox
CUTL1	0.00059	0.00744	CUX1	CUT
Egr-2	0.00100	0.01171	EGR2	zf-C2H2
Max	0.00140	0.01531	MAX	bHLH
Pax-2	0.00151	0.01548	PAX2	PAX
TBP	0.00317	0.03058	TBP	
c-Fos	0.00344	0.03134	FOS	bZIP
c-Myc	0.00381	0.03239	MYC	bHLH
c-Jun	0.00395	0.03239	JUN	bZIP
POU3F2	0.00548	0.04280	POU3F2	POU
STAT5B	0.00584	0.04353	STAT5B	STAT
Egr-1	0.00639	0.04556	EGR1	zf-C2H2
FAC1	0.00861	0.05884	BPTF	
Max1	0.01366	0.08961	MAX	bHLH
STAT4	0.01610	0.10155	STAT4	STAT
ZID	0.02177	0.13223	ZBTB6	ZBTB
Egr-3	0.02328	0.13635	EGR3	zf-C2H2
LHX3b	0.02842	0.16072	LHX3	Homeobox
LHX3a	0.02974	0.16258	LHX3	Homeobox
E4BP4	0.03139	0.16606	NFIL3	bZIP
HNF-4alpha2	0.03719	0.19060	HNF4A	Thyroid hormone receptor
USF1	0.04334	0.21208	USF1	bHLH
STAT5A	0.04437	0.21208	STAT5A	STAT
C/EBPbeta	0.04526	0.21208	CEBPB	C/EBP
GATA-1	0.04795	0.21574	GATA1	zf-GATA
GATA-2	0.04888	0.21574	GATA2	zf-GATA
