# Supplementary material for: Downregulation of miR-181b-5p Inhibits the Viability, Migration, and Glycolysis of Gallbladder Cancer by Upregulating PDHX Under Hypoxia
Source: Front Oncol. 2021 Aug 16;11:683725. doi: 10.3389/fonc.2021.683725 (PMC8415503; doi:10.3389/fonc.2021.683725)
Supplement: Supplementary file 3 [file DataSheet_1.zip › RNA seq raw data/tfbsEnrich/README.pdf]

转录因子富集分析说明

KC-TBY

October 18, 2013

1 概述

转录因子构成、作用机理多种多样，且命名方式无统一标准，目前为止尚未有注释良好的数据库。本分析使用的数据基于 UCSC 归类整理的TFBS Conserved track 中的位点信息，注释信息由康成专家对其中的所有 164 个转录因子名称进行手工文献与位点追踪，整合多个数据库内容后梳理并纠错所得。

2 原理

某一转录因子激活后，则包含该转录因子位点的所有转录本都会有差异表达 (可能激活或抑制) 的趋势，度量该趋势的强弱，即可反映该转录因子的活性。分析的原理基于如下假设：差异基因中的该转录因子的位点数应显著多于从背景基因中 (该实验条件下表达但并非差异表达) 随机抽样估计得到的期望位点数。

3 结果解读

每一次检测包含结果文件两份，一份为显著富集的转录因子表格，一份为差异基因中富集转录因子的位点分布统计。

3.1 转录因子富集表格

该表格列出所有经过 10^5 次随机取样后，$p < 0.05$ 的转录因子，即在当前分组下，显著富集的转录因子。表格中蓝色字体为超链接，点击可外链至相关数据库查看详细信息。$q-$value 为 BH 校正后的 FDR。GeneSymbol(s) 为转录因子 (可能为复合物) 对应的基因，Family 为该转录因子所属分类。

3.2 位点分布统计

该表格 (图3) 统计了所有包含富集转录因子的差异基因序列，给出了该转录本功能、基因组位置、启动子区间 (默认取 tss 上游 5kbp，下游 1kb) 坐标，启动子区所有富集位点总数与各个富集转录因子的分别计数。

其中蓝色字体项为超链接，点击可外链至相关数据库查看详细信息。注：由于 Excel 的限制，Pathway 项只能显示一条外链，用户可点击该外链，再点击天蓝色项 (命中基因)，便可获取此基因命中的所有 pathway 信息。

4 其他建议

对于结果的解读，建议客户再参考相关文献中列出的 GO term (如GO:0001228，见图3)，来确定后继的实验与分析。

TF Name	p-value	q-value	GeneSymbol(s)	Family
Cdc5	0.01856	0.76223	CDC5L	MYB
LCR-F1	0.02794	0.76223	NFE2L1	bZIP
RSRFC4	0.03685	0.76223	MEF2A	Mef2
E4BP4	0.03779	0.76223	NFIL3	bZIP

图 1: 富集位点于差异基因上的统计

Transcript Annotations					Genome Position				Promoter Region				
Seqname	GeneSymbo	Pathway	Antibody	Genome Fe	Chromosom	Strand	Transcript	Transcript	Promoter S	Promoter E	Total Sites	GATA-6	TBP
NM_01340(	FST	TGF-beta si	ENSG00000	chr5:527750	chr5	+	52776264	52782304	52771264	52777263	23	1	2
NM_00315:	STC1		ENSG00000	chr8:236968	chr8	-	23699434	23712320	23711321	23717320	18	1	2
NM_14497(	CXorf38		ENSG00000	chrX:40482(	chrX	-	40486173	40506819	40505820	40511819	10	0	1

图 2: 富集位点于差异基因上的统计

<table>
<tr><td rowspan="2" colspan="2"></td><td colspan="3">Transcription regulation terms (BP)</td></tr>
<tr><td>GO:0006357 regulation of transcription from RNA polymerase II promoter</td><td>GO:0045944 positive regulation of transcription from RNA polymerase II promoter</td><td>GO:0000122 negative regulation of transcription from RNA polymerase II promoter</td></tr>
<tr><td></td><td>Binding terms (MF)</td><td colspan="3"></td></tr>
<tr><td rowspan="5">Specific DNA binding terms (M)</td><td>GO:0043565 sequence-specific DNA binding</td><td rowspan="3">GO: 0000981 sequence-specific DNA binding RNA polymerase II transcription factor activity</td><td rowspan="3">GO:0001228 RNA polymerase II transcription regulatory region sequence-specific DNA binding transcription factor activity involved in positive regulation of transcription</td><td rowspan="3">GO:0001227 RNA polymerase II transcription regulatory region sequence-specific DNA binding transcription factor activity involved in negative regulation of transcription</td></tr>
<tr><td>GO:0000976 transcription regulatory region sequence-specific DNA binding</td></tr>
<tr><td>GO:0000977 RNA polymerase II regulatory region sequence-specific DNA binding</td></tr>
<tr><td>GO:0000978 RNA polymerase II core promoter proximal region sequence-specific DNA binding</td><td>GO:0000982 RNA polymerase II core promoter proximal region sequence-specific DNA binding transcription factor activity</td><td>GO:0001077 RNA polymerase II core promoter proximal region sequence-specific DNA binding transcription factor activity involved in positive regulation of transcription</td><td>GO:0001078 RNA polymerase II core promoter proximal region sequence-specific DNA binding transcription factor activity involved in negative regulation of transcription</td></tr>
<tr><td>GO:0000980 RNA polymerase II distal enhancer sequence-specific DNA binding</td><td>GO:0003705 sequence-specific distal enhancer binding RNA polymerase II transcription factor activity</td><td>GO:0001205 RNA polymerase II distal enhancer sequence-specific DNA binding transcription factor activity involved in positive regulation of transcription</td><td>GO:0001206 RNA polymerase II distal enhancer sequence-specific DNA binding transcription factor activity involved in negative regulation of transcription</td></tr>
<tr><td rowspan="2">TF binding terms (MF)</td><td>GO: 0008134 Transcription factor binding</td><td rowspan="2">GO: 0001076 RNA polymerase II transcription factor binding transcription factor activity</td><td rowspan="2">GO:0001190 RNA polymerase II transcription factor binding transcription factor activity involved in positive regulation of transcription</td><td rowspan="2">GO:0001191 RNA polymerase II transcription factor binding transcription factor activity involved in negative regulation of transcription</td></tr>
<tr><td>GO: 0001085 RNA polymerase II transcription factor binding</td></tr>
</table>

图 3: 转录因子相关 GO Term
