# Supplementary material for: Downregulation of miR-181b-5p Inhibits the Viability, Migration, and Glycolysis of Gallbladder Cancer by Upregulating PDHX Under Hypoxia
Source: Front Oncol. 2021 Aug 16;11:683725. doi: 10.3389/fonc.2021.683725 (PMC8415503; doi:10.3389/fonc.2021.683725)
Supplement: Supplementary file 3 [file DataSheet_1.zip › RNA seq raw data/HuGene 2.0 ST Data/Pathway Analysis/A vs B_up/hsa_pathwayResult.html]

| PathwayID | Definition | OriginalWebSite | Fisher-Pvalue | SelectionCounts | SelectionSize | Count | Size | FDR | Enrichment\_Score | Genes |
| --- | --- | --- | --- | --- | --- | --- | --- | --- | --- | --- |
| hsa05322 | Systemic lupus erythematosus - Homo sapiens (human) | http://www.genome.jp/kegg-bin/show\_pathway?hsa05322+733+8334+8330+3018+8347+8339+8348+8357+8354+8356+8363+8368+723790+440689+653604+554313 | 5.365601e-009 | 16 | 124 | 137 | 6462 | 1.411153e-006 | 8.270382e+000 | C8G//HIST1H2AC//HIST1H2AK//HIST1H2BB//HIST1H2BC//HIST1H2BG//HIST1H2BO//HIST1H3H//HIST1H3I//HIST1H3J//HIST1H4J//HIST1H4L//HIST2H2AA4//HIST2H2BF//HIST2H3D//HIST2H4B |
| hsa05034 | Alcoholism - Homo sapiens (human) | http://www.genome.jp/kegg-bin/show\_pathway?hsa05034+8334+8330+3018+8347+8339+8348+8357+8354+8356+8363+8368+723790+440689+653604+554313 | 1.377972e-006 | 15 | 124 | 179 | 6462 | 1.812033e-004 | 5.860760e+000 | HIST1H2AC//HIST1H2AK//HIST1H2BB//HIST1H2BC//HIST1H2BG//HIST1H2BO//HIST1H3H//HIST1H3I//HIST1H3J//HIST1H4J//HIST1H4L//HIST2H2AA4//HIST2H2BF//HIST2H3D//HIST2H4B |
| hsa05203 | Viral carcinogenesis - Homo sapiens (human) | http://www.genome.jp/kegg-bin/show\_pathway?hsa05203+898+1029+3018+8347+8339+8348+8363+8368+440689+554313+27044 | 1.983330e-003 | 11 | 124 | 207 | 6462 | 1.738720e-001 | 2.702605e+000 | CCNE1//CDKN2A//HIST1H2BB//HIST1H2BC//HIST1H2BG//HIST1H2BO//HIST1H4J//HIST1H4L//HIST2H2BF//HIST2H4B//SND1 |
| hsa04976 | Bile secretion - Homo sapiens (human) | http://www.genome.jp/kegg-bin/show\_pathway?hsa04976+366+6344+10998+6513+6822 | 1.205772e-002 | 5 | 124 | 72 | 6462 | 7.927952e-001 | 1.918735e+000 | AQP9//SCTR//SLC27A5//SLC2A1//SULT2A1 |
| hsa05202 | Transcriptional misregulation in cancer - Homo sapiens (human) | http://www.genome.jp/kegg-bin/show\_pathway?hsa05202+1050+8357+8354+8356+653604+3486+7850+3728 | 2.199507e-002 | 8 | 124 | 180 | 6462 | 1.000000e+000 | 1.657675e+000 | CEBPA//HIST1H3H//HIST1H3I//HIST1H3J//HIST2H3D//IGFBP3//IL1R2//JUP |
| hsa04060 | Cytokine-cytokine receptor interaction - Homo sapiens (human) | http://www.genome.jp/kegg-bin/show\_pathway?hsa04060+6369+414062+6374+6372+55540+7850+8794+4982+8764+9966 | 3.481515e-002 | 10 | 124 | 271 | 6462 | 1.000000e+000 | 1.458232e+000 | CCL24//CCL3L3//CXCL5//CXCL6//IL17RB//IL1R2//TNFRSF10C//TNFRSF11B//TNFRSF14//TNFSF15 |
| hsa04115 | p53 signaling pathway - Homo sapiens (human) | http://www.genome.jp/kegg-bin/show\_pathway?hsa04115+898+1029+3486+55240 | 4.104370e-002 | 4 | 124 | 68 | 6462 | 1.000000e+000 | 1.386754e+000 | CCNE1//CDKN2A//IGFBP3//STEAP3 |
| hsa00120 | Primary bile acid biosynthesis - Homo sapiens (human) | http://www.genome.jp/kegg-bin/show\_pathway?hsa00120+80270+10998 | 4.116533e-002 | 2 | 124 | 17 | 6462 | 1.000000e+000 | 1.385468e+000 | HSD3B7//SLC27A5 |
| hsa04610 | Complement and coagulation cascades - Homo sapiens (human) | http://www.genome.jp/kegg-bin/show\_pathway?hsa04610+733+2243+2244+2266 | 4.294583e-002 | 4 | 124 | 69 | 6462 | 1.000000e+000 | 1.367079e+000 | C8G//FGA//FGB//FGG |
| hsa05200 | Pathways in cancer - Homo sapiens (human) | http://www.genome.jp/kegg-bin/show\_pathway?hsa05200+23624+898+1029+1050+7855+3728+3909+2122+6513+9618+7474 | 4.863190e-002 | 11 | 124 | 327 | 6462 | 1.000000e+000 | 1.313079e+000 | CBLC//CCNE1//CDKN2A//CEBPA//FZD5//JUP//LAMA3//MECOM//SLC2A1//TRAF4//WNT5A |
