# Supplementary material for: Downregulation of miR-181b-5p Inhibits the Viability, Migration, and Glycolysis of Gallbladder Cancer by Upregulating PDHX Under Hypoxia
Source: Front Oncol. 2021 Aug 16;11:683725. doi: 10.3389/fonc.2021.683725 (PMC8415503; doi:10.3389/fonc.2021.683725)
Supplement: Supplementary file 3 [file DataSheet_1.zip › RNA seq raw data/HuGene 2.0 ST Data/Pathway Analysis/A vs B_down/hsa_pathwayResult.html]

| PathwayID | Definition | OriginalWebSite | Fisher-Pvalue | SelectionCounts | SelectionSize | Count | Size | FDR | Enrichment\_Score | Genes |
| --- | --- | --- | --- | --- | --- | --- | --- | --- | --- | --- |
| hsa05414 | Dilated cardiomyopathy - Homo sapiens (human) | http://www.genome.jp/kegg-bin/show\_pathway?hsa05414+70+55799+785+1674+1756+3479+8516+3680+3908+5350+6444+7169+7273 | 1.950572e-006 | 13 | 191 | 90 | 6462 | 5.130003e-004 | 5.709838e+000 | ACTC1//CACNA2D3//CACNB4//DES//DMD//IGF1//ITGA8//ITGA9//LAMA2//PLN//SGCD//TPM2//TTN |
| hsa05410 | Hypertrophic cardiomyopathy (HCM) - Homo sapiens (human) | http://www.genome.jp/kegg-bin/show\_pathway?hsa05410+70+55799+785+1674+1756+3479+8516+3680+3908+6444+7169+7273 | 6.259064e-006 | 12 | 191 | 85 | 6462 | 8.230669e-004 | 5.203491e+000 | ACTC1//CACNA2D3//CACNB4//DES//DMD//IGF1//ITGA8//ITGA9//LAMA2//SGCD//TPM2//TTN |
| hsa04514 | Cell adhesion molecules (CAMs) - Homo sapiens (human) | http://www.genome.jp/kegg-bin/show\_pathway?hsa04514+57863+947+1003+1272+8516+3680+58494+4685+257194+22871+6403+7412 | 1.141774e-003 | 12 | 191 | 145 | 6462 | 7.945877e-002 | 2.942420e+000 | CADM3//CD34//CDH5//CNTN1//ITGA8//ITGA9//JAM2//NCAM2//NEGR1//NLGN1//SELP//VCAM1 |
| hsa05412 | Arrhythmogenic right ventricular cardiomyopathy (ARVC) - Homo sapiens (human) | http://www.genome.jp/kegg-bin/show\_pathway?hsa05412+55799+785+1674+1756+8516+3680+3908+6444 | 1.427095e-003 | 8 | 191 | 74 | 6462 | 7.945877e-002 | 2.845547e+000 | CACNA2D3//CACNB4//DES//DMD//ITGA8//ITGA9//LAMA2//SGCD |
| hsa04810 | Regulation of actin cytoskeleton - Homo sapiens (human) | http://www.genome.jp/kegg-bin/show\_pathway?hsa04810+623+1073+1129+2255+2247+2252+2260+10788+8516+3680+4628+10398+80310+5295+4659 | 1.510623e-003 | 15 | 191 | 213 | 6462 | 7.945877e-002 | 2.820844e+000 | BDKRB1//CFL2//CHRM2//FGF10//FGF2//FGF7//FGFR1//IQGAP2//ITGA8//ITGA9//MYH10//MYL9//PDGFD//PIK3R1//PPP1R12A |
| hsa05205 | Proteoglycans in cancer - Homo sapiens (human) | http://www.genome.jp/kegg-bin/show\_pathway?hsa05205+287+857+960+2255+2247+2252+2260+2318+8322+2719+3479+3791+5295+4659+7078 | 2.702073e-003 | 15 | 191 | 226 | 6462 | 1.025627e-001 | 2.568303e+000 | ANK2//CAV1//CD44//FGF10//FGF2//FGF7//FGFR1//FLNC//FZD4//GPC3//IGF1//KDR//PIK3R1//PPP1R12A//TIMP3 |
| hsa04151 | PI3K-Akt signaling pathway - Homo sapiens (human) | http://www.genome.jp/kegg-bin/show\_pathway?hsa04151+284+1129+2255+2247+2252+2260+2690+2791+2788+3479+8516+3680+3791+3815+3908+1902+80310+5295+7010+7148 | 2.729805e-003 | 20 | 191 | 344 | 6462 | 1.025627e-001 | 2.563868e+000 | ANGPT1//CHRM2//FGF10//FGF2//FGF7//FGFR1//GHR//GNG11//GNG7//IGF1//ITGA8//ITGA9//KDR//KIT//LAMA2//LPAR1//PDGFD//PIK3R1//TEK//TNXB |
| hsa04610 | Complement and coagulation cascades - Homo sapiens (human) | http://www.genome.jp/kegg-bin/show\_pathway?hsa04610+2+623+718+730+1675+3075+2162 | 4.047803e-003 | 7 | 191 | 69 | 6462 | 1.301398e-001 | 2.392781e+000 | A2M//BDKRB1//C3//C7//CFD//CFH//F13A1 |
| hsa05218 | Melanoma - Homo sapiens (human) | http://www.genome.jp/kegg-bin/show\_pathway?hsa05218+2255+2247+2252+2260+3479+80310+5295 | 4.748826e-003 | 7 | 191 | 71 | 6462 | 1.301398e-001 | 2.323414e+000 | FGF10//FGF2//FGF7//FGFR1//IGF1//PDGFD//PIK3R1 |
| hsa04270 | Vascular smooth muscle contraction - Homo sapiens (human) | http://www.genome.jp/kegg-bin/show\_pathway?hsa04270+59+72+10203+3778+27345+4629+10398+5320+5332+4659 | 4.948282e-003 | 10 | 191 | 130 | 6462 | 1.301398e-001 | 2.305546e+000 | ACTA2//ACTG2//CALCRL//KCNMA1//KCNMB4//MYH11//MYL9//PLA2G2A//PLCB4//PPP1R12A |
| hsa04510 | Focal adhesion - Homo sapiens (human) | http://www.genome.jp/kegg-bin/show\_pathway?hsa04510+857+2318+3479+8516+3680+3791+3908+10398+55742+80310+5295+4659+7148 | 7.472265e-003 | 13 | 191 | 205 | 6462 | 1.786551e-001 | 2.126548e+000 | CAV1//FLNC//IGF1//ITGA8//ITGA9//KDR//LAMA2//MYL9//PARVA//PDGFD//PIK3R1//PPP1R12A//TNXB |
| hsa05144 | Malaria - Homo sapiens (human) | http://www.genome.jp/kegg-bin/show\_pathway?hsa05144+948+2532+3820+6403+7412 | 1.423792e-002 | 5 | 191 | 49 | 6462 | 3.120479e-001 | 1.846553e+000 | CD36//DARC//KLRB1//SELP//VCAM1 |
| hsa05416 | Viral myocarditis - Homo sapiens (human) | http://www.genome.jp/kegg-bin/show\_pathway?hsa05416+857+1756+3908+4628+4629+6444 | 2.036964e-002 | 6 | 191 | 73 | 6462 | 4.120934e-001 | 1.691017e+000 | CAV1//DMD//LAMA2//MYH10//MYH11//SGCD |
| hsa04975 | Fat digestion and absorption - Homo sapiens (human) | http://www.genome.jp/kegg-bin/show\_pathway?hsa04975+948+5320+8611+8613 | 3.195688e-002 | 4 | 191 | 41 | 6462 | 6.003328e-001 | 1.495436e+000 | CD36//PLA2G2A//PPAP2A//PPAP2B |
| hsa04512 | ECM-receptor interaction - Homo sapiens (human) | http://www.genome.jp/kegg-bin/show\_pathway?hsa04512+948+960+8516+3680+3908+7148 | 4.120944e-002 | 6 | 191 | 86 | 6462 | 7.225389e-001 | 1.385003e+000 | CD36//CD44//ITGA8//ITGA9//LAMA2//TNXB |
| hsa04020 | Calcium signaling pathway - Homo sapiens (human) | http://www.genome.jp/kegg-bin/show\_pathway?hsa04020+623+1129+1910+2774+5136+5332+5350+5733+5737+7417 | 4.413317e-002 | 10 | 191 | 183 | 6462 | 7.254389e-001 | 1.355235e+000 | BDKRB1//CHRM2//EDNRB//GNAL//PDE1A//PLCB4//PLN//PTGER3//PTGFR//VDAC2 |
