# Supplementary material for: Downregulation of miR-181b-5p Inhibits the Viability, Migration, and Glycolysis of Gallbladder Cancer by Upregulating PDHX Under Hypoxia
Source: Front Oncol. 2021 Aug 16;11:683725. doi: 10.3389/fonc.2021.683725 (PMC8415503; doi:10.3389/fonc.2021.683725)
Supplement: Supplementary file 3 [file DataSheet_1.zip › RNA seq raw data/HuGene 2.0 ST Data/GO Analysis/A vs B_up/MF_result(Human).html]

| GO.ID | Term | Ontology | Count | Pop.Hits | List.Total | Pop.Total | Fold.Enrichment | Pvalue | FDR | Enrichment.Score | GENES |
| --- | --- | --- | --- | --- | --- | --- | --- | --- | --- | --- | --- |
| GO:0043498 | cell surface binding | Molecular function | 7 | 52 | 217 | 15325 | 9.50682382133995 | 8.06951941188898e-06 | 0.00782743382953231 | 5.09315232933985 | FGA//FGB//FGG//LBP//CRP//ALB//MIF |
| GO:0016722 | oxidoreductase activity, oxidizing metal ions | Molecular function | 3 | 11 | 217 | 15325 | 19.2605781315459 | 0.000424832545047274 | 0.194652476326548 | 3.37178222075652 | CYB561//STEAP3//CP |
| GO:0046982 | protein heterodimerization activity | Molecular function | 14 | 360 | 217 | 15325 | 2.74641577060932 | 0.000629894757487656 | 0.194652476326548 | 3.20073200619792 | ALOX5AP//APOB//BIK//CEBPA//HIST1H2BB//HIST1H2AK//HIST1H2AC//HIST1H2BC//HIST1H2BO//UGT1A9//HIST2H2BF//HIST2H4B//HIST2H3D//HIST2H2AA4 |
| GO:0005544 | calcium-dependent phospholipid binding | Molecular function | 4 | 30 | 217 | 15325 | 9.41628264208909 | 0.000802690624027003 | 0.194652476326548 | 3.09545180984884 | ANXA8L2//ANXA3//ANXA8//ANXA8L1 |
| GO:0042379 | chemokine receptor binding | Molecular function | 5 | 55 | 217 | 15325 | 6.42019271051529 | 0.00106364666200175 | 0.206347452428339 | 2.97320261849602 | CCL24//CXCL6//CXCL5//CCL3L3//S100A14 |
| GO:0004867 | serine-type endopeptidase inhibitor activity | Molecular function | 6 | 95 | 217 | 15325 | 4.46034440941062 | 0.00229516535423715 | 0.371051732268339 | 2.63918602043354 | ITIH2//PI3//SERPINB3//SERPINB4//SPINK1//SERPINB7 |
| GO:0005125 | cytokine activity | Molecular function | 9 | 207 | 217 | 15325 | 3.07052694850731 | 0.00279482282365257 | 0.38728259127757 | 2.55364571877897 | CCL24//CXCL6//CXCL5//CCL3L3//GDF2//MIF//TNFRSF11B//WNT5A//TNFSF15 |
| GO:0043499 | eukaryotic cell surface binding | Molecular function | 3 | 23 | 217 | 15325 | 9.21158084552194 | 0.00402317501425218 | 0.466889304913779 | 2.39543107454514 | FGA//FGB//FGG |
| GO:0008009 | chemokine activity | Molecular function | 4 | 47 | 217 | 15325 | 6.01039317580155 | 0.00433196262291135 | 0.466889304913779 | 2.36331529921509 | CCL24//CXCL6//CXCL5//CCL3L3 |
| GO:0033293 | monocarboxylic acid binding | Molecular function | 4 | 50 | 217 | 15325 | 5.64976958525346 | 0.00541230246069143 | 0.515275474569496 | 2.26661794131705 | UGT1A9//ALB//APOC1//ALOX5AP |
| GO:0005509 | calcium ion binding | Molecular function | 18 | 662 | 217 | 15325 | 1.92023890737466 | 0.00623144337191001 | 0.515275474569496 | 2.20541134726496 | ADAM8//ANXA8L2//ANXA3//CDH2//CDH3//CDH4//DSG3//MMP12//CELSR1//CGREF1//SULF1//CBLC//CDHR2//S100A14//MMP28//NOTCH2NL//ANXA8//ANXA8L1 |
| GO:0005504 | fatty acid binding | Molecular function | 3 | 27 | 217 | 15325 | 7.84690220174091 | 0.00637454195343706 | 0.515275474569496 | 2.19555101626524 | ALOX5AP//ALB//APOC1 |
| GO:0005097 | Rab GTPase activator activity | Molecular function | 4 | 54 | 217 | 15325 | 5.23126813449394 | 0.00711382407341172 | 0.530800719323798 | 2.14789687922491 | TBC1D3F//TBC1D3B//TBC1D3G//TBC1D3 |
| GO:0008289 | lipid binding | Molecular function | 19 | 731 | 217 | 15325 | 1.83559545348522 | 0.00796464078842486 | 0.551835826055151 | 2.0988338064445 | LBP//UGT1A9//SULT1E1//ALB//APOC1//APOB//GRB14//ARHGEF5//OSBPL7//ANXA8L2//ANXA3//ANXA8//ANXA8L1//CRP//C8G//GRB7//NCF1B//ALOX5AP//MGLL |
| GO:0004859 | phospholipase inhibitor activity | Molecular function | 2 | 12 | 217 | 15325 | 11.7703533026114 | 0.0119971793455317 | 0.775817597677717 | 1.92092084884102 | ANXA3//APOC1 |
| GO:0016863 | intramolecular oxidoreductase activity, transposing C=C bonds | Molecular function | 2 | 13 | 217 | 15325 | 10.8649415101028 | 0.0140473727446083 | 0.851621972641878 | 1.85240489349644 | EBP//MIF |
| GO:0015645 | fatty acid ligase activity | Molecular function | 2 | 14 | 217 | 15325 | 10.0888742593812 | 0.0162372293205846 | 0.896297357633001 | 1.78948807567604 | SLC27A5//ACSM3 |
| GO:0004857 | enzyme inhibitor activity | Molecular function | 10 | 322 | 217 | 15325 | 2.19323353464808 | 0.0166323221004062 | 0.896297357633001 | 1.77904711307807 | APOC1//CDKN2A//ITIH2//PI3//SPINK1//SERPINB3//SERPINB4//SERPINB7//ANXA3//UGT1A9 |
| GO:0016411 | acylglycerol O-acyltransferase activity | Molecular function | 2 | 15 | 217 | 15325 | 9.41628264208909 | 0.018562420601831 | 0.947660420198741 | 1.7313653909689 | MOGAT3//PNPLA3 |
| GO:0051635 | bacterial cell surface binding | Molecular function | 2 | 16 | 217 | 15325 | 8.82776497695852 | 0.0210187117087462 | 0.970864302737324 | 1.67739390652513 | LBP//CRP |
| GO:0055102 | lipase inhibitor activity | Molecular function | 2 | 16 | 217 | 15325 | 8.82776497695852 | 0.0210187117087462 | 0.970864302737324 | 1.67739390652513 | APOC1//ANXA3 |
| GO:0051087 | chaperone binding | Molecular function | 3 | 45 | 217 | 15325 | 4.70814132104455 | 0.0256935514934242 | 1 | 1.5901758611913 | ALB//CP//FGB |
| GO:0090484 | drug transporter activity | Molecular function | 2 | 18 | 217 | 15325 | 7.84690220174091 | 0.0263081112966879 | 1 | 1.57991032952241 | MFSD10//EBP |
| GO:0019900 | kinase binding | Molecular function | 11 | 400 | 217 | 15325 | 1.94210829493088 | 0.0274468012059593 | 1 | 1.56150826321939 | PARD6A//CCNE1//CDKN2A//GRB7//JUP//PTPRR//FZD5//TRAF4//PIK3IP1//PFKFB1//SLC2A1 |
| GO:0019200 | carbohydrate kinase activity | Molecular function | 2 | 19 | 217 | 15325 | 7.43390734901771 | 0.0291332022689433 | 1 | 1.53561177588398 | PFKFB1//GALK1 |
| GO:0004866 | endopeptidase inhibitor activity | Molecular function | 6 | 164 | 217 | 15325 | 2.58373609081713 | 0.029359076184037 | 1 | 1.53225761409954 | ITIH2//PI3//SERPINB3//SERPINB4//SPINK1//SERPINB7 |
| GO:0016538 | cyclin-dependent protein kinase regulator activity | Molecular function | 2 | 20 | 217 | 15325 | 7.06221198156682 | 0.0320733546760743 | 1 | 1.49385561315816 | CDKN2A//CCNE1 |
| GO:0016878 | acid-thiol ligase activity | Molecular function | 2 | 20 | 217 | 15325 | 7.06221198156682 | 0.0320733546760743 | 1 | 1.49385561315816 | SLC27A5//ACSM3 |
| GO:0061135 | endopeptidase regulator activity | Molecular function | 6 | 168 | 217 | 15325 | 2.52221856484529 | 0.0324854956835698 | 1 | 1.48831050211712 | ITIH2//PI3//SPINK1//SERPINB3//SERPINB4//SERPINB7 |
| GO:0005126 | cytokine receptor binding | Molecular function | 7 | 216 | 217 | 15325 | 2.2886798088411 | 0.0343376189007945 | 1 | 1.46422982373827 | TNFSF15//CCL24//CXCL6//CXCL5//CCL3L3//S100A14//MIF |
| GO:0030414 | peptidase inhibitor activity | Molecular function | 6 | 171 | 217 | 15325 | 2.47796911633924 | 0.0349673940878667 | 1 | 1.45633673187232 | ITIH2//PI3//SPINK1//SERPINB3//SERPINB4//SERPINB7 |
| GO:0008146 | sulfotransferase activity | Molecular function | 3 | 51 | 217 | 15325 | 4.15424234209813 | 0.0354681465610757 | 1 | 1.45016150561683 | SULT1B1//SULT1E1//SULT2A1 |
| GO:0017048 | Rho GTPase binding | Molecular function | 3 | 51 | 217 | 15325 | 4.15424234209813 | 0.0354681465610757 | 1 | 1.45016150561683 | KCTD13//NCF2//PARD6A |
| GO:0046983 | protein dimerization activity | Molecular function | 20 | 918 | 217 | 15325 | 1.53860827485116 | 0.0366361186727035 | 1 | 1.43609054287304 | ALOX5AP//CEBPA//CRP//MECOM//JUP//SLC11A1//MGLL//UGT1A9//APOB//BIK//HIST1H2BB//HIST1H2AK//HIST1H2AC//HIST1H2BC//HIST1H2BO//HIST2H2BF//HIST2H4B//HIST2H3D//HIST2H2AA4//CELSR1 |
| GO:0030674 | protein binding, bridging | Molecular function | 5 | 130 | 217 | 15325 | 2.7162353775257 | 0.0375739052485545 | 1 | 1.42511366413927 | GRB7//GRB14//FGA//FGB//FGG |
| GO:0050997 | quaternary ammonium group binding | Molecular function | 2 | 23 | 217 | 15325 | 6.14105389701463 | 0.0415466687634727 | 1 | 1.3814637924729 | APOC1//CRP |
| GO:0005520 | insulin-like growth factor binding | Molecular function | 2 | 25 | 217 | 15325 | 5.64976958525346 | 0.0483701802785642 | 1 | 1.31542229396694 | IGFBP3//IGFBP1 |
| GO:0005507 | copper ion binding | Molecular function | 3 | 58 | 217 | 15325 | 3.65286826632767 | 0.0489472603139434 | 1 | 1.31027161160006 | ALB//CP//HEPH |
