# Supplementary material for: Downregulation of miR-181b-5p Inhibits the Viability, Migration, and Glycolysis of Gallbladder Cancer by Upregulating PDHX Under Hypoxia
Source: Front Oncol. 2021 Aug 16;11:683725. doi: 10.3389/fonc.2021.683725 (PMC8415503; doi:10.3389/fonc.2021.683725)
Supplement: Supplementary file 3 [file DataSheet_1.zip › RNA seq raw data/HuGene 2.0 ST Data/GO Analysis/A vs B_up/CC_result(Human).html]

| GO.ID | Term | Ontology | Count | Pop.Hits | List.Total | Pop.Total | Fold.Enrichment | Pvalue | FDR | Enrichment.Score | GENES |
| --- | --- | --- | --- | --- | --- | --- | --- | --- | --- | --- | --- |
| GO:0000786 | nucleosome | Cellular component | 10 | 67 | 233 | 17068 | 10.9333162513612 | 2.32667512772679e-08 | 1.34947157408154e-05 | 7.63326425276196 | HIST1H1B//HIST1H2BB//HIST1H2AK//HIST1H2AC//HIST1H2BC//HIST1H2BO//HIST2H2BF//HIST2H4B//HIST2H3D//HIST2H2AA4 |
| GO:0005576 | extracellular region | Cellular component | 59 | 2164 | 233 | 17068 | 1.99719959064838 | 1.01050089962416e-07 | 2.93045260891006e-05 | 6.99546329550678 | FGA//FGB//FGG//CHI3L1//MMP12//MUC4//TNFRSF11B//PI3//WNT5A//SPON2//MMP28//ALB//LAMA3//APOB//C8G//CP//CRP//GDF2//HP//IGFBP1//IGFBP3//LBP//MIF//REG3A//CCL24//CXCL6//CXCL5//TNFSF15//OLFM4//SULF1//SMPDL3B//LRG1//CCL3L3//APOC1//LSR//HPR//SERPINB3//EFNA1//IGHG1//IGHG3//IGHG4//ITIH2//LFNG//OLR1//SERPINB4//SPINK1//IL1R2//CGREF1//ACP6//TREM2//IL17RB//PRSS22//LPAL2//PVRL4//SIGLEC10//DHRS13//NOTCH2NL//HIST2H4B//HIST2H3D |
| GO:0032993 | protein-DNA complex | Cellular component | 11 | 110 | 233 | 17068 | 7.32532188841202 | 3.15381078963511e-07 | 6.09736752662788e-05 | 6.5011643653769 | HIST1H1B//HIST1H2BB//HIST1H2AK//HIST1H2AC//HIST1H2BC//HIST1H2BO//HIST2H2BF//HIST2H4B//HIST2H3D//HIST2H2AA4//JUP |
| GO:0044421 | extracellular region part | Cellular component | 37 | 1130 | 233 | 17068 | 2.39855672452429 | 6.05102278038227e-07 | 8.77398303155429e-05 | 6.21817121206911 | FGA//FGB//FGG//CHI3L1//MMP12//MUC4//TNFRSF11B//PI3//WNT5A//SPON2//MMP28//ALB//LAMA3//APOB//C8G//CP//CRP//GDF2//HP//IGFBP1//IGFBP3//LBP//MIF//REG3A//CCL24//CXCL6//CXCL5//TNFSF15//OLFM4//SULF1//SMPDL3B//LRG1//CCL3L3//APOC1//LSR//HPR//SERPINB3 |
| GO:0005615 | extracellular space | Cellular component | 30 | 848 | 233 | 17068 | 2.59150538505142 | 1.7002284620677e-06 | 0.000197226501599853 | 5.76949271794592 | FGA//FGB//FGG//IGFBP3//APOB//APOC1//LSR//HPR//ALB//C8G//CHI3L1//CP//CRP//GDF2//HP//IGFBP1//LBP//MIF//TNFRSF11B//REG3A//CCL24//CXCL6//CXCL5//WNT5A//TNFSF15//OLFM4//SULF1//SMPDL3B//LRG1//CCL3L3 |
| GO:0016323 | basolateral plasma membrane | Cellular component | 10 | 158 | 233 | 17068 | 4.63627967621014 | 6.35554959135452e-05 | 0.00614369793830937 | 4.19684688819853 | CLDN4//TACSTD2//SLC27A5//AQP9//CEACAM5//JUP//SLC2A1//STX4//SLC7A7//RAB17 |
| GO:0042581 | specific granule | Cellular component | 3 | 10 | 233 | 17068 | 21.9759656652361 | 0.000280761998525146 | 0.023263137020655 | 3.55166167484597 | ADAM8//ANXA3//STX4 |
| GO:0042627 | chylomicron | Cellular component | 3 | 12 | 233 | 17068 | 18.31330472103 | 0.00050442613016639 | 0.0365708944370633 | 3.29720242429336 | APOB//APOC1//LSR |
| GO:0031983 | vesicle lumen | Cellular component | 5 | 55 | 233 | 17068 | 6.65938353492002 | 0.000906435245393535 | 0.0530559833690117 | 3.04266321591791 | ALB//FGA//FGB//FGG//APOB |
| GO:0000785 | chromatin | Cellular component | 12 | 301 | 233 | 17068 | 2.92039410833702 | 0.000914758333948478 | 0.0530559833690117 | 3.03869362516319 | HIST1H1B//HIST1H2BB//HIST1H2AK//HIST1H2AC//HIST1H2BC//HIST1H2BO//HIST2H2BF//HIST2H4B//HIST2H3D//HIST2H2AA4//SIRT7//CDKN2A |
| GO:0043296 | apical junction complex | Cellular component | 7 | 117 | 233 | 17068 | 4.38267121528924 | 0.00112280977324554 | 0.0592026971347648 | 2.94969381580235 | JUP//CLDN4//TRAF4//F11R//PARD6A//MARVELD3//CLDN23 |
| GO:0034358 | plasma lipoprotein particle | Cellular component | 4 | 37 | 233 | 17068 | 7.91926690639137 | 0.00156957464947656 | 0.0758627747247004 | 2.80421802428711 | APOB//APOC1//LSR//HPR |
| GO:0032994 | protein-lipid complex | Cellular component | 4 | 38 | 233 | 17068 | 7.71086514569686 | 0.0017356594433023 | 0.0774371136242565 | 2.76053548446119 | APOB//APOC1//LSR//HPR |
| GO:0005911 | cell-cell junction | Cellular component | 11 | 292 | 233 | 17068 | 2.75953906755247 | 0.00231438311168493 | 0.0842824429910064 | 2.63556474844442 | CDH2//CDH3//JUP//CLDN4//TRAF4//F11R//PARD6A//MARVELD3//CLDN23//DSG3//SLC2A1 |
| GO:0034361 | very-low-density lipoprotein particle | Cellular component | 3 | 20 | 233 | 17068 | 10.987982832618 | 0.00241138461570342 | 0.0842824429910064 | 2.61773351415354 | APOB//APOC1//LSR |
| GO:0034385 | triglyceride-rich lipoprotein particle | Cellular component | 3 | 20 | 233 | 17068 | 10.987982832618 | 0.00241138461570342 | 0.0842824429910064 | 2.61773351415354 | APOB//APOC1//LSR |
| GO:0005923 | tight junction | Cellular component | 6 | 101 | 233 | 17068 | 4.35167636935367 | 0.00261566202385882 | 0.0842824429910064 | 2.58241837298374 | CLDN4//TRAF4//F11R//PARD6A//MARVELD3//CLDN23 |
| GO:0070160 | occluding junction | Cellular component | 6 | 101 | 233 | 17068 | 4.35167636935367 | 0.00261566202385882 | 0.0842824429910064 | 2.58241837298374 | CLDN4//TRAF4//F11R//PARD6A//MARVELD3//CLDN23 |
| GO:0031093 | platelet alpha granule lumen | Cellular component | 4 | 47 | 233 | 17068 | 6.23431650077618 | 0.00381175366757058 | 0.116358796167944 | 2.41887517316265 | ALB//FGA//FGB//FGG |
| GO:0005887 | integral to plasma membrane | Cellular component | 28 | 1216 | 233 | 17068 | 1.68675175062119 | 0.00457140189619289 | 0.131680417714424 | 2.3399505959003 | C8G//ITGAX//NCF2//ADAM8//AQP9//CDH4//CEACAM5//CLDN4//EFNA1//EFNA3//TACSTD2//MUC4//OLR1//P2RY2//SCTR//SLC11A1//TFR2//TNFRSF14//TNFRSF10C//SLC7A7//SLC16A5//CELSR1//TNFSF15//IGSF6//EBP//SLC39A14//OPN3//IL17RB |
| GO:0034774 | secretory granule lumen | Cellular component | 4 | 50 | 233 | 17068 | 5.86025751072961 | 0.00476773926207396 | 0.131680417714424 | 2.32168750329693 | ALB//FGA//FGB//FGG |
| GO:0009925 | basal plasma membrane | Cellular component | 3 | 26 | 233 | 17068 | 8.45229448662925 | 0.00517842835847226 | 0.136522202177905 | 2.28580202766974 | CLDN4//TACSTD2//SLC27A5 |
| GO:0060205 | cytoplasmic membrane-bounded vesicle lumen | Cellular component | 4 | 54 | 233 | 17068 | 5.42616436178668 | 0.00627603212701575 | 0.158265157985615 | 2.20231484192545 | ALB//FGA//FGB//FGG |
| GO:0031226 | intrinsic to plasma membrane | Cellular component | 28 | 1260 | 233 | 17068 | 1.627849308536 | 0.00733927886883437 | 0.176706344808778 | 2.13434661020267 | EFNA1//ADAM8//AQP9//CDH4//CEACAM5//CLDN4//EFNA3//TACSTD2//MUC4//OLR1//P2RY2//SCTR//SLC11A1//TFR2//TNFRSF14//TNFRSF10C//SLC7A7//SLC16A5//CELSR1//TNFSF15//IGSF6//EBP//SLC39A14//OPN3//IL17RB//C8G//ITGAX//NCF2 |
| GO:0016327 | apicolateral plasma membrane | Cellular component | 2 | 10 | 233 | 17068 | 14.650643776824 | 0.00776862435428637 | 0.176706344808778 | 2.10965587801188 | CLDN4//JUP |
| GO:0030141 | secretory granule | Cellular component | 9 | 252 | 233 | 17068 | 2.61618638871858 | 0.00792131890522109 | 0.176706344808778 | 2.10120250205043 | NCF2//FGA//FGB//FGG//ALB//ADAM8//ANXA3//STX4//SLC11A1 |
| GO:0031091 | platelet alpha granule | Cellular component | 4 | 60 | 233 | 17068 | 4.88354792560801 | 0.00908375947925299 | 0.195132611035805 | 2.04173437359542 | ALB//FGA//FGB//FGG |
| GO:0005886 | plasma membrane | Cellular component | 75 | 4312 | 233 | 17068 | 1.27411674775255 | 0.0100449540325574 | 0.202661560161544 | 1.99805204631766 | EFNA1//ADAM8//AQP9//CDH4//CEACAM5//CLDN4//EFNA3//TACSTD2//MUC4//OLR1//P2RY2//SCTR//SLC11A1//TFR2//TNFRSF14//TNFRSF10C//SLC7A7//SLC16A5//CELSR1//TNFSF15//IGSF6//EBP//SLC39A14//OPN3//IL17RB//C8G//ITGAX//SLC2A1//FGA//FGB//FGG//JUP//SLC27A5//STX4//RAB17//CDHR2//CDH2//NCF2//ANXA3//APOB//CDH3//CRP//DIO1//DSG3//GRB7//GRB14//PTPRR//WNT5A//IL1R2//FZD5//ARHGEF5//SCEL//TRAF4//HEPH//PRSS21//RIPK3//MGLL//SULF1//CNNM4//F11R//PARD6A//LSR//TREM2//STEAP3//PLXNA3//CD177//S100A14//RHBDF2//PVRL4//SIGLEC10//ABCC10//OSBPL7//CLDN23//GPR115//ANO9 |
| GO:0016328 | lateral plasma membrane | Cellular component | 3 | 33 | 233 | 17068 | 6.65938353492002 | 0.0101330780080772 | 0.202661560161544 | 1.99425861398066 | CLDN4//JUP//TACSTD2 |
| GO:0044459 | plasma membrane part | Cellular component | 38 | 1904 | 233 | 17068 | 1.46198651134273 | 0.0105522892392841 | 0.202840265451998 | 1.97665331324415 | EFNA1//ADAM8//AQP9//CDH4//CEACAM5//CLDN4//EFNA3//TACSTD2//MUC4//OLR1//P2RY2//SCTR//SLC11A1//TFR2//TNFRSF14//TNFRSF10C//SLC7A7//SLC16A5//CELSR1//TNFSF15//IGSF6//EBP//SLC39A14//OPN3//IL17RB//C8G//ITGAX//SLC2A1//FGA//FGB//FGG//JUP//SLC27A5//STX4//RAB17//CDHR2//CDH2//NCF2 |
| GO:0045178 | basal part of cell | Cellular component | 3 | 34 | 233 | 17068 | 6.46351931330472 | 0.0110035765449537 | 0.202840265451998 | 1.95846613108938 | CLDN4//TACSTD2//SLC27A5 |
| GO:0005916 | fascia adherens | Cellular component | 2 | 12 | 233 | 17068 | 12.20886981402 | 0.0111911870594206 | 0.202840265451998 | 1.95112384501646 | CDH2//JUP |
| GO:0034362 | low-density lipoprotein particle | Cellular component | 2 | 13 | 233 | 17068 | 11.2697259821723 | 0.0131079186029425 | 0.23038159968808 | 1.88246626414675 | APOB//LSR |
| GO:0044425 | membrane part | Cellular component | 101 | 6172 | 233 | 17068 | 1.19873219496049 | 0.0135700393190602 | 0.231122019595601 | 1.8674188939743 | ALOX5AP//AQP9//BIK//CDH2//CDH3//CYB561//DIO1//DSG3//PTPRR//SLC2A1//SPG7//STX4//IL1R2//FZD5//SIGLEC5//SYNGR2//CELSR1//HEPH//TNFSF15//MFSD10//SLC39A14//OPN3//ABCA12//CNNM4//F11R//LSR//TREM2//UGT1A9//CDHR2//ELOVL2//HRASLS2//STEAP3//PLXNA3//SLAMF8//SLC45A4//SLC17A9//FAM173A//MBOAT7//RHBDF2//HSD3B7//PNPLA3//PVRL4//TMEM164//SIGLEC10//ABCC10//SIGLEC12//MARVELD3//PIK3IP1//GOLT1A//LRRC15//TMEM139//CLDN23//FAAH2//B3GNT6//GPR115//CYB561D1//ANO9//MOGAT3//TMEM81//FAM26F//SLC35E2B//EFNA1//ADAM8//CDH4//CEACAM5//CLDN4//EFNA3//TACSTD2//MUC4//OLR1//P2RY2//SCTR//SLC11A1//TFR2//TNFRSF14//TNFRSF10C//SLC7A7//SLC16A5//IGSF6//EBP//IL17RB//C8G//ITGAX//APOB//PCYT2//SLC27A5//MGLL//CYP2S1//ERO1L//AP1S1//FGA//FGB//FGG//JUP//PRSS21//CD177//RAB17//COX6B2//LFNG//NCF2//SULF1 |
| GO:0045335 | phagocytic vesicle | Cellular component | 4 | 68 | 233 | 17068 | 4.30901287553648 | 0.0139470184238725 | 0.231122019595601 | 1.85551862539818 | ANXA3//SLC11A1//ADAM8//NCF2 |
| GO:0031225 | anchored to membrane | Cellular component | 6 | 146 | 233 | 17068 | 3.01040625551179 | 0.0151388524038315 | 0.243903733172841 | 1.81990704515122 | EFNA1//CEACAM5//EFNA3//TNFRSF10C//PRSS21//CD177 |
| GO:0016020 | membrane | Cellular component | 124 | 7856 | 233 | 17068 | 1.15623716161289 | 0.0158604336870886 | 0.248166948897758 | 1.79968494152445 | ADAM8//ANXA3//APOB//AQP9//CDH2//CDH3//CDH4//CLDN4//CRP//DIO1//DSG3//FGA//FGB//FGG//GRB7//GRB14//ITGAX//JUP//OLR1//P2RY2//PTPRR//SCTR//SLC2A1//SLC11A1//STX4//WNT5A//IL1R2//FZD5//ARHGEF5//TNFRSF14//SCEL//SLC7A7//TRAF4//HEPH//TNFSF15//PRSS21//RIPK3//MGLL//SULF1//SLC39A14//CNNM4//F11R//PARD6A//LSR//TREM2//STEAP3//PLXNA3//CD177//S100A14//RHBDF2//PVRL4//SIGLEC10//ABCC10//OSBPL7//CLDN23//GPR115//ANO9//AP1S1//GOLT1A//B3GNT6//ALOX5AP//BIK//CYB561//SPG7//SIGLEC5//SYNGR2//CELSR1//MFSD10//OPN3//ABCA12//UGT1A9//CDHR2//ELOVL2//HRASLS2//SLAMF8//SLC45A4//SLC17A9//FAM173A//MBOAT7//HSD3B7//PNPLA3//TMEM164//SIGLEC12//MARVELD3//PIK3IP1//LRRC15//TMEM139//FAAH2//CYB561D1//MOGAT3//TMEM81//FAM26F//SLC35E2B//EFNA1//CEACAM5//EFNA3//TACSTD2//MUC4//TFR2//TNFRSF10C//SLC16A5//IGSF6//EBP//IL17RB//C8G//MYO19//PCYT2//SLC27A5//CYP2S1//ERO1L//RAB17//COX6B2//LFNG//N4BP3//SULT1E1//NCF2//EN2//GRK6//IGHG1//IGHG3//IGHG4//DNAJC5B//LRG1//RFFL |
| GO:0071944 | cell periphery | Cellular component | 75 | 4400 | 233 | 17068 | 1.2486344127975 | 0.0162592138933014 | 0.248166948897758 | 1.78890045567026 | ADAM8//ANXA3//APOB//AQP9//CDH2//CDH3//CDH4//CLDN4//CRP//DIO1//DSG3//FGA//FGB//FGG//GRB7//GRB14//ITGAX//JUP//OLR1//P2RY2//PTPRR//SCTR//SLC2A1//SLC11A1//STX4//WNT5A//IL1R2//FZD5//ARHGEF5//TNFRSF14//SCEL//SLC7A7//TRAF4//HEPH//TNFSF15//PRSS21//RIPK3//MGLL//SULF1//SLC39A14//CNNM4//F11R//PARD6A//LSR//TREM2//STEAP3//PLXNA3//CD177//S100A14//RHBDF2//PVRL4//SIGLEC10//ABCC10//OSBPL7//CLDN23//GPR115//ANO9//EFNA1//CEACAM5//EFNA3//TACSTD2//MUC4//TFR2//TNFRSF10C//SLC16A5//CELSR1//IGSF6//EBP//OPN3//IL17RB//C8G//SLC27A5//RAB17//CDHR2//NCF2 |
| GO:0009986 | cell surface | Cellular component | 13 | 488 | 233 | 17068 | 1.95141771617533 | 0.0168710973401588 | 0.250903498904926 | 1.77285666884675 | FGA//FGB//FGG//ITGAX//TNFRSF14//ADAM8//MIF//PTPRR//STX4//WNT5A//FZD5//SULF1//IL17RB |
| GO:0005913 | cell-cell adherens junction | Cellular component | 3 | 43 | 233 | 17068 | 5.11068968958978 | 0.0207542749931973 | 0.300936987401361 | 1.68289243318179 | JUP//CDH2//CDH3 |
| GO:0005578 | proteinaceous extracellular matrix | Cellular component | 10 | 362 | 233 | 17068 | 2.02356958243426 | 0.0273437380926452 | 0.386813855944737 | 1.56314211441941 | ALB//LAMA3//CHI3L1//MMP12//MUC4//TNFRSF11B//PI3//WNT5A//SPON2//MMP28 |
| GO:0016235 | aggresome | Cellular component | 2 | 21 | 233 | 17068 | 6.97649703658287 | 0.0328604480129769 | 0.453787139226824 | 1.48332651977383 | HSPA1B//UBD |
| GO:0031224 | intrinsic to membrane | Cellular component | 88 | 5455 | 233 | 17068 | 1.18172012132036 | 0.0339288193738632 | 0.457644540391643 | 1.46943125230116 | ALOX5AP//AQP9//BIK//CDH2//CDH3//CYB561//DIO1//DSG3//PTPRR//SLC2A1//SPG7//STX4//IL1R2//FZD5//SIGLEC5//SYNGR2//CELSR1//HEPH//TNFSF15//MFSD10//SLC39A14//OPN3//ABCA12//CNNM4//F11R//LSR//TREM2//UGT1A9//CDHR2//ELOVL2//HRASLS2//STEAP3//PLXNA3//SLAMF8//SLC45A4//SLC17A9//FAM173A//MBOAT7//RHBDF2//HSD3B7//PNPLA3//PVRL4//TMEM164//SIGLEC10//ABCC10//SIGLEC12//MARVELD3//PIK3IP1//GOLT1A//LRRC15//TMEM139//CLDN23//FAAH2//B3GNT6//GPR115//CYB561D1//ANO9//MOGAT3//TMEM81//FAM26F//SLC35E2B//EFNA1//ADAM8//CDH4//CEACAM5//CLDN4//EFNA3//TACSTD2//MUC4//OLR1//P2RY2//SCTR//SLC11A1//TFR2//TNFRSF14//TNFRSF10C//SLC7A7//SLC16A5//IGSF6//EBP//IL17RB//C8G//ITGAX//PRSS21//CD177//LFNG//SLC27A5//NCF2 |
| GO:0030057 | desmosome | Cellular component | 2 | 22 | 233 | 17068 | 6.65938353492002 | 0.035827222742306 | 0.472267936148579 | 1.44578685620562 | DSG3//JUP |
| GO:0016021 | integral to membrane | Cellular component | 86 | 5337 | 233 | 17068 | 1.18039663182206 | 0.0373872068378521 | 0.481879554798983 | 1.42727697935554 | ADAM8//AQP9//CDH4//CEACAM5//CLDN4//EFNA1//EFNA3//TACSTD2//MUC4//OLR1//P2RY2//SCTR//SLC11A1//TFR2//TNFRSF14//TNFRSF10C//SLC7A7//SLC16A5//CELSR1//TNFSF15//IGSF6//EBP//SLC39A14//OPN3//IL17RB//C8G//ITGAX//LFNG//SLC27A5//NCF2//ALOX5AP//BIK//CDH2//CDH3//CYB561//DIO1//DSG3//PTPRR//SLC2A1//SPG7//STX4//IL1R2//FZD5//SIGLEC5//SYNGR2//HEPH//MFSD10//ABCA12//CNNM4//F11R//LSR//TREM2//UGT1A9//CDHR2//ELOVL2//HRASLS2//STEAP3//PLXNA3//SLAMF8//SLC45A4//SLC17A9//FAM173A//MBOAT7//RHBDF2//HSD3B7//PNPLA3//PVRL4//TMEM164//SIGLEC10//ABCC10//SIGLEC12//MARVELD3//PIK3IP1//GOLT1A//LRRC15//TMEM139//CLDN23//FAAH2//B3GNT6//GPR115//CYB561D1//ANO9//MOGAT3//TMEM81//FAM26F//SLC35E2B |
| GO:0034364 | high-density lipoprotein particle | Cellular component | 2 | 25 | 233 | 17068 | 5.86025751072961 | 0.0453096130878542 | 0.571295121542509 | 1.34380964637361 | HPR//APOC1 |
