# Supplementary material for: Downregulation of miR-181b-5p Inhibits the Viability, Migration, and Glycolysis of Gallbladder Cancer by Upregulating PDHX Under Hypoxia
Source: Front Oncol. 2021 Aug 16;11:683725. doi: 10.3389/fonc.2021.683725 (PMC8415503; doi:10.3389/fonc.2021.683725)
Supplement: Supplementary file 3 [file DataSheet_1.zip › RNA seq raw data/HuGene 2.0 ST Data/GO Analysis/A vs B_up/BP_result(Human).html]

| GO.ID | Term | Ontology | Count | Pop.Hits | List.Total | Pop.Total | Fold.Enrichment | Pvalue | FDR | Enrichment.Score | GENES |
| --- | --- | --- | --- | --- | --- | --- | --- | --- | --- | --- | --- |
| GO:0031497 | chromatin assembly | Biological process | 12 | 120 | 208 | 14747 | 7.08990384615385 | 1.25264325399619e-07 | 0.000597385567830783 | 6.90217259610945 | HIST1H1B//HIST1H2BB//HIST1H2AK//HIST1H2AC//HIST1H2BC//HIST1H2BO//HIST2H2BF//HIST2H4B//HIST2H3D//HIST2H2AA4//BAHD1//CDKN2A |
| GO:0006323 | DNA packaging | Biological process | 13 | 154 | 208 | 14747 | 5.98498376623377 | 2.74264510220476e-07 | 0.000650298712379913 | 6.5618303863896 | HIST1H1B//HIST1H2BB//HIST1H2AK//HIST1H2AC//HIST1H2BC//HIST1H2BO//HIST2H2BF//HIST2H4B//HIST2H3D//HIST2H2AA4//PHF13//BAHD1//CDKN2A |
| GO:0071103 | DNA conformation change | Biological process | 14 | 190 | 208 | 14747 | 5.22413967611336 | 5.04755845411404e-07 | 0.000650298712379913 | 6.29691864293367 | HIST1H1B//HIST1H2BB//HIST1H2AK//HIST1H2AC//HIST1H2BC//HIST1H2BO//HIST2H2BF//HIST2H4B//HIST2H3D//HIST2H2AA4//MCM4//PHF13//BAHD1//CDKN2A |
| GO:0009611 | response to wounding | Biological process | 37 | 1096 | 208 | 14747 | 2.39348943711398 | 5.45438215458094e-07 | 0.000650298712379913 | 6.26325443701935 | LBP//ADAM8//ALOX5AP//CEBPA//CRP//HP//REG3A//ALB//APOB//FGA//FGB//FGG//GRB7//GRB14//ITGAX//OLR1//STX4//SLC7A7//MGLL//F11R//CD177//ANXA8//HIST2H3D//CHI3L1//MECOM//MIF//CCL24//CXCL6//SLC11A1//CCL3L3//BLOC1S3//MMP12//WNT5A//CELSR1//IGFBP1//MAPK13//DUSP10 |
| GO:0006333 | chromatin assembly or disassembly | Biological process | 12 | 141 | 208 | 14747 | 6.03396072013093 | 7.30627813246237e-07 | 0.000696872808274261 | 6.13630379926146 | HIST1H1B//HIST1H2BB//HIST1H2AK//HIST1H2AC//HIST1H2BC//HIST1H2BO//HIST2H2BF//HIST2H4B//HIST2H3D//HIST2H2AA4//BAHD1//CDKN2A |
| GO:0006334 | nucleosome assembly | Biological process | 10 | 108 | 208 | 14747 | 6.56472578347578 | 2.92174485607654e-06 | 0.002052046085514 | 5.53435771189115 | HIST2H4B//HIST1H1B//HIST1H2BB//HIST1H2AK//HIST1H2AC//HIST1H2BC//HIST1H2BO//HIST2H2BF//HIST2H3D//HIST2H2AA4 |
| GO:0006952 | defense response | Biological process | 36 | 1129 | 208 | 14747 | 2.26073107583294 | 3.01201983614971e-06 | 0.002052046085514 | 5.52114217234451 | LBP//ADAM8//ALOX5AP//CEBPA//CRP//HP//REG3A//ANXA3//SLC11A1//HIST1H2BC//S100A14//CHI3L1//MECOM//MIF//OLR1//CCL24//CXCL6//MGLL//F11R//CCL3L3//C8G//NCF2//UBD//IGHG1//IGHG3//IGHG4//SPON2//RIPK3//AP1S1//MAPK13//WNT5A//IRF5//ITGAX//DUSP10//IRF6//IL17RB |
| GO:0002376 | immune system process | Biological process | 49 | 1829 | 208 | 14747 | 1.89942749295538 | 5.57406858799792e-06 | 0.00332284163702026 | 5.25382769166761 | MECOM//LBP//SLC11A1//ARHGEF5//RAB17//NCF2//ADAM8//WNT5A//DUSP10//MIF//IGHG1//IGHG3//IGHG4//AQP9//SERPINB4//CCL24//CXCL6//CXCL5//IL1R2//TNFRSF14//TNFSF15//IGSF6//CCL3L3//C8G//CRP//TREM2//AP1S1//EBP//CEBPA//CDKN2A//BLOC1S3//FZD5//S100A14//UBD//ANXA3//SPON2//RIPK3//HSPA1B//HIST2H4B//APOB//GRB7//GRB14//ITGAX//OLR1//SLC7A7//F11R//CD177//IRF5//IRF6 |
| GO:0022607 | cellular component assembly | Biological process | 43 | 1561 | 208 | 14747 | 1.95301643423841 | 1.23065041618659e-05 | 0.00639292412782814 | 4.90986529709527 | JUP//HIST1H1B//HIST1H2BB//HIST1H2AK//HIST1H2AC//HIST1H2BC//HIST1H2BO//HIST2H2BF//HIST2H4B//HIST2H3D//HIST2H2AA4//SLC7A7//CCL24//BAHD1//LAMA3//BIK//GRB7//CDH2//CDH3//CDH4//F11R//PARD6A//PVRL4//CLDN23//APOB//APOC1//CDKN2A//RIPK3//KCTD13//RAB17//CRP//FGA//FGB//FGG//PCBD2//TACSTD2//VILL//WNT5A//ALOX5AP//MIF//UBD//ARHGEF5//HSPA1B |
| GO:0034728 | nucleosome organization | Biological process | 10 | 128 | 208 | 14747 | 5.53898737980769 | 1.34051669696543e-05 | 0.00639292412782814 | 4.87272777224075 | HIST1H1B//HIST1H2BB//HIST1H2AK//HIST1H2AC//HIST1H2BC//HIST1H2BO//HIST2H2BF//HIST2H4B//HIST2H3D//HIST2H2AA4 |
| GO:0050900 | leukocyte migration | Biological process | 14 | 258 | 208 | 14747 | 3.84723464519976 | 1.80058189074877e-05 | 0.00724612443107425 | 4.7445871220619 | LBP//ARHGEF5//ADAM8//WNT5A//CCL24//S100A14//APOB//GRB7//GRB14//ITGAX//OLR1//SLC7A7//F11R//CD177 |
| GO:0042060 | wound healing | Biological process | 23 | 612 | 208 | 14747 | 2.66450634741076 | 1.82330662975238e-05 | 0.00724612443107425 | 4.73914028888136 | ALB//APOB//FGA//FGB//FGG//GRB7//GRB14//ITGAX//OLR1//STX4//SLC7A7//MGLL//F11R//CD177//ANXA8//HIST2H3D//BLOC1S3//MMP12//IGFBP1//CRP//SLC11A1//WNT5A//CELSR1 |
| GO:0006954 | inflammatory response | Biological process | 20 | 493 | 208 | 14747 | 2.8762287408332 | 2.25530071213844e-05 | 0.00826992795470973 | 4.64679554295883 | LBP//ADAM8//ALOX5AP//CEBPA//CRP//HP//REG3A//MGLL//MAPK13//CCL24//WNT5A//DUSP10//CHI3L1//MECOM//MIF//OLR1//CXCL6//SLC11A1//F11R//CCL3L3 |
| GO:0065004 | protein-DNA complex assembly | Biological process | 10 | 137 | 208 | 14747 | 5.17511229646266 | 2.42774148387369e-05 | 0.00826992795470973 | 4.61479756063819 | HIST1H1B//HIST1H2BB//HIST1H2AK//HIST1H2AC//HIST1H2BC//HIST1H2BO//HIST2H2BF//HIST2H4B//HIST2H3D//HIST2H2AA4 |
| GO:0006950 | response to stress | Biological process | 66 | 2953 | 208 | 14747 | 1.58460431373571 | 3.98766206342255e-05 | 0.0126781069203081 | 4.39928165332163 | SWSAP1//CRP//LBP//ADAM8//ALOX5AP//HSPA1B//HP//IL17RB//CEBPA//REG3A//ANXA3//SLC11A1//HIST1H2BC//S100A14//ALB//APOB//FGA//FGB//FGG//GRB7//GRB14//ITGAX//OLR1//STX4//SLC7A7//MGLL//F11R//CD177//ANXA8//HIST2H3D//CHI3L1//MECOM//MIF//CCL24//CXCL6//CCL3L3//C8G//NCF2//AQP9//MAPK13//SLC2A1//KIAA0101//ATAD5//IGFBP1//DUSP10//WNT5A//BLOC1S3//ERO1L//YOD1//UBD//PYCR1//MMP12//CELSR1//PFKFB1//FZD5//CDKN2A//IGHG1//IGHG3//IGHG4//SPON2//RIPK3//TRAF4//AP1S1//IRF5//IRF6//CGREF1 |
| GO:0044085 | cellular component biogenesis | Biological process | 44 | 1703 | 208 | 14747 | 1.83180134604092 | 4.62059905522086e-05 | 0.0131727998007675 | 4.33530171502593 | PES1//JUP//HIST1H1B//HIST1H2BB//HIST1H2AK//HIST1H2AC//HIST1H2BC//HIST1H2BO//HIST2H2BF//HIST2H4B//HIST2H3D//HIST2H2AA4//CDKN2A//SLC7A7//CCL24//BAHD1//LAMA3//BIK//GRB7//CDH2//CDH3//CDH4//F11R//PARD6A//PVRL4//CLDN23//APOB//APOC1//RIPK3//KCTD13//RAB17//CRP//FGA//FGB//FGG//PCBD2//TACSTD2//VILL//WNT5A//ALOX5AP//MIF//UBD//ARHGEF5//HSPA1B |
| GO:0006879 | cellular iron ion homeostasis | Biological process | 7 | 68 | 208 | 14747 | 7.29843042986425 | 4.69569294638387e-05 | 0.0131727998007675 | 4.32830030955665 | CP//GDF2//HP//SLC11A1//TFR2//HEPH//STEAP3 |
| GO:0071824 | protein-DNA complex subunit organization | Biological process | 10 | 157 | 208 | 14747 | 4.51586232239099 | 7.76380795381676e-05 | 0.0205697778509734 | 4.10992521589434 | HIST1H1B//HIST1H2BB//HIST1H2AK//HIST1H2AC//HIST1H2BC//HIST1H2BO//HIST2H2BF//HIST2H4B//HIST2H3D//HIST2H2AA4 |
| GO:0006955 | immune response | Biological process | 31 | 1087 | 208 | 14747 | 2.02195969853514 | 0.000132052015071361 | 0.0331450557829116 | 3.87925496721211 | LBP//SLC11A1//RAB17//WNT5A//DUSP10//IGHG1//IGHG3//IGHG4//C8G//CRP//TREM2//S100A14//UBD//ADAM8//ANXA3//MIF//NCF2//SPON2//RIPK3//IRF5//IRF6//AQP9//SERPINB4//CCL24//CXCL6//CXCL5//IL1R2//TNFRSF14//TNFSF15//IGSF6//CCL3L3 |
| GO:0001819 | positive regulation of cytokine production | Biological process | 11 | 203 | 208 | 14747 | 3.84181981811292 | 0.000146110053718351 | 0.0348399423091408 | 3.8353198995744 | SULF1//RIPK3//LBP//IRF5//SLC11A1//WNT5A//FZD5//MAPK13//GDF2//MIF//ADAM8 |
| GO:0016477 | cell migration | Biological process | 26 | 851 | 208 | 14747 | 2.16612808460635 | 0.000166102014953795 | 0.0377209766340309 | 3.77962509916807 | WNT5A//CELSR1//LBP//ARHGEF5//ADAM8//ANXA3//GDF2//TACSTD2//IGFBP3//LAMA3//GRB7//CCL24//SULF1//MMP12//EFNA1//APOB//GRB14//ITGAX//OLR1//SLC7A7//F11R//CD177//S100A14//CDH2//JUP//KCTD13 |
| GO:0040011 | locomotion | Biological process | 34 | 1266 | 208 | 14747 | 1.90408160165269 | 0.00019161077242331 | 0.0415359897130348 | 3.71758007838646 | WNT5A//CELSR1//LBP//ARHGEF5//ADAM8//CCL24//CXCL6//CXCL5//CCL3L3//CDH4//SPON2//TREM2//PLXNA3//ANXA3//GDF2//TACSTD2//IGFBP3//CDH2//EFNA1//JUP//KCTD13//APOB//LAMA3//GRB7//SULF1//MMP12//GRB14//ITGAX//OLR1//SLC7A7//F11R//CD177//MIF//S100A14 |
| GO:0055072 | iron ion homeostasis | Biological process | 7 | 88 | 208 | 14747 | 5.63969624125874 | 0.000242477339682156 | 0.0502771492584436 | 3.61532884143628 | CP//GDF2//HP//SLC11A1//TFR2//HEPH//STEAP3 |
| GO:0045730 | respiratory burst | Biological process | 4 | 23 | 208 | 14747 | 12.3302675585284 | 0.000275867397436357 | 0.0548171507655828 | 3.55929962228315 | LBP//DUSP10//NCF2//SLC11A1 |
| GO:0009617 | response to bacterium | Biological process | 14 | 344 | 208 | 14747 | 2.88542598389982 | 0.000383016452270219 | 0.073064218435067 | 3.4167825707397 | ANXA3//HP//SLC11A1//HIST1H2BC//S100A14//LBP//MIF//IRF5//APOB//NCF2//DUSP10//FZD5//WNT5A//MECOM |
| GO:0006953 | acute-phase response | Biological process | 5 | 45 | 208 | 14747 | 7.87767094017094 | 0.000410837571408314 | 0.0753570914633173 | 3.38632984669798 | CEBPA//CRP//HP//LBP//REG3A |
| GO:0050896 | response to stimulus | Biological process | 118 | 6642 | 208 | 14747 | 1.25957340235796 | 0.000429133927683482 | 0.0757977667082417 | 3.36740714842878 | MAPK13//EFNA1//WNT5A//DUSP10//SWSAP1//CRP//LBP//SLC11A1//ARHGEF5//RAB17//ADAM8//ALOX5AP//JUP//HSPA1B//HP//IL17RB//CEBPA//REG3A//ANXA3//HIST1H2BC//S100A14//APOB//AP1S1//SERPINB3//ALB//FGA//FGB//FGG//GRB7//GRB14//ITGAX//OLR1//STX4//SLC7A7//MGLL//F11R//CD177//ANXA8//HIST2H3D//IGHG1//IGHG3//IGHG4//CCNE1//GRK6//FRAT2//IGFBP1//TNFRSF11B//CCL24//CXCL6//CXCL5//TNFRSF10C//TRAF4//TNFSF15//RIPK3//ARRDC2//CBLC//SULT1E1//SULT2A1//OPLAH//SULT1B1//CYP2S1//UGT1A9//EBP//CCL3L3//CHI3L1//CGREF1//MECOM//MIF//AQP9//SERPINB4//IL1R2//TNFRSF14//IGSF6//C8G//TREM2//NCF2//SLC2A1//KIAA0101//ATAD5//TACSTD2//PARD6A//SCTR//CELSR1//OPN3//GPR115//P2RY2//LFNG//NOTCH2NL//PFKFB1//CDKN2A//CDH4//SPON2//PLXNA3//ERO1L//AK4//BLOC1S3//SNRPN//IGFBP3//UBD//GDF2//PIK3IP1//MFSD10//IRF5//IRF6//HIST2H4B//SULF1//YOD1//CLDN4//PYCR1//KCTD13//MMP12//CDH2//FZD5//EFNA3//OSBPL7//BIK//CDH3//CNNM4 |
| GO:0050729 | positive regulation of inflammatory response | Biological process | 6 | 70 | 208 | 14747 | 6.07706043956044 | 0.000453265037042593 | 0.0771708819486032 | 3.34364777929799 | ADAM8//ALOX5AP//LBP//MAPK13//CCL24//WNT5A |
| GO:0007155 | cell adhesion | Biological process | 27 | 963 | 208 | 14747 | 1.98782350826743 | 0.000485831376927333 | 0.0771708819486032 | 3.31351444015593 | CDH2//CDH4//REG3A//CDKN2A//CDH3//DSG3//CELSR1//CDHR2//OLR1//MUC4//ADAM8//JUP//CLDN4//CLDN23//LAMA3//EFNA1//TACSTD2//WNT5A//ITGAX//SIGLEC5//SPON2//OLFM4//CGREF1//F11R//PVRL4//SIGLEC10//SIGLEC12 |
| GO:0010035 | response to inorganic substance | Biological process | 13 | 312 | 208 | 14747 | 2.9541266025641 | 0.000495281257966263 | 0.0771708819486032 | 3.30514810592797 | APOB//CRP//ALB//AQP9//TNFRSF11B//HP//OLR1//FGA//FGB//FGG//ALOX5AP//WNT5A//CEBPA |
| GO:0022610 | biological adhesion | Biological process | 27 | 965 | 208 | 14747 | 1.98370366679952 | 0.000501635005327469 | 0.0771708819486032 | 3.29961216498016 | CDH2//CDH4//REG3A//CDKN2A//CDH3//ITGAX//LAMA3//SIGLEC5//SPON2//OLFM4//CGREF1//F11R//CDHR2//PVRL4//SIGLEC10//SIGLEC12//DSG3//CELSR1//OLR1//MUC4//ADAM8//JUP//CLDN4//CLDN23//EFNA1//TACSTD2//WNT5A |
| GO:0034330 | cell junction organization | Biological process | 10 | 199 | 208 | 14747 | 3.5627657518361 | 0.000530874886589876 | 0.0777868139292106 | 3.27500781877903 | JUP//LAMA3//CDH2//CDH3//CDH4//F11R//PARD6A//PVRL4//CLDN23//MARVELD3 |
| GO:0042221 | response to chemical stimulus | Biological process | 59 | 2786 | 208 | 14747 | 1.50145128113093 | 0.000538260612217226 | 0.0777868139292106 | 3.26900739897415 | LBP//ARHGEF5//HSPA1B//SULT1E1//SULT2A1//OPLAH//SULT1B1//CYP2S1//UGT1A9//EBP//CCL24//CXCL6//CXCL5//CCL3L3//IGFBP1//F11R//PARD6A//CDH4//WNT5A//SPON2//TREM2//PLXNA3//ALB//TNFRSF11B//AK4//NCF2//BLOC1S3//SNRPN//APOB//AQP9//UBD//CRP//MFSD10//CEBPA//IRF5//IRF6//IL17RB//ERO1L//YOD1//MIF//SLC11A1//DUSP10//S100A14//CLDN4//PFKFB1//TNFRSF14//PYCR1//CHI3L1//SULF1//HP//OLR1//ADAM8//FGA//FGB//FGG//FZD5//ALOX5AP//OSBPL7//JUP |
| GO:0065003 | macromolecular complex assembly | Biological process | 28 | 1023 | 208 | 14747 | 1.94054064215355 | 0.000555633179432309 | 0.0779357244915495 | 3.25521182842376 | HIST1H1B//HIST1H2BB//HIST1H2AK//HIST1H2AC//HIST1H2BC//HIST1H2BO//HIST2H2BF//HIST2H4B//HIST2H3D//HIST2H2AA4//SLC7A7//CCL24//BIK//GRB7//APOB//APOC1//RIPK3//KCTD13//CRP//FGA//FGB//FGG//PCBD2//JUP//VILL//ALOX5AP//MIF//ARHGEF5 |
| GO:0048870 | cell motility | Biological process | 26 | 925 | 208 | 14747 | 1.99283783783784 | 0.000598909185119512 | 0.079338830662082 | 3.22263902634453 | WNT5A//CELSR1//LBP//ARHGEF5//ADAM8//ANXA3//GDF2//TACSTD2//IGFBP3//CDH2//EFNA1//JUP//KCTD13//APOB//LAMA3//GRB7//CCL24//SULF1//MMP12//GRB14//ITGAX//OLR1//SLC7A7//F11R//CD177//S100A14 |
| GO:0051674 | localization of cell | Biological process | 26 | 925 | 208 | 14747 | 1.99283783783784 | 0.000598909185119512 | 0.079338830662082 | 3.22263902634453 | WNT5A//CELSR1//LBP//ARHGEF5//ADAM8//ANXA3//GDF2//TACSTD2//IGFBP3//CDH2//EFNA1//JUP//KCTD13//APOB//LAMA3//GRB7//CCL24//SULF1//MMP12//GRB14//ITGAX//OLR1//SLC7A7//F11R//CD177//S100A14 |
| GO:0045216 | cell-cell junction organization | Biological process | 9 | 170 | 208 | 14747 | 3.75347850678733 | 0.000692269343365969 | 0.0881855653695224 | 3.1597249003715 | JUP//CDH2//CDH3//CDH4//PVRL4//PARD6A//F11R//CLDN23//MARVELD3 |
| GO:0060601 | lateral sprouting from an epithelium | Biological process | 3 | 13 | 208 | 14747 | 16.3613165680473 | 0.000712611624146155 | 0.0881855653695224 | 3.14714709770019 | CELSR1//WNT5A//SULF1 |
| GO:0006958 | complement activation, classical pathway | Biological process | 5 | 51 | 208 | 14747 | 6.95088612368024 | 0.000737700000454859 | 0.0881855653695224 | 3.13212021627384 | C8G//CRP//IGHG1//IGHG3//IGHG4 |
| GO:0007596 | blood coagulation | Biological process | 17 | 500 | 208 | 14747 | 2.41056730769231 | 0.000739656660679576 | 0.0881855653695224 | 3.13096982753187 | ALB//FGA//FGB//FGG//STX4//MGLL//BLOC1S3//APOB//GRB7//GRB14//ITGAX//OLR1//SLC7A7//F11R//CD177//ANXA8//HIST2H3D |
| GO:0050817 | coagulation | Biological process | 17 | 503 | 208 | 14747 | 2.39619016669215 | 0.000790309205773873 | 0.0891921833914237 | 3.10220295923057 | ALB//APOB//FGA//FGB//FGG//GRB7//GRB14//ITGAX//OLR1//STX4//SLC7A7//MGLL//F11R//CD177//ANXA8//HIST2H3D//BLOC1S3 |
| GO:0050878 | regulation of body fluid levels | Biological process | 19 | 596 | 208 | 14747 | 2.2602042462571 | 0.000790956761991236 | 0.0891921833914237 | 3.10184725675752 | ALB//APOB//FGA//FGB//FGG//GRB7//GRB14//ITGAX//OLR1//STX4//SLC7A7//MGLL//F11R//CD177//ANXA8//HIST2H3D//BLOC1S3//ABCA12//P2RY2 |
| GO:0007599 | hemostasis | Biological process | 17 | 504 | 208 | 14747 | 2.39143582112332 | 0.000807840697282923 | 0.0891921833914237 | 3.09267427178971 | ALB//APOB//FGA//FGB//FGG//GRB7//GRB14//ITGAX//OLR1//STX4//SLC7A7//MGLL//F11R//CD177//ANXA8//HIST2H3D//BLOC1S3 |
| GO:0034329 | cell junction assembly | Biological process | 9 | 175 | 208 | 14747 | 3.64623626373626 | 0.000851378233797243 | 0.0891921833914237 | 3.06987745712175 | JUP//LAMA3//F11R//PARD6A//CLDN23//CDH2//CDH3//CDH4//PVRL4 |
| GO:0034035 | purine ribonucleoside bisphosphate metabolic process | Biological process | 3 | 14 | 208 | 14747 | 15.1926510989011 | 0.000897564598836957 | 0.0891921833914237 | 3.04693428491251 | SULT1E1//SULT2A1//SULT1B1 |
| GO:0050427 | 3'-phosphoadenosine 5'-phosphosulfate metabolic process | Biological process | 3 | 14 | 208 | 14747 | 15.1926510989011 | 0.000897564598836957 | 0.0891921833914237 | 3.04693428491251 | SULT1E1//SULT2A1//SULT1B1 |
| GO:0051923 | sulfation | Biological process | 3 | 14 | 208 | 14747 | 15.1926510989011 | 0.000897564598836957 | 0.0891921833914237 | 3.04693428491251 | SULT1E1//SULT2A1//SULT1B1 |
| GO:0002237 | response to molecule of bacterial origin | Biological process | 10 | 213 | 208 | 14747 | 3.32859335500181 | 0.000897719606372057 | 0.0891921833914237 | 3.04685928963642 | LBP//MIF//IRF5//APOB//NCF2//SLC11A1//DUSP10//S100A14//FZD5//WNT5A |
| GO:0002526 | acute inflammatory response | Biological process | 7 | 110 | 208 | 14747 | 4.51175699300699 | 0.0009368485129649 | 0.0911802154761144 | 3.0283306282181 | ADAM8//ALOX5AP//CEBPA//CRP//HP//LBP//REG3A |
| GO:0010038 | response to metal ion | Biological process | 10 | 217 | 208 | 14747 | 3.2672367954626 | 0.00103424502502051 | 0.0986462904864562 | 2.98537655949345 | CRP//ALB//AQP9//TNFRSF11B//FGA//FGB//FGG//ALOX5AP//WNT5A//CEBPA |
| GO:0046503 | glycerolipid catabolic process | Biological process | 4 | 34 | 208 | 14747 | 8.34106334841629 | 0.00127989086727583 | 0.119682344039969 | 2.89282705985252 | APOC1//APOB//MGLL//PNPLA3 |
| GO:0051592 | response to calcium ion | Biological process | 6 | 86 | 208 | 14747 | 4.94644454382826 | 0.0013492287307232 | 0.121693644332156 | 2.86991441943829 | ALOX5AP//CRP//WNT5A//FGA//FGB//FGG |
| GO:2000108 | positive regulation of leukocyte apoptotic process | Biological process | 3 | 16 | 208 | 14747 | 13.2935697115385 | 0.00135243513306862 | 0.121693644332156 | 2.86888355581543 | ADAM8//WNT5A//CDKN2A |
| GO:0000041 | transition metal ion transport | Biological process | 6 | 90 | 208 | 14747 | 4.72660256410256 | 0.00170568970407962 | 0.150637670347328 | 2.76809997203301 | CP//HEPH//SLC11A1//TFR2//STEAP3//SLC39A14 |
| GO:0008202 | steroid metabolic process | Biological process | 11 | 276 | 208 | 14747 | 2.8256863154961 | 0.00189482955521448 | 0.164298948160325 | 2.72242984984314 | FDPS//EBP//SLC27A5//HSD3B7//APOB//CEBPA//ALB//SULT1E1//APOC1//SULT2A1//SULT1B1 |
| GO:0065008 | regulation of biological quality | Biological process | 53 | 2569 | 208 | 14747 | 1.46268938826841 | 0.00207062129099061 | 0.1761709907777 | 2.68389932470515 | HIST2H4B//MECOM//MIF//ALB//APOB//FGA//FGB//FGG//GRB7//GRB14//ITGAX//OLR1//STX4//SLC7A7//MGLL//F11R//CD177//ANXA8//HIST2H3D//DIO1//P2RY2//SLC11A1//CP//GDF2//HP//TFR2//HEPH//STEAP3//SLC39A14//JUP//SLC2A1//SULT1E1//ACSM3//CCL24//CDKN2A//SWSAP1//ABCA12//AQP9//BLOC1S3//SULT1B1//UGT1A9//HSPA1B//CDH4//NCF2//TNFRSF11B//ADAM8//WNT5A//PLXNA3//ERO1L//VILL//RAB17//S100A14//CRP |
| GO:0045087 | innate immune response | Biological process | 17 | 551 | 208 | 14747 | 2.18744764763367 | 0.00210562937184502 | 0.1761709907777 | 2.67661806996334 | C8G//S100A14//SLC11A1//UBD//ADAM8//LBP//DUSP10//IRF5//IRF6//WNT5A//IGHG1//IGHG3//IGHG4//MIF//NCF2//SPON2//RIPK3 |
| GO:0034622 | cellular macromolecular complex assembly | Biological process | 17 | 555 | 208 | 14747 | 2.17168225918226 | 0.00227040608430328 | 0.185044400290277 | 2.64389645804948 | HIST1H1B//HIST1H2BB//HIST1H2AK//HIST1H2AC//HIST1H2BC//HIST1H2BO//HIST2H2BF//HIST2H4B//HIST2H3D//HIST2H2AA4//CCL24//GRB7//CRP//FGA//FGB//FGG//VILL |
| GO:0042116 | macrophage activation | Biological process | 4 | 40 | 208 | 14747 | 7.08990384615385 | 0.00236145755446854 | 0.185044400290277 | 2.62681985615366 | LBP//MIF//WNT5A//SLC11A1 |
| GO:0002455 | humoral immune response mediated by circulating immunoglobulin | Biological process | 5 | 66 | 208 | 14747 | 5.37113927738928 | 0.00236689209849169 | 0.185044400290277 | 2.62582154016884 | C8G//CRP//IGHG1//IGHG3//IGHG4 |
| GO:0006956 | complement activation | Biological process | 5 | 66 | 208 | 14747 | 5.37113927738928 | 0.00236689209849169 | 0.185044400290277 | 2.62582154016884 | C8G//CRP//IGHG1//IGHG3//IGHG4 |
| GO:0044763 | single-organism cellular process | Biological process | 156 | 9709 | 208 | 14747 | 1.13917499227521 | 0.00261415642080473 | 0.199933280413075 | 2.58266842950991 | SLC11A1//PHF13//CDKN2A//MCM4//CDC45//CCNE1//PES1//MAPK13//EFNA1//WNT5A//DUSP10//HIST2H4B//SWSAP1//ADAM8//JUP//IGFBP3//IL17RB//GDF2//CDH2//CDH4//REG3A//CELSR1//SULF1//LBP//ARHGEF5//ALB//FGA//FGB//FGG//MIF//MECOM//KIAA0101//HIST1H1B//HIST1H2BB//HIST1H2AK//HIST1H2AC//HIST1H2BC//HIST1H2BO//HIST2H2BF//HIST2H3D//HIST2H2AA4//KRT7//CCL24//VILL//BIK//DSG3//TNFRSF11B//FZD5//TNFRSF10C//TRAF4//MFSD10//STEAP3//RFFL//CDH3//ITGAX//LAMA3//SIGLEC5//SPON2//OLFM4//CGREF1//F11R//CDHR2//PVRL4//SIGLEC10//SIGLEC12//GRK6//FRAT2//GRB14//IGFBP1//CXCL6//CXCL5//TNFSF15//RIPK3//ARRDC2//CBLC//SYNGR2//SULT1E1//SULT2A1//OPLAH//SULT1B1//CYP2S1//UGT1A9//EBP//P2RY2//CP//HP//TFR2//HEPH//SLC39A14//AP1S1//STX4//LLGL2//SLC17A9//BLOC1S3//ATAD5//CEBPA//SPG7//PARD6A//IRF6//SIRT7//LFNG//NCF1B//OLR1//MUC4//TACSTD2//TNFRSF14//IGSF6//GRB7//SCTR//OPN3//GPR115//NOTCH2NL//PFKFB1//CHI3L1//RAB17//EFNA3//TREM2//PLXNA3//SLC2A1//HSPA1B//C8G//MGLL//ANXA3//APOB//CRP//PIK3IP1//SLC27A5//CLDN4//CLDN23//KCTD13//AK4//IRF5//ABCA12//KRT6A//SCEL//ERO1L//YOD1//SND1//BAHD1//NCF2//S100A14//PYCR1//MMP12//UBD//MARVELD3//APOC1//EN2//RHBDF2//LRG1//SLC7A7//CD177//AQP9//ABCC10//TARS2//ALOX5AP//OSBPL7 |
| GO:0015721 | bile acid and bile salt transport | Biological process | 3 | 20 | 208 | 14747 | 10.6348557692308 | 0.00264118193877621 | 0.199933280413075 | 2.57820168122965 | AQP9//ALB//SLC27A5 |
| GO:0051270 | regulation of cellular component movement | Biological process | 15 | 470 | 208 | 14747 | 2.2627352700491 | 0.00277599719358253 | 0.206855165878048 | 2.55658097727058 | ANXA3//WNT5A//GDF2//TACSTD2//IGFBP3//LAMA3//GRB7//CCL24//SULF1//EFNA1//PLXNA3//S100A14//JUP//LBP//ADAM8 |
| GO:0009306 | protein secretion | Biological process | 8 | 172 | 208 | 14747 | 3.29762969588551 | 0.00306973783859896 | 0.225224303881207 | 2.51289871250699 | WNT5A//BLOC1S3//RHBDF2//ADAM8//MIF//TNFSF15//CHI3L1//STEAP3 |
| GO:0008206 | bile acid metabolic process | Biological process | 4 | 44 | 208 | 14747 | 6.44536713286713 | 0.00335740068054043 | 0.238976773813393 | 2.47399682587629 | SLC27A5//HSD3B7//SULT2A1//ALB |
| GO:0032637 | interleukin-8 production | Biological process | 4 | 44 | 208 | 14747 | 6.44536713286713 | 0.00335740068054043 | 0.238976773813393 | 2.47399682587629 | GDF2//LBP//CHI3L1//WNT5A |
| GO:2000106 | regulation of leukocyte apoptotic process | Biological process | 4 | 45 | 208 | 14747 | 6.30213675213675 | 0.00364487025216308 | 0.255623326949496 | 2.43831792681721 | MIF//ADAM8//WNT5A//CDKN2A |
| GO:0006641 | triglyceride metabolic process | Biological process | 6 | 105 | 208 | 14747 | 4.05137362637363 | 0.00370593247479072 | 0.25613901409097 | 2.43110249815607 | APOB//SLC27A5//ELOVL2//PNPLA3//MOGAT3//MGLL |
| GO:0006928 | cellular component movement | Biological process | 29 | 1221 | 208 | 14747 | 1.68392474642475 | 0.003822328655461 | 0.26040979082705 | 2.41767197371307 | WNT5A//CELSR1//LBP//ARHGEF5//ADAM8//SPG7//BLOC1S3//ANXA3//GDF2//TACSTD2//IGFBP3//CDH2//EFNA1//JUP//KCTD13//APOB//LAMA3//GRB7//CCL24//SULF1//MMP12//PLXNA3//GRB14//ITGAX//OLR1//SLC7A7//F11R//CD177//S100A14 |
| GO:0051707 | response to other organism | Biological process | 17 | 586 | 208 | 14747 | 2.05679804410606 | 0.00395187492737574 | 0.263459082268113 | 2.40319680882381 | ANXA3//HP//SLC11A1//HIST1H2BC//S100A14//APOB//AP1S1//SERPINB3//LBP//MECOM//MIF//IRF5//NCF2//DUSP10//ITGAX//FZD5//WNT5A |
| GO:0043030 | regulation of macrophage activation | Biological process | 3 | 23 | 208 | 14747 | 9.24770066889632 | 0.00397757473753494 | 0.263459082268113 | 2.40038165133089 | LBP//WNT5A//MIF |
| GO:0030334 | regulation of cell migration | Biological process | 13 | 398 | 208 | 14747 | 2.31579773869347 | 0.00431898909379881 | 0.282154232716802 | 2.36461789262047 | ANXA3//WNT5A//GDF2//TACSTD2//IGFBP3//GRB7//CCL24//SULF1//EFNA1//S100A14//LBP//ADAM8//LAMA3 |
| GO:0019433 | triglyceride catabolic process | Biological process | 3 | 24 | 208 | 14747 | 8.86237980769231 | 0.00449902745681629 | 0.289944080291309 | 2.34688135639935 | APOB//MGLL//PNPLA3 |
| GO:0006639 | acylglycerol metabolic process | Biological process | 6 | 110 | 208 | 14747 | 3.86722027972028 | 0.00465542487266505 | 0.296022949569862 | 2.33204067742514 | APOB//SLC27A5//ELOVL2//PNPLA3//MOGAT3//MGLL |
| GO:0006638 | neutral lipid metabolic process | Biological process | 6 | 111 | 208 | 14747 | 3.83238045738046 | 0.00486498245082713 | 0.305277648789402 | 2.31291872199844 | APOB//SLC27A5//ELOVL2//PNPLA3//MOGAT3//MGLL |
| GO:0002532 | production of molecular mediator involved in inflammatory response | Biological process | 3 | 25 | 208 | 14747 | 8.50788461538462 | 0.00505996431468445 | 0.305455314135825 | 2.29585254600413 | ALOX5AP//LBP//DUSP10 |
| GO:0046461 | neutral lipid catabolic process | Biological process | 3 | 25 | 208 | 14747 | 8.50788461538462 | 0.00505996431468445 | 0.305455314135825 | 2.29585254600413 | APOB//MGLL//PNPLA3 |
| GO:0046464 | acylglycerol catabolic process | Biological process | 3 | 25 | 208 | 14747 | 8.50788461538462 | 0.00505996431468445 | 0.305455314135825 | 2.29585254600413 | APOB//MGLL//PNPLA3 |
| GO:0006805 | xenobiotic metabolic process | Biological process | 7 | 149 | 208 | 14747 | 3.33082731027362 | 0.00521709861382313 | 0.30866755034999 | 2.282570954147 | AK4//UGT1A9//SULT1E1//SULT2A1//OPLAH//SULT1B1//CYP2S1 |
| GO:0040012 | regulation of locomotion | Biological process | 14 | 455 | 208 | 14747 | 2.18150887573964 | 0.00524262352240495 | 0.30866755034999 | 2.28045132825091 | ANXA3//WNT5A//GDF2//TACSTD2//IGFBP3//LAMA3//GRB7//CCL24//SULF1//EFNA1//PLXNA3//S100A14//LBP//ADAM8 |
| GO:0071466 | cellular response to xenobiotic stimulus | Biological process | 7 | 150 | 208 | 14747 | 3.30862179487179 | 0.00540873465381573 | 0.310873268185501 | 2.26690432401371 | SULT1E1//SULT2A1//OPLAH//SULT1B1//CYP2S1//UGT1A9//AK4 |
| GO:0016337 | cell-cell adhesion | Biological process | 13 | 409 | 208 | 14747 | 2.25351466992665 | 0.0054104594798483 | 0.310873268185501 | 2.26676585114313 | CDH2//CDH4//REG3A//CDH3//DSG3//CELSR1//CDHR2//OLR1//CLDN4//CLDN23//ADAM8//JUP//WNT5A |
| GO:0072330 | monocarboxylic acid biosynthetic process | Biological process | 8 | 190 | 208 | 14747 | 2.98522267206478 | 0.00558457260455298 | 0.317057461322776 | 2.25301005846364 | MIF//ACSM3//MGLL//SLC27A5//HSD3B7//ALOX5AP//ELOVL2//APOC1 |
| GO:0009410 | response to xenobiotic stimulus | Biological process | 7 | 152 | 208 | 14747 | 3.26508729757085 | 0.00580780200672766 | 0.325851856118638 | 2.23598819755096 | SULT1E1//SULT2A1//OPLAH//SULT1B1//CYP2S1//UGT1A9//AK4 |
| GO:0032103 | positive regulation of response to external stimulus | Biological process | 7 | 153 | 208 | 14747 | 3.24374685771745 | 0.0060154076122352 | 0.333575336078485 | 2.22073493893982 | ADAM8//ALOX5AP//WNT5A//MAPK13//CCL24//LBP//S100A14 |
| GO:0050727 | regulation of inflammatory response | Biological process | 8 | 193 | 208 | 14747 | 2.9388202471104 | 0.00612353102811818 | 0.335668039920639 | 2.21299807723233 | ADAM8//ALOX5AP//MAPK13//CCL24//WNT5A//LBP//DUSP10//MGLL |
| GO:0009607 | response to biotic stimulus | Biological process | 17 | 614 | 208 | 14747 | 1.96300269356051 | 0.00625161084758241 | 0.338794683319551 | 2.20400806392343 | ANXA3//HP//SLC11A1//HIST1H2BC//S100A14//APOB//AP1S1//SERPINB3//LBP//MECOM//MIF//IRF5//NCF2//DUSP10//ITGAX//FZD5//WNT5A |
| GO:0002274 | myeloid leukocyte activation | Biological process | 6 | 118 | 208 | 14747 | 3.60503585397653 | 0.00653113280079909 | 0.349965981202369 | 2.18501148542193 | LBP//SLC11A1//UBD//MIF//WNT5A//ANXA3 |
| GO:0072376 | protein activation cascade | Biological process | 5 | 84 | 208 | 14747 | 4.22018086080586 | 0.00667197403649201 | 0.353540490889227 | 2.17574565239283 | IGHG1//IGHG3//IGHG4//C8G//CRP |
| GO:2000145 | regulation of cell motility | Biological process | 13 | 421 | 208 | 14747 | 2.18928147268409 | 0.00684289163410534 | 0.357762155270314 | 2.16476033755487 | ANXA3//WNT5A//GDF2//TACSTD2//IGFBP3//LAMA3//GRB7//CCL24//SULF1//EFNA1//S100A14//LBP//ADAM8 |
| GO:0045765 | regulation of angiogenesis | Biological process | 7 | 157 | 208 | 14747 | 3.16110362567369 | 0.00690168133463387 | 0.357762155270314 | 2.16104509688531 | GDF2//SULF1//ANXA3//CHI3L1//CCL24//WNT5A//EFNA1 |
| GO:0034332 | adherens junction organization | Biological process | 5 | 86 | 208 | 14747 | 4.12203711985689 | 0.00735901890357929 | 0.361209925962263 | 2.1331800814727 | CDH2//CDH3//CDH4//JUP//PVRL4 |
| GO:0002252 | immune effector process | Biological process | 14 | 475 | 208 | 14747 | 2.08965587044534 | 0.00755769490011352 | 0.361209925962263 | 2.12161064428876 | LBP//SLC11A1//WNT5A//IGHG1//IGHG3//IGHG4//C8G//CRP//ANXA3//AP1S1//IRF5//ITGAX//DUSP10//MIF |
| GO:0006270 | DNA replication initiation | Biological process | 3 | 29 | 208 | 14747 | 7.33438328912467 | 0.00771375080288374 | 0.361209925962263 | 2.11273439537413 | CCNE1//MCM4//CDC45 |
| GO:0007157 | heterophilic cell-cell adhesion | Biological process | 3 | 29 | 208 | 14747 | 7.33438328912467 | 0.00771375080288374 | 0.361209925962263 | 2.11273439537413 | CDH2//CDH4//REG3A |
| GO:0001558 | regulation of cell growth | Biological process | 10 | 288 | 208 | 14747 | 2.46177216880342 | 0.0078408896067531 | 0.361209925962263 | 2.10563466060818 | IGFBP1//CDKN2A//GDF2//HSPA1B//CDHR2//CDH4//WNT5A//PLXNA3//IGFBP3//IL17RB |
| GO:0048584 | positive regulation of response to stimulus | Biological process | 27 | 1173 | 208 | 14747 | 1.63194717686406 | 0.00788027134811918 | 0.361209925962263 | 2.10345882781985 | EFNA1//WNT5A//ADAM8//ALOX5AP//SLC11A1//IGHG1//IGHG3//IGHG4//C8G//CRP//MIF//GDF2//SULF1//S100A14//LBP//UBD//CDH2//IGFBP3//FZD5//CDKN2A//LFNG//ALB//TRAF4//MAPK13//CCL24//CHI3L1//JUP |
| GO:0030595 | leukocyte chemotaxis | Biological process | 6 | 123 | 208 | 14747 | 3.45848968105066 | 0.00795154611084615 | 0.361209925962263 | 2.09954841824616 | LBP//ARHGEF5//WNT5A//CCL24//ADAM8//S100A14 |
| GO:0032496 | response to lipopolysaccharide | Biological process | 8 | 202 | 208 | 14747 | 2.80788271134806 | 0.00797932039498979 | 0.361209925962263 | 2.09803409627765 | LBP//MIF//WNT5A//APOB//NCF2//SLC11A1//DUSP10//S100A14 |
| GO:0043032 | positive regulation of macrophage activation | Biological process | 2 | 10 | 208 | 14747 | 14.1798076923077 | 0.00826962834526905 | 0.361209925962263 | 2.08251400812892 | LBP//WNT5A |
| GO:0046689 | response to mercury ion | Biological process | 2 | 10 | 208 | 14747 | 14.1798076923077 | 0.00826962834526905 | 0.361209925962263 | 2.08251400812892 | ALB//AQP9 |
| GO:0060513 | prostatic bud formation | Biological process | 2 | 10 | 208 | 14747 | 14.1798076923077 | 0.00826962834526905 | 0.361209925962263 | 2.08251400812892 | WNT5A//SULF1 |
| GO:0070841 | inclusion body assembly | Biological process | 2 | 10 | 208 | 14747 | 14.1798076923077 | 0.00826962834526905 | 0.361209925962263 | 2.08251400812892 | UBD//HSPA1B |
| GO:0072677 | eosinophil migration | Biological process | 2 | 10 | 208 | 14747 | 14.1798076923077 | 0.00826962834526905 | 0.361209925962263 | 2.08251400812892 | CCL24//ADAM8 |
| GO:0071887 | leukocyte apoptotic process | Biological process | 4 | 57 | 208 | 14747 | 4.97537112010796 | 0.00847809228764603 | 0.361209925962263 | 2.07170186026163 | MIF//ADAM8//WNT5A//CDKN2A |
| GO:0045766 | positive regulation of angiogenesis | Biological process | 5 | 89 | 208 | 14747 | 3.98309204840104 | 0.00847985860190026 | 0.361209925962263 | 2.07161138936197 | ANXA3//CHI3L1//GDF2//CCL24//WNT5A |
| GO:0033865 | nucleoside bisphosphate metabolic process | Biological process | 3 | 30 | 208 | 14747 | 7.08990384615385 | 0.0084830177621668 | 0.361209925962263 | 2.07144962364988 | SULT1E1//SULT2A1//SULT1B1 |
| GO:0033875 | ribonucleoside bisphosphate metabolic process | Biological process | 3 | 30 | 208 | 14747 | 7.08990384615385 | 0.0084830177621668 | 0.361209925962263 | 2.07144962364988 | SULT1E1//SULT2A1//SULT1B1 |
| GO:0034032 | purine nucleoside bisphosphate metabolic process | Biological process | 3 | 30 | 208 | 14747 | 7.08990384615385 | 0.0084830177621668 | 0.361209925962263 | 2.07144962364988 | SULT1E1//SULT2A1//SULT1B1 |
| GO:0050873 | brown fat cell differentiation | Biological process | 3 | 30 | 208 | 14747 | 7.08990384615385 | 0.0084830177621668 | 0.361209925962263 | 2.07144962364988 | CEBPA//ERO1L//LRG1 |
| GO:0071277 | cellular response to calcium ion | Biological process | 3 | 30 | 208 | 14747 | 7.08990384615385 | 0.0084830177621668 | 0.361209925962263 | 2.07144962364988 | ALOX5AP//CRP//WNT5A |
| GO:0001816 | cytokine production | Biological process | 13 | 434 | 208 | 14747 | 2.12370391705069 | 0.00871937746973997 | 0.365996368020303 | 2.05951452092843 | SLC11A1//WNT5A//SULF1//RIPK3//LBP//IRF5//FZD5//MAPK13//GDF2//MIF//TNFSF15//CHI3L1//ADAM8 |
| GO:0001817 | regulation of cytokine production | Biological process | 12 | 386 | 208 | 14747 | 2.2041151853328 | 0.0087628346125252 | 0.365996368020303 | 2.0573553849912 | SLC11A1//WNT5A//SULF1//RIPK3//LBP//IRF5//FZD5//MAPK13//GDF2//MIF//TNFSF15//ADAM8 |
| GO:0043933 | macromolecular complex subunit organization | Biological process | 29 | 1298 | 208 | 14747 | 1.58403090553514 | 0.00882566205123397 | 0.365996368020303 | 2.05425270640324 | HIST1H1B//HIST1H2BB//HIST1H2AK//HIST1H2AC//HIST1H2BC//HIST1H2BO//HIST2H2BF//HIST2H4B//HIST2H3D//HIST2H2AA4//SLC7A7//CCL24//BIK//GRB7//APOC1//APOB//RIPK3//KCTD13//CRP//FGA//FGB//FGG//PCBD2//JUP//TACSTD2//VILL//ALOX5AP//MIF//ARHGEF5 |
| GO:0006959 | humoral immune response | Biological process | 6 | 126 | 208 | 14747 | 3.37614468864469 | 0.0089036076696474 | 0.366045732556452 | 2.05043398509082 | IGHG1//IGHG3//IGHG4//C8G//CRP//TREM2 |
| GO:0032729 | positive regulation of interferon-gamma production | Biological process | 3 | 31 | 208 | 14747 | 6.86119727047147 | 0.00929575148942458 | 0.373101164602977 | 2.0317154951317 | SLC11A1//WNT5A//FZD5 |
| GO:0070228 | regulation of lymphocyte apoptotic process | Biological process | 3 | 31 | 208 | 14747 | 6.86119727047147 | 0.00929575148942458 | 0.373101164602977 | 2.0317154951317 | MIF//ADAM8//WNT5A |
| GO:0009605 | response to external stimulus | Biological process | 28 | 1247 | 208 | 14747 | 1.59195916353094 | 0.00942880370323623 | 0.373101164602977 | 2.02554340567635 | LBP//ARHGEF5//ADAM8//ALOX5AP//CCL24//CXCL6//CXCL5//CCL3L3//CHI3L1//CDH4//WNT5A//SPON2//TREM2//PLXNA3//ALB//TNFRSF11B//OPN3//CEBPA//BLOC1S3//SLC2A1//PFKFB1//MGLL//MAPK13//MIF//JUP//DUSP10//S100A14//MECOM |
| GO:0043549 | regulation of kinase activity | Biological process | 17 | 643 | 208 | 14747 | 1.874469135064 | 0.00967887803976036 | 0.373101164602977 | 2.01417498250371 | CCNE1//EFNA1//WNT5A//DUSP10//CBLC//CDKN2A//IGFBP1//CHI3L1//TNFSF15//SLC11A1//RIPK3//PFKFB1//ADAM8//MIF//FZD5//PIK3IP1//TRAF4 |
| GO:0016053 | organic acid biosynthetic process | Biological process | 10 | 298 | 208 | 14747 | 2.37916236448116 | 0.00983396877117784 | 0.373101164602977 | 2.00727117519236 | MIF//PYCR1//ACSM3//MGLL//SLC27A5//HSD3B7//ALOX5AP//OPLAH//ELOVL2//APOC1 |
| GO:0046394 | carboxylic acid biosynthetic process | Biological process | 10 | 298 | 208 | 14747 | 2.37916236448116 | 0.00983396877117784 | 0.373101164602977 | 2.00727117519236 | MIF//PYCR1//ACSM3//MGLL//SLC27A5//HSD3B7//ALOX5AP//OPLAH//ELOVL2//APOC1 |
| GO:0003306 | Wnt receptor signaling pathway involved in heart development | Biological process | 2 | 11 | 208 | 14747 | 12.8907342657343 | 0.0100140383873309 | 0.373101164602977 | 1.99939074813044 | JUP//WNT5A |
| GO:0040037 | negative regulation of fibroblast growth factor receptor signaling pathway | Biological process | 2 | 11 | 208 | 14747 | 12.8907342657343 | 0.0100140383873309 | 0.373101164602977 | 1.99939074813044 | WNT5A//SULF1 |
| GO:0048841 | regulation of axon extension involved in axon guidance | Biological process | 2 | 11 | 208 | 14747 | 12.8907342657343 | 0.0100140383873309 | 0.373101164602977 | 1.99939074813044 | WNT5A//PLXNA3 |
| GO:0070230 | positive regulation of lymphocyte apoptotic process | Biological process | 2 | 11 | 208 | 14747 | 12.8907342657343 | 0.0100140383873309 | 0.373101164602977 | 1.99939074813044 | ADAM8//WNT5A |
| GO:0070243 | regulation of thymocyte apoptotic process | Biological process | 2 | 11 | 208 | 14747 | 12.8907342657343 | 0.0100140383873309 | 0.373101164602977 | 1.99939074813044 | ADAM8//WNT5A |
| GO:2000406 | positive regulation of T cell migration | Biological process | 2 | 11 | 208 | 14747 | 12.8907342657343 | 0.0100140383873309 | 0.373101164602977 | 1.99939074813044 | WNT5A//ADAM8 |
| GO:0010951 | negative regulation of endopeptidase activity | Biological process | 6 | 130 | 208 | 14747 | 3.27226331360947 | 0.0102965169329813 | 0.380651854677425 | 1.98730966195587 | ITIH2//PI3//SERPINB3//SERPINB4//SPINK1//SERPINB7 |
| GO:1901342 | regulation of vasculature development | Biological process | 7 | 170 | 208 | 14747 | 2.9193721719457 | 0.0104571694049103 | 0.383617237630902 | 1.98058585640477 | GDF2//SULF1//EFNA1//ANXA3//CHI3L1//CCL24//WNT5A |
| GO:0044699 | single-organism process | Biological process | 167 | 10796 | 208 | 14747 | 1.09671539672813 | 0.0107385629840538 | 0.390932876877501 | 1.96905383128555 | SLC11A1//PHF13//CDKN2A//MCM4//CDC45//CCNE1//PES1//MAPK13//EFNA1//WNT5A//DUSP10//FZD5//HIST2H4B//SWSAP1//ADAM8//JUP//TNFRSF11B//EBP//GDF2//LFNG//IGFBP3//IL17RB//CDH2//CDH4//REG3A//BLOC1S3//APOB//MECOM//PTPRR//CELSR1//SULF1//AK4//CEBPA//LSR//CCL24//LBP//ARHGEF5//RAB17//ALOX5AP//ALB//FGA//FGB//FGG//MIF//KIAA0101//HIST1H1B//HIST1H2BB//HIST1H2AK//HIST1H2AC//HIST1H2BC//HIST1H2BO//HIST2H2BF//HIST2H3D//HIST2H2AA4//GRB7//GRB14//ITGAX//OLR1//STX4//SLC7A7//MGLL//F11R//CD177//ANXA8//IGHG1//IGHG3//IGHG4//KRT7//VILL//BIK//DSG3//TNFRSF10C//TRAF4//MFSD10//STEAP3//RFFL//CDH3//LAMA3//SIGLEC5//SPON2//OLFM4//CGREF1//CDHR2//PVRL4//SIGLEC10//SIGLEC12//GRK6//FRAT2//IGFBP1//CXCL6//CXCL5//TNFSF15//RIPK3//ARRDC2//CBLC//SYNGR2//SULT1E1//SULT2A1//OPLAH//SULT1B1//CYP2S1//UGT1A9//CNNM4//CP//HEPH//TFR2//AQP9//ABCA12//OSBPL7//P2RY2//HP//SLC39A14//AP1S1//LLGL2//SLC17A9//ANXA3//C8G//CRP//ATAD5//SPG7//PARD6A//IRF6//SIRT7//NCF1B//MUC4//TACSTD2//TNFRSF14//IGSF6//SCTR//OPN3//GPR115//NOTCH2NL//PFKFB1//CHI3L1//EFNA3//EN2//PLXNA3//SCEL//TREM2//NCF2//SLC2A1//HSPA1B//ACSM3//KRT6A//CCL3L3//APOC1//PIK3IP1//SLC16A5//SLC27A5//CLDN4//CLDN23//KCTD13//IRF5//MBOAT7//ERO1L//YOD1//SND1//BAHD1//S100A14//PYCR1//MMP12//UBD//MARVELD3//RHBDF2//LRG1//ABCC10//TARS2 |
| GO:0032755 | positive regulation of interleukin-6 production | Biological process | 3 | 33 | 208 | 14747 | 6.44536713286713 | 0.0110534968193906 | 0.396791709707151 | 1.9565003093884 | LBP//MAPK13//WNT5A |
| GO:0015718 | monocarboxylic acid transport | Biological process | 5 | 95 | 208 | 14747 | 3.73152834008097 | 0.0110659042547811 | 0.396791709707151 | 1.95601309173981 | ALB//SLC27A5//AQP9//MIF//SLC16A5 |
| GO:0010466 | negative regulation of peptidase activity | Biological process | 6 | 133 | 208 | 14747 | 3.19845286292655 | 0.0114381297268892 | 0.407077915429363 | 1.94164498215963 | ITIH2//PI3//SERPINB3//SERPINB4//SPINK1//SERPINB7 |
| GO:0006633 | fatty acid biosynthetic process | Biological process | 6 | 134 | 208 | 14747 | 3.17458381171068 | 0.0118377747896926 | 0.408489384767883 | 1.9267299266149 | MIF//ALOX5AP//ELOVL2//APOC1//ACSM3//MGLL |
| GO:0009812 | flavonoid metabolic process | Biological process | 2 | 12 | 208 | 14747 | 11.8165064102564 | 0.011906065104369 | 0.408489384767883 | 1.92423174698241 | UGT1A9//SULT1B1 |
| GO:0050919 | negative chemotaxis | Biological process | 2 | 12 | 208 | 14747 | 11.8165064102564 | 0.011906065104369 | 0.408489384767883 | 1.92423174698241 | WNT5A//PLXNA3 |
| GO:0070242 | thymocyte apoptotic process | Biological process | 2 | 12 | 208 | 14747 | 11.8165064102564 | 0.011906065104369 | 0.408489384767883 | 1.92423174698241 | ADAM8//WNT5A |
| GO:0071425 | hematopoietic stem cell proliferation | Biological process | 2 | 12 | 208 | 14747 | 11.8165064102564 | 0.011906065104369 | 0.408489384767883 | 1.92423174698241 | MECOM//WNT5A |
| GO:0006629 | lipid metabolic process | Biological process | 26 | 1159 | 208 | 14747 | 1.59048748921484 | 0.012399117214586 | 0.422367071402576 | 1.90660923439275 | SLC27A5//MIF//ACSM3//MGLL//APOB//PCYT2//MBOAT7//PNPLA3//SMPDL3B//FDPS//EBP//HSD3B7//SULT1E1//SULT2A1//SULT1B1//CEBPA//ALB//APOC1//HRASLS2//LSR//ALOX5AP//ELOVL2//MOGAT3//UGT1A9//PIK3IP1//ACP6 |
| GO:0043436 | oxoacid metabolic process | Biological process | 23 | 991 | 208 | 14747 | 1.6454872700458 | 0.0127101184222304 | 0.426704139527052 | 1.89585040303517 | SLC27A5//MIF//TARS2//SLC7A7//YOD1//PYCR1//AFMID//DIO1//ACSM3//MGLL//HSD3B7//ALOX5AP//OPLAH//ALB//SLC2A1//SULF1//SULT2A1//AGMAT//ELOVL2//SULT1B1//UGT1A9//APOC1//SULT1E1 |
| GO:0006259 | DNA metabolic process | Biological process | 21 | 880 | 208 | 14747 | 1.69190887237762 | 0.0127907649996553 | 0.426704139527052 | 1.89310348013484 | KRT7//MCM4//CDC45//KIAA0101//KCTD13//HIST2H4B//SWSAP1//HIST1H1B//HIST1H2BB//HIST1H2AK//HIST1H2AC//HIST1H2BC//HIST1H2BO//HIST2H2BF//HIST2H3D//HIST2H2AA4//CCNE1//CDKN2A//PHF13//GDF2//BAHD1 |
| GO:0044283 | small molecule biosynthetic process | Biological process | 13 | 456 | 208 | 14747 | 2.02124451754386 | 0.0127948609671563 | 0.426704139527052 | 1.89296442892685 | MIF//PYCR1//ACSM3//MGLL//FDPS//EBP//SLC27A5//HSD3B7//ALOX5AP//OPLAH//ELOVL2//APOB//APOC1 |
| GO:0051338 | regulation of transferase activity | Biological process | 17 | 664 | 208 | 14747 | 1.81518622567192 | 0.0129925772382478 | 0.427762544106956 | 1.88630469270444 | CCNE1//EFNA1//WNT5A//DUSP10//CBLC//CDKN2A//IGFBP1//CHI3L1//TNFSF15//SLC11A1//RIPK3//PFKFB1//ADAM8//MIF//FZD5//PIK3IP1//TRAF4 |
| GO:0019752 | carboxylic acid metabolic process | Biological process | 21 | 882 | 208 | 14747 | 1.68807234432234 | 0.0130942729428427 | 0.427762544106956 | 1.88291861069254 | SLC27A5//MIF//TARS2//SLC7A7//YOD1//PYCR1//AFMID//DIO1//ACSM3//MGLL//HSD3B7//ALOX5AP//OPLAH//ALB//SLC2A1//SULT2A1//AGMAT//ELOVL2//SULT1B1//UGT1A9//APOC1 |
| GO:0030308 | negative regulation of cell growth | Biological process | 6 | 137 | 208 | 14747 | 3.1050673778776 | 0.0130956870286466 | 0.427762544106956 | 1.88287171259397 | WNT5A//PLXNA3//CDKN2A//GDF2//HSPA1B//CDHR2 |
| GO:0002687 | positive regulation of leukocyte migration | Biological process | 4 | 65 | 208 | 14747 | 4.36301775147929 | 0.0133232165828882 | 0.432234148869346 | 1.87539091213603 | WNT5A//S100A14//LBP//ADAM8 |
| GO:0010800 | positive regulation of peptidyl-threonine phosphorylation | Biological process | 2 | 13 | 208 | 14747 | 10.9075443786982 | 0.0139412384644096 | 0.441672586155232 | 1.8556986441456 | CHI3L1//WNT5A |
| GO:0070207 | protein homotrimerization | Biological process | 2 | 13 | 208 | 14747 | 10.9075443786982 | 0.0139412384644096 | 0.441672586155232 | 1.8556986441456 | ALOX5AP//MIF |
| GO:2000404 | regulation of T cell migration | Biological process | 2 | 13 | 208 | 14747 | 10.9075443786982 | 0.0139412384644096 | 0.441672586155232 | 1.8556986441456 | WNT5A//ADAM8 |
| GO:0007156 | homophilic cell adhesion | Biological process | 6 | 139 | 208 | 14747 | 3.06039014941893 | 0.0139846006520109 | 0.441672586155232 | 1.85434993094453 | CDH2//CDH3//CDH4//DSG3//CELSR1//CDHR2 |
| GO:0008610 | lipid biosynthetic process | Biological process | 15 | 566 | 208 | 14747 | 1.87894978254961 | 0.0144156886180922 | 0.452292230392643 | 1.8411646071159 | MIF//ACSM3//MGLL//PCYT2//FDPS//EBP//SLC27A5//HSD3B7//ALOX5AP//ELOVL2//PNPLA3//MOGAT3//APOB//APOC1//MBOAT7 |
| GO:0003156 | regulation of organ formation | Biological process | 3 | 37 | 208 | 14747 | 5.74857068607069 | 0.0151086609431538 | 0.470935974103925 | 1.82077402479196 | JUP//WNT5A//SULF1 |
| GO:0006082 | organic acid metabolic process | Biological process | 23 | 1008 | 208 | 14747 | 1.61773599664225 | 0.01528805822494 | 0.473433439446356 | 1.81564767192884 | SLC27A5//MIF//TARS2//SLC7A7//YOD1//PYCR1//AFMID//DIO1//ACSM3//MGLL//HSD3B7//ALOX5AP//OPLAH//ALB//SLC2A1//SULF1//SULT2A1//AGMAT//ELOVL2//SULT1B1//UGT1A9//APOC1//SULT1E1 |
| GO:0044711 | single-organism biosynthetic process | Biological process | 13 | 468 | 208 | 14747 | 1.96941773504274 | 0.0155664571393313 | 0.478944736112716 | 1.80781021979667 | MIF//PYCR1//ACSM3//MGLL//FDPS//EBP//SLC27A5//HSD3B7//ALOX5AP//OPLAH//ELOVL2//APOB//APOC1 |
| GO:0071900 | regulation of protein serine/threonine kinase activity | Biological process | 11 | 369 | 208 | 14747 | 2.11352147175318 | 0.0157769918601313 | 0.48231073192927 | 1.80197579853362 | CCNE1//EFNA1//WNT5A//DUSP10//CHI3L1//TNFSF15//ADAM8//MIF//CBLC//FZD5//CDKN2A |
| GO:0042742 | defense response to bacterium | Biological process | 6 | 143 | 208 | 14747 | 2.97478483055406 | 0.0158872247177744 | 0.482587099866663 | 1.79895196151031 | LBP//SLC11A1//ANXA3//HP//HIST1H2BC//S100A14 |
| GO:0030516 | regulation of axon extension | Biological process | 3 | 38 | 208 | 14747 | 5.59729251012146 | 0.0162365787100341 | 0.490077492836409 | 1.78950547779726 | CDH4//WNT5A//PLXNA3 |
| GO:0070887 | cellular response to chemical stimulus | Biological process | 35 | 1723 | 208 | 14747 | 1.4402010134381 | 0.0166220088121546 | 0.498555723428713 | 1.77931649178446 | LBP//ARHGEF5//SULT1E1//SULT2A1//OPLAH//SULT1B1//CYP2S1//UGT1A9//IGFBP1//F11R//PARD6A//WNT5A//AK4//CEBPA//IRF5//IRF6//IL17RB//ERO1L//YOD1//MIF//NCF2//TNFRSF14//PYCR1//SULF1//CCL24//ADAM8//ALOX5AP//CRP//AQP9//APOB//CHI3L1//OSBPL7//S100A14//JUP//HP |
| GO:0051716 | cellular response to stimulus | Biological process | 87 | 5102 | 208 | 14747 | 1.20898007568676 | 0.0173394621001957 | 0.516824342223958 | 1.7609643792093 | MAPK13//EFNA1//WNT5A//DUSP10//SWSAP1//LBP//ARHGEF5//JUP//CCNE1//GRK6//FRAT2//FGA//FGB//FGG//GRB14//IGFBP1//TNFRSF11B//CCL24//CXCL6//CXCL5//TNFRSF10C//TRAF4//TNFSF15//RIPK3//ARRDC2//CBLC//SULT1E1//SULT2A1//OPLAH//SULT1B1//CYP2S1//UGT1A9//KIAA0101//ATAD5//TACSTD2//MIF//TNFRSF14//IGSF6//GRB7//F11R//PARD6A//SCTR//CELSR1//OPN3//GPR115//P2RY2//LFNG//NOTCH2NL//ITGAX//PFKFB1//CHI3L1//RAB17//CDKN2A//ALB//MGLL//IGFBP3//GDF2//PIK3IP1//AK4//CEBPA//IRF5//IRF6//IL17RB//HIST2H4B//SULF1//ERO1L//YOD1//NCF2//S100A14//PYCR1//KCTD13//ADAM8//BLOC1S3//SLC2A1//UBD//CDH2//FZD5//MECOM//EFNA3//ALOX5AP//CRP//AQP9//APOB//OSBPL7//PLXNA3//BIK//HP |
| GO:2000027 | regulation of organ morphogenesis | Biological process | 6 | 147 | 208 | 14747 | 2.89383830455259 | 0.0179620454883472 | 0.517420682190718 | 1.74564420810755 | JUP//TNFRSF11B//WNT5A//SULF1//CELSR1//TACSTD2 |
| GO:0006825 | copper ion transport | Biological process | 2 | 15 | 208 | 14747 | 9.45320512820513 | 0.0184236215632542 | 0.517420682190718 | 1.73462499572763 | CP//HEPH |
| GO:0007350 | blastoderm segmentation | Biological process | 2 | 15 | 208 | 14747 | 9.45320512820513 | 0.0184236215632542 | 0.517420682190718 | 1.73462499572763 | WNT5A//FZD5 |
| GO:0007351 | tripartite regional subdivision | Biological process | 2 | 15 | 208 | 14747 | 9.45320512820513 | 0.0184236215632542 | 0.517420682190718 | 1.73462499572763 | WNT5A//FZD5 |
| GO:0008595 | anterior/posterior axis specification, embryo | Biological process | 2 | 15 | 208 | 14747 | 9.45320512820513 | 0.0184236215632542 | 0.517420682190718 | 1.73462499572763 | WNT5A//FZD5 |
| GO:0090178 | regulation of establishment of planar polarity involved in neural tube closure | Biological process | 2 | 15 | 208 | 14747 | 9.45320512820513 | 0.0184236215632542 | 0.517420682190718 | 1.73462499572763 | WNT5A//CELSR1 |
| GO:0090179 | planar cell polarity pathway involved in neural tube closure | Biological process | 2 | 15 | 208 | 14747 | 9.45320512820513 | 0.0184236215632542 | 0.517420682190718 | 1.73462499572763 | WNT5A//CELSR1 |
| GO:2000403 | positive regulation of lymphocyte migration | Biological process | 2 | 15 | 208 | 14747 | 9.45320512820513 | 0.0184236215632542 | 0.517420682190718 | 1.73462499572763 | WNT5A//ADAM8 |
| GO:0051240 | positive regulation of multicellular organismal process | Biological process | 14 | 531 | 208 | 14747 | 1.86927785021005 | 0.018519912760207 | 0.517420682190718 | 1.73236106343432 | SLC11A1//JUP//SULF1//RIPK3//LBP//IRF5//WNT5A//FZD5//MAPK13//GDF2//ADAM8//ALB//MIF//P2RY2 |
| GO:0070830 | tight junction assembly | Biological process | 3 | 40 | 208 | 14747 | 5.31742788461538 | 0.0186307633436992 | 0.517420682190718 | 1.72976935073098 | F11R//PARD6A//CLDN23 |
| GO:0045926 | negative regulation of growth | Biological process | 7 | 191 | 208 | 14747 | 2.59839407974225 | 0.0187686013793245 | 0.517420682190718 | 1.72656808943902 | CDKN2A//GDF2//HSPA1B//CDHR2//LBP//WNT5A//PLXNA3 |
| GO:0001936 | regulation of endothelial cell proliferation | Biological process | 4 | 72 | 208 | 14747 | 3.93883547008547 | 0.0187699261939598 | 0.517420682190718 | 1.72653743508345 | GDF2//SULF1//CCL24//WNT5A |
| GO:0007043 | cell-cell junction assembly | Biological process | 4 | 72 | 208 | 14747 | 3.93883547008547 | 0.0187699261939598 | 0.517420682190718 | 1.72653743508345 | JUP//F11R//PARD6A//CLDN23 |
| GO:0040013 | negative regulation of locomotion | Biological process | 6 | 149 | 208 | 14747 | 2.85499483737739 | 0.019066122929724 | 0.522565173861228 | 1.71973761117075 | GDF2//TACSTD2//IGFBP3//SULF1//WNT5A//PLXNA3 |
| GO:0060326 | cell chemotaxis | Biological process | 6 | 150 | 208 | 14747 | 2.83596153846154 | 0.0196351807745535 | 0.527226543555867 | 1.70696509597087 | LBP//ARHGEF5//WNT5A//CCL24//ADAM8//S100A14 |
| GO:0010594 | regulation of endothelial cell migration | Biological process | 4 | 73 | 208 | 14747 | 3.88487881981033 | 0.0196449905347025 | 0.527226543555867 | 1.70674817610275 | ANXA3//WNT5A//GDF2//EFNA1 |
| GO:0010876 | lipid localization | Biological process | 8 | 238 | 208 | 14747 | 2.38316095669037 | 0.0198054788817275 | 0.527226543555867 | 1.70321465221564 | ABCA12//OSBPL7//APOB//CRP//SLC27A5//LBP//APOC1//MIF |
| GO:0032677 | regulation of interleukin-8 production | Biological process | 3 | 41 | 208 | 14747 | 5.18773452157599 | 0.0198972787832971 | 0.527226543555867 | 1.70120631505806 | GDF2//LBP//WNT5A |
| GO:0070227 | lymphocyte apoptotic process | Biological process | 3 | 41 | 208 | 14747 | 5.18773452157599 | 0.0198972787832971 | 0.527226543555867 | 1.70120631505806 | MIF//ADAM8//WNT5A |
| GO:0006325 | chromatin organization | Biological process | 14 | 536 | 208 | 14747 | 1.85184055683123 | 0.0198995130719346 | 0.527226543555867 | 1.70115755036211 | HIST1H1B//HIST1H2BB//HIST1H2AK//HIST1H2AC//HIST1H2BC//HIST1H2BO//HIST2H2BF//HIST2H4B//HIST2H3D//HIST2H2AA4//PHF13//BAHD1//CDKN2A//SIRT7 |
| GO:0030517 | negative regulation of axon extension | Biological process | 2 | 16 | 208 | 14747 | 8.86237980769231 | 0.0208623624818521 | 0.537797873924068 | 1.68063651303623 | WNT5A//PLXNA3 |
| GO:0045922 | negative regulation of fatty acid metabolic process | Biological process | 2 | 16 | 208 | 14747 | 8.86237980769231 | 0.0208623624818521 | 0.537797873924068 | 1.68063651303623 | APOC1//UGT1A9 |
| GO:0060039 | pericardium development | Biological process | 2 | 16 | 208 | 14747 | 8.86237980769231 | 0.0208623624818521 | 0.537797873924068 | 1.68063651303623 | WNT5A//MECOM |
| GO:0070232 | regulation of T cell apoptotic process | Biological process | 2 | 16 | 208 | 14747 | 8.86237980769231 | 0.0208623624818521 | 0.537797873924068 | 1.68063651303623 | ADAM8//WNT5A |
| GO:0090177 | establishment of planar polarity involved in neural tube closure | Biological process | 2 | 16 | 208 | 14747 | 8.86237980769231 | 0.0208623624818521 | 0.537797873924068 | 1.68063651303623 | WNT5A//CELSR1 |
| GO:0034341 | response to interferon-gamma | Biological process | 5 | 112 | 208 | 14747 | 3.1651356456044 | 0.0211870960868008 | 0.540916384842502 | 1.67392856381311 | IRF5//IRF6//WNT5A//SLC11A1//UBD |
| GO:0019748 | secondary metabolic process | Biological process | 3 | 42 | 208 | 14747 | 5.06421703296703 | 0.0212101832597081 | 0.540916384842502 | 1.67345557908664 | SULT1B1//AK4//UGT1A9 |
| GO:0033674 | positive regulation of kinase activity | Biological process | 12 | 437 | 208 | 14747 | 1.94688435134659 | 0.0213661623247833 | 0.541995894291976 | 1.67027347647145 | EFNA1//WNT5A//IGFBP1//CHI3L1//TNFSF15//SLC11A1//RIPK3//PFKFB1//ADAM8//MIF//FZD5//TRAF4 |
| GO:0030323 | respiratory tube development | Biological process | 6 | 154 | 208 | 14747 | 2.7623001998002 | 0.0220271231992424 | 0.555806087498344 | 1.65704221915777 | CEBPA//CHI3L1//WNT5A//ABCA12//CELSR1//TRAF4 |
| GO:0051704 | multi-organism process | Biological process | 29 | 1399 | 208 | 14747 | 1.46967270577885 | 0.0225902280018511 | 0.558624181173314 | 1.64607938574869 | ANXA3//HP//SLC11A1//HIST1H2BC//S100A14//APOB//AP1S1//SERPINB3//CLDN4//SULT1E1//PI3//LBP//PARD6A//MECOM//ALB//CEBPA//FDPS//KRT7//TNFRSF14//SND1//F11R//PVRL4//MIF//IRF5//NCF2//DUSP10//ITGAX//FZD5//WNT5A |
| GO:0042325 | regulation of phosphorylation | Biological process | 20 | 876 | 208 | 14747 | 1.6186995082543 | 0.0229312662282303 | 0.558624181173314 | 1.63957196355396 | CCNE1//EFNA1//WNT5A//DUSP10//IGFBP3//CBLC//CDKN2A//IGFBP1//CHI3L1//TNFSF15//GDF2//SLC11A1//RIPK3//PFKFB1//MIF//ADAM8//FZD5//PIK3IP1//TRAF4//TNFRSF14 |
| GO:0007250 | activation of NF-kappaB-inducing kinase activity | Biological process | 2 | 17 | 208 | 14747 | 8.34106334841629 | 0.0234273089189899 | 0.558624181173314 | 1.63027759568625 | CHI3L1//TNFSF15 |
| GO:0009698 | phenylpropanoid metabolic process | Biological process | 2 | 17 | 208 | 14747 | 8.34106334841629 | 0.0234273089189899 | 0.558624181173314 | 1.63027759568625 | SULT1B1//UGT1A9 |
| GO:0032402 | melanosome transport | Biological process | 2 | 17 | 208 | 14747 | 8.34106334841629 | 0.0234273089189899 | 0.558624181173314 | 1.63027759568625 | RAB17//BLOC1S3 |
| GO:0034377 | plasma lipoprotein particle assembly | Biological process | 2 | 17 | 208 | 14747 | 8.34106334841629 | 0.0234273089189899 | 0.558624181173314 | 1.63027759568625 | APOB//APOC1 |
| GO:0042249 | establishment of planar polarity of embryonic epithelium | Biological process | 2 | 17 | 208 | 14747 | 8.34106334841629 | 0.0234273089189899 | 0.558624181173314 | 1.63027759568625 | WNT5A//CELSR1 |
| GO:0060572 | morphogenesis of an epithelial bud | Biological process | 2 | 17 | 208 | 14747 | 8.34106334841629 | 0.0234273089189899 | 0.558624181173314 | 1.63027759568625 | WNT5A//SULF1 |
| GO:0061082 | myeloid leukocyte cytokine production | Biological process | 2 | 17 | 208 | 14747 | 8.34106334841629 | 0.0234273089189899 | 0.558624181173314 | 1.63027759568625 | WNT5A//MIF |
| GO:0065005 | protein-lipid complex assembly | Biological process | 2 | 17 | 208 | 14747 | 8.34106334841629 | 0.0234273089189899 | 0.558624181173314 | 1.63027759568625 | APOB//APOC1 |
| GO:1901222 | regulation of NIK/NF-kappaB cascade | Biological process | 2 | 17 | 208 | 14747 | 8.34106334841629 | 0.0234273089189899 | 0.558624181173314 | 1.63027759568625 | CHI3L1//TNFSF15 |
| GO:0006695 | cholesterol biosynthetic process | Biological process | 3 | 44 | 208 | 14747 | 4.83402534965035 | 0.0239752792735539 | 0.568846302764072 | 1.62023632529361 | APOB//FDPS//EBP |
| GO:0016064 | immunoglobulin mediated immune response | Biological process | 5 | 116 | 208 | 14747 | 3.05599303713528 | 0.0242271859799026 | 0.571977474941364 | 1.61569702681885 | C8G//CRP//IGHG1//IGHG3//IGHG4 |
| GO:0051258 | protein polymerization | Biological process | 6 | 159 | 208 | 14747 | 2.67543541364296 | 0.0252842677180825 | 0.59140317998679 | 1.59714961986815 | CCL24//VILL//CRP//FGA//FGB//FGG |
| GO:0051347 | positive regulation of transferase activity | Biological process | 12 | 448 | 208 | 14747 | 1.89908138736264 | 0.0253045816727724 | 0.59140317998679 | 1.59680083791292 | EFNA1//WNT5A//IGFBP1//CHI3L1//TNFSF15//SLC11A1//RIPK3//PFKFB1//ADAM8//MIF//FZD5//TRAF4 |
| GO:0071248 | cellular response to metal ion | Biological process | 4 | 79 | 208 | 14747 | 3.5898247322298 | 0.0254220280765972 | 0.59140317998679 | 1.59478980597157 | ALOX5AP//CRP//WNT5A//CEBPA |
| GO:0052548 | regulation of endopeptidase activity | Biological process | 8 | 250 | 208 | 14747 | 2.26876923076923 | 0.0256477196793369 | 0.593757160926008 | 1.59095124165175 | CDKN2A//TNFSF15//ITIH2//PI3//SERPINB3//SERPINB4//SPINK1//SERPINB7 |
| GO:0032401 | establishment of melanosome localization | Biological process | 2 | 18 | 208 | 14747 | 7.87767094017094 | 0.026114451633267 | 0.595884305450002 | 1.58311908930417 | RAB17//BLOC1S3 |
| GO:0051904 | pigment granule transport | Biological process | 2 | 18 | 208 | 14747 | 7.87767094017094 | 0.026114451633267 | 0.595884305450002 | 1.58311908930417 | RAB17//BLOC1S3 |
| GO:0090342 | regulation of cell aging | Biological process | 2 | 18 | 208 | 14747 | 7.87767094017094 | 0.026114451633267 | 0.595884305450002 | 1.58311908930417 | MIF//CDKN2A |
| GO:0031345 | negative regulation of cell projection organization | Biological process | 4 | 80 | 208 | 14747 | 3.54495192307692 | 0.0264739775251299 | 0.601211422939736 | 1.57718080416737 | WNT5A//PLXNA3//EFNA1//TACSTD2 |
| GO:0019724 | B cell mediated immunity | Biological process | 5 | 119 | 208 | 14747 | 2.97895119586296 | 0.0266827327279543 | 0.602863751170054 | 1.57376969394982 | C8G//CRP//IGHG1//IGHG3//IGHG4 |
| GO:0045859 | regulation of protein kinase activity | Biological process | 15 | 612 | 208 | 14747 | 1.73772153092006 | 0.0268919991106756 | 0.602863751170054 | 1.57037691177953 | CCNE1//EFNA1//WNT5A//DUSP10//CBLC//CDKN2A//IGFBP1//CHI3L1//TNFSF15//SLC11A1//RIPK3//ADAM8//MIF//FZD5//TRAF4 |
| GO:0061387 | regulation of extent of cell growth | Biological process | 3 | 46 | 208 | 14747 | 4.62385033444816 | 0.0269259758857667 | 0.602863751170054 | 1.56982854748688 | CDH4//WNT5A//PLXNA3 |
| GO:0016043 | cellular component organization | Biological process | 72 | 4176 | 208 | 14747 | 1.22239721485411 | 0.027055305984492 | 0.602928758131039 | 1.56774755000991 | PES1//HIST2H4B//ADAM8//JUP//IGFBP3//IL17RB//WNT5A//EFNA1//HIST1H1B//HIST1H2BB//HIST1H2AK//HIST1H2AC//HIST1H2BC//HIST1H2BO//HIST2H2BF//HIST2H3D//HIST2H2AA4//ARHGEF5//KRT7//CCL24//VILL//CDKN2A//SLC7A7//DSG3//CEBPA//SPG7//PHF13//FZD5//CDH4//SPON2//TREM2//PLXNA3//AP1S1//STX4//BLOC1S3//TNFRSF11B//IGFBP1//GDF2//HSPA1B//CDHR2//BAHD1//LAMA3//BIK//SULF1//CELSR1//GRB7//CDH2//CDH3//F11R//PARD6A//PVRL4//CLDN23//APOC1//APOB//UBD//RIPK3//KCTD13//MARVELD3//RAB17//SLC11A1//CRP//FGA//FGB//FGG//PCBD2//KIAA0101//TACSTD2//TARS2//ALOX5AP//MIF//SIRT7//NCF2 |
| GO:0006869 | lipid transport | Biological process | 7 | 208 | 208 | 14747 | 2.38602533284024 | 0.0282567167932196 | 0.625700908885642 | 1.54887830113652 | SLC27A5//LBP//APOB//APOC1//ABCA12//MIF//OSBPL7 |
| GO:0002367 | cytokine production involved in immune response | Biological process | 3 | 47 | 208 | 14747 | 4.5254705400982 | 0.0284707689721502 | 0.625700908885642 | 1.5456008027564 | SLC11A1//WNT5A//MIF |
| GO:0043297 | apical junction assembly | Biological process | 3 | 47 | 208 | 14747 | 4.5254705400982 | 0.0284707689721502 | 0.625700908885642 | 1.5456008027564 | F11R//PARD6A//CLDN23 |
| GO:0006750 | glutathione biosynthetic process | Biological process | 2 | 19 | 208 | 14747 | 7.46305668016194 | 0.0289198684201183 | 0.626903874979746 | 1.53880368733002 | ALOX5AP//OPLAH |
| GO:0051905 | establishment of pigment granule localization | Biological process | 2 | 19 | 208 | 14747 | 7.46305668016194 | 0.0289198684201183 | 0.626903874979746 | 1.53880368733002 | RAB17//BLOC1S3 |
| GO:0072678 | T cell migration | Biological process | 2 | 19 | 208 | 14747 | 7.46305668016194 | 0.0289198684201183 | 0.626903874979746 | 1.53880368733002 | WNT5A//ADAM8 |
| GO:0002576 | platelet degranulation | Biological process | 4 | 83 | 208 | 14747 | 3.41682113067655 | 0.0297849187785138 | 0.634324385231743 | 1.5260035798556 | ALB//FGA//FGB//FGG |
| GO:0033559 | unsaturated fatty acid metabolic process | Biological process | 4 | 83 | 208 | 14747 | 3.41682113067655 | 0.0297849187785138 | 0.634324385231743 | 1.5260035798556 | MIF//MGLL//ALOX5AP//ELOVL2 |
| GO:0071241 | cellular response to inorganic substance | Biological process | 4 | 83 | 208 | 14747 | 3.41682113067655 | 0.0297849187785138 | 0.634324385231743 | 1.5260035798556 | ALOX5AP//CRP//WNT5A//CEBPA |
| GO:0016055 | Wnt receptor signaling pathway | Biological process | 9 | 306 | 208 | 14747 | 2.08526583710407 | 0.0297942256850305 | 0.634324385231743 | 1.52586789686743 | JUP//WNT5A//SULF1//FZD5//CDH2//CELSR1//CCNE1//GRK6//FRAT2 |
| GO:0006826 | iron ion transport | Biological process | 3 | 48 | 208 | 14747 | 4.43118990384615 | 0.0300617190280764 | 0.634807236998564 | 1.52198618865058 | STEAP3//SLC11A1//TFR2 |
| GO:0052547 | regulation of peptidase activity | Biological process | 8 | 258 | 208 | 14747 | 2.19841979725701 | 0.0301541224754764 | 0.634807236998564 | 1.52065330554831 | CDKN2A//TNFSF15//SERPINB3//SERPINB4//ITIH2//PI3//SPINK1//SERPINB7 |
| GO:0002250 | adaptive immune response | Biological process | 7 | 211 | 208 | 14747 | 2.35210080204156 | 0.0302162387919216 | 0.634807236998564 | 1.51975959603848 | SLC11A1//DUSP10//C8G//CRP//IGHG1//IGHG3//IGHG4 |
| GO:0001935 | endothelial cell proliferation | Biological process | 4 | 84 | 208 | 14747 | 3.37614468864469 | 0.0309405660156149 | 0.643405239307635 | 1.50947174573653 | GDF2//SULF1//CCL24//WNT5A |
| GO:0043408 | regulation of MAPK cascade | Biological process | 12 | 462 | 208 | 14747 | 1.84153346653347 | 0.0310578666651491 | 0.643405239307635 | 1.50782837885128 | EFNA1//WNT5A//DUSP10//ADAM8//MIF//CBLC//CDH2//IGFBP3//FZD5//MECOM//TRAF4//CHI3L1 |
| GO:0032642 | regulation of chemokine production | Biological process | 3 | 49 | 208 | 14747 | 4.34075745682889 | 0.0316986878921346 | 0.643405239307635 | 1.49895871421755 | LBP//WNT5A//MIF |
| GO:0010799 | regulation of peptidyl-threonine phosphorylation | Biological process | 2 | 20 | 208 | 14747 | 7.08990384615385 | 0.0318397224736007 | 0.643405239307635 | 1.49703072638377 | CHI3L1//WNT5A |
| GO:0019184 | nonribosomal peptide biosynthetic process | Biological process | 2 | 20 | 208 | 14747 | 7.08990384615385 | 0.0318397224736007 | 0.643405239307635 | 1.49703072638377 | ALOX5AP//OPLAH |
| GO:0032757 | positive regulation of interleukin-8 production | Biological process | 2 | 20 | 208 | 14747 | 7.08990384615385 | 0.0318397224736007 | 0.643405239307635 | 1.49703072638377 | GDF2//LBP |
| GO:0033081 | regulation of T cell differentiation in thymus | Biological process | 2 | 20 | 208 | 14747 | 7.08990384615385 | 0.0318397224736007 | 0.643405239307635 | 1.49703072638377 | CDKN2A//ADAM8 |
| GO:0042403 | thyroid hormone metabolic process | Biological process | 2 | 20 | 208 | 14747 | 7.08990384615385 | 0.0318397224736007 | 0.643405239307635 | 1.49703072638377 | DIO1//SULT1B1 |
| GO:0050829 | defense response to Gram-negative bacterium | Biological process | 2 | 20 | 208 | 14747 | 7.08990384615385 | 0.0318397224736007 | 0.643405239307635 | 1.49703072638377 | LBP//SLC11A1 |
| GO:0045995 | regulation of embryonic development | Biological process | 4 | 85 | 208 | 14747 | 3.33642533936652 | 0.0321223414979573 | 0.643661540351926 | 1.49319280515616 | WNT5A//SULF1//CELSR1//LAMA3 |
| GO:0050714 | positive regulation of protein secretion | Biological process | 4 | 85 | 208 | 14747 | 3.33642533936652 | 0.0321223414979573 | 0.643661540351926 | 1.49319280515616 | WNT5A//MIF//TNFSF15//ADAM8 |
| GO:0002685 | regulation of leukocyte migration | Biological process | 4 | 86 | 208 | 14747 | 3.29762969588551 | 0.0333303084537806 | 0.648990709363778 | 1.4771606671629 | WNT5A//S100A14//LBP//ADAM8 |
| GO:0016126 | sterol biosynthetic process | Biological process | 3 | 50 | 208 | 14747 | 4.25394230769231 | 0.0333815118652805 | 0.648990709363778 | 1.47649399779818 | FDPS//EBP//APOB |
| GO:0045860 | positive regulation of protein kinase activity | Biological process | 11 | 415 | 208 | 14747 | 1.8792516218721 | 0.0336117309531722 | 0.648990709363778 | 1.47350912144739 | EFNA1//WNT5A//IGFBP1//CHI3L1//TNFSF15//SLC11A1//RIPK3//ADAM8//MIF//FZD5//TRAF4 |
| GO:0006576 | cellular biogenic amine metabolic process | Biological process | 5 | 127 | 208 | 14747 | 2.79130072683222 | 0.0339880459469166 | 0.648990709363778 | 1.46867380331532 | AFMID//AGMAT//SMPDL3B//APOC1//SULT1B1 |
| GO:0050708 | regulation of protein secretion | Biological process | 5 | 127 | 208 | 14747 | 2.79130072683222 | 0.0339880459469166 | 0.648990709363778 | 1.46867380331532 | WNT5A//RHBDF2//ADAM8//MIF//TNFSF15 |
| GO:0046486 | glycerolipid metabolic process | Biological process | 9 | 314 | 208 | 14747 | 2.03213804507594 | 0.0343004812754139 | 0.648990709363778 | 1.46469978626072 | APOB//SLC27A5//PCYT2//APOC1//ELOVL2//PNPLA3//MOGAT3//MGLL//MBOAT7 |
| GO:0015849 | organic acid transport | Biological process | 7 | 217 | 208 | 14747 | 2.28706575682382 | 0.0344059021378615 | 0.648990709363778 | 1.46336705027628 | SLC7A7//SLC16A5//ALB//SLC27A5//AQP9//SLC11A1//MIF |
| GO:0046942 | carboxylic acid transport | Biological process | 7 | 217 | 208 | 14747 | 2.28706575682382 | 0.0344059021378615 | 0.648990709363778 | 1.46336705027628 | SLC7A7//SLC16A5//ALB//SLC27A5//AQP9//SLC11A1//MIF |
| GO:0043086 | negative regulation of catalytic activity | Biological process | 13 | 523 | 208 | 14747 | 1.76230879541109 | 0.0344642227990692 | 0.648990709363778 | 1.46263151081208 | DUSP10//CBLC//CDKN2A//SERPINB3//SERPINB4//ITIH2//PI3//SPINK1//SERPINB7//PIK3IP1//APOC1//HP//UGT1A9 |
| GO:0016049 | cell growth | Biological process | 10 | 365 | 208 | 14747 | 1.94243940990516 | 0.0345019316460408 | 0.648990709363778 | 1.46215658957216 | IGFBP3//IL17RB//IGFBP1//CDKN2A//GDF2//HSPA1B//CDHR2//CDH4//WNT5A//PLXNA3 |
| GO:0002720 | positive regulation of cytokine production involved in immune response | Biological process | 2 | 21 | 208 | 14747 | 6.75228937728938 | 0.0348702607767259 | 0.648990709363778 | 1.45754480474706 | WNT5A//MIF |
| GO:0010771 | negative regulation of cell morphogenesis involved in differentiation | Biological process | 2 | 21 | 208 | 14747 | 6.75228937728938 | 0.0348702607767259 | 0.648990709363778 | 1.45754480474706 | EFNA1//TACSTD2 |
| GO:0032400 | melanosome localization | Biological process | 2 | 21 | 208 | 14747 | 6.75228937728938 | 0.0348702607767259 | 0.648990709363778 | 1.45754480474706 | RAB17//BLOC1S3 |
| GO:0046329 | negative regulation of JNK cascade | Biological process | 2 | 21 | 208 | 14747 | 6.75228937728938 | 0.0348702607767259 | 0.648990709363778 | 1.45754480474706 | MECOM//DUSP10 |
| GO:2000401 | regulation of lymphocyte migration | Biological process | 2 | 21 | 208 | 14747 | 6.75228937728938 | 0.0348702607767259 | 0.648990709363778 | 1.45754480474706 | WNT5A//ADAM8 |
| GO:0051239 | regulation of multicellular organismal process | Biological process | 35 | 1822 | 208 | 14747 | 1.36194640293844 | 0.034902178965933 | 0.648990709363778 | 1.45714745890498 | SLC11A1//WNT5A//JUP//SULF1//TACSTD2//EFNA1//APOC1//GDF2//RIPK3//LBP//IRF5//FZD5//MAPK13//CDKN2A//ADAM8//TNFRSF11B//HSPA1B//HIST2H4B//IGFBP3//CEBPA//ANXA3//CHI3L1//CCL24//CDH4//LAMA3//ALB//PLXNA3//MIF//TNFSF15//RAB17//MGLL//DUSP10//P2RY2//CELSR1//CRP |
| GO:0001938 | positive regulation of endothelial cell proliferation | Biological process | 3 | 51 | 208 | 14747 | 4.17053167420814 | 0.0351100027292629 | 0.648990709363778 | 1.45456913677493 | GDF2//CCL24//WNT5A |
| GO:0043588 | skin development | Biological process | 3 | 51 | 208 | 14747 | 4.17053167420814 | 0.0351100027292629 | 0.648990709363778 | 1.45456913677493 | ABCA12//IRF6//JUP |
| GO:0043604 | amide biosynthetic process | Biological process | 3 | 51 | 208 | 14747 | 4.17053167420814 | 0.0351100027292629 | 0.648990709363778 | 1.45456913677493 | CEBPA//ALOX5AP//OPLAH |
| GO:0060688 | regulation of morphogenesis of a branching structure | Biological process | 3 | 51 | 208 | 14747 | 4.17053167420814 | 0.0351100027292629 | 0.648990709363778 | 1.45456913677493 | WNT5A//SULF1//TACSTD2 |
| GO:0048520 | positive regulation of behavior | Biological process | 4 | 88 | 208 | 14747 | 3.22268356643357 | 0.0358250248566522 | 0.659650747263994 | 1.44581349960185 | WNT5A//ALB//S100A14//LBP |
| GO:0030168 | platelet activation | Biological process | 7 | 220 | 208 | 14747 | 2.2558784965035 | 0.0366388624361355 | 0.672041288299731 | 1.43605801877544 | ALB//FGA//FGB//FGG//STX4//MGLL//BLOC1S3 |
| GO:0002690 | positive regulation of leukocyte chemotaxis | Biological process | 3 | 52 | 208 | 14747 | 4.09032914201183 | 0.0368839487379066 | 0.673944641881519 | 1.43316259024091 | WNT5A//S100A14//LBP |
| GO:0002443 | leukocyte mediated immunity | Biological process | 7 | 221 | 208 | 14747 | 2.24567090149669 | 0.0374039698885554 | 0.676340514579216 | 1.42708230129695 | SLC11A1//C8G//CRP//IGHG1//IGHG3//IGHG4//ANXA3 |
| GO:0043410 | positive regulation of MAPK cascade | Biological process | 9 | 320 | 208 | 14747 | 1.99403545673077 | 0.0379751619132489 | 0.676340514579216 | 1.42050036577642 | EFNA1//WNT5A//ADAM8//MIF//FZD5//TRAF4//CHI3L1//CDH2//IGFBP3 |
| GO:0002675 | positive regulation of acute inflammatory response | Biological process | 2 | 22 | 208 | 14747 | 6.44536713286713 | 0.0380078125198637 | 0.676340514579216 | 1.42012712481653 | ADAM8//ALOX5AP |
| GO:0009950 | dorsal/ventral axis specification | Biological process | 2 | 22 | 208 | 14747 | 6.44536713286713 | 0.0380078125198637 | 0.676340514579216 | 1.42012712481653 | FZD5//WNT5A |
| GO:0048247 | lymphocyte chemotaxis | Biological process | 2 | 22 | 208 | 14747 | 6.44536713286713 | 0.0380078125198637 | 0.676340514579216 | 1.42012712481653 | WNT5A//ADAM8 |
| GO:0050922 | negative regulation of chemotaxis | Biological process | 2 | 22 | 208 | 14747 | 6.44536713286713 | 0.0380078125198637 | 0.676340514579216 | 1.42012712481653 | WNT5A//PLXNA3 |
| GO:0051875 | pigment granule localization | Biological process | 2 | 22 | 208 | 14747 | 6.44536713286713 | 0.0380078125198637 | 0.676340514579216 | 1.42012712481653 | RAB17//BLOC1S3 |
| GO:0000165 | MAPK cascade | Biological process | 13 | 531 | 208 | 14747 | 1.73575800376648 | 0.0381971003068002 | 0.677182049677064 | 1.4179696048515 | EFNA1//WNT5A//DUSP10//ADAM8//MIF//CBLC//CDH2//IGFBP3//FZD5//MECOM//TRAF4//CHI3L1//MAPK13 |
| GO:0032940 | secretion by cell | Biological process | 16 | 698 | 208 | 14747 | 1.62519285871721 | 0.0385098378063821 | 0.680197838883838 | 1.41442831051104 | ALB//FGA//FGB//FGG//WNT5A//LLGL2//SLC17A9//SLC2A1//STEAP3//BLOC1S3//ANXA3//RHBDF2//ADAM8//MIF//TNFSF15//CHI3L1 |
| GO:0032602 | chemokine production | Biological process | 3 | 53 | 208 | 14747 | 4.01315312046444 | 0.0387031155818574 | 0.681089144685896 | 1.41225407308068 | LBP//WNT5A//MIF |
| GO:0046328 | regulation of JNK cascade | Biological process | 5 | 133 | 208 | 14747 | 2.66537738577212 | 0.0402086368589517 | 0.690901387906233 | 1.39568064996772 | WNT5A//FZD5//DUSP10//MECOM//TRAF4 |
| GO:0034612 | response to tumor necrosis factor | Biological process | 4 | 92 | 208 | 14747 | 3.08256688963211 | 0.041130551859184 | 0.690901387906233 | 1.38583546341106 | TNFRSF14//APOB//CHI3L1//UBD |
| GO:0000083 | regulation of transcription involved in G1/S phase of mitotic cell cycle | Biological process | 2 | 23 | 208 | 14747 | 6.16513377926421 | 0.0412487875467521 | 0.690901387906233 | 1.38458881243512 | CCNE1//CDC45 |
| GO:0007398 | ectoderm development | Biological process | 2 | 23 | 208 | 14747 | 6.16513377926421 | 0.0412487875467521 | 0.690901387906233 | 1.38458881243512 | JUP//KRT6A |
| GO:0008089 | anterograde axon cargo transport | Biological process | 2 | 23 | 208 | 14747 | 6.16513377926421 | 0.0412487875467521 | 0.690901387906233 | 1.38458881243512 | SPG7//BLOC1S3 |
| GO:0016338 | calcium-independent cell-cell adhesion | Biological process | 2 | 23 | 208 | 14747 | 6.16513377926421 | 0.0412487875467521 | 0.690901387906233 | 1.38458881243512 | CLDN4//CLDN23 |
| GO:0034367 | macromolecular complex remodeling | Biological process | 2 | 23 | 208 | 14747 | 6.16513377926421 | 0.0412487875467521 | 0.690901387906233 | 1.38458881243512 | APOC1//APOB |
| GO:0034368 | protein-lipid complex remodeling | Biological process | 2 | 23 | 208 | 14747 | 6.16513377926421 | 0.0412487875467521 | 0.690901387906233 | 1.38458881243512 | APOC1//APOB |
| GO:0034369 | plasma lipoprotein particle remodeling | Biological process | 2 | 23 | 208 | 14747 | 6.16513377926421 | 0.0412487875467521 | 0.690901387906233 | 1.38458881243512 | APOB//APOC1 |
| GO:0038061 | NIK/NF-kappaB cascade | Biological process | 2 | 23 | 208 | 14747 | 6.16513377926421 | 0.0412487875467521 | 0.690901387906233 | 1.38458881243512 | CHI3L1//TNFSF15 |
| GO:0048846 | axon extension involved in axon guidance | Biological process | 2 | 23 | 208 | 14747 | 6.16513377926421 | 0.0412487875467521 | 0.690901387906233 | 1.38458881243512 | WNT5A//PLXNA3 |
| GO:0061311 | cell surface receptor signaling pathway involved in heart development | Biological process | 2 | 23 | 208 | 14747 | 6.16513377926421 | 0.0412487875467521 | 0.690901387906233 | 1.38458881243512 | JUP//WNT5A |
| GO:0070231 | T cell apoptotic process | Biological process | 2 | 23 | 208 | 14747 | 6.16513377926421 | 0.0412487875467521 | 0.690901387906233 | 1.38458881243512 | ADAM8//WNT5A |
| GO:0001932 | regulation of protein phosphorylation | Biological process | 18 | 819 | 208 | 14747 | 1.55822062552832 | 0.0414048044086255 | 0.690901387906233 | 1.38294926257246 | CCNE1//EFNA1//WNT5A//DUSP10//IGFBP3//CBLC//CDKN2A//IGFBP1//CHI3L1//TNFSF15//GDF2//SLC11A1//RIPK3//MIF//ADAM8//FZD5//TRAF4//TNFRSF14 |
| GO:0002449 | lymphocyte mediated immunity | Biological process | 6 | 179 | 208 | 14747 | 2.37650408250967 | 0.0414710727185158 | 0.690901387906233 | 1.38225473073903 | SLC11A1//C8G//CRP//IGHG1//IGHG3//IGHG4 |
| GO:1901700 | response to oxygen-containing compound | Biological process | 17 | 762 | 208 | 14747 | 1.58173707853826 | 0.0415786744242166 | 0.690901387906233 | 1.38112936058956 | APOB//NCF2//LBP//MIF//IRF5//SLC11A1//DUSP10//S100A14//CLDN4//HP//OLR1//CRP//PFKFB1//WNT5A//AQP9//OSBPL7//JUP |
| GO:0001775 | cell activation | Biological process | 17 | 763 | 208 | 14747 | 1.57966402863192 | 0.0420084260872126 | 0.695618694478878 | 1.3766635896937 | LBP//SLC11A1//ALB//FGA//FGB//FGG//STX4//MGLL//BLOC1S3//CDKN2A//MIF//TNFRSF14//FZD5//ADAM8//UBD//WNT5A//ANXA3 |
| GO:0033993 | response to lipid | Biological process | 13 | 539 | 208 | 14747 | 1.70999536178108 | 0.0422101017687653 | 0.696111220260715 | 1.37458360075817 | LBP//MIF//APOB//NCF2//SLC11A1//DUSP10//S100A14//CLDN4//TNFRSF11B//CEBPA//PFKFB1//WNT5A//OSBPL7 |
| GO:0006970 | response to osmotic stress | Biological process | 3 | 55 | 208 | 14747 | 3.86722027972028 | 0.0424760673298109 | 0.696111220260715 | 1.37185569946013 | AQP9//MAPK13//SLC2A1 |
| GO:0045833 | negative regulation of lipid metabolic process | Biological process | 3 | 55 | 208 | 14747 | 3.86722027972028 | 0.0424760673298109 | 0.696111220260715 | 1.37185569946013 | APOC1//PIK3IP1//UGT1A9 |
| GO:0030335 | positive regulation of cell migration | Biological process | 7 | 228 | 208 | 14747 | 2.17672486504723 | 0.0430552283959893 | 0.703186247330387 | 1.3659741026156 | ANXA3//WNT5A//S100A14//LBP//ADAM8//GRB7//CCL24 |
| GO:0032787 | monocarboxylic acid metabolic process | Biological process | 11 | 433 | 208 | 14747 | 1.801130307337 | 0.0434722807843406 | 0.707574426827714 | 1.36178757384025 | SLC27A5//MIF//ACSM3//MGLL//HSD3B7//ALB//ALOX5AP//SULT2A1//ELOVL2//UGT1A9//APOC1 |
| GO:0019432 | triglyceride biosynthetic process | Biological process | 3 | 56 | 208 | 14747 | 3.79816277472527 | 0.0444292791221798 | 0.709266786234182 | 1.35233073325472 | ELOVL2//PNPLA3//MOGAT3 |
| GO:0000038 | very long-chain fatty acid metabolic process | Biological process | 2 | 24 | 208 | 14747 | 5.90825320512821 | 0.0445896748276554 | 0.709266786234182 | 1.35076569475221 | ELOVL2//SLC27A5 |
| GO:0003179 | heart valve morphogenesis | Biological process | 2 | 24 | 208 | 14747 | 5.90825320512821 | 0.0445896748276554 | 0.709266786234182 | 1.35076569475221 | EFNA1//JUP |
| GO:0015893 | drug transport | Biological process | 2 | 24 | 208 | 14747 | 5.90825320512821 | 0.0445896748276554 | 0.709266786234182 | 1.35076569475221 | EBP//MFSD10 |
| GO:0030104 | water homeostasis | Biological process | 2 | 24 | 208 | 14747 | 5.90825320512821 | 0.0445896748276554 | 0.709266786234182 | 1.35076569475221 | ABCA12//AQP9 |
| GO:0032101 | regulation of response to external stimulus | Biological process | 10 | 382 | 208 | 14747 | 1.85599577124446 | 0.0446938929657931 | 0.709266786234182 | 1.34975181540167 | ADAM8//ALOX5AP//WNT5A//PLXNA3//MGLL//MAPK13//CCL24//LBP//DUSP10//S100A14 |
| GO:0050795 | regulation of behavior | Biological process | 5 | 137 | 208 | 14747 | 2.58755614823133 | 0.0447159020357919 | 0.709266786234182 | 1.34953800397653 | WNT5A//ALB//PLXNA3//S100A14//LBP |
| GO:0031349 | positive regulation of defense response | Biological process | 7 | 230 | 208 | 14747 | 2.15779682274247 | 0.0447660521401738 | 0.709266786234182 | 1.3490512037925 | ADAM8//ALOX5AP//S100A14//LBP//MAPK13//CCL24//WNT5A |
| GO:0006935 | chemotaxis | Biological process | 14 | 600 | 208 | 14747 | 1.6543108974359 | 0.0451429949238584 | 0.709669264935123 | 1.345409631883 | LBP//ARHGEF5//CDH4//WNT5A//SPON2//TREM2//PLXNA3//CCL24//ADAM8//MIF//S100A14//CXCL6//CXCL5//CCL3L3 |
| GO:0042330 | taxis | Biological process | 14 | 600 | 208 | 14747 | 1.6543108974359 | 0.0451429949238584 | 0.709669264935123 | 1.345409631883 | LBP//ARHGEF5//CCL24//CXCL6//CXCL5//CCL3L3//CDH4//WNT5A//SPON2//TREM2//PLXNA3//ADAM8//MIF//S100A14 |
| GO:0044710 | single-organism metabolic process | Biological process | 54 | 3083 | 208 | 14747 | 1.24182551959879 | 0.0453168524393366 | 0.709669264935123 | 1.34374026243836 | SLC27A5//CEBPA//MIF//MOGAT3//PFKFB1//SLC2A1//CYP2S1//CYB561//ERO1L//CYB561D1//IMPDH1//SWSAP1//TARS2//SLC7A7//YOD1//PYCR1//AFMID//DIO1//ALB//APOB//APOC1//MGLL//ACP6//ACSM3//PCYT2//MBOAT7//PNPLA3//SMPDL3B//FDPS//EBP//HSD3B7//ALOX5AP//OPLAH//TPK1//MOCOS//SULT1E1//SULT2A1//SULT1B1//AK4//HRASLS2//LSR//ELOVL2//SULF1//WNT5A//ARHGEF5//CCL24//AGMAT//UGT1A9//PIK3IP1//GALK1//NCF2//SLC11A1//LBP//DUSP10 |
| GO:0050663 | cytokine secretion | Biological process | 4 | 95 | 208 | 14747 | 2.98522267206478 | 0.0453866902506212 | 0.709669264935123 | 1.34307148630982 | WNT5A//MIF//TNFSF15//CHI3L1 |
| GO:0071902 | positive regulation of protein serine/threonine kinase activity | Biological process | 7 | 231 | 208 | 14747 | 2.14845571095571 | 0.0456376761800191 | 0.711261691838271 | 1.34067647748538 | EFNA1//WNT5A//CHI3L1//TNFSF15//ADAM8//MIF//FZD5 |
| GO:0050715 | positive regulation of cytokine secretion | Biological process | 3 | 57 | 208 | 14747 | 3.73152834008097 | 0.0464265672796539 | 0.713401160812229 | 1.33323342631912 | WNT5A//MIF//TNFSF15 |
| GO:0006790 | sulfur compound metabolic process | Biological process | 7 | 232 | 208 | 14747 | 2.1391951259947 | 0.0465201524875784 | 0.713401160812229 | 1.33235887037512 | ALOX5AP//OPLAH//TPK1//SULF1//SULT1E1//SULT2A1//SULT1B1 |
| GO:0048514 | blood vessel morphogenesis | Biological process | 11 | 438 | 208 | 14747 | 1.78056945907973 | 0.0465325795437044 | 0.713401160812229 | 1.33224287159 | ADAM8//EFNA1//FZD5//GDF2//SULF1//ANXA3//CHI3L1//CCL24//WNT5A//APOB//CDH2 |
| GO:0006909 | phagocytosis | Biological process | 4 | 96 | 208 | 14747 | 2.9541266025641 | 0.0468581223034286 | 0.713401160812229 | 1.32921511841934 | CRP//LBP//SLC11A1//ANXA3 |
| GO:0080135 | regulation of cellular response to stress | Biological process | 8 | 282 | 208 | 14747 | 2.01132024004364 | 0.0468662580563121 | 0.713401160812229 | 1.32913972047958 | WNT5A//DUSP10//FZD5//CDKN2A//MIF//MECOM//TRAF4//HP |
| GO:0006694 | steroid biosynthetic process | Biological process | 5 | 139 | 208 | 14747 | 2.55032512451577 | 0.0470785446456681 | 0.713401160812229 | 1.32717697110738 | FDPS//EBP//SLC27A5//HSD3B7//APOB |
| GO:0071840 | cellular component organization or biogenesis | Biological process | 72 | 4290 | 208 | 14747 | 1.18991393222162 | 0.0471827073986279 | 0.713401160812229 | 1.32621714241293 | PES1//HIST2H4B//ADAM8//JUP//IGFBP3//IL17RB//WNT5A//EFNA1//HIST1H1B//HIST1H2BB//HIST1H2AK//HIST1H2AC//HIST1H2BC//HIST1H2BO//HIST2H2BF//HIST2H3D//HIST2H2AA4//ARHGEF5//KRT7//CCL24//VILL//CDKN2A//SLC7A7//DSG3//CEBPA//SPG7//PHF13//FZD5//CDH4//SPON2//TREM2//PLXNA3//AP1S1//STX4//BLOC1S3//TNFRSF11B//IGFBP1//GDF2//HSPA1B//CDHR2//BAHD1//LAMA3//BIK//SULF1//CELSR1//GRB7//CDH2//CDH3//F11R//PARD6A//PVRL4//CLDN23//APOC1//APOB//UBD//RIPK3//KCTD13//MARVELD3//RAB17//SLC11A1//CRP//FGA//FGB//FGG//PCBD2//KIAA0101//TACSTD2//TARS2//ALOX5AP//MIF//SIRT7//NCF2 |
| GO:2000147 | positive regulation of cell motility | Biological process | 7 | 233 | 208 | 14747 | 2.13001403103334 | 0.0474135126310386 | 0.713401160812229 | 1.32409786877371 | ANXA3//WNT5A//GRB7//CCL24//S100A14//LBP//ADAM8 |
| GO:0001937 | negative regulation of endothelial cell proliferation | Biological process | 2 | 25 | 208 | 14747 | 5.67192307692308 | 0.0480270409592181 | 0.713401160812229 | 1.31851417027695 | GDF2//SULF1 |
| GO:0040036 | regulation of fibroblast growth factor receptor signaling pathway | Biological process | 2 | 25 | 208 | 14747 | 5.67192307692308 | 0.0480270409592181 | 0.713401160812229 | 1.31851417027695 | WNT5A//SULF1 |
| GO:0043516 | regulation of DNA damage response, signal transduction by p53 class mediator | Biological process | 2 | 25 | 208 | 14747 | 5.67192307692308 | 0.0480270409592181 | 0.713401160812229 | 1.31851417027695 | CDKN2A//MIF |
| GO:0045124 | regulation of bone resorption | Biological process | 2 | 25 | 208 | 14747 | 5.67192307692308 | 0.0480270409592181 | 0.713401160812229 | 1.31851417027695 | TNFRSF11B//ADAM8 |
| GO:1901796 | regulation of signal transduction by p53 class mediator | Biological process | 2 | 25 | 208 | 14747 | 5.67192307692308 | 0.0480270409592181 | 0.713401160812229 | 1.31851417027695 | CDKN2A//MIF |
| GO:1901615 | organic hydroxy compound metabolic process | Biological process | 10 | 387 | 208 | 14747 | 1.83201649771417 | 0.0480442835362968 | 0.713401160812229 | 1.31835827867872 | MOGAT3//DIO1//SMPDL3B//FDPS//EBP//APOB//CEBPA//APOC1//SULT1B1//AK4 |
| GO:0050770 | regulation of axonogenesis | Biological process | 4 | 97 | 208 | 14747 | 2.92367168913561 | 0.048355870830428 | 0.713401160812229 | 1.3155507911885 | CDH4//WNT5A//PLXNA3//EFNA1 |
| GO:0046460 | neutral lipid biosynthetic process | Biological process | 3 | 58 | 208 | 14747 | 3.66719164456233 | 0.0484675982602563 | 0.713401160812229 | 1.31454850056115 | ELOVL2//PNPLA3//MOGAT3 |
| GO:0046463 | acylglycerol biosynthetic process | Biological process | 3 | 58 | 208 | 14747 | 3.66719164456233 | 0.0484675982602563 | 0.713401160812229 | 1.31454850056115 | ELOVL2//PNPLA3//MOGAT3 |
| GO:0048645 | organ formation | Biological process | 3 | 58 | 208 | 14747 | 3.66719164456233 | 0.0484675982602563 | 0.713401160812229 | 1.31454850056115 | WNT5A//SULF1 |
| GO:0032147 | activation of protein kinase activity | Biological process | 7 | 235 | 208 | 14747 | 2.11188625204583 | 0.0492330044675374 | 0.722437533248264 | 1.30774366039101 | EFNA1//WNT5A//CHI3L1//TNFSF15//ADAM8//SLC11A1//RIPK3 |
