# Supplementary material for: Downregulation of miR-181b-5p Inhibits the Viability, Migration, and Glycolysis of Gallbladder Cancer by Upregulating PDHX Under Hypoxia
Source: Front Oncol. 2021 Aug 16;11:683725. doi: 10.3389/fonc.2021.683725 (PMC8415503; doi:10.3389/fonc.2021.683725)
Supplement: Supplementary file 3 [file DataSheet_1.zip › RNA seq raw data/HuGene 2.0 ST Data/GO Analysis/A vs B_down/MF_result(Human).html]

| GO.ID | Term | Ontology | Count | Pop.Hits | List.Total | Pop.Total | Fold.Enrichment | Pvalue | FDR | Enrichment.Score | GENES |
| --- | --- | --- | --- | --- | --- | --- | --- | --- | --- | --- | --- |
| GO:0005539 | glycosaminoglycan binding | Molecular function | 27 | 172 | 434 | 15325 | 5.54301521809024 | 4.02382337295305e-13 | 3.90310867176446e-10 | 12.3953610910325 | CD44//LYVE1//SUSD5//CTSG//ECM2//FGF2//FGF7//FGF10//FGFR1//CFH//PTN//SELP//SFRP1//SOD3//TGFBR3//TNXB//SLIT2//LIPG//ADAMTS1//ABI3BP//SMOC2//CRISPLD2//CCDC80//DPYSL3//VIT//TGFBR2//MAMDC2 |
| GO:0097367 | carbohydrate derivative binding | Molecular function | 28 | 191 | 434 | 15325 | 5.17649045769296 | 8.3463753854382e-13 | 4.04799206193753e-10 | 12.0785020864158 | LGALS2//VIT//TGFBR2//TGFBR3//MAMDC2//CD44//LYVE1//SUSD5//CTSG//ECM2//FGF2//FGF7//FGF10//FGFR1//CFH//PTN//SELP//SFRP1//SOD3//TNXB//SLIT2//LIPG//ADAMTS1//ABI3BP//SMOC2//CRISPLD2//CCDC80//DPYSL3 |
| GO:1901681 | sulfur compound binding | Molecular function | 25 | 168 | 434 | 15325 | 5.25462201009436 | 1.02584007539713e-11 | 3.31688291045072e-09 | 10.9889203388208 | CTSG//ECM2//FGF2//FGF7//FGF10//FGFR1//CFH//PTN//SELP//SFRP1//SOD3//TGFBR3//TNXB//SLIT2//LIPG//ADAMTS1//ABI3BP//SMOC2//CRISPLD2//CCDC80//DPYSL3//CD34//GPC5//GPC3//GPC6 |
| GO:0008201 | heparin binding | Molecular function | 20 | 129 | 434 | 15325 | 5.47458293144715 | 5.88793009231156e-10 | 1.42782304738555e-07 | 9.23003735504005 | CTSG//ECM2//FGF2//FGF7//FGF10//FGFR1//CFH//PTN//SELP//SFRP1//SOD3//TGFBR3//TNXB//SLIT2//LIPG//ADAMTS1//ABI3BP//SMOC2//CRISPLD2//CCDC80 |
| GO:0003779 | actin binding | Molecular function | 27 | 356 | 434 | 15325 | 2.67808600424584 | 3.50288024602807e-06 | 0.000679558767729446 | 5.45557470977857 | MYH10//TTN//NEXN//CFL2//CNN1//CNN3//DMD//EPB41L2//FLNC//KCNMA1//MYH11//OPHN1//TAGLN//TMOD1//TNS1//TPM2//CAP2//SORBS1//IQGAP2//WASF3//DAAM2//LMOD1//TMOD2//PARVA//KLHL4//NCALD//SYNPO2 |
| GO:0019955 | cytokine binding | Molecular function | 9 | 58 | 434 | 15325 | 5.4793023994915 | 3.35351443713655e-05 | 0.00542151500670409 | 4.47449981964829 | CD36//TGFBR2//TGFBR3//DARC//A2M//IL6ST//CNTFR//KIT//FZD4 |
| GO:0019838 | growth factor binding | Molecular function | 12 | 105 | 434 | 15325 | 4.03554970375247 | 4.12522475036001e-05 | 0.00571638286835601 | 4.38455238525171 | NTRK2//IGFBP4//CD36//TGFBR2//TGFBR3//FGF2//FGFR1//A2M//IL6ST//KDR//GHR//LIFR |
| GO:0008092 | cytoskeletal protein binding | Molecular function | 36 | 635 | 434 | 15325 | 2.00188686091658 | 6.10845518106973e-05 | 0.00740650190704705 | 4.21406860827879 | CFL2//CNN1//CNN3//DMD//EPB41L2//FLNC//KCNMA1//MYH10//MYH11//OPHN1//TAGLN//TMOD1//TNS1//TPM2//CAP2//SORBS1//IQGAP2//WASF3//DAAM2//LMOD1//TMOD2//PARVA//KLHL4//NCALD//SYNPO2//TTN//NEXN//CAPN6//CETN2//CRYAB//PRNP//ACTC1//SYNM//ANK2//AMPD1//DES |
| GO:0019199 | transmembrane receptor protein kinase activity | Molecular function | 10 | 82 | 434 | 15325 | 4.30622681802855 | 0.000103528558409296 | 0.0111580779618908 | 3.98493983331085 | TGFBR2//KDR//KIT//MUSK//ROR1//TEK//EFEMP1//FGFR1//TGFBR3//NTRK2 |
| GO:0043394 | proteoglycan binding | Molecular function | 5 | 19 | 434 | 15325 | 9.29238418627213 | 0.00014901631534959 | 0.0144545825889102 | 3.82676617938377 | GPC5//GPC3//CFH//GPC6//SLIT2 |
| GO:0001948 | glycoprotein binding | Molecular function | 8 | 58 | 434 | 15325 | 4.87049102177022 | 0.000214841788686891 | 0.018945139547844 | 3.66788124051916 | DMD//SLIT2//GPC5//GPC3//CFH//GPC6//CNTN1//SELP |
| GO:0005518 | collagen binding | Molecular function | 7 | 46 | 434 | 15325 | 5.3734221598878 | 0.000286629578675503 | 0.0213869762550183 | 3.54267899470379 | ECM2//CD44//TLL1//TNXB//COL14A1//ABI3BP//PODN |
| GO:0008307 | structural constituent of muscle | Molecular function | 7 | 46 | 434 | 15325 | 5.3734221598878 | 0.000286629578675503 | 0.0213869762550183 | 3.54267899470379 | DMD//MYH11//TPM2//TTN//MYL9//SYNM//NEXN |
| GO:0004871 | signal transducer activity | Molecular function | 69 | 1635 | 434 | 15325 | 1.49019151904621 | 0.000450354541273272 | 0.0283140226179588 | 3.34644545357088 | KCNH1//PDE8B//CALCRL//LPAR1//NR4A2//PTGER3//RORB//NR2F1//TGFBR2//MAPK4//KDR//KIT//MUSK//ROR1//TEK//CD44//DARC//KLRB1//TRGC2//LYVE1//SLC22A17//CNTFR//IL6ST//LIFR//GHR//FRZB//SFRP1//FZD4//BAI3//GLP2R//GPR64//LPHN3//ELTD1//OR4F5//OR8J3//OR4A5//OR56B1//OR4F21//BDKRB1//CCRL1//PTGFR//EDNRB//PTPRD//PTPRS//EFEMP1//FGFR1//TGFBR3//EDA2R//DCLK1//DOK5//SMAD9//COLEC12//ABCC9//GFRA1//CHRM2//FCER1A//CD160//NTRK2//CD36//BMX//EPAS1//ERG//GNAL//GNG7//GNG11//PPP1R12A//PLCB4//SLC44A2//PLCXD3 |
| GO:0060089 | molecular transducer activity | Molecular function | 69 | 1635 | 434 | 15325 | 1.49019151904621 | 0.000450354541273272 | 0.0283140226179588 | 3.34644545357088 | KCNH1//PDE8B//CALCRL//LPAR1//NR4A2//PTGER3//RORB//NR2F1//TGFBR2//MAPK4//KDR//KIT//MUSK//ROR1//TEK//BMX//EPAS1//ERG//GNAL//GNG7//GNG11//PPP1R12A//PLCB4//SLC44A2//PLCXD3//CD44//DARC//KLRB1//TRGC2//LYVE1//SLC22A17//CNTFR//IL6ST//LIFR//GHR//FRZB//SFRP1//FZD4//BAI3//GLP2R//GPR64//LPHN3//ELTD1//OR4F5//OR8J3//OR4A5//OR56B1//OR4F21//BDKRB1//CCRL1//PTGFR//EDNRB//PTPRD//PTPRS//EFEMP1//FGFR1//TGFBR3//EDA2R//DCLK1//DOK5//SMAD9//COLEC12//ABCC9//GFRA1//CHRM2//FCER1A//CD160//NTRK2//CD36 |
| GO:0004714 | transmembrane receptor protein tyrosine kinase activity | Molecular function | 8 | 65 | 434 | 15325 | 4.34597660404112 | 0.000476124246830353 | 0.0283140226179588 | 3.32227970133059 | EFEMP1//FGFR1//KIT//KDR//NTRK2//MUSK//ROR1//TEK |
| GO:0043395 | heparan sulfate proteoglycan binding | Molecular function | 4 | 14 | 434 | 15325 | 10.0888742593812 | 0.000506674711435646 | 0.0283140226179588 | 3.29527077117415 | GPC5//GPC3//CFH//GPC6 |
| GO:0005102 | receptor binding | Molecular function | 54 | 1207 | 434 | 15325 | 1.57978229910774 | 0.00052541485270439 | 0.0283140226179588 | 3.27949765451077 | GNAL//PIK3R1//FGF2//FGF10//NTRK2//ANKS1B//SFRP1//CAV1//TGFBR3//SCG2//IL6ST//LIFR//EFEMP1//IGF1//SORBS1//DOK5//ECM2//GFRA1//KDR//TNXB//VCAM1//PPAP2B//FBLN5//PTHLH//RLN1//VIP//ANGPT1//CCL21//CXCL12//FGF7//OGN//PTN//HDGFRP3//PDGFD//EDNRB//C3//TGFBR2//OPHN1//SLIT3//SLIT2//PLSCR4//TCF21//ANXA1//CDH5//CNTFR//ECH1//LAMA2//MFAP4//PTPRD//ANGPTL1//ECI2//CD160//CROT//CNTNAP3 |
| GO:0005523 | tropomyosin binding | Molecular function | 4 | 15 | 434 | 15325 | 9.41628264208909 | 0.000675550488819697 | 0.0344886302186898 | 3.1703421874319 | CNN3//TMOD1//LMOD1//TMOD2 |
| GO:0004713 | protein tyrosine kinase activity | Molecular function | 12 | 145 | 434 | 15325 | 2.92229461306213 | 0.000865068934697842 | 0.0419558433328453 | 3.06294928355713 | KDR//KIT//MUSK//ROR1//TEK//BMX//WEE1//EFEMP1//FGFR1//NTRK2//TTN//DYRK3 |
| GO:0017147 | Wnt-protein binding | Molecular function | 5 | 28 | 434 | 15325 | 6.30554641211323 | 0.00102095752611669 | 0.0471585143015804 | 2.99099232505985 | FRZB//ROR1//SFRP1//FZD4//APCDD1 |
| GO:0005178 | integrin binding | Molecular function | 8 | 81 | 434 | 15325 | 3.48751208966263 | 0.00204910799079543 | 0.0866309454274758 | 2.68843515308212 | ECM2//GFRA1//IGF1//KDR//TNXB//VCAM1//PPAP2B//FBLN5 |
| GO:0004857 | enzyme inhibitor activity | Molecular function | 19 | 322 | 434 | 15325 | 2.08357185791568 | 0.00213733572030827 | 0.0866309454274758 | 2.67012725601753 | PTN//PPP1R1C//C3//CST3//RECK//A2M//SERPINA6//SERPINF1//TFPI2//WFDC1//CST4//SLIT2//IQGAP2//TIMP3//PI16//ANXA1//PLN//GPC3//PPP1R12A |
| GO:0042056 | chemoattractant activity | Molecular function | 4 | 20 | 434 | 15325 | 7.06221198156682 | 0.00214344607243239 | 0.0866309454274758 | 2.66888743855393 | FGF2//FGF7//FGF10//SCG2 |
| GO:0061134 | peptidase regulator activity | Molecular function | 14 | 206 | 434 | 15325 | 2.39978077043533 | 0.00225136630052082 | 0.0873530124602078 | 2.64755383891652 | C3//CST3//RECK//A2M//SERPINA6//SERPINF1//TFPI2//WFDC1//CST4//TIMP3//CAV1//FBLN1//PI16//GPC3 |
| GO:0008195 | phosphatidate phosphatase activity | Molecular function | 3 | 11 | 434 | 15325 | 9.63028906577294 | 0.00314132193078154 | 0.116875979112503 | 2.50288755370097 | PPAP2A//PPAP2B//LPPR4 |
| GO:0005100 | Rho GTPase activator activity | Molecular function | 5 | 36 | 434 | 15325 | 4.90431387608807 | 0.00325324890313154 | 0.116875979112503 | 2.48768270796892 | PREX2//ARHGAP6//OPHN1//DLC1//ERRFI1 |
| GO:0030414 | peptidase inhibitor activity | Molecular function | 12 | 171 | 434 | 15325 | 2.47796911633924 | 0.00349454587256982 | 0.121061053442597 | 2.45660925421056 | C3//CST3//RECK//A2M//SERPINA6//SERPINF1//TFPI2//WFDC1//CST4//TIMP3//GPC3//PI16 |
| GO:0030674 | protein binding, bridging | Molecular function | 10 | 130 | 434 | 15325 | 2.7162353775257 | 0.00388452324269808 | 0.129930605014384 | 2.41066227564582 | PIK3R1//ARHGAP6//SH2D1A//SHB//SORBS1//OPHN1//ANK2//ANXA1//COL19A1//COL14A1 |
| GO:0050431 | transforming growth factor beta binding | Molecular function | 3 | 12 | 434 | 15325 | 8.82776497695853 | 0.00410106224850484 | 0.132601012701657 | 2.38710363866741 | CD36//TGFBR2//TGFBR3 |
| GO:0005509 | calcium ion binding | Molecular function | 31 | 662 | 434 | 15325 | 1.65353905912818 | 0.00429542509528211 | 0.134405236852376 | 2.36699384990032 | ANXA1//CASQ2//CDH5//CETN2//DMD//FBLN1//EFEMP1//MAN1A1//MGP//PCDH9//PLA2G2A//PLCB4//ITSN1//SLIT3//TLL1//TTN//SPARCL1//AOC3//SLIT2//MYL9//FBLN5//CDH19//KCNIP1//PCDH18//PLSCR4//HEG1//SMOC2//ELTD1//FAT4//SVEP1//NCALD |
| GO:0038023 | signaling receptor activity | Molecular function | 54 | 1331 | 434 | 15325 | 1.43260498499102 | 0.00451426571800033 | 0.136838679576885 | 2.34541288113223 | KCNH1//CALCRL//LPAR1//NR4A2//PTGER3//RORB//NR2F1//TGFBR2//KDR//KIT//MUSK//ROR1//TEK//CD44//DARC//KLRB1//TRGC2//LYVE1//SLC22A17//CNTFR//IL6ST//LIFR//GHR//FRZB//SFRP1//FZD4//BAI3//GLP2R//GPR64//LPHN3//ELTD1//OR4F5//OR8J3//OR4A5//OR56B1//OR4F21//BDKRB1//CCRL1//PTGFR//EDNRB//PTPRD//PTPRS//EFEMP1//FGFR1//TGFBR3//EDA2R//COLEC12//ABCC9//GFRA1//CHRM2//FCER1A//CD160//NTRK2//CD36 |
| GO:0004872 | receptor activity | Molecular function | 60 | 1533 | 434 | 15325 | 1.38203756977824 | 0.0060491329762168 | 0.177807848088797 | 2.21830686842662 | KCNH1//CALCRL//LPAR1//NR4A2//PTGER3//RORB//NR2F1//TGFBR2//KDR//KIT//MUSK//ROR1//TEK//CD44//DARC//KLRB1//TRGC2//LYVE1//SLC22A17//CNTFR//IL6ST//LIFR//GHR//FRZB//SFRP1//FZD4//BAI3//GLP2R//GPR64//LPHN3//ELTD1//OR4F5//OR8J3//OR4A5//OR56B1//OR4F21//BDKRB1//CCRL1//PTGFR//EDNRB//PTPRD//PTPRS//EFEMP1//FGFR1//TGFBR3//EDA2R//CD36//CORIN//SCARA3//COLEC12//ABCC9//GFRA1//CHRM2//FCER1A//CD160//NTRK2//KLRG1//SEC63//NLGN1//SEMA3D |
| GO:0060090 | binding, bridging | Molecular function | 10 | 142 | 434 | 15325 | 2.48669435970663 | 0.00719379248237753 | 0.205234667879594 | 2.14304209432654 | ANK2//ANXA1//COL19A1//COL14A1//PIK3R1//ARHGAP6//SH2D1A//SHB//SORBS1//OPHN1 |
| GO:0005158 | insulin receptor binding | Molecular function | 4 | 28 | 434 | 15325 | 5.04443712969059 | 0.00757918596052617 | 0.210051725191725 | 2.12037743709042 | IGF1//PIK3R1//SORBS1//DOK5 |
| GO:0004896 | cytokine receptor activity | Molecular function | 7 | 82 | 434 | 15325 | 3.01435877261998 | 0.00853925647647811 | 0.230085521727327 | 2.06857994221736 | CNTFR//IL6ST//LIFR//GHR//DARC//CCRL1//GFRA1 |
| GO:0030246 | carbohydrate binding | Molecular function | 13 | 220 | 434 | 15325 | 2.08656263091747 | 0.0100658755039664 | 0.263889168617498 | 1.99714844531527 | PTX3//COLEC12//CLN5//DPM1//SELP//CD34//CNTN1//KLRB1//LGALS2//KLRG1//LPHN3//CHODL//FREM1 |
| GO:0005488 | binding | Molecular function | 358 | 11958 | 434 | 15325 | 1.05714663380202 | 0.0118264079028283 | 0.301884622782722 | 1.92714714589396 | CRYAB//SEC63//ERO1LB//PSTK//ECI2//TRIM9//KCNMA1//REV3L//VDAC2//RCAN2//DHRS7B//RBMS3//RBM11//RAVER2//LARP6//PPIL4//RDH10//RBPMS2//TSPYL2//A2M//ABCD2//ANK2//ANXA1//APOD//AQP1//BMX//C3//CACNB4//CAV1//CCNC//CD44//CDH5//CETN2//CFL2//CLU//CLN5//CST3//DES//DMD//RCAN1//ECH1//LPAR1//EPAS1//ERG//EFEMP1//FGF2//FGF7//FGFR1//FOXC1//FLNC//GEM//GHR//GPC3//CFH//IGF1//IL6ST//KCNH1//KDR//KIT//LSAMP//MAP1B//MEF2C//MEOX2//CD200//MUSK//MYH10//MYH11//PPP1R12A//NFATC2//ROR1//NR4A2//OGN//PBX1//PIK3R1//PLN//PMP22//MAPK4//PRNP//PTGIS//PTPRD//PTPRS//RNASE1//SELP//ITSN1//SNRPN//SOD3//TACC1//ZEB1//TEAD1//TEK//NR2F1//TGFBR2//TGFBR3//TIMP3//TNS1//TRPC1//TTN//WEE1//FZD4//SDPR//PPAP2B//MPDZ//DPM1//KSR1//FCGBP//DIRAS3//SLIT2//ITM2A//FHL5//AKAP12//CALCRL//SPRY1//DLC1//SPON1//FBLN5//SORBS1//SYNM//TENC1//KANK2//KCNMB4//CCDC59//KCNIP1//CDON//RAB9B//TAF9B//MPP6//ERRFI1//ZRANB1//ADI1//PARVA//PLSCR4//JAM2//EDA2R//FAT4//ARID5B//TSPYL5//BOC//MYOCD//OSBPL6//HSPB6//SH3D19//SYNPO2//NEGR1//CCDC141//PGM5//DYRK3//BEX1//CASQ2//FBLN1//MAN1A1//MGP//PCDH9//PLA2G2A//PLCB4//SLIT3//TLL1//SPARCL1//AOC3//MYL9//CDH19//PCDH18//HEG1//SMOC2//ELTD1//SVEP1//NCALD//BCHE//LGALS2//GNAL//PTX3//CNTN1//CCDC80//TIPARP//OSR1//ZNF300P1//EBF1//FOSB//SMAD9//NFIB//NFIX//PLAGL1//ZNF43//LHFP//BNC2//ZNF331//ZNF462//ZNF518B//FOXP2//ZFP3//ZNF280B//ZNF738//ZNF483//FAM171B//EBF3//GTF2H5//ZNF876P//ZNF826P//TOR1AIP1//AFF3//SSBP2//CNN1//CNN3//EPB41L2//OPHN1//TAGLN//TMOD1//TPM2//CAP2//IQGAP2//WASF3//DAAM2//LMOD1//TMOD2//KLHL4//COL19A1//COL14A1//NEXN//IGJ//CNTFR//GFRA1//LAMA2//MFAP4//CXCL12//ANGPTL1//CD160//CROT//CNTNAP3//CPE//VCAM1//NLGN1//CD34//KLRB1//KLRG1//LPHN3//CHODL//FREM1//LIFR//NTRK2//ARHGAP6//SH2D1A//SHB//FGF10//ANKS1B//SFRP1//SCG2//DOK5//ECM2//TNXB//PTHLH//RLN1//VIP//SERPINA6//ALDH1A1//PTGDS//FABP4//PMP2//AMPD1//ASPA//CYB5A//EYA4//F13A1//HTN3//ITGA9//MAT2A//PDE1A//ITGA8//PDE8B//PDE7B//SOBP//CACNA2D3//RIMKLB//CYBRD1//COLEC12//CYB5D1//PRUNE2//CH25H//C5ORF4//SCD5//MT1A//ABI3BP//PODN//IGFBP4//ACTA2//ACTC1//ACTG2//PDK4//PIK3C2A//NME5//PAPSS2//DCLK1//ABCC9//HIPK3//ABCA8//UBE2J1//NRK//POTEE//RHOJ//RERGL//RABL3//VIT//MAMDC2//LYVE1//SUSD5//FERMT2//ARHGEF26//VEPH1//PREX2//CD36//APOLD1//ANGPT1//CCL21//CAPN6//PTN//HDGFRP3//PDGFD//GPX3//CTSG//LIPG//ADAMTS1//CRISPLD2//RORB//PDLIM1//PDZRN4//ADAMTS9//ADAMTSL3//RNF150//RNF122//CPXM2//ZDHHC15//PRICKLE2//MSRB3//RNF180//SEPP1//RBP7//EDNRB//DNAJC15//DPYSL3//SH3BGRL2//LDB2//FRZB//APCDD1//FCER1A//SPDYA//PRKAR2B//DARC//PXDC1//BDKRB1//CMA1//OXCT1//CADM3//PLVAP//TCF21//GPC5//GPC6//CTNNAL1//FMO1//ANKRD42//CMAHP |
| GO:0070064 | proline-rich region binding | Molecular function | 3 | 18 | 434 | 15325 | 5.88517665130568 | 0.0134128381362515 | 0.333601358773435 | 1.87247931636936 | GHR//ITSN1//SH3D19 |
| GO:0005099 | Ras GTPase activator activity | Molecular function | 8 | 112 | 434 | 15325 | 2.52221856484529 | 0.0142608612411552 | 0.345825885098014 | 1.84585424580345 | TBC1D19//ARHGAP6//OPHN1//DLC1//ERRFI1//PREX2//ALDH1A1//IQGAP2 |
| GO:0004888 | transmembrane signaling receptor activity | Molecular function | 48 | 1235 | 434 | 15325 | 1.37241366443404 | 0.0153989875137259 | 0.364317509471076 | 1.81250783316726 | CALCRL//LPAR1//TGFBR2//KDR//KIT//MUSK//ROR1//TEK//CNTFR//IL6ST//LIFR//GHR//FRZB//SFRP1//FZD4//BAI3//GLP2R//GPR64//LPHN3//ELTD1//OR4F5//OR8J3//OR4A5//OR56B1//OR4F21//BDKRB1//DARC//CCRL1//PTGER3//PTGFR//EDNRB//PTPRD//PTPRS//EFEMP1//FGFR1//TGFBR3//EDA2R//ABCC9//GFRA1//CHRM2//FCER1A//CD160//NTRK2//CD44//KLRB1//TRGC2//LYVE1//SLC22A17 |
| GO:0005540 | hyaluronic acid binding | Molecular function | 3 | 20 | 434 | 15325 | 5.29665898617512 | 0.0179734505134495 | 0.381024358931821 | 1.745368539747 | CD44//LYVE1//SUSD5 |
| GO:0017134 | fibroblast growth factor binding | Molecular function | 3 | 20 | 434 | 15325 | 5.29665898617512 | 0.0179734505134495 | 0.381024358931821 | 1.745368539747 | FGF2//FGFR1//TGFBR3 |
| GO:0042813 | Wnt-activated receptor activity | Molecular function | 3 | 20 | 434 | 15325 | 5.29665898617512 | 0.0179734505134495 | 0.381024358931821 | 1.745368539747 | FRZB//SFRP1//FZD4 |
| GO:0050839 | cell adhesion molecule binding | Molecular function | 5 | 54 | 434 | 15325 | 3.26954258405871 | 0.0180543355163232 | 0.381024358931821 | 1.74341849100368 | CTNNAL1//CPE//PTPRD//VCAM1//NLGN1 |
| GO:0004866 | endopeptidase inhibitor activity | Molecular function | 10 | 164 | 434 | 15325 | 2.15311340901427 | 0.0184620050204078 | 0.381024358931821 | 1.73372113527037 | A2M//SERPINA6//SERPINF1//TFPI2//RECK//WFDC1//CST3//CST4//TIMP3//C3 |
| GO:0008083 | growth factor activity | Molecular function | 10 | 164 | 434 | 15325 | 2.15311340901427 | 0.0184620050204078 | 0.381024358931821 | 1.73372113527037 | EFEMP1//FGF2//FGF7//FGF10//IGF1//OGN//PTN//CXCL12//HDGFRP3//PDGFD |
| GO:0005515 | protein binding | Molecular function | 231 | 7396 | 434 | 15325 | 1.10287382892235 | 0.0201551514229863 | 0.407302018339515 | 1.69561393470168 | CRYAB//SEC63//ERO1LB//TRIM9//BEX1//GNAL//CNTN1//SELP//LPAR1//CCDC80//CST3//KIT//TTN//DMD//TOR1AIP1//PRNP//CFL2//CNN1//CNN3//EPB41L2//FLNC//KCNMA1//MYH10//MYH11//OPHN1//TAGLN//TMOD1//TNS1//TPM2//CAP2//SORBS1//IQGAP2//WASF3//DAAM2//LMOD1//TMOD2//PARVA//KLHL4//NCALD//SYNPO2//ANK2//ANXA1//COL19A1//COL14A1//NEXN//C3//CAV1//CDH5//CNTFR//ECH1//GFRA1//LAMA2//MFAP4//PTPRD//CXCL12//ANGPTL1//ECI2//CD160//CROT//CNTNAP3//CPE//VCAM1//NLGN1//PIK3R1//A2M//GHR//IL6ST//KDR//LIFR//NTRK2//ARHGAP6//SH2D1A//SHB//AKAP12//FGF2//FGF10//ANKS1B//SFRP1//TGFBR3//SCG2//EFEMP1//IGF1//DOK5//ECM2//TNXB//PPAP2B//FBLN5//PTHLH//RLN1//VIP//GEM//KCNH1//PDE1A//CD44//TLL1//ABI3BP//PODN//IGFBP4//ANGPT1//CD36//TGFBR2//CCL21//CAPN6//CETN2//MPDZ//FGF7//OGN//PTN//HDGFRP3//PDGFD//DES//CD34//EPAS1//FOXC1//FOSB//GPX3//PBX1//MYOCD//FOXP2//FGFR1//PLN//CLU//ACTC1//DNAJC15//DPYSL3//PLSCR4//SH3BGRL2//BCHE//CYB5A//LDB2//FRZB//ROR1//FZD4//APCDD1//SYNM//FCER1A//CCNC//PPP1R12A//MAPK4//ERRFI1//SPDYA//PRKAR2B//TACC1//DARC//RCAN2//RNF180//EDNRB//AMPD1//SOBP//ITSN1//MEF2C//SLIT3//SLIT2//DLC1//ABCD2//CASQ2//OXCT1//AOC3//CADM3//PLVAP//HSPB6//TCF21//TRPC1//ABCC9//GPC5//GPC3//CFH//GPC6//CTNNAL1//NR4A2//KCNIP1//TAF9B//JAM2//EBF1//ELTD1//EBF3//ANKRD42//SH3D19//ZRANB1//APOD//AQP1//BMX//CACNB4//CLN5//RCAN1//ERG//LSAMP//MAP1B//MEOX2//CD200//MUSK//NFATC2//PMP22//PTGIS//PTPRS//RNASE1//SNRPN//SOD3//ZEB1//TEAD1//TEK//NR2F1//TIMP3//VDAC2//WEE1//SDPR//DPM1//KSR1//FCGBP//DIRAS3//ITM2A//FHL5//CALCRL//SPRY1//SPON1//TENC1//KANK2//KCNMB4//CCDC59//CDON//RAB9B//MPP6//ADI1//EDA2R//FAT4//ARID5B//TSPYL5//BOC//OSBPL6//NEGR1//CCDC141 |
| GO:0061135 | endopeptidase regulator activity | Molecular function | 10 | 168 | 434 | 15325 | 2.10184880403774 | 0.0214415187236145 | 0.424454554324614 | 1.66874445638945 | C3//CST3//RECK//A2M//SERPINA6//SERPINF1//TFPI2//WFDC1//CST4//TIMP3 |
| GO:0005201 | extracellular matrix structural constituent | Molecular function | 6 | 78 | 434 | 15325 | 2.7162353775257 | 0.0231038784347743 | 0.442997317033721 | 1.63631510922323 | COL19A1//FBLN1//MGP//COL14A1//TFPI2//MFAP5 |
| GO:0005227 | calcium activated cation channel activity | Molecular function | 3 | 22 | 434 | 15325 | 4.81514453288647 | 0.0232916115141441 | 0.442997317033721 | 1.63280046219575 | KCNMA1//KCNMB4//ANO6 |
| GO:0032403 | protein complex binding | Molecular function | 16 | 328 | 434 | 15325 | 1.72249072721142 | 0.0249518207747823 | 0.465447425991131 | 1.60289775771238 | IGF1//PIK3R1//SORBS1//DOK5//ECM2//GFRA1//KDR//TNXB//VCAM1//PPAP2B//FBLN5//SYNM//FCER1A//CETN2//GNAL//TGFBR3 |
| GO:0005057 | receptor signaling protein activity | Molecular function | 7 | 103 | 434 | 15325 | 2.39978077043533 | 0.027004961185416 | 0.494241742450066 | 1.56855644259948 | TGFBR2//MAPK4//KDR//KIT//SMAD9//DCLK1//DOK5 |
| GO:0004114 | 3',5'-cyclic-nucleotide phosphodiesterase activity | Molecular function | 3 | 24 | 434 | 15325 | 4.41388248847926 | 0.0293696106805912 | 0.517973133821336 | 1.53210181038532 | PDE7B//PDE1A//PDE8B |
| GO:0004364 | glutathione transferase activity | Molecular function | 3 | 24 | 434 | 15325 | 4.41388248847926 | 0.0293696106805912 | 0.517973133821336 | 1.53210181038532 | GSTA2//GSTM5//GSTT2 |
| GO:0005198 | structural molecule activity | Molecular function | 26 | 624 | 434 | 15325 | 1.47129416282642 | 0.0321751419316084 | 0.556337379498688 | 1.49247952865113 | DES//DMD//SYNM//COL19A1//FBLN1//MGP//COL14A1//TFPI2//MFAP5//CRYAB//HSPB6//MYH11//TPM2//TTN//MYL9//NEXN//CAV1//ITSN1//ANXA1//EPB41L2//LAMA2//MAP1B//PGM5//CTNNAL1//BVES//KRT222 |
| GO:0004112 | cyclic-nucleotide phosphodiesterase activity | Molecular function | 3 | 25 | 434 | 15325 | 4.23732718894009 | 0.0326919903416755 | 0.556337379498688 | 1.48555863805856 | PDE8B//PDE7B//PDE1A |
| GO:0004955 | prostaglandin receptor activity | Molecular function | 2 | 11 | 434 | 15325 | 6.42019271051529 | 0.0371632275456304 | 0.600805511987691 | 1.42988657550756 | PTGER3//PTGFR |
| GO:0017166 | vinculin binding | Molecular function | 2 | 11 | 434 | 15325 | 6.42019271051529 | 0.0371632275456304 | 0.600805511987691 | 1.42988657550756 | DMD//SYNM |
| GO:0019956 | chemokine binding | Molecular function | 2 | 11 | 434 | 15325 | 6.42019271051529 | 0.0371632275456304 | 0.600805511987691 | 1.42988657550756 | DARC//A2M |
| GO:0035591 | signaling adaptor activity | Molecular function | 5 | 66 | 434 | 15325 | 2.67508029604804 | 0.0388692888719676 | 0.608116293642074 | 1.41039340487441 | PIK3R1//ARHGAP6//SH2D1A//SHB//SORBS1 |
| GO:0043178 | alcohol binding | Molecular function | 5 | 66 | 434 | 15325 | 2.67508029604804 | 0.0388692888719676 | 0.608116293642074 | 1.41039340487441 | APOD//CAV1//PMP2//TRPC1//DPM1 |
| GO:0005504 | fatty acid binding | Molecular function | 3 | 27 | 434 | 15325 | 3.92345110087046 | 0.0398964488489471 | 0.614278656880614 | 1.39906575879801 | FABP4//PMP2//PTGDS |
| GO:0005516 | calmodulin binding | Molecular function | 9 | 162 | 434 | 15325 | 1.96172555043523 | 0.0411816206516104 | 0.615396967926143 | 1.38529656626092 | CNN1//CNN3//GEM//KCNH1//MYH10//MYH11//PDE1A//TTN//IQGAP2 |
| GO:0004953 | icosanoid receptor activity | Molecular function | 2 | 12 | 434 | 15325 | 5.88517665130568 | 0.0437756606050555 | 0.615396967926143 | 1.35876729139036 | PTGER3//PTGFR |
| GO:0004954 | prostanoid receptor activity | Molecular function | 2 | 12 | 434 | 15325 | 5.88517665130568 | 0.0437756606050555 | 0.615396967926143 | 1.35876729139036 | PTGER3//PTGFR |
| GO:0008191 | metalloendopeptidase inhibitor activity | Molecular function | 2 | 12 | 434 | 15325 | 5.88517665130568 | 0.0437756606050555 | 0.615396967926143 | 1.35876729139036 | TIMP3//RECK |
| GO:0008430 | selenium binding | Molecular function | 2 | 12 | 434 | 15325 | 5.88517665130568 | 0.0437756606050555 | 0.615396967926143 | 1.35876729139036 | GPX3//SEPP1 |
| GO:0048551 | metalloenzyme inhibitor activity | Molecular function | 2 | 12 | 434 | 15325 | 5.88517665130568 | 0.0437756606050555 | 0.615396967926143 | 1.35876729139036 | TIMP3//RECK |
| GO:0030234 | enzyme regulator activity | Molecular function | 37 | 982 | 434 | 15325 | 1.33045745070251 | 0.0463123039364241 | 0.634404960641381 | 1.33430361326249 | PPP1R12A//PLN//PTN//PPP1R1C//C3//CST3//RECK//A2M//SERPINA6//SERPINF1//TFPI2//WFDC1//CST4//NRK//ITSN1//ARHGEF26//SLIT2//IQGAP2//RGS5//RGS22//ARHGAP28//TBC1D19//ALDH1A1//ARHGAP6//OPHN1//DLC1//ERRFI1//TIMP3//PRKAR2B//SPA17//CAV1//FBLN1//PI16//ANXA1//PREX2//PIK3R1//GPC3 |
| GO:0004879 | ligand-activated sequence-specific DNA binding RNA polymerase II transcription factor activity | Molecular function | 4 | 48 | 434 | 15325 | 2.94258832565284 | 0.0464358270160186 | 0.634404960641381 | 1.33314681531903 | NR4A2//PTGER3//RORB//NR2F1 |
| GO:0044325 | ion channel binding | Molecular function | 4 | 49 | 434 | 15325 | 2.88253550268033 | 0.0494787732394288 | 0.647780920650088 | 1.30558107666522 | ANK2//CDH5//TRPC1//ABCC9 |
