# Supplementary material for: Downregulation of miR-181b-5p Inhibits the Viability, Migration, and Glycolysis of Gallbladder Cancer by Upregulating PDHX Under Hypoxia
Source: Front Oncol. 2021 Aug 16;11:683725. doi: 10.3389/fonc.2021.683725 (PMC8415503; doi:10.3389/fonc.2021.683725)
Supplement: Supplementary file 3 [file DataSheet_1.zip › RNA seq raw data/HuGene 2.0 ST Data/GO Analysis/A vs B_down/CC_result(Human).html]

| GO.ID | Term | Ontology | Count | Pop.Hits | List.Total | Pop.Total | Fold.Enrichment | Pvalue | FDR | Enrichment.Score | GENES |
| --- | --- | --- | --- | --- | --- | --- | --- | --- | --- | --- | --- |
| GO:0044421 | extracellular region part | Cellular component | 84 | 1130 | 479 | 17068 | 2.648792654313 | 1.14821625624409e-16 | 6.65965428621572e-14 | 15.9399763087599 | MFAP4//MFAP5//COL19A1//COL14A1//SCARA3//COLEC12//DPT//ECM2//FBLN1//EFEMP1//GPC5//FMOD//GPC3//MGP//OMD//OGN//SFRP1//TGFBR3//TNXB//TFPI2//SPARCL1//CILP//GPC6//SPON1//FBLN5//ADAMTS9//ADAMTSL3//PODN//CST3//LAMA2//TIMP3//ADAMTS1//SMOC2//CCDC80//FREM1//VIT//ABI3BP//MAMDC2//A2M//ANGPT1//ANXA1//APOD//BCHE//C3//SERPINA6//CLU//CTSG//CFD//DPYSL3//FGF2//FGF10//FRZB//GFRA1//GHR//GPX3//CFH//IGF1//IGFBP4//IL6ST//KIT//SERPINF1//PLA2G2A//PTGDS//PTGIS//PTHLH//PTN//CCL21//CXCL12//SELP//SEPP1//SLIT3//SOD3//VCAM1//SCG2//ANGPTL1//SLIT2//LIPG//WFDC1//CPXM2//CBLN4//AQP1//CD34//ACTC1//CNTFR |
| GO:0031012 | extracellular matrix | Cellular component | 47 | 424 | 479 | 17068 | 3.94983653050774 | 6.99755926509915e-16 | 2.02929218687875e-13 | 15.1550534146344 | MFAP4//MFAP5//COL19A1//COL14A1//SCARA3//COLEC12//DPT//ECM2//FBLN1//EFEMP1//GPC5//FMOD//GPC3//MGP//OMD//OGN//SFRP1//TGFBR3//TNXB//TFPI2//SPARCL1//CILP//GPC6//SPON1//FBLN5//ADAMTS9//ADAMTSL3//PODN//CST3//LAMA2//TIMP3//ADAMTS1//SMOC2//CCDC80//FREM1//VIT//ABI3BP//MAMDC2//CLU//CMA1//CTSG//FGF10//SERPINF1//SOD3//CDON//CRISPLD2//CPXM2 |
| GO:0005576 | extracellular region | Cellular component | 123 | 2164 | 479 | 17068 | 2.02532617629921 | 4.11461856422437e-15 | 7.95492922416712e-13 | 14.3856704188067 | MFAP4//MFAP5//COL19A1//COL14A1//SCARA3//COLEC12//DPT//ECM2//FBLN1//EFEMP1//GPC5//FMOD//GPC3//MGP//OMD//OGN//SFRP1//TGFBR3//TNXB//TFPI2//SPARCL1//CILP//GPC6//SPON1//FBLN5//ADAMTS9//ADAMTSL3//PODN//CST3//LAMA2//TIMP3//ADAMTS1//SMOC2//CCDC80//FREM1//VIT//ABI3BP//MAMDC2//A2M//ANGPT1//ANXA1//APOD//BCHE//C3//SERPINA6//CLU//CTSG//CFD//DPYSL3//FGF2//FGF10//FRZB//GFRA1//GHR//GPX3//CFH//IGF1//IGFBP4//IL6ST//KIT//SERPINF1//PLA2G2A//PTGDS//PTGIS//PTHLH//PTN//CCL21//CXCL12//SELP//SEPP1//SLIT3//SOD3//VCAM1//SCG2//ANGPTL1//SLIT2//LIPG//WFDC1//CPXM2//CBLN4//AQP1//CD34//ACTC1//CNTFR//C7//CMA1//CPE//CST4//F13A1//FGF7//FGFR1//HTN3//IGJ//KDR//LIFR//PTGFR//PTX3//RLN1//RNASE1//TEK//TLL1//TTN//VIP//FCGBP//CORIN//MMRN1//CHRDL2//EMCN//RNLS//OLFML3//HEG1//CNTNAP3//SVEP1//PDGFD//APOLD1//CRISPLD1//CRISPLD2//CHRDL1//C16ORF89//PI16//SEMA3D//OLFML1//IGSF10 |
| GO:0005615 | extracellular space | Cellular component | 63 | 848 | 479 | 17068 | 2.64723086619136 | 1.30747175959161e-12 | 1.89583405140783e-10 | 11.8835676827941 | IGF1//CLU//CNTFR//A2M//ANGPT1//ANXA1//APOD//BCHE//C3//SERPINA6//CST3//CTSG//CFD//DPT//DPYSL3//FBLN1//EFEMP1//FGF2//FGF10//GPC5//FMOD//FRZB//GFRA1//GHR//GPC3//GPX3//CFH//IGFBP4//IL6ST//KIT//OGN//SERPINF1//PLA2G2A//PTGDS//PTGIS//PTHLH//PTN//CCL21//CXCL12//SELP//SEPP1//SFRP1//SLIT3//SOD3//TGFBR3//TNXB//COL14A1//VCAM1//SCG2//SPARCL1//CILP//ANGPTL1//SLIT2//LIPG//GPC6//SPON1//FBLN5//ABI3BP//ADAMTS9//WFDC1//CPXM2//PODN//CBLN4 |
| GO:0005578 | proteinaceous extracellular matrix | Cellular component | 38 | 362 | 479 | 17068 | 3.7404352991384 | 2.59005245513468e-12 | 3.00446084795623e-10 | 11.5866914402644 | MFAP4//MFAP5//COL19A1//COL14A1//SCARA3//COLEC12//TNXB//CST3//FBLN1//LAMA2//TIMP3//ADAMTS1//SMOC2//CCDC80//FREM1//ECM2//VIT//ABI3BP//MAMDC2//DPT//EFEMP1//GPC5//FMOD//GPC3//MGP//OMD//OGN//SFRP1//TGFBR3//TFPI2//SPARCL1//CILP//GPC6//SPON1//FBLN5//ADAMTS9//ADAMTSL3//PODN |
| GO:0071944 | cell periphery | Cellular component | 182 | 4400 | 479 | 17068 | 1.47389257923705 | 1.69106617917405e-09 | 1.59406687502853e-07 | 8.77183939613513 | ANGPT1//ANK2//ANXA1//AQP1//BAI3//BDKRB1//C3//CACNB4//CAV1//CD34//CD36//CD44//CDH5//CHRM2//CNTFR//CNTN1//CPE//CRYAB//CTSG//DMD//LPAR1//EDNRB//EMP1//EPB41L2//FCER1A//FGF10//FGFR1//GPC5//FLNC//DARC//GHR//GPC3//GNAL//GNG7//GNG11//IL6ST//ITGA9//KCNH1//KCNMA1//KDR//KIT//KLRB1//LSAMP//MAP1B//CD200//MYH10//NCAM2//NFATC2//ROR1//CNTN3//PCDH9//PIK3C2A//PIK3R1//FXYD1//PMP22//PRKAR2B//PRNP//PTGER3//PTGFR//SELP//SFRP1//SGCD//ITSN1//SHB//SLCO2A1//TEK//TGFBR2//TRPC1//VCAM1//FZD4//RECK//SDPR//RGS5//ITGA8//PPAP2A//PPAP2B//AOC3//CTNNAL1//DIRAS3//GLP2R//SLIT2//AKAP12//ABCC9//GPC6//CALCRL//KLRG1//SPRY1//ABCA8//CAP2//SORBS1//CORIN//LYVE1//CD160//BVES//FAIM2//LPHN3//TENC1//FRRS1L//PARM1//RGS22//KCNMB4//CDH19//KCNIP1//CDON//RAB9B//NRN1//CCRL1//MPP6//EMCN//CHIC1//PCDH18//EXOC6//BNC2//ADI1//PARVA//ANKS1B//PLSCR4//SLC44A2//RHOJ//CADM3//JAM2//TSPYL2//ELTD1//OR4F5//FAT4//VEPH1//CLMP//CYBRD1//CNTNAP3//OR8J3//OR4A5//APOLD1//TMEM47//BOC//OSBPL6//KCTD12//RFTN2//SH3D19//ANO6//VSTM4//CADM2//CCDC110//NEGR1//OR56B1//OR4F21//SCN7A//LIFR//MUSK//NTRK2//PTPRD//PTPRS//TRGC2//TGFBR3//TSPAN7//SLC16A4//DCLK1//GPR64//NLGN1//SLC22A17//EDA2R//APCDD1//C7//CASQ2//PTGIS//DLC1//PLVAP//FERMT2//GFRA1//CXCL12//HEG1//GEM//PGM5//PTP4A1//MPDZ//SLC5A12//PRICKLE2//TMOD1//ERRFI1//BMX//DES//LAMA2//ACTG2 |
| GO:0005886 | plasma membrane | Cellular component | 179 | 4312 | 479 | 17068 | 1.47918127205333 | 1.9238738146896e-09 | 1.59406687502853e-07 | 8.71582341638405 | GNAL//GNG7//GNG11//SCN7A//CD160//ANK2//AQP1//BDKRB1//CAV1//CD34//CD36//CD44//CHRM2//LPAR1//EDNRB//FCER1A//FGFR1//GPC5//GHR//GPC3//KDR//LIFR//CD200//MUSK//NTRK2//ROR1//FXYD1//PTGER3//PTGFR//PTPRD//PTPRS//SELP//SLCO2A1//TRGC2//TEK//TGFBR3//TSPAN7//TRPC1//FZD4//PPAP2A//SLC16A4//DCLK1//GPC6//GPR64//CALCRL//CORIN//LYVE1//NLGN1//KCNMB4//SLC22A17//CCRL1//JAM2//EDA2R//APCDD1//C7//ITGA9//ITGA8//CACNB4//CASQ2//IL6ST//SORBS1//KCNMA1//PTGIS//TGFBR2//SDPR//DLC1//PLVAP//KCNH1//ABCC9//GFRA1//KIT//CXCL12//VCAM1//HEG1//GEM//PGM5//PTP4A1//DMD//SGCD//ANXA1//MPDZ//SLC5A12//PRICKLE2//BVES//ERRFI1//CYBRD1//BMX//CADM2//DES//FLNC//LAMA2//ANGPT1//BAI3//C3//CDH5//CNTFR//CNTN1//CPE//CRYAB//CTSG//EMP1//EPB41L2//FGF10//DARC//KLRB1//LSAMP//MAP1B//MYH10//NCAM2//NFATC2//CNTN3//PCDH9//PIK3C2A//PIK3R1//PMP22//PRKAR2B//PRNP//SFRP1//ITSN1//SHB//RECK//RGS5//PPAP2B//AOC3//CTNNAL1//DIRAS3//GLP2R//SLIT2//AKAP12//KLRG1//SPRY1//ABCA8//CAP2//FAIM2//LPHN3//TENC1//FRRS1L//PARM1//RGS22//CDH19//KCNIP1//CDON//RAB9B//NRN1//MPP6//EMCN//CHIC1//PCDH18//EXOC6//BNC2//ADI1//PARVA//ANKS1B//PLSCR4//SLC44A2//RHOJ//CADM3//TSPYL2//ELTD1//OR4F5//FAT4//VEPH1//CLMP//CNTNAP3//OR8J3//OR4A5//APOLD1//TMEM47//BOC//OSBPL6//KCTD12//RFTN2//SH3D19//ANO6//VSTM4//CCDC110//NEGR1//OR56B1//OR4F21 |
| GO:0043292 | contractile fiber | Cellular component | 22 | 176 | 479 | 17068 | 4.45407098121086 | 4.68669158226566e-09 | 3.3978513971426e-07 | 8.32913362521401 | MYH11//MYL9//TPM2//TTN//TMOD1//ACTC1//TRPC1//ABCC9//ANK2//CASQ2//CRYAB//DES//DMD//FLNC//PGM5//PARVA//NEXN//SYNPO2//ACTA2//SYNM//CST3//PPP1R12A |
| GO:0044449 | contractile fiber part | Cellular component | 19 | 159 | 479 | 17068 | 4.25797980593742 | 1.09802065099425e-07 | 7.07613308418517e-06 | 6.95938949182718 | MYH11//MYL9//TPM2//TTN//ACTC1//TRPC1//ABCC9//ANK2//CASQ2//CRYAB//DES//DMD//FLNC//PGM5//PARVA//NEXN//SYNPO2//ACTA2//SYNM |
| GO:0030016 | myofibril | Cellular component | 18 | 166 | 479 | 17068 | 3.86377241743592 | 1.0145176500302e-06 | 5.88420237017516e-05 | 5.99374039294405 | TPM2//TTN//ACTC1//TRPC1//ABCC9//ANK2//CASQ2//CRYAB//DES//DMD//FLNC//PGM5//MYL9//PARVA//NEXN//SYNPO2//SYNM//TMOD1 |
| GO:0005614 | interstitial matrix | Cellular component | 6 | 14 | 479 | 17068 | 15.2711005070086 | 1.17431262730892e-06 | 6.19183021671976e-05 | 5.93021626915246 | ECM2//VIT//ABI3BP//SMOC2//CCDC80//MAMDC2 |
| GO:0044459 | plasma membrane part | Cellular component | 88 | 1904 | 479 | 17068 | 1.64688338801074 | 1.48401957070299e-06 | 7.17276125839779e-05 | 5.82856037170371 | GNAL//GNG7//GNG11//SCN7A//CD160//ANK2//AQP1//BDKRB1//CAV1//CD34//CD36//CD44//CHRM2//LPAR1//EDNRB//FCER1A//FGFR1//GPC5//GHR//GPC3//KDR//LIFR//CD200//MUSK//NTRK2//ROR1//FXYD1//PTGER3//PTGFR//PTPRD//PTPRS//SELP//SLCO2A1//TRGC2//TEK//TGFBR3//TSPAN7//TRPC1//FZD4//PPAP2A//SLC16A4//DCLK1//GPC6//GPR64//CALCRL//CORIN//LYVE1//NLGN1//KCNMB4//SLC22A17//CCRL1//JAM2//EDA2R//APCDD1//C7//ITGA9//ITGA8//CACNB4//CASQ2//IL6ST//SORBS1//KCNMA1//PTGIS//TGFBR2//SDPR//DLC1//PLVAP//KCNH1//ABCC9//GFRA1//KIT//CXCL12//VCAM1//HEG1//GEM//PGM5//PTP4A1//DMD//SGCD//ANXA1//MPDZ//SLC5A12//PRICKLE2//BVES//ERRFI1//CYBRD1//BMX//CADM2 |
| GO:0031674 | I band | Cellular component | 13 | 96 | 479 | 17068 | 4.82524356297843 | 2.75283428504123e-06 | 0.000119790295524821 | 5.56021993145386 | ANK2//CASQ2//CRYAB//DES//DMD//FLNC//PGM5//TTN//MYL9//PARVA//NEXN//SYNPO2//ACTC1 |
| GO:0030017 | sarcomere | Cellular component | 16 | 145 | 479 | 17068 | 3.93186955582751 | 3.20654685935961e-06 | 0.000119790295524821 | 5.49396240907199 | TPM2//TTN//ANK2//CASQ2//CRYAB//DES//DMD//FLNC//PGM5//MYL9//PARVA//NEXN//SYNPO2//ACTC1//TRPC1//ABCC9 |
| GO:0045121 | membrane raft | Cellular component | 18 | 180 | 479 | 17068 | 3.56325678496868 | 3.27884101319552e-06 | 0.000119790295524821 | 5.48427964120372 | CAV1//KCNMA1//PTGIS//TGFBR2//SDPR//DLC1//PLVAP//ANGPT1//CD36//DMD//EDNRB//KDR//PRKAR2B//PRNP//TEK//TRPC1//SORBS1//FAIM2 |
| GO:0030018 | Z disc | Cellular component | 12 | 83 | 479 | 17068 | 5.15169655658123 | 3.30455987654678e-06 | 0.000119790295524821 | 5.48088637457937 | ANK2//CASQ2//CRYAB//DES//DMD//FLNC//PGM5//TTN//MYL9//PARVA//NEXN//SYNPO2 |
| GO:0043034 | costamere | Cellular component | 6 | 17 | 479 | 17068 | 12.5762004175365 | 4.50410673176361e-06 | 0.000153669523789582 | 5.34639132675645 | ANK2//DMD//FLNC//PGM5//TRPC1//SYNM |
| GO:0005887 | integral to plasma membrane | Cellular component | 59 | 1216 | 479 | 17068 | 1.72888281507527 | 2.57732812583629e-05 | 0.000807368728103775 | 4.5888302868552 | SCN7A//C7//ITGA9//ITGA8//IL6ST//SORBS1//KCNH1//KCNMA1//ABCC9//KCNMB4//NLGN1//TGFBR2//ANK2//AQP1//BDKRB1//CAV1//CD34//CD36//CD44//CHRM2//LPAR1//EDNRB//FCER1A//FGFR1//GPC5//GHR//GPC3//KDR//LIFR//CD200//MUSK//NTRK2//ROR1//FXYD1//PTGER3//PTGFR//PTPRD//PTPRS//SELP//SLCO2A1//TRGC2//TEK//TGFBR3//TSPAN7//TRPC1//FZD4//PPAP2A//SLC16A4//DCLK1//GPC6//GPR64//CALCRL//CORIN//LYVE1//SLC22A17//CCRL1//JAM2//EDA2R//APCDD1 |
| GO:0032432 | actin filament bundle | Cellular component | 8 | 44 | 479 | 17068 | 6.47864869994306 | 2.64482859206409e-05 | 0.000807368728103775 | 4.57760246878301 | MYH10//MYH11//PGM5//TEK//MYL9//SORBS1//FERMT2//CRYAB |
| GO:0031226 | intrinsic to plasma membrane | Cellular component | 60 | 1260 | 479 | 17068 | 1.69678894522318 | 3.77513918532018e-05 | 0.00109479036374285 | 4.42306703176931 | SCN7A//CD160//ANK2//AQP1//BDKRB1//CAV1//CD34//CD36//CD44//CHRM2//LPAR1//EDNRB//FCER1A//FGFR1//GPC5//GHR//GPC3//KDR//LIFR//CD200//MUSK//NTRK2//ROR1//FXYD1//PTGER3//PTGFR//PTPRD//PTPRS//SELP//SLCO2A1//TRGC2//TEK//TGFBR3//TSPAN7//TRPC1//FZD4//PPAP2A//SLC16A4//DCLK1//GPC6//GPR64//CALCRL//CORIN//LYVE1//NLGN1//KCNMB4//SLC22A17//CCRL1//JAM2//EDA2R//APCDD1//C7//ITGA9//ITGA8//IL6ST//SORBS1//KCNH1//KCNMA1//ABCC9//TGFBR2 |
| GO:0044420 | extracellular matrix part | Cellular component | 16 | 178 | 479 | 17068 | 3.20292744716286 | 4.3084367453429e-05 | 0.0011899491963328 | 4.36568027880062 | MFAP4//MFAP5//COL19A1//COL14A1//SCARA3//COLEC12//TNXB//CST3//FBLN1//LAMA2//TIMP3//ADAMTS1//SMOC2//CCDC80//FREM1//FBLN5 |
| GO:0016020 | membrane | Cellular component | 263 | 7856 | 479 | 17068 | 1.1928927373304 | 4.79790286784285e-05 | 0.00126490166515857 | 4.31894854844548 | ANGPT1//ANK2//ANXA1//AQP1//BAI3//BDKRB1//C3//CACNB4//CAV1//CD34//CD36//CD44//CDH5//CHRM2//CNTFR//CNTN1//CPE//CRYAB//CTSG//DMD//LPAR1//EDNRB//EMP1//EPB41L2//FCER1A//FGF10//FGFR1//GPC5//FLNC//DARC//GHR//GPC3//GNAL//GNG7//GNG11//IL6ST//ITGA9//KCNH1//KCNMA1//KDR//KIT//KLRB1//LSAMP//MAP1B//CD200//MYH10//NCAM2//NFATC2//ROR1//CNTN3//PCDH9//PIK3C2A//PIK3R1//FXYD1//PMP22//PRKAR2B//PRNP//PTGER3//PTGFR//SELP//SFRP1//SGCD//ITSN1//SHB//SLCO2A1//TEK//TGFBR2//TRPC1//VCAM1//FZD4//RECK//SDPR//RGS5//ITGA8//PPAP2A//PPAP2B//AOC3//CTNNAL1//DIRAS3//GLP2R//SLIT2//AKAP12//ABCC9//GPC6//CALCRL//KLRG1//SPRY1//ABCA8//CAP2//SORBS1//CORIN//LYVE1//CD160//BVES//FAIM2//LPHN3//TENC1//FRRS1L//PARM1//RGS22//KCNMB4//CDH19//KCNIP1//CDON//RAB9B//NRN1//CCRL1//MPP6//EMCN//CHIC1//PCDH18//EXOC6//BNC2//ADI1//PARVA//ANKS1B//PLSCR4//SLC44A2//RHOJ//CADM3//JAM2//TSPYL2//ELTD1//OR4F5//FAT4//VEPH1//CLMP//CYBRD1//CNTNAP3//OR8J3//OR4A5//APOLD1//TMEM47//BOC//OSBPL6//KCTD12//RFTN2//SH3D19//ANO6//VSTM4//CADM2//CCDC110//NEGR1//OR56B1//OR4F21//MAN1A1//UGCG//B3GALT2//UST//GCNT4//SCARA3//PDGFD//ABCD2//CLN5//CYB5A//EPHX1//FMO1//PLN//PTGIS//CH25H//PTPLA//ITM2A//LPPR4//GPR64//LHFP//PEMT//C5ORF4//SEC63//DHRS7B//SUSD5//TOR1AIP1//DNAJC15//ERGIC2//UBE2J1//TMEM100//CACNA2D3//RNF150//HEG1//GPAM//CACHD1//EDA2R//LRRC19//RNF122//SCD5//COLEC12//GLT8D2//PLVAP//TMTC1//TSPAN18//TMEM88//CYYR1//TM4SF18//C4ORF32//CHODL//MFSD4//RDH10//FREM1//ZDHHC15//SLC5A12//TMTC2//FAM171B//FAM171A1//FAM162B//PI16//KIAA1324L//SLC9A9//RNF180//TMEM150C//GFRA1//SCN7A//LIFR//MUSK//NTRK2//PTPRD//PTPRS//TRGC2//TGFBR3//TSPAN7//SLC16A4//DCLK1//NLGN1//SLC22A17//APCDD1//C7//VDAC2//GATM//PDK4//GSTM5//PLA2G2A//DPM1//KSR1//ERO1LB//CASQ2//DLC1//CXCL12//GEM//PGM5//PTP4A1//MPDZ//PRICKLE2//NCALD//ST6GALNAC3//ERRFI1//CST3//PTGDS//CLU//BMX//DES//LAMA2//BCHE//FRZB//TMOD1//SRPX//FILIP1L//SYNM//LMOD1//METTL7A//SPA17//PALMD//RERGL//SVEP1//SEMA3D//MAMDC2 |
| GO:0009986 | cell surface | Cellular component | 30 | 488 | 479 | 17068 | 2.19052671207091 | 5.30131909249649e-05 | 0.00133685437984694 | 4.27561605430221 | CD34//CD36//CD44//FCER1A//GFRA1//IL6ST//KCNMA1//KDR//KIT//CXCL12//SELP//TGFBR2//TGFBR3//VCAM1//HEG1//CRYAB//CTSG//DMD//LPAR1//FGF10//GHR//NTRK2//SFRP1//TEK//FZD4//SRPX//AOC3//SLIT2//CORIN//NLGN1 |
| GO:0042641 | actomyosin | Cellular component | 8 | 51 | 479 | 17068 | 5.58942240779401 | 8.00123670830896e-05 | 0.001933632204508 | 4.09684288124754 | MYH10//MYH11//PGM5//TEK//MYL9//SORBS1//FERMT2//ACTC1 |
| GO:0042383 | sarcolemma | Cellular component | 10 | 83 | 479 | 17068 | 4.29308046381769 | 0.000107255274234712 | 0.00248812364372933 | 3.96958134236271 | ANK2//ANXA1//AQP1//DES//DMD//FLNC//LAMA2//PGM5//SGCD//VCAM1 |
| GO:0015629 | actin cytoskeleton | Cellular component | 24 | 366 | 479 | 17068 | 2.33656182620897 | 0.000111536577132694 | 0.00248812364372933 | 3.95258268739684 | MYH10//MYH11//PGM5//TEK//MYL9//SORBS1//FERMT2//VCAM1//TPM2//TTN//ACTC1//ARHGAP6//EPB41L2//FILIP1L//DPYSL3//CRYAB//ACTA2//DMD//NFATC2//OPHN1//CTNNAL1//IQGAP2//PARVA//SYNPO2 |
| GO:0001725 | stress fiber | Cellular component | 7 | 41 | 479 | 17068 | 6.08360914506849 | 0.000129019116467128 | 0.00277152176114571 | 3.88934593651393 | MYH10//MYH11//PGM5//TEK//MYL9//SORBS1//FERMT2 |
| GO:0030054 | cell junction | Cellular component | 39 | 759 | 479 | 17068 | 1.83092245867956 | 0.000208246372567188 | 0.00431367486032032 | 3.68142255480845 | CDH5//TEK//FZD4//HEG1//CADM3//TMEM47//PPAP2B//SYNM//PGM5//SORBS1//DES//PMP22//MPDZ//BVES//JAM2//CLMP//NEXN//TNS1//DLC1//FERMT2//TENC1//PARVA//ANK2//DMD//CHRM2//EPB41L2//MAP1B//MUSK//OPHN1//ITSN1//NLGN1//FAIM2//FRRS1L//NRN1//ANKS1B//TRIM9//KCTD12//CBLN4//CADM2 |
| GO:0031225 | anchored to membrane | Cellular component | 13 | 146 | 479 | 17068 | 3.17276289072554 | 0.000242985560046549 | 0.00485971120093098 | 3.61441953454341 | CD160//CNTFR//CNTN1//GPC5//GFRA1//GPC3//LSAMP//CNTN3//PRNP//RECK//GPC6//NRN1//NEGR1 |
| GO:0031091 | platelet alpha granule | Cellular component | 8 | 60 | 479 | 17068 | 4.75100904662491 | 0.000257887659624721 | 0.00498582808607794 | 3.58856943911493 | CD36//SELP//A2M//CLU//CFD//F13A1//IGF1//MMRN1 |
| GO:0009897 | external side of plasma membrane | Cellular component | 15 | 202 | 479 | 17068 | 2.64598276111536 | 0.0005978131630464 | 0.0111848914376423 | 3.22343452660775 | CD34//CD36//CD44//FCER1A//GFRA1//IL6ST//KCNMA1//KDR//KIT//CXCL12//SELP//TGFBR2//TGFBR3//VCAM1//HEG1 |
| GO:0005901 | caveola | Cellular component | 7 | 57 | 479 | 17068 | 4.37592938504926 | 0.00103157799570857 | 0.0186973511722178 | 2.9864979302451 | CAV1//KCNMA1//PTGIS//TGFBR2//SDPR//DLC1//PLVAP |
| GO:0031093 | platelet alpha granule lumen | Cellular component | 6 | 47 | 479 | 17068 | 4.54883844889619 | 0.00191677352880904 | 0.0330344741792879 | 2.71742919698045 | A2M//CLU//CFD//F13A1//IGF1//MMRN1 |
| GO:0043197 | dendritic spine | Cellular component | 11 | 140 | 479 | 17068 | 2.79970175961825 | 0.00199345964875013 | 0.0330344741792879 | 2.70039255082302 | CNN3//NTRK2//PLCB4//MPDZ//NLGN1//ANKS1B//LPAR1//MAP1B//MYH10//OPHN1//PALMD |
| GO:0044309 | neuron spine | Cellular component | 11 | 140 | 479 | 17068 | 2.79970175961825 | 0.00199345964875013 | 0.0330344741792879 | 2.70039255082302 | CNN3//NTRK2//PLCB4//MPDZ//NLGN1//ANKS1B//LPAR1//MAP1B//MYH10//OPHN1//PALMD |
| GO:0034774 | secretory granule lumen | Cellular component | 6 | 50 | 479 | 17068 | 4.27590814196242 | 0.00264333242601172 | 0.0425870224190777 | 2.57784821647325 | A2M//CLU//CFD//F13A1//IGF1//MMRN1 |
| GO:0005604 | basement membrane | Cellular component | 8 | 86 | 479 | 17068 | 3.31465747438947 | 0.00283989578356122 | 0.0445172852558245 | 2.54669759708492 | LAMA2//CST3//FBLN1//TIMP3//ADAMTS1//SMOC2//CCDC80//FREM1 |
| GO:0044425 | membrane part | Cellular component | 202 | 6172 | 479 | 17068 | 1.16619875334361 | 0.00341408730390849 | 0.0521097535859717 | 2.4667253774448 | GNAL//GNG7//GNG11//ABCD2//BAI3//CDH5//CLN5//CYB5A//EMP1//EPHX1//FGFR1//FMO1//DARC//GHR//KCNMA1//KIT//KLRB1//MAN1A1//NCAM2//PCDH9//PLN//PMP22//PTGER3//PTGIS//SGCD//TGFBR2//UGCG//VCAM1//PPAP2B//AOC3//B3GALT2//CH25H//PTPLA//GLP2R//ITM2A//LPPR4//UST//GPR64//LHFP//KLRG1//ABCA8//PEMT//CORIN//C5ORF4//BVES//SEC63//FAIM2//LPHN3//FRRS1L//PARM1//DHRS7B//SUSD5//TOR1AIP1//CDH19//DNAJC15//CDON//ERGIC2//GCNT4//SCARA3//UBE2J1//EMCN//PCDH18//TMEM100//CACNA2D3//PLSCR4//SLC44A2//RNF150//HEG1//GPAM//CACHD1//CADM3//EDA2R//ELTD1//LRRC19//OR4F5//FAT4//CLMP//RNF122//CYBRD1//CNTNAP3//SCD5//COLEC12//OR8J3//OR4A5//APOLD1//GLT8D2//PLVAP//TMEM47//TMTC1//TSPAN18//BOC//TMEM88//CYYR1//TM4SF18//C4ORF32//CHODL//MFSD4//RDH10//FREM1//ZDHHC15//SLC5A12//TMTC2//FAM171B//VSTM4//FAM171A1//FAM162B//PI16//KIAA1324L//CADM2//SLC9A9//RNF180//OR56B1//TMEM150C//OR4F21//CNTFR//EPB41L2//GFRA1//PRNP//SCN7A//CD160//ANK2//AQP1//BDKRB1//CAV1//CD34//CD36//CD44//CHRM2//LPAR1//EDNRB//FCER1A//GPC5//GPC3//KDR//LIFR//CD200//MUSK//NTRK2//ROR1//FXYD1//PTGFR//PTPRD//PTPRS//SELP//SLCO2A1//TRGC2//TEK//TGFBR3//TSPAN7//TRPC1//FZD4//PPAP2A//SLC16A4//DCLK1//GPC6//CALCRL//LYVE1//NLGN1//KCNMB4//SLC22A17//CCRL1//JAM2//APCDD1//C7//ITGA9//ITGA8//GSTM5//PLA2G2A//DPM1//KSR1//ERO1LB//CACNB4//CASQ2//IL6ST//SORBS1//SDPR//DLC1//ITSN1//KCNH1//ABCC9//CXCL12//GEM//PGM5//PTP4A1//CNTN1//LSAMP//CNTN3//RECK//NRN1//NEGR1//VDAC2//DMD//ANXA1//MPDZ//PRICKLE2//NCALD//ST6GALNAC3//ERRFI1//BMX//ANO6//ANGPT1//PRKAR2B |
| GO:0060205 | cytoplasmic membrane-bounded vesicle lumen | Cellular component | 6 | 54 | 479 | 17068 | 3.95917420552076 | 0.00391106773111762 | 0.0581645970268774 | 2.40770466295456 | A2M//CLU//CFD//F13A1//IGF1//MMRN1 |
| GO:0031983 | vesicle lumen | Cellular component | 6 | 55 | 479 | 17068 | 3.88718921996584 | 0.00428824592388823 | 0.0621795658963793 | 2.36772031652306 | A2M//CLU//CFD//F13A1//IGF1//MMRN1 |
| GO:0005775 | vacuolar lumen | Cellular component | 7 | 75 | 479 | 17068 | 3.32570633263744 | 0.00503429131795053 | 0.0712168040100319 | 2.29806165686121 | GPC5//FMOD//GPC3//OMD//OGN//GPC6//CLN5 |
| GO:0009925 | basal plasma membrane | Cellular component | 4 | 26 | 479 | 17068 | 5.48193351533644 | 0.00560930818552229 | 0.0774618749429269 | 2.25109069841179 | AQP1//CD34//ITGA9//TEK |
| GO:0030055 | cell-substrate junction | Cellular component | 10 | 139 | 479 | 17068 | 2.56349408990553 | 0.00586582733417937 | 0.079120461716838 | 2.23167072500988 | SORBS1//NEXN//PGM5//TEK//TNS1//DLC1//FERMT2//TENC1//PARVA//DMD |
| GO:0045202 | synapse | Cellular component | 24 | 493 | 479 | 17068 | 1.73464833345332 | 0.00654788203896771 | 0.0863129905136653 | 2.18389915306689 | TRIM9//CNN3//NTRK2//PLCB4//MPDZ//NLGN1//ANKS1B//DES//MUSK//MYH10//KCTD12//KCNMA1//OPHN1//ANK2//CHRM2//DMD//FAIM2//CACNB4//MAP1B//ITSN1//FRRS1L//NRN1//CBLN4//CADM2 |
| GO:0009898 | internal side of plasma membrane | Cellular component | 8 | 102 | 479 | 17068 | 2.79471120389701 | 0.00797604060528481 | 0.102802301134782 | 2.09821264373774 | GNAL//GNG7//GNG11//ERRFI1//CACNB4//GEM//PGM5//PTP4A1 |
| GO:0097458 | neuron part | Cellular component | 32 | 729 | 479 | 17068 | 1.56411820465018 | 0.00848696451723866 | 0.107009552608661 | 2.07124761354146 | CNN3//NTRK2//PLCB4//MPDZ//NLGN1//ANKS1B//BDKRB1//ITSN1//GFRA1//MYH10//NCAM2//CADM2//APOD//IL6ST//KCNIP1//TTLL7//TRIM9//DPYSL3//TMOD2//CST3//KCTD12//GHR//VIP//BOC//KCNMA1//OPHN1//LPAR1//MAP1B//PALMD//NFIB//DMD//SYNM |
| GO:0005912 | adherens junction | Cellular component | 12 | 197 | 479 | 17068 | 2.17051174718905 | 0.00986352907049758 | 0.121720145976353 | 2.00596767110075 | PGM5//SORBS1//DES//NEXN//TEK//TNS1//DLC1//FERMT2//TENC1//PARVA//PPAP2B//SYNM |
| GO:0031224 | intrinsic to membrane | Cellular component | 177 | 5455 | 479 | 17068 | 1.15618047834914 | 0.0106062839377894 | 0.125786282118715 | 1.97443675068865 | ABCD2//BAI3//CDH5//CLN5//CYB5A//EMP1//EPHX1//FGFR1//FMO1//DARC//GHR//KCNMA1//KIT//KLRB1//MAN1A1//NCAM2//PCDH9//PLN//PMP22//PTGER3//PTGIS//SGCD//TGFBR2//UGCG//VCAM1//PPAP2B//AOC3//B3GALT2//CH25H//PTPLA//GLP2R//ITM2A//LPPR4//UST//GPR64//LHFP//KLRG1//ABCA8//PEMT//CORIN//C5ORF4//BVES//SEC63//FAIM2//LPHN3//FRRS1L//PARM1//DHRS7B//SUSD5//TOR1AIP1//CDH19//DNAJC15//CDON//ERGIC2//GCNT4//SCARA3//UBE2J1//EMCN//PCDH18//TMEM100//CACNA2D3//PLSCR4//SLC44A2//RNF150//HEG1//GPAM//CACHD1//CADM3//EDA2R//ELTD1//LRRC19//OR4F5//FAT4//CLMP//RNF122//CYBRD1//CNTNAP3//SCD5//COLEC12//OR8J3//OR4A5//APOLD1//GLT8D2//PLVAP//TMEM47//TMTC1//TSPAN18//BOC//TMEM88//CYYR1//TM4SF18//C4ORF32//CHODL//MFSD4//RDH10//FREM1//ZDHHC15//SLC5A12//TMTC2//FAM171B//VSTM4//FAM171A1//FAM162B//PI16//KIAA1324L//CADM2//SLC9A9//RNF180//OR56B1//TMEM150C//OR4F21//SCN7A//CD160//ANK2//AQP1//BDKRB1//CAV1//CD34//CD36//CD44//CHRM2//LPAR1//EDNRB//FCER1A//GPC5//GPC3//KDR//LIFR//CD200//MUSK//NTRK2//ROR1//FXYD1//PTGFR//PTPRD//PTPRS//SELP//SLCO2A1//TRGC2//TEK//TGFBR3//TSPAN7//TRPC1//FZD4//PPAP2A//SLC16A4//DCLK1//GPC6//CALCRL//LYVE1//NLGN1//KCNMB4//SLC22A17//CCRL1//JAM2//APCDD1//C7//ITGA9//ITGA8//CACNB4//CASQ2//IL6ST//SORBS1//KCNH1//ABCC9//CNTFR//CNTN1//GFRA1//LSAMP//CNTN3//PRNP//RECK//NRN1//NEGR1//VDAC2//ST6GALNAC3//ANO6 |
| GO:0005783 | endoplasmic reticulum | Cellular component | 51 | 1309 | 479 | 17068 | 1.38828186427351 | 0.0106267721100294 | 0.125786282118715 | 1.97359863271923 | BCHE//COL19A1//FMO1//COL14A1//GLT25D2//PDGFD//KDELC2//CYB5A//EPHX1//GSTM5//PLA2G2A//PTGIS//DPM1//KSR1//CH25H//PTPLA//PEMT//SEC63//ERGIC2//SCARA3//UBE2J1//ERO1LB//SCD5//RDH10//PLCB4//PTGDS//CASQ2//PLN//RNF180//CAV1//OSBPL6//APOD//BDKRB1//CLU//CLN5//CST3//KCNMA1//MAN1A1//PRNP//PTN//TGFBR3//PTP4A1//SRPX//CALCRL//METTL7A//RNF122//CPED1//TMTC2//GLYATL2//MSRB3//MAMDC2 |
| GO:0019898 | extrinsic to membrane | Cellular component | 9 | 130 | 479 | 17068 | 2.4668700819014 | 0.0110878997307777 | 0.128619636877021 | 1.95515071009219 | GNAL//GNG7//GNG11//ERRFI1//CNTFR//EPB41L2//GFRA1//GHR//PRNP |
| GO:0043005 | neuron projection | Cellular component | 28 | 634 | 479 | 17068 | 1.57367807538049 | 0.0123565832202747 | 0.140062589210135 | 1.90810160155467 | CNN3//NTRK2//PLCB4//MPDZ//NLGN1//ANKS1B//GFRA1//MYH10//NCAM2//CADM2//APOD//IL6ST//KCNIP1//TTLL7//TRIM9//DPYSL3//TMOD2//CST3//KCNMA1//OPHN1//LPAR1//MAP1B//PALMD//BOC//NFIB//DMD//BDKRB1//ITSN1 |
| GO:0005924 | cell-substrate adherens junction | Cellular component | 9 | 133 | 479 | 17068 | 2.41122639584347 | 0.012743053746498 | 0.140062589210135 | 1.89472648516141 | PGM5//TEK//TNS1//DLC1//SORBS1//FERMT2//TENC1//PARVA//NEXN |
| GO:0005884 | actin filament | Cellular component | 5 | 50 | 479 | 17068 | 3.56325678496868 | 0.012798822807133 | 0.140062589210135 | 1.89282997346752 | DPYSL3//FERMT2//ACTC1//ARHGAP6//TEK |
| GO:0043202 | lysosomal lumen | Cellular component | 6 | 71 | 479 | 17068 | 3.01120291687495 | 0.0145875206670561 | 0.153929210886694 | 1.83601851565389 | GPC5//FMOD//GPC3//OMD//OGN//GPC6 |
| GO:0045178 | basal part of cell | Cellular component | 4 | 34 | 479 | 17068 | 4.19206680584551 | 0.0145967355151175 | 0.153929210886694 | 1.83574426108508 | AQP1//CD34//ITGA9//TEK |
| GO:0044297 | cell body | Cellular component | 15 | 287 | 479 | 17068 | 1.86232933012301 | 0.0159081509024455 | 0.164762991489614 | 1.79838029801114 | CADM2//APOD//CNN3//CST3//GFRA1//GHR//IL6ST//MYH10//NTRK2//VIP//KCNIP1//BOC//TTLL7//DPYSL3//RDH10 |
| GO:0016010 | dystrophin-associated glycoprotein complex | Cellular component | 3 | 20 | 479 | 17068 | 5.34488517745303 | 0.0175552492510952 | 0.178632360800618 | 1.75559300000058 | SGCD//DMD//PGM5 |
| GO:0005911 | cell-cell junction | Cellular component | 15 | 292 | 479 | 17068 | 1.83044012926474 | 0.0183032392071959 | 0.182557321222989 | 1.73747204440496 | PGM5//SORBS1//DES//CDH5//PMP22//MPDZ//BVES//JAM2//CLMP//ANK2//TEK//FZD4//HEG1//CADM3//TMEM47 |
| GO:0070161 | anchoring junction | Cellular component | 12 | 215 | 479 | 17068 | 1.98879448463368 | 0.0185704861244075 | 0.182557321222989 | 1.73117672747269 | PPAP2B//SYNM//PGM5//SORBS1//DES//NEXN//TEK//TNS1//DLC1//FERMT2//TENC1//PARVA |
| GO:0005796 | Golgi lumen | Cellular component | 6 | 77 | 479 | 17068 | 2.77656372854703 | 0.0210166786736256 | 0.203161227178381 | 1.67743591569536 | GPC5//FMOD//GPC3//OMD//OGN//GPC6 |
| GO:0030425 | dendrite | Cellular component | 16 | 328 | 479 | 17068 | 1.73817404144814 | 0.0233179972793876 | 0.221712105279423 | 1.63230875270637 | CNN3//NTRK2//PLCB4//MPDZ//NLGN1//ANKS1B//LPAR1//MAP1B//MYH10//OPHN1//PALMD//APOD//IL6ST//KCNIP1//TTLL7//TRIM9 |
| GO:0030141 | secretory granule | Cellular component | 13 | 252 | 479 | 17068 | 1.83818802399178 | 0.0260659050699649 | 0.243842337751285 | 1.58392719074205 | CAV1//SELP//CD36//A2M//CLU//CFD//F13A1//IGF1//MMRN1//CPE//CTSG//PLA2G2A//SCG2 |
| GO:0005925 | focal adhesion | Cellular component | 8 | 128 | 479 | 17068 | 2.22703549060543 | 0.02791860680944 | 0.257028443642463 | 1.55410625761295 | PGM5//TEK//TNS1//DLC1//SORBS1//FERMT2//TENC1//PARVA |
| GO:0016460 | myosin II complex | Cellular component | 3 | 24 | 479 | 17068 | 4.45407098121086 | 0.0287060625334357 | 0.260148691709261 | 1.54202637341341 | MYH11//MYL9//MYH10 |
| GO:0016327 | apicolateral plasma membrane | Cellular component | 2 | 10 | 479 | 17068 | 7.12651356993737 | 0.030466876501001 | 0.271858282624317 | 1.51617206786177 | MPDZ//PRICKLE2 |
| GO:0043025 | neuronal cell body | Cellular component | 13 | 260 | 479 | 17068 | 1.78162839248434 | 0.0324250169537073 | 0.284947118684094 | 1.48911978816911 | CADM2//TTLL7//APOD//CNN3//CST3//GFRA1//GHR//IL6ST//MYH10//NTRK2//VIP//KCNIP1//BOC |
| GO:0016323 | basolateral plasma membrane | Cellular component | 9 | 158 | 479 | 17068 | 2.02970323194419 | 0.0343682964718013 | 0.297516596323056 | 1.46384199393876 | AQP1//CD34//ITGA9//TEK//ANK2//ANXA1//CAV1//CD44//TRPC1 |
| GO:0045211 | postsynaptic membrane | Cellular component | 10 | 185 | 479 | 17068 | 1.92608474863172 | 0.0362330725137202 | 0.309046794969966 | 1.44089483684109 | ANK2//CHRM2//DMD//KCNMA1//MUSK//MPDZ//NLGN1//FAIM2//ANKS1B//KCTD12 |
| GO:0043230 | extracellular organelle | Cellular component | 5 | 66 | 479 | 17068 | 2.69943695830961 | 0.0376487997774009 | 0.311947198155607 | 1.42424886430685 | AQP1//A2M//ACTC1//ANXA1//C3 |
| GO:0065010 | extracellular membrane-bounded organelle | Cellular component | 5 | 66 | 479 | 17068 | 2.69943695830961 | 0.0376487997774009 | 0.311947198155607 | 1.42424886430685 | AQP1//A2M//ACTC1//ANXA1//C3 |
| GO:0043205 | fibril | Cellular component | 2 | 12 | 479 | 17068 | 5.93876130828114 | 0.0430678461534521 | 0.351821841816933 | 1.36584684699896 | MFAP4//MFAP5 |
| GO:0005856 | cytoskeleton | Cellular component | 62 | 1785 | 479 | 17068 | 1.23765781886867 | 0.0451214958942738 | 0.363478716926094 | 1.34561651080088 | ANXA1//DES//SYNM//KRT222//KRTAP6-2//MYH10//MYH11//PGM5//TEK//MYL9//SORBS1//FERMT2//VCAM1//CETN2//PPP1R12A//PRKAR2B//TSPYL2//MAP7D3//TACC1//PTP4A1//TPM2//TTN//MAP1B//IQGAP2//CAPN6//ACTC1//ARHGAP6//TTLL7//EPB41L2//CNN3//NTRK2//PLCB4//MPDZ//NLGN1//ANKS1B//ACTA2//DMD//NFATC2//OPHN1//CTNNAL1//PARVA//SYNPO2//CRYAB//KLHL4//FILIP1L//TMOD1//DPYSL3//ACTG2//ANK2//CFL2//CNN1//FLNC//SGCD//TNS1//PDLIM1//AKAP12//WASF3//LMOD1//TMOD2//NEXN//TRIM9//TBCEL |
| GO:0043296 | apical junction complex | Cellular component | 7 | 117 | 479 | 17068 | 2.13186303374195 | 0.0467720052686395 | 0.371613192545355 | 1.33001401006935 | SORBS1//CDH5//PMP22//MPDZ//BVES//JAM2//CLMP |
| GO:0016021 | integral to membrane | Cellular component | 167 | 5337 | 479 | 17068 | 1.11497823325795 | 0.0482662211442955 | 0.378302814374208 | 1.3163567016044 | SCN7A//ANK2//AQP1//BDKRB1//CAV1//CD34//CD36//CD44//CHRM2//LPAR1//EDNRB//FCER1A//FGFR1//GPC5//GHR//GPC3//KDR//LIFR//CD200//MUSK//NTRK2//ROR1//FXYD1//PTGER3//PTGFR//PTPRD//PTPRS//SELP//SLCO2A1//TRGC2//TEK//TGFBR3//TSPAN7//TRPC1//FZD4//PPAP2A//SLC16A4//DCLK1//GPC6//GPR64//CALCRL//CORIN//LYVE1//NLGN1//KCNMB4//SLC22A17//CCRL1//JAM2//EDA2R//APCDD1//C7//ITGA9//ITGA8//CACNB4//CASQ2//IL6ST//SORBS1//KCNH1//KCNMA1//ABCC9//VDAC2//ST6GALNAC3//ANO6//TGFBR2//ABCD2//BAI3//CDH5//CLN5//CYB5A//EMP1//EPHX1//FMO1//DARC//KIT//KLRB1//MAN1A1//NCAM2//PCDH9//PLN//PMP22//PTGIS//SGCD//UGCG//VCAM1//PPAP2B//AOC3//B3GALT2//CH25H//PTPLA//GLP2R//ITM2A//LPPR4//UST//LHFP//KLRG1//ABCA8//PEMT//C5ORF4//BVES//SEC63//FAIM2//LPHN3//FRRS1L//PARM1//DHRS7B//SUSD5//TOR1AIP1//CDH19//DNAJC15//CDON//ERGIC2//GCNT4//SCARA3//UBE2J1//EMCN//PCDH18//TMEM100//CACNA2D3//PLSCR4//SLC44A2//RNF150//HEG1//GPAM//CACHD1//CADM3//ELTD1//LRRC19//OR4F5//FAT4//CLMP//RNF122//CYBRD1//CNTNAP3//SCD5//COLEC12//OR8J3//OR4A5//APOLD1//GLT8D2//PLVAP//TMEM47//TMTC1//TSPAN18//BOC//TMEM88//CYYR1//TM4SF18//C4ORF32//CHODL//MFSD4//RDH10//FREM1//ZDHHC15//SLC5A12//TMTC2//FAM171B//VSTM4//FAM171A1//FAM162B//PI16//KIAA1324L//CADM2//SLC9A9//RNF180//OR56B1//TMEM150C//OR4F21 |
| GO:0043235 | receptor complex | Cellular component | 9 | 169 | 479 | 17068 | 1.89759237069338 | 0.0491622490496782 | 0.380188059317511 | 1.30836825744705 | ITGA9//ITGA8//IL6ST//SORBS1//NLGN1//TGFBR2//GHR//GFRA1//KCTD12 |
