# Supplementary material for: Downregulation of miR-181b-5p Inhibits the Viability, Migration, and Glycolysis of Gallbladder Cancer by Upregulating PDHX Under Hypoxia
Source: Front Oncol. 2021 Aug 16;11:683725. doi: 10.3389/fonc.2021.683725 (PMC8415503; doi:10.3389/fonc.2021.683725)
Supplement: Supplementary file 3 [file DataSheet_1.zip › RNA seq raw data/HuGene 2.0 ST Data/GO Analysis/A vs B_down/BP_result(Human).html]

| GO.ID | Term | Ontology | Count | Pop.Hits | List.Total | Pop.Total | Fold.Enrichment | Pvalue | FDR | Enrichment.Score | GENES |
| --- | --- | --- | --- | --- | --- | --- | --- | --- | --- | --- | --- |
| GO:0007155 | cell adhesion | Biological process | 72 | 963 | 428 | 14747 | 2.57612018516901 | 7.14412534727563e-14 | 1.89013126725936e-10 | 13.1460509341373 | MGP//PTPRD//SELP//VCAM1//NLGN1//CADM3//PIK3R1//CD36//CCL21//SFRP1//CDH5//PCDH9//CDH19//PCDH18//FAT4//NCAM2//CD44//ECM2//TEK//TNXB//ITGA8//FBLN5//SORBS1//LYVE1//FREM1//ANGPT1//JAM2//VIT//ABI3BP//SMOC2//CCDC80//FZD4//CD34//COL19A1//COL14A1//SLIT2//LAMA2//PPP1R12A//PPAP2B//PARVA//BVES//ARHGAP6//DLC1//KDR//APOD//KIT//BMX//CNTN1//DPT//ITGA9//LSAMP//MFAP4//OMD//CNTN3//PGM5//PTPRS//CXCL12//SRPX//AOC3//CTNNAL1//MPDZ//SPON1//FERMT2//MMRN1//SUSD5//CDON//CNTNAP3//SVEP1//BOC//CPXM2//CADM2//NEGR1 |
| GO:0022610 | biological adhesion | Biological process | 72 | 965 | 428 | 14747 | 2.57078107597695 | 7.92674047917533e-14 | 1.89013126725936e-10 | 13.1009053603391 | MGP//PTPRD//SELP//VCAM1//NLGN1//CADM3//PIK3R1//CD36//CCL21//SFRP1//BMX//CNTN1//COL19A1//DPT//ITGA9//LAMA2//LSAMP//MFAP4//OMD//CNTN3//PGM5//PTPRS//CXCL12//TNXB//SRPX//AOC3//CTNNAL1//MPDZ//SPON1//FERMT2//MMRN1//SUSD5//CDON//CNTNAP3//SVEP1//BOC//CPXM2//CADM2//NEGR1//CDH5//PCDH9//CDH19//PCDH18//FAT4//NCAM2//CD44//ECM2//TEK//ITGA8//FBLN5//SORBS1//LYVE1//FREM1//ANGPT1//JAM2//VIT//ABI3BP//SMOC2//CCDC80//FZD4//CD34//COL14A1//SLIT2//PPP1R12A//PPAP2B//PARVA//BVES//ARHGAP6//DLC1//KDR//APOD//KIT |
| GO:0044707 | single-multicellular organism process | Biological process | 227 | 5432 | 428 | 14747 | 1.43987903114806 | 3.26863662753588e-12 | 5.19604269223954e-09 | 11.4856333570053 | COL19A1//FGFR1//FOXC1//FRZB//IGF1//IGFBP4//PTHLH//TLL1//PAPSS2//MGP//CHRDL2//CHRDL1//IGSF10//APOD//CAV1//EPAS1//FGF10//KDR//MEOX2//SHB//TEK//SCG2//CALCRL//APOLD1//KIT//ADAMTS1//MEF2C//TGFBR2//TGFBR3//PPAP2B//CXCL12//NTRK2//FZD4//TIPARP//HEG1//MYOCD//SFRP1//CST3//OSR1//ITGA8//SLIT2//SPRY1//RDH10//SMAD9//TCF21//CD44//FGF2//PBX1//GNG7//ANGPT1//MYH10//TTN//EDNRB//NR4A2//NR2F1//DCLK1//CCL21//FABP4//NFATC2//GFRA1//TENC1//ARID5B//DLC1//CD34//LDB2//APCDD1//CDH5//RECK//MN1//PLN//ANK2//CASQ2//DMD//PARVA//NFIB//NLGN1//CRYAB//CDON//ANO6//CORIN//AQP1//CPE//ACTA2//ACTG2//DES//MYH11//FXYD1//SCN7A//TPM2//MYL9//SORBS1//LMOD1//A2M//CD36//CLU//CFD//F13A1//KCNMA1//PDE1A//PIK3R1//PRKAR2B//SELP//WEE1//TFPI2//MMRN1//KCNMB4//PLSCR4//JAM2//COLEC12//CNN3//CNN1//CACNB4//GNAL//KCNH1//PMP22//ABCC9//PDE7B//KCNIP1//NCALD//TMOD2//BCHE//EBF1//EMP1//ERG//FOSB//SERPINF1//PTP4A1//PTPLA//RBM11//OLFML3//ADAMTS9//PDGFD//SPDYA//EBF3//SPA17//GATM//EDA2R//FOXP2//SLIT3//CHRM2//CNTFR//LSAMP//OPHN1//CNTN3//PTN//NRN1//DOK5//BEX1//TTLL7//SEMA3D//CNTN1//DPYSL3//ITGA9//LAMA2//PRNP//TRPC1//CAP2//BOC//RCAN1//RCAN2//CLN5//NNAT//OXCT1//SEPP1//PCDH18//CADM2//VCAM1//SGCD//TAGLN//BVES//CHODL//MUSK//FBLN1//VIP//EYA4//EFEMP1//RORB//TIMP3//SOBP//CNTNAP3//TMOD1//FGF7//UGCG//C3//IL6ST//LPAR1//LIPG//WASF3//PIK3C2A//TRIM9//ZDHHC15//MAP1B//BAI3//BDKRB1//PTPRS//FAIM2//NME5//TACC1//ASPA//ACTC1//PTPRD//ANXA1//DYRK3//EXOC6//TEX15//CMA1//PRICKLE2//HTN3//PTGER3//DARC//TNXB//PLA2G2A//AFF3//GHR//GPAM//MPDZ//ERRFI1//PTGDS//FCER1A//ZEB1//PTGIS//ITSN1//LPPR4//GCNT4//GPC3//FREM1//ANKRD42 |
| GO:0032501 | multicellular organismal process | Biological process | 233 | 5645 | 428 | 14747 | 1.42217122091339 | 4.58532241261689e-12 | 5.46685064644249e-09 | 11.3386301219138 | COL19A1//FGFR1//FOXC1//FRZB//IGF1//IGFBP4//PTHLH//TLL1//PAPSS2//MGP//CHRDL2//CHRDL1//IGSF10//APOD//CAV1//EPAS1//FGF10//KDR//MEOX2//SHB//TEK//SCG2//CALCRL//APOLD1//KIT//ADAMTS1//MEF2C//TGFBR2//TGFBR3//PPAP2B//CXCL12//NTRK2//FZD4//TIPARP//HEG1//MYOCD//SFRP1//CST3//OSR1//ITGA8//SLIT2//SPRY1//RDH10//SMAD9//TCF21//CD44//FGF2//PBX1//GNG7//ANGPT1//MYH10//TTN//EDNRB//NR4A2//NR2F1//DCLK1//CCL21//FABP4//NFATC2//GFRA1//TENC1//ARID5B//DLC1//CD34//LDB2//APCDD1//CDH5//RECK//MN1//PLN//ANK2//CASQ2//DMD//PARVA//NFIB//NLGN1//CRYAB//CDON//ANO6//CORIN//AQP1//CPE//ACTA2//ACTG2//DES//MYH11//FXYD1//SCN7A//TPM2//MYL9//SORBS1//LMOD1//A2M//CD36//CLU//CFD//F13A1//KCNMA1//PDE1A//PIK3R1//PRKAR2B//SELP//WEE1//TFPI2//MMRN1//KCNMB4//PLSCR4//JAM2//COLEC12//CNN3//CNN1//CACNB4//GNAL//KCNH1//PMP22//ABCC9//PDE7B//KCNIP1//NCALD//TMOD2//BCHE//EBF1//EMP1//ERG//FOSB//SERPINF1//PTP4A1//PTPLA//RBM11//OLFML3//ADAMTS9//PDGFD//SPDYA//EBF3//CETN2//NME5//GPR64//CCNI//SPA17//TEX15//GATM//EDA2R//FOXP2//SLIT3//CHRM2//CNTFR//LSAMP//OPHN1//CNTN3//PTN//NRN1//DOK5//BEX1//TTLL7//SEMA3D//CNTN1//DPYSL3//ITGA9//LAMA2//PRNP//TRPC1//CAP2//BOC//RCAN1//RCAN2//CLN5//NNAT//OXCT1//SEPP1//PCDH18//CADM2//VCAM1//SGCD//TAGLN//BVES//CHODL//MUSK//RLN1//FBLN1//PTGFR//VIP//EYA4//EFEMP1//RORB//TIMP3//SOBP//CNTNAP3//TMOD1//PPAP2A//FGF7//UGCG//C3//IL6ST//LPAR1//LIPG//WASF3//PIK3C2A//TRIM9//ZDHHC15//MAP1B//BAI3//BDKRB1//PTPRS//FAIM2//TACC1//ASPA//ACTC1//PTPRD//ANXA1//DYRK3//EXOC6//CMA1//PRICKLE2//HTN3//PTGER3//DARC//TNXB//PLA2G2A//AFF3//GHR//GPAM//MPDZ//ERRFI1//PTGDS//FCER1A//ZEB1//PTGIS//ITSN1//LPPR4//GCNT4//GPC3//FREM1//ANKRD42 |
| GO:0048731 | system development | Biological process | 156 | 3407 | 428 | 14747 | 1.57765622728357 | 1.83449430504074e-10 | 1.74974066814786e-07 | 9.73648363211911 | COL19A1//FGFR1//FOXC1//FRZB//IGF1//IGFBP4//PTHLH//TLL1//PAPSS2//MGP//APOD//CAV1//EPAS1//FGF10//KDR//MEOX2//SHB//TEK//SCG2//CALCRL//APOLD1//KIT//ADAMTS1//MEF2C//TGFBR2//TGFBR3//PPAP2B//CXCL12//NTRK2//FZD4//TIPARP//HEG1//MYOCD//CST3//CHRDL1//OSR1//ITGA8//SLIT2//SPRY1//RDH10//SMAD9//SFRP1//TCF21//CD44//FGF2//PBX1//EDNRB//MYH10//NR4A2//NR2F1//DCLK1//GFRA1//TENC1//ARID5B//DLC1//LDB2//APCDD1//CDH5//RECK//ANGPT1//PARVA//NFIB//CRYAB//CDON//CPE//ANK2//SLIT3//CHRM2//CNTFR//LSAMP//OPHN1//CNTN3//PTN//NLGN1//TMOD2//NRN1//DOK5//BEX1//TTLL7//SEMA3D//CACNB4//CNTN1//DPYSL3//ITGA9//LAMA2//MYH11//PRNP//TRPC1//MYL9//CAP2//BOC//RCAN1//RCAN2//CLN5//NNAT//OXCT1//SEPP1//PCDH18//CADM2//PMP22//VCAM1//TTN//DMD//SGCD//TAGLN//BVES//CHODL//FOXP2//KCNH1//MUSK//EMP1//FGF7//UGCG//EDA2R//LPAR1//WASF3//MAP1B//BAI3//SERPINF1//PTPRS//FAIM2//NME5//AQP1//TACC1//ASPA//CD34//PTPRD//PIK3R1//ANXA1//DYRK3//EXOC6//TEX15//CMA1//PRICKLE2//HTN3//EFEMP1//RORB//SOBP//EYA4//KCNMA1//MPDZ//ERRFI1//ZEB1//C3//PTGIS//IL6ST//PLN//WEE1//LPPR4//CHRDL2//ACTC1//GHR//GCNT4//GPC3//ACTA2//FREM1 |
| GO:0001944 | vasculature development | Biological process | 43 | 523 | 428 | 14747 | 2.83287021318418 | 7.08414979633905e-10 | 5.63071839645682e-07 | 9.14971226410595 | APOD//CAV1//EPAS1//FGF10//FGFR1//KDR//MEOX2//SHB//TEK//SCG2//CALCRL//APOLD1//MEF2C//TGFBR2//TGFBR3//PPAP2B//CXCL12//FOXC1//NTRK2//FZD4//TIPARP//HEG1//MYOCD//CDH5//RECK//ANGPT1//PARVA//FGF2//SLIT2//BAI3//SERPINF1//SFRP1//AQP1//C3//CD34//CMA1//PTGIS//IGF1//TCF21//ERRFI1//ACTA2//OSR1//FRZB |
| GO:0048646 | anatomical structure formation involved in morphogenesis | Biological process | 89 | 1615 | 428 | 14747 | 1.89879199097248 | 1.33587793255842e-09 | 6.69159692778938e-07 | 8.87423322422447 | APOD//CAV1//EPAS1//FGF10//FGFR1//KDR//MEOX2//SHB//TEK//SCG2//CALCRL//APOLD1//CXCL12//TGFBR2//FOXC1//NTRK2//FZD4//TIPARP//HEG1//MYOCD//CD44//FGF2//PBX1//TCF21//SPRY1//SFRP1//DLC1//SMAD9//MEF2C//PTHLH//ANGPT1//PARVA//SLIT2//CPE//ANK2//CACNB4//CNTN1//DPYSL3//GFRA1//ITGA9//LAMA2//MYH10//MYH11//OPHN1//PRNP//SLIT3//TRPC1//MYL9//CAP2//BOC//KCNH1//CDON//IGF1//BAI3//SERPINF1//DMD//FAIM2//NR4A2//DCLK1//NFIB//TMOD1//ACTC1//TTN//FGF7//PMP22//OSR1//RORB//ITGA8//SOBP//EYA4//ERRFI1//AQP1//C3//CD34//CMA1//PTGIS//MAP1B//RDH10//WEE1//PTPRD//TGFBR3//ARID5B//ADAMTS1//GHR//CRYAB//VCAM1//FRZB//FREM1//CD36 |
| GO:0072358 | cardiovascular system development | Biological process | 54 | 769 | 428 | 14747 | 2.41950949770913 | 1.38577545228195e-09 | 6.69159692778938e-07 | 8.85830713605566 | APOD//CAV1//EPAS1//FGF10//FGFR1//KDR//MEOX2//SHB//TEK//SCG2//CALCRL//APOLD1//MEF2C//TGFBR2//TGFBR3//PPAP2B//CXCL12//FOXC1//NTRK2//FZD4//TIPARP//HEG1//MYOCD//FRZB//SFRP1//TCF21//CDH5//RECK//ANGPT1//PARVA//FGF2//SLIT2//DLC1//CPE//ANK2//OXCT1//VCAM1//OSR1//MYH10//TTN//BAI3//SERPINF1//AQP1//C3//CD34//CMA1//PTGIS//PLN//MYH11//ACTC1//ADAMTS1//IGF1//ERRFI1//ACTA2 |
| GO:0072359 | circulatory system development | Biological process | 54 | 769 | 428 | 14747 | 2.41950949770913 | 1.38577545228195e-09 | 6.69159692778938e-07 | 8.85830713605566 | APOD//CAV1//EPAS1//FGF10//FGFR1//KDR//MEOX2//SHB//TEK//SCG2//CALCRL//APOLD1//MEF2C//TGFBR2//TGFBR3//PPAP2B//CXCL12//FOXC1//NTRK2//FZD4//TIPARP//HEG1//MYOCD//FRZB//SFRP1//TCF21//CDH5//RECK//ANGPT1//PARVA//FGF2//SLIT2//DLC1//CPE//ANK2//OXCT1//VCAM1//OSR1//MYH10//TTN//BAI3//SERPINF1//AQP1//C3//CD34//CMA1//PTGIS//PLN//MYH11//ACTC1//ADAMTS1//IGF1//ERRFI1//ACTA2 |
| GO:0031589 | cell-substrate adhesion | Biological process | 26 | 222 | 428 | 14747 | 4.03534141618254 | 1.47473311701456e-09 | 6.69159692778938e-07 | 8.83128656700447 | PIK3R1//CD36//CCL21//SFRP1//CD44//ECM2//TEK//TNXB//ITGA8//FBLN5//SORBS1//LYVE1//FREM1//VIT//ABI3BP//SMOC2//CCDC80//FZD4//BVES//PARVA//CD34//ARHGAP6//DLC1//KDR//APOD//ANGPT1 |
| GO:0009653 | anatomical structure morphogenesis | Biological process | 106 | 2071 | 428 | 14747 | 1.76354147393692 | 1.5434591362064e-09 | 6.69159692778938e-07 | 8.8115048641767 | MGP//APOD//CAV1//EPAS1//FGF10//FGFR1//KDR//MEOX2//SHB//TEK//SCG2//CALCRL//APOLD1//CXCL12//TGFBR2//FOXC1//NTRK2//FZD4//TIPARP//HEG1//MYOCD//CD44//FGF2//PBX1//TCF21//PPAP2B//SPRY1//TGFBR3//SFRP1//DLC1//SMAD9//MEF2C//PTHLH//ANGPT1//PARVA//SLIT2//CPE//SLIT3//ANK2//CACNB4//CNTN1//DPYSL3//GFRA1//ITGA9//LAMA2//MYH10//MYH11//OPHN1//PRNP//TRPC1//MYL9//CAP2//BOC//KCNH1//CDON//KIT//WASF3//FERMT2//BVES//PALMD//RHOJ//IGF1//BAI3//SERPINF1//DMD//FAIM2//NR4A2//DCLK1//NFIB//ZRANB1//SH3D19//TMOD1//ACTC1//TTN//FGF7//PMP22//OSR1//RDH10//AFF3//GATM//RORB//ITGA8//SOBP//EYA4//AQP1//CD34//ERRFI1//C3//CMA1//PTGIS//MAP1B//ARID5B//GCNT4//WEE1//PTPRD//FRZB//CST3//ADAMTS1//GHR//FOXP2//CRYAB//VCAM1//FREM1//CD36//GPC3//LYVE1 |
| GO:0007275 | multicellular organismal development | Biological process | 171 | 3972 | 428 | 14747 | 1.48336074484005 | 2.06207403480152e-09 | 8.19502589330704e-07 | 8.68569574626464 | COL19A1//FGFR1//FOXC1//FRZB//IGF1//IGFBP4//PTHLH//TLL1//PAPSS2//MGP//APOD//CAV1//EPAS1//FGF10//KDR//MEOX2//SHB//TEK//SCG2//CALCRL//APOLD1//KIT//ADAMTS1//MEF2C//TGFBR2//TGFBR3//PPAP2B//CXCL12//NTRK2//FZD4//TIPARP//HEG1//MYOCD//CST3//CHRDL1//OSR1//ITGA8//SLIT2//SPRY1//RDH10//SMAD9//SFRP1//TCF21//CD44//FGF2//PBX1//ANGPT1//MYH10//TTN//EDNRB//NR4A2//NR2F1//DCLK1//GFRA1//TENC1//ARID5B//DLC1//LDB2//APCDD1//CDH5//RECK//PARVA//NFIB//CRYAB//CDON//CPE//ANK2//GATM//EDA2R//FOXP2//SLIT3//CHRM2//CNTFR//LSAMP//OPHN1//CNTN3//PTN//NLGN1//TMOD2//NRN1//DOK5//BEX1//TTLL7//SEMA3D//CACNB4//CNTN1//DPYSL3//ITGA9//LAMA2//MYH11//PRNP//TRPC1//MYL9//CAP2//BOC//RCAN1//RCAN2//CLN5//NNAT//OXCT1//SEPP1//PCDH18//CADM2//PMP22//VCAM1//DMD//SGCD//TAGLN//BVES//CHODL//KCNH1//MUSK//FBLN1//EMP1//FGF7//UGCG//LPAR1//WASF3//MAP1B//BAI3//SERPINF1//PTPRS//FAIM2//NME5//AQP1//TACC1//ASPA//CD34//PTPRD//PIK3R1//ANXA1//DYRK3//EXOC6//TEX15//CMA1//PRICKLE2//HTN3//EFEMP1//AFF3//RORB//SOBP//EYA4//KCNMA1//MPDZ//ERRFI1//ZEB1//C3//PTGIS//IL6ST//PLN//WEE1//LPPR4//CHRDL2//ACTC1//GHR//GCNT4//GPC3//ACTA2//FREM1//EBF1//ERG//FOSB//PTP4A1//PTPLA//RBM11//OLFML3//ADAMTS9//PDGFD//SPDYA//EBF3//IGSF10 |
| GO:0061061 | muscle structure development | Biological process | 39 | 464 | 428 | 14747 | 2.896053214631 | 2.47901156308823e-09 | 9.09415857259059e-07 | 8.6057214475066 | CRYAB//DMD//IGF1//LAMA2//MEF2C//SGCD//TAGLN//BVES//CHODL//CAV1//COL19A1//MEOX2//FOXP2//KCNH1//CDON//CACNB4//MUSK//MYOCD//TCF21//TMOD1//ACTC1//TTN//MYH11//ANK2//EDNRB//BOC//FGF10//EPAS1//ARID5B//RCAN1//FLNC//MYH10//FOXC1//TGFBR3//TGFBR2//FGF2//FGFR1//CNTFR//ITGA8 |
| GO:0072001 | renal system development | Biological process | 27 | 246 | 428 | 14747 | 3.78171301572829 | 2.99671663382502e-09 | 1.02081011619368e-06 | 8.52335432151361 | FGF10//FOXC1//ITGA8//SLIT2//SPRY1//RDH10//FGFR1//SMAD9//SFRP1//TCF21//OSR1//CD44//FGF2//PBX1//GFRA1//ADAMTS1//TENC1//TIPARP//ARID5B//MYOCD//GCNT4//MEF2C//CD34//ANGPT1//TEK//GPC3//ACTA2 |
| GO:0048856 | anatomical structure development | Biological process | 169 | 3934 | 428 | 14747 | 1.48017225814728 | 3.30748349728406e-09 | 1.05155925323651e-06 | 8.48050231405092 | COL19A1//FGFR1//FOXC1//FRZB//IGF1//IGFBP4//PTHLH//TLL1//PAPSS2//MGP//APOD//CAV1//EPAS1//FGF10//KDR//MEOX2//SHB//TEK//SCG2//CALCRL//APOLD1//KIT//ADAMTS1//MEF2C//TGFBR2//TGFBR3//PPAP2B//CXCL12//NTRK2//FZD4//TIPARP//HEG1//MYOCD//CST3//CHRDL1//OSR1//ITGA8//SLIT2//SPRY1//RDH10//SMAD9//SFRP1//TCF21//CD44//FGF2//PBX1//ANGPT1//MYH10//TTN//EDNRB//NR4A2//NR2F1//DCLK1//GFRA1//TENC1//ARID5B//DLC1//LDB2//APCDD1//CDH5//RECK//PARVA//NFIB//CRYAB//CDON//CD34//CPE//ANK2//CNTFR//NME5//EYA4//GPC3//LYVE1//GATM//EDA2R//FOXP2//SLIT3//CHRM2//LSAMP//OPHN1//CNTN3//PTN//NLGN1//TMOD2//NRN1//DOK5//BEX1//TTLL7//SEMA3D//CACNB4//CNTN1//DPYSL3//ITGA9//LAMA2//MYH11//PRNP//TRPC1//MYL9//CAP2//BOC//RCAN1//RCAN2//CLN5//NNAT//OXCT1//SEPP1//PCDH18//CADM2//PMP22//BMX//VCAM1//DMD//SGCD//TAGLN//BVES//CHODL//KCNH1//MUSK//WASF3//FERMT2//PALMD//RHOJ//EMP1//FGF7//UGCG//LPAR1//MAP1B//BAI3//SERPINF1//PTPRS//FAIM2//AQP1//TACC1//ASPA//ZRANB1//SH3D19//PTPRD//PIK3R1//ANXA1//DYRK3//EXOC6//TMOD1//ACTC1//TEX15//CMA1//PRICKLE2//HTN3//EFEMP1//AFF3//RORB//SOBP//KCNMA1//MPDZ//ERRFI1//ZEB1//C3//PTGIS//CCL21//IL6ST//GCNT4//PLN//FLNC//WEE1//LPPR4//CHRDL2//GHR//ACTA2//FREM1//CD36 |
| GO:0001655 | urogenital system development | Biological process | 29 | 285 | 428 | 14747 | 3.50600918183309 | 4.46188227959993e-09 | 1.32991978696325e-06 | 8.35048189214151 | FGF10//FOXC1//ITGA8//SLIT2//SPRY1//RDH10//FGFR1//SMAD9//SFRP1//TCF21//OSR1//CD44//FGF2//PBX1//GFRA1//ADAMTS1//TENC1//TIPARP//ARID5B//MYOCD//IGF1//SERPINF1//GCNT4//MEF2C//CD34//ANGPT1//TEK//GPC3//ACTA2 |
| GO:0001568 | blood vessel development | Biological process | 40 | 498 | 428 | 14747 | 2.76751867282213 | 5.63650170108319e-09 | 1.58120450661563e-06 | 8.24899035759342 | APOD//CAV1//EPAS1//FGF10//FGFR1//KDR//MEOX2//SHB//TEK//SCG2//CALCRL//APOLD1//CXCL12//TGFBR2//FOXC1//NTRK2//FZD4//TIPARP//HEG1//MYOCD//CDH5//RECK//ANGPT1//PARVA//FGF2//SLIT2//BAI3//SERPINF1//SFRP1//AQP1//C3//CD34//CMA1//PTGIS//ACTA2//OSR1//TCF21//MEF2C//TGFBR3//PPAP2B |
| GO:0032502 | developmental process | Biological process | 186 | 4496 | 428 | 14747 | 1.42543215984302 | 6.24193894187881e-09 | 1.65376704521222e-06 | 8.20468048388197 | COL19A1//FGFR1//FOXC1//FRZB//IGF1//IGFBP4//PTHLH//TLL1//PAPSS2//MGP//APOD//CAV1//EPAS1//FGF10//KDR//MEOX2//SHB//TEK//SCG2//CALCRL//APOLD1//KIT//ADAMTS1//MEF2C//TGFBR2//TGFBR3//PPAP2B//CXCL12//NTRK2//FZD4//TIPARP//HEG1//MYOCD//SFRP1//CST3//CHRDL1//OSR1//ITGA8//SLIT2//SPRY1//RDH10//SMAD9//TCF21//CD44//FGF2//PBX1//ANGPT1//MYH10//TTN//CDON//EDNRB//NR4A2//NR2F1//DCLK1//GFRA1//TENC1//ARID5B//DLC1//LDB2//APCDD1//CDH5//RECK//PARVA//NFIB//CRYAB//CD34//CPE//ANK2//CNTFR//EBF1//EMP1//ERG//FOSB//SERPINF1//PTP4A1//PTPLA//RBM11//OLFML3//ADAMTS9//PDGFD//SPDYA//EBF3//IGSF10//NME5//EYA4//GPC3//LYVE1//GATM//EDA2R//FOXP2//SLIT3//CHRM2//LSAMP//OPHN1//CNTN3//PTN//NLGN1//TMOD2//NRN1//DOK5//BEX1//TTLL7//SEMA3D//CACNB4//CNTN1//DPYSL3//ITGA9//LAMA2//MYH11//PRNP//TRPC1//MYL9//CAP2//BOC//RCAN1//RCAN2//CLN5//NNAT//OXCT1//SEPP1//PCDH18//CADM2//PMP22//BMX//VCAM1//DMD//SGCD//TAGLN//BVES//CHODL//KCNH1//MUSK//FBLN1//WASF3//FERMT2//PALMD//RHOJ//FGF7//UGCG//CD36//PLA2G2A//LPAR1//ZDHHC15//MAP1B//BAI3//PTPRS//FAIM2//AQP1//TACC1//ASPA//ZRANB1//SH3D19//CHRDL2//PTPRD//PIK3R1//ANXA1//DYRK3//EXOC6//TMOD1//ACTC1//TEX15//CMA1//PRICKLE2//HTN3//EFEMP1//AFF3//RORB//SOBP//KCNMA1//MPDZ//ERRFI1//GHR//ZEB1//IL6ST//C3//PTGIS//CCL21//GCNT4//PLN//FLNC//WEE1//LPPR4//A2M//FABP4//ACTA2//FREM1 |
| GO:0044699 | single-organism process | Biological process | 362 | 10796 | 428 | 14747 | 1.1553288168342 | 9.27570272356774e-09 | 2.3282013836155e-06 | 8.03265317848844 | WEE1//CCNC//DIRAS3//SPDYA//CETN2//PRKAR2B//CAV1//FGFR1//MEF2C//MAPK4//SCG2//DOK5//FGF2//LPAR1//FGF10//GHR//KIT//TACC1//MYH10//COL19A1//FOXC1//FRZB//IGF1//IGFBP4//PTHLH//TLL1//PAPSS2//MGP//CHRDL2//CHRDL1//IGSF10//KCNMB4//APOD//EPAS1//KDR//MEOX2//SHB//TEK//CALCRL//APOLD1//SPA17//ADAMTS1//EMP1//TGFBR3//FBLN5//MYOCD//TGFBR2//PPAP2B//CXCL12//NTRK2//FZD4//TIPARP//HEG1//PTPRD//SELP//VCAM1//NLGN1//CADM3//CAPN6//MAP1B//SFRP1//CST3//OSR1//ITGA8//SLIT2//SPRY1//RDH10//SMAD9//TCF21//CD44//PBX1//GNG7//ANGPT1//TTN//CDON//EDNRB//NR4A2//NR2F1//DCLK1//CCL21//FABP4//NFATC2//FCER1A//GFRA1//TENC1//ARID5B//CLU//DLC1//SRPX//A2M//CD34//BVES//VIP//LDB2//APCDD1//PIK3R1//CD36//CDH5//RECK//MN1//C3//PLN//ANK2//CASQ2//DMD//PARVA//FOXP2//NFIB//CRYAB//COLEC12//ANO6//CFD//F13A1//MMRN1//BDKRB1//CORIN//AQP1//CPE//NME5//CNTFR//TSPYL2//TSPYL5//ACTA2//ACTG2//DES//MYH11//FXYD1//SCN7A//TPM2//MYL9//SORBS1//LMOD1//DPYSL3//OPHN1//TNXB//FERMT2//DAAM2//RHOJ//KCNMA1//PDE1A//TFPI2//PLSCR4//JAM2//C7//CFH//SGCD//CAP2//WASF3//ZRANB1//SH3D19//ACTC1//BMX//ITSN1//HIPK3//FAIM2//KANK2//PRUNE2//CNTN1//DPT//ITGA9//LAMA2//LSAMP//MFAP4//OMD//CNTN3//PGM5//PTPRS//AOC3//CTNNAL1//MPDZ//SPON1//SUSD5//CNTNAP3//SVEP1//BOC//CPXM2//CADM2//NEGR1//ANXA1//RCAN1//ERG//FGF7//GEM//GNAL//GNG11//PPP1R12A//RLN1//SPARCL1//RGS5//PTPLA//IQGAP2//PDE7B//KCNIP1//SLC44A2//SMOC2//PPP1R1C//MUSK//ROR1//ANGPTL1//PLCB4//PREX2//ASB5//DCDC1//ERRFI1//EYA4//GTF2H5//AKAP12//SEC63//ALDH1A1//FMO1//GSTA2//GSTM5//MAT2A//PTGIS//SLC22A17//SLC9A9//KCNH1//ABCC9//SLC5A12//TRPC1//CACHD1//VDAC2//SLCO2A1//OSBPL6//CACNB4//PRNP//CYBRD1//PIK3C2A//RAB9B//EXOC6//PLAGL1//LYVE1//CNN3//CNN1//CLN5//TNS1//PTP4A1//PYHIN1//CAB39L//TEX15//FREM1//PXDC1//PCDH9//CDH19//PCDH18//FAT4//NCAM2//ECM2//KLRB1//LIFR//KLRG1//CD160//EFEMP1//FMOD//PTN//BAI3//CHRM2//PTGER3//PTGFR//GPR64//LPHN3//CCRL1//ELTD1//GLP2R//PPAP2A//RCAN2//ARHGAP6//RERGL//ARHGAP28//RABL3//KSR1//SH2D1A//PMP22//NCALD//TMOD2//BCHE//EBF1//FOSB//SERPINF1//RBM11//OLFML3//ADAMTS9//PDGFD//EBF3//CCNI//GATM//EDA2R//SLIT3//NRN1//BEX1//TTLL7//SEMA3D//NNAT//OXCT1//SEPP1//TAGLN//CHODL//FBLN1//RORB//TIMP3//SOBP//IL6ST//TAF9B//PTX3//KCTD12//ZEB1//LIPG//PEMT//HDGFRP3//PODN//PDK4//TMOD1//PALMD//UGCG//CBLN4//PLA2G2A//VIT//ABI3BP//CCDC80//SLC16A4//TRIM9//ZDHHC15//ZNF738//COL14A1//GPC6//SOD3//REV3L//ASPA//CMA1//CTSG//GLT25D2//CRISPLD2//DYRK3//WFDC1//NEXN//EPB41L2//PRICKLE2//HTN3//DARC//FLNC//TOR1AIP1//ARHGEF26//AFF3//TEAD1//RGS22//GPAM//GPX3//MFAP5//CILP//SYNM//PTGDS//PDE8B//RANBP3L//OGN//GCNT4//LPPR4//ABCD2//ABCA8//MFSD4//MT1A//GPC3//ANKRD42 |
| GO:0050896 | response to stimulus | Biological process | 250 | 6642 | 428 | 14747 | 1.29688375024976 | 1.21667027425834e-08 | 2.90115026896901e-06 | 7.91482710251949 | CAV1//FGFR1//MEF2C//MAPK4//SCG2//DOK5//FGF2//LPAR1//FGF10//GHR//KIT//APOD//MYOCD//GNG7//CRYAB//CST3//EPAS1//KCNMA1//SMAD9//NR4A2//SOD3//TGFBR3//VCAM1//PDLIM1//APOLD1//MYH10//C3//FCER1A//CLU//A2M//PTX3//CNTFR//GNAL//GCNT4//COLEC12//CD44//CCL21//ANO6//ANXA1//IL6ST//SLIT2//DARC//HTN3//CTSG//ACTA2//CHRM2//CXCL12//DCLK1//ANGPT1//CD36//CFD//F13A1//IGF1//PDE1A//PIK3R1//PRKAR2B//SELP//TEK//TTN//WEE1//TFPI2//PAPSS2//MMRN1//KCNMB4//PLSCR4//JAM2//C7//CFH//FRZB//FZD4//ZRANB1//APCDD1//BMX//CD34//RCAN1//ERG//FGF7//GEM//GNG11//IGFBP4//MFAP4//PPP1R12A//OPHN1//RLN1//SHB//NR2F1//TNXB//SPARCL1//RGS5//PTPLA//CAP2//IQGAP2//PDE7B//KCNIP1//SPA17//SLC44A2//SMOC2//CNTNAP3//PPP1R1C//KDR//MUSK//NTRK2//ROR1//ANGPTL1//PLCB4//TENC1//PREX2//ASB5//DCDC1//ERRFI1//EYA4//CETN2//GTF2H5//ALDH1A1//FMO1//GSTA2//GSTM5//MAT2A//PTGIS//CCRL1//TGFBR2//HSPB6//BDKRB1//AOC3//KLRG1//IGJ//ZEB1//TRGC2//SH2D1A//CD160//NFATC2//SPDYA//GATM//PRNP//SEPP1//SCARA3//GPX3//TSPYL2//EDNRB//GFRA1//KLRB1//LIFR//EFEMP1//PIK3C2A//SPRY1//CCNC//FMOD//PTN//PTPRD//BAI3//PTGER3//PTGFR//VIP//AKAP12//GPR64//LPHN3//ELTD1//CALCRL//GLP2R//PTHLH//PPAP2A//CACNB4//CPE//CNTN1//FOXC1//CDON//BOC//ITGA9//ITGA8//ADAMTS1//RCAN2//ARHGAP6//ITSN1//DIRAS3//DLC1//RAB9B//RHOJ//RERGL//ARHGAP28//RABL3//KSR1//CTNNAL1//ANK2//DPYSL3//LAMA2//MYH11//SLIT3//TRPC1//MYL9//OXCT1//LIPG//FOSB//TMOD2//SOBP//PLA2G2A//KCTD12//PDK4//SORBS1//CAB39L//TMOD1//SFRP1//ACTC1//AQP1//WFDC1//EPHX1//LYVE1//SNRPN//NNAT//GPAM//AMPD1//CYBRD1//TEX15//NLGN1//CASQ2//DMD//PLN//TAF9B//REV3L//PDGFD//SRPX//PPAP2B//CHRDL1//RORB//KANK2//EDA2R//AFF3//ARHGEF26//TEAD1//RGS22//FOXP2//HIPK3//CILP//PTGDS//TIPARP//ARID5B//CMA1//FABP4//CD200//ABCC9//TSPYL5//TCF21//MT1A//OSR1//TIMP3//PARVA//NFIB//ANKRD42//FBLN5//VDAC2 |
| GO:0048513 | organ development | Biological process | 114 | 2417 | 428 | 14747 | 1.62513001751611 | 3.44023005930196e-08 | 7.81259864419574e-06 | 7.46341251378181 | MGP//FOXC1//KDR//KIT//ADAMTS1//CST3//CHRDL1//FGF10//ITGA8//SLIT2//SPRY1//RDH10//EDNRB//FGF2//FGFR1//GFRA1//TCF21//TENC1//TIPARP//ARID5B//OSR1//TGFBR3//EPAS1//LDB2//APCDD1//MEF2C//PTHLH//NFIB//CRYAB//TGFBR2//CDON//SFRP1//DLC1//PARVA//HEG1//CPE//MYOCD//ANK2//PBX1//SLIT3//APOD//CLN5//FRZB//NNAT//OXCT1//CXCL12//SEPP1//FZD4//PCDH18//CADM2//TEK//VCAM1//CALCRL//MYH10//TTN//DMD//IGF1//LAMA2//SGCD//TAGLN//BVES//CHODL//CAV1//COL19A1//MEOX2//FOXP2//KCNH1//CACNB4//MUSK//EMP1//FGF7//UGCG//EDA2R//CNTN1//PTPRS//FAIM2//NME5//AQP1//NR4A2//NTRK2//TACC1//ANGPT1//CD34//PIK3R1//ANXA1//DYRK3//EXOC6//PTN//TEX15//NR2F1//DCLK1//CMA1//SMAD9//HTN3//EFEMP1//RORB//SOBP//EYA4//KCNMA1//ERRFI1//BOC//PLN//MYH11//RCAN1//LPPR4//CD44//CHRDL2//ACTC1//GHR//CNTFR//SERPINF1//GCNT4//ACTA2//FREM1 |
| GO:0003012 | muscle system process | Biological process | 28 | 295 | 428 | 14747 | 3.27036274354507 | 3.78048208612088e-08 | 8.19505412214113e-06 | 7.42245281550873 | TTN//ACTA2//ACTG2//CRYAB//DES//MYH11//FXYD1//SCN7A//TPM2//MYL9//SORBS1//LMOD1//CALCRL//CNN3//CAV1//CNN1//CASQ2//MYOCD//IGF1//IL6ST//ANK2//DMD//PLN//EDNRB//PIK3C2A//ACTC1//TMOD1//KCNMA1 |
| GO:0006936 | muscle contraction | Biological process | 26 | 263 | 428 | 14747 | 3.4062577733556 | 5.16036409500146e-08 | 1.06999027691574e-05 | 7.28731965518062 | CALCRL//MYL9//CNN3//MYH11//CAV1//CNN1//CASQ2//TTN//MYOCD//ANK2//DMD//PLN//EDNRB//ACTA2//PIK3C2A//ACTC1//DES//TMOD1//TPM2//KCNMA1//ACTG2//CRYAB//FXYD1//SCN7A//SORBS1//LMOD1 |
| GO:0007517 | muscle organ development | Biological process | 30 | 343 | 428 | 14747 | 3.0136099833792 | 7.72694467616957e-08 | 1.53540829836053e-05 | 7.11199219723087 | CAV1//COL19A1//MEF2C//MEOX2//FOXP2//KCNH1//CDON//CACNB4//MUSK//MYOCD//TCF21//DMD//IGF1//BOC//ARID5B//RCAN1//ACTC1//TTN//FOXC1//TGFBR3//TGFBR2//FGF2//FGFR1//CNTFR//CRYAB//LAMA2//SGCD//TAGLN//BVES//CHODL |
| GO:0009605 | response to external stimulus | Biological process | 69 | 1247 | 428 | 14747 | 1.9065251931739 | 1.19781533902831e-07 | 2.2849525407304e-05 | 6.92161012971174 | MYOCD//C3//FCER1A//A2M//CCL21//ANO6//KIT//ANXA1//IL6ST//SLIT2//FGF2//CXCL12//CCRL1//RCAN1//TGFBR2//ANK2//CACNB4//CNTN1//DPYSL3//FGFR1//GFRA1//ITGA9//LAMA2//MYH10//MYH11//OPHN1//PRNP//SLIT3//TRPC1//MYL9//CAP2//BOC//GATM//OXCT1//VCAM1//LIPG//CAV1//PDK4//SFRP1//SELP//NTRK2//AQP1//CD36//CD34//CFH//PTGER3//CST3//NR4A2//GHR//TTN//FOXP2//SCG2//EDNRB//CMA1//PTGIS//TEK//CALCRL//FABP4//PLA2G2A//ANGPT1//FGF7//FGF10//KDR//KCNMA1//PARVA//LPAR1//NFIB//APOD//ANKRD42 |
| GO:0044767 | single-organism developmental process | Biological process | 147 | 3455 | 428 | 14747 | 1.46598387816655 | 1.38737818824449e-07 | 2.54477176143768e-05 | 6.85780513757147 | MGP//APOD//CAV1//EPAS1//FGF10//FGFR1//KDR//MEOX2//SHB//TEK//SCG2//CALCRL//APOLD1//FOXC1//KIT//ADAMTS1//MEF2C//TGFBR2//TGFBR3//PPAP2B//CXCL12//NTRK2//FZD4//TIPARP//HEG1//MYOCD//CST3//CHRDL1//ITGA8//SLIT2//SPRY1//RDH10//CD44//FGF2//PBX1//TCF21//ANGPT1//MYH10//TTN//EDNRB//GFRA1//TENC1//ARID5B//OSR1//SFRP1//DLC1//SMAD9//CDH5//RECK//PTHLH//PARVA//CRYAB//CDON//CPE//ANK2//CNTFR//NME5//GATM//EDA2R//FOXP2//SLIT3//CACNB4//CNTN1//DPYSL3//ITGA9//LAMA2//MYH11//OPHN1//PRNP//TRPC1//MYL9//CAP2//BOC//CLN5//FRZB//NNAT//OXCT1//SEPP1//PCDH18//CADM2//VCAM1//DMD//IGF1//SGCD//TAGLN//BVES//CHODL//COL19A1//KCNH1//MUSK//WASF3//FERMT2//PALMD//RHOJ//LPAR1//PMP22//ZDHHC15//MAP1B//BAI3//SERPINF1//PTPRS//FAIM2//AQP1//NFIB//NR4A2//DCLK1//TACC1//ASPA//ZRANB1//SH3D19//CD34//PIK3R1//DYRK3//EXOC6//TMOD1//ACTC1//TEX15//NR2F1//CMA1//ANXA1//FGF7//PRICKLE2//LDB2//AFF3//RORB//SOBP//EYA4//KCNMA1//ERRFI1//NLGN1//ZEB1//C3//PTGIS//CCL21//IL6ST//GCNT4//PLN//RCAN1//FLNC//WEE1//LPPR4//PTPRD//PAPSS2//GHR//ACTA2//FREM1//CD36 |
| GO:0030198 | extracellular matrix organization | Biological process | 22 | 209 | 428 | 14747 | 3.62690605017216 | 1.87094657213298e-07 | 3.25642574407593e-05 | 6.72793861429536 | CST3//CMA1//CTSG//DPT//FOXC1//TNXB//COL14A1//MFAP5//MYH11//FBLN5//COL19A1//ECM2//FBLN1//VIT//TLL1//RECK//ITGA8//GLT25D2//ABI3BP//SMOC2//CRISPLD2//CCDC80 |
| GO:0003008 | system process | Biological process | 86 | 1711 | 428 | 14747 | 1.73184233956204 | 1.91192956246857e-07 | 3.25642574407593e-05 | 6.71852811163874 | PLN//ANK2//CASQ2//DMD//EPAS1//NLGN1//HEG1//CORIN//CAV1//EDNRB//CD34//AQP1//TTN//ACTA2//ACTG2//CRYAB//DES//MYH11//FXYD1//SCN7A//TPM2//MYL9//SORBS1//LMOD1//CALCRL//CNN3//CNN1//CACNB4//GNAL//GNG7//KCNH1//KCNMA1//PMP22//ABCC9//PDE7B//KCNMB4//KCNIP1//NCALD//TMOD2//BCHE//CLN5//EYA4//EFEMP1//RORB//TIMP3//RDH10//FGFR1//FZD4//SOBP//MEF2C//PRNP//VIP//NTRK2//PRKAR2B//PTN//MUSK//ITGA8//RCAN1//MEOX2//CXCL12//CHRM2//MYOCD//IGF1//IL6ST//PIK3C2A//TRIM9//ZDHHC15//BDKRB1//ASPA//ACTC1//TMOD1//WASF3//LAMA2//FOXP2//LPAR1//MPDZ//TEK//ANGPT1//SLIT2//TRPC1//FGF10//OPHN1//ITSN1//FOXC1//MYH10//KIT |
| GO:0043062 | extracellular structure organization | Biological process | 22 | 210 | 428 | 14747 | 3.60963506898086 | 2.03476783880852e-07 | 3.34614062871649e-05 | 6.69148513537053 | CST3//CMA1//CTSG//COL19A1//ECM2//FBLN1//VIT//TLL1//COL14A1//RECK//ITGA8//GLT25D2//ABI3BP//SMOC2//CRISPLD2//CCDC80//DPT//FOXC1//TNXB//MFAP5//MYH11//FBLN5 |
| GO:0060537 | muscle tissue development | Biological process | 28 | 322 | 428 | 14747 | 2.996139780577 | 2.38970668660543e-07 | 3.7988370628071e-05 | 6.62165540123011 | CAV1//COL19A1//MEF2C//MEOX2//FOXP2//KCNH1//CDON//CACNB4//MUSK//MYOCD//TCF21//DMD//IGF1//BOC//PLN//MYH11//TTN//RCAN1//ITGA8//TIPARP//ACTC1//MYH10//FOXC1//TGFBR3//TGFBR2//FGF2//FGFR1//OSR1 |
| GO:0007399 | nervous system development | Biological process | 86 | 1729 | 428 | 14747 | 1.71381274898245 | 3.01724511336545e-07 | 4.6416909502064e-05 | 6.52038940740605 | FGFR1//MYH10//NTRK2//NR4A2//NR2F1//DCLK1//SFRP1//DLC1//ANK2//CACNB4//CNTN1//DPYSL3//GFRA1//ITGA9//LAMA2//MYH11//OPHN1//PRNP//SLIT3//TRPC1//SLIT2//MYL9//CAP2//BOC//NLGN1//RCAN1//RCAN2//APOD//CLN5//CST3//FOXC1//FRZB//NNAT//OXCT1//CXCL12//SEPP1//TGFBR2//FZD4//PCDH18//CADM2//EDNRB//PMP22//DMD//MUSK//FGF2//IGF1//NFIB//LPAR1//WASF3//MAP1B//PTPRS//FAIM2//FOXP2//NME5//AQP1//FGF10//TACC1//CDON//ASPA//MEF2C//PTPRD//CMA1//SMAD9//PRICKLE2//ANXA1//RORB//KCNMA1//MPDZ//PBX1//ZEB1//IL6ST//WEE1//SERPINF1//CHRM2//CNTFR//LSAMP//CNTN3//PTN//ITGA8//TMOD2//NRN1//DOK5//BEX1//TTLL7//CHRDL1//SEMA3D |
| GO:0007160 | cell-matrix adhesion | Biological process | 18 | 149 | 428 | 14747 | 4.16242237972778 | 3.19539977846602e-07 | 4.76214423234514e-05 | 6.49547479925194 | PIK3R1//CD36//CCL21//SFRP1//CD34//ARHGAP6//DLC1//SORBS1//KDR//TEK//APOD//CD44//ECM2//TNXB//ITGA8//FBLN5//LYVE1//FREM1 |
| GO:0023052 | signaling | Biological process | 185 | 4704 | 428 | 14747 | 1.3550781001653 | 4.05350961155281e-07 | 5.68564333455746e-05 | 6.39216879286025 | CAV1//FGFR1//MEF2C//MAPK4//SCG2//DOK5//FGF2//LPAR1//FGF10//GHR//KIT//SPRY1//FCER1A//CLU//BVES//COLEC12//CD44//FRZB//FZD4//ZRANB1//APCDD1//ANXA1//BMX//C3//CD34//CNTFR//RCAN1//EPAS1//ERG//FGF7//GEM//GNAL//GNG11//IGF1//IGFBP4//MFAP4//PPP1R12A//NR4A2//OPHN1//PDE1A//PRKAR2B//RLN1//CXCL12//SHB//TEK//NR2F1//TNXB//SPARCL1//RGS5//PTPLA//CAP2//IQGAP2//PDE7B//KCNIP1//SPA17//SLC44A2//SMOC2//CNTNAP3//PPP1R1C//KDR//MUSK//NTRK2//ROR1//ANGPTL1//PLCB4//DCLK1//TENC1//PREX2//ASB5//DCDC1//ERRFI1//NLGN1//TSPYL2//CD36//EDNRB//GFRA1//KLRB1//LIFR//KLRG1//CD160//GNG7//ANGPT1//EFEMP1//PIK3C2A//PIK3R1//CCNC//SMAD9//TGFBR2//TGFBR3//FMOD//PTN//PTPRD//BAI3//CHRM2//PTGER3//PTGFR//VIP//AKAP12//GPR64//LPHN3//CCRL1//ELTD1//CALCRL//GLP2R//PTHLH//BDKRB1//PPAP2A//CACNB4//CPE//CNTN1//FOXC1//CDON//BOC//ITGA9//ITGA8//ADAMTS1//RCAN2//A2M//ARHGAP6//ITSN1//DIRAS3//DLC1//RAB9B//RHOJ//RERGL//ARHGAP28//RABL3//KSR1//CTNNAL1//SH2D1A//CCL21//KCNH1//KCNMA1//PMP22//ABCC9//KCNMB4//NCALD//TMOD2//BCHE//KCTD12//PDK4//SORBS1//CAB39L//IL6ST//APOD//ANK2//CASQ2//DMD//PLN//SELP//TRIM9//ZDHHC15//VCAM1//ASPA//PPAP2B//SFRP1//CHRDL1//MYOCD//RORB//WASF3//NNAT//OXCT1//LAMA2//KANK2//EDA2R//ARHGEF26//TEAD1//PTGIS//SLIT3//SLIT2//RGS22//MPDZ//HIPK3//CILP//PDE8B//TIPARP//ARID5B//NFATC2//PRNP//CRYAB//TSPYL5//TCF21//DARC//VDAC2 |
| GO:0044700 | single organism signaling | Biological process | 185 | 4704 | 428 | 14747 | 1.3550781001653 | 4.05350961155281e-07 | 5.68564333455746e-05 | 6.39216879286025 | CAV1//FGFR1//MEF2C//MAPK4//SCG2//DOK5//FGF2//LPAR1//FGF10//GHR//KIT//SPRY1//FCER1A//CLU//BVES//COLEC12//CD44//FRZB//FZD4//ZRANB1//APCDD1//ANXA1//BMX//C3//CD34//CNTFR//RCAN1//EPAS1//ERG//FGF7//GEM//GNAL//GNG11//IGF1//IGFBP4//MFAP4//PPP1R12A//NR4A2//OPHN1//PDE1A//PRKAR2B//RLN1//CXCL12//SHB//TEK//NR2F1//TNXB//SPARCL1//RGS5//PTPLA//CAP2//IQGAP2//PDE7B//KCNIP1//SPA17//SLC44A2//SMOC2//CNTNAP3//PPP1R1C//KDR//MUSK//NTRK2//ROR1//ANGPTL1//PLCB4//DCLK1//TENC1//PREX2//ASB5//DCDC1//ERRFI1//NLGN1//TSPYL2//CD36//EDNRB//GFRA1//KLRB1//LIFR//KLRG1//CD160//GNG7//ANGPT1//EFEMP1//PIK3C2A//PIK3R1//CCNC//SMAD9//TGFBR2//TGFBR3//FMOD//PTN//PTPRD//BAI3//CHRM2//PTGER3//PTGFR//VIP//AKAP12//GPR64//LPHN3//CCRL1//ELTD1//CALCRL//GLP2R//PTHLH//BDKRB1//PPAP2A//CACNB4//CPE//CNTN1//FOXC1//CDON//BOC//ITGA9//ITGA8//ADAMTS1//RCAN2//A2M//ARHGAP6//ITSN1//DIRAS3//DLC1//RAB9B//RHOJ//RERGL//ARHGAP28//RABL3//KSR1//CTNNAL1//SH2D1A//CCL21//KCNH1//KCNMA1//PMP22//ABCC9//KCNMB4//NCALD//TMOD2//BCHE//KCTD12//PDK4//SORBS1//CAB39L//IL6ST//APOD//ANK2//CASQ2//DMD//PLN//SELP//TRIM9//ZDHHC15//VCAM1//ASPA//PPAP2B//SFRP1//CHRDL1//MYOCD//RORB//WASF3//NNAT//OXCT1//LAMA2//KANK2//EDA2R//ARHGEF26//TEAD1//PTGIS//SLIT3//SLIT2//RGS22//MPDZ//HIPK3//CILP//PDE8B//TIPARP//ARID5B//NFATC2//PRNP//CRYAB//TSPYL5//TCF21//DARC//VDAC2 |
| GO:0048514 | blood vessel morphogenesis | Biological process | 33 | 438 | 428 | 14747 | 2.59597042632185 | 5.63137958285423e-07 | 7.67315692303766e-05 | 6.2493851980749 | APOD//CAV1//EPAS1//FGF10//FGFR1//KDR//MEOX2//SHB//TEK//SCG2//CALCRL//APOLD1//CXCL12//TGFBR2//FOXC1//NTRK2//FZD4//TIPARP//HEG1//MYOCD//ANGPT1//PARVA//FGF2//SLIT2//BAI3//SERPINF1//SFRP1//AQP1//C3//CD34//CMA1//PTGIS//TCF21 |
| GO:0060548 | negative regulation of cell death | Biological process | 42 | 637 | 428 | 14747 | 2.27179829516278 | 5.94949261249579e-07 | 7.88142507472012e-05 | 6.22552007040409 | ANGPT1//ANXA1//AQP1//CD44//CLU//CRYAB//EDNRB//FGF10//FGFR1//FOXC1//IGF1//IL6ST//KDR//MEF2C//PIK3R1//PRNP//PTGFR//CXCL12//SFRP1//TEK//VIP//SCG2//NME5//HIPK3//FAIM2//TAF9B//OSR1//MYOCD//KIT//KANK2//CNTFR//NTRK2//NR4A2//ITSN1//CD34//GPAM//GHR//CCL21//CAV1//PDK4//CST3//FGF2 |
| GO:0007165 | signal transduction | Biological process | 168 | 4196 | 428 | 14747 | 1.37953814491772 | 6.8577056467149e-07 | 8.83902654842794e-05 | 6.16382116004215 | CAV1//FGFR1//MEF2C//MAPK4//SCG2//DOK5//FGF2//LPAR1//FGF10//GHR//KIT//CLU//COLEC12//CD44//FRZB//FZD4//ZRANB1//APCDD1//KDR//MUSK//NTRK2//ROR1//TEK//ANGPTL1//BMX//PLCB4//PRKAR2B//DCLK1//TENC1//PREX2//ASB5//DCDC1//ERRFI1//TSPYL2//ANXA1//CD36//EDNRB//GEM//GFRA1//KLRB1//LIFR//KLRG1//CD160//GNG7//ANGPT1//EFEMP1//PDE1A//PIK3C2A//PIK3R1//SPRY1//CCNC//SMAD9//TGFBR2//TGFBR3//FMOD//PTN//PTPRD//BAI3//C3//CHRM2//GNG11//PTGER3//PTGFR//CXCL12//VIP//AKAP12//GPR64//LPHN3//CCRL1//ELTD1//CALCRL//GLP2R//GNAL//PTHLH//BDKRB1//PPAP2A//CACNB4//CPE//CNTN1//FOXC1//CDON//BOC//ITGA9//ITGA8//ADAMTS1//RCAN1//RCAN2//FCER1A//A2M//ARHGAP6//OPHN1//ITSN1//DIRAS3//DLC1//IQGAP2//RAB9B//RHOJ//RERGL//ARHGAP28//RABL3//IGF1//KSR1//CTNNAL1//RGS5//KCTD12//FGF7//PDK4//SORBS1//CAB39L//IL6ST//APOD//NLGN1//ANK2//CASQ2//DMD//PLN//SELP//VCAM1//PPAP2B//SFRP1//CHRDL1//MYOCD//NR4A2//RORB//NR2F1//KANK2//EDA2R//ARHGEF26//TEAD1//PTGIS//SLIT3//SLIT2//RGS22//CCL21//SLC44A2//IGFBP4//TNXB//HIPK3//CILP//TMOD2//TIPARP//ARID5B//NFATC2//PRNP//CRYAB//TSPYL5//TCF21//DARC//CNTFR//VDAC2//CD34//EPAS1//ERG//MFAP4//PPP1R12A//RLN1//SHB//SPARCL1//PTPLA//CAP2//PDE7B//KCNIP1//SPA17//SMOC2//CNTNAP3//PPP1R1C |
| GO:0007420 | brain development | Biological process | 36 | 507 | 428 | 14747 | 2.4465520101753 | 7.0893135151165e-07 | 8.89708846147121e-05 | 6.14939581714855 | CNTN1//PTPRS//FAIM2//FOXP2//DLC1//NME5//MYH10//AQP1//NFIB//FGF2//SLIT2//FGFR1//IGF1//FGF10//NR4A2//NTRK2//TACC1//CDON//NR2F1//DCLK1//CMA1//SMAD9//FZD4//APOD//CLN5//CST3//FOXC1//FRZB//NNAT//OXCT1//CXCL12//SEPP1//SFRP1//TGFBR2//PCDH18//CADM2 |
| GO:0048468 | cell development | Biological process | 76 | 1496 | 428 | 14747 | 1.75041856164726 | 7.70581682123348e-07 | 9.40135627704832e-05 | 6.11318131902832 | EDNRB//TGFBR3//HEG1//PTHLH//CXCL12//KIT//NME5//ANK2//CACNB4//CNTN1//DPYSL3//FGFR1//GFRA1//ITGA9//LAMA2//MYH10//MYH11//OPHN1//PRNP//SLIT3//TRPC1//SLIT2//MYL9//CAP2//BOC//MUSK//DMD//SFRP1//FRZB//MYOCD//NTRK2//LPAR1//PMP22//WASF3//APOD//FOXC1//RDH10//IGF1//MAP1B//NR4A2//DCLK1//NFIB//ASPA//MEF2C//TMOD1//ACTC1//TTN//PRICKLE2//TEK//FZD4//BVES//PARVA//FGF10//LDB2//RORB//CLN5//NLGN1//PBX1//ZEB1//CDON//FGF2//EPAS1//KCNMA1//KDR//CCL21//IL6ST//RCAN1//FLNC//WEE1//SERPINF1//PTPRD//CST3//CRYAB//OSR1//ACTA2//ARID5B |
| GO:0051716 | cellular response to stimulus | Biological process | 196 | 5102 | 428 | 14747 | 1.32365720608008 | 7.88539004155867e-07 | 9.40135627704832e-05 | 6.10317681993721 | CAV1//FGFR1//MEF2C//MAPK4//SCG2//DOK5//FGF2//LPAR1//FGF10//GHR//KIT//MYOCD//CLU//COLEC12//CCL21//SLIT2//CD44//FRZB//FZD4//ZRANB1//APCDD1//ANXA1//BMX//C3//CD34//CNTFR//RCAN1//EPAS1//ERG//FGF7//GEM//GNAL//GNG11//IGF1//IGFBP4//MFAP4//PPP1R12A//NR4A2//OPHN1//PDE1A//PRKAR2B//RLN1//CXCL12//SHB//TEK//NR2F1//TNXB//SPARCL1//RGS5//PTPLA//CAP2//IQGAP2//PDE7B//KCNIP1//SPA17//SLC44A2//SMOC2//CNTNAP3//PPP1R1C//KDR//MUSK//NTRK2//ROR1//ANGPTL1//PLCB4//DCLK1//TENC1//PREX2//ASB5//DCDC1//ERRFI1//EYA4//CETN2//GTF2H5//ALDH1A1//FMO1//GSTA2//GSTM5//MAT2A//PTGIS//PAPSS2//NFATC2//SPDYA//TSPYL2//CD36//EDNRB//GFRA1//KLRB1//LIFR//KLRG1//CD160//GNG7//ANGPT1//EFEMP1//PIK3C2A//PIK3R1//SPRY1//CCNC//SMAD9//TGFBR2//TGFBR3//FMOD//PTN//PTPRD//BAI3//CHRM2//PTGER3//PTGFR//VIP//AKAP12//GPR64//LPHN3//CCRL1//ELTD1//CALCRL//GLP2R//PTHLH//BDKRB1//PPAP2A//CACNB4//CPE//CNTN1//FOXC1//CDON//BOC//ITGA9//ITGA8//ADAMTS1//RCAN2//FCER1A//A2M//ARHGAP6//ITSN1//DIRAS3//DLC1//RAB9B//RHOJ//RERGL//ARHGAP28//RABL3//KSR1//CTNNAL1//KCTD12//PDK4//SORBS1//CAB39L//IL6ST//SFRP1//TEX15//APOD//NLGN1//ANK2//CASQ2//DMD//PLN//SELP//VCAM1//SOD3//REV3L//PDGFD//AQP1//SRPX//PPAP2B//CHRDL1//RORB//SLIT3//KANK2//EDA2R//ARHGEF26//TEAD1//RGS22//GPX3//HIPK3//CILP//TMOD2//TIPARP//ARID5B//PRNP//CRYAB//TSPYL5//TCF21//DARC//CST3//PLSCR4//OXCT1//MT1A//FOSB//FABP4//OSR1//TIMP3//CMA1//DPYSL3//PARVA//FBLN5//VDAC2 |
| GO:0007154 | cell communication | Biological process | 187 | 4814 | 428 | 14747 | 1.33842928918381 | 8.34330630725814e-07 | 9.70468970227172e-05 | 6.07866181189861 | CAV1//FGFR1//MEF2C//MAPK4//SCG2//DOK5//FGF2//LPAR1//FGF10//GHR//KIT//MYOCD//SPRY1//FCER1A//CLU//COLEC12//CD44//FRZB//FZD4//ZRANB1//APCDD1//ANXA1//BMX//C3//CD34//CNTFR//RCAN1//EPAS1//ERG//FGF7//GEM//GNAL//GNG11//IGF1//IGFBP4//MFAP4//PPP1R12A//NR4A2//OPHN1//PDE1A//PRKAR2B//RLN1//CXCL12//SHB//TEK//NR2F1//TNXB//SPARCL1//RGS5//PTPLA//CAP2//IQGAP2//PDE7B//KCNIP1//SPA17//SLC44A2//SMOC2//CNTNAP3//PPP1R1C//KDR//MUSK//NTRK2//ROR1//ANGPTL1//PLCB4//DCLK1//TENC1//PREX2//ASB5//DCDC1//ERRFI1//NLGN1//TSPYL2//CD36//EDNRB//GFRA1//KLRB1//LIFR//KLRG1//CD160//GNG7//ANGPT1//EFEMP1//PIK3C2A//PIK3R1//CCNC//SMAD9//TGFBR2//TGFBR3//FMOD//PTN//PTPRD//BAI3//CHRM2//PTGER3//PTGFR//VIP//AKAP12//GPR64//LPHN3//CCRL1//ELTD1//CALCRL//GLP2R//PTHLH//BDKRB1//PPAP2A//CACNB4//CPE//CNTN1//FOXC1//CDON//BOC//ITGA9//ITGA8//ADAMTS1//RCAN2//A2M//ARHGAP6//ITSN1//DIRAS3//DLC1//RAB9B//RHOJ//RERGL//ARHGAP28//RABL3//KSR1//CTNNAL1//SH2D1A//CCL21//KCNH1//KCNMA1//PMP22//ABCC9//KCNMB4//NCALD//TMOD2//BCHE//KCTD12//PDK4//SORBS1//CAB39L//IL6ST//SFRP1//APOD//CASQ2//ANK2//DMD//PLN//SELP//TRIM9//ZDHHC15//VCAM1//ASPA//PPAP2B//CHRDL1//RORB//WASF3//NNAT//OXCT1//LAMA2//KANK2//EDA2R//ARHGEF26//TEAD1//PTGIS//SLIT3//SLIT2//RGS22//MPDZ//HIPK3//CILP//PDE8B//TIPARP//ARID5B//NFATC2//PRNP//CRYAB//TSPYL5//TCF21//DARC//VDAC2//FREM1//PXDC1 |
| GO:0018108 | peptidyl-tyrosine phosphorylation | Biological process | 20 | 201 | 428 | 14747 | 3.42841865439159 | 1.64993171672152e-06 | 0.000187345818024879 | 5.78253402891621 | ERRFI1//KDR//IL6ST//KIT//GHR//IGF1//CAV1//PDGFD//ANGPT1//CD36//CD44//FCER1A//FGF7//FGF10//PPAP2B//SFRP1//EFEMP1//FGFR1//TEK//WEE1 |
| GO:0009888 | tissue development | Biological process | 67 | 1288 | 428 | 14747 | 1.79233361873803 | 1.74033744367811e-06 | 0.000193015564393044 | 5.75936653578708 | MGP//CXCL12//TGFBR2//CD44//FGF2//PBX1//TCF21//EDNRB//TGFBR3//SFRP1//DLC1//HEG1//LDB2//APCDD1//MEF2C//FGFR1//NFIB//OSR1//CD34//KDR//PTHLH//BMX//CAV1//COL19A1//MEOX2//FOXP2//KCNH1//CDON//CACNB4//MUSK//DMD//EMP1//FGF7//UGCG//EDA2R//CST3//MYOCD//FOXC1//RDH10//FRZB//IGF1//ANXA1//PTN//FGF10//HTN3//EFEMP1//APOD//GATM//ERRFI1//APOLD1//BOC//GCNT4//PLN//MYH11//TTN//RCAN1//ITGA8//TIPARP//SLIT2//SMAD9//CHRDL2//ACTC1//MYH10//GHR//OXCT1//ARID5B//ACTA2 |
| GO:0018212 | peptidyl-tyrosine modification | Biological process | 20 | 203 | 428 | 14747 | 3.39464113070301 | 1.9258628569989e-06 | 0.000205048337377221 | 5.71537464274069 | ERRFI1//EFEMP1//FGFR1//KDR//KIT//TEK//WEE1//IL6ST//GHR//IGF1//CAV1//PDGFD//ANGPT1//CD36//CD44//FCER1A//FGF7//FGF10//PPAP2B//SFRP1 |
| GO:0042221 | response to chemical stimulus | Biological process | 120 | 2786 | 428 | 14747 | 1.48408933854855 | 1.93482390060284e-06 | 0.000205048337377221 | 5.71335855647284 | APOD//CAV1//GNAL//NR4A2//CCL21//KIT//SLIT2//KCNMB4//KCNIP1//ALDH1A1//FMO1//GSTA2//GSTM5//MAT2A//PTGIS//PAPSS2//FGF2//CXCL12//CCRL1//GPX3//TSPYL2//CCNC//SMAD9//TGFBR2//TGFBR3//FMOD//ANK2//CACNB4//CNTN1//DPYSL3//FGFR1//GFRA1//ITGA9//LAMA2//MYH10//MYH11//OPHN1//PRNP//SLIT3//TRPC1//MYL9//CAP2//BOC//GATM//OXCT1//VCAM1//LIPG//FGF7//FGF10//PDK4//PIK3C2A//PIK3R1//SORBS1//CAB39L//PDE1A//PRKAR2B//ACTC1//ANXA1//AQP1//CST3//NFATC2//SFRP1//WFDC1//EPHX1//SNRPN//NNAT//GPAM//GHR//COLEC12//AMPD1//CYBRD1//EDNRB//TAF9B//AOC3//CD44//IL6ST//LIFR//SOD3//PDGFD//ITGA8//MYOCD//FZD4//CRYAB//BDKRB1//CTSG//PTGFR//SELP//EDA2R//KCNMA1//AFF3//CD36//MEF2C//KDR//SPRY1//EPAS1//RCAN1//SCG2//ANGPT1//PTGDS//TTN//CLU//DARC//CNTFR//PLSCR4//MT1A//FOSB//FABP4//OSR1//TIMP3//CASQ2//CALCRL//CMA1//GNG7//GNG11//LPAR1//PARVA//NFIB//DMD//CD34//FBLN5 |
| GO:0032101 | regulation of response to external stimulus | Biological process | 29 | 382 | 428 | 14747 | 2.61573983461369 | 2.39054910751071e-06 | 0.00024783758029823 | 5.62150233044472 | C3//FCER1A//A2M//ANO6//ANXA1//IL6ST//SLIT2//SELP//CAV1//CD36//CD34//CFH//PTGER3//TGFBR2//CMA1//PTGIS//TEK//CALCRL//FABP4//PLA2G2A//CCL21//KDR//FGF10//CXCL12//SCG2//FGF2//LPAR1//APOD//ANKRD42 |
| GO:0010810 | regulation of cell-substrate adhesion | Biological process | 14 | 107 | 428 | 14747 | 4.50821032404577 | 2.52812463889951e-06 | 0.000256523966019399 | 5.59720151873936 | PIK3R1//CD36//CCL21//SFRP1//ECM2//VIT//ABI3BP//SMOC2//CCDC80//FZD4//KDR//TEK//APOD//ARHGAP6 |
| GO:0044763 | single-organism cellular process | Biological process | 325 | 9709 | 428 | 14747 | 1.15337031928175 | 2.84122762886415e-06 | 0.000282287803376107 | 5.54649397078272 | WEE1//CCNC//DIRAS3//SPDYA//CETN2//PRKAR2B//CAV1//FGFR1//MEF2C//MAPK4//SCG2//DOK5//FGF2//LPAR1//FGF10//GHR//KIT//TACC1//MYH10//MGP//KCNMB4//SPA17//EMP1//TGFBR3//IGFBP4//FBLN5//MYOCD//FOXC1//KDR//NTRK2//TGFBR2//FZD4//TIPARP//HEG1//PTPRD//SELP//VCAM1//NLGN1//CADM3//CAPN6//MAP1B//SFRP1//CXCL12//CDON//EDNRB//SPRY1//NR4A2//NR2F1//DCLK1//CCL21//CST3//FCER1A//CLU//SRPX//PIK3R1//CD36//SLIT2//NFIB//OSR1//PTHLH//COLEC12//A2M//CFD//F13A1//IGF1//TTN//MMRN1//BDKRB1//NME5//TSPYL2//TSPYL5//DPYSL3//OPHN1//TNXB//DLC1//FERMT2//DAAM2//RHOJ//DES//SGCD//CAP2//WASF3//ZRANB1//SH3D19//ACTC1//BMX//ITSN1//SHB//HIPK3//FAIM2//KANK2//PRUNE2//CNTN1//COL19A1//DPT//ITGA9//LAMA2//LSAMP//MFAP4//OMD//CNTN3//PGM5//PTPRS//AOC3//CTNNAL1//MPDZ//SPON1//SUSD5//CNTNAP3//SVEP1//BOC//CPXM2//CADM2//NEGR1//CD44//FRZB//APCDD1//ANXA1//C3//CD34//CNTFR//RCAN1//EPAS1//ERG//FGF7//GEM//GNAL//GNG11//PPP1R12A//PDE1A//RLN1//TEK//SPARCL1//RGS5//PTPLA//IQGAP2//PDE7B//KCNIP1//SLC44A2//SMOC2//PPP1R1C//MUSK//ROR1//ANGPTL1//PLCB4//TENC1//PREX2//ASB5//DCDC1//ERRFI1//CASQ2//EYA4//GTF2H5//AKAP12//SEC63//ALDH1A1//FMO1//GSTA2//GSTM5//MAT2A//PTGIS//PAPSS2//ANK2//CACNB4//PRNP//CYBRD1//AQP1//PIK3C2A//RAB9B//EXOC6//PLAGL1//LYVE1//NFATC2//CLN5//TNS1//PTP4A1//PYHIN1//CAB39L//TEX15//FREM1//PXDC1//CDH5//PCDH9//CDH19//PCDH18//FAT4//NCAM2//ECM2//ITGA8//SORBS1//ANGPT1//JAM2//PARVA//GFRA1//KLRB1//LIFR//KLRG1//CD160//GNG7//EFEMP1//SMAD9//FMOD//PTN//BAI3//CHRM2//PTGER3//PTGFR//VIP//GPR64//LPHN3//CCRL1//ELTD1//CALCRL//GLP2R//PPAP2A//CPE//ADAMTS1//RCAN2//ARHGAP6//RERGL//ARHGAP28//RABL3//KSR1//SH2D1A//KCNH1//KCNMA1//PMP22//ABCC9//NCALD//TMOD2//BCHE//MYH11//SLIT3//TRPC1//MYL9//DMD//CRYAB//IL6ST//TAF9B//C7//KCTD12//PDK4//PPAP2B//BVES//PALMD//PLSCR4//ANO6//CBLN4//APOD//PLA2G2A//ARID5B//VIT//ABI3BP//CCDC80//PLN//RDH10//SLC22A17//TRIM9//ZDHHC15//ZNF738//COL14A1//GPC6//SOD3//REV3L//ASPA//CMA1//CTSG//PDGFD//TMOD1//TPM2//TLL1//CHRDL2//RBM11//BEX1//EDA2R//TTLL7//APOLD1//CHRDL1//SEMA3D//IGSF10//NNAT//FBLN1//RECK//GLT25D2//CRISPLD2//DYRK3//WFDC1//NEXN//PODN//RORB//TCF21//EPB41L2//CNN1//CNN3//PRICKLE2//OXCT1//VDAC2//FLNC//TOR1AIP1//LDB2//ARHGEF26//TEAD1//RGS22//GPAM//GPX3//MFAP5//CILP//SYNM//ADAMTS9//PBX1//ZEB1//PDE8B//RANBP3L//SERPINF1//FABP4//ABCD2//SLCO2A1//ABCA8//MFSD4//SLC9A9//DARC//MT1A//FOSB//TIMP3//ACTA2 |
| GO:0016477 | cell migration | Biological process | 49 | 851 | 428 | 14747 | 1.98393039524693 | 3.40096036700642e-06 | 0.000331003673270482 | 5.46839842907601 | CXCL12//EDNRB//FGFR1//MYH10//NTRK2//NR4A2//NR2F1//DCLK1//FGF2//KDR//SLIT2//CCL21//KIT//BDKRB1//SELP//OPHN1//FOXC1//PPAP2A//PPAP2B//ANGPT1//TEK//FGF10//TGFBR3//TGFBR2//TNS1//ARID5B//IGF1//LAMA2//NEXN//PIK3R1//PTP4A1//DPYSL3//SFRP1//DLC1//PODN//ITGA9//SCG2//CAV1//CD34//JAM2//VCAM1//FGF7//PARVA//LPAR1//APOD//GFRA1//NFATC2//GPC6//ZRANB1 |
| GO:0007166 | cell surface receptor signaling pathway | Biological process | 104 | 2349 | 428 | 14747 | 1.52549305132826 | 3.62427390250774e-06 | 0.000345683244821188 | 5.44077898818821 | CAV1//CD44//FRZB//FZD4//ZRANB1//APCDD1//KDR//MUSK//NTRK2//ROR1//TEK//ANGPTL1//DOK5//ERRFI1//GNG7//ANGPT1//EFEMP1//PDE1A//PIK3C2A//PIK3R1//PRKAR2B//SPRY1//CCNC//SMAD9//TGFBR2//TGFBR3//FMOD//PTN//PTPRD//BAI3//C3//CHRM2//LPAR1//GNG11//PTGER3//PTGFR//CXCL12//VIP//AKAP12//GPR64//LPHN3//CCRL1//ELTD1//PREX2//CALCRL//GLP2R//GNAL//PTHLH//EDNRB//BDKRB1//PPAP2A//CACNB4//CPE//CNTN1//FOXC1//CDON//BOC//ITGA9//ITGA8//ADAMTS1//MEF2C//RGS5//KCTD12//FGF2//FGF7//FGF10//FGFR1//PDK4//SORBS1//CAB39L//IL6ST//APOD//KIT//LIFR//VCAM1//PPAP2B//SFRP1//CHRDL1//MYOCD//EDA2R//SLIT3//SLIT2//GFRA1//RGS22//IGF1//IGFBP4//CILP//TMOD2//TIPARP//ARID5B//GHR//ITSN1//NFATC2//PRNP//DARC//CNTFR//PLN//NLGN1//ANXA1//CD36//GEM//KLRB1//KLRG1//CD160 |
| GO:0040011 | locomotion | Biological process | 65 | 1266 | 428 | 14747 | 1.76904777723642 | 3.9486827355484e-06 | 0.000364094565354139 | 5.40354775908084 | SPA17//CXCL12//EDNRB//FGFR1//MYH10//NTRK2//NR4A2//NR2F1//DCLK1//FGF2//KDR//SLIT2//CCL21//KIT//BDKRB1//SELP//OPHN1//CCRL1//ANK2//CACNB4//CNTN1//DPYSL3//GFRA1//ITGA9//LAMA2//MYH11//PRNP//SLIT3//TRPC1//MYL9//CAP2//BOC//FOXC1//PPAP2A//PPAP2B//ANGPT1//TEK//FGF10//TGFBR3//TGFBR2//TNS1//ARID5B//IGF1//NFATC2//GPC6//ZRANB1//NEXN//PIK3R1//PTP4A1//SFRP1//DLC1//PODN//FZD4//BVES//TTN//SCG2//CD34//CAV1//JAM2//VCAM1//FGF7//PARVA//LPAR1//NFIB//APOD |
| GO:0003013 | circulatory system process | Biological process | 27 | 350 | 428 | 14747 | 2.65800400534045 | 3.96999735760437e-06 | 0.000364094565354139 | 5.40120978229941 | PLN//ANK2//CASQ2//DMD//EPAS1//HEG1//CORIN//CAV1//RCAN1//MEOX2//CXCL12//CHRM2//DES//ACTA2//CD34//EDNRB//VIP//PIK3C2A//KCNMB4//TEK//ANGPT1//SLIT2//BDKRB1//FOXC1//ACTC1//TTN//KCNMA1 |
| GO:0014706 | striated muscle tissue development | Biological process | 25 | 310 | 428 | 14747 | 2.77867802230932 | 4.16483656309043e-06 | 0.000374756708856194 | 5.38040203654786 | CAV1//COL19A1//MEF2C//MEOX2//FOXP2//KCNH1//CDON//CACNB4//MUSK//MYOCD//TCF21//DMD//IGF1//BOC//PLN//MYH11//TTN//RCAN1//ACTC1//MYH10//FOXC1//TGFBR3//TGFBR2//FGF2//FGFR1 |
| GO:0048869 | cellular developmental process | Biological process | 122 | 2891 | 428 | 14747 | 1.4540242518677 | 4.3838383141233e-06 | 0.000387157868889889 | 5.35814547199152 | CAV1//FOXC1//KDR//NTRK2//TGFBR2//FZD4//TIPARP//HEG1//MYOCD//MEF2C//SFRP1//CDON//EDNRB//FGF2//FGF10//FGFR1//SPRY1//MYH10//NR4A2//NR2F1//DCLK1//TGFBR3//NFIB//OSR1//PTHLH//KIT//CXCL12//NME5//ANK2//CACNB4//CNTN1//DPYSL3//GFRA1//ITGA9//LAMA2//MYH11//OPHN1//PRNP//SLIT3//TRPC1//SLIT2//MYL9//CAP2//BOC//KCNH1//MUSK//DMD//DLC1//WASF3//FERMT2//BVES//PALMD//PARVA//RHOJ//IGF1//FRZB//CD36//PLA2G2A//LPAR1//PMP22//APOD//RDH10//MAP1B//FAIM2//CLN5//TACC1//ASPA//ZRANB1//SH3D19//ANGPT1//COL19A1//MGP//SHB//TLL1//CHRDL2//RBM11//BEX1//EDA2R//TTLL7//APOLD1//CHRDL1//SEMA3D//IGSF10//NNAT//PTPRD//PIK3R1//VCAM1//ANXA1//EPAS1//DYRK3//EXOC6//TMOD1//ACTC1//TTN//TCF21//PRICKLE2//EFEMP1//TEK//LDB2//RORB//KCNMA1//ARID5B//GHR//SMAD9//ERRFI1//ADAMTS9//NLGN1//PBX1//ZEB1//IL6ST//CCL21//RCAN1//FLNC//WEE1//A2M//SERPINF1//FABP4//CST3//CD34//CRYAB//ACTA2//ITGA8 |
| GO:0007167 | enzyme linked receptor protein signaling pathway | Biological process | 50 | 886 | 428 | 14747 | 1.94444737452796 | 4.71124698440989e-06 | 0.000408507943066378 | 5.32686412752686 | KDR//MUSK//NTRK2//ROR1//TEK//ANGPTL1//DOK5//ERRFI1//GNG7//ANGPT1//EFEMP1//PDE1A//PIK3C2A//PIK3R1//PRKAR2B//SPRY1//CCNC//SMAD9//TGFBR2//TGFBR3//FMOD//PTN//PTPRD//FGF2//FGF7//FGF10//FGFR1//PDK4//SORBS1//CAB39L//APOD//CHRDL1//ITGA8//MYOCD//CAV1//SFRP1//FZD4//GFRA1//KIT//IGF1//IGFBP4//CILP//TIPARP//ARID5B//GHR//FOXC1//MEF2C//ITSN1//IL6ST//LIFR |
| GO:0030154 | cell differentiation | Biological process | 116 | 2718 | 428 | 14747 | 1.47051157736929 | 4.8951537550272e-06 | 0.000416874790316513 | 5.31023366261239 | CAV1//FOXC1//KDR//NTRK2//TGFBR2//FZD4//TIPARP//HEG1//MYOCD//MEF2C//SFRP1//CDON//EDNRB//FGF2//FGF10//FGFR1//SPRY1//MYH10//NR4A2//NR2F1//DCLK1//TGFBR3//NFIB//OSR1//PTHLH//KIT//CXCL12//NME5//ANK2//CACNB4//CNTN1//DPYSL3//GFRA1//ITGA9//LAMA2//MYH11//OPHN1//PRNP//SLIT3//TRPC1//SLIT2//MYL9//CAP2//BOC//KCNH1//MUSK//DMD//IGF1//FRZB//CD36//PLA2G2A//LPAR1//PMP22//WASF3//APOD//RDH10//MAP1B//FAIM2//CLN5//TACC1//ASPA//NNAT//PTPRD//PIK3R1//VCAM1//ANXA1//EPAS1//DYRK3//EXOC6//TMOD1//ACTC1//TTN//TCF21//PRICKLE2//EFEMP1//TEK//BVES//PARVA//LDB2//RORB//KCNMA1//ARID5B//GHR//SMAD9//APOLD1//ERRFI1//ADAMTS9//NLGN1//PBX1//ZEB1//IL6ST//CCL21//RCAN1//FLNC//WEE1//A2M//SERPINF1//FABP4//CST3//CD34//CRYAB//ACTA2//ITGA8//ANGPT1//COL19A1//MGP//SHB//TLL1//CHRDL2//RBM11//BEX1//EDA2R//TTLL7//CHRDL1//SEMA3D//IGSF10 |
| GO:0009611 | response to wounding | Biological process | 58 | 1096 | 428 | 14747 | 1.82338068763217 | 5.6689425209178e-06 | 0.000457877584764855 | 5.2464979464267 | MYH10//C3//FCER1A//A2M//CD44//VCAM1//ANO6//ANXA1//IL6ST//ANGPT1//CAV1//CD36//CLU//CFD//F13A1//IGF1//KCNMA1//PDE1A//PIK3R1//PRKAR2B//SELP//TEK//TTN//WEE1//TFPI2//PAPSS2//MMRN1//KCNMB4//PLSCR4//JAM2//BDKRB1//DARC//IGFBP4//KIT//PTX3//CCL21//SCG2//AOC3//KLRG1//APOD//CD34//CFH//PTGER3//FGF2//FGF10//TGFBR2//GATM//CST3//DPYSL3//CMA1//PTGIS//CALCRL//FABP4//PLA2G2A//ANKRD42//FGF7//LYVE1 |
| GO:0030029 | actin filament-based process | Biological process | 33 | 487 | 428 | 14747 | 2.33477422326278 | 5.70431036412485e-06 | 0.000457877584764855 | 5.24379685311414 | DPYSL3//OPHN1//TNXB//DLC1//FERMT2//DAAM2//RHOJ//CXCL12//ACTC1//MYH10//ARHGAP6//WASF3//DES//DMD//TMOD1//TPM2//TTN//MYH11//SLIT2//CCL21//EPB41L2//AQP1//CNN1//CNN3//FGF7//FGF10//KIT//SORBS1//SFRP1//PARVA//ANK2//PLN//TEK |
| GO:0010811 | positive regulation of cell-substrate adhesion | Biological process | 10 | 58 | 428 | 14747 | 5.94062197873026 | 5.72641936754334e-06 | 0.000457877584764855 | 5.2421168501075 | CD36//CCL21//SFRP1//KDR//TEK//ECM2//VIT//ABI3BP//SMOC2//CCDC80 |
| GO:0051174 | regulation of phosphorus metabolic process | Biological process | 64 | 1254 | 428 | 14747 | 1.75849990311377 | 5.76067416353351e-06 | 0.000457877584764855 | 5.23952668873483 | CCNC//DIRAS3//FGF2//LPAR1//FGF10//GHR//KIT//CAV1//BDKRB1//PRNP//PPAP2B//SLIT2//C3//EDNRB//MUSK//TEK//CDON//ERRFI1//FABP4//TSPYL2//ANGPT1//GNAL//NTRK2//CAP2//FCER1A//PDK4//VIP//MEF2C//PTHLH//AKAP12//CALCRL//TGFBR2//BVES//ALDH1A1//DLC1//CNN3//PLN//SFRP1//PREX2//CCL21//DMD//CD44//PRKAR2B//SPRY1//PPP1R12A//IL6ST//IGF1//ARHGAP6//FGFR1//TPM2//TNXB//FZD4//HIPK3//RGS5//RGS22//PIK3R1//RCAN1//MYOCD//SPDYA//GNG7//TTN//PDGFD//CD36//FGF7 |
| GO:0022603 | regulation of anatomical structure morphogenesis | Biological process | 38 | 605 | 428 | 14747 | 2.16415385803661 | 6.57636161027841e-06 | 0.000514142106875701 | 5.18201431453267 | FGF2//FGF10//FGFR1//SPRY1//KDR//KIT//MYH10//DLC1//WASF3//FERMT2//BVES//PALMD//PARVA//RHOJ//SFRP1//TGFBR3//BAI3//SERPINF1//TEK//ZRANB1//SH3D19//CD44//CD34//TGFBR2//AQP1//C3//CMA1//PTGIS//MAP1B//NTRK2//SLIT2//PTPRD//CST3//FGF7//FOXP2//CD36//OSR1//NFIB |
| GO:0001822 | kidney development | Biological process | 19 | 203 | 428 | 14747 | 3.22490907416786 | 7.37158661709388e-06 | 0.000558017405982868 | 5.13243902708139 | FGF10//FOXC1//ITGA8//SLIT2//SPRY1//RDH10//OSR1//TCF21//GCNT4//CD34//ANGPT1//TEK//MEF2C//ACTA2//GFRA1//ADAMTS1//TENC1//TIPARP//ARID5B |
| GO:0061448 | connective tissue development | Biological process | 19 | 203 | 428 | 14747 | 3.22490907416786 | 7.37158661709388e-06 | 0.000558017405982868 | 5.13243902708139 | MGP//FGFR1//MEF2C//NFIB//OSR1//EFEMP1//PTHLH//CD44//SMAD9//TGFBR2//CHRDL2//GHR//FGF2//OXCT1//ARID5B//IGF1//FRZB//ACTA2//CD34 |
| GO:0030324 | lung development | Biological process | 16 | 151 | 428 | 14747 | 3.65092529553754 | 8.13215793490347e-06 | 0.000599001468598265 | 5.08979419550485 | IGF1//KDR//PTHLH//TCF21//ERRFI1//MYOCD//FOXP2//FGF10//RDH10//TGFBR2//FGFR1//NFIB//FGF7//EPAS1//FGF2//HEG1 |
| GO:0019220 | regulation of phosphate metabolic process | Biological process | 63 | 1241 | 428 | 14747 | 1.74915654393879 | 8.16420538034959e-06 | 0.000599001468598265 | 5.0880860786215 | CCNC//DIRAS3//FGF2//LPAR1//FGF10//GHR//KIT//CAV1//BDKRB1//PRNP//PPAP2B//SLIT2//C3//EDNRB//MUSK//TEK//CDON//ERRFI1//FABP4//TSPYL2//ANGPT1//GNAL//NTRK2//CAP2//FCER1A//VIP//MEF2C//PTHLH//AKAP12//CALCRL//TGFBR2//BVES//ALDH1A1//DLC1//CNN3//PLN//SFRP1//PREX2//CCL21//DMD//CD44//PRKAR2B//SPRY1//PPP1R12A//IL6ST//IGF1//ARHGAP6//FGFR1//TPM2//TNXB//FZD4//HIPK3//RGS5//RGS22//PIK3R1//RCAN1//MYOCD//SPDYA//GNG7//TTN//PDGFD//CD36//FGF7 |
| GO:0048286 | lung alveolus development | Biological process | 8 | 37 | 428 | 14747 | 7.4498610760293 | 8.68940079823044e-06 | 0.000619988697378135 | 5.0610101705012 | FGF10//IGF1//KDR//PTHLH//TCF21//ERRFI1//MYOCD//FOXP2 |
| GO:0048608 | reproductive structure development | Biological process | 22 | 263 | 428 | 14747 | 2.88221811591628 | 8.84026660132379e-06 | 0.000619988697378135 | 5.05353463750526 | FOXC1//KDR//KIT//ADAMTS1//FRZB//SFRP1//OSR1//RDH10//GFRA1//ARID5B//TIPARP//FGF10//TEX15//FZD4//TCF21//CST3//MYOCD//CD44//IGF1//SERPINF1//SLIT3//SLIT2 |
| GO:0061458 | reproductive system development | Biological process | 22 | 263 | 428 | 14747 | 2.88221811591628 | 8.84026660132379e-06 | 0.000619988697378135 | 5.05353463750526 | FOXC1//KDR//KIT//ADAMTS1//FRZB//SFRP1//OSR1//RDH10//GFRA1//ARID5B//TIPARP//FGF10//TEX15//FZD4//TCF21//CST3//MYOCD//CD44//IGF1//SERPINF1//SLIT3//SLIT2 |
| GO:0043069 | negative regulation of programmed cell death | Biological process | 38 | 614 | 428 | 14747 | 2.13243173308168 | 9.22196452414896e-06 | 0.00063738476544444 | 5.03517655279499 | ANGPT1//ANXA1//AQP1//CD44//CLU//CRYAB//EDNRB//FGF10//FGFR1//FOXC1//IGF1//IL6ST//KDR//MEF2C//PIK3R1//PRNP//PTGFR//CXCL12//SFRP1//TEK//VIP//SCG2//NME5//HIPK3//FAIM2//TAF9B//OSR1//MYOCD//CNTFR//NTRK2//NR4A2//ITSN1//GPAM//CCL21//CAV1//PDK4//KIT//KANK2 |
| GO:0006928 | cellular component movement | Biological process | 62 | 1221 | 428 | 14747 | 1.74958858603718 | 9.65759254541629e-06 | 0.000657957983558433 | 5.01513112146727 | SPA17//CXCL12//EDNRB//FGFR1//MYH10//NTRK2//NR4A2//NR2F1//DCLK1//FGF2//KDR//SLIT2//CCL21//KIT//BDKRB1//SELP//NME5//OPHN1//FOXC1//PPAP2A//PPAP2B//ACTC1//ANGPT1//TEK//FGF10//TGFBR3//TGFBR2//TNS1//ARID5B//IGF1//GFRA1//NFATC2//GPC6//ZRANB1//DES//DMD//TMOD1//TPM2//TTN//LAMA2//NEXN//PIK3R1//PTP4A1//DPYSL3//SFRP1//DLC1//PODN//ITGA9//SCG2//MAP1B//CD34//CAV1//JAM2//VCAM1//FGF7//PARVA//LPAR1//ANK2//PLN//APOD//ANXA1//LYVE1 |
| GO:0030323 | respiratory tube development | Biological process | 16 | 154 | 428 | 14747 | 3.5798033741959 | 1.04620765975389e-05 | 0.000702727370333282 | 4.98038210463293 | EPAS1//FGF2//HEG1//IGF1//KDR//PTHLH//TCF21//ERRFI1//MYOCD//FOXP2//FGF10//RDH10//TGFBR2//FGFR1//NFIB//FGF7 |
| GO:0008015 | blood circulation | Biological process | 26 | 349 | 428 | 14747 | 2.56689339367485 | 1.1172820232311e-05 | 0.000740044162331822 | 4.95183718885982 | PLN//ANK2//CASQ2//DMD//EPAS1//CORIN//CAV1//CHRM2//DES//ACTA2//CD34//EDNRB//VIP//PIK3C2A//KCNMB4//TEK//ANGPT1//SLIT2//BDKRB1//FOXC1//ACTC1//TTN//KCNMA1//RCAN1//MEOX2//CXCL12 |
| GO:0072012 | glomerulus vasculature development | Biological process | 6 | 19 | 428 | 14747 | 10.8807181505165 | 1.13557824063999e-05 | 0.000741859264330426 | 4.94478293779158 | CD34//ACTA2//OSR1//TCF21//ANGPT1//TEK |
| GO:0007417 | central nervous system development | Biological process | 41 | 692 | 428 | 14747 | 2.04144495165037 | 1.15430799381652e-05 | 0.000743904705744728 | 4.93767829675361 | APOD//CLN5//CST3//FOXC1//FRZB//NNAT//OXCT1//CXCL12//SEPP1//SFRP1//TGFBR2//FZD4//PCDH18//CADM2//WASF3//PTPRS//CNTN1//FAIM2//FOXP2//DLC1//NME5//MYH10//AQP1//NFIB//FGF2//SLIT2//FGFR1//IGF1//NR4A2//DCLK1//NTRK2//FGF10//TACC1//CDON//ASPA//NR2F1//CMA1//SMAD9//IL6ST//RCAN1//RCAN2 |
| GO:0065007 | biological regulation | Biological process | 295 | 8713 | 428 | 14747 | 1.1665791582242 | 1.25383733857289e-05 | 0.000797273369020548 | 4.90175880125833 | WEE1//CCNC//DIRAS3//CAV1//CD36//EDNRB//FGFR1//FOSB//MEF2C//NFIB//NFIX//ZEB1//TCF21//NR2F1//KANK2//TAF9B//MYOCD//OSR1//MAPK4//SCG2//DOK5//FGF2//LPAR1//FGF10//GHR//KIT//KCNMB4//PSTK//IGFBP4//FBLN5//SPRY1//ANXA1//C3//FCER1A//CLU//A2M//CD34//BVES//BDKRB1//PRNP//PPAP2B//SLIT2//MUSK//TEK//CDON//KDR//VIP//PIK3R1//CCL21//SFRP1//PLN//ANK2//CASQ2//DMD//EPAS1//TGFBR2//FOXP2//NLGN1//ANGPT1//RDH10//COLEC12//ANO6//IL6ST//SELP//CORIN//AQP1//CFD//F13A1//IGF1//KCNMA1//PDE1A//PRKAR2B//TTN//TFPI2//PAPSS2//MMRN1//PLSCR4//JAM2//C7//CFH//CD44//FRZB//FZD4//ZRANB1//APCDD1//BMX//CNTFR//RCAN1//ERG//FGF7//GEM//GNAL//GNG11//MFAP4//PPP1R12A//NR4A2//OPHN1//RLN1//CXCL12//SHB//TNXB//SPARCL1//RGS5//PTPLA//CAP2//IQGAP2//PDE7B//KCNIP1//SPA17//SLC44A2//SMOC2//CNTNAP3//PPP1R1C//NTRK2//ROR1//ANGPTL1//PLCB4//DCLK1//TENC1//PREX2//ASB5//DCDC1//ERRFI1//EYA4//EFEMP1//FOXC1//KCNH1//AFF3//NFATC2//RORB//ZNF43//PDE8B//PDLIM1//HIPK3//SSBP2//CCDC59//BNC2//ZNF331//TSPYL2//ZNF518B//ZFP3//ZNF300P1//ZNF280B//ZNF738//GTF2H5//ZNF876P//ZNF826P//LARP6//FABP4//CACNB4//CYBRD1//PDK4//SLC9A9//PLAGL1//DLC1//CALCRL//MYL9//CNN1//CLN5//CAB39L//GFRA1//KLRB1//LIFR//KLRG1//CD160//GNG7//PIK3C2A//SMAD9//TGFBR3//FMOD//PTN//PTPRD//BAI3//CHRM2//PTGER3//PTGFR//AKAP12//GPR64//LPHN3//CCRL1//ELTD1//GLP2R//PTHLH//ARHGAP6//PPAP2A//CPE//CNTN1//BOC//ITGA9//ITGA8//ADAMTS1//RCAN2//ITSN1//RAB9B//RHOJ//RERGL//ARHGAP28//RABL3//KSR1//CTNNAL1//DES//CRYAB//NME5//FAIM2//TIPARP//ACTA2//KCTD12//CST3//PBX1//TSPYL5//SPDYA//CDH5//DPT//PMP22//SLIT3//PODN//SORBS1//MYH10//WASF3//FERMT2//PALMD//PARVA//TEX15//APOD//LDB2//PLA2G2A//ECM2//VIT//ABI3BP//CCDC80//SERPINA6//SERPINF1//RECK//DPYSL3//LIPG//SLC22A17//TRIM9//EBF1//EBF3//ARID5B//VCAM1//ASPA//SH3D19//LAMA2//DYRK3//EXOC6//WFDC1//NEXN//PTP4A1//MGP//CHRDL1//HTN3//ABCD2//NNAT//OXCT1//PTGIS//VDAC2//ALDH1A1//MAP1B//RNF180//CETN2//DARC//CNN3//TACC1//EDA2R//CTSG//ARHGEF26//TEAD1//RGS22//GPAM//GCNT4//MPDZ//SRPX//ZNF462//TPM2//CILP//PTGDS//PTX3//APOLD1//ADAMTS9//TMOD2//CMA1//MT1A//FHL5//BEX1//SH2D1A//TRPC1//OGN//PDGFD//CD200//HEG1//TIMP3//ANKRD42//CAPN6//PARM1 |
| GO:0007548 | sex differentiation | Biological process | 21 | 251 | 428 | 14747 | 2.88274006776632 | 1.40867918234682e-05 | 0.00088394618692263 | 4.85118790341367 | FOXC1//KDR//KIT//ADAMTS1//SMAD9//FRZB//SFRP1//OSR1//RDH10//GFRA1//ARID5B//TIPARP//FGF10//TEX15//FZD4//TCF21//CST3//SLIT3//SLIT2//CNTFR//PBX1 |
| GO:0048870 | cell motility | Biological process | 50 | 925 | 428 | 14747 | 1.86246526900733 | 1.52540341054917e-05 | 0.000932647290372948 | 4.81661528693575 | SPA17//CXCL12//EDNRB//FGFR1//MYH10//NTRK2//NR4A2//NR2F1//DCLK1//FGF2//KDR//SLIT2//CCL21//KIT//BDKRB1//SELP//OPHN1//FOXC1//PPAP2A//PPAP2B//ANGPT1//TEK//FGF10//TGFBR3//TGFBR2//TNS1//ARID5B//IGF1//GFRA1//NFATC2//GPC6//ZRANB1//LAMA2//NEXN//PIK3R1//PTP4A1//DPYSL3//SFRP1//DLC1//PODN//ITGA9//SCG2//CAV1//CD34//JAM2//VCAM1//FGF7//PARVA//LPAR1//APOD |
| GO:0051674 | localization of cell | Biological process | 50 | 925 | 428 | 14747 | 1.86246526900733 | 1.52540341054917e-05 | 0.000932647290372948 | 4.81661528693575 | SPA17//CXCL12//EDNRB//FGFR1//MYH10//NTRK2//NR4A2//NR2F1//DCLK1//FGF2//KDR//SLIT2//CCL21//KIT//BDKRB1//SELP//OPHN1//FOXC1//PPAP2A//PPAP2B//ANGPT1//TEK//FGF10//TGFBR3//TGFBR2//TNS1//ARID5B//IGF1//GFRA1//NFATC2//GPC6//ZRANB1//LAMA2//NEXN//PIK3R1//PTP4A1//DPYSL3//SFRP1//DLC1//PODN//ITGA9//SCG2//CD34//CAV1//JAM2//VCAM1//FGF7//PARVA//LPAR1//APOD |
| GO:0061437 | renal system vasculature development | Biological process | 6 | 20 | 428 | 14747 | 10.3366822429907 | 1.58269657368651e-05 | 0.000943484994988871 | 4.80060233782529 | CD34//ANGPT1//TEK//ACTA2//OSR1//TCF21 |
| GO:0061440 | kidney vasculature development | Biological process | 6 | 20 | 428 | 14747 | 10.3366822429907 | 1.58269657368651e-05 | 0.000943484994988871 | 4.80060233782529 | CD34//ANGPT1//TEK//ACTA2//OSR1//TCF21 |
| GO:0001525 | angiogenesis | Biological process | 26 | 362 | 428 | 14747 | 2.47471213920587 | 2.11527434787379e-05 | 0.00124540041543335 | 4.67463329730041 | CXCL12//TGFBR2//ANGPT1//TEK//PARVA//FGF2//KDR//SLIT2//BAI3//SERPINF1//SFRP1//AQP1//C3//CD34//CMA1//PTGIS//APOD//CAV1//EPAS1//FGF10//FGFR1//MEOX2//SHB//SCG2//CALCRL//APOLD1 |
| GO:0050793 | regulation of developmental process | Biological process | 70 | 1479 | 428 | 14747 | 1.63075897455341 | 2.55678332047724e-05 | 0.00148698776284829 | 4.59230607553582 | FGF2//FGF10//FGFR1//SPRY1//TGFBR2//KDR//KIT//MYH10//DLC1//WASF3//FERMT2//BVES//PALMD//PARVA//RHOJ//MUSK//CST3//SFRP1//TGFBR3//FRZB//CD36//PLA2G2A//MYOCD//CNTN1//DMD//DPYSL3//NTRK2//LPAR1//PMP22//EDNRB//IGF1//BAI3//SERPINF1//TEK//ZRANB1//SH3D19//MGP//MEF2C//OSR1//CAV1//EFEMP1//PTHLH//CD44//LDB2//CD34//GHR//SMAD9//APOLD1//ERRFI1//ADAMTS9//BOC//NLGN1//PBX1//ZEB1//CDON//IL6ST//PIK3R1//AQP1//C3//CMA1//PTGIS//MAP1B//LAMA2//ASPA//SLIT2//PTPRD//RCAN1//FGF7//FOXP2//NFIB |
| GO:0051239 | regulation of multicellular organismal process | Biological process | 82 | 1822 | 428 | 14747 | 1.55069144516142 | 2.89603751552897e-05 | 0.00166400035079008 | 4.53819581654889 | FGF2//FGF10//FGFR1//SPRY1//PLN//ANK2//CASQ2//DMD//EPAS1//NLGN1//ANO6//TGFBR2//CAV1//CORIN//EDNRB//CALCRL//MYL9//CNN1//CHRM2//DES//MUSK//VIP//MYOCD//AQP1//SELP//C3//IL6ST//IGF1//TGFBR3//CST3//SFRP1//CNTN1//DPYSL3//NTRK2//LPAR1//PMP22//LIPG//BAI3//SERPINF1//TEK//KCNMB4//CD36//CD34//PBX1//MEF2C//MGP//OSR1//PTGER3//WASF3//LAMA2//EFEMP1//PTHLH//DARC//PRNP//CD44//GHR//GPAM//NR4A2//PTGDS//FCER1A//APOLD1//ERRFI1//BOC//ZEB1//CDON//PIK3R1//CMA1//KDR//PTGIS//MAP1B//FOXC1//ASPA//SLIT2//PTPRD//OPHN1//KCNMA1//FGF7//FOXP2//FRZB//APOD//ANKRD42//NFIB |
| GO:0001570 | vasculogenesis | Biological process | 10 | 70 | 428 | 14747 | 4.92222963951936 | 3.19198202601963e-05 | 0.00181220979548662 | 4.49593956281707 | CD34//CAV1//FOXC1//KDR//NTRK2//TGFBR2//FZD4//TIPARP//HEG1//MYOCD |
| GO:0008354 | germ cell migration | Biological process | 5 | 14 | 428 | 14747 | 12.3055740987984 | 3.24286631766744e-05 | 0.00181943876105365 | 4.48907095410101 | KIT//FOXC1//CXCL12//PPAP2A//PPAP2B |
| GO:0014068 | positive regulation of phosphatidylinositol 3-kinase cascade | Biological process | 8 | 44 | 428 | 14747 | 6.26465590484282 | 3.33877499166587e-05 | 0.00183018596956949 | 4.47641284814258 | ANGPT1//FGFR1//IGF1//KDR//KIT//NTRK2//SELP//TEK |
| GO:0060425 | lung morphogenesis | Biological process | 8 | 44 | 428 | 14747 | 6.26465590484282 | 3.33877499166587e-05 | 0.00183018596956949 | 4.47641284814258 | RDH10//FGF10//IGF1//TGFBR2//FGF7//FOXP2//NFIB//TCF21 |
| GO:0048518 | positive regulation of biological process | Biological process | 138 | 3513 | 428 | 14747 | 1.35350806483794 | 3.64004682258155e-05 | 0.00197265719282857 | 4.43889302990644 | FGF2//LPAR1//FGF10//GHR//KIT//FGFR1//SPRY1//C3//FCER1A//A2M//BVES//EDNRB//MUSK//TEK//CDON//KDR//VIP//SCG2//CD36//CCL21//SFRP1//TGFBR2//FOXP2//ANGPT1//COLEC12//MEF2C//ANO6//IL6ST//BDKRB1//SELP//MYOCD//C7//CLU//CFD//CFH//PLAGL1//DLC1//TSPYL2//GNAL//NTRK2//CAP2//CNTFR//CST3//FGF7//IGF1//LIFR//PBX1//PTHLH//PTN//GLP2R//TSPYL5//SPDYA//AQP1//CAV1//ANK2//CD34//CD44//CNTN1//OSR1//AKAP12//NLGN1//PLA2G2A//ECM2//VIT//ABI3BP//SMOC2//CCDC80//DMD//DPYSL3//LIPG//PIK3R1//SORBS1//EBF1//FOXC1//SMAD9//NFATC2//RORB//FZD4//EBF3//CACNB4//ZRANB1//TAF9B//PPP1R1C//PTP4A1//TGFBR3//ITGA8//CALCRL//ANXA1//PTGER3//WASF3//ABCD2//NNAT//OXCT1//LAMA2//RNF180//PRKAR2B//PTGIS//GPAM//VCAM1//FRZB//KCNMA1//SRPX//SLIT2//SLC44A2//IGFBP4//LDB2//PPAP2B//PTX3//ADAMTS9//BOC//ZEB1//TMOD2//CMA1//MAP1B//CCNC//EPAS1//PPP1R12A//NFIB//NFIX//NR4A2//TCF21//FHL5//BEX1//ZNF462//SH2D1A//EDA2R//ASPA//FABP4//SERPINF1//PTPRD//CTSG//PRNP//CXCL12//SH3D19//TRPC1//PDGFD//ITSN1//ANKRD42 |
| GO:0043066 | negative regulation of apoptotic process | Biological process | 36 | 606 | 428 | 14747 | 2.04686777088924 | 3.80997220460899e-05 | 0.00204154578020003 | 4.41907819267844 | MYOCD//IGF1//CNTFR//MEF2C//NTRK2//NR4A2//ITSN1//GPAM//CCL21//CXCL12//SFRP1//ANGPT1//KDR//TEK//CAV1//PDK4//ANXA1//AQP1//CD44//CLU//CRYAB//EDNRB//FGF10//FGFR1//FOXC1//IL6ST//PIK3R1//PRNP//PTGFR//VIP//SCG2//NME5//HIPK3//FAIM2//TAF9B//OSR1 |
| GO:0050673 | epithelial cell proliferation | Biological process | 19 | 229 | 428 | 14747 | 2.85876219238461 | 4.03386686152631e-05 | 0.00213750122917989 | 4.39427843985123 | CD34//TEK//CAV1//SCG2//FGF2//KDR//VIP//FGF10//EDNRB//FGF7//IGF1//SFRP1//OSR1//TGFBR3//WFDC1//FOXP2//SERPINF1//NFIB//MEF2C |
| GO:0008283 | cell proliferation | Biological process | 72 | 1557 | 428 | 14747 | 1.59332288909297 | 4.12929231524415e-05 | 0.0021546391847287 | 4.3841243720529 | CD34//TEK//CAV1//SCG2//FGF2//KDR//VIP//FGFR1//TGFBR2//FOXP2//CNTFR//CST3//EDNRB//FGF7//IGF1//IL6ST//KIT//LIFR//NTRK2//PBX1//PTHLH//PTN//SFRP1//GLP2R//TSPYL5//MYOCD//SPDYA//CDH5//DPT//FGF10//FRZB//PMP22//SLIT3//PPAP2A//SLIT2//ADAMTS1//SPRY1//DLC1//TENC1//KANK2//PODN//ANGPT1//TACC1//MEF2C//NFATC2//CXCL12//VCAM1//GPAM//ANXA1//IGFBP4//PRNP//AQP1//CALCRL//APOD//OGN//OSR1//TGFBR3//WFDC1//FOXC1//SRPX//SERPINF1//GPC3//CDON//NFIB//EMP1//ERG//MYH10//ZEB1//LIPG//PEMT//CD160//HDGFRP3 |
| GO:0060541 | respiratory system development | Biological process | 16 | 172 | 428 | 14747 | 3.20517278852423 | 4.15656961616775e-05 | 0.0021546391847287 | 4.38126494133451 | EPAS1//FGF2//HEG1//RDH10//IGF1//KDR//PTHLH//TCF21//ERRFI1//MYOCD//FOXP2//FGF10//TGFBR2//FGFR1//NFIB//FGF7 |
| GO:0001657 | ureteric bud development | Biological process | 12 | 103 | 428 | 14747 | 4.01424553125851 | 4.30112557745595e-05 | 0.00218446448656147 | 4.36641787741801 | CD44//FGF2//PBX1//TCF21//GPC3//FGFR1//FOXC1//SMAD9//SFRP1//SLIT2//SPRY1//OSR1 |
| GO:0009887 | organ morphogenesis | Biological process | 44 | 808 | 428 | 14747 | 1.87629545664847 | 4.30571737757975e-05 | 0.00218446448656147 | 4.36595448017574 | MGP//FGF2//FGF10//FGFR1//SPRY1//MEF2C//PTHLH//TGFBR3//DLC1//PARVA//HEG1//CPE//FGF7//TGFBR2//OSR1//RORB//ITGA8//SOBP//EYA4//FOXC1//AQP1//CD34//ERRFI1//NTRK2//TCF21//ARID5B//RDH10//TIPARP//ACTC1//TTN//CDON//SFRP1//TEK//ADAMTS1//GHR//CD44//IGF1//FOXP2//GCNT4//FRZB//FREM1//NFIB//PBX1//SLIT3 |
| GO:0006027 | glycosaminoglycan catabolic process | Biological process | 9 | 59 | 428 | 14747 | 5.25594012355457 | 4.66012311002179e-05 | 0.00233938180123094 | 4.33160261007032 | CD44//FGF2//LYVE1//FMOD//OMD//OGN//GPC5//GPC3//GPC6 |
| GO:0042127 | regulation of cell proliferation | Biological process | 58 | 1185 | 428 | 14747 | 1.68643479632478 | 5.53659899401808e-05 | 0.00275042089609086 | 4.25675693052308 | CAV1//SCG2//FGF2//KDR//TEK//VIP//FGFR1//TGFBR2//FOXP2//CNTFR//CST3//EDNRB//FGF7//IGF1//IL6ST//KIT//LIFR//NTRK2//PBX1//PTHLH//PTN//SFRP1//GLP2R//TSPYL5//MYOCD//SPDYA//CDH5//DPT//FGF10//FRZB//PMP22//SLIT3//PPAP2A//SLIT2//ADAMTS1//SPRY1//DLC1//TENC1//KANK2//PODN//ANGPT1//MEF2C//NFATC2//VCAM1//GPAM//PRNP//AQP1//CALCRL//APOD//OGN//OSR1//TGFBR3//WFDC1//SRPX//SERPINF1//CDON//NFIB//ANXA1 |
| GO:0050789 | regulation of biological process | Biological process | 278 | 8229 | 428 | 14747 | 1.16401250194491 | 5.79257380496753e-05 | 0.0028479159253495 | 4.23712842402294 | WEE1//CCNC//DIRAS3//CAV1//CD36//EDNRB//FGFR1//FOSB//MEF2C//NFIB//NFIX//ZEB1//TCF21//NR2F1//KANK2//TAF9B//MYOCD//OSR1//MAPK4//SCG2//DOK5//FGF2//LPAR1//FGF10//GHR//KIT//PSTK//IGFBP4//FBLN5//SPRY1//C3//FCER1A//CLU//A2M//BVES//BDKRB1//PRNP//PPAP2B//SLIT2//MUSK//TEK//CDON//KDR//VIP//PIK3R1//CCL21//SFRP1//PLN//ANK2//CASQ2//DMD//EPAS1//TGFBR2//FOXP2//NLGN1//ANGPT1//COLEC12//ANO6//ANXA1//IL6ST//SELP//CORIN//C7//CFD//CFH//CD44//FRZB//FZD4//ZRANB1//APCDD1//BMX//CD34//CNTFR//RCAN1//ERG//FGF7//GEM//GNAL//GNG11//IGF1//MFAP4//PPP1R12A//NR4A2//OPHN1//PDE1A//PRKAR2B//RLN1//CXCL12//SHB//TNXB//SPARCL1//RGS5//PTPLA//CAP2//IQGAP2//PDE7B//KCNIP1//SPA17//SLC44A2//SMOC2//CNTNAP3//PPP1R1C//NTRK2//ROR1//ANGPTL1//PLCB4//DCLK1//TENC1//PREX2//ASB5//DCDC1//ERRFI1//EYA4//EFEMP1//FOXC1//KCNH1//AFF3//NFATC2//RORB//ZNF43//PDE8B//PDLIM1//HIPK3//SSBP2//CCDC59//BNC2//ZNF331//TSPYL2//ZNF518B//ZFP3//ZNF300P1//ZNF280B//ZNF738//GTF2H5//ZNF876P//ZNF826P//LARP6//FABP4//PLAGL1//DLC1//CALCRL//MYL9//CNN1//CAB39L//JAM2//GFRA1//KLRB1//LIFR//KLRG1//CD160//GNG7//PIK3C2A//SMAD9//TGFBR3//FMOD//PTN//PTPRD//BAI3//CHRM2//PTGER3//PTGFR//AKAP12//GPR64//LPHN3//CCRL1//ELTD1//GLP2R//PTHLH//ARHGAP6//PPAP2A//CACNB4//CPE//CNTN1//BOC//ITGA9//ITGA8//ADAMTS1//RCAN2//ITSN1//RAB9B//RHOJ//RERGL//ARHGAP28//RABL3//KSR1//CTNNAL1//DES//AQP1//CRYAB//NME5//FAIM2//KCTD12//CST3//PBX1//TSPYL5//SPDYA//CDH5//DPT//PMP22//SLIT3//PODN//PDK4//SORBS1//MYH10//WASF3//FERMT2//PALMD//PARVA//TEX15//APOD//PLA2G2A//ECM2//VIT//ABI3BP//CCDC80//SERPINA6//SERPINF1//RECK//DPYSL3//LIPG//EBF1//EBF3//ARID5B//VCAM1//KCNMB4//SH3D19//LAMA2//WFDC1//NEXN//PTP4A1//MGP//CHRDL1//ABCD2//NNAT//OXCT1//PTGIS//VDAC2//ALDH1A1//KCNMA1//MAP1B//RNF180//CETN2//DARC//CNN3//TACC1//EDA2R//CTSG//LDB2//ARHGEF26//TEAD1//TRIM9//RGS22//GPAM//SRPX//TPM2//CILP//PTGDS//PTX3//APOLD1//ADAMTS9//TMOD2//CMA1//TTN//MT1A//FHL5//BEX1//ZNF462//SH2D1A//TIPARP//OGN//ASPA//PDGFD//CD200//TIMP3//ANKRD42//TRPC1//CAPN6//PARM1 |
| GO:0050678 | regulation of epithelial cell proliferation | Biological process | 17 | 196 | 428 | 14747 | 2.98849656685104 | 5.86952144355255e-05 | 0.00285630079227573 | 4.2313973064009 | CAV1//SCG2//FGF2//KDR//TEK//VIP//FGF10//FGF7//IGF1//SFRP1//OSR1//TGFBR3//WFDC1//FOXP2//SERPINF1//NFIB//EDNRB |
| GO:0050730 | regulation of peptidyl-tyrosine phosphorylation | Biological process | 15 | 160 | 428 | 14747 | 3.23021320093458 | 6.57454109295044e-05 | 0.00316706934063441 | 4.18213455580472 | ERRFI1//IL6ST//KIT//GHR//IGF1//CAV1//ANGPT1//CD36//CD44//FCER1A//FGF7//FGF10//PPAP2B//SFRP1//PDGFD |
| GO:0046058 | cAMP metabolic process | Biological process | 12 | 108 | 428 | 14747 | 3.82840083073728 | 6.88442683652947e-05 | 0.00327215209371231 | 4.16213221112154 | CALCRL//PDE8B//PDE7B//GNAL//NTRK2//CAP2//EDNRB//VIP//PTHLH//AKAP12//GNG7//CACNB4 |
| GO:0045785 | positive regulation of cell adhesion | Biological process | 13 | 125 | 428 | 14747 | 3.58338317757009 | 6.92990902631461e-05 | 0.00327215209371231 | 4.15927246663334 | CD36//CCL21//SFRP1//ECM2//VIT//ABI3BP//SMOC2//CCDC80//CD44//PPAP2B//KDR//TEK//ANGPT1 |
| GO:0001501 | skeletal system development | Biological process | 26 | 389 | 428 | 14747 | 2.30294548687024 | 7.11931863297443e-05 | 0.00332863044712304 | 4.14756156929845 | MGP//MEF2C//PTHLH//FGFR1//NFIB//OSR1//EFEMP1//IGF1//TGFBR2//RDH10//TIPARP//ARID5B//PBX1//CD44//SMAD9//CHRDL2//SFRP1//PAPSS2//GHR//FGF2//FRZB//FREM1//COL19A1//FOXC1//IGFBP4//TLL1 |
| GO:0045137 | development of primary sexual characteristics | Biological process | 18 | 219 | 428 | 14747 | 2.83196773780566 | 7.23995936831952e-05 | 0.00335217147839959 | 4.14026387111834 | FOXC1//KDR//KIT//ADAMTS1//FRZB//SFRP1//OSR1//RDH10//GFRA1//ARID5B//TIPARP//TEX15//FZD4//FGF10//TCF21//CST3//SLIT3//SLIT2 |
| GO:0010463 | mesenchymal cell proliferation | Biological process | 8 | 49 | 428 | 14747 | 5.62540530230784 | 7.48366600495744e-05 | 0.00343169261323481 | 4.12588560324589 | FGFR1//KDR//TGFBR2//FOXP2//GPC3//OSR1//NFIB//FGF7 |
| GO:1901135 | carbohydrate derivative metabolic process | Biological process | 63 | 1339 | 428 | 14747 | 1.62113761839286 | 8.18726487021504e-05 | 0.00368593122011653 | 4.08686115930971 | GPC5//GPC3//GPC6//CALCRL//AQP1//NME5//GNAL//GNG11//RHOJ//PDE8B//PDE7B//ACTC1//TIPARP//B3GALT2//ST6GALNAC3//DPM1//GCNT4//ADAMTS9//MAT2A//UGCG//KIT//NTRK2//CAP2//EDNRB//CACNB4//PTHLH//GLT25D2//CCL21//VIP//FMOD//OMD//OGN//MAN1A1//UBE2J1//MAMDC2//IGF1//CD44//FOXC1//UST//LYVE1//ANGPT1//FGF2//AKAP12//AMPD1//BVES//ALDH1A1//DLC1//ERRFI1//CNN3//PLN//FGF10//SFRP1//PREX2//SPRY1//PLA2G2A//ARHGAP6//TPM2//RGS5//RGS22//GNG7//CMAHP//PAPSS2//CST3 |
| GO:0035295 | tube development | Biological process | 28 | 438 | 428 | 14747 | 2.20264157384885 | 8.19267580902396e-05 | 0.00368593122011653 | 4.08657423019341 | CXCL12//TGFBR2//FGFR1//FOXC1//SMAD9//SFRP1//TCF21//SLIT2//SPRY1//OSR1//CD44//FGF2//PBX1//DLC1//MEF2C//EPAS1//HEG1//IGF1//KDR//PTHLH//ERRFI1//MYOCD//FOXP2//FGF10//RDH10//NFIB//FGF7//GPC3 |
| GO:0040017 | positive regulation of locomotion | Biological process | 19 | 242 | 428 | 14747 | 2.70519232254576 | 8.50578420633721e-05 | 0.00379103597009553 | 4.07028563963114 | BDKRB1//SELP//ANGPT1//KDR//TEK//FGF10//TGFBR2//IGF1//PIK3R1//PTP4A1//FGF2//PPAP2B//CCL21//SLIT2//CXCL12//SCG2//FGF7//LPAR1//BVES |
| GO:1900542 | regulation of purine nucleotide metabolic process | Biological process | 24 | 351 | 428 | 14747 | 2.3559389727614 | 9.48837619408014e-05 | 0.00418982093236742 | 4.02280810477932 | GNAL//NTRK2//CAP2//EDNRB//VIP//PTHLH//AKAP12//CALCRL//BVES//ALDH1A1//DLC1//ERRFI1//CNN3//PLN//FGF10//SFRP1//PREX2//CCL21//SPRY1//ARHGAP6//TPM2//RGS5//RGS22//GNG7 |
| GO:0030036 | actin cytoskeleton organization | Biological process | 27 | 422 | 428 | 14747 | 2.20450569163308 | 0.000108081649068809 | 0.00472882004045092 | 3.96624803763499 | DPYSL3//CXCL12//ARHGAP6//WASF3//TMOD1//ACTC1//TTN//MYH11//SLIT2//CCL21//EPB41L2//AQP1//CNN1//CNN3//FGF7//FGF10//KIT//DLC1//SORBS1//SFRP1//MYH10//TEK//OPHN1//TNXB//FERMT2//DAAM2//RHOJ |
| GO:0006468 | protein phosphorylation | Biological process | 55 | 1133 | 428 | 14747 | 1.67260230469104 | 0.000109142901183694 | 0.00473184087040942 | 3.96200450619404 | CCNC//DIRAS3//FGF2//LPAR1//FGF10//GHR//KIT//CAV1//BDKRB1//PRNP//PPAP2B//SLIT2//C3//EDNRB//MUSK//TEK//CDON//ERRFI1//FABP4//TSPYL2//ANGPT1//TGFBR2//FCER1A//HIPK3//EFEMP1//FGFR1//KDR//WEE1//DMD//CD44//PRKAR2B//IL6ST//IGF1//SPRY1//TNXB//FZD4//SFRP1//MYOCD//SPDYA//TTN//CCL21//BMX//NTRK2//PDGFD//CD36//FGF7//TGFBR3//ERG//SMAD9//PDK4//PIK3R1//MAPK4//DYRK3//KSR1//DCLK1 |
| GO:0006140 | regulation of nucleotide metabolic process | Biological process | 24 | 355 | 428 | 14747 | 2.32939318151902 | 0.000112967985556126 | 0.004853552460515 | 3.94704461550987 | GNAL//NTRK2//CAP2//EDNRB//VIP//PTHLH//AKAP12//CALCRL//BVES//ALDH1A1//DLC1//ERRFI1//CNN3//PLN//FGF10//SFRP1//PREX2//CCL21//SPRY1//ARHGAP6//TPM2//RGS5//RGS22//GNG7 |
| GO:0006026 | aminoglycan catabolic process | Biological process | 9 | 66 | 428 | 14747 | 4.69849192863211 | 0.000114756114781087 | 0.00488635635170539 | 3.94022416378695 | GPC5//GPC3//GPC6//CD44//FGF2//LYVE1//FMOD//OMD//OGN |
| GO:0070887 | cellular response to chemical stimulus | Biological process | 76 | 1723 | 428 | 14747 | 1.51980624969489 | 0.000116443618587277 | 0.0049143328941834 | 3.93388430694271 | CAV1//CCL21//KIT//SLIT2//ALDH1A1//FMO1//GSTA2//GSTM5//MAT2A//PTGIS//PAPSS2//TSPYL2//CCNC//SMAD9//TGFBR2//TGFBR3//FMOD//FGF2//FGF7//FGF10//FGFR1//PDK4//PIK3C2A//PIK3R1//SORBS1//CAB39L//PDE1A//PRKAR2B//GHR//COLEC12//CD44//IL6ST//LIFR//VCAM1//SOD3//PDGFD//ITGA8//MYOCD//ITGA9//FZD4//SLIT3//EDA2R//MEF2C//KDR//SPRY1//GPX3//EPAS1//SFRP1//SCG2//EDNRB//CD36//DARC//CXCL12//CCRL1//CNTFR//ANXA1//AQP1//CST3//PLSCR4//OXCT1//MT1A//FOSB//FABP4//OSR1//TIMP3//CASQ2//CALCRL//CMA1//DPYSL3//GNG7//GNG11//LPAR1//PARVA//DMD//CD34//FBLN5 |
| GO:0030155 | regulation of cell adhesion | Biological process | 20 | 269 | 428 | 14747 | 2.56175520272383 | 0.000118064973634303 | 0.00492109049895847 | 3.92787892553262 | PIK3R1//CD36//CCL21//SFRP1//ANGPT1//JAM2//ECM2//VIT//ABI3BP//SMOC2//CCDC80//FZD4//CD44//PPAP2B//KDR//TEK//APOD//ARHGAP6//LAMA2//PPP1R12A |
| GO:0010035 | response to inorganic substance | Biological process | 22 | 312 | 428 | 14747 | 2.4295620656602 | 0.000118667520943641 | 0.00492109049895847 | 3.92566813028802 | KCNMB4//KCNIP1//CYBRD1//SOD3//PRNP//GATM//CRYAB//GPX3//CAV1//KCNMA1//TRPC1//TTN//ANXA1//AQP1//CST3//MT1A//FOSB//MEF2C//FABP4//CD36//FBLN5//NR4A2 |
| GO:0030203 | glycosaminoglycan metabolic process | Biological process | 14 | 150 | 428 | 14747 | 3.21585669781931 | 0.000120043106994338 | 0.00493522049358619 | 3.92066277255213 | GPC5//GPC3//GPC6//FMOD//OMD//OGN//MAMDC2//UST//ANGPT1//CD44//LYVE1//FGF2//IGF1//FOXC1 |
| GO:0008406 | gonad development | Biological process | 16 | 189 | 428 | 14747 | 2.91687682341888 | 0.000128099421362707 | 0.0051771706820233 | 3.89245283200044 | FOXC1//KDR//KIT//ADAMTS1//GFRA1//SFRP1//ARID5B//TIPARP//FZD4//TCF21//CST3//SLIT3//SLIT2//FRZB//OSR1//RDH10 |
| GO:0060538 | skeletal muscle organ development | Biological process | 16 | 189 | 428 | 14747 | 2.91687682341888 | 0.000128099421362707 | 0.0051771706820233 | 3.89245283200044 | CAV1//COL19A1//MEF2C//MEOX2//FOXP2//KCNH1//CDON//CACNB4//MUSK//MYOCD//TCF21//DMD//IGF1//BOC//RCAN1//CNTFR |
| GO:0003006 | developmental process involved in reproduction | Biological process | 27 | 427 | 428 | 14747 | 2.17869180765611 | 0.000131286299983832 | 0.00526138121531844 | 3.88178059113712 | FOXC1//KDR//KIT//ADAMTS1//SMAD9//CXCL12//NME5//TCF21//CNTFR//PBX1//FRZB//SFRP1//OSR1//RDH10//GFRA1//ARID5B//TIPARP//FGF10//TEX15//FZD4//CST3//MYOCD//CD44//IGF1//SERPINF1//SLIT3//SLIT2 |
| GO:0019722 | calcium-mediated signaling | Biological process | 11 | 99 | 428 | 14747 | 3.82840083073728 | 0.000137417062241386 | 0.00546118308190975 | 3.86195934022236 | ANK2//CASQ2//DMD//PLN//KDR//NTRK2//FCER1A//PRNP//IGF1//RCAN1//RCAN2 |
| GO:0055002 | striated muscle cell development | Biological process | 13 | 134 | 428 | 14747 | 3.3427081880318 | 0.000141394371425867 | 0.00557280791181785 | 3.84956787842696 | CACNB4//MUSK//MYOCD//TMOD1//ACTC1//TTN//MYH11//MEF2C//BOC//RCAN1//DMD//FLNC//MYH10 |
| GO:0042692 | muscle cell differentiation | Biological process | 21 | 295 | 428 | 14747 | 2.4527720576588 | 0.000147891865062982 | 0.00578111724988001 | 3.83005571413984 | KCNH1//CDON//MEF2C//CACNB4//MUSK//MYOCD//DMD//IGF1//TMOD1//ACTC1//TTN//MYH11//ANK2//EDNRB//FGF10//BOC//EPAS1//RCAN1//FLNC//MYH10//ITGA8 |
| GO:0008285 | negative regulation of cell proliferation | Biological process | 31 | 526 | 428 | 14747 | 2.03065367257738 | 0.000152844267455389 | 0.00592613261377846 | 3.81575084518898 | CAV1//SCG2//SFRP1//PRNP//APOD//OGN//VIP//TGFBR3//WFDC1//TGFBR2//SRPX//SERPINF1//NFIB//CDH5//DPT//FGF2//FGF10//FRZB//IGF1//PMP22//PTHLH//SLIT3//PPAP2A//SLIT2//ADAMTS1//SPRY1//DLC1//TENC1//KANK2//MYOCD//PODN |
| GO:0050918 | positive chemotaxis | Biological process | 7 | 41 | 428 | 14747 | 5.88266469113289 | 0.000158051273648197 | 0.00607860100022783 | 3.80120200007018 | KDR//FGF10//CXCL12//SCG2//ANGPT1//FGF2//FGF7 |
| GO:0010033 | response to organic substance | Biological process | 81 | 1885 | 428 | 14747 | 1.48058578546816 | 0.000159405331401769 | 0.00608163220364029 | 3.7974971574716 | CAV1//GNAL//NR4A2//GPX3//TSPYL2//CCNC//SMAD9//TGFBR2//TGFBR3//FMOD//FGF2//FGF7//FGF10//FGFR1//PDK4//PIK3C2A//PIK3R1//SORBS1//CAB39L//PDE1A//PRKAR2B//OXCT1//SNRPN//CST3//NNAT//GPAM//GHR//COLEC12//EDNRB//EPHX1//SFRP1//TAF9B//CD44//IL6ST//KIT//LIFR//VCAM1//PDGFD//ITGA8//MYOCD//FZD4//ANXA1//CRYAB//WFDC1//BDKRB1//CTSG//FMO1//PTGFR//SELP//SLIT3//SLIT2//EDA2R//KCNMA1//AFF3//CCL21//CD36//KDR//MEF2C//SPRY1//GATM//RCAN1//ACTC1//PTGDS//CLU//DARC//CXCL12//CCRL1//CNTFR//PLSCR4//AQP1//OSR1//TIMP3//CASQ2//CALCRL//CMA1//DPYSL3//PTGIS//GNG7//GNG11//DMD//AMPD1 |
| GO:0051128 | regulation of cellular component organization | Biological process | 59 | 1260 | 428 | 14747 | 1.61339749295357 | 0.000160840828518936 | 0.00608769770799052 | 3.79360369843735 | IGFBP4//FBLN5//MYOCD//ANGPT1//CXCL12//KDR//KIT//MYH10//DLC1//WASF3//FERMT2//BVES//PALMD//PARVA//RHOJ//MUSK//SLIT2//CST3//SFRP1//TGFBR3//CNTN1//DMD//DPYSL3//FGFR1//NTRK2//LPAR1//PMP22//ZRANB1//SH3D19//OPHN1//TAF9B//PPP1R1C//BDKRB1//CRYAB//FGF2//FRZB//SLIT3//WFDC1//TSPYL2//CCL21//ANXA1//VDAC2//CLU//SELP//TRIM9//MAP1B//IGF1//C3//PTX3//PTPRD//NLGN1//CAPN6//NEXN//ARHGAP6//TEK//APOD//CD36//PIK3R1//SORBS1 |
| GO:0070252 | actin-mediated cell contraction | Biological process | 9 | 69 | 428 | 14747 | 4.4942096708655 | 0.000162734537083828 | 0.0061108740736439 | 3.78852026714199 | ACTC1//DES//DMD//TMOD1//TPM2//TTN//ANK2//PLN//PARVA |
| GO:0007169 | transmembrane receptor protein tyrosine kinase signaling pathway | Biological process | 35 | 627 | 428 | 14747 | 1.92335926903069 | 0.000167092023921104 | 0.00622548329749801 | 3.77704428050593 | ERRFI1//ANGPT1//EFEMP1//PDE1A//PIK3C2A//PIK3R1//PRKAR2B//SPRY1//FGF2//FGF7//FGF10//FGFR1//PDK4//SORBS1//CAB39L//APOD//FZD4//NTRK2//GFRA1//KDR//KIT//IGF1//IGFBP4//CILP//TIPARP//ARID5B//GHR//FOXC1//MEF2C//ITSN1//TEK//MUSK//ROR1//ANGPTL1//DOK5 |
| GO:0030048 | actin filament-based movement | Biological process | 10 | 85 | 428 | 14747 | 4.05360087960418 | 0.0001710186055702 | 0.0063223855035991 | 3.76695663898261 | ACTC1//DES//DMD//TMOD1//TPM2//TTN//PARVA//ANK2//PLN//MYH10 |
| GO:0014066 | regulation of phosphatidylinositol 3-kinase cascade | Biological process | 8 | 55 | 428 | 14747 | 5.01172472387426 | 0.000173569058304118 | 0.00636731414655645 | 3.76052769278552 | ANGPT1//FGFR1//IGF1//KDR//KIT//NTRK2//SELP//TEK |
| GO:0055017 | cardiac muscle tissue growth | Biological process | 7 | 42 | 428 | 14747 | 5.74260124610592 | 0.000184982823305483 | 0.00666463941756004 | 3.73286859640766 | FOXC1//TGFBR3//TGFBR2//FGF2//FGFR1//MEF2C//MYOCD |
| GO:0048583 | regulation of response to stimulus | Biological process | 97 | 2368 | 428 | 14747 | 1.41139946166961 | 0.000187114808994594 | 0.00666463941756004 | 3.72789183938335 | FGF2//LPAR1//FGF10//GHR//KIT//CAV1//C3//FCER1A//A2M//COLEC12//MEF2C//ANO6//ANXA1//IL6ST//SLIT2//C7//CLU//CFD//CFH//ERRFI1//GNG7//RGS5//KCTD12//VIP//TSPYL2//TEX15//SELP//APOD//AKAP12//TEK//NLGN1//ANGPT1//FGFR1//IGF1//KDR//NTRK2//CACNB4//PIK3R1//PPAP2B//SFRP1//ZRANB1//FRZB//APCDD1//CD36//CD34//TGFBR3//ITGA8//MYOCD//TGFBR2//FZD4//PTGER3//BVES//ALDH1A1//DLC1//PREX2//CCL21//KANK2//SPRY1//ITSN1//ARHGEF26//PTGIS//RGS22//SLC44A2//IGFBP4//CDON//TNXB//HIPK3//CD44//CILP//PTGDS//TMOD2//SH2D1A//EDA2R//CMA1//CALCRL//FABP4//PLA2G2A//CD200//VCAM1//KLRG1//CD160//CTSG//NFATC2//PRNP//CXCL12//SCG2//ARHGAP6//OPHN1//RHOJ//ARHGAP28//TSPYL5//TCF21//SLIT3//DMD//ANKRD42//FBLN5//VDAC2 |
| GO:0034332 | adherens junction organization | Biological process | 10 | 86 | 428 | 14747 | 4.00646598565529 | 0.000188603964022468 | 0.00666463941756004 | 3.72444918362948 | ARHGAP6//DLC1//SORBS1//KDR//SFRP1//TEK//APOD//CDH5//CADM3//CADM2 |
| GO:0040007 | growth | Biological process | 41 | 784 | 428 | 14747 | 1.80188763589548 | 0.000189740091913951 | 0.00666463941756004 | 3.72184089337423 | EMP1//TGFBR3//IGFBP4//FBLN5//MYOCD//MUSK//IGF1//SFRP1//TAF9B//PPP1R1C//BDKRB1//CRYAB//FGF2//FRZB//SLIT3//SLIT2//WFDC1//TSPYL2//CD36//CTSG//TENC1//HEG1//ARID5B//TSPYL5//GHR//GPAM//APOD//FGF10//GATM//TGFBR2//MAP1B//MT1A//FOXC1//DCLK1//FGFR1//MEF2C//RDH10//PTHLH//FGF7//SEPP1//FOXP2 |
| GO:0007010 | cytoskeleton organization | Biological process | 42 | 810 | 428 | 14747 | 1.78658705434406 | 0.000189979579136308 | 0.00666463941756004 | 3.72129307875794 | TACC1//WEE1//CAPN6//MAP1B//DPYSL3//OPHN1//TNXB//DLC1//FERMT2//DAAM2//RHOJ//CETN2//CXCL12//ARHGAP6//WASF3//TMOD1//ACTC1//TTN//MYH11//SLIT2//CCL21//EPB41L2//AQP1//CNN1//CNN3//CRYAB//FGF7//FGF10//KIT//SORBS1//SYNM//NLGN1//PPP1R12A//NEXN//SFRP1//MYH10//TEK//DES//SGCD//CAP2//ZRANB1//SH3D19 |
| GO:0009187 | cyclic nucleotide metabolic process | Biological process | 13 | 138 | 428 | 14747 | 3.24581809562508 | 0.000190058913983679 | 0.00666463941756004 | 3.72111175666789 | CALCRL//AQP1//PDE8B//PDE7B//GNAL//NTRK2//CAP2//EDNRB//CACNB4//PTHLH//VIP//AKAP12//GNG7 |
| GO:0046541 | saliva secretion | Biological process | 4 | 11 | 428 | 14747 | 12.5293118096856 | 0.000196329313760172 | 0.00683426640381212 | 3.70701485142482 | AQP1//FGF10//KCNMA1//TRPC1 |
| GO:1901564 | organonitrogen compound metabolic process | Biological process | 74 | 1696 | 428 | 14747 | 1.50336966584377 | 0.000203747082386308 | 0.0070410857673935 | 3.69090860154553 | MAT2A//ADI1//GHR//GPC5//GPC3//GPC6//GSTA2//GSTM5//AMPD1//CALCRL//AQP1//NME5//GNAL//GNG11//RHOJ//PDE8B//PDE7B//ACTC1//DPYSL3//CLN5//CMA1//ASPA//CD34//GATM//PEMT//SLC44A2//UGCG//PPAP2A//PPAP2B//ST6GALNAC3//KIT//NTRK2//CAP2//EDNRB//CACNB4//PTHLH//AOC3//VIP//FMOD//OMD//OGN//BCHE//MAMDC2//CD44//FOXC1//UST//LYVE1//ANGPT1//FGF2//AKAP12//BVES//ALDH1A1//DLC1//ERRFI1//CNN3//PLN//FGF10//SFRP1//PREX2//CCL21//SPRY1//PLA2G2A//NR4A2//GCNT4//EPAS1//DMD//ARHGAP6//TPM2//RGS5//RGS22//GNG7//PAPSS2//IGF1//FMO1 |
| GO:1901700 | response to oxygen-containing compound | Biological process | 40 | 762 | 428 | 14747 | 1.80869330586013 | 0.000210554324557473 | 0.0072239825454287 | 3.67663583421446 | APOD//GPX3//CST3//NNAT//TGFBR2//GPAM//COLEC12//SOD3//PDGFD//ANXA1//CRYAB//FGF10//GHR//WFDC1//BDKRB1//CTSG//FMO1//PTGFR//SELP//CAV1//KCNMA1//CCL21//TGFBR3//ACTC1//OXCT1//SLIT3//SLIT2//AQP1//CD36//EDNRB//MEF2C//PLSCR4//FZD4//OSR1//SFRP1//CALCRL//CMA1//VCAM1//PDK4//FBLN5 |
| GO:0048041 | focal adhesion assembly | Biological process | 7 | 43 | 428 | 14747 | 5.60905237991741 | 0.000215503414795142 | 0.00734096989398594 | 3.6665458437634 | KDR//SFRP1//TEK//APOD//ARHGAP6//DLC1//SORBS1 |
| GO:0006022 | aminoglycan metabolic process | Biological process | 14 | 159 | 428 | 14747 | 3.03382707341445 | 0.000222282818586788 | 0.00746525888619994 | 3.65309410491829 | GPC5//GPC3//GPC6//FMOD//OMD//OGN//MAMDC2//CD44//FOXC1//UST//LYVE1//ANGPT1//FGF2//IGF1 |
| GO:0055001 | muscle cell development | Biological process | 14 | 159 | 428 | 14747 | 3.03382707341445 | 0.000222282818586788 | 0.00746525888619994 | 3.65309410491829 | CACNB4//MUSK//MYOCD//TMOD1//ACTC1//TTN//MYH11//ANK2//MEF2C//BOC//RCAN1//DMD//FLNC//MYH10 |
| GO:0070482 | response to oxygen levels | Biological process | 18 | 240 | 428 | 14747 | 2.58417056074766 | 0.000230047514527646 | 0.0076720041733031 | 3.63818245456506 | CAV1//CRYAB//CST3//EPAS1//KCNMA1//SMAD9//NR4A2//SOD3//TGFBR3//VCAM1//PDLIM1//APOLD1//MYOCD//LPAR1//AQP1//PTGIS//SFRP1//CD34 |
| GO:0008585 | female gonad development | Biological process | 10 | 89 | 428 | 14747 | 3.87141657040848 | 0.000250734731866117 | 0.00830384677964939 | 3.60078550324545 | FOXC1//KDR//KIT//ADAMTS1//FZD4//SLIT3//SLIT2//SFRP1//TIPARP//ARID5B |
| GO:0046545 | development of primary female sexual characteristics | Biological process | 11 | 106 | 428 | 14747 | 3.57558190795274 | 0.000252819343600879 | 0.00831514103194891 | 3.59718970057267 | FOXC1//KDR//KIT//ADAMTS1//SFRP1//TIPARP//ARID5B//FZD4//FGF10//SLIT3//SLIT2 |
| GO:0030334 | regulation of cell migration | Biological process | 25 | 398 | 428 | 14747 | 2.16429695204997 | 0.000256328923852898 | 0.00837282628667446 | 3.59120238577451 | BDKRB1//SELP//SLIT2//ANGPT1//KDR//TEK//TGFBR3//FGF10//TGFBR2//FGF2//IGF1//PIK3R1//PTP4A1//DPYSL3//SFRP1//DLC1//PODN//PPAP2B//FGF7//LPAR1//CCL21//CXCL12//APOD//LAMA2//NEXN |
| GO:0048584 | positive regulation of response to stimulus | Biological process | 55 | 1173 | 428 | 14747 | 1.61556556795819 | 0.000264704022047078 | 0.00858757470164976 | 3.57723945975803 | LPAR1//FGF2//FGF10//GHR//KIT//C3//FCER1A//A2M//COLEC12//MEF2C//IL6ST//C7//CLU//CFD//CFH//AKAP12//TEK//NLGN1//ANGPT1//FGFR1//IGF1//KDR//NTRK2//SELP//CACNB4//PIK3R1//SFRP1//ZRANB1//TGFBR3//ITGA8//MYOCD//PTGER3//PTGIS//CD36//CCL21//SLC44A2//IGFBP4//CDON//FZD4//PPAP2B//TMOD2//SH2D1A//EDA2R//FABP4//PLA2G2A//CTSG//NFATC2//PRNP//SLIT2//CXCL12//SCG2//ITSN1//TSPYL5//CD44//CAV1 |
| GO:0032989 | cellular component morphogenesis | Biological process | 49 | 1010 | 428 | 14747 | 1.67160867955954 | 0.000269569813453059 | 0.00868432676817223 | 3.56932874190215 | TGFBR3//HEG1//ANK2//CACNB4//CNTN1//DPYSL3//FGFR1//GFRA1//ITGA9//LAMA2//MYH10//MYH11//OPHN1//PRNP//SLIT3//TRPC1//SLIT2//MYL9//CAP2//BOC//CXCL12//KDR//KIT//DLC1//WASF3//FERMT2//BVES//PALMD//PARVA//RHOJ//SFRP1//APOD//FOXC1//NR4A2//DCLK1//NFIB//ZRANB1//SH3D19//MEF2C//TMOD1//ACTC1//TTN//PMP22//TEK//FZD4//MAP1B//WEE1//NTRK2//PTPRD |
| GO:0007610 | behavior | Biological process | 30 | 519 | 428 | 14747 | 1.99165361136621 | 0.000271874616437162 | 0.00868432676817223 | 3.56563133840691 | GNG7//CNTFR//GCNT4//SLIT2//MEF2C//PRNP//VIP//TMOD2//NTRK2//PRKAR2B//PTN//MUSK//ITGA8//SEPP1//SOBP//CACNB4//KCNMA1//NR4A2//TMOD1//FZD4//FOXP2//CST3//PTGDS//CCL21//KDR//FGF10//CXCL12//SCG2//LPAR1//FOSB |
| GO:0033002 | muscle cell proliferation | Biological process | 11 | 107 | 428 | 14747 | 3.54216525460739 | 0.000274591013876351 | 0.00868432676817223 | 3.56131367936354 | IGF1//TGFBR2//CALCRL//APOD//OGN//VIP//FOXC1//TGFBR3//FGF2//FGFR1//MEF2C |
| GO:0060428 | lung epithelium development | Biological process | 6 | 32 | 428 | 14747 | 6.46042640186916 | 0.000275430544825742 | 0.00868432676817223 | 3.55998789881517 | NFIB//FGF7//FOXP2//IGF1//FGF10//ERRFI1 |
| GO:0007519 | skeletal muscle tissue development | Biological process | 15 | 182 | 428 | 14747 | 2.83974786895348 | 0.000276791291415848 | 0.00868432676817223 | 3.55784757805032 | KCNH1//CDON//CACNB4//MUSK//MYOCD//TCF21//MEF2C//DMD//IGF1//BOC//RCAN1//CAV1//COL19A1//MEOX2//FOXP2 |
| GO:0001952 | regulation of cell-matrix adhesion | Biological process | 8 | 59 | 428 | 14747 | 4.67194677649295 | 0.000285711334934823 | 0.00884777504093618 | 3.54407252964877 | PIK3R1//CD36//CCL21//SFRP1//KDR//TEK//APOD//ARHGAP6 |
| GO:0007044 | cell-substrate junction assembly | Biological process | 8 | 59 | 428 | 14747 | 4.67194677649295 | 0.000285711334934823 | 0.00884777504093618 | 3.54407252964877 | ARHGAP6//DLC1//SORBS1//KDR//SFRP1//TEK//APOD//TNS1 |
| GO:0014065 | phosphatidylinositol 3-kinase cascade | Biological process | 9 | 75 | 428 | 14747 | 4.13467289719626 | 0.000309439817366571 | 0.00952076444529792 | 3.5094238039205 | ANGPT1//FGFR1//IGF1//KDR//KIT//NTRK2//SELP//TEK//PIK3R1 |
| GO:0001666 | response to hypoxia | Biological process | 17 | 225 | 428 | 14747 | 2.60331256490135 | 0.000312778569781421 | 0.00956180127748459 | 3.50476301054987 | EPAS1//AQP1//PTGIS//SFRP1//MYOCD//CD34//CAV1//CRYAB//CST3//KCNMA1//SMAD9//NR4A2//SOD3//TGFBR3//VCAM1//PDLIM1//APOLD1 |
| GO:2000026 | regulation of multicellular organismal development | Biological process | 53 | 1127 | 428 | 14747 | 1.62036130990389 | 0.000321282960595972 | 0.00975922572663816 | 3.49311230686484 | FGF2//FGF10//FGFR1//SPRY1//TGFBR2//MUSK//CST3//SFRP1//TGFBR3//MYOCD//CNTN1//DMD//DPYSL3//NTRK2//LPAR1//PMP22//EDNRB//BAI3//SERPINF1//TEK//IGF1//MGP//MEF2C//OSR1//CAV1//WASF3//EFEMP1//PTHLH//CD44//CD34//APOLD1//ERRFI1//BOC//NLGN1//PBX1//ZEB1//CDON//PIK3R1//AQP1//C3//CMA1//KDR//PTGIS//MAP1B//LAMA2//IL6ST//ASPA//SLIT2//PTPRD//FGF7//FOXP2//FRZB//NFIB |
| GO:0044057 | regulation of system process | Biological process | 27 | 452 | 428 | 14747 | 2.0581889421884 | 0.000327912432145742 | 0.00989755942343699 | 3.48424211760955 | PLN//ANK2//CASQ2//DMD//EPAS1//NLGN1//CAV1//CORIN//EDNRB//CALCRL//MYL9//CNN1//CHRM2//DES//VIP//MYOCD//AQP1//IGF1//IL6ST//KCNMB4//WASF3//LAMA2//FGF10//NTRK2//OPHN1//MEF2C//KCNMA1 |
| GO:0036293 | response to decreased oxygen levels | Biological process | 17 | 227 | 428 | 14747 | 2.58037589032072 | 0.000346788103705413 | 0.0104014620539064 | 3.45993580907273 | CAV1//CRYAB//CST3//EPAS1//KCNMA1//SMAD9//NR4A2//SOD3//TGFBR3//VCAM1//PDLIM1//APOLD1//MYOCD//AQP1//PTGIS//SFRP1//CD34 |
| GO:0040012 | regulation of locomotion | Biological process | 27 | 455 | 428 | 14747 | 2.0446184656465 | 0.000363770057465359 | 0.0108426212753269 | 3.43917305132185 | BDKRB1//SELP//SLIT2//ANGPT1//KDR//TEK//TGFBR3//FGF10//TGFBR2//FGF2//IGF1//LAMA2//NEXN//PIK3R1//PTP4A1//DPYSL3//SFRP1//DLC1//PODN//BVES//PPAP2B//CCL21//CXCL12//SCG2//FGF7//LPAR1//APOD |
| GO:0090257 | regulation of muscle system process | Biological process | 12 | 129 | 428 | 14747 | 3.20517278852423 | 0.000373834769923651 | 0.0110734038370552 | 3.42732030786965 | CALCRL//MYL9//CAV1//CNN1//MYOCD//IGF1//IL6ST//ANK2//CASQ2//DMD//PLN//KCNMA1 |
| GO:0046660 | female sex differentiation | Biological process | 11 | 111 | 428 | 14747 | 3.41451965984676 | 0.000378178765735777 | 0.0111329292209501 | 3.42230285989784 | FOXC1//KDR//KIT//ADAMTS1//SFRP1//TIPARP//ARID5B//FZD4//FGF10//SLIT3//SLIT2 |
| GO:0016337 | cell-cell adhesion | Biological process | 25 | 409 | 428 | 14747 | 2.10608847656696 | 0.000385903880593415 | 0.0112906478929448 | 3.41352085420437 | MGP//PTPRD//SELP//VCAM1//NLGN1//CADM3//CDH5//PCDH9//CDH19//PCDH18//FAT4//NCAM2//PPAP2B//PARVA//CD44//BVES//KIT//CD34//COL19A1//TEK//TNXB//COL14A1//ITGA8//SLIT2//JAM2 |
| GO:0051216 | cartilage development | Biological process | 14 | 168 | 428 | 14747 | 2.87130062305296 | 0.00039205071116234 | 0.0114005478142268 | 3.40665775401639 | MGP//FGFR1//MEF2C//NFIB//OSR1//EFEMP1//PTHLH//GHR//FGF2//FRZB//CD44//SMAD9//TGFBR2//CHRDL2 |
| GO:0051209 | release of sequestered calcium ion into cytosol | Biological process | 8 | 62 | 428 | 14747 | 4.4458848356949 | 0.000403864249247194 | 0.0115298123094805 | 3.39376458961748 | CASQ2//DMD//ANK2//PLN//BDKRB1//TRPC1//FGF2//CCL21 |
| GO:0051282 | regulation of sequestering of calcium ion | Biological process | 8 | 62 | 428 | 14747 | 4.4458848356949 | 0.000403864249247194 | 0.0115298123094805 | 3.39376458961748 | CASQ2//DMD//ANK2//PLN//FGF2//CCL21//BDKRB1//TRPC1 |
| GO:0051283 | negative regulation of sequestering of calcium ion | Biological process | 8 | 62 | 428 | 14747 | 4.4458848356949 | 0.000403864249247194 | 0.0115298123094805 | 3.39376458961748 | CASQ2//DMD//ANK2//PLN//FGF2//CCL21//BDKRB1//TRPC1 |
| GO:0010881 | regulation of cardiac muscle contraction by regulation of the release of sequestered calcium ion | Biological process | 4 | 13 | 428 | 14747 | 10.6017253774263 | 0.000406166590059285 | 0.0115298123094805 | 3.39129580311152 | ANK2//CASQ2//DMD//PLN |
| GO:0022612 | gland morphogenesis | Biological process | 11 | 112 | 428 | 14747 | 3.38403287716956 | 0.000408666772531766 | 0.0115321410544615 | 3.3886306720333 | CAV1//FRZB//CD44//TGFBR2//FGF7//FGFR1//IGF1//SFRP1//FGF10//PTHLH//SLIT2 |
| GO:0006796 | phosphate-containing compound metabolic process | Biological process | 99 | 2483 | 428 | 14747 | 1.37378378581833 | 0.000422929268535845 | 0.0118644098920438 | 3.37373225875528 | CCNC//DIRAS3//FGF2//LPAR1//FGF10//GHR//KIT//CAV1//BDKRB1//PRNP//PPAP2B//SLIT2//C3//EDNRB//MUSK//TEK//CDON//ERRFI1//CALCRL//AQP1//NME5//GNAL//GNG11//RHOJ//PDE8B//PDE7B//ACTC1//BMX//ERG//FGFR1//SMAD9//PDK4//PIK3R1//MAPK4//TGFBR2//DYRK3//KSR1//DCLK1//HIPK3//FABP4//PPP1R12A//PTPRD//PTPLA//DPM1//PIK3C2A//PLA2G2A//UGCG//PPAP2A//PEMT//SLC44A2//GPAM//TSPYL2//ANGPT1//NTRK2//CAP2//FCER1A//CACNB4//PTHLH//LIPG//VIP//MEF2C//EFEMP1//KDR//WEE1//AKAP12//AMPD1//BVES//ALDH1A1//DLC1//CNN3//PLN//SFRP1//PREX2//CCL21//DMD//CD44//PRKAR2B//SPRY1//PTP4A1//IL6ST//IGF1//ARHGAP6//TPM2//TNXB//FZD4//RGS5//RGS22//RCAN1//MYOCD//SPDYA//GNG7//TTN//CMAHP//PAPSS2//PDGFD//CD36//FGF7//TGFBR3//FMO1 |
| GO:0065009 | regulation of molecular function | Biological process | 77 | 1827 | 428 | 14747 | 1.45215203924517 | 0.000433318369548339 | 0.0120524639016366 | 3.36319289964696 | CCNC//DIRAS3//FGF2//LPAR1//FGF10//GHR//KIT//CAV1//ERRFI1//FABP4//DLC1//TSPYL2//ANGPT1//GNAL//NTRK2//CAP2//EDNRB//ARHGAP6//PDE1A//PRKAR2B//FCER1A//CST3//FGFR1//VIP//MEF2C//SERPINA6//SERPINF1//RECK//PTGIS//TGFBR2//BVES//ALDH1A1//CNN3//PLN//SFRP1//PREX2//CCL21//SPRY1//PPP1R12A//NR4A2//AQP1//CD44//CRYAB//IGF1//MYOCD//NFIB//ZNF462//TGFBR3//PBX1//PRNP//PTHLH//TPM2//CDON//TNXB//FZD4//HIPK3//RGS5//RGS22//PIK3R1//TEK//RCAN1//SPDYA//GNG7//TTN//FOXC1//FOXP2//PPAP2B//BEX1//ARID5B//CLU//EDA2R//ANKRD42//PARM1//DMD//CASQ2//ANK2//NLGN1 |
| GO:0060419 | heart growth | Biological process | 7 | 48 | 428 | 14747 | 5.02477609034268 | 0.000434687312032187 | 0.0120524639016366 | 3.36182303612576 | FOXC1//TGFBR3//TGFBR2//FGF2//FGFR1//MEF2C//MYOCD |
| GO:0001932 | regulation of protein phosphorylation | Biological process | 41 | 819 | 428 | 14747 | 1.72488389077174 | 0.000466276855282077 | 0.0128536088025447 | 3.33135614121019 | CCNC//DIRAS3//FGF2//LPAR1//FGF10//GHR//KIT//CAV1//BDKRB1//PRNP//PPAP2B//SLIT2//C3//EDNRB//MUSK//TEK//CDON//ERRFI1//FABP4//TSPYL2//ANGPT1//FCER1A//TGFBR2//DMD//CD44//PRKAR2B//IL6ST//IGF1//FGFR1//SPRY1//TNXB//FZD4//SFRP1//HIPK3//MYOCD//SPDYA//TTN//CCL21//PDGFD//CD36//FGF7 |
| GO:0050794 | regulation of cellular process | Biological process | 260 | 7791 | 428 | 14747 | 1.14984699575475 | 0.000492052376384862 | 0.0134861941550541 | 3.307988666411 | WEE1//CCNC//DIRAS3//CAV1//CD36//EDNRB//FGFR1//FOSB//MEF2C//NFIB//NFIX//ZEB1//TCF21//NR2F1//KANK2//TAF9B//MYOCD//OSR1//MAPK4//SCG2//DOK5//FGF2//LPAR1//FGF10//GHR//KIT//PSTK//IGFBP4//FBLN5//CLU//BVES//BDKRB1//PRNP//PPAP2B//SLIT2//C3//MUSK//TEK//CDON//KDR//VIP//PIK3R1//CCL21//SFRP1//TGFBR2//FOXP2//ANGPT1//COLEC12//SELP//CD44//FRZB//FZD4//ZRANB1//APCDD1//ANXA1//BMX//CD34//CNTFR//RCAN1//EPAS1//ERG//FGF7//GEM//GNAL//GNG11//IGF1//MFAP4//PPP1R12A//NR4A2//OPHN1//PDE1A//PRKAR2B//RLN1//CXCL12//SHB//TNXB//SPARCL1//RGS5//PTPLA//CAP2//IQGAP2//PDE7B//KCNIP1//SPA17//SLC44A2//SMOC2//CNTNAP3//PPP1R1C//NTRK2//ROR1//ANGPTL1//PLCB4//DCLK1//TENC1//PREX2//ASB5//DCDC1//ERRFI1//DMD//EYA4//EFEMP1//FOXC1//KCNH1//AFF3//NFATC2//RORB//ZNF43//PDE8B//PDLIM1//HIPK3//SSBP2//CCDC59//BNC2//ZNF331//TSPYL2//ZNF518B//ZFP3//ZNF300P1//ZNF280B//ZNF738//GTF2H5//ZNF876P//ZNF826P//LARP6//FABP4//PLAGL1//DLC1//CAB39L//JAM2//GFRA1//KLRB1//LIFR//KLRG1//CD160//GNG7//PIK3C2A//SPRY1//SMAD9//TGFBR3//FMOD//PTN//PTPRD//BAI3//CHRM2//PTGER3//PTGFR//AKAP12//GPR64//LPHN3//CCRL1//ELTD1//CALCRL//GLP2R//PTHLH//PPAP2A//CACNB4//CPE//CNTN1//BOC//ITGA9//ITGA8//ADAMTS1//RCAN2//FCER1A//A2M//ARHGAP6//ITSN1//RAB9B//RHOJ//RERGL//ARHGAP28//RABL3//KSR1//CTNNAL1//AQP1//CRYAB//IL6ST//NME5//FAIM2//KCTD12//CST3//PBX1//TSPYL5//SPDYA//CDH5//DPT//PMP22//SLIT3//PODN//PDK4//SORBS1//MYH10//WASF3//FERMT2//PALMD//PARVA//TEX15//APOD//CASQ2//NLGN1//PLA2G2A//ECM2//VIT//ABI3BP//CCDC80//ANK2//PLN//DPYSL3//EBF1//EBF3//ARID5B//VCAM1//SH3D19//LAMA2//WFDC1//NEXN//PTP4A1//CHRDL1//ABCD2//NNAT//OXCT1//PTGIS//VDAC2//ALDH1A1//KCNMA1//MAP1B//RNF180//CETN2//CNN3//TACC1//EDA2R//LDB2//ARHGEF26//TEAD1//TRIM9//RGS22//GPAM//SRPX//TPM2//CILP//PTX3//APOLD1//ADAMTS9//TMOD2//TTN//FHL5//BEX1//ZNF462//KCNMB4//TIPARP//OGN//ASPA//PDGFD//LIPG//SERPINF1//TIMP3//ANKRD42//TRPC1//CAPN6//DARC |
| GO:0050679 | positive regulation of epithelial cell proliferation | Biological process | 11 | 115 | 428 | 14747 | 3.2957537586347 | 0.000512768212873888 | 0.0139736663268318 | 3.29007890509874 | FGF2//KDR//TEK//VIP//SCG2//FGF10//FGF7//FOXP2//IGF1//SFRP1//OSR1 |
| GO:0050727 | regulation of inflammatory response | Biological process | 15 | 193 | 428 | 14747 | 2.67789695414266 | 0.000517594613286413 | 0.014025049492971 | 3.28601025210783 | C3//FCER1A//A2M//ANXA1//IL6ST//CFH//PTGER3//PTGIS//TEK//CALCRL//FABP4//PLA2G2A//APOD//ANKRD42//CMA1 |
| GO:0032103 | positive regulation of response to external stimulus | Biological process | 13 | 153 | 428 | 14747 | 2.92760063526968 | 0.000521103777123317 | 0.0140403610909667 | 3.28307577891946 | C3//FCER1A//IL6ST//PTGER3//FABP4//PLA2G2A//CCL21//KDR//SLIT2//FGF10//CXCL12//SCG2//LPAR1 |
| GO:0060038 | cardiac muscle cell proliferation | Biological process | 6 | 36 | 428 | 14747 | 5.74260124610592 | 0.000536756791196986 | 0.0143808603214518 | 3.27022245204175 | TGFBR2//FGF2//FGFR1//MEF2C//FOXC1//TGFBR3 |
| GO:0006957 | complement activation, alternative pathway | Biological process | 4 | 14 | 428 | 14747 | 9.84445927903872 | 0.000555661760812331 | 0.0146209866768723 | 3.25518948919557 | C3//C7//CFD//CFH |
| GO:0010882 | regulation of cardiac muscle contraction by calcium ion signaling | Biological process | 4 | 14 | 428 | 14747 | 9.84445927903872 | 0.000555661760812331 | 0.0146209866768723 | 3.25518948919557 | ANK2//CASQ2//DMD//PLN |
| GO:0051208 | sequestering of calcium ion | Biological process | 8 | 65 | 428 | 14747 | 4.24069015097052 | 0.000558968652370211 | 0.0146209866768723 | 3.25261254718402 | FGF2//CCL21//CASQ2 |
| GO:0031032 | actomyosin structure organization | Biological process | 7 | 50 | 428 | 14747 | 4.82378504672897 | 0.000561048555644294 | 0.0146209866768723 | 3.2509995513359 | TMOD1//ACTC1//TTN//MYH11//MYH10//CNN1//CNN3 |
| GO:0034333 | adherens junction assembly | Biological process | 7 | 50 | 428 | 14747 | 4.82378504672897 | 0.000561048555644294 | 0.0146209866768723 | 3.2509995513359 | ARHGAP6//DLC1//SORBS1//KDR//SFRP1//TEK//APOD |
| GO:0006954 | inflammatory response | Biological process | 28 | 493 | 428 | 14747 | 1.95691076946409 | 0.000571023441175511 | 0.0148000586465544 | 3.24334606309529 | C3//FCER1A//A2M//CD44//VCAM1//ANO6//ANXA1//IL6ST//CFH//PTGER3//CMA1//PTGIS//TEK//CALCRL//FABP4//PLA2G2A//APOD//ANKRD42//BDKRB1//DARC//IGFBP4//KIT//PTX3//CCL21//SELP//SCG2//AOC3//KLRG1 |
| GO:0048522 | positive regulation of cellular process | Biological process | 121 | 3186 | 428 | 14747 | 1.30857768508252 | 0.000581457630495376 | 0.0149890348099051 | 3.23548192584088 | FGF2//LPAR1//FGF10//GHR//KIT//BVES//C3//EDNRB//MUSK//TEK//CDON//KDR//VIP//SCG2//CD36//CCL21//SFRP1//FGFR1//TGFBR2//FOXP2//ANGPT1//BDKRB1//SELP//MYOCD//PLAGL1//DLC1//TSPYL2//GNAL//NTRK2//CAP2//FCER1A//CNTFR//CST3//FGF7//IGF1//IL6ST//LIFR//PBX1//PTHLH//PTN//GLP2R//TSPYL5//SPDYA//CAV1//AKAP12//NLGN1//PLA2G2A//ECM2//VIT//ABI3BP//SMOC2//CCDC80//CNTN1//DMD//DPYSL3//EBF1//FOXC1//SMAD9//MEF2C//NFATC2//RORB//FZD4//EBF3//ZRANB1//TAF9B//PPP1R1C//PIK3R1//PTP4A1//TGFBR3//ITGA8//CALCRL//ANXA1//WASF3//ABCD2//NNAT//OXCT1//LAMA2//CLU//RNF180//CD44//PRKAR2B//PTGIS//VCAM1//GPAM//FRZB//KCNMA1//SRPX//SLIT2//SLC44A2//IGFBP4//LDB2//PPAP2B//PTX3//ADAMTS9//BOC//ZEB1//SORBS1//TMOD2//MAP1B//CCNC//EPAS1//PPP1R12A//NFIB//NFIX//NR4A2//TCF21//FHL5//BEX1//ZNF462//OSR1//EDA2R//AQP1//ASPA//SERPINF1//PTPRD//SH3D19//TRPC1//PDGFD//ITSN1//CXCL12//CD34 |
| GO:2000145 | regulation of cell motility | Biological process | 25 | 421 | 428 | 14747 | 2.04605745110662 | 0.000590021496419785 | 0.0150829930561111 | 3.22913216529612 | BDKRB1//SELP//SLIT2//ANGPT1//KDR//TEK//TGFBR3//FGF10//TGFBR2//FGF2//IGF1//LAMA2//NEXN//PIK3R1//PTP4A1//DPYSL3//SFRP1//DLC1//PODN//PPAP2B//FGF7//LPAR1//CCL21//CXCL12//APOD |
| GO:0034329 | cell junction assembly | Biological process | 14 | 175 | 428 | 14747 | 2.75644859813084 | 0.000591427909727989 | 0.0150829930561111 | 3.22809818478986 | TNS1//ARHGAP6//DLC1//SORBS1//KDR//SFRP1//TEK//APOD//CDH5//FLNC//FERMT2//PARVA//CADM3//CADM2 |
| GO:0051270 | regulation of cellular component movement | Biological process | 27 | 470 | 428 | 14747 | 1.979364684828 | 0.000600134154351458 | 0.0152236158622452 | 3.22175165631292 | BDKRB1//SELP//SLIT2//ANGPT1//KDR//TEK//TGFBR3//FGF10//TGFBR2//FGF2//IGF1//LAMA2//NEXN//PIK3R1//PTP4A1//DPYSL3//SFRP1//DLC1//PODN//PPAP2B//FGF7//LPAR1//ANK2//PLN//CCL21//CXCL12//APOD |
| GO:0006939 | smooth muscle contraction | Biological process | 9 | 82 | 428 | 14747 | 3.78171301572829 | 0.000604091116221248 | 0.0152429128743869 | 3.21889755096886 | CAV1//CNN1//MYOCD//EDNRB//ACTA2//PIK3C2A//KCNMA1//CNN3//MYH11 |
| GO:0051094 | positive regulation of developmental process | Biological process | 36 | 699 | 428 | 14747 | 1.77453772411857 | 0.000621484534815608 | 0.0155675568409175 | 3.20656967398583 | FGF2//FGF10//FGFR1//SPRY1//CD36//PLA2G2A//MEF2C//OSR1//CD34//TGFBR2//GHR//SMAD9//FRZB//SFRP1//ADAMTS9//BOC//DMD//ZEB1//CDON//IGF1//IL6ST//AQP1//C3//CMA1//KDR//PTGIS//TEK//MAP1B//ASPA//SERPINF1//NTRK2//SLIT2//PTPRD//MYOCD//NLGN1//LPAR1 |
| GO:0051279 | regulation of release of sequestered calcium ion into cytosol | Biological process | 6 | 37 | 428 | 14747 | 5.58739580702197 | 0.000625162837018109 | 0.0155675568409175 | 3.20400684664401 | CASQ2//DMD//ANK2//PLN//BDKRB1//TRPC1 |
| GO:0019932 | second-messenger-mediated signaling | Biological process | 13 | 156 | 428 | 14747 | 2.87130062305296 | 0.000626750034274724 | 0.0155675568409175 | 3.20290563361973 | RCAN1//RCAN2//CD36//ANK2//CASQ2//DMD//PLN//EDNRB//KDR//NTRK2//FCER1A//PRNP//IGF1 |
| GO:0032835 | glomerulus development | Biological process | 7 | 51 | 428 | 14747 | 4.72920102620487 | 0.000634326378462375 | 0.0156741062118501 | 3.19768722809103 | CD34//ANGPT1//TEK//MEF2C//ACTA2//OSR1//TCF21 |
| GO:0051146 | striated muscle cell differentiation | Biological process | 16 | 218 | 428 | 14747 | 2.52885192489068 | 0.000640006446854986 | 0.0157329419848012 | 3.19381565129823 | KCNH1//CDON//CACNB4//MUSK//MYOCD//MEF2C//DMD//IGF1//TMOD1//ACTC1//TTN//MYH11//BOC//RCAN1//FLNC//MYH10 |
| GO:0002576 | platelet degranulation | Biological process | 9 | 83 | 428 | 14747 | 3.73615020830987 | 0.000660539608470823 | 0.0159642453943135 | 3.18010113531734 | A2M//CD36//CLU//CFD//F13A1//IGF1//SELP//TTN//MMRN1 |
| GO:0022602 | ovulation cycle process | Biological process | 9 | 83 | 428 | 14747 | 3.73615020830987 | 0.000660539608470823 | 0.0159642453943135 | 3.18010113531734 | FOXC1//KDR//KIT//ADAMTS1//SFRP1//FZD4//ANXA1//SLIT3//SLIT2 |
| GO:0001954 | positive regulation of cell-matrix adhesion | Biological process | 5 | 25 | 428 | 14747 | 6.8911214953271 | 0.000661119935837846 | 0.0159642453943135 | 3.17971974664507 | KDR//SFRP1//TEK//CD36//CCL21 |
| GO:0006631 | fatty acid metabolic process | Biological process | 20 | 307 | 428 | 14747 | 2.24466498219124 | 0.000662805742938577 | 0.0159642453943135 | 3.17861373725371 | ABCD2//PTGDS//PTGIS//CH25H//PTPLA//C5ORF4//SCD5//ECH1//CROT//ECI2//PDK4//CAV1//FCER1A//ANXA1//CAB39L//C3//GHR//PRKAR2B//TNXB//GPAM |
| GO:0048738 | cardiac muscle tissue development | Biological process | 12 | 138 | 428 | 14747 | 2.996139780577 | 0.000688747324048562 | 0.0165010172677059 | 3.16194007547693 | MYH11//TTN//ACTC1//MYH10//MYOCD//FOXC1//TGFBR3//MEF2C//TGFBR2//FGF2//FGFR1//PLN |
| GO:0006935 | chemotaxis | Biological process | 32 | 600 | 428 | 14747 | 1.83763239875389 | 0.000695471686057641 | 0.0165010172677059 | 3.15772054624596 | CCL21//KIT//SLIT2//ANK2//CACNB4//CNTN1//DPYSL3//FGFR1//GFRA1//ITGA9//LAMA2//MYH10//MYH11//OPHN1//PRNP//SLIT3//TRPC1//MYL9//CAP2//BOC//CXCL12//SCG2//EDNRB//ANGPT1//FGF2//FGF7//FGF10//KDR//PARVA//LPAR1//NFIB//CCRL1 |
| GO:0042330 | taxis | Biological process | 32 | 600 | 428 | 14747 | 1.83763239875389 | 0.000695471686057641 | 0.0165010172677059 | 3.15772054624596 | CCL21//KIT//SLIT2//FGF2//CXCL12//CCRL1//ANK2//CACNB4//CNTN1//DPYSL3//FGFR1//GFRA1//ITGA9//LAMA2//MYH10//MYH11//OPHN1//PRNP//SLIT3//TRPC1//MYL9//CAP2//BOC//SCG2//EDNRB//ANGPT1//FGF7//FGF10//KDR//PARVA//LPAR1//NFIB |
| GO:0034330 | cell junction organization | Biological process | 15 | 199 | 428 | 14747 | 2.59715634245996 | 0.000712089667378465 | 0.0167713658895039 | 3.14746531591967 | TNS1//CDH5//FLNC//FERMT2//PARVA//CADM3//CADM2//HEG1//ARHGAP6//DLC1//SORBS1//KDR//SFRP1//TEK//APOD |
| GO:0022008 | neurogenesis | Biological process | 52 | 1138 | 428 | 14747 | 1.57442143126981 | 0.000715296874755646 | 0.0167713658895039 | 3.1455136724522 | FGFR1//MYH10//NTRK2//NR4A2//NR2F1//DCLK1//ANK2//CACNB4//CNTN1//DPYSL3//GFRA1//ITGA9//LAMA2//MYH11//OPHN1//PRNP//SLIT3//TRPC1//SLIT2//MYL9//CAP2//BOC//CXCL12//FGF2//IGF1//NFIB//DMD//LPAR1//PMP22//WASF3//APOD//EDNRB//MAP1B//FAIM2//ASPA//MEF2C//NNAT//PTPRD//FZD4//PRICKLE2//ANXA1//RORB//KCNMA1//CLN5//NLGN1//PBX1//ZEB1//CDON//IL6ST//WEE1//SERPINF1//TACC1 |
| GO:0060402 | calcium ion transport into cytosol | Biological process | 9 | 84 | 428 | 14747 | 3.69167222963952 | 0.000721224303946274 | 0.0167713658895039 | 3.14192964676951 | CAV1//CASQ2//DMD//ANK2//PLN//FGF2//CCL21//BDKRB1//TRPC1 |
| GO:0001974 | blood vessel remodeling | Biological process | 6 | 38 | 428 | 14747 | 5.44035907525824 | 0.000724449858091381 | 0.0167713658895039 | 3.13999166831264 | CST3//EPAS1//FGF10//FOXC1//IGF1//MEF2C |
| GO:0014855 | striated muscle cell proliferation | Biological process | 6 | 38 | 428 | 14747 | 5.44035907525824 | 0.000724449858091381 | 0.0167713658895039 | 3.13999166831264 | FOXC1//TGFBR3//TGFBR2//FGF2//FGFR1//MEF2C |
| GO:0001656 | metanephros development | Biological process | 9 | 85 | 428 | 14747 | 3.64824079164376 | 0.000786380241911861 | 0.0180300354503734 | 3.10436740684485 | FGF10//OSR1//TCF21//CD34//FOXC1//ITGA8//SLIT2//SPRY1//RDH10 |
| GO:0060401 | cytosolic calcium ion transport | Biological process | 9 | 85 | 428 | 14747 | 3.64824079164376 | 0.000786380241911861 | 0.0180300354503734 | 3.10436740684485 | CAV1//CASQ2//DMD//ANK2//PLN//FGF2//CCL21//BDKRB1//TRPC1 |
| GO:0000902 | cell morphogenesis | Biological process | 45 | 950 | 428 | 14747 | 1.63210772257747 | 0.000798745621468798 | 0.0180639431523388 | 3.09759150952481 | TGFBR3//HEG1//ANK2//CACNB4//CNTN1//DPYSL3//FGFR1//GFRA1//ITGA9//LAMA2//MYH10//MYH11//OPHN1//PRNP//SLIT3//TRPC1//SLIT2//MYL9//CAP2//BOC//CXCL12//KDR//KIT//DLC1//WASF3//FERMT2//BVES//PALMD//PARVA//RHOJ//SFRP1//APOD//FOXC1//NR4A2//DCLK1//NFIB//ZRANB1//SH3D19//MEF2C//TEK//FZD4//MAP1B//WEE1//NTRK2//PTPRD |
| GO:0051893 | regulation of focal adhesion assembly | Biological process | 5 | 26 | 428 | 14747 | 6.62607836089144 | 0.000799222479585548 | 0.0180639431523388 | 3.09733230928826 | KDR//SFRP1//TEK//APOD//ARHGAP6 |
| GO:0090109 | regulation of cell-substrate junction assembly | Biological process | 5 | 26 | 428 | 14747 | 6.62607836089144 | 0.000799222479585548 | 0.0180639431523388 | 3.09733230928826 | KDR//SFRP1//TEK//APOD//ARHGAP6 |
| GO:0010522 | regulation of calcium ion transport into cytosol | Biological process | 7 | 53 | 428 | 14747 | 4.55074061012167 | 0.00080357407411186 | 0.018076626223771 | 3.09497408343672 | CAV1//CASQ2//DMD//ANK2//PLN//BDKRB1//TRPC1 |
| GO:0006793 | phosphorus metabolic process | Biological process | 99 | 2530 | 428 | 14747 | 1.34826290125965 | 0.000813649542650601 | 0.0182173458633836 | 3.08956261534009 | CCNC//DIRAS3//FGF2//LPAR1//FGF10//GHR//KIT//CAV1//BDKRB1//PRNP//PPAP2B//SLIT2//C3//EDNRB//MUSK//TEK//CDON//ERRFI1//CALCRL//AQP1//NME5//GNAL//GNG11//RHOJ//PDE8B//PDE7B//ACTC1//BMX//ERG//FGFR1//SMAD9//PDK4//PIK3R1//MAPK4//TGFBR2//DYRK3//KSR1//DCLK1//HIPK3//FABP4//PPP1R12A//PTPRD//PTPLA//DPM1//GPAM//PIK3C2A//PLA2G2A//UGCG//PPAP2A//PEMT//SLC44A2//TSPYL2//ANGPT1//NTRK2//CAP2//FCER1A//CACNB4//PTHLH//LIPG//VIP//MEF2C//EFEMP1//KDR//WEE1//AKAP12//AMPD1//BVES//ALDH1A1//DLC1//CNN3//PLN//SFRP1//PREX2//CCL21//DMD//CD44//PRKAR2B//SPRY1//PTP4A1//IL6ST//IGF1//ARHGAP6//TPM2//TNXB//FZD4//RGS5//RGS22//RCAN1//MYOCD//SPDYA//GNG7//TTN//CMAHP//PAPSS2//PDGFD//CD36//FGF7//TGFBR3//FMO1 |
| GO:0030049 | muscle filament sliding | Biological process | 6 | 39 | 428 | 14747 | 5.30086268871316 | 0.00083550023430468 | 0.0185325610111582 | 3.07805342397859 | ACTC1//DES//DMD//TMOD1//TPM2//TTN |
| GO:0033275 | actin-myosin filament sliding | Biological process | 6 | 39 | 428 | 14747 | 5.30086268871316 | 0.00083550023430468 | 0.0185325610111582 | 3.07805342397859 | ACTC1//DES//DMD//TMOD1//TPM2//TTN |
| GO:0000165 | MAPK cascade | Biological process | 29 | 531 | 428 | 14747 | 1.88175634053188 | 0.000851410230095394 | 0.0187980342005784 | 3.06986113587454 | FGF2//LPAR1//FGF10//GHR//KIT//CAV1//FCER1A//FGFR1//SPRY1//CD36//IGF1//IGFBP4//KDR//NTRK2//CDON//TNXB//FZD4//SFRP1//HIPK3//CCL21//EDA2R//CRYAB//MEF2C//ANGPT1//CD44//TEK//MAPK4//SCG2//DOK5 |
| GO:0030817 | regulation of cAMP biosynthetic process | Biological process | 9 | 86 | 428 | 14747 | 3.60581938708976 | 0.000856250196470364 | 0.0188177750551482 | 3.06739931584873 | GNAL//NTRK2//CAP2//EDNRB//VIP//PTHLH//AKAP12//CALCRL//GNG7 |
| GO:0002673 | regulation of acute inflammatory response | Biological process | 7 | 54 | 428 | 14747 | 4.46646763586016 | 0.000900598716173984 | 0.0197016297129988 | 3.0454686764703 | C3//FCER1A//A2M//ANXA1//IL6ST//CFH//PTGER3 |
| GO:0042325 | regulation of phosphorylation | Biological process | 42 | 876 | 428 | 14747 | 1.65198118038663 | 0.00094482945103048 | 0.0205742557873377 | 3.02464657790441 | CCNC//DIRAS3//FGF2//LPAR1//FGF10//GHR//KIT//CAV1//BDKRB1//PRNP//PPAP2B//SLIT2//C3//EDNRB//MUSK//TEK//CDON//ERRFI1//FABP4//TSPYL2//ANGPT1//FCER1A//TGFBR2//DMD//CD44//PRKAR2B//IL6ST//IGF1//FGFR1//SPRY1//TNXB//FZD4//SFRP1//HIPK3//PIK3R1//CCL21//MYOCD//SPDYA//TTN//PDGFD//CD36//FGF7 |
| GO:0007507 | heart development | Biological process | 23 | 387 | 428 | 14747 | 2.04774928155715 | 0.000956766261446085 | 0.0205742557873377 | 3.01919414765974 | MEF2C//TGFBR3//DLC1//PARVA//HEG1//CPE//MYOCD//ANK2//MYH10//TTN//FOXC1//PLN//MYH11//ACTC1//TGFBR2//FGF2//FGFR1//TEK//ADAMTS1//OXCT1//VCAM1//CALCRL//OSR1 |
| GO:0034394 | protein localization to cell surface | Biological process | 5 | 27 | 428 | 14747 | 6.3806680512288 | 0.000957744765105677 | 0.0205742557873377 | 3.0187502131292 | ANGPT1//ANK2//FGF7//FGF10//FBLN5 |
| GO:0043551 | regulation of phosphatidylinositol 3-kinase activity | Biological process | 5 | 27 | 428 | 14747 | 6.3806680512288 | 0.000957744765105677 | 0.0205742557873377 | 3.0187502131292 | FGF2//KIT//CCL21//TEK//PIK3R1 |
| GO:0046903 | secretion | Biological process | 39 | 796 | 428 | 14747 | 1.68815162259898 | 0.000962984277676483 | 0.0205811850313755 | 3.01638080339892 | FCER1A//A2M//CD36//CLU//CFD//F13A1//IGF1//SELP//TTN//MMRN1//CORIN//EDNRB//NLGN1//MYH10//PIK3C2A//EXOC6//VIP//CAV1//PRKAR2B//AQP1//SCG2//CBLN4//NTRK2//CACNB4//TRIM9//ANXA1//NNAT//OXCT1//FZD4//KIT//KCNMA1//TRPC1//SFRP1//PDE8B//FGF10//KCNMB4//GPAM//FGF7//CD34 |
| GO:0042060 | wound healing | Biological process | 32 | 612 | 428 | 14747 | 1.80160039093519 | 0.000966698563017007 | 0.0205811850313755 | 3.0147089269789 | MYH10//CD44//ANO6//A2M//ANGPT1//CAV1//CD36//CLU//CFD//F13A1//IGF1//KCNMA1//PDE1A//PIK3R1//PRKAR2B//SELP//TEK//TTN//WEE1//TFPI2//PAPSS2//MMRN1//KCNMB4//PLSCR4//JAM2//CD34//APOD//FGF10//GATM//TGFBR2//FGF2 |
| GO:0030814 | regulation of cAMP metabolic process | Biological process | 9 | 88 | 428 | 14747 | 3.52386894647409 | 0.00101114186409399 | 0.0213323618426547 | 2.99518790823593 | GNAL//NTRK2//CAP2//EDNRB//VIP//PTHLH//AKAP12//CALCRL//GNG7 |
| GO:0008360 | regulation of cell shape | Biological process | 10 | 106 | 428 | 14747 | 3.25052900722977 | 0.00101540074193387 | 0.0213323618426547 | 2.99336252360064 | KDR//KIT//MYH10//DLC1//WASF3//FERMT2//BVES//PALMD//PARVA//RHOJ |
| GO:0048747 | muscle fiber development | Biological process | 10 | 106 | 428 | 14747 | 3.25052900722977 | 0.00101540074193387 | 0.0213323618426547 | 2.99336252360064 | CACNB4//MUSK//MYOCD//MEF2C//BOC//MYH11//TTN//RCAN1//DMD//FLNC |
| GO:0050731 | positive regulation of peptidyl-tyrosine phosphorylation | Biological process | 11 | 125 | 428 | 14747 | 3.03209345794393 | 0.00103247426915771 | 0.0215385655829034 | 2.98612076282126 | IL6ST//KIT//GHR//IGF1//ANGPT1//CD36//CD44//FCER1A//FGF7//FGF10//PPAP2B |
| GO:0030335 | positive regulation of cell migration | Biological process | 16 | 228 | 428 | 14747 | 2.41793736678144 | 0.00103424858848498 | 0.0215385655829034 | 2.98537506314563 | BDKRB1//SELP//ANGPT1//KDR//TEK//FGF10//TGFBR2//IGF1//FGF2//PPAP2B//FGF7//LPAR1//CCL21//CXCL12//PIK3R1//PTP4A1 |
| GO:0010562 | positive regulation of phosphorus metabolic process | Biological process | 34 | 667 | 428 | 14747 | 1.75635780240721 | 0.00104641143583868 | 0.0216031867424877 | 2.98029752275213 | FGF2//LPAR1//FGF10//GHR//KIT//C3//EDNRB//MUSK//TEK//CDON//TSPYL2//ANGPT1//GNAL//NTRK2//CAP2//FCER1A//VIP//PTHLH//AKAP12//CALCRL//TGFBR2//CAV1//CD44//PRKAR2B//DLC1//IL6ST//IGF1//FGFR1//FZD4//SPDYA//CCL21//CD36//FGF7//PPAP2B |
| GO:0045937 | positive regulation of phosphate metabolic process | Biological process | 34 | 667 | 428 | 14747 | 1.75635780240721 | 0.00104641143583868 | 0.0216031867424877 | 2.98029752275213 | FGF2//LPAR1//FGF10//GHR//KIT//C3//EDNRB//MUSK//TEK//CDON//TSPYL2//ANGPT1//GNAL//NTRK2//CAP2//FCER1A//VIP//PTHLH//AKAP12//CALCRL//TGFBR2//CAV1//CD44//PRKAR2B//DLC1//IL6ST//IGF1//FGFR1//FZD4//SPDYA//CCL21//CD36//FGF7//PPAP2B |
| GO:0008284 | positive regulation of cell proliferation | Biological process | 34 | 668 | 428 | 14747 | 1.75372852425989 | 0.00107339617780004 | 0.0220647688445189 | 2.9692399555026 | FGF2//KDR//TEK//VIP//SCG2//FGFR1//TGFBR2//FOXP2//FGF7//FGF10//IGF1//MEF2C//NFATC2//IL6ST//VCAM1//GPAM//AQP1//CALCRL//SFRP1//OSR1//CDON//CNTFR//CST3//EDNRB//KIT//LIFR//NTRK2//PBX1//PTHLH//PTN//GLP2R//TSPYL5//MYOCD//SPDYA |
| GO:0042698 | ovulation cycle | Biological process | 9 | 89 | 428 | 14747 | 3.48427491336764 | 0.00109668766646962 | 0.0224467960574833 | 2.95991704065003 | FOXC1//KDR//KIT//ADAMTS1//SFRP1//FZD4//ANXA1//SLIT3//SLIT2 |
| GO:0071804 | cellular potassium ion transport | Biological process | 5 | 28 | 428 | 14747 | 6.1527870493992 | 0.00113848854503891 | 0.0231040505161301 | 2.94367133470314 | ABCC9//AQP1//KCNMA1//CASQ2//ANK2 |
| GO:0071805 | potassium ion transmembrane transport | Biological process | 5 | 28 | 428 | 14747 | 6.1527870493992 | 0.00113848854503891 | 0.0231040505161301 | 2.94367133470314 | ABCC9//CASQ2//ANK2//AQP1//KCNMA1 |
| GO:0048732 | gland development | Biological process | 18 | 275 | 428 | 14747 | 2.25527612574342 | 0.00115088676337996 | 0.0232566905701654 | 2.9389674048311 | CST3//FGF10//CAV1//PBX1//ARID5B//IGF1//FOXC1//SFRP1//FRZB//FGF2//CD44//TGFBR2//FGF7//FGFR1//PTHLH//SLIT2//SERPINF1//TCF21 |
| GO:0035265 | organ growth | Biological process | 9 | 90 | 428 | 14747 | 3.44556074766355 | 0.00118799558960465 | 0.023905278341032 | 2.9251851716561 | FOXC1//TGFBR3//TGFBR2//FGF2//FGFR1//MEF2C//FGF7//FGF10//MYOCD |
| GO:0010880 | regulation of release of sequestered calcium ion into cytosol by sarcoplasmic reticulum | Biological process | 4 | 17 | 428 | 14747 | 8.10720175920835 | 0.00123295746910743 | 0.0247057738242577 | 2.90905190414273 | ANK2//CASQ2//DMD//PLN |
| GO:0006790 | sulfur compound metabolic process | Biological process | 16 | 232 | 428 | 14747 | 2.3762487914921 | 0.0012415303964569 | 0.0247734663627739 | 2.90604264312359 | MAT2A//ADI1//PAPSS2//GSTA2//GSTM5//UST//FMOD//OMD//OGN//GHR//MAMDC2//GPC5//GPC3//GPC6//ANGPT1//IGF1 |
| GO:0061138 | morphogenesis of a branching epithelium | Biological process | 14 | 189 | 428 | 14747 | 2.55226722049152 | 0.00125468901692162 | 0.0249317163404134 | 2.90146390364196 | CXCL12//TGFBR2//CD44//FGF2//PBX1//TCF21//FGF10//IGF1//KDR//SLIT2//RDH10//FGF7//FGFR1//SFRP1 |
| GO:2000147 | positive regulation of cell motility | Biological process | 16 | 233 | 428 | 14747 | 2.36605029882476 | 0.00129851154742966 | 0.0255902296808729 | 2.88655418391478 | BDKRB1//SELP//ANGPT1//KDR//TEK//FGF10//TGFBR2//IGF1//PIK3R1//PTP4A1//FGF2//PPAP2B//FGF7//LPAR1//CCL21//CXCL12 |
| GO:0000904 | cell morphogenesis involved in differentiation | Biological process | 35 | 702 | 428 | 14747 | 1.71787216763852 | 0.00129856061706254 | 0.0255902296808729 | 2.88653777261048 | TGFBR3//HEG1//ANK2//CACNB4//CNTN1//DPYSL3//FGFR1//GFRA1//ITGA9//LAMA2//MYH10//MYH11//OPHN1//PRNP//SLIT3//TRPC1//SLIT2//MYL9//CAP2//BOC//CXCL12//SFRP1//APOD//FOXC1//NR4A2//DCLK1//NFIB//MEF2C//TEK//FZD4//BVES//PARVA//MAP1B//NTRK2//PTPRD |
| GO:0003018 | vascular process in circulatory system | Biological process | 11 | 129 | 428 | 14747 | 2.93807505614721 | 0.00133625506528235 | 0.026147673576683 | 2.87411063551936 | VIP//EDNRB//ACTA2//PIK3C2A//KCNMB4//CAV1//TEK//ANGPT1//SLIT2//FOXC1//KCNMA1 |
| GO:0007157 | heterophilic cell-cell adhesion | Biological process | 5 | 29 | 428 | 14747 | 5.94062197873026 | 0.00134329629404222 | 0.026147673576683 | 2.8718281834035 | PTPRD//SELP//VCAM1//NLGN1//CADM3 |
| GO:0070098 | chemokine-mediated signaling pathway | Biological process | 5 | 29 | 428 | 14747 | 5.94062197873026 | 0.00134329629404222 | 0.026147673576683 | 2.8718281834035 | SLIT3//SLIT2//DARC//CXCL12//CCRL1 |
| GO:0065008 | regulation of biological quality | Biological process | 99 | 2569 | 428 | 14747 | 1.32779491638261 | 0.00135969180005027 | 0.0263592284326819 | 2.86655952156075 | KCNMB4//ANXA1//MEF2C//CD34//PLN//ANK2//CASQ2//DMD//EPAS1//RDH10//ANO6//CORIN//CAV1//EDNRB//AQP1//A2M//ANGPT1//CD36//CLU//CFD//F13A1//IGF1//KCNMA1//PDE1A//PIK3R1//PRKAR2B//SELP//TEK//TTN//WEE1//TFPI2//PAPSS2//MMRN1//PLSCR4//JAM2//NLGN1//FGF2//CACNB4//CXCL12//PRNP//CYBRD1//PDK4//SLC9A9//CLN5//BDKRB1//LPAR1//VIP//TIPARP//ACTA2//KDR//KIT//MYH10//DLC1//WASF3//FERMT2//BVES//PALMD//PARVA//RHOJ//MUSK//LDB2//PIK3C2A//SLC22A17//TRIM9//PPAP2B//TENC1//ASPA//CPE//DYRK3//EXOC6//SLIT2//CCL21//PTGER3//HTN3//NNAT//OXCT1//PMP22//GCNT4//MPDZ//ALDH1A1//FABP4//LIPG//FZD4//PTHLH//KCNIP1//MAP1B//TRPC1//SFRP1//PDE8B//FGF10//NTRK2//TEX15//HEG1//FOXC1//GPAM//TGFBR3//CTSG//CMA1//VCAM1 |
| GO:0048645 | organ formation | Biological process | 7 | 58 | 428 | 14747 | 4.15843538511118 | 0.00138450692622662 | 0.0267108166452141 | 2.85870486728245 | FGF2//FGF10//FGFR1//SPRY1//MEF2C//RDH10//TGFBR2 |
| GO:0006171 | cAMP biosynthetic process | Biological process | 9 | 92 | 428 | 14747 | 3.37065725314913 | 0.00138902967666452 | 0.0267108166452141 | 2.85728847544744 | GNAL//NTRK2//CAP2//EDNRB//VIP//PTHLH//AKAP12//CALCRL//GNG7 |
| GO:0044255 | cellular lipid metabolic process | Biological process | 40 | 840 | 428 | 14747 | 1.64074321317312 | 0.00141627887613754 | 0.027125437591566 | 2.8488512223223 | ABCD2//PTGDS//PTGIS//RDH10//DPM1//C3//GHR//PRKAR2B//TNXB//CROT//GPAM//CH25H//PTPLA//C5ORF4//SCD5//ECH1//CAV1//PIK3C2A//PIK3R1//PLA2G2A//UGCG//PPAP2A//PPAP2B//PEMT//SLC44A2//FGF2//ST6GALNAC3//KIT//ECI2//GLT25D2//LIPG//PDK4//FCER1A//FABP4//ANXA1//ALDH1A1//CAB39L//APOD//CD36//OXCT1 |
| GO:2000021 | regulation of ion homeostasis | Biological process | 12 | 150 | 428 | 14747 | 2.75644859813084 | 0.00142937748663521 | 0.0272668049350533 | 2.84485306252245 | CORIN//EDNRB//CAV1//CASQ2//DMD//ANK2//PLN//WASF3//FGF2//CCL21//BDKRB1//TRPC1 |
| GO:0052652 | cyclic purine nucleotide metabolic process | Biological process | 10 | 111 | 428 | 14747 | 3.10410878167888 | 0.00144569663048723 | 0.0274308792657944 | 2.8399228311964 | CALCRL//AQP1//GNAL//NTRK2//CAP2//EDNRB//VIP//PTHLH//AKAP12//GNG7 |
| GO:0051238 | sequestering of metal ion | Biological process | 8 | 75 | 428 | 14747 | 3.67526479750779 | 0.00145280398733891 | 0.0274308792657944 | 2.83779297686476 | CASQ2//FGF2//CCL21 |
| GO:0050900 | leukocyte migration | Biological process | 17 | 258 | 428 | 14747 | 2.27033072520467 | 0.00145523431626043 | 0.0274308792657944 | 2.83706707261223 | CCL21//KIT//BDKRB1//SELP//SLIT2//ITGA9//SCG2//EDNRB//VCAM1//CXCL12//APOD//ANGPT1//CAV1//CD34//PIK3R1//TEK//JAM2 |
| GO:0006937 | regulation of muscle contraction | Biological process | 10 | 112 | 428 | 14747 | 3.0763935246996 | 0.00154736622440443 | 0.0288601308683447 | 2.81040688706668 | CAV1//CNN1//MYOCD//ANK2//CASQ2//DMD//PLN//KCNMA1//CALCRL//MYL9 |
| GO:0009190 | cyclic nucleotide biosynthetic process | Biological process | 10 | 112 | 428 | 14747 | 3.0763935246996 | 0.00154736622440443 | 0.0288601308683447 | 2.81040688706668 | CALCRL//AQP1//GNAL//NTRK2//CAP2//EDNRB//VIP//PTHLH//AKAP12//GNG7 |
| GO:0042522 | regulation of tyrosine phosphorylation of Stat5 protein | Biological process | 4 | 18 | 428 | 14747 | 7.65680166147456 | 0.00154921230914159 | 0.0288601308683447 | 2.80988906102043 | GHR//IGF1//KIT//CAV1 |
| GO:0051145 | smooth muscle cell differentiation | Biological process | 6 | 44 | 428 | 14747 | 4.69849192863212 | 0.00160013028939257 | 0.0296926900782613 | 2.79584465380625 | EDNRB//MYOCD//RCAN1//ITGA8//FGF10//MEF2C |
| GO:0051272 | positive regulation of cellular component movement | Biological process | 16 | 239 | 428 | 14747 | 2.30665154655301 | 0.00168879028105462 | 0.0310367669559659 | 2.77242427904697 | BDKRB1//SELP//ANGPT1//KDR//TEK//FGF10//TGFBR2//IGF1//PIK3R1//PTP4A1//FGF2//PPAP2B//FGF7//LPAR1//CCL21//CXCL12 |
| GO:0030816 | positive regulation of cAMP metabolic process | Biological process | 7 | 60 | 428 | 14747 | 4.01982087227414 | 0.00169236195760776 | 0.0310367669559659 | 2.77150674568222 | GNAL//NTRK2//CAP2//VIP//PTHLH//AKAP12//CALCRL |
| GO:0030819 | positive regulation of cAMP biosynthetic process | Biological process | 7 | 60 | 428 | 14747 | 4.01982087227414 | 0.00169236195760776 | 0.0310367669559659 | 2.77150674568222 | GNAL//NTRK2//CAP2//VIP//PTHLH//AKAP12//CALCRL |
| GO:0010038 | response to metal ion | Biological process | 15 | 217 | 428 | 14747 | 2.38172401912227 | 0.0017051023154672 | 0.0310367669559659 | 2.76824955584473 | KCNMB4//KCNIP1//CYBRD1//PRNP//GATM//SOD3//CAV1//KCNMA1//TRPC1//TTN//MT1A//FOSB//MEF2C//AQP1//FABP4 |
| GO:0019216 | regulation of lipid metabolic process | Biological process | 15 | 217 | 428 | 14747 | 2.38172401912227 | 0.0017051023154672 | 0.0310367669559659 | 2.76824955584473 | PDK4//C3//SORBS1//CAV1//ANXA1//ABCD2//KCNMA1//PIK3R1//FGF2//KIT//CCL21//TEK//CAB39L//APOD//PPAP2A |
| GO:0080134 | regulation of response to stress | Biological process | 36 | 741 | 428 | 14747 | 1.673956638541 | 0.0017377301860051 | 0.0315104002169518 | 2.76001765471457 | C3//FCER1A//A2M//COLEC12//MEF2C//ANO6//ANXA1//IL6ST//TEX15//SELP//CAV1//CD36//CD34//CFH//PTGER3//TGFBR2//TNXB//FZD4//SFRP1//HIPK3//CD44//PPAP2B//FGF10//SH2D1A//CCL21//EDA2R//CMA1//PTGIS//TEK//CALCRL//FABP4//PLA2G2A//FGF2//APOD//ANKRD42//FBLN5 |
| GO:0003205 | cardiac chamber development | Biological process | 10 | 114 | 428 | 14747 | 3.0224217084768 | 0.00176811852412013 | 0.0319399895512458 | 2.75248862583537 | PARVA//HEG1//MEF2C//CPE//MYOCD//ANK2//FOXC1//TGFBR3//TEK//ADAMTS1 |
| GO:0007163 | establishment or maintenance of cell polarity | Biological process | 10 | 115 | 428 | 14747 | 2.996139780577 | 0.00188767569661977 | 0.0339710392346403 | 2.72407261558152 | CCL21//WEE1//AQP1//IGF1//TEK//PRICKLE2//MAP1B//CDH5//CAP2//PARVA |
| GO:1901137 | carbohydrate derivative biosynthetic process | Biological process | 33 | 665 | 428 | 14747 | 1.7098271379383 | 0.00191409782958218 | 0.0343170396589377 | 2.71803586919139 | GPC5//GPC3//GPC6//CALCRL//AQP1//NME5//TIPARP//B3GALT2//ST6GALNAC3//DPM1//GCNT4//MAT2A//UGCG//GNAL//NTRK2//CAP2//EDNRB//GLT25D2//CCL21//VIP//FMOD//OMD//OGN//MAN1A1//UBE2J1//IGF1//UST//ANGPT1//PTHLH//AKAP12//AMPD1//GNG7//PAPSS2 |
| GO:0006163 | purine nucleotide metabolic process | Biological process | 34 | 693 | 428 | 14747 | 1.6904627044814 | 0.00197789349385253 | 0.0353279927797105 | 2.70379709811674 | CALCRL//AQP1//NME5//GNAL//GNG11//RHOJ//PDE8B//PDE7B//ACTC1//NTRK2//CAP2//EDNRB//CACNB4//PTHLH//VIP//AKAP12//AMPD1//BVES//ALDH1A1//DLC1//ERRFI1//CNN3//PLN//FGF10//SFRP1//PREX2//CCL21//SPRY1//ARHGAP6//TPM2//RGS5//RGS22//GNG7//PAPSS2 |
| GO:0030902 | hindbrain development | Biological process | 10 | 116 | 428 | 14747 | 2.97031098936513 | 0.00201367850626653 | 0.0358329581954667 | 2.69600986551009 | CNTN1//PTPRS//FAIM2//FOXP2//DLC1//MYH10//FGF2//IGF1//FZD4//SMAD9 |
| GO:0014902 | myotube differentiation | Biological process | 6 | 46 | 428 | 14747 | 4.4942096708655 | 0.00202234884499667 | 0.0358534633523759 | 2.69414392867097 | KCNH1//CDON//MYOCD//DMD//IGF1//MEF2C |
| GO:0010863 | positive regulation of phospholipase C activity | Biological process | 7 | 62 | 428 | 14747 | 3.89014923123304 | 0.00205093936755437 | 0.0362256660883955 | 2.68804717860082 | ARHGAP6//LPAR1//PDE1A//PRKAR2B//FGF2//FGFR1//KIT |
| GO:0043550 | regulation of lipid kinase activity | Biological process | 5 | 32 | 428 | 14747 | 5.3836886682243 | 0.00212101382689515 | 0.0372016420994758 | 2.67345650030725 | PIK3R1//FGF2//KIT//CCL21//TEK |
| GO:1901136 | carbohydrate derivative catabolic process | Biological process | 29 | 564 | 428 | 14747 | 1.77165357592629 | 0.00212179632020495 | 0.0372016420994758 | 2.67329630811451 | GPC5//GPC3//GPC6//GNAL//GNG11//RHOJ//PDE8B//PDE7B//ACTC1//ADAMTS9//CD44//FGF2//LYVE1//NTRK2//BVES//ALDH1A1//DLC1//ERRFI1//SFRP1//PREX2//CCL21//SPRY1//FMOD//OMD//OGN//ARHGAP6//RGS5//RGS22//CST3 |
| GO:0001763 | morphogenesis of a branching structure | Biological process | 14 | 200 | 428 | 14747 | 2.41189252336449 | 0.00213771142814387 | 0.037343391211788 | 2.67005092102773 | CXCL12//TGFBR2//CD44//FGF2//PBX1//TCF21//FGF10//IGF1//KDR//SLIT2//RDH10//FGF7//FGFR1//SFRP1 |
| GO:0030802 | regulation of cyclic nucleotide biosynthetic process | Biological process | 9 | 98 | 428 | 14747 | 3.16429048254816 | 0.00216118172795821 | 0.0376156046008493 | 2.66530871292651 | GNAL//NTRK2//CAP2//EDNRB//VIP//PTHLH//AKAP12//CALCRL//GNG7 |
| GO:0033993 | response to lipid | Biological process | 28 | 539 | 428 | 14747 | 1.78990168709795 | 0.00217446384448882 | 0.0377091566340625 | 2.66264780909294 | ANXA1//CRYAB//CST3//FGF10//GHR//WFDC1//BDKRB1//CTSG//FMO1//PTGFR//SELP//CAV1//CCL21//TGFBR3//RCAN1//TGFBR2//PTGDS//SLIT3//SLIT2//CD36//EDNRB//MEF2C//PLSCR4//AQP1//FZD4//OSR1//SFRP1//PDK4 |
| GO:1900274 | regulation of phospholipase C activity | Biological process | 7 | 63 | 428 | 14747 | 3.82840083073728 | 0.00225092131756742 | 0.0388936368241994 | 2.64763968578292 | ARHGAP6//LPAR1//PDE1A//PRKAR2B//FGF2//FGFR1//KIT |
| GO:0044708 | single-organism behavior | Biological process | 20 | 340 | 428 | 14747 | 2.02680043980209 | 0.00226882379876915 | 0.0390614465571483 | 2.64419923095229 | GNG7//MEF2C//PRNP//VIP//TMOD2//NTRK2//PRKAR2B//PTN//MUSK//ITGA8//SEPP1//SOBP//CACNB4//KCNMA1//NR4A2//TMOD1//FZD4//FOXP2//CST3//PTGDS |
| GO:0042506 | tyrosine phosphorylation of Stat5 protein | Biological process | 4 | 20 | 428 | 14747 | 6.8911214953271 | 0.00234288287286952 | 0.0399549067431742 | 2.63024942243075 | GHR//IGF1//KIT//CAV1 |
| GO:0048745 | smooth muscle tissue development | Biological process | 4 | 20 | 428 | 14747 | 6.8911214953271 | 0.00234288287286952 | 0.0399549067431742 | 2.63024942243075 | OSR1//ITGA8//TIPARP//FOXP2 |
| GO:0016310 | phosphorylation | Biological process | 55 | 1288 | 428 | 14747 | 1.47131864224763 | 0.00234585319523774 | 0.0399549067431742 | 2.62969916975377 | CCNC//DIRAS3//FGF2//LPAR1//FGF10//GHR//KIT//CAV1//BDKRB1//PRNP//PPAP2B//SLIT2//C3//EDNRB//MUSK//TEK//CDON//ERRFI1//BMX//ERG//FGFR1//SMAD9//PDK4//PIK3R1//MAPK4//TGFBR2//DYRK3//KSR1//DCLK1//HIPK3//FABP4//TSPYL2//ANGPT1//FCER1A//EFEMP1//KDR//WEE1//DMD//CD44//PRKAR2B//IL6ST//IGF1//SPRY1//TNXB//FZD4//SFRP1//CCL21//MYOCD//SPDYA//TTN//NTRK2//PDGFD//CD36//FGF7//TGFBR3 |
| GO:0044710 | single-organism metabolic process | Biological process | 114 | 3083 | 428 | 14747 | 1.27406398064757 | 0.00235515948704859 | 0.0399706604759243 | 2.62797967791943 | ABCD2//MAT2A//ADI1//PTGDS//PTGIS//RDH10//IL6ST//GPC5//GPC3//GPC6//ALDH1A1//FMO1//PDK4//GHR//GNG7//GNG11//PRKAR2B//CYB5A//ERO1LB//CYBRD1//GSTA2//GSTM5//AMPD1//CALCRL//AQP1//NME5//GNAL//RHOJ//PDE8B//PDE7B//ACTC1//DPYSL3//DPM1//ASPA//CD34//GATM//APOD//CD36//CLU//FABP4//PPAP2A//PPAP2B//CH25H//LIPG//PEMT//C3//TNXB//CROT//GPAM//PTPLA//C5ORF4//SCD5//ECH1//CAV1//PIK3C2A//PIK3R1//PLA2G2A//UGCG//SLC44A2//FGF2//ST6GALNAC3//KIT//PBX1//NTRK2//CAP2//EDNRB//TIPARP//SERPINA6//CACNB4//PTHLH//ECI2//GLT25D2//IGF1//VIP//CST3//PLCB4//PLCXD3//SORBS1//FMOD//OMD//OGN//FCER1A//BCHE//MAMDC2//UST//ANGPT1//CD44//LYVE1//AKAP12//ANXA1//BVES//DLC1//ERRFI1//KCNMA1//CNN3//PLN//FGF10//SFRP1//PREX2//CCL21//TENC1//SPRY1//NR4A2//OXCT1//GCNT4//EPAS1//ARHGAP6//TPM2//RGS5//RGS22//TEK//PAPSS2//CAB39L//CMAHP |
| GO:0032879 | regulation of localization | Biological process | 58 | 1376 | 428 | 14747 | 1.45234391980004 | 0.0023841578074882 | 0.0401890093219012 | 2.62266500201355 | ANGPT1//BDKRB1//SELP//SLIT2//CORIN//EDNRB//CASQ2//VIP//PRKAR2B//AQP1//CAV1//KDR//TEK//TGFBR3//FGF10//TGFBR2//FGF2//CNTN1//C3//DMD//ANK2//PLN//CD36//IGF1//PIK3R1//SORBS1//OPHN1//LAMA2//NEXN//PTP4A1//DPYSL3//SFRP1//DLC1//PODN//ANXA1//NNAT//OXCT1//LIPG//CRYAB//MAP1B//TEX15//TRIM9//APOD//FCER1A//PPAP2B//PDE8B//PPP1R12A//NTRK2//KCNMB4//CCL21//GPAM//PTX3//TRPC1//FGF7//LPAR1//CXCL12//CD34//NLGN1 |
| GO:0072521 | purine-containing compound metabolic process | Biological process | 35 | 728 | 428 | 14747 | 1.65651959022286 | 0.00238487935376348 | 0.0401890093219012 | 2.6225335861501 | AMPD1//CALCRL//AQP1//NME5//GNAL//GNG11//RHOJ//PDE8B//PDE7B//ACTC1//MAT2A//NTRK2//CAP2//EDNRB//CACNB4//PTHLH//VIP//AKAP12//BVES//ALDH1A1//DLC1//ERRFI1//CNN3//PLN//FGF10//SFRP1//PREX2//CCL21//SPRY1//ARHGAP6//TPM2//RGS5//RGS22//GNG7//PAPSS2 |
| GO:0048771 | tissue remodeling | Biological process | 10 | 119 | 428 | 14747 | 2.89542919971727 | 0.00243288687749621 | 0.0408478811824343 | 2.61387808409069 | EPAS1//FGF10//FOXC1//IGF1//MEF2C//CST3//SFRP1//CAV1//FRZB//ANXA1 |
| GO:0042246 | tissue regeneration | Biological process | 5 | 33 | 428 | 14747 | 5.22054658736902 | 0.00244110843719727 | 0.0408478811824343 | 2.61241292823115 | IGF1//TGFBR2//APOD//FGF10//GATM |
| GO:0050878 | regulation of body fluid levels | Biological process | 30 | 596 | 428 | 14747 | 1.73434265821991 | 0.00246180340992297 | 0.041050141475254 | 2.60874663109379 | ANO6//AQP1//EDNRB//A2M//ANGPT1//CAV1//CD36//CLU//CFD//F13A1//IGF1//KCNMA1//PDE1A//PIK3R1//PRKAR2B//SELP//TEK//TTN//WEE1//TFPI2//PAPSS2//MMRN1//KCNMB4//PLSCR4//JAM2//VIP//CD34//TRPC1//FGF10//HEG1 |
| GO:0040008 | regulation of growth | Biological process | 27 | 518 | 428 | 14747 | 1.79594865225706 | 0.00247384378625113 | 0.041107181242619 | 2.60662772787225 | IGFBP4//FBLN5//MYOCD//MUSK//SFRP1//TAF9B//PPP1R1C//BDKRB1//CRYAB//FGF2//FRZB//SLIT3//SLIT2//WFDC1//TSPYL2//CD36//CTSG//GHR//IGF1//GPAM//TGFBR2//MAP1B//MT1A//FOXC1//FGFR1//MEF2C//TSPYL5 |
| GO:0043206 | extracellular fibril organization | Biological process | 3 | 10 | 428 | 14747 | 10.3366822429907 | 0.00250198392137752 | 0.0411446942105151 | 2.60171548556165 | CST3//TNXB//MFAP5 |
| GO:0045019 | negative regulation of nitric oxide biosynthetic process | Biological process | 3 | 10 | 428 | 14747 | 10.3366822429907 | 0.00250198392137752 | 0.0411446942105151 | 2.60171548556165 | CAV1//CD34//PTGIS |
| GO:0097435 | fibril organization | Biological process | 3 | 10 | 428 | 14747 | 10.3366822429907 | 0.00250198392137752 | 0.0411446942105151 | 2.60171548556165 | CST3//TNXB//MFAP5 |
| GO:0002040 | sprouting angiogenesis | Biological process | 6 | 48 | 428 | 14747 | 4.30695093457944 | 0.00252372551641308 | 0.0412179691362123 | 2.59795788127793 | FGF2//KDR//SLIT2//ANGPT1//TEK//PARVA |
| GO:0035272 | exocrine system development | Biological process | 6 | 48 | 428 | 14747 | 4.30695093457944 | 0.00252372551641308 | 0.0412179691362123 | 2.59795788127793 | CST3//FGF10//IGF1//FOXC1//FGF7//FGFR1 |
| GO:0061041 | regulation of wound healing | Biological process | 8 | 82 | 428 | 14747 | 3.36152268064737 | 0.00257508430393558 | 0.0419132322370948 | 2.58920854831805 | ANO6//SELP//CAV1//CD36//CD34//TGFBR2//PPAP2B//FGF2 |
| GO:0043410 | positive regulation of MAPK cascade | Biological process | 19 | 320 | 428 | 14747 | 2.04580169392523 | 0.00260807465069781 | 0.0420148569355687 | 2.58367998198838 | LPAR1//FGF2//FGF10//GHR//KIT//FCER1A//FGFR1//FZD4//CCL21//EDA2R//ANGPT1//CD44//KDR//TEK//CD36//IGF1//IGFBP4//NTRK2//CDON |
| GO:0035556 | intracellular signal transduction | Biological process | 77 | 1947 | 428 | 14747 | 1.36265114314378 | 0.00262998901805264 | 0.0420148569355687 | 2.58004606497378 | CAV1//FGFR1//MEF2C//MAPK4//SCG2//DOK5//FGF2//LPAR1//FGF10//GHR//KIT//RCAN1//RCAN2//TGFBR3//FCER1A//CD36//A2M//ARHGAP6//GEM//OPHN1//ITSN1//DIRAS3//DLC1//IQGAP2//RAB9B//RHOJ//RERGL//ARHGAP28//RABL3//IGF1//KSR1//CTNNAL1//AKAP12//TEK//NLGN1//ANK2//CASQ2//DMD//PLN//PIK3R1//ANGPT1//KDR//NTRK2//SELP//EDNRB//PIK3C2A//ARHGEF26//TEAD1//IL6ST//CCL21//SLC44A2//SPRY1//IGFBP4//CDON//TNXB//FZD4//SFRP1//HIPK3//CD44//EDA2R//SLIT2//CRYAB//TSPYL5//PRNP//CLU//VDAC2//BMX//PLCB4//PRKAR2B//DCLK1//TENC1//PREX2//ASB5//DCDC1 |
| GO:0051056 | regulation of small GTPase mediated signal transduction | Biological process | 21 | 369 | 428 | 14747 | 1.96088823037763 | 0.00264631297285433 | 0.0420148569355687 | 2.57735879418264 | NTRK2//BVES//ALDH1A1//DLC1//ERRFI1//SFRP1//PREX2//CCL21//SPRY1//ITSN1//ARHGEF26//LPAR1//FGF10//IGF1//CDON//SLIT2//A2M//ARHGAP6//OPHN1//RHOJ//ARHGAP28 |
| GO:0009725 | response to hormone stimulus | Biological process | 32 | 652 | 428 | 14747 | 1.69107275958947 | 0.00264709127690151 | 0.0420148569355687 | 2.57723108311452 | FGF2//FGF7//FGF10//FGFR1//PDK4//PIK3C2A//PIK3R1//SORBS1//CAB39L//GHR//TGFBR3//ANXA1//CRYAB//CST3//WFDC1//CAV1//SLIT3//SLIT2//CCL21//GATM//RCAN1//TGFBR2//PTGDS//MEF2C//GNG7//GNG11//PRKAR2B//SFRP1//AQP1//KIT//OXCT1//SNRPN |
| GO:1901566 | organonitrogen compound biosynthetic process | Biological process | 32 | 652 | 428 | 14747 | 1.69107275958947 | 0.00264709127690151 | 0.0420148569355687 | 2.57723108311452 | GPC5//GPC3//GPC6//CALCRL//AQP1//NME5//MAT2A//GATM//PEMT//SLC44A2//UGCG//GNAL//NTRK2//CAP2//EDNRB//VIP//FMOD//OMD//OGN//ADI1//PPAP2A//PPAP2B//UST//ANGPT1//PTHLH//AKAP12//AMPD1//NR4A2//DMD//GNG7//PAPSS2//IGF1 |
| GO:0006024 | glycosaminoglycan biosynthetic process | Biological process | 9 | 101 | 428 | 14747 | 3.07030165633386 | 0.00265832600848842 | 0.0420148569355687 | 2.57539175965907 | FMOD//OMD//OGN//UST//ANGPT1//IGF1//GPC5//GPC3//GPC6 |
| GO:0030808 | regulation of nucleotide biosynthetic process | Biological process | 9 | 101 | 428 | 14747 | 3.07030165633386 | 0.00265832600848842 | 0.0420148569355687 | 2.57539175965907 | GNAL//NTRK2//CAP2//EDNRB//VIP//PTHLH//AKAP12//CALCRL//GNG7 |
| GO:1900371 | regulation of purine nucleotide biosynthetic process | Biological process | 9 | 101 | 428 | 14747 | 3.07030165633386 | 0.00265832600848842 | 0.0420148569355687 | 2.57539175965907 | GNAL//NTRK2//CAP2//EDNRB//VIP//PTHLH//AKAP12//CALCRL//GNG7 |
| GO:0033121 | regulation of purine nucleotide catabolic process | Biological process | 16 | 250 | 428 | 14747 | 2.20515887850467 | 0.00266061790617357 | 0.0420148569355687 | 2.57501749040739 | NTRK2//BVES//ALDH1A1//DLC1//ERRFI1//CNN3//PLN//FGF10//SFRP1//PREX2//CCL21//SPRY1//ARHGAP6//TPM2//RGS5//RGS22 |
| GO:0048589 | developmental growth | Biological process | 17 | 274 | 428 | 14747 | 2.13775666825841 | 0.00274389844886804 | 0.0431869693156821 | 2.56163196581964 | MUSK//IGF1//APOD//FGF10//GATM//TGFBR2//MAP1B//DCLK1//SLIT3//SLIT2//FGF2//FGFR1//MEF2C//RDH10//PTHLH//MYOCD//SFRP1 |
| GO:0030811 | regulation of nucleotide catabolic process | Biological process | 16 | 251 | 428 | 14747 | 2.19637338496481 | 0.00276826685132959 | 0.0434162714967874 | 2.55779204771457 | NTRK2//BVES//ALDH1A1//DLC1//ERRFI1//CNN3//PLN//FGF10//SFRP1//PREX2//CCL21//SPRY1//ARHGAP6//TPM2//RGS5//RGS22 |
| GO:0010092 | specification of organ identity | Biological process | 5 | 34 | 428 | 14747 | 5.06700109950522 | 0.00279488264825199 | 0.0434162714967874 | 2.55363642258491 | FGF2//FGF10//FGFR1//SPRY1//MEF2C |
| GO:0048009 | insulin-like growth factor receptor signaling pathway | Biological process | 5 | 34 | 428 | 14747 | 5.06700109950522 | 0.00279488264825199 | 0.0434162714967874 | 2.55363642258491 | IGF1//IGFBP4//CILP//GHR//PIK3R1 |
| GO:1901888 | regulation of cell junction assembly | Biological process | 5 | 34 | 428 | 14747 | 5.06700109950522 | 0.00279488264825199 | 0.0434162714967874 | 2.55363642258491 | KDR//SFRP1//TEK//APOD//ARHGAP6 |
| GO:0014808 | release of sequestered calcium ion into cytosol by sarcoplasmic reticulum | Biological process | 4 | 21 | 428 | 14747 | 6.56297285269248 | 0.00282858979971535 | 0.043514660499492 | 2.54843002908486 | CASQ2//DMD//ANK2//PLN |
| GO:0021952 | central nervous system projection neuron axonogenesis | Biological process | 4 | 21 | 428 | 14747 | 6.56297285269248 | 0.00282858979971535 | 0.043514660499492 | 2.54843002908486 | NFIB//SLIT2//NR4A2//DCLK1 |
| GO:0048488 | synaptic vesicle endocytosis | Biological process | 4 | 21 | 428 | 14747 | 6.56297285269248 | 0.00282858979971535 | 0.043514660499492 | 2.54843002908486 | ZDHHC15//NLGN1//OPHN1//ITSN1 |
| GO:0006023 | aminoglycan biosynthetic process | Biological process | 9 | 102 | 428 | 14747 | 3.04020065970313 | 0.00284278261887012 | 0.0435923804160502 | 2.54625634855249 | GPC5//GPC3//GPC6//FMOD//OMD//OGN//UST//ANGPT1//IGF1 |
| GO:0051960 | regulation of nervous system development | Biological process | 24 | 447 | 428 | 14747 | 1.84996550210124 | 0.00290237416335235 | 0.0442899519320487 | 2.5372466006493 | MUSK//CNTN1//DMD//DPYSL3//FGFR1//NTRK2//LPAR1//PMP22//EDNRB//FGF2//IGF1//WASF3//NLGN1//PBX1//MEF2C//ZEB1//CDON//MAP1B//IL6ST//ASPA//SERPINF1//SLIT2//PTPRD//SFRP1 |
| GO:0048699 | generation of neurons | Biological process | 47 | 1071 | 428 | 14747 | 1.51205747096346 | 0.00290684733796 | 0.0442899519320487 | 2.53657777594286 | FGFR1//MYH10//NTRK2//NR4A2//NR2F1//DCLK1//ANK2//CACNB4//CNTN1//DPYSL3//GFRA1//ITGA9//LAMA2//MYH11//OPHN1//PRNP//SLIT3//TRPC1//SLIT2//MYL9//CAP2//BOC//CXCL12//DMD//LPAR1//PMP22//APOD//EDNRB//MAP1B//FAIM2//NFIB//MEF2C//NNAT//PTPRD//FZD4//PRICKLE2//RORB//KCNMA1//CLN5//NLGN1//PBX1//ZEB1//CDON//IL6ST//ASPA//WEE1//SERPINF1 |
| GO:0007186 | G-protein coupled receptor signaling pathway | Biological process | 30 | 603 | 428 | 14747 | 1.7142093271958 | 0.00292403454993211 | 0.0444099387535867 | 2.5340175001286 | CHRM2//CALCRL//GLP2R//GNAL//PTHLH//EDNRB//BDKRB1//PPAP2A//CACNB4//BAI3//CPE//GPR64//LPHN3//ELTD1//GNG7//RGS5//KCTD12//VIP//RGS22//C3//TMOD2//LPAR1//PLN//GNG11//PTGER3//PTGFR//CXCL12//AKAP12//CCRL1//PREX2 |
| GO:0097285 | cell-type specific apoptotic process | Biological process | 17 | 276 | 428 | 14747 | 2.12226567790871 | 0.00295714944987775 | 0.044770303893546 | 2.52912672633046 | MYOCD//ANXA1//IGF1//FAIM2//CNTFR//MEF2C//NTRK2//NR4A2//ITSN1//MUSK//GPAM//CCL21//CXCL12//SFRP1//TEK//ANGPT1//KDR |
| GO:0009118 | regulation of nucleoside metabolic process | Biological process | 16 | 253 | 428 | 14747 | 2.17901074951055 | 0.00299441933541389 | 0.0451910943373065 | 2.5236873815801 | NTRK2//BVES//ALDH1A1//DLC1//ERRFI1//CNN3//PLN//FGF10//SFRP1//PREX2//CCL21//SPRY1//ARHGAP6//TPM2//RGS5//RGS22 |
| GO:0048762 | mesenchymal cell differentiation | Biological process | 11 | 143 | 428 | 14747 | 2.65043134435658 | 0.00302672094159451 | 0.0454117901705873 | 2.5190276183934 | EDNRB//TGFBR3//SFRP1//FOXC1//RDH10//FRZB//MEF2C//FGF10//TCF21//OSR1//FGFR1 |
| GO:0006629 | lipid metabolic process | Biological process | 50 | 1159 | 428 | 14747 | 1.48643690580826 | 0.00302808749722096 | 0.0454117901705873 | 2.51883157996086 | ABCD2//PTGDS//PTGIS//RDH10//DPM1//C3//GHR//PRKAR2B//TNXB//CROT//GPAM//CH25H//PTPLA//C5ORF4//SCD5//ECH1//CAV1//PIK3C2A//PIK3R1//PLA2G2A//UGCG//PPAP2A//PPAP2B//PEMT//SLC44A2//FGF2//ST6GALNAC3//KIT//PBX1//TIPARP//SERPINA6//ECI2//GLT25D2//LIPG//PDK4//PLCB4//PLCXD3//SORBS1//FCER1A//FABP4//ANXA1//KCNMA1//ALDH1A1//CCL21//TEK//CD36//OXCT1//CAB39L//APOD//CLU |
| GO:0030804 | positive regulation of cyclic nucleotide biosynthetic process | Biological process | 7 | 67 | 428 | 14747 | 3.59983958711117 | 0.00320507563954068 | 0.0479153784481803 | 2.49416171670757 | GNAL//NTRK2//CAP2//VIP//PTHLH//AKAP12//CALCRL |
| GO:0010941 | regulation of cell death | Biological process | 53 | 1250 | 428 | 14747 | 1.46091775700935 | 0.00326591841561074 | 0.0486723903876488 | 2.48599466837235 | CLU//PLAGL1//DLC1//ANGPT1//ANXA1//AQP1//CD44//CRYAB//EDNRB//FGF10//FGFR1//FOXC1//IGF1//IL6ST//KDR//MEF2C//PIK3R1//PRNP//PTGFR//CXCL12//SFRP1//TEK//VIP//SCG2//NME5//HIPK3//FAIM2//TAF9B//OSR1//MYOCD//ZNF738//LPAR1//FRZB//KCNMA1//PTGIS//SRPX//SLIT2//CST3//KIT//KANK2//CNTFR//NTRK2//NR4A2//ITSN1//MUSK//FGF2//CD34//GPAM//CAV1//GHR//CCL21//PDK4//VDAC2 |
| GO:0060284 | regulation of cell development | Biological process | 26 | 503 | 428 | 14747 | 1.78100555545233 | 0.00330230456550316 | 0.0490613410370236 | 2.48118287504123 | MUSK//SFRP1//TGFBR3//FRZB//MYOCD//CNTN1//DMD//DPYSL3//FGFR1//NTRK2//LPAR1//PMP22//EDNRB//MEF2C//BOC//NLGN1//PBX1//ZEB1//CDON//MAP1B//FGF2//IL6ST//ASPA//SERPINF1//SLIT2//PTPRD |
| GO:0022604 | regulation of cell morphogenesis | Biological process | 18 | 303 | 428 | 14747 | 2.04686777088924 | 0.00332482594015708 | 0.049242530772078 | 2.47823108576177 | KDR//KIT//MYH10//DLC1//WASF3//FERMT2//BVES//PALMD//PARVA//RHOJ//SFRP1//TGFBR3//MAP1B//NTRK2//SLIT2//PTPRD//ZRANB1//SH3D19 |
| GO:0048875 | chemical homeostasis within a tissue | Biological process | 3 | 11 | 428 | 14747 | 9.39698385726423 | 0.00336660439923402 | 0.0492786795409718 | 2.47280791340805 | EPAS1//KDR//PTHLH |
| GO:0051894 | positive regulation of focal adhesion assembly | Biological process | 3 | 11 | 428 | 14747 | 9.39698385726423 | 0.00336660439923402 | 0.0492786795409718 | 2.47280791340805 | KDR//SFRP1//TEK |
| GO:1901890 | positive regulation of cell junction assembly | Biological process | 3 | 11 | 428 | 14747 | 9.39698385726423 | 0.00336660439923402 | 0.0492786795409718 | 2.47280791340805 | KDR//SFRP1//TEK |
| GO:0002675 | positive regulation of acute inflammatory response | Biological process | 4 | 22 | 428 | 14747 | 6.26465590484282 | 0.00337893231492929 | 0.0492786795409718 | 2.47122050770635 | C3//FCER1A//PTGER3//IL6ST |
| GO:0070296 | sarcoplasmic reticulum calcium ion transport | Biological process | 4 | 22 | 428 | 14747 | 6.26465590484282 | 0.00337893231492929 | 0.0492786795409718 | 2.47122050770635 | CASQ2//DMD//ANK2//PLN |
| GO:0060443 | mammary gland morphogenesis | Biological process | 6 | 51 | 428 | 14747 | 4.05360087960418 | 0.00344359899337202 | 0.0500686695103389 | 2.46298742780651 | CAV1//FRZB//FGF10//PTHLH//SLIT2//TGFBR2 |
| GO:0030799 | regulation of cyclic nucleotide metabolic process | Biological process | 9 | 105 | 428 | 14747 | 2.95333778371162 | 0.00345746748406252 | 0.0501175149893439 | 2.46124189554508 | GNAL//NTRK2//CAP2//EDNRB//VIP//PTHLH//AKAP12//CALCRL//GNG7 |
| GO:0051592 | response to calcium ion | Biological process | 8 | 86 | 428 | 14747 | 3.20517278852423 | 0.00346850779057006 | 0.0501251928885716 | 2.45985732570019 | KCNMB4//KCNIP1//FOSB//MEF2C//CAV1//KCNMA1//TRPC1//TTN |
| GO:0043627 | response to estrogen stimulus | Biological process | 10 | 125 | 428 | 14747 | 2.75644859813084 | 0.00348064513189919 | 0.0501486303142817 | 2.45834025281862 | ANXA1//CRYAB//CST3//FGF10//GHR//WFDC1//SFRP1//CAV1//RCAN1//TGFBR2 |
| GO:0030810 | positive regulation of nucleotide biosynthetic process | Biological process | 7 | 69 | 428 | 14747 | 3.49549641067317 | 0.00378479559701676 | 0.0540409886292603 | 2.42195757018894 | GNAL//NTRK2//CAP2//VIP//PTHLH//AKAP12//CALCRL |
| GO:0060021 | palate development | Biological process | 7 | 69 | 428 | 14747 | 3.49549641067317 | 0.00378479559701676 | 0.0540409886292603 | 2.42195757018894 | MEOX2//TCF21//TGFBR2//TGFBR3//TIPARP//ARID5B//OSR1 |
| GO:1900373 | positive regulation of purine nucleotide biosynthetic process | Biological process | 7 | 69 | 428 | 14747 | 3.49549641067317 | 0.00378479559701676 | 0.0540409886292603 | 2.42195757018894 | GNAL//NTRK2//CAP2//VIP//PTHLH//AKAP12//CALCRL |
| GO:0071310 | cellular response to organic substance | Biological process | 56 | 1347 | 428 | 14747 | 1.43245287207987 | 0.00380946156328785 | 0.0542308125233426 | 2.41913640401411 | CAV1//TSPYL2//CCNC//SMAD9//TGFBR2//TGFBR3//FMOD//FGF2//FGF7//FGF10//FGFR1//PDK4//PIK3C2A//PIK3R1//SORBS1//CAB39L//PDE1A//PRKAR2B//GHR//COLEC12//CD44//IL6ST//KIT//LIFR//VCAM1//PDGFD//ITGA8//MYOCD//FZD4//SLIT3//SLIT2//EDA2R//KDR//MEF2C//SPRY1//SFRP1//CD36//DARC//CXCL12//CCRL1//CNTFR//EDNRB//PLSCR4//AQP1//OSR1//CASQ2//CALCRL//CMA1//OXCT1//DPYSL3//PTGIS//GNG7//GNG11//ANXA1//DMD//TIMP3 |
| GO:0044281 | small molecule metabolic process | Biological process | 96 | 2561 | 428 | 14747 | 1.29158075664077 | 0.00384102515239128 | 0.054517407594506 | 2.41555284891163 | ABCD2//MAT2A//ADI1//PTGDS//PTGIS//RDH10//ALDH1A1//FMO1//PDK4//GHR//GSTA2//GSTM5//AMPD1//CALCRL//AQP1//NME5//GNAL//GNG11//RHOJ//PDE8B//PDE7B//ACTC1//DPYSL3//ASPA//CD34//GATM//C3//PRKAR2B//TNXB//CROT//GPAM//CH25H//PTPLA//C5ORF4//SCD5//ECH1//PEMT//SLC44A2//CYB5A//NTRK2//CAP2//EDNRB//CACNB4//PTHLH//ECI2//IGF1//VIP//FMOD//OMD//OGN//CAV1//DPM1//FCER1A//BCHE//MAMDC2//GPC5//GPC3//GPC6//UST//ANGPT1//AKAP12//ANXA1//BVES//DLC1//ERRFI1//KCNMA1//CNN3//PLN//FGF10//SFRP1//PREX2//CCL21//FGF2//SPRY1//PLA2G2A//NR4A2//OXCT1//GCNT4//EPAS1//ARHGAP6//TPM2//RGS5//RGS22//GNG7//CAB39L//CMAHP//PAPSS2//CD36//CD44//FABP4//PIK3C2A//PIK3R1//UGCG//PPAP2A//PPAP2B//LYVE1 |
| GO:0048520 | positive regulation of behavior | Biological process | 8 | 88 | 428 | 14747 | 3.13232795242141 | 0.00399689404748552 | 0.05624074672015 | 2.39827736417479 | CCL21//KDR//SLIT2//FGF10//CXCL12//SCG2//LPAR1//MEF2C |
| GO:0033144 | negative regulation of intracellular steroid hormone receptor signaling pathway | Biological process | 4 | 23 | 428 | 14747 | 5.992279561154 | 0.00399782200422119 | 0.05624074672015 | 2.39817654596212 | KANK2//IGF1//SFRP1//TCF21 |
| GO:0050927 | positive regulation of positive chemotaxis | Biological process | 4 | 23 | 428 | 14747 | 5.992279561154 | 0.00399782200422119 | 0.05624074672015 | 2.39817654596212 | FGF10//CXCL12//SCG2//KDR |
| GO:0032787 | monocarboxylic acid metabolic process | Biological process | 23 | 433 | 428 | 14747 | 1.83020547797371 | 0.00401094119173057 | 0.0562593486569503 | 2.39675370558109 | ABCD2//PTGDS//PTGIS//RDH10//PDK4//C3//GHR//PRKAR2B//TNXB//CROT//GPAM//CH25H//PTPLA//C5ORF4//SCD5//ECH1//ECI2//IGF1//CAV1//FCER1A//UST//ANXA1//CAB39L |
| GO:1901342 | regulation of vasculature development | Biological process | 12 | 170 | 428 | 14747 | 2.43216052776251 | 0.00404386718072167 | 0.0565548462899168 | 2.39320311708374 | BAI3//SERPINF1//TEK//FGF2//SFRP1//AQP1//C3//CD34//CMA1//KDR//PTGIS//MYOCD |
| GO:0030801 | positive regulation of cyclic nucleotide metabolic process | Biological process | 7 | 70 | 428 | 14747 | 3.44556074766355 | 0.00410290788234409 | 0.0570459699443119 | 2.38690823361528 | GNAL//NTRK2//CAP2//VIP//PTHLH//AKAP12//CALCRL |
| GO:0032355 | response to estradiol stimulus | Biological process | 7 | 70 | 428 | 14747 | 3.44556074766355 | 0.00410290788234409 | 0.0570459699443119 | 2.38690823361528 | SFRP1//ANXA1//CRYAB//CST3//FGF10//GHR//WFDC1 |
| GO:0045766 | positive regulation of angiogenesis | Biological process | 8 | 89 | 428 | 14747 | 3.09713325632679 | 0.00428333418388216 | 0.0593814555899245 | 2.36821804081376 | AQP1//C3//CD34//CMA1//FGF2//KDR//PTGIS//TEK |
| GO:0007411 | axon guidance | Biological process | 20 | 360 | 428 | 14747 | 1.91420041536864 | 0.00433271515375349 | 0.0598919378789866 | 2.36323986190479 | CXCL12//SLIT2//SLIT3//NFIB//ANK2//CACNB4//CNTN1//DPYSL3//FGFR1//GFRA1//ITGA9//LAMA2//MYH10//MYH11//OPHN1//PRNP//TRPC1//MYL9//CAP2//BOC |
| GO:0043408 | regulation of MAPK cascade | Biological process | 24 | 462 | 428 | 14747 | 1.78990168709795 | 0.00439280034015924 | 0.0602001951502863 | 2.35725853566881 | FGF2//LPAR1//FGF10//GHR//KIT//CAV1//FCER1A//FGFR1//SPRY1//CD36//IGF1//IGFBP4//KDR//NTRK2//CDON//TNXB//FZD4//SFRP1//HIPK3//CCL21//EDA2R//ANGPT1//CD44//TEK |
| GO:0042340 | keratan sulfate catabolic process | Biological process | 3 | 12 | 428 | 14747 | 8.61390186915888 | 0.00439288486313685 | 0.0602001951502863 | 2.35725017937971 | FMOD//OMD//OGN |
| GO:0085029 | extracellular matrix assembly | Biological process | 3 | 12 | 428 | 14747 | 8.61390186915888 | 0.00439288486313685 | 0.0602001951502863 | 2.35725017937971 | MYH11//TNXB//FBLN5 |
| GO:0048729 | tissue morphogenesis | Biological process | 25 | 489 | 428 | 14747 | 1.76153412457237 | 0.00451616841208614 | 0.061712341424753 | 2.34522987120324 | CXCL12//TGFBR2//CD44//FGF2//PBX1//TCF21//SFRP1//DLC1//MEF2C//FGF7//FGF10//ERRFI1//FOXC1//FGFR1//IGF1//KDR//SLIT2//ACTC1//TTN//TGFBR3//FRZB//RDH10//PTHLH//OSR1//GCNT4 |
| GO:0030239 | myofibril assembly | Biological process | 5 | 38 | 428 | 14747 | 4.5336325627152 | 0.00458601010329564 | 0.0623928329619702 | 2.33856499282442 | ACTC1//TTN//MYH11//MYH10//TMOD1 |
| GO:0045761 | regulation of adenylate cyclase activity | Biological process | 6 | 54 | 428 | 14747 | 3.82840083073728 | 0.00459213343880301 | 0.0623928329619702 | 2.33798550064192 | GNAL//NTRK2//CAP2//EDNRB//VIP//GNG7 |
| GO:0007264 | small GTPase mediated signal transduction | Biological process | 30 | 623 | 428 | 14747 | 1.65917853017506 | 0.00467287391693862 | 0.063169715896916 | 2.33041593691203 | FGF2//IGF1//KSR1//ARHGAP6//CTNNAL1//RHOJ//ITSN1//ARHGEF26//DLC1//LPAR1//FGF10//SPRY1//A2M//OPHN1//ARHGAP28//CDON//SLIT2//GEM//DIRAS3//IQGAP2//RAB9B//RERGL//RABL3 |
| GO:0050926 | regulation of positive chemotaxis | Biological process | 4 | 24 | 428 | 14747 | 5.74260124610592 | 0.00468904999528376 | 0.063169715896916 | 2.3289151367301 | KDR//FGF10//CXCL12//SCG2 |
| GO:0060343 | trabecula formation | Biological process | 4 | 24 | 428 | 14747 | 5.74260124610592 | 0.00468904999528376 | 0.063169715896916 | 2.3289151367301 | SFRP1//TEK//TGFBR3//ADAMTS1 |
| GO:0002526 | acute inflammatory response | Biological process | 9 | 110 | 428 | 14747 | 2.81909515717927 | 0.00470914435050617 | 0.0632617166410251 | 2.32705799682263 | C3//FCER1A//A2M//ANO6//ANXA1//IL6ST//CFH//PTGER3//VCAM1 |
| GO:1900544 | positive regulation of purine nucleotide metabolic process | Biological process | 7 | 72 | 428 | 14747 | 3.34985072689512 | 0.00479942419854211 | 0.0642934101203576 | 2.31881086312356 | GNAL//NTRK2//CAP2//VIP//PTHLH//AKAP12//CALCRL |
| GO:0030182 | neuron differentiation | Biological process | 43 | 986 | 428 | 14747 | 1.50262791226707 | 0.00486487811481378 | 0.0649876855169381 | 2.31292803612058 | ANK2//CACNB4//CNTN1//DPYSL3//FGFR1//GFRA1//ITGA9//LAMA2//MYH10//MYH11//OPHN1//PRNP//SLIT3//TRPC1//SLIT2//MYL9//CAP2//BOC//CXCL12//DMD//NTRK2//LPAR1//PMP22//APOD//EDNRB//MAP1B//FAIM2//NR4A2//DCLK1//NFIB//PRICKLE2//RORB//KCNMA1//CLN5//NLGN1//PBX1//MEF2C//ZEB1//CDON//WEE1//PTPRD//NNAT//FZD4 |
| GO:0043067 | regulation of programmed cell death | Biological process | 51 | 1217 | 428 | 14747 | 1.44390795506032 | 0.00490740648958533 | 0.0653726858905934 | 2.30914796711135 | CLU//PLAGL1//DLC1//ANGPT1//ANXA1//AQP1//CD44//CRYAB//EDNRB//FGF10//FGFR1//FOXC1//IGF1//IL6ST//KDR//MEF2C//PIK3R1//PRNP//PTGFR//CXCL12//SFRP1//TEK//VIP//SCG2//NME5//HIPK3//FAIM2//TAF9B//OSR1//MYOCD//ZNF738//LPAR1//FRZB//KCNMA1//PTGIS//SRPX//SLIT2//KIT//KANK2//CNTFR//NTRK2//NR4A2//ITSN1//MUSK//FGF2//GPAM//CAV1//CCL21//PDK4//VDAC2//CST3 |
| GO:0001667 | ameboidal cell migration | Biological process | 9 | 111 | 428 | 14747 | 2.79369790351099 | 0.0049970956199196 | 0.0663820306724138 | 2.30128234020998 | EDNRB//FGF10//TGFBR3//TGFBR2//TNS1//ARID5B//FGF2//KIT//CXCL12 |
| GO:0010518 | positive regulation of phospholipase activity | Biological process | 7 | 73 | 428 | 14747 | 3.30396236077327 | 0.00517936034734305 | 0.0684220761675319 | 2.28572387245025 | ARHGAP6//LPAR1//PDE1A//PRKAR2B//FGF2//FGFR1//KIT |
| GO:0045981 | positive regulation of nucleotide metabolic process | Biological process | 7 | 73 | 428 | 14747 | 3.30396236077327 | 0.00517936034734305 | 0.0684220761675319 | 2.28572387245025 | GNAL//NTRK2//CAP2//VIP//PTHLH//AKAP12//CALCRL |
| GO:0048878 | chemical homeostasis | Biological process | 39 | 877 | 428 | 14747 | 1.53223339975916 | 0.00525727157608851 | 0.0692594700175859 | 2.27923958795204 | KCNMB4//CORIN//EDNRB//AQP1//CASQ2//ANK2//CACNB4//CAV1//CXCL12//PRNP//CYBRD1//PDK4//SLC9A9//CLN5//BDKRB1//LPAR1//DMD//PLN//SLC22A17//KCNMA1//ASPA//IGF1//WASF3//PMP22//MPDZ//FABP4//LIPG//EPAS1//KDR//PTHLH//KCNIP1//FGF2//CCL21//TRPC1//GPAM//CMA1//OXCT1//VCAM1//NLGN1 |
| GO:0007423 | sensory organ development | Biological process | 23 | 443 | 428 | 14747 | 1.78889158456573 | 0.00527334758303745 | 0.0692798749958832 | 2.27791360200058 | CST3//FOXC1//CHRDL1//CRYAB//TGFBR2//CDON//FGF10//RDH10//RORB//FGFR1//ITGA8//SOBP//EYA4//OSR1//KCNMA1//FOXP2//NTRK2//FGF2//IGF1//LPPR4//MYH10//FZD4//FRZB |
| GO:0021700 | developmental maturation | Biological process | 12 | 176 | 428 | 14747 | 2.34924596431606 | 0.00532716970732404 | 0.069794704214913 | 2.27350346770168 | CDH5//RECK//EDNRB//ZDHHC15//IGF1//CLN5//NR4A2//EPAS1//FGFR1//KCNMA1//KDR//CCL21 |
| GO:0045595 | regulation of cell differentiation | Biological process | 45 | 1049 | 428 | 14747 | 1.47807658384042 | 0.00538028130072689 | 0.0702974288305933 | 2.26919501724073 | MUSK//SFRP1//TGFBR3//FRZB//CD36//PLA2G2A//MYOCD//CNTN1//DMD//DPYSL3//FGFR1//NTRK2//LPAR1//PMP22//EDNRB//IGF1//KIT//CAV1//OSR1//EFEMP1//PTHLH//FGF10//LDB2//FGF2//GHR//SMAD9//APOLD1//ERRFI1//ADAMTS9//MEF2C//BOC//NLGN1//PBX1//ZEB1//CDON//IL6ST//PIK3R1//MAP1B//ASPA//SERPINF1//SLIT2//PTPRD//TGFBR2//RCAN1//CD34 |
| GO:0010740 | positive regulation of intracellular protein kinase cascade | Biological process | 25 | 496 | 428 | 14747 | 1.73667376394332 | 0.00539751752090164 | 0.070329948243661 | 2.26780593922652 | LPAR1//FGF2//FGF10//GHR//KIT//FCER1A//AKAP12//IL6ST//IGF1//CD36//CCL21//SLC44A2//FGFR1//IGFBP4//KDR//NTRK2//CDON//FZD4//EDA2R//ANGPT1//ITSN1//TEK//TSPYL5//CD44//NLGN1 |
| GO:0001759 | organ induction | Biological process | 4 | 25 | 428 | 14747 | 5.51289719626168 | 0.00545627476851155 | 0.0703873721963989 | 2.2631037674858 | FGF2//FGF10//FGFR1//SPRY1 |
| GO:0035162 | embryonic hemopoiesis | Biological process | 4 | 25 | 428 | 14747 | 5.51289719626168 | 0.00545627476851155 | 0.0703873721963989 | 2.2631037674858 | KDR//KIT//PBX1//TGFBR2 |
| GO:0043552 | positive regulation of phosphatidylinositol 3-kinase activity | Biological process | 4 | 25 | 428 | 14747 | 5.51289719626168 | 0.00545627476851155 | 0.0703873721963989 | 2.2631037674858 | FGF2//KIT//CCL21//TEK |
| GO:1901701 | cellular response to oxygen-containing compound | Biological process | 21 | 393 | 428 | 14747 | 1.84113933081258 | 0.00546096198630061 | 0.0703873721963989 | 2.26273084657329 | COLEC12//SOD3//PDGFD//GPX3//ANXA1//AQP1//CST3//CD36//EDNRB//MEF2C//PLSCR4//FZD4//OSR1//SFRP1//CALCRL//CMA1//OXCT1//VCAM1//PDK4//SLIT2//FBLN5 |
| GO:0014829 | vascular smooth muscle contraction | Biological process | 3 | 13 | 428 | 14747 | 7.95129403306973 | 0.00558890034506012 | 0.0709004564720607 | 2.25267363416229 | EDNRB//ACTA2//PIK3C2A |
| GO:0030214 | hyaluronan catabolic process | Biological process | 3 | 13 | 428 | 14747 | 7.95129403306973 | 0.00558890034506012 | 0.0709004564720607 | 2.25267363416229 | CD44//FGF2//LYVE1 |
| GO:0032878 | regulation of establishment or maintenance of cell polarity | Biological process | 3 | 13 | 428 | 14747 | 7.95129403306973 | 0.00558890034506012 | 0.0709004564720607 | 2.25267363416229 | CDH5//IGF1//TEK |
| GO:0034695 | response to prostaglandin E stimulus | Biological process | 3 | 13 | 428 | 14747 | 7.95129403306973 | 0.00558890034506012 | 0.0709004564720607 | 2.25267363416229 | SFRP1//CCL21//TGFBR3 |
| GO:0060766 | negative regulation of androgen receptor signaling pathway | Biological process | 3 | 13 | 428 | 14747 | 7.95129403306973 | 0.00558890034506012 | 0.0709004564720607 | 2.25267363416229 | IGF1//SFRP1//TCF21 |
| GO:0071453 | cellular response to oxygen levels | Biological process | 8 | 93 | 428 | 14747 | 2.9639232237966 | 0.00558997098626438 | 0.0709004564720607 | 2.25259044623486 | EPAS1//CAV1//AQP1//PTGIS//SFRP1//MYOCD//CD34//LPAR1 |
| GO:0055086 | nucleobase-containing small molecule metabolic process | Biological process | 39 | 881 | 428 | 14747 | 1.52527660793279 | 0.00566254925424363 | 0.0716304970649546 | 2.24698800736265 | AMPD1//CALCRL//AQP1//NME5//GNAL//GNG11//RHOJ//PDE8B//PDE7B//ACTC1//DPYSL3//MAT2A//NTRK2//CAP2//EDNRB//CACNB4//PTHLH//VIP//DPM1//AKAP12//BVES//ALDH1A1//DLC1//ERRFI1//CNN3//PLN//FGF10//SFRP1//PREX2//CCL21//SPRY1//ARHGAP6//TPM2//RGS5//RGS22//GNG7//CMAHP//PAPSS2//FMO1 |
| GO:0045765 | regulation of angiogenesis | Biological process | 11 | 157 | 428 | 14747 | 2.41408714804453 | 0.00612515190835871 | 0.0772773794999013 | 2.21288313600117 | BAI3//SERPINF1//TEK//AQP1//C3//CD34//CMA1//FGF2//KDR//PTGIS//SFRP1 |
| GO:0007266 | Rho protein signal transduction | Biological process | 13 | 202 | 428 | 14747 | 2.21744008513001 | 0.00615936420944178 | 0.077503978667092 | 2.21046411487805 | ITSN1//ARHGEF26//DLC1//LPAR1//ARHGAP6//CTNNAL1//RHOJ |
| GO:0048666 | neuron development | Biological process | 36 | 802 | 428 | 14747 | 1.5466357470809 | 0.0062581181686532 | 0.0779193214601077 | 2.20355624056511 | ANK2//CACNB4//CNTN1//DPYSL3//FGFR1//GFRA1//ITGA9//LAMA2//MYH10//MYH11//OPHN1//PRNP//SLIT3//TRPC1//SLIT2//MYL9//CAP2//BOC//CXCL12//DMD//NTRK2//LPAR1//PMP22//APOD//EDNRB//MAP1B//NR4A2//DCLK1//NFIB//PRICKLE2//RORB//CLN5//WEE1//PTPRD//NLGN1//MEF2C |
| GO:0045597 | positive regulation of cell differentiation | Biological process | 25 | 502 | 428 | 14747 | 1.71591670700376 | 0.00626290041802677 | 0.0779193214601077 | 2.2032244936548 | CD36//PLA2G2A//FGF2//FRZB//SFRP1//ADAMTS9//MEF2C//BOC//DMD//FGFR1//ZEB1//CDON//IGF1//IL6ST//MAP1B//ASPA//SERPINF1//NTRK2//SLIT2//PTPRD//TGFBR2//MYOCD//CD34//GHR//SMAD9 |
| GO:0001960 | negative regulation of cytokine-mediated signaling pathway | Biological process | 4 | 26 | 428 | 14747 | 5.30086268871316 | 0.00630301154831628 | 0.0779193214601077 | 2.20045189716354 | SLIT3//SLIT2//IL6ST//CAV1 |
| GO:0043462 | regulation of ATPase activity | Biological process | 4 | 26 | 428 | 14747 | 5.30086268871316 | 0.00630301154831628 | 0.0779193214601077 | 2.20045189716354 | CNN3//PLN//FGF10//TPM2 |
| GO:0048536 | spleen development | Biological process | 4 | 26 | 428 | 14747 | 5.30086268871316 | 0.00630301154831628 | 0.0779193214601077 | 2.20045189716354 | CACNB4//FGF10//PBX1//TCF21 |
| GO:0090218 | positive regulation of lipid kinase activity | Biological process | 4 | 26 | 428 | 14747 | 5.30086268871316 | 0.00630301154831628 | 0.0779193214601077 | 2.20045189716354 | FGF2//KIT//CCL21//TEK |
| GO:0031346 | positive regulation of cell projection organization | Biological process | 10 | 136 | 428 | 14747 | 2.53350054975261 | 0.00630674315026244 | 0.0779193214601077 | 2.20019485581422 | CNTN1//DMD//DPYSL3//FGFR1//NTRK2//CCL21//MAP1B//SLIT2//PTPRD//NLGN1 |
| GO:0010464 | regulation of mesenchymal cell proliferation | Biological process | 5 | 41 | 428 | 14747 | 4.20190335080921 | 0.00637861101364978 | 0.0781994753832797 | 2.19527388158937 | FGFR1//KDR//TGFBR2//FOXP2//NFIB |
| GO:0055024 | regulation of cardiac muscle tissue development | Biological process | 5 | 41 | 428 | 14747 | 4.20190335080921 | 0.00637861101364978 | 0.0781994753832797 | 2.19527388158937 | TGFBR2//FGF2//FGFR1//MEF2C//MYOCD |
| GO:0061005 | cell differentiation involved in kidney development | Biological process | 5 | 41 | 428 | 14747 | 4.20190335080921 | 0.00637861101364978 | 0.0781994753832797 | 2.19527388158937 | OSR1//ACTA2//MEF2C//TCF21//CD34 |
| GO:0006887 | exocytosis | Biological process | 16 | 274 | 428 | 14747 | 2.01200627600791 | 0.00643120736086069 | 0.0786421228306273 | 2.1917074872722 | A2M//CD36//CLU//CFD//F13A1//IGF1//SELP//TTN//MMRN1//NLGN1//EXOC6//TRIM9//KIT//FCER1A//MYH10//PIK3C2A |
| GO:0009117 | nucleotide metabolic process | Biological process | 37 | 832 | 428 | 14747 | 1.53228062095615 | 0.00649146883681716 | 0.0788772484271609 | 2.18765702345406 | CALCRL//AQP1//NME5//GNAL//GNG11//RHOJ//PDE8B//PDE7B//ACTC1//NTRK2//CAP2//EDNRB//CACNB4//PTHLH//VIP//DPM1//AKAP12//AMPD1//BVES//ALDH1A1//DLC1//ERRFI1//CNN3//PLN//FGF10//SFRP1//PREX2//CCL21//SPRY1//ARHGAP6//TPM2//RGS5//RGS22//GNG7//CMAHP//PAPSS2//FMO1 |
| GO:0031329 | regulation of cellular catabolic process | Biological process | 23 | 451 | 428 | 14747 | 1.75715958306567 | 0.00650660005307764 | 0.0788772484271609 | 2.18664588760757 | ABCD2//NTRK2//BVES//ALDH1A1//DLC1//ERRFI1//CLU//RNF180//CNN3//PLN//FGF10//SFRP1//PREX2//CCL21//SPRY1//ARHGAP6//TPM2//RGS5//RGS22//IGF1//SH3D19//TIMP3//CST3 |
| GO:0031279 | regulation of cyclase activity | Biological process | 6 | 58 | 428 | 14747 | 3.56437318723816 | 0.00653313338828445 | 0.0788772484271609 | 2.18487847464281 | GNAL//NTRK2//CAP2//EDNRB//VIP//GNG7 |
| GO:0035914 | skeletal muscle cell differentiation | Biological process | 6 | 58 | 428 | 14747 | 3.56437318723816 | 0.00653313338828445 | 0.0788772484271609 | 2.18487847464281 | KCNH1//CDON//MYOCD//MEF2C//DMD//IGF1 |
| GO:0050654 | chondroitin sulfate proteoglycan metabolic process | Biological process | 6 | 58 | 428 | 14747 | 3.56437318723816 | 0.00653313338828445 | 0.0788772484271609 | 2.18487847464281 | MAMDC2//GPC5//GPC3//GPC6//UST//IGF1 |
| GO:0051240 | positive regulation of multicellular organismal process | Biological process | 26 | 531 | 428 | 14747 | 1.68709189151134 | 0.00664205910141201 | 0.0796514060661653 | 2.17769726434394 | FGF2//FGF10//FGFR1//SPRY1//ANO6//EDNRB//VIP//MYOCD//AQP1//C3//IL6ST//IGF1//LIPG//CD36//MEF2C//OSR1//PTGER3//WASF3//LAMA2//NLGN1//CD34//GHR//GPAM//CAV1//NTRK2//ANKRD42 |
| GO:0044272 | sulfur compound biosynthetic process | Biological process | 9 | 116 | 428 | 14747 | 2.67327989042862 | 0.0066473599526806 | 0.0796514060661653 | 2.1773508036645 | MAT2A//FMOD//OMD//OGN//ADI1//UST//ANGPT1//PAPSS2//IGF1 |
| GO:0070372 | regulation of ERK1 and ERK2 cascade | Biological process | 9 | 116 | 428 | 14747 | 2.67327989042862 | 0.0066473599526806 | 0.0796514060661653 | 2.1773508036645 | IGF1//SPRY1//ANGPT1//CD44//FGF2//FGF10//KDR//CCL21//TEK |
| GO:0002684 | positive regulation of immune system process | Biological process | 27 | 558 | 428 | 14747 | 1.66720681338559 | 0.00667888740033097 | 0.079828606546813 | 2.17529587826414 | C3//FCER1A//A2M//COLEC12//MEF2C//CCL21//TGFBR2//BDKRB1//SELP//IL6ST//C7//CLU//CFD//CFH//CACNB4//PIK3R1//NFATC2//CAV1//VCAM1//IGF1//GPAM//SH2D1A//FGF10//CTSG//PRNP//CD36//CXCL12 |
| GO:0023056 | positive regulation of signaling | Biological process | 38 | 863 | 428 | 14747 | 1.51716463975915 | 0.00683428645449295 | 0.081418576932041 | 2.16530682199638 | LPAR1//FGF2//FGF10//GHR//KIT//BVES//FCER1A//AKAP12//TEK//NLGN1//ANGPT1//FGFR1//IGF1//KDR//NTRK2//SELP//SFRP1//ZRANB1//TGFBR3//ITGA8//MYOCD//LAMA2//PTGIS//IL6ST//CD36//CCL21//SLC44A2//IGFBP4//CDON//FZD4//C3//TMOD2//EDA2R//ITSN1//TSPYL5//CD44//CAV1//CLU |
| GO:0031399 | regulation of protein modification process | Biological process | 44 | 1035 | 428 | 14747 | 1.46477944828209 | 0.00689798239745905 | 0.081418576932041 | 2.16127791821684 | CCNC//DIRAS3//FGF2//LPAR1//FGF10//GHR//KIT//CAV1//BDKRB1//PRNP//PPAP2B//SLIT2//C3//EDNRB//MUSK//TEK//CDON//ERRFI1//FABP4//TSPYL2//ANGPT1//FCER1A//TSPYL5//TGFBR2//DMD//CD44//PRKAR2B//MYOCD//DLC1//IL6ST//IGF1//FGFR1//SPRY1//TNXB//FZD4//SFRP1//HIPK3//SPDYA//TTN//CCL21//PDGFD//CD36//FGF7//CLU |
| GO:0048511 | rhythmic process | Biological process | 12 | 182 | 428 | 14747 | 2.27179829516278 | 0.00691555321393755 | 0.081418576932041 | 2.16017307251051 | FOXC1//KDR//KIT//ADAMTS1//SFRP1//FZD4//CST3//PTGDS//KCNMA1//ANXA1//SLIT3//SLIT2 |
| GO:0050921 | positive regulation of chemotaxis | Biological process | 7 | 77 | 428 | 14747 | 3.13232795242141 | 0.00692834907153126 | 0.081418576932041 | 2.159370239348 | KDR//SLIT2//FGF10//CXCL12//SCG2//LPAR1//CCL21 |
| GO:0060347 | heart trabecula formation | Biological process | 3 | 14 | 428 | 14747 | 7.38334445927904 | 0.00696160177712771 | 0.081418576932041 | 2.15729082318652 | TEK//TGFBR3//ADAMTS1 |
| GO:0072577 | endothelial cell apoptotic process | Biological process | 3 | 14 | 428 | 14747 | 7.38334445927904 | 0.00696160177712771 | 0.081418576932041 | 2.15729082318652 | TEK//ANGPT1//KDR |
| GO:2000351 | regulation of endothelial cell apoptotic process | Biological process | 3 | 14 | 428 | 14747 | 7.38334445927904 | 0.00696160177712771 | 0.081418576932041 | 2.15729082318652 | ANGPT1//KDR//TEK |
| GO:0048015 | phosphatidylinositol-mediated signaling | Biological process | 10 | 138 | 428 | 14747 | 2.49678315048083 | 0.0069719797576476 | 0.081418576932041 | 2.15664388248498 | PIK3R1//ANGPT1//FGFR1//IGF1//KDR//KIT//NTRK2//SELP//TEK//PIK3C2A |
| GO:0048017 | inositol lipid-mediated signaling | Biological process | 10 | 138 | 428 | 14747 | 2.49678315048083 | 0.0069719797576476 | 0.081418576932041 | 2.15664388248498 | PIK3R1//ANGPT1//FGFR1//IGF1//KDR//KIT//NTRK2//SELP//TEK//PIK3C2A |
| GO:0071495 | cellular response to endogenous stimulus | Biological process | 33 | 724 | 428 | 14747 | 1.57049039603449 | 0.00698263744290308 | 0.081418576932041 | 2.1559805070975 | CCNC//SMAD9//TGFBR2//TGFBR3//FMOD//FGF2//FGF7//FGF10//FGFR1//PDK4//PIK3C2A//PIK3R1//SORBS1//CAB39L//PDE1A//PRKAR2B//GHR//PDGFD//ITGA8//MYOCD//SLIT3//SLIT2//SPRY1//CD44//SFRP1//CD36//CASQ2//AQP1//MEF2C//GNG7//GNG11//ANXA1//KIT |
| GO:0031175 | neuron projection development | Biological process | 32 | 697 | 428 | 14747 | 1.581893026187 | 0.00708028067170162 | 0.0823257439324768 | 2.14994952596063 | ANK2//CACNB4//CNTN1//DPYSL3//FGFR1//GFRA1//ITGA9//LAMA2//MYH10//MYH11//OPHN1//PRNP//SLIT3//TRPC1//SLIT2//MYL9//CAP2//BOC//CXCL12//DMD//NTRK2//LPAR1//PMP22//APOD//MAP1B//NR4A2//DCLK1//NFIB//WEE1//PTPRD//NLGN1//PRICKLE2 |
| GO:0006082 | organic acid metabolic process | Biological process | 43 | 1008 | 428 | 14747 | 1.46983246180092 | 0.00711065306271885 | 0.0823257439324768 | 2.14809051059085 | ABCD2//MAT2A//ADI1//PTGDS//PTGIS//RDH10//PDK4//GHR//GSTA2//GSTM5//ASPA//CD34//GATM//C3//PRKAR2B//TNXB//CROT//GPAM//CH25H//PTPLA//C5ORF4//SCD5//ECH1//ECI2//IGF1//FMOD//OMD//OGN//CAV1//FCER1A//MAMDC2//CYB5A//GPC5//GPC3//GPC6//UST//ANGPT1//ANXA1//PLA2G2A//GCNT4//CAB39L//PAPSS2//FMO1 |
| GO:0072522 | purine-containing compound biosynthetic process | Biological process | 14 | 229 | 428 | 14747 | 2.10645635228339 | 0.00711222614807726 | 0.0823257439324768 | 2.14799444251864 | CALCRL//AQP1//NME5//MAT2A//GNAL//NTRK2//CAP2//EDNRB//VIP//PTHLH//AKAP12//AMPD1//GNG7//PAPSS2 |
| GO:0048705 | skeletal system morphogenesis | Biological process | 12 | 183 | 428 | 14747 | 2.25938409682856 | 0.00721318942741261 | 0.0831141683453843 | 2.1418726625417 | MGP//MEF2C//PTHLH//OSR1//TGFBR2//RDH10//SFRP1//GHR//FREM1//FGFR1//TIPARP//ARID5B |
| GO:0021955 | central nervous system neuron axonogenesis | Biological process | 4 | 27 | 428 | 14747 | 5.10453444098304 | 0.00723262316278769 | 0.0831141683453843 | 2.14070416210948 | NR4A2//DCLK1//NFIB//SLIT2 |
| GO:0060043 | regulation of cardiac muscle cell proliferation | Biological process | 4 | 27 | 428 | 14747 | 5.10453444098304 | 0.00723262316278769 | 0.0831141683453843 | 2.14070416210948 | TGFBR2//FGF2//FGFR1//MEF2C |
| GO:0048754 | branching morphogenesis of an epithelial tube | Biological process | 11 | 161 | 428 | 14747 | 2.35410982759622 | 0.00736016670961878 | 0.0843765265340672 | 2.13311234867234 | CXCL12//TGFBR2//CD44//FGF2//PBX1//TCF21//RDH10//FGF10//IGF1//KDR//SLIT2 |
| GO:0006979 | response to oxidative stress | Biological process | 15 | 254 | 428 | 14747 | 2.03477996909265 | 0.00741358956733842 | 0.0847851526298248 | 2.12997146114084 | APOD//GPX3//SOD3//CRYAB//EPAS1//ANXA1//AQP1//CST3//CD36//FBLN5//GATM//PRNP//SEPP1//PDLIM1//SCARA3 |
| GO:0042592 | homeostatic process | Biological process | 51 | 1244 | 428 | 14747 | 1.4125691168074 | 0.00745348882299448 | 0.085037531571437 | 2.12764039545195 | KCNMB4//ANXA1//MEF2C//CD34//CORIN//EDNRB//AQP1//CASQ2//ANK2//CACNB4//CAV1//CXCL12//PRNP//CYBRD1//PDK4//SLC9A9//CLN5//BDKRB1//LPAR1//LDB2//DMD//PLN//SLC22A17//KCNMA1//TENC1//ASPA//IGF1//EPAS1//KIT//DYRK3//EXOC6//PTGER3//WASF3//PMP22//MPDZ//FABP4//LIPG//KDR//PTHLH//KCNIP1//GCNT4//TEX15//FGF2//CCL21//TRPC1//GPAM//TGFBR3//CMA1//OXCT1//VCAM1//NLGN1 |
| GO:0009152 | purine ribonucleotide biosynthetic process | Biological process | 13 | 207 | 428 | 14747 | 2.16387873041672 | 0.00751182343974525 | 0.0854985345683654 | 2.12425462841573 | CALCRL//AQP1//NME5//GNAL//NTRK2//CAP2//EDNRB//VIP//PTHLH//AKAP12//AMPD1//GNG7//PAPSS2 |
| GO:0006753 | nucleoside phosphate metabolic process | Biological process | 37 | 841 | 428 | 14747 | 1.51588284974496 | 0.00766370186110304 | 0.0870195099419057 | 2.11556139884031 | CALCRL//AQP1//NME5//GNAL//GNG11//RHOJ//PDE8B//PDE7B//ACTC1//NTRK2//CAP2//EDNRB//CACNB4//PTHLH//VIP//DPM1//AKAP12//AMPD1//BVES//ALDH1A1//DLC1//ERRFI1//CNN3//PLN//FGF10//SFRP1//PREX2//CCL21//SPRY1//ARHGAP6//TPM2//RGS5//RGS22//GNG7//CMAHP//PAPSS2//FMO1 |
| GO:0009719 | response to endogenous stimulus | Biological process | 45 | 1071 | 428 | 14747 | 1.44771459985863 | 0.00774152722248511 | 0.0876944021948491 | 2.11117335470854 | GNAL//NR4A2//CCNC//SMAD9//TGFBR2//TGFBR3//FMOD//FGF2//FGF7//FGF10//FGFR1//PDK4//PIK3C2A//PIK3R1//SORBS1//CAB39L//PDE1A//PRKAR2B//OXCT1//SNRPN//GHR//PDGFD//ITGA8//MYOCD//ANXA1//CRYAB//CST3//WFDC1//CAV1//SLIT3//SLIT2//CCL21//SPRY1//GATM//RCAN1//CD44//SFRP1//PTGDS//CD36//CASQ2//AQP1//MEF2C//GNG7//GNG11//KIT |
| GO:0055080 | cation homeostasis | Biological process | 22 | 432 | 428 | 14747 | 1.75468371408792 | 0.00781223462421262 | 0.0879122422074905 | 2.10722472205716 | CORIN//EDNRB//CASQ2//ANK2//CACNB4//CAV1//CXCL12//PRNP//CYBRD1//PDK4//SLC9A9//CLN5//BDKRB1//LPAR1//DMD//PLN//SLC22A17//KCNMA1//FGF2//CCL21//TRPC1//KDR |
| GO:0010595 | positive regulation of endothelial cell migration | Biological process | 5 | 43 | 428 | 14747 | 4.00646598565529 | 0.00781606011658125 | 0.0879122422074905 | 2.10701210893628 | ANGPT1//FGF2//PPAP2B//KDR//TEK |
| GO:0045445 | myoblast differentiation | Biological process | 5 | 43 | 428 | 14747 | 4.00646598565529 | 0.00781606011658125 | 0.0879122422074905 | 2.10701210893628 | MYOCD//MEF2C//BOC//EPAS1//IGF1 |
| GO:0051336 | regulation of hydrolase activity | Biological process | 34 | 759 | 428 | 14747 | 1.54346594756997 | 0.00802189143531078 | 0.0898775480659993 | 2.09572321986179 | DLC1//TSPYL2//ARHGAP6//LPAR1//PDE1A//PRKAR2B//CST3//FGFR1//MEF2C//FGF2//KIT//SERPINA6//SERPINF1//RECK//NTRK2//BVES//ALDH1A1//ERRFI1//CNN3//PLN//FGF10//SFRP1//PREX2//CCL21//SPRY1//PPP1R12A//AQP1//CD44//CRYAB//TPM2//RGS5//RGS22//RCAN1//CAV1 |
| GO:0009150 | purine ribonucleotide metabolic process | Biological process | 30 | 648 | 428 | 14747 | 1.5951670128072 | 0.00802848301029895 | 0.0898775480659993 | 2.09536650733585 | CALCRL//AQP1//NME5//GNAL//GNG11//RHOJ//PDE8B//PDE7B//ACTC1//NTRK2//CAP2//EDNRB//CACNB4//PTHLH//VIP//AKAP12//AMPD1//BVES//ALDH1A1//DLC1//ERRFI1//SFRP1//PREX2//CCL21//SPRY1//ARHGAP6//RGS5//RGS22//GNG7//PAPSS2 |
| GO:0071363 | cellular response to growth factor stimulus | Biological process | 22 | 434 | 428 | 14747 | 1.746597614023 | 0.00822349155424005 | 0.0918450379910323 | 2.08494374927464 | CCNC//SMAD9//TGFBR2//TGFBR3//FMOD//FGF2//FGF7//FGF10//FGFR1//PDE1A//PIK3R1//PRKAR2B//ITGA8//MYOCD//FZD4//KDR//SPRY1//CD44//SFRP1//MEF2C//DMD//SLIT2 |
| GO:0051057 | positive regulation of small GTPase mediated signal transduction | Biological process | 4 | 28 | 428 | 14747 | 4.92222963951936 | 0.00824831226574582 | 0.0919070121386491 | 2.08363490583279 | LPAR1//FGF10//IGF1//CDON |
| GO:0051339 | regulation of lyase activity | Biological process | 6 | 61 | 428 | 14747 | 3.38907614524284 | 0.00833656681032822 | 0.0923114633970603 | 2.07901276498634 | GNAL//NTRK2//CAP2//EDNRB//VIP//GNG7 |
| GO:0030900 | forebrain development | Biological process | 16 | 282 | 428 | 14747 | 1.95492808378074 | 0.00837927026661254 | 0.0923114633970603 | 2.07679380153475 | AQP1//MYH10//FOXP2//FGF2//PTPRS//SLIT2//FGFR1//NFIB//FGF10//NR4A2//NTRK2//TACC1//CDON//NR2F1//DCLK1//DLC1 |
| GO:0010517 | regulation of phospholipase activity | Biological process | 7 | 80 | 428 | 14747 | 3.01486565420561 | 0.00850259671374576 | 0.0923114633970603 | 2.07044841943555 | ARHGAP6//LPAR1//PDE1A//PRKAR2B//FGFR1//FGF2//KIT |
| GO:0034614 | cellular response to reactive oxygen species | Biological process | 7 | 80 | 428 | 14747 | 3.01486565420561 | 0.00850259671374576 | 0.0923114633970603 | 2.07044841943555 | SOD3//GPX3//ANXA1//AQP1//CST3//CD36//FBLN5 |
| GO:0050680 | negative regulation of epithelial cell proliferation | Biological process | 7 | 80 | 428 | 14747 | 3.01486565420561 | 0.00850259671374576 | 0.0923114633970603 | 2.07044841943555 | CAV1//SCG2//SERPINF1//NFIB//SFRP1//TGFBR3//WFDC1 |
| GO:0019430 | removal of superoxide radicals | Biological process | 3 | 15 | 428 | 14747 | 6.8911214953271 | 0.00851688905319911 | 0.0923114633970603 | 2.06971901013352 | CD36//FBLN5//SOD3 |
| GO:0050930 | induction of positive chemotaxis | Biological process | 3 | 15 | 428 | 14747 | 6.8911214953271 | 0.00851688905319911 | 0.0923114633970603 | 2.06971901013352 | FGF10//CXCL12//SCG2 |
| GO:0055003 | cardiac myofibril assembly | Biological process | 3 | 15 | 428 | 14747 | 6.8911214953271 | 0.00851688905319911 | 0.0923114633970603 | 2.06971901013352 | ACTC1//MYH10//TTN |
| GO:0060442 | branching involved in prostate gland morphogenesis | Biological process | 3 | 15 | 428 | 14747 | 6.8911214953271 | 0.00851688905319911 | 0.0923114633970603 | 2.06971901013352 | IGF1//SFRP1//CD44 |
| GO:0071451 | cellular response to superoxide | Biological process | 3 | 15 | 428 | 14747 | 6.8911214953271 | 0.00851688905319911 | 0.0923114633970603 | 2.06971901013352 | SOD3//CD36//FBLN5 |
| GO:0072202 | cell differentiation involved in metanephros development | Biological process | 3 | 15 | 428 | 14747 | 6.8911214953271 | 0.00851688905319911 | 0.0923114633970603 | 2.06971901013352 | TCF21//OSR1//CD34 |
| GO:2000811 | negative regulation of anoikis | Biological process | 3 | 15 | 428 | 14747 | 6.8911214953271 | 0.00851688905319911 | 0.0923114633970603 | 2.06971901013352 | CAV1//NTRK2//PDK4 |
| GO:0003206 | cardiac chamber morphogenesis | Biological process | 8 | 100 | 428 | 14747 | 2.75644859813084 | 0.00858110514463836 | 0.0927148871625824 | 2.06645677656734 | PARVA//HEG1//MEF2C//CPE//FOXC1//TGFBR3//TEK//ADAMTS1 |
| GO:0009967 | positive regulation of signal transduction | Biological process | 36 | 819 | 428 | 14747 | 1.51453219677519 | 0.00859299226795165 | 0.0927148871625824 | 2.06585557898926 | LPAR1//FGF2//FGF10//GHR//KIT//FCER1A//AKAP12//TEK//NLGN1//ANGPT1//FGFR1//IGF1//KDR//NTRK2//SELP//SFRP1//ZRANB1//TGFBR3//ITGA8//MYOCD//PTGIS//IL6ST//CD36//CCL21//SLC44A2//IGFBP4//CDON//FZD4//C3//TMOD2//EDA2R//ITSN1//TSPYL5//CD44//CAV1//CLU |
| GO:0043436 | oxoacid metabolic process | Biological process | 42 | 991 | 428 | 14747 | 1.46027801616417 | 0.0086561467871107 | 0.0931854718458937 | 2.06267538760122 | ABCD2//MAT2A//ADI1//PTGDS//PTGIS//RDH10//PDK4//GHR//GSTA2//GSTM5//ASPA//CD34//GATM//C3//PRKAR2B//TNXB//CROT//GPAM//CH25H//PTPLA//C5ORF4//SCD5//ECH1//ECI2//IGF1//FMOD//OMD//OGN//CAV1//FCER1A//MAMDC2//CYB5A//GPC5//GPC3//GPC6//UST//ANGPT1//ANXA1//PLA2G2A//GCNT4//CAB39L//PAPSS2 |
| GO:0048812 | neuron projection morphogenesis | Biological process | 27 | 571 | 428 | 14747 | 1.62924939031376 | 0.00893339182696699 | 0.0958032837246496 | 2.04898361702903 | ANK2//CACNB4//CNTN1//DPYSL3//FGFR1//GFRA1//ITGA9//LAMA2//MYH10//MYH11//OPHN1//PRNP//SLIT3//TRPC1//SLIT2//MYL9//CAP2//BOC//CXCL12//APOD//NR4A2//DCLK1//NFIB//MAP1B//NTRK2//PTPRD//WEE1 |
| GO:0007409 | axonogenesis | Biological process | 25 | 517 | 428 | 14747 | 1.66613188958586 | 0.00893949701351836 | 0.0958032837246496 | 2.04868691637173 | ANK2//CACNB4//CNTN1//DPYSL3//FGFR1//GFRA1//ITGA9//LAMA2//MYH10//MYH11//OPHN1//PRNP//SLIT3//TRPC1//SLIT2//MYL9//CAP2//BOC//CXCL12//APOD//NR4A2//DCLK1//NFIB//MAP1B//NTRK2 |
| GO:1901565 | organonitrogen compound catabolic process | Biological process | 33 | 737 | 428 | 14747 | 1.54278839447622 | 0.00899520773899205 | 0.096184183200119 | 2.04598880242892 | GPC5//GPC3//GPC6//GNAL//GNG11//RHOJ//PDE8B//PDE7B//ACTC1//DPYSL3//ASPA//CD44//FGF2//LYVE1//NTRK2//BVES//ALDH1A1//DLC1//ERRFI1//CNN3//PLN//FGF10//SFRP1//PREX2//CCL21//SPRY1//FMOD//OMD//OGN//ARHGAP6//TPM2//RGS5//RGS22 |
| GO:0060193 | positive regulation of lipase activity | Biological process | 7 | 81 | 428 | 14747 | 2.97764509057344 | 0.00908149713262234 | 0.0968896192963667 | 2.04184254984461 | ARHGAP6//LPAR1//PDE1A//PRKAR2B//FGFR1//FGF2//KIT |
| GO:0010631 | epithelial cell migration | Biological process | 4 | 29 | 428 | 14747 | 4.75249758298421 | 0.0093531148212184 | 0.0993429946155691 | 2.02904373411224 | TGFBR3//FGF10//TGFBR2//KIT |
| GO:0090132 | epithelium migration | Biological process | 4 | 29 | 428 | 14747 | 4.75249758298421 | 0.0093531148212184 | 0.0993429946155691 | 2.02904373411224 | FGF10//TGFBR3//TGFBR2//KIT |
| GO:0032870 | cellular response to hormone stimulus | Biological process | 20 | 387 | 428 | 14747 | 1.78065154918013 | 0.00938400617342497 | 0.0994496120912526 | 2.02761171518457 | FGF2//FGF7//FGF10//FGFR1//PDK4//PIK3C2A//PIK3R1//SORBS1//CAB39L//GHR//MEF2C//GNG7//GNG11//PRKAR2B//SFRP1//ANXA1//AQP1//KIT//SLIT3//SLIT2 |
| GO:0006164 | purine nucleotide biosynthetic process | Biological process | 13 | 213 | 428 | 14747 | 2.10292439998245 | 0.00943357529196648 | 0.0997532606815702 | 2.02532367997789 | CALCRL//AQP1//NME5//GNAL//NTRK2//CAP2//EDNRB//VIP//PTHLH//AKAP12//AMPD1//GNG7//PAPSS2 |
| GO:0060485 | mesenchyme development | Biological process | 11 | 167 | 428 | 14747 | 2.26953103139515 | 0.00956729112655273 | 0.100943388014447 | 2.01921101053286 | EDNRB//TGFBR3//SFRP1//FOXC1//RDH10//FRZB//MEF2C//FGFR1//FGF10//OSR1//TCF21 |
| GO:0003015 | heart process | Biological process | 10 | 145 | 428 | 14747 | 2.3762487914921 | 0.00973631356005998 | 0.102274183629793 | 2.01160544800612 | PLN//ANK2//CASQ2//DMD//EPAS1//CAV1//CHRM2//DES//ACTC1//TTN |
| GO:0060047 | heart contraction | Biological process | 10 | 145 | 428 | 14747 | 2.3762487914921 | 0.00973631356005998 | 0.102274183629793 | 2.01160544800612 | PLN//ANK2//CASQ2//DMD//EPAS1//CAV1//CHRM2//DES//ACTC1//TTN |
| GO:0045926 | negative regulation of growth | Biological process | 12 | 191 | 428 | 14747 | 2.16475020795616 | 0.00997290762844594 | 0.104529223033096 | 2.00117820348473 | BDKRB1//CRYAB//FGF2//FRZB//SFRP1//SLIT3//SLIT2//WFDC1//TSPYL2//CD36//CTSG//MT1A |
| GO:0042523 | positive regulation of tyrosine phosphorylation of Stat5 protein | Biological process | 3 | 16 | 428 | 14747 | 6.46042640186916 | 0.010259680391162 | 0.107064367145408 | 1.98886616812447 | GHR//IGF1//KIT |
| GO:0072224 | metanephric glomerulus development | Biological process | 3 | 16 | 428 | 14747 | 6.46042640186916 | 0.010259680391162 | 0.107064367145408 | 1.98886616812447 | OSR1//CD34//TCF21 |
| GO:0021987 | cerebral cortex development | Biological process | 6 | 64 | 428 | 14747 | 3.23021320093458 | 0.0104721215025072 | 0.108901402795884 | 1.97996532753806 | SLIT2//NTRK2//PTPRS//TACC1//CDON//FOXP2 |
| GO:0003300 | cardiac muscle hypertrophy | Biological process | 4 | 30 | 428 | 14747 | 4.59408099688473 | 0.0105498947560701 | 0.108901402795884 | 1.9767518727915 | IGF1//IL6ST//MYOCD//TTN |
| GO:0014897 | striated muscle hypertrophy | Biological process | 4 | 30 | 428 | 14747 | 4.59408099688473 | 0.0105498947560701 | 0.108901402795884 | 1.9767518727915 | TTN//IGF1//IL6ST//MYOCD |
| GO:0055021 | regulation of cardiac muscle tissue growth | Biological process | 4 | 30 | 428 | 14747 | 4.59408099688473 | 0.0105498947560701 | 0.108901402795884 | 1.9767518727915 | TGFBR2//FGF2//FGFR1//MEF2C |
| GO:0060761 | negative regulation of response to cytokine stimulus | Biological process | 4 | 30 | 428 | 14747 | 4.59408099688473 | 0.0105498947560701 | 0.108901402795884 | 1.9767518727915 | CAV1//SLIT3//SLIT2//IL6ST |
| GO:0046661 | male sex differentiation | Biological process | 9 | 125 | 428 | 14747 | 2.48080373831776 | 0.0106351630593084 | 0.109544476522336 | 1.97325584706685 | SMAD9//GFRA1//KIT//SFRP1//ARID5B//TEX15//FGF10//TCF21//CST3 |
| GO:0060562 | epithelial tube morphogenesis | Biological process | 16 | 290 | 428 | 14747 | 1.90099903319368 | 0.0107728207188551 | 0.110723237086681 | 1.96767056761735 | CXCL12//TGFBR2//CD44//FGF2//PBX1//TCF21//SFRP1//DLC1//MEF2C//FGF10//IGF1//KDR//SLIT2//RDH10//PTHLH//OSR1 |
| GO:0045216 | cell-cell junction organization | Biological process | 11 | 170 | 428 | 14747 | 2.2294804837823 | 0.0108465371619263 | 0.111175574956504 | 1.96470889144733 | CDH5//CADM3//CADM2//ARHGAP6//DLC1//SORBS1//KDR//SFRP1//TEK//APOD//HEG1 |
| GO:0009259 | ribonucleotide metabolic process | Biological process | 30 | 663 | 428 | 14747 | 1.55907726138622 | 0.0108634552169702 | 0.111175574956504 | 1.96403202161623 | CALCRL//AQP1//NME5//GNAL//GNG11//RHOJ//PDE8B//PDE7B//ACTC1//NTRK2//CAP2//EDNRB//CACNB4//PTHLH//VIP//AKAP12//AMPD1//BVES//ALDH1A1//DLC1//ERRFI1//SFRP1//PREX2//CCL21//SPRY1//ARHGAP6//RGS5//RGS22//GNG7//PAPSS2 |
| GO:0001935 | endothelial cell proliferation | Biological process | 7 | 84 | 428 | 14747 | 2.87130062305296 | 0.0109914524773615 | 0.112223758320883 | 1.95894491347075 | CAV1//SCG2//FGF2//KDR//TEK//VIP//CD34 |
| GO:0016043 | cellular component organization | Biological process | 143 | 4176 | 428 | 14747 | 1.1798735318867 | 0.0110129416846662 | 0.112223758320883 | 1.95809666043083 | TACC1//WEE1//IGFBP4//FBLN5//MYOCD//CAPN6//MAP1B//MYH10//CLU//TGFBR3//SRPX//HEG1//ANGPT1//TSPYL2//TSPYL5//DPYSL3//OPHN1//TNXB//DLC1//FERMT2//DAAM2//RHOJ//DES//SGCD//CAP2//WASF3//ZRANB1//SH3D19//SFRP1//MPP6//BMX//EPAS1//CRYAB//CLN5//TNS1//CETN2//PPP1R12A//TTN//TEX15//FMOD//ANK2//CACNB4//CNTN1//FGFR1//GFRA1//ITGA9//LAMA2//MYH11//PRNP//SLIT3//TRPC1//SLIT2//MYL9//BOC//NLGN1//MUSK//CXCL12//KDR//KIT//BVES//PALMD//PARVA//PLSCR4//ANO6//ARHGAP6//DMD//NME5//CST3//NTRK2//LPAR1//PMP22//APOD//FOXC1//CAV1//ZDHHC15//EYA4//NR4A2//DCLK1//NFIB//CMA1//CTSG//ITGA8//COL19A1//ECM2//FBLN1//VIT//TLL1//COL14A1//RECK//GLT25D2//ABI3BP//SMOC2//CRISPLD2//CCDC80//DPT//MEF2C//TMOD1//ACTC1//TAF9B//PPP1R1C//BDKRB1//FGF2//FRZB//WFDC1//CCL21//EPB41L2//AQP1//CNN1//CNN3//PRICKLE2//ANXA1//FGF7//FGF10//VDAC2//SELP//CDH5//FLNC//CADM3//CADM2//PLA2G2A//LIPG//TEK//FZD4//TRIM9//KCNMA1//COLEC12//KCTD12//SORBS1//MFAP5//SYNM//IGF1//PIK3C2A//C3//PTX3//PTPRD//GPX3//NEXN//CD36//PMP2//TOR1AIP1//CD34//CPE//PIK3R1 |
| GO:0010646 | regulation of cell communication | Biological process | 77 | 2063 | 428 | 14747 | 1.28603091405765 | 0.0110867523945865 | 0.112735015287384 | 1.95519565165649 | FGF2//LPAR1//FGF10//GHR//KIT//CAV1//ERRFI1//FCER1A//PRKAR2B//GNG7//RGS5//KCTD12//IL6ST//VIP//TSPYL2//APOD//CASQ2//AKAP12//TEK//NLGN1//ANGPT1//FGFR1//IGF1//KDR//NTRK2//SELP//PPAP2B//SFRP1//ZRANB1//FRZB//APCDD1//TGFBR3//ITGA8//MYOCD//TGFBR2//FZD4//WASF3//NNAT//OXCT1//LAMA2//BVES//ALDH1A1//DLC1//PREX2//CCL21//KANK2//SPRY1//ITSN1//ARHGEF26//PTGIS//RGS22//CD36//SLC44A2//IGFBP4//CDON//TNXB//HIPK3//CD44//CILP//C3//TMOD2//EDA2R//PDE8B//KCNMB4//PRNP//A2M//ARHGAP6//OPHN1//RHOJ//ARHGAP28//SLIT2//TSPYL5//MEF2C//TCF21//SLIT3//VDAC2//CLU |
| GO:0007596 | blood coagulation | Biological process | 24 | 500 | 428 | 14747 | 1.6538691588785 | 0.0112053122117056 | 0.113698157314094 | 1.95057603828716 | ANO6//A2M//SELP//CD36//CLU//CFD//F13A1//IGF1//PIK3R1//TTN//MMRN1//CAV1//CD34//ANGPT1//KCNMA1//PDE1A//PRKAR2B//TEK//WEE1//TFPI2//PAPSS2//KCNMB4//PLSCR4//JAM2 |
| GO:0007204 | elevation of cytosolic calcium ion concentration | Biological process | 11 | 171 | 428 | 14747 | 2.21644258621632 | 0.0113008418355013 | 0.114296928023811 | 1.94688920334003 | CAV1//CASQ2//DMD//ANK2//PLN//FGF2//CCL21//BDKRB1//TRPC1//LPAR1//EDNRB |
| GO:0097305 | response to alcohol | Biological process | 13 | 218 | 428 | 14747 | 2.05469218897368 | 0.0113122562439167 | 0.114296928023811 | 1.94645076584751 | ANXA1//CRYAB//CST3//FGF10//GHR//WFDC1//ACTC1//OXCT1//SLIT3//SLIT2//TGFBR2//SFRP1//AQP1 |
| GO:0070848 | response to growth factor stimulus | Biological process | 22 | 447 | 428 | 14747 | 1.69580171025947 | 0.0113469887706836 | 0.114405474518795 | 1.94511937492771 | CCNC//SMAD9//TGFBR2//TGFBR3//FMOD//FGF2//FGF7//FGF10//FGFR1//PDE1A//PIK3R1//PRKAR2B//ITGA8//MYOCD//FZD4//KDR//SPRY1//CD44//SFRP1//MEF2C//DMD//SLIT2 |
| GO:0060429 | epithelium development | Biological process | 28 | 610 | 428 | 14747 | 1.58156886777999 | 0.0114075429009887 | 0.114773358849821 | 1.94280788927653 | CXCL12//TGFBR2//CD44//FGF2//PBX1//TCF21//SFRP1//DLC1//HEG1//MEF2C//DMD//ANXA1//PTHLH//CAV1//OSR1//KDR//APOLD1//ERRFI1//FGF10//IGF1//SLIT2//FRZB//RDH10//FGF7//FGFR1//NFIB//FOXP2//CD34 |
| GO:0010647 | positive regulation of cell communication | Biological process | 37 | 865 | 428 | 14747 | 1.473823672411 | 0.0116818525190532 | 0.117285799291294 | 1.93248828093879 | LPAR1//FGF2//FGF10//GHR//KIT//FCER1A//AKAP12//TEK//NLGN1//ANGPT1//FGFR1//IGF1//KDR//NTRK2//SELP//SFRP1//ZRANB1//TGFBR3//ITGA8//MYOCD//LAMA2//PTGIS//IL6ST//CD36//CCL21//SLC44A2//IGFBP4//CDON//FZD4//C3//TMOD2//EDA2R//ITSN1//TSPYL5//CD44//CAV1//CLU |
| GO:0014909 | smooth muscle cell migration | Biological process | 4 | 31 | 428 | 14747 | 4.4458848356949 | 0.0118413396919217 | 0.118388572307704 | 1.92659916012196 | IGF1//SLIT2//PARVA//LPAR1 |
| GO:0060740 | prostate gland epithelium morphogenesis | Biological process | 4 | 31 | 428 | 14747 | 4.4458848356949 | 0.0118413396919217 | 0.118388572307704 | 1.92659916012196 | CD44//IGF1//SFRP1//FGF10 |
| GO:0019693 | ribose phosphate metabolic process | Biological process | 30 | 668 | 428 | 14747 | 1.54740752140579 | 0.0119731698631232 | 0.1193588490541 | 1.92179085612541 | CALCRL//AQP1//NME5//GNAL//GNG11//RHOJ//PDE8B//PDE7B//ACTC1//NTRK2//CAP2//EDNRB//CACNB4//PTHLH//VIP//AKAP12//AMPD1//BVES//ALDH1A1//DLC1//ERRFI1//SFRP1//PREX2//CCL21//SPRY1//ARHGAP6//RGS5//RGS22//GNG7//PAPSS2 |
| GO:0050817 | coagulation | Biological process | 24 | 503 | 428 | 14747 | 1.64400512810985 | 0.0119884438450228 | 0.1193588490541 | 1.92123718665766 | ANO6//A2M//ANGPT1//CAV1//CD36//CLU//CFD//F13A1//IGF1//KCNMA1//PDE1A//PIK3R1//PRKAR2B//SELP//TEK//TTN//WEE1//TFPI2//PAPSS2//MMRN1//KCNMB4//PLSCR4//JAM2//CD34 |
| GO:0006956 | complement activation | Biological process | 6 | 66 | 428 | 14747 | 3.13232795242141 | 0.0120950377083923 | 0.119422846441662 | 1.9173927733111 | A2M//C3//C7//CFD//CFH//CLU |
| GO:0014033 | neural crest cell differentiation | Biological process | 6 | 66 | 428 | 14747 | 3.13232795242141 | 0.0120950377083923 | 0.119422846441662 | 1.9173927733111 | EDNRB//FOXC1//RDH10//SFRP1//FRZB//MEF2C |
| GO:0021549 | cerebellum development | Biological process | 6 | 66 | 428 | 14747 | 3.13232795242141 | 0.0120950377083923 | 0.119422846441662 | 1.9173927733111 | MYH10//FAIM2//FZD4//CNTN1//PTPRS//FOXP2 |
| GO:0051897 | positive regulation of protein kinase B signaling cascade | Biological process | 6 | 66 | 428 | 14747 | 3.13232795242141 | 0.0120950377083923 | 0.119422846441662 | 1.9173927733111 | ANGPT1//IGF1//CCL21//ITSN1//TEK//TSPYL5 |
| GO:0010761 | fibroblast migration | Biological process | 3 | 17 | 428 | 14747 | 6.08040131940627 | 0.0121939781601737 | 0.119903261537873 | 1.91385458714947 | FGF2//TNS1//ARID5B |
| GO:0060572 | morphogenesis of an epithelial bud | Biological process | 3 | 17 | 428 | 14747 | 6.08040131940627 | 0.0121939781601737 | 0.119903261537873 | 1.91385458714947 | RDH10//FGF10//PTHLH |
| GO:0007599 | hemostasis | Biological process | 24 | 504 | 428 | 14747 | 1.64074321317312 | 0.0122590788337194 | 0.120295364111127 | 1.91154216215551 | ANO6//A2M//ANGPT1//CAV1//CD36//CLU//CFD//F13A1//IGF1//KCNMA1//PDE1A//PIK3R1//PRKAR2B//SELP//TEK//TTN//WEE1//TFPI2//PAPSS2//MMRN1//KCNMB4//PLSCR4//JAM2//CD34 |
| GO:0030850 | prostate gland development | Biological process | 5 | 48 | 428 | 14747 | 3.5891257788162 | 0.012362154823339 | 0.121057733783375 | 1.90790582161616 | SFRP1//CD44//IGF1//FGF10//SERPINF1 |
| GO:0032940 | secretion by cell | Biological process | 31 | 698 | 428 | 14747 | 1.53026336930616 | 0.01243125432571 | 0.121484942375637 | 1.90548504840979 | FCER1A//A2M//CD36//CLU//CFD//F13A1//IGF1//SELP//TTN//MMRN1//NLGN1//MYH10//PIK3C2A//EXOC6//PRKAR2B//SCG2//CBLN4//NTRK2//TRIM9//ANXA1//NNAT//OXCT1//FZD4//KIT//SFRP1//PDE8B//KCNMB4//GPAM//FGF7//FGF10//CD34 |
| GO:0051246 | regulation of protein metabolic process | Biological process | 57 | 1460 | 428 | 14747 | 1.34518467545769 | 0.0124821714375142 | 0.121733078906964 | 1.90370985685727 | CCNC//DIRAS3//FGF2//LPAR1//FGF10//GHR//KIT//CAV1//PSTK//A2M//BDKRB1//PRNP//PPAP2B//SLIT2//C3//EDNRB//MUSK//TEK//CDON//ERRFI1//LARP6//FABP4//TSPYL2//ANGPT1//FCER1A//CCL21//SERPINA6//SERPINF1//CFH//TSPYL5//TGFBR2//CLU//RNF180//DMD//CD44//PRKAR2B//MYOCD//DLC1//IL6ST//IGF1//FGFR1//SPRY1//TNXB//FZD4//SFRP1//HIPK3//VIP//SPDYA//TTN//PDGFD//CD36//FGF7//LIPG//SH3D19//TIMP3//CST3//APOD |
| GO:0009260 | ribonucleotide biosynthetic process | Biological process | 13 | 221 | 428 | 14747 | 2.02680043980209 | 0.012571461521899 | 0.122353673465176 | 1.90061422955362 | CALCRL//AQP1//NME5//GNAL//NTRK2//CAP2//EDNRB//VIP//PTHLH//AKAP12//AMPD1//GNG7//PAPSS2 |
| GO:0048660 | regulation of smooth muscle cell proliferation | Biological process | 6 | 67 | 428 | 14747 | 3.08557678895243 | 0.0129694602069679 | 0.125970174596802 | 1.88707809901335 | IGF1//TGFBR2//CALCRL//APOD//OGN//VIP |
| GO:0010828 | positive regulation of glucose transport | Biological process | 4 | 32 | 428 | 14747 | 4.30695093457944 | 0.0132299576726398 | 0.127719975993561 | 1.87844154527345 | IGF1//PIK3R1//SORBS1//C3 |
| GO:0014896 | muscle hypertrophy | Biological process | 4 | 32 | 428 | 14747 | 4.30695093457944 | 0.0132299576726398 | 0.127719975993561 | 1.87844154527345 | TTN//IGF1//IL6ST//MYOCD |
| GO:1901019 | regulation of calcium ion transmembrane transporter activity | Biological process | 4 | 32 | 428 | 14747 | 4.30695093457944 | 0.0132299576726398 | 0.127719975993561 | 1.87844154527345 | DMD//PLN//CASQ2//ANK2 |
| GO:0001508 | regulation of action potential | Biological process | 10 | 152 | 428 | 14747 | 2.2668162813576 | 0.013268003586575 | 0.127828503241164 | 1.87719441975509 | KCNMA1//KCNMB4//ASPA//WASF3//PMP22//LPAR1//MPDZ//KCNIP1//ANK2//DMD |
| GO:0001933 | negative regulation of protein phosphorylation | Biological process | 12 | 199 | 428 | 14747 | 2.07772507396797 | 0.0134892851065553 | 0.12964772488202 | 1.87001106608089 | CAV1//ERRFI1//FABP4//DMD//SPRY1//SFRP1//HIPK3//MYOCD//BDKRB1//PRNP//PPAP2B//SLIT2 |
| GO:0000302 | response to reactive oxygen species | Biological process | 9 | 130 | 428 | 14747 | 2.38538820992092 | 0.0135112013559161 | 0.12964772488202 | 1.86930603372786 | SOD3//CRYAB//GPX3//ANXA1//AQP1//CST3//CD36//FBLN5//APOD |
| GO:0070374 | positive regulation of ERK1 and ERK2 cascade | Biological process | 7 | 88 | 428 | 14747 | 2.74078695836873 | 0.0139705899131345 | 0.133402737335802 | 1.85478525525947 | ANGPT1//CD44//FGF2//FGF10//KDR//CCL21//TEK |
| GO:0016202 | regulation of striated muscle tissue development | Biological process | 8 | 109 | 428 | 14747 | 2.52885192489068 | 0.014014420508542 | 0.133402737335802 | 1.85342485545958 | MUSK//MYOCD//MEF2C//BOC//CDON//TGFBR2//FGF2//FGFR1 |
| GO:0034599 | cellular response to oxidative stress | Biological process | 8 | 109 | 428 | 14747 | 2.52885192489068 | 0.014014420508542 | 0.133402737335802 | 1.85342485545958 | SOD3//GPX3//EPAS1//ANXA1//AQP1//CST3//CD36//FBLN5 |
| GO:1901861 | regulation of muscle tissue development | Biological process | 8 | 109 | 428 | 14747 | 2.52885192489068 | 0.014014420508542 | 0.133402737335802 | 1.85342485545958 | MUSK//MYOCD//MEF2C//BOC//CDON//TGFBR2//FGF2//FGFR1 |
| GO:0042391 | regulation of membrane potential | Biological process | 15 | 274 | 428 | 14747 | 1.88625588375742 | 0.0141864590719909 | 0.134771361183914 | 1.84812599054652 | KCNMB4//KCNMA1//ASPA//WASF3//PMP22//LPAR1//MPDZ//KCNIP1//CACNB4//CAV1//CASQ2//ANK2//DMD//PLN//NLGN1 |
| GO:0000303 | response to superoxide | Biological process | 3 | 18 | 428 | 14747 | 5.74260124610592 | 0.0143229313303802 | 0.134862426944866 | 1.84396809024551 | SOD3//CD36//FBLN5 |
| GO:0001953 | negative regulation of cell-matrix adhesion | Biological process | 3 | 18 | 428 | 14747 | 5.74260124610592 | 0.0143229313303802 | 0.134862426944866 | 1.84396809024551 | APOD//ARHGAP6//PIK3R1 |
| GO:0006929 | substrate-dependent cell migration | Biological process | 3 | 18 | 428 | 14747 | 5.74260124610592 | 0.0143229313303802 | 0.134862426944866 | 1.84396809024551 | MYH10//OPHN1//SLIT2 |
| GO:0034694 | response to prostaglandin stimulus | Biological process | 3 | 18 | 428 | 14747 | 5.74260124610592 | 0.0143229313303802 | 0.134862426944866 | 1.84396809024551 | CCL21//TGFBR3//SFRP1 |
| GO:0048667 | cell morphogenesis involved in neuron differentiation | Biological process | 26 | 566 | 428 | 14747 | 1.5827664211882 | 0.0143374398115008 | 0.134862426944866 | 1.84352839225287 | ANK2//CACNB4//CNTN1//DPYSL3//FGFR1//GFRA1//ITGA9//LAMA2//MYH10//MYH11//OPHN1//PRNP//SLIT3//TRPC1//SLIT2//MYL9//CAP2//BOC//CXCL12//APOD//NR4A2//DCLK1//NFIB//MAP1B//NTRK2//PTPRD |
| GO:0046390 | ribose phosphate biosynthetic process | Biological process | 13 | 225 | 428 | 14747 | 1.99076843198339 | 0.0144155808768475 | 0.13524187827694 | 1.84116785299642 | CALCRL//AQP1//NME5//GNAL//NTRK2//CAP2//EDNRB//VIP//PTHLH//AKAP12//AMPD1//GNG7//PAPSS2 |
| GO:0007611 | learning or memory | Biological process | 10 | 154 | 428 | 14747 | 2.23737710887244 | 0.014434496968539 | 0.13524187827694 | 1.84059834635855 | NTRK2//PRKAR2B//PTN//MUSK//ITGA8//FOXP2//MEF2C//PRNP//VIP//TMOD2 |
| GO:0023051 | regulation of signaling | Biological process | 76 | 2057 | 428 | 14747 | 1.27303168119801 | 0.0146164397012037 | 0.13600640154958 | 1.83515840073118 | FGF2//LPAR1//FGF10//GHR//KIT//CAV1//BVES//ERRFI1//FCER1A//PRKAR2B//GNG7//RGS5//KCTD12//IL6ST//VIP//TSPYL2//APOD//AKAP12//TEK//NLGN1//ANGPT1//FGFR1//IGF1//KDR//NTRK2//SELP//PPAP2B//SFRP1//ZRANB1//FRZB//APCDD1//TGFBR3//ITGA8//MYOCD//TGFBR2//FZD4//WASF3//NNAT//OXCT1//LAMA2//ALDH1A1//DLC1//PREX2//CCL21//KANK2//SPRY1//ITSN1//ARHGEF26//PTGIS//RGS22//CD36//SLC44A2//IGFBP4//CDON//TNXB//HIPK3//CD44//CILP//C3//TMOD2//EDA2R//PDE8B//KCNMB4//PRNP//A2M//ARHGAP6//OPHN1//RHOJ//ARHGAP28//SLIT2//TSPYL5//MEF2C//TCF21//SLIT3//VDAC2//CLU |
| GO:0055065 | metal ion homeostasis | Biological process | 18 | 352 | 428 | 14747 | 1.76193447323704 | 0.0146873227014468 | 0.13600640154958 | 1.83305736295427 | CORIN//EDNRB//CASQ2//ANK2//CACNB4//CAV1//CXCL12//CYBRD1//BDKRB1//LPAR1//DMD//PLN//SLC22A17//KCNMA1//FGF2//CCL21//TRPC1//KDR |
| GO:0010812 | negative regulation of cell-substrate adhesion | Biological process | 4 | 33 | 428 | 14747 | 4.17643726989521 | 0.0147180748085628 | 0.13600640154958 | 1.83214899398617 | PIK3R1//APOD//ARHGAP6//FZD4 |
| GO:0030318 | melanocyte differentiation | Biological process | 4 | 33 | 428 | 14747 | 4.17643726989521 | 0.0147180748085628 | 0.13600640154958 | 1.83214899398617 | ADAMTS9//EDNRB//KIT//MEF2C |
| GO:0045762 | positive regulation of adenylate cyclase activity | Biological process | 4 | 33 | 428 | 14747 | 4.17643726989521 | 0.0147180748085628 | 0.13600640154958 | 1.83214899398617 | GNAL//NTRK2//CAP2//VIP |
| GO:0060512 | prostate gland morphogenesis | Biological process | 4 | 33 | 428 | 14747 | 4.17643726989521 | 0.0147180748085628 | 0.13600640154958 | 1.83214899398617 | CD44//IGF1//SFRP1//FGF10 |
| GO:0048634 | regulation of muscle organ development | Biological process | 8 | 110 | 428 | 14747 | 2.50586236193713 | 0.0147442460895644 | 0.13600640154958 | 1.83137742911508 | MUSK//MYOCD//MEF2C//BOC//CDON//TGFBR2//FGF2//FGFR1 |
| GO:0050880 | regulation of blood vessel size | Biological process | 8 | 110 | 428 | 14747 | 2.50586236193713 | 0.0147442460895644 | 0.13600640154958 | 1.83137742911508 | VIP//EDNRB//ACTA2//PIK3C2A//KCNMB4//CAV1//KCNMA1//FOXC1 |
| GO:0072006 | nephron development | Biological process | 7 | 89 | 428 | 14747 | 2.70999159928594 | 0.0147975507680707 | 0.136084937237753 | 1.82981016135745 | TCF21//CD34//ANGPT1//TEK//MEF2C//ACTA2//OSR1 |
| GO:0070371 | ERK1 and ERK2 cascade | Biological process | 9 | 132 | 428 | 14747 | 2.34924596431606 | 0.0148098306618565 | 0.136084937237753 | 1.82944990724798 | IGF1//SPRY1//ANGPT1//CD44//FGF2//FGF10//KDR//CCL21//TEK |
| GO:0009966 | regulation of signal transduction | Biological process | 68 | 1812 | 428 | 14747 | 1.29303604216954 | 0.0151416588202442 | 0.138866482526432 | 1.81982654379136 | FGF2//LPAR1//FGF10//GHR//KIT//CAV1//ERRFI1//FCER1A//GNG7//RGS5//KCTD12//IL6ST//APOD//AKAP12//TEK//NLGN1//ANGPT1//FGFR1//IGF1//KDR//NTRK2//SELP//PPAP2B//SFRP1//ZRANB1//FRZB//APCDD1//TGFBR3//ITGA8//MYOCD//TGFBR2//FZD4//BVES//ALDH1A1//DLC1//PREX2//CCL21//KANK2//SPRY1//ITSN1//ARHGEF26//PTGIS//RGS22//CD36//SLC44A2//IGFBP4//CDON//TNXB//HIPK3//CD44//CILP//C3//TMOD2//EDA2R//PRNP//A2M//ARHGAP6//OPHN1//RHOJ//ARHGAP28//SLIT2//TSPYL5//TCF21//SLIT3//VDAC2//CLU//VIP//TSPYL2 |
| GO:0007265 | Ras protein signal transduction | Biological process | 19 | 380 | 428 | 14747 | 1.72278037383178 | 0.015381303758118 | 0.140793546300316 | 1.81300685107823 | ARHGAP6//CTNNAL1//RHOJ//ITSN1//ARHGEF26//DLC1//LPAR1//FGF10//IGF1//SPRY1//FGF2//KSR1 |
| GO:0035150 | regulation of tube size | Biological process | 8 | 111 | 428 | 14747 | 2.4832870253431 | 0.0155013063202164 | 0.14162017210941 | 1.80963170158738 | VIP//EDNRB//ACTA2//PIK3C2A//KCNMB4//CAV1//FOXC1//KCNMA1 |
| GO:0051896 | regulation of protein kinase B signaling cascade | Biological process | 7 | 90 | 428 | 14747 | 2.6798805815161 | 0.0156588233743071 | 0.142785714478146 | 1.80524087454142 | ANGPT1//IGF1//CCL21//ITSN1//TEK//TSPYL5//NTRK2 |
| GO:0001938 | positive regulation of endothelial cell proliferation | Biological process | 5 | 51 | 428 | 14747 | 3.37800073300348 | 0.0158085565342972 | 0.143222240362094 | 1.80110778331314 | FGF2//KDR//TEK//VIP//SCG2 |
| GO:0002062 | chondrocyte differentiation | Biological process | 6 | 70 | 428 | 14747 | 2.95333778371162 | 0.0158568552969565 | 0.143222240362094 | 1.79978293698023 | EFEMP1//PTHLH//FGFR1//MEF2C//NFIB//OSR1 |
| GO:0045807 | positive regulation of endocytosis | Biological process | 6 | 70 | 428 | 14747 | 2.95333778371162 | 0.0158568552969565 | 0.143222240362094 | 1.79978293698023 | ANGPT1//CCL21//C3//PTX3//CD36//NLGN1 |
| GO:0048659 | smooth muscle cell proliferation | Biological process | 6 | 70 | 428 | 14747 | 2.95333778371162 | 0.0158568552969565 | 0.143222240362094 | 1.79978293698023 | IGF1//TGFBR2//CALCRL//APOD//OGN//VIP |
| GO:0050729 | positive regulation of inflammatory response | Biological process | 6 | 70 | 428 | 14747 | 2.95333778371162 | 0.0158568552969565 | 0.143222240362094 | 1.79978293698023 | C3//FCER1A//IL6ST//PTGER3//FABP4//PLA2G2A |
| GO:0050790 | regulation of catalytic activity | Biological process | 56 | 1449 | 428 | 14747 | 1.33161768025644 | 0.0159595233953857 | 0.143877064409441 | 1.7969800822738 | CCNC//DIRAS3//FGF2//LPAR1//FGF10//GHR//KIT//CAV1//ERRFI1//FABP4//DLC1//TSPYL2//ANGPT1//GNAL//NTRK2//CAP2//EDNRB//ARHGAP6//PDE1A//PRKAR2B//FCER1A//CST3//FGFR1//VIP//MEF2C//SERPINA6//SERPINF1//RECK//TGFBR2//BVES//ALDH1A1//CNN3//PLN//SFRP1//PREX2//CCL21//SPRY1//PPP1R12A//NR4A2//AQP1//CD44//CRYAB//TPM2//TNXB//FZD4//HIPK3//RGS5//RGS22//PIK3R1//TEK//RCAN1//MYOCD//SPDYA//GNG7//TTN//PARM1 |
| GO:0006950 | response to stress | Biological process | 104 | 2953 | 428 | 14747 | 1.21347212244162 | 0.0160853086126446 | 0.14473742787491 | 1.79357060231048 | APOD//GNG7//CAV1//CRYAB//CST3//EPAS1//KCNMA1//SMAD9//NR4A2//SOD3//TGFBR3//VCAM1//PDLIM1//APOLD1//MYOCD//MYH10//C3//FCER1A//A2M//COLEC12//MEF2C//CD44//ANO6//ANXA1//IL6ST//DARC//HTN3//CTSG//ANGPT1//CD36//CLU//CFD//F13A1//IGF1//PDE1A//PIK3R1//PRKAR2B//SELP//TEK//TTN//WEE1//TFPI2//PAPSS2//MMRN1//KCNMB4//PLSCR4//JAM2//EYA4//CETN2//GTF2H5//BDKRB1//IGFBP4//KIT//PTX3//CCL21//SCG2//AOC3//KLRG1//C7//CFH//SH2D1A//CD160//FMO1//NFATC2//SPDYA//GATM//PRNP//SEPP1//SCARA3//GPX3//TSPYL2//PLA2G2A//PDK4//SFRP1//FGF7//LYVE1//TEX15//REV3L//AQP1//SRPX//CD34//PTGER3//FGF2//FGF10//TGFBR2//OXCT1//TNXB//FZD4//HIPK3//EDA2R//EDNRB//DPYSL3//CMA1//PTGIS//CALCRL//FABP4//ABCC9//GPAM//ANKRD42//FBLN5//BMX//ERRFI1//HSPB6 |
| GO:0050801 | ion homeostasis | Biological process | 30 | 684 | 428 | 14747 | 1.5112108542384 | 0.0161590114656715 | 0.145126790357415 | 1.79158521089655 | KCNMB4//CORIN//EDNRB//CASQ2//ANK2//CACNB4//CAV1//CXCL12//PRNP//CYBRD1//PDK4//SLC9A9//CLN5//BDKRB1//LPAR1//DMD//PLN//SLC22A17//KCNMA1//ASPA//WASF3//PMP22//MPDZ//KCNIP1//FGF2//CCL21//TRPC1//KDR//GPAM//NLGN1 |
| GO:0006633 | fatty acid biosynthetic process | Biological process | 9 | 134 | 428 | 14747 | 2.31418259171433 | 0.0161982547192282 | 0.145205783375939 | 1.79053177599074 | PTGDS//PTGIS//FCER1A//ANXA1//PDK4//CH25H//PTPLA//C5ORF4//SCD5 |
| GO:0035239 | tube morphogenesis | Biological process | 16 | 304 | 428 | 14747 | 1.81345302508608 | 0.0162313472523905 | 0.145229446616605 | 1.78964543088261 | CXCL12//TGFBR2//CD44//FGF2//PBX1//TCF21//SFRP1//DLC1//MEF2C//FGF10//IGF1//KDR//SLIT2//RDH10//PTHLH//OSR1 |
| GO:0034446 | substrate adhesion-dependent cell spreading | Biological process | 4 | 34 | 428 | 14747 | 4.05360087960418 | 0.016307833763271 | 0.14536833498512 | 1.78760372425337 | TEK//FZD4//BVES//PARVA |
| GO:0090130 | tissue migration | Biological process | 4 | 34 | 428 | 14747 | 4.05360087960418 | 0.016307833763271 | 0.14536833498512 | 1.78760372425337 | FGF10//TGFBR3//TGFBR2//KIT |
| GO:0032844 | regulation of homeostatic process | Biological process | 15 | 279 | 428 | 14747 | 1.85245201487288 | 0.0164636872424173 | 0.146253019204907 | 1.7834728927142 | CORIN//EDNRB//CAV1//CASQ2//DMD//ANK2//PLN//PTGER3//WASF3//ANXA1//FGF2//CCL21//BDKRB1//TRPC1//GPAM |
| GO:0021591 | ventricular system development | Biological process | 3 | 19 | 428 | 14747 | 5.44035907525824 | 0.0166488946961865 | 0.146253019204907 | 1.77861459358595 | MYH10//AQP1//NME5 |
| GO:0051647 | nucleus localization | Biological process | 3 | 19 | 428 | 14747 | 5.44035907525824 | 0.0166488946961865 | 0.146253019204907 | 1.77861459358595 | MYH10//TACC1//DMD |
| GO:0060045 | positive regulation of cardiac muscle cell proliferation | Biological process | 3 | 19 | 428 | 14747 | 5.44035907525824 | 0.0166488946961865 | 0.146253019204907 | 1.77861459358595 | FGF2//FGFR1//MEF2C |
| GO:0060314 | regulation of ryanodine-sensitive calcium-release channel activity | Biological process | 3 | 19 | 428 | 14747 | 5.44035907525824 | 0.0166488946961865 | 0.146253019204907 | 1.77861459358595 | CASQ2//DMD//PLN |
| GO:0060487 | lung epithelial cell differentiation | Biological process | 3 | 19 | 428 | 14747 | 5.44035907525824 | 0.0166488946961865 | 0.146253019204907 | 1.77861459358595 | NFIB//IGF1//FGF10 |
| GO:2000209 | regulation of anoikis | Biological process | 3 | 19 | 428 | 14747 | 5.44035907525824 | 0.0166488946961865 | 0.146253019204907 | 1.77861459358595 | CAV1//NTRK2//PDK4 |
| GO:0003007 | heart morphogenesis | Biological process | 11 | 181 | 428 | 14747 | 2.09398719471266 | 0.0166830871141685 | 0.146253019204907 | 1.77772358243961 | MEF2C//PARVA//HEG1//CPE//ACTC1//TTN//FOXC1//TGFBR3//TEK//ADAMTS1//DLC1 |
| GO:0051480 | cytosolic calcium ion homeostasis | Biological process | 11 | 181 | 428 | 14747 | 2.09398719471266 | 0.0166830871141685 | 0.146253019204907 | 1.77772358243961 | BDKRB1//LPAR1//EDNRB//CAV1//CASQ2//DMD//ANK2//PLN//FGF2//CCL21//TRPC1 |
| GO:0019637 | organophosphate metabolic process | Biological process | 48 | 1212 | 428 | 14747 | 1.36457851392616 | 0.0168323576700323 | 0.147290850877769 | 1.77385504912385 | CALCRL//AQP1//NME5//GNAL//GNG11//RHOJ//PDE8B//PDE7B//ACTC1//DPM1//PIK3C2A//PIK3R1//PLA2G2A//UGCG//PPAP2A//PPAP2B//PEMT//SLC44A2//GPAM//FGF2//NTRK2//CAP2//EDNRB//CACNB4//PTHLH//LIPG//VIP//AKAP12//AMPD1//BVES//ALDH1A1//DLC1//ERRFI1//CNN3//PLN//FGF10//SFRP1//PREX2//CCL21//SPRY1//ARHGAP6//TPM2//RGS5//RGS22//GNG7//CMAHP//PAPSS2//FMO1 |
| GO:0044344 | cellular response to fibroblast growth factor stimulus | Biological process | 10 | 158 | 428 | 14747 | 2.18073465041997 | 0.0169951835835414 | 0.148443279322178 | 1.76967413976651 | FGF2//FGF7//FGF10//FGFR1//PDE1A//PIK3R1//PRKAR2B//SPRY1//CD44//SFRP1 |
| GO:0042509 | regulation of tyrosine phosphorylation of STAT protein | Biological process | 5 | 52 | 428 | 14747 | 3.31303918044572 | 0.0170861153358065 | 0.148807260036697 | 1.76735666636032 | IL6ST//KIT//GHR//IGF1//CAV1 |
| GO:0055123 | digestive system development | Biological process | 8 | 113 | 428 | 14747 | 2.43933504259366 | 0.017099261585261 | 0.148807260036697 | 1.76702264378157 | EDNRB//IGF1//SFRP1//FGF10//TCF21//KIT//TGFBR2//MYOCD |
| GO:0009894 | regulation of catabolic process | Biological process | 24 | 520 | 428 | 14747 | 1.59025880661395 | 0.0172976320063618 | 0.150259393512458 | 1.76201334640646 | ABCD2//NTRK2//BVES//ALDH1A1//DLC1//ERRFI1//CLU//RNF180//CNN3//PLN//FGF10//SFRP1//PREX2//CCL21//SPRY1//ARHGAP6//TPM2//RGS5//RGS22//VIP//IGF1//SH3D19//TIMP3//CST3 |
| GO:0048545 | response to steroid hormone stimulus | Biological process | 14 | 256 | 428 | 14747 | 1.8842910338785 | 0.0175986009714269 | 0.152595869150427 | 1.75452185573922 | ANXA1//CRYAB//CST3//FGF10//GHR//WFDC1//CAV1//RCAN1//TGFBR2//PTGDS//SLIT3//SLIT2//SFRP1//AQP1 |
| GO:0071774 | response to fibroblast growth factor stimulus | Biological process | 10 | 159 | 428 | 14747 | 2.16701933815318 | 0.0176847618690434 | 0.153064663073445 | 1.75240078372351 | FGF2//FGF7//FGF10//FGFR1//PDE1A//PIK3R1//PRKAR2B//SPRY1//CD44//SFRP1 |
| GO:0002009 | morphogenesis of an epithelium | Biological process | 19 | 386 | 428 | 14747 | 1.69600140429035 | 0.0178300739819802 | 0.153662970683291 | 1.74884685481124 | CXCL12//TGFBR2//CD44//FGF2//PBX1//TCF21//SFRP1//DLC1//MEF2C//FGF10//IGF1//KDR//SLIT2//FRZB//RDH10//FGF7//FGFR1//PTHLH//OSR1 |
| GO:0014812 | muscle cell migration | Biological process | 4 | 35 | 428 | 14747 | 3.93778371161549 | 0.0180011930131115 | 0.153662970683291 | 1.74469871146104 | IGF1//SLIT2//PARVA//LPAR1 |
| GO:0060420 | regulation of heart growth | Biological process | 4 | 35 | 428 | 14747 | 3.93778371161549 | 0.0180011930131115 | 0.153662970683291 | 1.74469871146104 | TGFBR2//FGF2//FGFR1//MEF2C |
| GO:0061383 | trabecula morphogenesis | Biological process | 4 | 35 | 428 | 14747 | 3.93778371161549 | 0.0180011930131115 | 0.153662970683291 | 1.74469871146104 | SFRP1//TEK//TGFBR3//ADAMTS1 |
| GO:2001257 | regulation of cation channel activity | Biological process | 4 | 35 | 428 | 14747 | 3.93778371161549 | 0.0180011930131115 | 0.153662970683291 | 1.74469871146104 | DMD//PLN//CASQ2//ANK2 |
| GO:0001936 | regulation of endothelial cell proliferation | Biological process | 6 | 72 | 428 | 14747 | 2.87130062305296 | 0.0180116587569636 | 0.153662970683291 | 1.74444628963335 | CAV1//SCG2//FGF2//KDR//TEK//VIP |
| GO:0007589 | body fluid secretion | Biological process | 6 | 72 | 428 | 14747 | 2.87130062305296 | 0.0180116587569636 | 0.153662970683291 | 1.74444628963335 | CAV1//AQP1//KCNMA1//TRPC1//FGF10//VIP |
| GO:0060048 | cardiac muscle contraction | Biological process | 6 | 72 | 428 | 14747 | 2.87130062305296 | 0.0180116587569636 | 0.153662970683291 | 1.74444628963335 | ANK2//CASQ2//DMD//PLN//ACTC1//TTN |
| GO:0032319 | regulation of Rho GTPase activity | Biological process | 7 | 93 | 428 | 14747 | 2.59343282082203 | 0.0184554705433945 | 0.156617976677316 | 1.7338748774911 | NTRK2//BVES//DLC1//ERRFI1//SFRP1//PREX2//CCL21 |
| GO:0030308 | negative regulation of cell growth | Biological process | 9 | 137 | 428 | 14747 | 2.2635070605089 | 0.0184565533429758 | 0.156617976677316 | 1.73384939777833 | BDKRB1//CRYAB//FGF2//FRZB//SFRP1//SLIT3//SLIT2//WFDC1//TSPYL2 |
| GO:0050795 | regulation of behavior | Biological process | 9 | 137 | 428 | 14747 | 2.2635070605089 | 0.0184565533429758 | 0.156617976677316 | 1.73384939777833 | SLIT2//PTGDS//CCL21//KDR//FGF10//CXCL12//SCG2//LPAR1//MEF2C |
| GO:0001934 | positive regulation of protein phosphorylation | Biological process | 26 | 579 | 428 | 14747 | 1.54722935128242 | 0.018579301190128 | 0.157296864682956 | 1.73097062484069 | FGF2//LPAR1//FGF10//GHR//KIT//TSPYL2//ANGPT1//FCER1A//TGFBR2//CAV1//CD44//TEK//PRKAR2B//IL6ST//IGF1//FGFR1//FZD4//SPDYA//CCL21//CD36//FGF7//PPAP2B//C3//EDNRB//MUSK//CDON |
| GO:0051050 | positive regulation of transport | Biological process | 23 | 496 | 428 | 14747 | 1.59773986282786 | 0.0186025228939373 | 0.157296864682956 | 1.73042815230824 | ANGPT1//EDNRB//AQP1//CAV1//CNTN1//C3//IGF1//PIK3R1//SORBS1//ANXA1//NNAT//OXCT1//LIPG//ANK2//FCER1A//CCL21//PTX3//BDKRB1//TRPC1//CD36//CD34//NLGN1//DMD |
| GO:0010594 | regulation of endothelial cell migration | Biological process | 6 | 73 | 428 | 14747 | 2.83196773780566 | 0.0191608198085714 | 0.160418157983501 | 1.71758591327825 | ANGPT1//KDR//TEK//SLIT2//FGF2//PPAP2B |
| GO:0030449 | regulation of complement activation | Biological process | 3 | 20 | 428 | 14747 | 5.16834112149533 | 0.0191734850179483 | 0.160418157983501 | 1.71729894155568 | A2M//C3//CFH |
| GO:0046688 | response to copper ion | Biological process | 3 | 20 | 428 | 14747 | 5.16834112149533 | 0.0191734850179483 | 0.160418157983501 | 1.71729894155568 | AQP1//PRNP//SOD3 |
| GO:0055012 | ventricular cardiac muscle cell differentiation | Biological process | 3 | 20 | 428 | 14747 | 5.16834112149533 | 0.0191734850179483 | 0.160418157983501 | 1.71729894155568 | MYH10//MEF2C//MYOCD |
| GO:0060479 | lung cell differentiation | Biological process | 3 | 20 | 428 | 14747 | 5.16834112149533 | 0.0191734850179483 | 0.160418157983501 | 1.71729894155568 | NFIB//IGF1//FGF10 |
| GO:2000257 | regulation of protein activation cascade | Biological process | 3 | 20 | 428 | 14747 | 5.16834112149533 | 0.0191734850179483 | 0.160418157983501 | 1.71729894155568 | A2M//C3//CFH |
| GO:0009743 | response to carbohydrate stimulus | Biological process | 9 | 138 | 428 | 14747 | 2.24710483543275 | 0.0192578026628824 | 0.16084143765199 | 1.71539326787742 | NNAT//TGFBR2//GPAM//COLEC12//CALCRL//CMA1//OXCT1//VCAM1//CST3 |
| GO:0045834 | positive regulation of lipid metabolic process | Biological process | 7 | 94 | 428 | 14747 | 2.56584310996222 | 0.019460886338522 | 0.162253438720999 | 1.71083738384345 | SORBS1//ANXA1//ABCD2//FGF2//KIT//CCL21//TEK |
| GO:0001503 | ossification | Biological process | 15 | 285 | 428 | 14747 | 1.81345302508608 | 0.0195562093557013 | 0.162763634236195 | 1.70871532211698 | MEF2C//SFRP1//MN1//PTHLH//PBX1//PTN//MGP//OSR1//IGF1//FGF2//IL6ST//FOXC1//CHRDL2//CHRDL1//IGSF10 |
| GO:0048469 | cell maturation | Biological process | 8 | 116 | 428 | 14747 | 2.3762487914921 | 0.0197136061265768 | 0.163716597342316 | 1.70523392480109 | EDNRB//CLN5//NR4A2//EPAS1//FGFR1//KCNMA1//KDR//CCL21 |
| GO:0031281 | positive regulation of cyclase activity | Biological process | 4 | 36 | 428 | 14747 | 3.82840083073728 | 0.0197999268138785 | 0.163716597342316 | 1.70333641501056 | GNAL//NTRK2//CAP2//VIP |
| GO:0048008 | platelet-derived growth factor receptor signaling pathway | Biological process | 4 | 36 | 428 | 14747 | 3.82840083073728 | 0.0197999268138785 | 0.163716597342316 | 1.70333641501056 | APOD//PIK3C2A//TIPARP//ARID5B |
| GO:0055074 | calcium ion homeostasis | Biological process | 14 | 260 | 428 | 14747 | 1.8553019410496 | 0.0198360234639734 | 0.163716597342316 | 1.70254538665497 | CASQ2//ANK2//CACNB4//CAV1//CXCL12//BDKRB1//LPAR1//EDNRB//DMD//PLN//FGF2//CCL21//TRPC1//KDR |
| GO:0042310 | vasoconstriction | Biological process | 5 | 54 | 428 | 14747 | 3.1903340256144 | 0.0198423554757515 | 0.163716597342316 | 1.70240677424834 | EDNRB//ACTA2//PIK3C2A//KCNMB4//CAV1 |
| GO:0006195 | purine nucleotide catabolic process | Biological process | 22 | 472 | 428 | 14747 | 1.60598170441945 | 0.0199944753396833 | 0.16468679256468 | 1.69908998738292 | GNAL//GNG11//RHOJ//PDE8B//PDE7B//ACTC1//NTRK2//BVES//ALDH1A1//DLC1//ERRFI1//CNN3//PLN//FGF10//SFRP1//PREX2//CCL21//SPRY1//ARHGAP6//TPM2//RGS5//RGS22 |
| GO:0016049 | cell growth | Biological process | 18 | 365 | 428 | 14747 | 1.6991806426834 | 0.0204139310435493 | 0.167562886655227 | 1.69007335657089 | IGFBP4//FBLN5//MYOCD//SFRP1//TAF9B//PPP1R1C//BDKRB1//CRYAB//FGF2//FRZB//SLIT3//SLIT2//WFDC1//TSPYL2//MAP1B//DCLK1//EMP1//TGFBR3 |
| GO:0071417 | cellular response to organic nitrogen | Biological process | 18 | 365 | 428 | 14747 | 1.6991806426834 | 0.0204139310435493 | 0.167562886655227 | 1.69007335657089 | FGF2//FGF7//FGF10//FGFR1//PDK4//PIK3C2A//PIK3R1//SORBS1//CAB39L//PDGFD//GHR//CASQ2//AQP1//GNG7//GNG11//PRKAR2B//SFRP1//SLIT2 |
| GO:0046546 | development of primary male sexual characteristics | Biological process | 8 | 117 | 428 | 14747 | 2.3559389727614 | 0.0206450677240884 | 0.169168948412676 | 1.68518368812715 | GFRA1//KIT//SFRP1//ARID5B//TEX15//FGF10//TCF21//CST3 |
| GO:1901699 | cellular response to nitrogen compound | Biological process | 19 | 393 | 428 | 14747 | 1.66579272787805 | 0.0210591878476884 | 0.172266323920456 | 1.67655838149187 | FGF2//FGF7//FGF10//FGFR1//PDK4//PIK3C2A//PIK3R1//SORBS1//CAB39L//PDGFD//GHR//MEF2C//CASQ2//AQP1//GNG7//GNG11//PRKAR2B//SFRP1//SLIT2 |
| GO:0071840 | cellular component organization or biogenesis | Biological process | 144 | 4290 | 428 | 14747 | 1.1565518593556 | 0.0211501885300397 | 0.172714467636574 | 1.67468575702695 | TACC1//WEE1//IGFBP4//FBLN5//MYOCD//CAPN6//MAP1B//MYH10//CLU//TGFBR3//SRPX//HEG1//ANGPT1//TSPYL2//TSPYL5//DPYSL3//OPHN1//TNXB//DLC1//FERMT2//DAAM2//RHOJ//DES//SGCD//CAP2//WASF3//ZRANB1//SH3D19//SFRP1//MPP6//BMX//EPAS1//CRYAB//CLN5//TNS1//CETN2//PPP1R12A//TTN//TEX15//FMOD//ANK2//CACNB4//CNTN1//FGFR1//GFRA1//ITGA9//LAMA2//MYH11//PRNP//SLIT3//TRPC1//SLIT2//MYL9//BOC//NLGN1//MUSK//CXCL12//KDR//KIT//BVES//PALMD//PARVA//PLSCR4//ANO6//ARHGAP6//DMD//NME5//CST3//NTRK2//LPAR1//PMP22//APOD//FOXC1//CAV1//ZDHHC15//EYA4//NR4A2//DCLK1//NFIB//CMA1//CTSG//ITGA8//COL19A1//ECM2//FBLN1//VIT//TLL1//COL14A1//RECK//GLT25D2//ABI3BP//SMOC2//CRISPLD2//CCDC80//DPT//MEF2C//TMOD1//ACTC1//TAF9B//PPP1R1C//BDKRB1//FGF2//FRZB//WFDC1//CCL21//EPB41L2//AQP1//CNN1//CNN3//PRICKLE2//ANXA1//FGF7//FGF10//VDAC2//SELP//CDH5//FLNC//CADM3//CADM2//PLA2G2A//LIPG//TEK//FZD4//TRIM9//KCNMA1//COLEC12//KCTD12//SORBS1//MFAP5//LDB2//SYNM//IGF1//PIK3C2A//C3//PTX3//PTPRD//GPX3//NEXN//CD36//PMP2//TOR1AIP1//CD34//CPE//PIK3R1 |
| GO:0001558 | regulation of cell growth | Biological process | 15 | 288 | 428 | 14747 | 1.7945628894081 | 0.0212592415753822 | 0.173308244569227 | 1.672452233016 | MYOCD//SFRP1//TAF9B//PPP1R1C//BDKRB1//CRYAB//FGF2//FRZB//SLIT3//SLIT2//WFDC1//TSPYL2//MAP1B//IGFBP4//FBLN5 |
| GO:0021954 | central nervous system neuron development | Biological process | 5 | 55 | 428 | 14747 | 3.13232795242141 | 0.0213234734807012 | 0.173535230425707 | 1.6711420496159 | NR4A2//DCLK1//NFIB//SLIT2//NTRK2 |
| GO:0002053 | positive regulation of mesenchymal cell proliferation | Biological process | 4 | 37 | 428 | 14747 | 3.72493053801465 | 0.0217056258130114 | 0.17376009584008 | 1.66342768813814 | FGFR1//KDR//TGFBR2//FOXP2 |
| GO:0003156 | regulation of organ formation | Biological process | 4 | 37 | 428 | 14747 | 3.72493053801465 | 0.0217056258130114 | 0.17376009584008 | 1.66342768813814 | FGF2//FGF10//FGFR1//SPRY1 |
| GO:0030199 | collagen fibril organization | Biological process | 4 | 37 | 428 | 14747 | 3.72493053801465 | 0.0217056258130114 | 0.17376009584008 | 1.66342768813814 | DPT//FOXC1//TNXB//COL14A1 |
| GO:0050931 | pigment cell differentiation | Biological process | 4 | 37 | 428 | 14747 | 3.72493053801465 | 0.0217056258130114 | 0.17376009584008 | 1.66342768813814 | EDNRB//KIT//MEF2C//ADAMTS9 |
| GO:0055013 | cardiac muscle cell development | Biological process | 4 | 37 | 428 | 14747 | 3.72493053801465 | 0.0217056258130114 | 0.17376009584008 | 1.66342768813814 | ACTC1//MYH10//TTN//MYOCD |
| GO:0072523 | purine-containing compound catabolic process | Biological process | 22 | 476 | 428 | 14747 | 1.5924860598445 | 0.0217601832361628 | 0.17376009584008 | 1.66233745189117 | GNAL//GNG11//RHOJ//PDE8B//PDE7B//ACTC1//NTRK2//BVES//ALDH1A1//DLC1//ERRFI1//CNN3//PLN//FGF10//SFRP1//PREX2//CCL21//SPRY1//ARHGAP6//TPM2//RGS5//RGS22 |
| GO:0048863 | stem cell differentiation | Biological process | 9 | 141 | 428 | 14747 | 2.19929409425333 | 0.0218126064962124 | 0.17376009584008 | 1.66129243532519 | CDON//IGF1//KIT//FGF10//SFRP1//LDB2//FGF2//A2M//OSR1 |
| GO:0010627 | regulation of intracellular protein kinase cascade | Biological process | 30 | 701 | 428 | 14747 | 1.47456237417841 | 0.0218170981304232 | 0.17376009584008 | 1.66120301496463 | FGF2//LPAR1//FGF10//GHR//KIT//CAV1//FCER1A//AKAP12//TEK//NLGN1//IL6ST//IGF1//CD36//CCL21//SLC44A2//FGFR1//SPRY1//IGFBP4//KDR//NTRK2//CDON//TNXB//FZD4//SFRP1//HIPK3//EDA2R//ANGPT1//ITSN1//TSPYL5//CD44 |
| GO:0001516 | prostaglandin biosynthetic process | Biological process | 3 | 21 | 428 | 14747 | 4.92222963951936 | 0.0218976342209872 | 0.17376009584008 | 1.65960280298044 | ANXA1//PTGDS//PTGIS |
| GO:0030511 | positive regulation of transforming growth factor beta receptor signaling pathway | Biological process | 3 | 21 | 428 | 14747 | 4.92222963951936 | 0.0218976342209872 | 0.17376009584008 | 1.65960280298044 | TGFBR3//ITGA8//MYOCD |
| GO:0043276 | anoikis | Biological process | 3 | 21 | 428 | 14747 | 4.92222963951936 | 0.0218976342209872 | 0.17376009584008 | 1.65960280298044 | CAV1//NTRK2//PDK4 |
| GO:0043567 | regulation of insulin-like growth factor receptor signaling pathway | Biological process | 3 | 21 | 428 | 14747 | 4.92222963951936 | 0.0218976342209872 | 0.17376009584008 | 1.65960280298044 | IGF1//IGFBP4//CILP |
| GO:0045661 | regulation of myoblast differentiation | Biological process | 3 | 21 | 428 | 14747 | 4.92222963951936 | 0.0218976342209872 | 0.17376009584008 | 1.65960280298044 | MEF2C//BOC//MYOCD |
| GO:0046457 | prostanoid biosynthetic process | Biological process | 3 | 21 | 428 | 14747 | 4.92222963951936 | 0.0218976342209872 | 0.17376009584008 | 1.65960280298044 | PTGDS//PTGIS//ANXA1 |
| GO:0048512 | circadian behavior | Biological process | 3 | 21 | 428 | 14747 | 4.92222963951936 | 0.0218976342209872 | 0.17376009584008 | 1.65960280298044 | CST3//PTGDS//KCNMA1 |
| GO:0030003 | cellular cation homeostasis | Biological process | 18 | 369 | 428 | 14747 | 1.68076134032368 | 0.0224829632168234 | 0.178108391330616 | 1.6481464500467 | CASQ2//ANK2//CACNB4//CAV1//CXCL12//PRNP//CYBRD1//CLN5//BDKRB1//LPAR1//EDNRB//DMD//PLN//SLC22A17//KCNMA1//FGF2//CCL21//TRPC1 |
| GO:0042326 | negative regulation of phosphorylation | Biological process | 12 | 214 | 428 | 14747 | 1.93209013887676 | 0.022539611805526 | 0.17826104262115 | 1.64705356797801 | CAV1//BDKRB1//PRNP//PPAP2B//SLIT2//ERRFI1//FABP4//DMD//SPRY1//SFRP1//HIPK3//MYOCD |
| GO:0006813 | potassium ion transport | Biological process | 8 | 119 | 428 | 14747 | 2.31634335977382 | 0.0226009715719873 | 0.178450386468224 | 1.6458728909728 | CASQ2//VIP//ABCC9//ANK2//AQP1//KCNMA1//KCNH1//KCNMB4 |
| GO:0050671 | positive regulation of lymphocyte proliferation | Biological process | 7 | 97 | 428 | 14747 | 2.48648713748916 | 0.0227058253340362 | 0.178686602339965 | 1.64386270730615 | MEF2C//NFATC2//IL6ST//VCAM1//IGF1//GPAM//FGF10 |
| GO:0060348 | bone development | Biological process | 7 | 97 | 428 | 14747 | 2.48648713748916 | 0.0227058253340362 | 0.178686602339965 | 1.64386270730615 | MEF2C//PTHLH//IGF1//GHR//FREM1//SMAD9//PAPSS2 |
| GO:0042981 | regulation of apoptotic process | Biological process | 47 | 1205 | 428 | 14747 | 1.34391166091441 | 0.0228411928265407 | 0.179078147071078 | 1.64128121984573 | CLU//PLAGL1//DLC1//ANGPT1//ANXA1//AQP1//CD44//CRYAB//EDNRB//FGF10//FGFR1//FOXC1//IGF1//IL6ST//KDR//MEF2C//PIK3R1//PRNP//PTGFR//CXCL12//SFRP1//TEK//VIP//SCG2//NME5//HIPK3//FAIM2//TAF9B//OSR1//MYOCD//LPAR1//FRZB//KCNMA1//PTGIS//SRPX//SLIT2//CNTFR//NTRK2//NR4A2//ITSN1//MUSK//GPAM//CAV1//CCL21//PDK4//VDAC2//ZNF738 |
| GO:0003158 | endothelium development | Biological process | 5 | 56 | 428 | 14747 | 3.0763935246996 | 0.0228747699107906 | 0.179078147071078 | 1.64064326565393 | HEG1//DMD//KDR//APOLD1//CD34 |
| GO:0030204 | chondroitin sulfate metabolic process | Biological process | 5 | 56 | 428 | 14747 | 3.0763935246996 | 0.0228747699107906 | 0.179078147071078 | 1.64064326565393 | MAMDC2//GPC5//GPC3//GPC6//UST |
| GO:0022037 | metencephalon development | Biological process | 6 | 76 | 428 | 14747 | 2.72017953762912 | 0.0229057810260763 | 0.179078147071078 | 1.64005489535123 | CNTN1//PTPRS//FAIM2//FOXP2//MYH10//FZD4 |
| GO:0043583 | ear development | Biological process | 11 | 190 | 428 | 14747 | 1.99479832759469 | 0.0229906971429813 | 0.179154631821696 | 1.63844785949368 | FGF10//FGFR1//ITGA8//SOBP//EYA4//OSR1//KCNMA1//IGF1//LPPR4//FRZB//RDH10 |
| GO:0072330 | monocarboxylic acid biosynthetic process | Biological process | 11 | 190 | 428 | 14747 | 1.99479832759469 | 0.0229906971429813 | 0.179154631821696 | 1.63844785949368 | PTGDS//PTGIS//RDH10//CH25H//PTPLA//C5ORF4//SCD5//FCER1A//UST//ANXA1//PDK4 |
| GO:0030030 | cell projection organization | Biological process | 38 | 938 | 428 | 14747 | 1.39585616643086 | 0.0233154471562095 | 0.181388853977101 | 1.63235625099602 | MYH10//OPHN1//ANK2//CACNB4//CNTN1//DPYSL3//FGFR1//GFRA1//ITGA9//LAMA2//MYH11//PRNP//SLIT3//TRPC1//SLIT2//MYL9//CAP2//BOC//CXCL12//DMD//NME5//NTRK2//LPAR1//PMP22//APOD//MAP1B//NR4A2//DCLK1//NFIB//KIT//WASF3//PRICKLE2//CCL21//WEE1//PTPRD//NLGN1//PARVA//ITGA8 |
| GO:0050767 | regulation of neurogenesis | Biological process | 19 | 398 | 428 | 14747 | 1.64486568355798 | 0.023628746658942 | 0.183331325934791 | 1.62655931406553 | CNTN1//DMD//DPYSL3//FGFR1//NTRK2//LPAR1//PMP22//EDNRB//NLGN1//PBX1//MEF2C//ZEB1//CDON//MAP1B//IL6ST//ASPA//SERPINF1//SLIT2//PTPRD |
| GO:0031128 | developmental induction | Biological process | 4 | 38 | 428 | 14747 | 3.62690605017216 | 0.0237196982494485 | 0.183331325934791 | 1.6248908401574 | FGF2//FGF10//FGFR1//SPRY1 |
| GO:0051349 | positive regulation of lyase activity | Biological process | 4 | 38 | 428 | 14747 | 3.62690605017216 | 0.0237196982494485 | 0.183331325934791 | 1.6248908401574 | GNAL//NTRK2//CAP2//VIP |
| GO:0070542 | response to fatty acid | Biological process | 4 | 38 | 428 | 14747 | 3.62690605017216 | 0.0237196982494485 | 0.183331325934791 | 1.6248908401574 | CCL21//TGFBR3//SFRP1//PDK4 |
| GO:0042327 | positive regulation of phosphorylation | Biological process | 26 | 592 | 428 | 14747 | 1.51325303106845 | 0.0237573410416651 | 0.183331325934791 | 1.62420216788074 | FGF2//LPAR1//FGF10//GHR//KIT//C3//EDNRB//MUSK//TEK//CDON//TSPYL2//ANGPT1//FCER1A//TGFBR2//CAV1//CD44//PRKAR2B//IL6ST//IGF1//FGFR1//FZD4//SPDYA//CCL21//CD36//FGF7//PPAP2B |
| GO:0032946 | positive regulation of mononuclear cell proliferation | Biological process | 7 | 98 | 428 | 14747 | 2.46111481975968 | 0.0238657661480386 | 0.183870498804517 | 1.62222461918482 | MEF2C//NFATC2//IL6ST//VCAM1//IGF1//GPAM//FGF10 |
| GO:0050867 | positive regulation of cell activation | Biological process | 13 | 241 | 428 | 14747 | 1.85860123317951 | 0.0239254754707145 | 0.184033213741673 | 1.62113942273583 | SELP//MEF2C//NFATC2//CAV1//PIK3R1//CCL21//IL6ST//VCAM1//IGF1//GPAM//FCER1A//FGF10//TGFBR2 |
| GO:0050890 | cognition | Biological process | 10 | 167 | 428 | 14747 | 2.06321002854105 | 0.0239642181222447 | 0.184034390056337 | 1.62043673611655 | MEF2C//PRNP//VIP//TMOD2//NTRK2//PRKAR2B//PTN//MUSK//ITGA8//FOXP2 |
| GO:0055067 | monovalent inorganic cation homeostasis | Biological process | 6 | 77 | 428 | 14747 | 2.68485253064692 | 0.0242559865433618 | 0.185975562420084 | 1.61518105698411 | CORIN//EDNRB//PDK4//SLC9A9//CLN5//KCNMA1 |
| GO:0007243 | intracellular protein kinase cascade | Biological process | 36 | 882 | 428 | 14747 | 1.40635132557696 | 0.0243317564784436 | 0.186257057216208 | 1.6138265387732 | CAV1//FGFR1//MEF2C//MAPK4//SCG2//DOK5//FGF2//LPAR1//FGF10//GHR//KIT//FCER1A//AKAP12//TEK//NLGN1//IL6ST//IGF1//CD36//CCL21//SLC44A2//SPRY1//IGFBP4//KDR//NTRK2//CDON//TNXB//FZD4//SFRP1//HIPK3//EDA2R//CRYAB//ANGPT1//ITSN1//TSPYL5//CD44//TGFBR3 |
| GO:0002027 | regulation of heart rate | Biological process | 5 | 57 | 428 | 14747 | 3.0224217084768 | 0.0244972942428159 | 0.186924153990382 | 1.6108818813623 | PLN//ANK2//CASQ2//DMD//EPAS1 |
| GO:0007260 | tyrosine phosphorylation of STAT protein | Biological process | 5 | 57 | 428 | 14747 | 3.0224217084768 | 0.0244972942428159 | 0.186924153990382 | 1.6108818813623 | IL6ST//KIT//GHR//IGF1//CAV1 |
| GO:0008016 | regulation of heart contraction | Biological process | 8 | 121 | 428 | 14747 | 2.27805669267012 | 0.0246840218037815 | 0.187213706255824 | 1.60758407863839 | PLN//ANK2//CASQ2//DMD//EPAS1//CAV1//CHRM2//DES |
| GO:0008610 | lipid biosynthetic process | Biological process | 25 | 566 | 428 | 14747 | 1.52189078960404 | 0.0247480235899341 | 0.187213706255824 | 1.60645947868027 | PTGDS//PTGIS//RDH10//DPM1//CH25H//PTPLA//C5ORF4//SCD5//PLA2G2A//GPAM//PEMT//SLC44A2//FGF2//PIK3C2A//PIK3R1//UGCG//PBX1//GLT25D2//C3//SORBS1//FCER1A//PPAP2A//PPAP2B//ANXA1//PDK4 |
| GO:0001945 | lymph vessel development | Biological process | 3 | 22 | 428 | 14747 | 4.69849192863212 | 0.0248216397851016 | 0.187213706255824 | 1.60516953121371 | FOXC1//KDR//HEG1 |
| GO:0010632 | regulation of epithelial cell migration | Biological process | 3 | 22 | 428 | 14747 | 4.69849192863212 | 0.0248216397851016 | 0.187213706255824 | 1.60516953121371 | TGFBR3//FGF10//TGFBR2 |
| GO:0051491 | positive regulation of filopodium assembly | Biological process | 3 | 22 | 428 | 14747 | 4.69849192863212 | 0.0248216397851016 | 0.187213706255824 | 1.60516953121371 | DPYSL3//CCL21//NLGN1 |
| GO:0060765 | regulation of androgen receptor signaling pathway | Biological process | 3 | 22 | 428 | 14747 | 4.69849192863212 | 0.0248216397851016 | 0.187213706255824 | 1.60516953121371 | IGF1//SFRP1//TCF21 |
| GO:0061384 | heart trabecula morphogenesis | Biological process | 3 | 22 | 428 | 14747 | 4.69849192863212 | 0.0248216397851016 | 0.187213706255824 | 1.60516953121371 | TEK//TGFBR3//ADAMTS1 |
| GO:0035023 | regulation of Rho protein signal transduction | Biological process | 10 | 168 | 428 | 14747 | 2.0509290164664 | 0.0248492925267219 | 0.187213706255824 | 1.60468597136182 | NTRK2//BVES//DLC1//ERRFI1//SFRP1//PREX2//CCL21//LPAR1//ITSN1//ARHGEF26 |
| GO:0007431 | salivary gland development | Biological process | 4 | 39 | 428 | 14747 | 3.5339084591421 | 0.0258433716868409 | 0.193784653419095 | 1.587650826224 | FGF7//FGFR1//FGF10//CST3 |
| GO:0035019 | somatic stem cell maintenance | Biological process | 4 | 39 | 428 | 14747 | 3.5339084591421 | 0.0258433716868409 | 0.193784653419095 | 1.587650826224 | IGF1//FGF10//SFRP1//LDB2 |
| GO:0045168 | cell-cell signaling involved in cell fate commitment | Biological process | 4 | 39 | 428 | 14747 | 3.5339084591421 | 0.0258433716868409 | 0.193784653419095 | 1.587650826224 | FGF2//FGF10//FGFR1//SPRY1 |
| GO:0010976 | positive regulation of neuron projection development | Biological process | 5 | 58 | 428 | 14747 | 2.97031098936513 | 0.0261920263989038 | 0.196090696854587 | 1.58183090020716 | CNTN1//DMD//DPYSL3//FGFR1//NTRK2 |
| GO:0090287 | regulation of cellular response to growth factor stimulus | Biological process | 9 | 146 | 428 | 14747 | 2.12397580335424 | 0.0265972500135644 | 0.198812359427412 | 1.57516326433412 | TGFBR3//ITGA8//MYOCD//TGFBR2//FZD4//FGF10//SPRY1//SLIT2//DMD |
| GO:0051049 | regulation of transport | Biological process | 40 | 1007 | 428 | 14747 | 1.3686437925178 | 0.0267464380347449 | 0.199614652562908 | 1.57273404709538 | ANGPT1//CORIN//EDNRB//CASQ2//VIP//PRKAR2B//AQP1//CAV1//CNTN1//C3//DMD//ANK2//PLN//IGF1//PIK3R1//SORBS1//OPHN1//ANXA1//NNAT//OXCT1//LIPG//CRYAB//MAP1B//TRIM9//APOD//CD36//FCER1A//SFRP1//PDE8B//PPP1R12A//FGF10//NTRK2//KCNMB4//CCL21//GPAM//PTX3//BDKRB1//TRPC1//CD34//NLGN1 |
| GO:0007267 | cell-cell signaling | Biological process | 43 | 1097 | 428 | 14747 | 1.35058443162746 | 0.0268322524077852 | 0.199942205832387 | 1.57134286936259 | FGF2//FGF10//FGFR1//SPRY1//FCER1A//NLGN1//CACNB4//GNAL//GNG7//KCNH1//KCNMA1//PMP22//ABCC9//PDE7B//KCNMB4//KCNIP1//NCALD//TMOD2//BCHE//PRKAR2B//MUSK//NTRK2//TRIM9//ZDHHC15//CD34//ANXA1//NNAT//OXCT1//LAMA2//FZD4//SFRP1//DMD//PDE8B//OPHN1//ITSN1//MEF2C//FGF7//ANK2//CASQ2//SH2D1A//PTHLH//CCL21//TEK |
| GO:0018193 | peptidyl-amino acid modification | Biological process | 28 | 656 | 428 | 14747 | 1.47066617278322 | 0.0269322552985425 | 0.2003742987812 | 1.56972727738311 | ERRFI1//TGFBR2//HIPK3//EFEMP1//FGFR1//KDR//KIT//TEK//WEE1//MAN1A1//DPM1//UBE2J1//MAMDC2//CAV1//DMD//ANGPT1//CD44//MYOCD//IL6ST//GHR//IGF1//PDGFD//CD36//FCER1A//FGF7//FGF10//PPAP2B//SFRP1 |
| GO:0032268 | regulation of cellular protein metabolic process | Biological process | 49 | 1279 | 428 | 14747 | 1.32003500105953 | 0.0270550336554925 | 0.20097422975552 | 1.56775192148429 | CCNC//DIRAS3//FGF2//LPAR1//FGF10//GHR//KIT//CAV1//PSTK//BDKRB1//PRNP//PPAP2B//SLIT2//C3//EDNRB//MUSK//TEK//CDON//ERRFI1//LARP6//FABP4//TSPYL2//ANGPT1//FCER1A//TSPYL5//TGFBR2//CLU//RNF180//DMD//CD44//PRKAR2B//MYOCD//DLC1//IL6ST//IGF1//FGFR1//SPRY1//TNXB//FZD4//SFRP1//HIPK3//SPDYA//TTN//CCL21//PDGFD//CD36//FGF7//SH3D19//TIMP3 |
| GO:0070665 | positive regulation of leukocyte proliferation | Biological process | 7 | 101 | 428 | 14747 | 2.38801239937078 | 0.027588677981614 | 0.204619603879187 | 1.5592691099248 | MEF2C//NFATC2//IL6ST//VCAM1//IGF1//GPAM//FGF10 |
| GO:2000027 | regulation of organ morphogenesis | Biological process | 9 | 147 | 428 | 14747 | 2.10952698836544 | 0.0276365110282262 | 0.204656088654675 | 1.55851678535105 | FGF2//FGF10//FGFR1//SPRY1//CD34//FGF7//FOXP2//SFRP1//NFIB |
| GO:0007622 | rhythmic behavior | Biological process | 3 | 23 | 428 | 14747 | 4.4942096708655 | 0.0279452124522336 | 0.206301421338548 | 1.55369258433106 | CST3//PTGDS//KCNMA1 |
| GO:0042474 | middle ear morphogenesis | Biological process | 3 | 23 | 428 | 14747 | 4.4942096708655 | 0.0279452124522336 | 0.206301421338548 | 1.55369258433106 | EYA4//FGFR1//OSR1 |
| GO:0007566 | embryo implantation | Biological process | 4 | 40 | 428 | 14747 | 3.44556074766355 | 0.028077695229223 | 0.206542755275669 | 1.55163854434533 | CST3//FBLN1//TGFBR2//RECK |
| GO:0055006 | cardiac cell development | Biological process | 4 | 40 | 428 | 14747 | 3.44556074766355 | 0.028077695229223 | 0.206542755275669 | 1.55163854434533 | ACTC1//MYH10//TTN//MYOCD |
| GO:0006875 | cellular metal ion homeostasis | Biological process | 16 | 325 | 428 | 14747 | 1.69627606038821 | 0.028151140895195 | 0.206542755275669 | 1.55050399958894 | CASQ2//ANK2//CACNB4//CAV1//CXCL12//CYBRD1//BDKRB1//LPAR1//EDNRB//DMD//PLN//SLC22A17//KCNMA1//FGF2//CCL21//TRPC1 |
| GO:0045664 | regulation of neuron differentiation | Biological process | 16 | 325 | 428 | 14747 | 1.69627606038821 | 0.028151140895195 | 0.206542755275669 | 1.55050399958894 | CNTN1//DMD//DPYSL3//FGFR1//NTRK2//LPAR1//PMP22//EDNRB//PBX1//MEF2C//ZEB1//CDON//MAP1B//SLIT2//PTPRD//NLGN1 |
| GO:0009166 | nucleotide catabolic process | Biological process | 22 | 489 | 428 | 14747 | 1.55015002962368 | 0.0283429609394844 | 0.207630692350847 | 1.54755478173471 | GNAL//GNG11//RHOJ//PDE8B//PDE7B//ACTC1//NTRK2//BVES//ALDH1A1//DLC1//ERRFI1//CNN3//PLN//FGF10//SFRP1//PREX2//CCL21//SPRY1//ARHGAP6//TPM2//RGS5//RGS22 |
| GO:0030855 | epithelial cell differentiation | Biological process | 15 | 299 | 428 | 14747 | 1.72854218110212 | 0.0284745920465124 | 0.208275045199107 | 1.54554248931094 | HEG1//DMD//ANXA1//CAV1//OSR1//KDR//APOLD1//ERRFI1//NFIB//IGF1//FGF10//FGF2//MEF2C//PTHLH//TCF21 |
| GO:0002682 | regulation of immune system process | Biological process | 36 | 893 | 428 | 14747 | 1.3890278490021 | 0.0286022053197511 | 0.208888081424032 | 1.5436004801131 | C3//FCER1A//A2M//COLEC12//MEF2C//CCL21//TGFBR2//BDKRB1//SELP//SLIT2//IL6ST//C7//CLU//CFD//CFH//CACNB4//PIK3R1//NFATC2//CAV1//ANXA1//KIT//VCAM1//IGF1//GPAM//SFRP1//SH2D1A//PRNP//FGF10//CD34//CD200//KLRG1//CD160//CTSG//CD36//CXCL12//APOD |
| GO:0043087 | regulation of GTPase activity | Biological process | 12 | 222 | 428 | 14747 | 1.86246526900733 | 0.0288963268463282 | 0.210491963835713 | 1.53915735903429 | NTRK2//BVES//ALDH1A1//DLC1//ERRFI1//SFRP1//PREX2//CCL21//SPRY1//RGS5//RGS22//ARHGAP6 |
| GO:0014070 | response to organic cyclic compound | Biological process | 23 | 518 | 428 | 14747 | 1.52988218525602 | 0.0289100935861589 | 0.210491963835713 | 1.53895050245295 | GNAL//ANXA1//CRYAB//CST3//FGF10//GHR//WFDC1//CAV1//CD36//MEF2C//RCAN1//TGFBR2//PTGDS//SLIT3//SLIT2//SFRP1//CASQ2//AQP1//SMAD9//PRKAR2B//EDNRB//EPHX1//TAF9B |
| GO:0009893 | positive regulation of metabolic process | Biological process | 73 | 2030 | 428 | 14747 | 1.2390440127066 | 0.0290896609380993 | 0.211476513740542 | 1.53626134075262 | FGF2//LPAR1//FGF10//GHR//KIT//BVES//C3//EDNRB//MUSK//TEK//CDON//ANGPT1//MYOCD//TSPYL2//GNAL//NTRK2//CAP2//FCER1A//CCL21//VIP//ANK2//CD34//CD44//CNTN1//MEF2C//OSR1//CD36//TGFBR2//SORBS1//EBF1//FOXC1//IGF1//SMAD9//NFATC2//RORB//SFRP1//FZD4//EBF3//PTHLH//AKAP12//CALCRL//ANXA1//TSPYL5//ABCD2//CLU//RNF180//CAV1//PRKAR2B//DLC1//IL6ST//FGFR1//PTX3//SPDYA//CST3//CCNC//EPAS1//PPP1R12A//NFIB//NFIX//NR4A2//PBX1//PIK3R1//PLAGL1//TCF21//LDB2//FHL5//BEX1//ZNF462//FGF7//PPAP2B//SH3D19//KDR//ITGA8 |
| GO:0030336 | negative regulation of cell migration | Biological process | 8 | 125 | 428 | 14747 | 2.20515887850467 | 0.0292460821294537 | 0.212290054300403 | 1.53393230474498 | SLIT2//TGFBR3//FGF2//APOD//DPYSL3//SFRP1//DLC1//PODN |
| GO:0006874 | cellular calcium ion homeostasis | Biological process | 13 | 248 | 428 | 14747 | 1.80614071450106 | 0.0293018073424203 | 0.212371305799396 | 1.53310559143318 | CASQ2//BDKRB1//LPAR1//EDNRB//CAV1//DMD//ANK2//PLN//FGF2//CCL21//TRPC1//CACNB4//CXCL12 |
| GO:0044093 | positive regulation of molecular function | Biological process | 43 | 1104 | 428 | 14747 | 1.34202094338345 | 0.0294021124508566 | 0.212774923032071 | 1.53162146575137 | FGF2//LPAR1//FGF10//GHR//KIT//DLC1//TSPYL2//ANGPT1//GNAL//NTRK2//CAP2//ARHGAP6//PDE1A//PRKAR2B//FCER1A//FGFR1//VIP//MEF2C//TGFBR2//ALDH1A1//ERRFI1//SFRP1//PREX2//CCL21//NR4A2//IGF1//MYOCD//FZD4//RGS5//RGS22//TEK//SPDYA//CAV1//PPAP2B//BEX1//ARID5B//CLU//TGFBR3//EDA2R//ANKRD42//PARM1//ANK2//DMD |
| GO:0050920 | regulation of chemotaxis | Biological process | 7 | 103 | 428 | 14747 | 2.34164322656746 | 0.0302786567265624 | 0.218786233225721 | 1.51886339565749 | SLIT2//CCL21//KDR//FGF10//CXCL12//SCG2//LPAR1 |
| GO:0048519 | negative regulation of biological process | Biological process | 109 | 3189 | 428 | 14747 | 1.17769244746104 | 0.0304106384832817 | 0.219168991866513 | 1.51697446157717 | WEE1//CAV1//CD36//EDNRB//FGFR1//FOSB//MEF2C//NFIB//NFIX//ZEB1//TCF21//NR2F1//KANK2//TAF9B//MYOCD//OSR1//A2M//BDKRB1//PRNP//PPAP2B//SLIT2//SCG2//PIK3R1//ANXA1//ERRFI1//CASQ2//VIP//FABP4//PLAGL1//CAB39L//ANGPT1//JAM2//TSPYL2//AQP1//CD44//CLU//CRYAB//FGF10//FOXC1//IGF1//IL6ST//KDR//PTGFR//CXCL12//SFRP1//TEK//NME5//HIPK3//FAIM2//CDH5//DPT//FGF2//FRZB//PMP22//PTHLH//SLIT3//PPAP2A//ADAMTS1//SPRY1//DLC1//TENC1//PODN//PLN//CD34//TGFBR3//APOD//CST3//FZD4//DMD//ANK2//DPYSL3//LPAR1//BAI3//SERPINF1//ARID5B//FOXP2//KIT//APCDD1//WFDC1//TGFBR2//VDAC2//EFEMP1//MAP1B//CTSG//LDB2//TRIM9//RGS5//RGS22//CNTFR//NTRK2//NR4A2//ITSN1//CILP//PTGIS//PBX1//MT1A//PDE8B//OGN//CALCRL//TIMP3//RCAN1//CCL21//TRPC1//ARHGAP6//KCNMA1//SRPX//GPAM//GHR//PDK4 |
| GO:0045428 | regulation of nitric oxide biosynthetic process | Biological process | 4 | 41 | 428 | 14747 | 3.36152268064737 | 0.0304235421714472 | 0.219168991866513 | 1.51679022302608 | CAV1//CD34//PTGIS//PTX3 |
| GO:0006959 | humoral immune response | Biological process | 8 | 126 | 428 | 14747 | 2.18765761756416 | 0.0304715968409018 | 0.219184080443832 | 1.51610478630832 | A2M//C3//C7//CLU//CFD//CFH//SH2D1A//MEF2C |
| GO:0033124 | regulation of GTP catabolic process | Biological process | 12 | 224 | 428 | 14747 | 1.84583611481976 | 0.0306701549621401 | 0.220280073816937 | 1.51328402972236 | NTRK2//BVES//ALDH1A1//DLC1//ERRFI1//SFRP1//PREX2//CCL21//SPRY1//ARHGAP6//RGS5//RGS22 |
| GO:0051338 | regulation of transferase activity | Biological process | 28 | 664 | 428 | 14747 | 1.45294730323162 | 0.0308113709752204 | 0.220961546136581 | 1.51128897699217 | CCNC//DIRAS3//FGF2//LPAR1//FGF10//GHR//KIT//CAV1//ERRFI1//FABP4//TSPYL2//ANGPT1//FCER1A//TGFBR2//PRKAR2B//FGFR1//SPRY1//TNXB//FZD4//SFRP1//HIPK3//PIK3R1//CCL21//TEK//MYOCD//SPDYA//TTN//PARM1 |
| GO:0060326 | cell chemotaxis | Biological process | 9 | 150 | 428 | 14747 | 2.06733644859813 | 0.0309252788870611 | 0.221445427946538 | 1.50968637517287 | CCL21//KIT//SLIT2//ITGA9//SCG2//EDNRB//PARVA//LPAR1//CXCL12 |
| GO:0072507 | divalent inorganic cation homeostasis | Biological process | 14 | 276 | 428 | 14747 | 1.74774820533658 | 0.0309803822871053 | 0.221507410985315 | 1.50891322750141 | CASQ2//ANK2//CACNB4//CAV1//CXCL12//BDKRB1//LPAR1//EDNRB//DMD//PLN//FGF2//CCL21//TRPC1//KDR |
| GO:0002042 | cell migration involved in sprouting angiogenesis | Biological process | 3 | 24 | 428 | 14747 | 4.30695093457944 | 0.0312675213746551 | 0.222227733883353 | 1.50490654459573 | FGF2//KDR//SLIT2 |
| GO:0010623 | developmental programmed cell death | Biological process | 3 | 24 | 428 | 14747 | 4.30695093457944 | 0.0312675213746551 | 0.222227733883353 | 1.50490654459573 | KIT//FGF2//CRYAB |
| GO:0060445 | branching involved in salivary gland morphogenesis | Biological process | 3 | 24 | 428 | 14747 | 4.30695093457944 | 0.0312675213746551 | 0.222227733883353 | 1.50490654459573 | FGF7//FGF10//FGFR1 |
| GO:2001242 | regulation of intrinsic apoptotic signaling pathway | Biological process | 3 | 24 | 428 | 14747 | 4.30695093457944 | 0.0312675213746551 | 0.222227733883353 | 1.50490654459573 | VDAC2//CAV1//CLU |
| GO:0048565 | digestive tract development | Biological process | 7 | 104 | 428 | 14747 | 2.31912742631201 | 0.0316875976109512 | 0.224878203878908 | 1.49911068551329 | EDNRB//SFRP1//FGF10//TCF21//KIT//TGFBR2//MYOCD |
| GO:0002863 | positive regulation of inflammatory response to antigenic stimulus | Biological process | 2 | 10 | 428 | 14747 | 6.8911214953271 | 0.0324125754288219 | 0.225987678684286 | 1.48928645955439 | C3//FCER1A |
| GO:0005513 | detection of calcium ion | Biological process | 2 | 10 | 428 | 14747 | 6.8911214953271 | 0.0324125754288219 | 0.225987678684286 | 1.48928645955439 | KCNMB4//KCNIP1 |
| GO:0006600 | creatine metabolic process | Biological process | 2 | 10 | 428 | 14747 | 6.8911214953271 | 0.0324125754288219 | 0.225987678684286 | 1.48928645955439 | GATM//GHR |
| GO:0009296 | flagellum assembly | Biological process | 2 | 10 | 428 | 14747 | 6.8911214953271 | 0.0324125754288219 | 0.225987678684286 | 1.48928645955439 | DMD//NME5 |
| GO:0021670 | lateral ventricle development | Biological process | 2 | 10 | 428 | 14747 | 6.8911214953271 | 0.0324125754288219 | 0.225987678684286 | 1.48928645955439 | AQP1//MYH10 |
| GO:0021936 | regulation of cerebellar granule cell precursor proliferation | Biological process | 2 | 10 | 428 | 14747 | 6.8911214953271 | 0.0324125754288219 | 0.225987678684286 | 1.48928645955439 | FGF2//IGF1 |
| GO:0042693 | muscle cell fate commitment | Biological process | 2 | 10 | 428 | 14747 | 6.8911214953271 | 0.0324125754288219 | 0.225987678684286 | 1.48928645955439 | MEF2C//FGF10 |
| GO:0043267 | negative regulation of potassium ion transport | Biological process | 2 | 10 | 428 | 14747 | 6.8911214953271 | 0.0324125754288219 | 0.225987678684286 | 1.48928645955439 | CASQ2//VIP |
| GO:0046689 | response to mercury ion | Biological process | 2 | 10 | 428 | 14747 | 6.8911214953271 | 0.0324125754288219 | 0.225987678684286 | 1.48928645955439 | AQP1//GATM |
| GO:0048643 | positive regulation of skeletal muscle tissue development | Biological process | 2 | 10 | 428 | 14747 | 6.8911214953271 | 0.0324125754288219 | 0.225987678684286 | 1.48928645955439 | MEF2C//CDON |
| GO:0070365 | hepatocyte differentiation | Biological process | 2 | 10 | 428 | 14747 | 6.8911214953271 | 0.0324125754288219 | 0.225987678684286 | 1.48928645955439 | FRZB//ANXA1 |
| GO:0072109 | glomerular mesangium development | Biological process | 2 | 10 | 428 | 14747 | 6.8911214953271 | 0.0324125754288219 | 0.225987678684286 | 1.48928645955439 | ACTA2//CD34 |
| GO:0046578 | regulation of Ras protein signal transduction | Biological process | 14 | 278 | 428 | 14747 | 1.73517447724064 | 0.0326420348214619 | 0.227255276005185 | 1.48622277626046 | NTRK2//BVES//ALDH1A1//DLC1//ERRFI1//SFRP1//PREX2//CCL21//SPRY1//ITSN1//ARHGEF26//LPAR1//FGF10//IGF1 |
| GO:0030879 | mammary gland development | Biological process | 8 | 128 | 428 | 14747 | 2.15347546728972 | 0.033027455746182 | 0.22926919425552 | 1.48112488068196 | CAV1//FRZB//TGFBR2//FGF10//FGF2//PTHLH//SLIT2//IGF1 |
| GO:2000146 | negative regulation of cell motility | Biological process | 8 | 128 | 428 | 14747 | 2.15347546728972 | 0.033027455746182 | 0.22926919425552 | 1.48112488068196 | SLIT2//TGFBR3//FGF2//DPYSL3//SFRP1//DLC1//PODN//APOD |
| GO:0001654 | eye development | Biological process | 14 | 279 | 428 | 14747 | 1.72895521388135 | 0.0334967399765813 | 0.232188885099297 | 1.47499745801018 | CRYAB//TGFBR2//CDON//FGF10//RDH10//RORB//FOXC1//FOXP2//NTRK2//FGF2//CHRDL1//MYH10//FZD4//CST3 |
| GO:0030193 | regulation of blood coagulation | Biological process | 5 | 62 | 428 | 14747 | 2.77867802230932 | 0.0337101551458936 | 0.232388367631234 | 1.47223924869972 | ANO6//SELP//CD36//CD34//CAV1 |
| GO:0032941 | secretion by tissue | Biological process | 5 | 62 | 428 | 14747 | 2.77867802230932 | 0.0337101551458936 | 0.232388367631234 | 1.47223924869972 | CAV1//AQP1//KCNMA1//TRPC1//FGF10 |
| GO:1900046 | regulation of hemostasis | Biological process | 5 | 62 | 428 | 14747 | 2.77867802230932 | 0.0337101551458936 | 0.232388367631234 | 1.47223924869972 | ANO6//SELP//CAV1//CD36//CD34 |
| GO:1901292 | nucleoside phosphate catabolic process | Biological process | 22 | 498 | 428 | 14747 | 1.52213527005217 | 0.0337204341373064 | 0.232388367631234 | 1.47210684264092 | GNAL//GNG11//RHOJ//PDE8B//PDE7B//ACTC1//NTRK2//BVES//ALDH1A1//DLC1//ERRFI1//CNN3//PLN//FGF10//SFRP1//PREX2//CCL21//SPRY1//ARHGAP6//TPM2//RGS5//RGS22 |
| GO:0048872 | homeostasis of number of cells | Biological process | 10 | 177 | 428 | 14747 | 1.9466444902054 | 0.0338976722918158 | 0.233272726060129 | 1.46983012319892 | ANXA1//MEF2C//EPAS1//KIT//DYRK3//EXOC6//TEX15//TGFBR3//GPAM//GCNT4 |
| GO:0055082 | cellular chemical homeostasis | Biological process | 28 | 670 | 428 | 14747 | 1.43993583484447 | 0.0339914733481169 | 0.233581176364798 | 1.46863001067174 | KCNMB4//CASQ2//ANK2//CACNB4//CAV1//CXCL12//PRNP//CYBRD1//CLN5//BDKRB1//LPAR1//EDNRB//DMD//PLN//SLC22A17//KCNMA1//ASPA//WASF3//PMP22//MPDZ//KCNIP1//FGF2//CCL21//TRPC1//CMA1//OXCT1//VCAM1//NLGN1 |
| GO:0043405 | regulation of MAP kinase activity | Biological process | 13 | 254 | 428 | 14747 | 1.76347597321363 | 0.0345697231884303 | 0.237212963864207 | 1.46130409798778 | LPAR1//FGF2//FGF10//GHR//KIT//CAV1//FCER1A//FGFR1//SPRY1//TNXB//FZD4//SFRP1//HIPK3 |
| GO:0019228 | regulation of action potential in neuron | Biological process | 7 | 106 | 428 | 14747 | 2.27537030506084 | 0.0346357527738976 | 0.237324576118847 | 1.4604753689627 | ASPA//WASF3//PMP22//LPAR1//MPDZ//KCNMA1//KCNMB4 |
| GO:0046006 | regulation of activated T cell proliferation | Biological process | 3 | 25 | 428 | 14747 | 4.13467289719626 | 0.0347872368209473 | 0.238020562982923 | 1.45858006625309 | IGF1//GPAM//PRNP |
| GO:0043549 | regulation of kinase activity | Biological process | 27 | 643 | 428 | 14747 | 1.44681399979651 | 0.0350884209610108 | 0.23973736327086 | 1.45483617533973 | CCNC//DIRAS3//FGF2//LPAR1//FGF10//GHR//KIT//CAV1//ERRFI1//FABP4//TSPYL2//ANGPT1//FCER1A//TGFBR2//PRKAR2B//FGFR1//SPRY1//TNXB//FZD4//SFRP1//HIPK3//PIK3R1//CCL21//TEK//MYOCD//SPDYA//TTN |
| GO:0072376 | protein activation cascade | Biological process | 6 | 84 | 428 | 14747 | 2.46111481975968 | 0.0352077728414831 | 0.240208681947114 | 1.45336144647899 | A2M//C3//C7//CLU//CFD//CFH |
| GO:0010951 | negative regulation of endopeptidase activity | Biological process | 8 | 130 | 428 | 14747 | 2.12034507548526 | 0.0357256000117829 | 0.243046200365467 | 1.4470204684959 | AQP1//CD44//CRYAB//SFRP1//SERPINA6//CST3//SERPINF1//RECK |
| GO:0060560 | developmental growth involved in morphogenesis | Biological process | 8 | 130 | 428 | 14747 | 2.12034507548526 | 0.0357256000117829 | 0.243046200365467 | 1.4470204684959 | MAP1B//DCLK1//SLIT3//SLIT2//FGF10//RDH10//PTHLH//SFRP1 |
| GO:0071322 | cellular response to carbohydrate stimulus | Biological process | 5 | 63 | 428 | 14747 | 2.7345720219552 | 0.0357781416506304 | 0.243056919561049 | 1.44638222083506 | COLEC12//CALCRL//CMA1//OXCT1//VCAM1 |
| GO:0043542 | endothelial cell migration | Biological process | 7 | 107 | 428 | 14747 | 2.25410516202288 | 0.0361758537869275 | 0.245060577712865 | 1.44158121029667 | FGF2//KDR//SLIT2//ANGPT1//TEK//SCG2 |
| GO:0060191 | regulation of lipase activity | Biological process | 7 | 107 | 428 | 14747 | 2.25410516202288 | 0.0361758537869275 | 0.245060577712865 | 1.44158121029667 | ARHGAP6//LPAR1//PDE1A//PRKAR2B//FGFR1//FGF2//KIT |
| GO:0010563 | negative regulation of phosphorus metabolic process | Biological process | 13 | 256 | 428 | 14747 | 1.7496988171729 | 0.0364679706729922 | 0.24633959226558 | 1.43808840312555 | CAV1//BDKRB1//PRNP//PPAP2B//SLIT2//ERRFI1//FABP4//EDNRB//DMD//SPRY1//SFRP1//HIPK3//MYOCD |
| GO:0045936 | negative regulation of phosphate metabolic process | Biological process | 13 | 256 | 428 | 14747 | 1.7496988171729 | 0.0364679706729922 | 0.24633959226558 | 1.43808840312555 | CAV1//BDKRB1//PRNP//PPAP2B//SLIT2//ERRFI1//FABP4//EDNRB//DMD//SPRY1//SFRP1//HIPK3//MYOCD |
| GO:0031325 | positive regulation of cellular metabolic process | Biological process | 69 | 1928 | 428 | 14747 | 1.23311043355179 | 0.0367151031994924 | 0.247658171369702 | 1.43515524673147 | FGF2//LPAR1//FGF10//GHR//KIT//BVES//C3//EDNRB//MUSK//TEK//CDON//ANGPT1//MYOCD//TSPYL2//GNAL//NTRK2//CAP2//FCER1A//CCL21//VIP//CD36//TGFBR2//EBF1//FOXC1//IGF1//SMAD9//MEF2C//NFATC2//RORB//SFRP1//FZD4//EBF3//PTHLH//AKAP12//CALCRL//ANXA1//TSPYL5//ABCD2//CLU//RNF180//CAV1//CD44//PRKAR2B//DLC1//IL6ST//FGFR1//PTX3//SORBS1//SPDYA//CST3//CCNC//EPAS1//PPP1R12A//NFIB//NFIX//NR4A2//PBX1//PIK3R1//PLAGL1//TCF21//LDB2//FHL5//BEX1//ZNF462//OSR1//FGF7//PPAP2B//SH3D19//ITGA8 |
| GO:0045995 | regulation of embryonic development | Biological process | 6 | 85 | 428 | 14747 | 2.43216052776251 | 0.0369940546045298 | 0.249187353685032 | 1.4318680667416 | CD44//FGFR1//FGF10//SFRP1//OSR1//LAMA2 |
| GO:0022412 | cellular process involved in reproduction in multicellular organism | Biological process | 10 | 180 | 428 | 14747 | 1.91420041536864 | 0.0373684460543784 | 0.25135418791725 | 1.42749496166442 | CXCL12//KIT//NME5//FOXC1//PPAP2A//PPAP2B//FZD4//SFRP1//SLIT3//SLIT2 |
| GO:0002696 | positive regulation of leukocyte activation | Biological process | 12 | 231 | 428 | 14747 | 1.78990168709795 | 0.0374964434622692 | 0.251859913903608 | 1.42600992315124 | MEF2C//NFATC2//CAV1//PIK3R1//CCL21//IL6ST//VCAM1//IGF1//GPAM//FCER1A//FGF10//TGFBR2 |
| GO:0048638 | regulation of developmental growth | Biological process | 7 | 108 | 428 | 14747 | 2.23323381793008 | 0.0377605265298244 | 0.253277005655039 | 1.42296195856351 | MUSK//TGFBR2//MAP1B//FGF2//FGFR1//MEF2C//SFRP1 |
| GO:0048858 | cell projection morphogenesis | Biological process | 28 | 677 | 428 | 14747 | 1.4250472811607 | 0.0380102725086784 | 0.253567505952375 | 1.42009901677966 | ANK2//CACNB4//CNTN1//DPYSL3//FGFR1//GFRA1//ITGA9//LAMA2//MYH10//MYH11//OPHN1//PRNP//SLIT3//TRPC1//SLIT2//MYL9//CAP2//BOC//CXCL12//APOD//NR4A2//DCLK1//NFIB//MAP1B//WEE1//NTRK2//PTPRD//PARVA |
| GO:0033143 | regulation of intracellular steroid hormone receptor signaling pathway | Biological process | 4 | 44 | 428 | 14747 | 3.13232795242141 | 0.0381363854589275 | 0.253567505952375 | 1.41866047147961 | KANK2//IGF1//SFRP1//TCF21 |
| GO:0014910 | regulation of smooth muscle cell migration | Biological process | 3 | 26 | 428 | 14747 | 3.97564701653487 | 0.0385025705521601 | 0.253567505952375 | 1.41451027466419 | IGF1//SLIT2//LPAR1 |
| GO:0043114 | regulation of vascular permeability | Biological process | 3 | 26 | 428 | 14747 | 3.97564701653487 | 0.0385025705521601 | 0.253567505952375 | 1.41451027466419 | ANGPT1//SLIT2//TEK |
| GO:0046579 | positive regulation of Ras protein signal transduction | Biological process | 3 | 26 | 428 | 14747 | 3.97564701653487 | 0.0385025705521601 | 0.253567505952375 | 1.41451027466419 | LPAR1//FGF10//IGF1 |
| GO:0048662 | negative regulation of smooth muscle cell proliferation | Biological process | 3 | 26 | 428 | 14747 | 3.97564701653487 | 0.0385025705521601 | 0.253567505952375 | 1.41451027466419 | APOD//OGN//VIP |
| GO:0060571 | morphogenesis of an epithelial fold | Biological process | 3 | 26 | 428 | 14747 | 3.97564701653487 | 0.0385025705521601 | 0.253567505952375 | 1.41451027466419 | RDH10//FGF10//PTHLH |
| GO:0002685 | regulation of leukocyte migration | Biological process | 6 | 86 | 428 | 14747 | 2.40387959139318 | 0.038837353136442 | 0.253567505952375 | 1.41075037601965 | BDKRB1//SELP//SLIT2//CCL21//CXCL12//APOD |
| GO:0036294 | cellular response to decreased oxygen levels | Biological process | 6 | 86 | 428 | 14747 | 2.40387959139318 | 0.038837353136442 | 0.253567505952375 | 1.41075037601965 | EPAS1//AQP1//PTGIS//SFRP1//MYOCD//CD34 |
| GO:0071456 | cellular response to hypoxia | Biological process | 6 | 86 | 428 | 14747 | 2.40387959139318 | 0.038837353136442 | 0.253567505952375 | 1.41075037601965 | EPAS1//CD34//AQP1//PTGIS//SFRP1//MYOCD |
| GO:0002864 | regulation of acute inflammatory response to antigenic stimulus | Biological process | 2 | 11 | 428 | 14747 | 6.26465590484282 | 0.0388672356576193 | 0.253567505952375 | 1.41041634646248 | C3//FCER1A |
| GO:0006516 | glycoprotein catabolic process | Biological process | 2 | 11 | 428 | 14747 | 6.26465590484282 | 0.0388672356576193 | 0.253567505952375 | 1.41041634646248 | CST3//ADAMTS9 |
| GO:0010613 | positive regulation of cardiac muscle hypertrophy | Biological process | 2 | 11 | 428 | 14747 | 6.26465590484282 | 0.0388672356576193 | 0.253567505952375 | 1.41041634646248 | IGF1//IL6ST |
| GO:0014742 | positive regulation of muscle hypertrophy | Biological process | 2 | 11 | 428 | 14747 | 6.26465590484282 | 0.0388672356576193 | 0.253567505952375 | 1.41041634646248 | IGF1//IL6ST |
| GO:0021924 | cell proliferation in external granule layer | Biological process | 2 | 11 | 428 | 14747 | 6.26465590484282 | 0.0388672356576193 | 0.253567505952375 | 1.41041634646248 | FGF2//IGF1 |
| GO:0021930 | cerebellar granule cell precursor proliferation | Biological process | 2 | 11 | 428 | 14747 | 6.26465590484282 | 0.0388672356576193 | 0.253567505952375 | 1.41041634646248 | FGF2//IGF1 |
| GO:0043116 | negative regulation of vascular permeability | Biological process | 2 | 11 | 428 | 14747 | 6.26465590484282 | 0.0388672356576193 | 0.253567505952375 | 1.41041634646248 | ANGPT1//SLIT2 |
| GO:0050872 | white fat cell differentiation | Biological process | 2 | 11 | 428 | 14747 | 6.26465590484282 | 0.0388672356576193 | 0.253567505952375 | 1.41041634646248 | FABP4//FGF10 |
| GO:1901379 | regulation of potassium ion transmembrane transport | Biological process | 2 | 11 | 428 | 14747 | 6.26465590484282 | 0.0388672356576193 | 0.253567505952375 | 1.41041634646248 | CASQ2//ANK2 |
| GO:2000725 | regulation of cardiac muscle cell differentiation | Biological process | 2 | 11 | 428 | 14747 | 6.26465590484282 | 0.0388672356576193 | 0.253567505952375 | 1.41041634646248 | MEF2C//MYOCD |
| GO:0050877 | neurological system process | Biological process | 47 | 1249 | 428 | 14747 | 1.29656809559797 | 0.0391406612592134 | 0.25500247752075 | 1.40737184145774 | NLGN1//CACNB4//GNAL//GNG7//KCNH1//KCNMA1//PMP22//ABCC9//PDE7B//KCNMB4//KCNIP1//NCALD//TMOD2//BCHE//CLN5//EPAS1//EYA4//EFEMP1//RORB//TIMP3//RDH10//FGFR1//FZD4//SOBP//MEF2C//PRNP//VIP//NTRK2//PRKAR2B//PTN//MUSK//ITGA8//TRIM9//ZDHHC15//BDKRB1//EDNRB//ASPA//WASF3//LAMA2//FOXP2//LPAR1//MPDZ//DMD//OPHN1//ITSN1//MYH10//KIT |
| GO:0060341 | regulation of cellular localization | Biological process | 28 | 679 | 428 | 14747 | 1.42084979285095 | 0.039221680995494 | 0.255181714416795 | 1.40647379691448 | PRKAR2B//CAV1//CASQ2//DMD//ANK2//PLN//NNAT//OXCT1//CRYAB//MAP1B//IGF1//APOD//CD36//FCER1A//TRIM9//SFRP1//PDE8B//PPP1R12A//NTRK2//KCNMB4//GPAM//ANXA1//BDKRB1//TRPC1//PIK3R1//SORBS1//CD34//NLGN1 |
| GO:0043491 | protein kinase B signaling cascade | Biological process | 7 | 109 | 428 | 14747 | 2.21274543427934 | 0.0393901612567751 | 0.255928718029374 | 1.40461224125777 | NTRK2//ANGPT1//IGF1//CCL21//ITSN1//TEK//TSPYL5 |
| GO:0051091 | positive regulation of sequence-specific DNA binding transcription factor activity | Biological process | 10 | 182 | 428 | 14747 | 1.89316524596898 | 0.0398145526887424 | 0.258334152071582 | 1.39995815915164 | CLU//TGFBR3//EDA2R//ANKRD42//KIT//FZD4//PPAP2B//BEX1//ARID5B//MYOCD |
| GO:0051090 | regulation of sequence-specific DNA binding transcription factor activity | Biological process | 16 | 340 | 428 | 14747 | 1.62144035184167 | 0.0399974012062381 | 0.259064262868957 | 1.39796822563343 | PTGIS//PBX1//PRNP//PTHLH//KIT//FZD4//PPAP2B//BEX1//ARID5B//MYOCD//CLU//TGFBR3//EDA2R//ANKRD42//FOXC1//FOXP2 |
| GO:0010466 | negative regulation of peptidase activity | Biological process | 8 | 133 | 428 | 14747 | 2.07251774295552 | 0.0400457453235199 | 0.259064262868957 | 1.3974436189224 | SERPINA6//CST3//SERPINF1//RECK//AQP1//CD44//CRYAB//SFRP1 |
| GO:0032321 | positive regulation of Rho GTPase activity | Biological process | 5 | 65 | 428 | 14747 | 2.65043134435658 | 0.0401443678465421 | 0.259064262868957 | 1.39637537653087 | SFRP1//PREX2//CCL21//DLC1//ERRFI1 |
| GO:0045666 | positive regulation of neuron differentiation | Biological process | 5 | 65 | 428 | 14747 | 2.65043134435658 | 0.0401443678465421 | 0.259064262868957 | 1.39637537653087 | DMD//FGFR1//MEF2C//ZEB1//CDON |
| GO:0048010 | vascular endothelial growth factor receptor signaling pathway | Biological process | 4 | 45 | 428 | 14747 | 3.06272066458982 | 0.0409336562293473 | 0.263444813168363 | 1.38791946206588 | FZD4//FGF10//FOXC1//KDR |
| GO:2000106 | regulation of leukocyte apoptotic process | Biological process | 4 | 45 | 428 | 14747 | 3.06272066458982 | 0.0409336562293473 | 0.263444813168363 | 1.38791946206588 | GPAM//CCL21//CXCL12//MEF2C |
| GO:0021537 | telencephalon development | Biological process | 9 | 158 | 428 | 14747 | 1.96266118537797 | 0.0410053449372141 | 0.263550525613981 | 1.38715953046452 | AQP1//MYH10//FOXP2//PTPRS//SLIT2//NFIB//NTRK2//TACC1//CDON |
| GO:0071900 | regulation of protein serine/threonine kinase activity | Biological process | 17 | 369 | 428 | 14747 | 1.5873857103057 | 0.0414244790173814 | 0.265886057111564 | 1.38274294485912 | CCNC//DIRAS3//LPAR1//FGF2//FGF10//GHR//KIT//CAV1//FCER1A//FGFR1//SPRY1//TNXB//FZD4//SFRP1//HIPK3//MYOCD//SPDYA |
| GO:0051271 | negative regulation of cellular component movement | Biological process | 8 | 134 | 428 | 14747 | 2.05705119263496 | 0.041559871711094 | 0.266396543266408 | 1.38132580171279 | SLIT2//TGFBR3//FGF2//DPYSL3//SFRP1//DLC1//PODN//APOD |
| GO:0046434 | organophosphate catabolic process | Biological process | 23 | 538 | 428 | 14747 | 1.4730092415662 | 0.0416191907210545 | 0.26641868530028 | 1.3807063688336 | GNAL//GNG11//RHOJ//PDE8B//PDE7B//ACTC1//LIPG//NTRK2//BVES//ALDH1A1//DLC1//ERRFI1//CNN3//PLN//FGF10//SFRP1//PREX2//CCL21//SPRY1//ARHGAP6//TPM2//RGS5//RGS22 |
| GO:0010524 | positive regulation of calcium ion transport into cytosol | Biological process | 3 | 27 | 428 | 14747 | 3.82840083073728 | 0.0424113139758317 | 0.268808790695445 | 1.37251827213795 | BDKRB1//TRPC1//CAV1 |
| GO:0018146 | keratan sulfate biosynthetic process | Biological process | 3 | 27 | 428 | 14747 | 3.82840083073728 | 0.0424113139758317 | 0.268808790695445 | 1.37251827213795 | FMOD//OMD//OGN |
| GO:0042517 | positive regulation of tyrosine phosphorylation of Stat3 protein | Biological process | 3 | 27 | 428 | 14747 | 3.82840083073728 | 0.0424113139758317 | 0.268808790695445 | 1.37251827213795 | GHR//IL6ST//KIT |
| GO:0050732 | negative regulation of peptidyl-tyrosine phosphorylation | Biological process | 3 | 27 | 428 | 14747 | 3.82840083073728 | 0.0424113139758317 | 0.268808790695445 | 1.37251827213795 | ERRFI1//CAV1//SFRP1 |
| GO:2000107 | negative regulation of leukocyte apoptotic process | Biological process | 3 | 27 | 428 | 14747 | 3.82840083073728 | 0.0424113139758317 | 0.268808790695445 | 1.37251827213795 | GPAM//CCL21//CXCL12 |
| GO:0046620 | regulation of organ growth | Biological process | 5 | 66 | 428 | 14747 | 2.61027329368451 | 0.0424434932677019 | 0.268808790695445 | 1.37218887914268 | TGFBR2//FGF2//FGFR1//MEF2C//FOXC1 |
| GO:0048641 | regulation of skeletal muscle tissue development | Biological process | 5 | 66 | 428 | 14747 | 2.61027329368451 | 0.0424434932677019 | 0.268808790695445 | 1.37218887914268 | MUSK//MYOCD//MEF2C//BOC//CDON |
| GO:0048644 | muscle organ morphogenesis | Biological process | 5 | 66 | 428 | 14747 | 2.61027329368451 | 0.0424434932677019 | 0.268808790695445 | 1.37218887914268 | ACTC1//TTN//FOXC1//TGFBR3//ARID5B |
| GO:0006029 | proteoglycan metabolic process | Biological process | 6 | 88 | 428 | 14747 | 2.34924596431606 | 0.0426967016963322 | 0.2696961197216 | 1.36960567275972 | MAMDC2//IGF1//GPC5//GPC3//GPC6//UST |
| GO:0007162 | negative regulation of cell adhesion | Biological process | 6 | 88 | 428 | 14747 | 2.34924596431606 | 0.0426967016963322 | 0.2696961197216 | 1.36960567275972 | PIK3R1//FZD4//APOD//ARHGAP6//ANGPT1//JAM2 |
| GO:0032386 | regulation of intracellular transport | Biological process | 13 | 263 | 428 | 14747 | 1.7031288866778 | 0.0436974650946336 | 0.275122967100444 | 1.35954375587497 | CAV1//CASQ2//DMD//ANK2//PLN//CRYAB//MAP1B//IGF1//APOD//CD36//PPP1R12A//BDKRB1//TRPC1 |
| GO:0072503 | cellular divalent inorganic cation homeostasis | Biological process | 13 | 263 | 428 | 14747 | 1.7031288866778 | 0.0436974650946336 | 0.275122967100444 | 1.35954375587497 | CASQ2//ANK2//CACNB4//CAV1//CXCL12//BDKRB1//LPAR1//EDNRB//DMD//PLN//FGF2//CCL21//TRPC1 |
| GO:0006940 | regulation of smooth muscle contraction | Biological process | 4 | 46 | 428 | 14747 | 2.996139780577 | 0.0438442975458875 | 0.275122967100444 | 1.35808688360692 | MYOCD//KCNMA1//CAV1//CNN1 |
| GO:0042531 | positive regulation of tyrosine phosphorylation of STAT protein | Biological process | 4 | 46 | 428 | 14747 | 2.996139780577 | 0.0438442975458875 | 0.275122967100444 | 1.35808688360692 | IL6ST//KIT//GHR//IGF1 |
| GO:0048742 | regulation of skeletal muscle fiber development | Biological process | 4 | 46 | 428 | 14747 | 2.996139780577 | 0.0438442975458875 | 0.275122967100444 | 1.35808688360692 | MUSK//MYOCD//MEF2C//BOC |
| GO:0010975 | regulation of neuron projection development | Biological process | 11 | 211 | 428 | 14747 | 1.79626389688621 | 0.0441964859301364 | 0.276968516952458 | 1.35461226010387 | CNTN1//DMD//DPYSL3//FGFR1//NTRK2//LPAR1//PMP22//MAP1B//SLIT2//PTPRD//NLGN1 |
| GO:0033135 | regulation of peptidyl-serine phosphorylation | Biological process | 5 | 67 | 428 | 14747 | 2.5713139907937 | 0.0448204314177611 | 0.279775703444113 | 1.34852396749191 | CAV1//DMD//ANGPT1//CD44//TEK |
| GO:0048489 | synaptic vesicle transport | Biological process | 5 | 67 | 428 | 14747 | 2.5713139907937 | 0.0448204314177611 | 0.279775703444113 | 1.34852396749191 | NLGN1//TRIM9//ZDHHC15//OPHN1//ITSN1 |
| GO:0050818 | regulation of coagulation | Biological process | 5 | 67 | 428 | 14747 | 2.5713139907937 | 0.0448204314177611 | 0.279775703444113 | 1.34852396749191 | ANO6//SELP//CAV1//CD36//CD34 |
| GO:0009790 | embryo development | Biological process | 36 | 927 | 428 | 14747 | 1.33808184375284 | 0.0455563323781957 | 0.279794208511492 | 1.3414512471045 | ANGPT1//FGFR1//FOXC1//MYH10//TGFBR2//TTN//SLIT2//HEG1//RDH10//PPAP2B//MEOX2//TGFBR3//SFRP1//DLC1//EPAS1//MEF2C//FGF10//CDON//PBX1//CD44//OSR1//AFF3//KDR//KIT//ITGA8//SOBP//EYA4//LAMA2//TCF21//FGF2//VCAM1//FRZB//GATM//FZD4//EDA2R//FOXP2 |
| GO:0032990 | cell part morphogenesis | Biological process | 28 | 689 | 428 | 14747 | 1.40022788003744 | 0.0457172969452335 | 0.279794208511492 | 1.33991945536497 | ANK2//CACNB4//CNTN1//DPYSL3//FGFR1//GFRA1//ITGA9//LAMA2//MYH10//MYH11//OPHN1//PRNP//SLIT3//TRPC1//SLIT2//MYL9//CAP2//BOC//CXCL12//APOD//NR4A2//DCLK1//NFIB//MAP1B//WEE1//NTRK2//PTPRD//PARVA |
| GO:0007158 | neuron cell-cell adhesion | Biological process | 2 | 12 | 428 | 14747 | 5.74260124610592 | 0.0457621058165157 | 0.279794208511492 | 1.33949399899977 | NCAM2//NLGN1 |
| GO:0010837 | regulation of keratinocyte proliferation | Biological process | 2 | 12 | 428 | 14747 | 5.74260124610592 | 0.0457621058165157 | 0.279794208511492 | 1.33949399899977 | FGF7//FGF10 |
| GO:0021534 | cell proliferation in hindbrain | Biological process | 2 | 12 | 428 | 14747 | 5.74260124610592 | 0.0457621058165157 | 0.279794208511492 | 1.33949399899977 | FGF2//IGF1 |
| GO:0042510 | regulation of tyrosine phosphorylation of Stat1 protein | Biological process | 2 | 12 | 428 | 14747 | 5.74260124610592 | 0.0457621058165157 | 0.279794208511492 | 1.33949399899977 | IL6ST//KIT |
| GO:0043064 | flagellum organization | Biological process | 2 | 12 | 428 | 14747 | 5.74260124610592 | 0.0457621058165157 | 0.279794208511492 | 1.33949399899977 | DMD//NME5 |
| GO:0043508 | negative regulation of JUN kinase activity | Biological process | 2 | 12 | 428 | 14747 | 5.74260124610592 | 0.0457621058165157 | 0.279794208511492 | 1.33949399899977 | SFRP1//HIPK3 |
| GO:0043568 | positive regulation of insulin-like growth factor receptor signaling pathway | Biological process | 2 | 12 | 428 | 14747 | 5.74260124610592 | 0.0457621058165157 | 0.279794208511492 | 1.33949399899977 | IGF1//IGFBP4 |
| GO:0050919 | negative chemotaxis | Biological process | 2 | 12 | 428 | 14747 | 5.74260124610592 | 0.0457621058165157 | 0.279794208511492 | 1.33949399899977 | SLIT2//SLIT3 |
| GO:0060306 | regulation of membrane repolarization | Biological process | 2 | 12 | 428 | 14747 | 5.74260124610592 | 0.0457621058165157 | 0.279794208511492 | 1.33949399899977 | ANK2//CASQ2 |
| GO:0060525 | prostate glandular acinus development | Biological process | 2 | 12 | 428 | 14747 | 5.74260124610592 | 0.0457621058165157 | 0.279794208511492 | 1.33949399899977 | IGF1//SFRP1 |
| GO:0060999 | positive regulation of dendritic spine development | Biological process | 2 | 12 | 428 | 14747 | 5.74260124610592 | 0.0457621058165157 | 0.279794208511492 | 1.33949399899977 | LPAR1//NLGN1 |
| GO:0086009 | membrane repolarization | Biological process | 2 | 12 | 428 | 14747 | 5.74260124610592 | 0.0457621058165157 | 0.279794208511492 | 1.33949399899977 | CASQ2//ANK2 |
| GO:0090136 | epithelial cell-cell adhesion | Biological process | 2 | 12 | 428 | 14747 | 5.74260124610592 | 0.0457621058165157 | 0.279794208511492 | 1.33949399899977 | KIT//BVES |
| GO:0090322 | regulation of superoxide metabolic process | Biological process | 2 | 12 | 428 | 14747 | 5.74260124610592 | 0.0457621058165157 | 0.279794208511492 | 1.33949399899977 | CD36//FBLN5 |
| GO:0030212 | hyaluronan metabolic process | Biological process | 3 | 28 | 428 | 14747 | 3.69167222963952 | 0.0465108741810419 | 0.283288286565857 | 1.33244549776144 | CD44//FGF2//LYVE1 |
| GO:0046326 | positive regulation of glucose import | Biological process | 3 | 28 | 428 | 14747 | 3.69167222963952 | 0.0465108741810419 | 0.283288286565857 | 1.33244549776144 | IGF1//PIK3R1//SORBS1 |
| GO:0051251 | positive regulation of lymphocyte activation | Biological process | 11 | 213 | 428 | 14747 | 1.77939756921592 | 0.0467460466847215 | 0.283288286565857 | 1.33025511154243 | MEF2C//NFATC2//CAV1//PIK3R1//CCL21//IL6ST//VCAM1//IGF1//GPAM//FGF10//TGFBR2 |
| GO:0009791 | post-embryonic development | Biological process | 6 | 90 | 428 | 14747 | 2.29704049844237 | 0.0467888423884765 | 0.283288286565857 | 1.32985769963612 | NR4A2//SEPP1//TIPARP//HEG1//ARID5B//FOXP2 |
| GO:0032318 | regulation of Ras GTPase activity | Biological process | 9 | 162 | 428 | 14747 | 1.91420041536864 | 0.0467940979940363 | 0.283288286565857 | 1.32980891979469 | NTRK2//BVES//ALDH1A1//DLC1//ERRFI1//SFRP1//PREX2//CCL21//SPRY1 |
| GO:0002444 | myeloid leukocyte mediated immunity | Biological process | 4 | 47 | 428 | 14747 | 2.93239212567111 | 0.0468682025792537 | 0.283288286565857 | 1.32912170158711 | C3//KIT//FCER1A//CTSG |
| GO:0006885 | regulation of pH | Biological process | 4 | 47 | 428 | 14747 | 2.93239212567111 | 0.0468682025792537 | 0.283288286565857 | 1.32912170158711 | CLN5//EDNRB//PDK4//SLC9A9 |
| GO:0007189 | adenylate cyclase-activating G-protein coupled receptor signaling pathway | Biological process | 4 | 47 | 428 | 14747 | 2.93239212567111 | 0.0468682025792537 | 0.283288286565857 | 1.32912170158711 | GNAL//VIP//PTHLH//CALCRL |
| GO:0043149 | stress fiber assembly | Biological process | 4 | 47 | 428 | 14747 | 2.93239212567111 | 0.0468682025792537 | 0.283288286565857 | 1.32912170158711 | SFRP1//ARHGAP6//DLC1//SORBS1 |
| GO:0055007 | cardiac muscle cell differentiation | Biological process | 5 | 68 | 428 | 14747 | 2.53350054975261 | 0.0472754468612555 | 0.285388108963706 | 1.32536435738593 | ACTC1//MYH10//TTN//MEF2C//MYOCD |
| GO:0048523 | negative regulation of cellular process | Biological process | 99 | 2922 | 428 | 14747 | 1.16738711163139 | 0.0478772180422624 | 0.288655439751643 | 1.31987109268491 | WEE1//CAV1//CD36//EDNRB//FGFR1//FOSB//MEF2C//NFIB//NFIX//ZEB1//TCF21//NR2F1//KANK2//TAF9B//MYOCD//OSR1//BDKRB1//PRNP//PPAP2B//SLIT2//SCG2//PIK3R1//ERRFI1//FABP4//PLAGL1//CAB39L//ANGPT1//JAM2//TSPYL2//ANXA1//AQP1//CD44//CLU//CRYAB//FGF10//FOXC1//IGF1//IL6ST//KDR//PTGFR//CXCL12//SFRP1//TEK//VIP//NME5//HIPK3//FAIM2//CDH5//DPT//FGF2//FRZB//PMP22//PTHLH//SLIT3//PPAP2A//ADAMTS1//SPRY1//DLC1//TENC1//PODN//TGFBR3//APOD//CST3//FZD4//DPYSL3//LPAR1//ARID5B//FOXP2//KIT//APCDD1//WFDC1//TGFBR2//VDAC2//EFEMP1//DMD//LDB2//TRIM9//RGS5//RGS22//CNTFR//NTRK2//NR4A2//ITSN1//CILP//CD34//PTGIS//PBX1//PDE8B//OGN//TIMP3//RCAN1//ARHGAP6//SRPX//SERPINF1//GPAM//PLN//GHR//CCL21//PDK4 |
| GO:0030031 | cell projection assembly | Biological process | 11 | 214 | 428 | 14747 | 1.77108262730369 | 0.0480577254220177 | 0.289377894618185 | 1.31823678825628 | MYH10//OPHN1//DMD//NME5//SLIT2//KIT//WASF3//CCL21//LPAR1//DPYSL3//NLGN1 |
| GO:0016192 | vesicle-mediated transport | Biological process | 35 | 902 | 428 | 14747 | 1.33696924798475 | 0.048681830671656 | 0.292766267935848 | 1.3126330985675 | SRPX//ANGPT1//A2M//CD36//CLU//CFD//F13A1//IGF1//SELP//TTN//MMRN1//NLGN1//MYH10//PIK3C2A//SH3D19//RAB9B//GHR//TGFBR2//EXOC6//COLEC12//PTX3//TRIM9//ZDHHC15//OPHN1//ANXA1//CALCRL//KIT//FCER1A//CCL21//ITSN1//C3//CAV1//BVES//ERGIC2//NCALD |
| GO:0048741 | skeletal muscle fiber development | Biological process | 6 | 91 | 428 | 14747 | 2.27179829516278 | 0.0489229882284486 | 0.293846008641652 | 1.3104870240092 | CACNB4//MUSK//MYOCD//MEF2C//BOC//RCAN1 |
