# Supplementary material for: Downregulation of miR-181b-5p Inhibits the Viability, Migration, and Glycolysis of Gallbladder Cancer by Upregulating PDHX Under Hypoxia
Source: Front Oncol. 2021 Aug 16;11:683725. doi: 10.3389/fonc.2021.683725 (PMC8415503; doi:10.3389/fonc.2021.683725)
Supplement: Supplementary file 3 [file DataSheet_1.zip › RNA seq raw data/mirPath Analysis/A vs B_dn miRNA up Gene/DEG Target/TargetsOnDEGs.pdf]

miRNA	Rfam	Gene	miRanda	TargetScan	PicTar	mirTarBase
hsa-miR-10b-5p	mir-10	CDKN2A				✔
hsa-miR-10b-5p	mir-10	CNNM4	✔	✔	✔	
hsa-miR-10b-5p	mir-10	ELOVL2	✔	✔		
hsa-miR-10b-5p	mir-10	HSPA1B				✔
hsa-miR-140-3p	mir-140	ANKRD13B	✔	✔		
hsa-miR-140-3p	mir-140	P2RY2	✔	✔		
hsa-miR-140-3p	mir-140	SULF1	✔		✔	
hsa-miR-140-3p	mir-140	WNT5A	✔	✔		
hsa-miR-26b-5p	mir-26	AGMAT				✔
hsa-miR-26b-5p	mir-26	AK4				✔
hsa-miR-26b-5p	mir-26	ANXA3				✔
hsa-miR-26b-5p	mir-26	ANXA8				✔
hsa-miR-26b-5p	mir-26	ANXA8L1				✔
hsa-miR-26b-5p	mir-26	ANXA8L2				✔
hsa-miR-26b-5p	mir-26	ARHGEF5				✔
hsa-miR-26b-5p	mir-26	BAHD1				✔
hsa-miR-26b-5p	mir-26	CBLC				✔
hsa-miR-26b-5p	mir-26	CCNE1	✔		✔	✔
hsa-miR-26b-5p	mir-26	CDH2	✔	✔	✔	
hsa-miR-26b-5p	mir-26	CDH4	✔	✔	✔	
hsa-miR-26b-5p	mir-26	CELSR1	✔	✔	✔	
hsa-miR-26b-5p	mir-26	CXCL6				✔
hsa-miR-26b-5p	mir-26	CYP2D7P1				✔
hsa-miR-26b-5p	mir-26	ELOVL2				✔
hsa-miR-26b-5p	mir-26	FBXL19	✔	✔		
hsa-miR-26b-5p	mir-26	FRAT2	✔	✔		
hsa-miR-26b-5p	mir-26	FZD5				✔
hsa-miR-26b-5p	mir-26	GALK1				✔
hsa-miR-26b-5p	mir-26	GRB14				✔
hsa-miR-26b-5p	mir-26	GRB7				✔
hsa-miR-26b-5p	mir-26	HIST1H2BC				✔
hsa-miR-26b-5p	mir-26	HIST2H2BF				✔
hsa-miR-26b-5p	mir-26	HIST2H4B				✔
hsa-miR-26b-5p	mir-26	IER5				✔
hsa-miR-26b-5p	mir-26	IGSF6				✔
hsa-miR-26b-5p	mir-26	IL17RB				✔
hsa-miR-26b-5p	mir-26	IL1R2				✔
hsa-miR-26b-5p	mir-26	ITGAX				✔
hsa-miR-26b-5p	mir-26	KIAA0101				✔
hsa-miR-26b-5p	mir-26	LAMA3				✔
hsa-miR-26b-5p	mir-26	MRM1				✔
hsa-miR-26b-5p	mir-26	SND1				✔
hsa-miR-26b-5p	mir-26	SNRPN				✔
hsa-miR-26b-5p	mir-26	SULF1	✔	✔		
hsa-miR-26b-5p	mir-26	SULT1B1				✔
hsa-miR-26b-5p	mir-26	TNFSF15				✔
hsa-miR-26b-5p	mir-26	TRIM65				✔
hsa-miR-26b-5p	mir-26	WNT5A	✔	✔	✔	
hsa-miR-26b-5p	mir-26	YOD1				✔
hsa-miR-335-5p	mir-335	ABCA12				✔
hsa-miR-335-5p	mir-335	ADAM8				✔
hsa-miR-335-5p	mir-335	APOC1				✔
hsa-miR-335-5p	mir-335	BIK				✔
hsa-miR-335-5p	mir-335	C10orf10				✔

To be continued...

miRNA	Rfam	Gene	miRanda	TargetScan	PicTar	mirTarBase
hsa-miR-335-5p	mir-335	C8G				✔
hsa-miR-335-5p	mir-335	CCL24				✔
hsa-miR-335-5p	mir-335	CD177				✔
hsa-miR-335-5p	mir-335	CDH3				✔
hsa-miR-335-5p	mir-335	CDHR2				✔
hsa-miR-335-5p	mir-335	CEACAM5				✔
hsa-miR-335-5p	mir-335	CLDN4				✔
hsa-miR-335-5p	mir-335	CYB561				✔
hsa-miR-335-5p	mir-335	DIO1				✔
hsa-miR-335-5p	mir-335	EFNA1				✔
hsa-miR-335-5p	mir-335	F11R				✔
hsa-miR-335-5p	mir-335	FAAH2				✔
hsa-miR-335-5p	mir-335	GRB7				✔
hsa-miR-335-5p	mir-335	HIST1H4L				✔
hsa-miR-335-5p	mir-335	HSD3B7				✔
hsa-miR-335-5p	mir-335	HSPA1A				✔
hsa-miR-335-5p	mir-335	HSPA1B				✔
hsa-miR-335-5p	mir-335	IRF6				✔
hsa-miR-335-5p	mir-335	JUP	✔	✔		✔
hsa-miR-335-5p	mir-335	LBP				✔
hsa-miR-335-5p	mir-335	LFNG				✔
hsa-miR-335-5p	mir-335	LLGL2				✔
hsa-miR-335-5p	mir-335	LPAL2				✔
hsa-miR-335-5p	mir-335	LRG1				✔
hsa-miR-335-5p	mir-335	MOCOS				✔
hsa-miR-335-5p	mir-335	MUC4				✔
hsa-miR-335-5p	mir-335	OLFM4				✔
hsa-miR-335-5p	mir-335	P2RY2				✔
hsa-miR-335-5p	mir-335	PCYT2				✔
hsa-miR-335-5p	mir-335	PLXNA3				✔
hsa-miR-335-5p	mir-335	PNPLA3				✔
hsa-miR-335-5p	mir-335	PRR15L				✔
hsa-miR-335-5p	mir-335	RHBDF2				✔
hsa-miR-335-5p	mir-335	SIGLEC5				✔
hsa-miR-335-5p	mir-335	SIRT7				✔
hsa-miR-335-5p	mir-335	SULT2A1				✔
hsa-miR-335-5p	mir-335	TACSTD2				✔
hsa-miR-335-5p	mir-335	TSTD1				✔
hsa-miR-335-5p	mir-335	UBD				✔
hsa-miR-335-5p	mir-335	VILL				✔
hsa-miR-451a	mir-451	MIF				✔
hsa-miR-494		EN2	✔	✔		
hsa-miR-494		F11R	✔	✔		
hsa-miR-494		FAM40B	✔	✔		
hsa-miR-494		NCF2	✔	✔		
						(END)
