# Supplementary material for: Downregulation of miR-181b-5p Inhibits the Viability, Migration, and Glycolysis of Gallbladder Cancer by Upregulating PDHX Under Hypoxia
Source: Front Oncol. 2021 Aug 16;11:683725. doi: 10.3389/fonc.2021.683725 (PMC8415503; doi:10.3389/fonc.2021.683725)
Supplement: Supplementary file 3 [file DataSheet_1.zip › RNA seq raw data/mirPath Analysis/A vs B_up miRNA dn Gene/DEG Target/TargetsOnDEGs.pdf]

miRNA	Rfam	Gene	miRanda	TargetScan	PicTar	mirTarBase
hsa-miR-125a-5p	mir-10	ANKRD42	✔	✔		
hsa-miR-125a-5p	mir-10	ANO6	✔	✔		
hsa-miR-125a-5p	mir-10	CD34	✔		✔	✔
hsa-miR-125a-5p	mir-10	CDH5	✔	✔		
hsa-miR-125a-5p	mir-10	CYB5D1	✔	✔		
hsa-miR-125a-5p	mir-10	FAT4	✔	✔		
hsa-miR-125a-5p	mir-10	GPC6	✔	✔		
hsa-miR-125a-5p	mir-10	ITGA9	✔	✔	✔	
hsa-miR-125a-5p	mir-10	LIFR		✔	✔	
hsa-miR-125a-5p	mir-10	MAMDC2	✔	✔	✔	
hsa-miR-125a-5p	mir-10	MAT2A				✔
hsa-miR-125a-5p	mir-10	MSRB3	✔	✔		
hsa-miR-125a-5p	mir-10	PLAGL1	✔	✔	✔	
hsa-miR-125a-5p	mir-10	SLCO2A1	✔	✔		
hsa-miR-125a-5p	mir-10	TAF9B	✔	✔	✔	
hsa-miR-125a-5p	mir-10	UBE2J1	✔	✔		
hsa-miR-125b-2-3p	mir-10	KDELC2				✔
hsa-miR-125b-2-3p	mir-10	MAMDC2	✔		✔	
hsa-miR-146b-3p	mir-146	KANK2	✔	✔		
hsa-miR-146b-3p	mir-146	MSRB3	✔	✔		
hsa-miR-149-5p	mir-149	CACHD1	✔	✔		
hsa-miR-149-5p	mir-149	CCNI	✔	✔	✔	
hsa-miR-149-5p	mir-149	HSPB6		✔	✔	
hsa-miR-149-5p	mir-149	IGJ	✔	✔		
hsa-miR-149-5p	mir-149	MSRB3	✔	✔		
hsa-miR-149-5p	mir-149	NCALD	✔	✔		
hsa-miR-149-5p	mir-149	NFIB		✔	✔	
hsa-miR-149-5p	mir-149	NFIX	✔	✔		✔
hsa-miR-149-5p	mir-149	NR2F1				✔
hsa-miR-149-5p	mir-149	PBX1				✔
hsa-miR-149-5p	mir-149	PDE1A	✔	✔		
hsa-miR-149-5p	mir-149	PPAP2B	✔	✔	✔	
hsa-miR-149-5p	mir-149	PTGIS	✔	✔	✔	
hsa-miR-149-5p	mir-149	RAVER2				✔
hsa-miR-149-5p	mir-149	TNXB	✔		✔	
hsa-miR-181a-5p	mir-181	ABI3BP	✔	✔	✔	
hsa-miR-181a-5p	mir-181	ADAMTS1	✔	✔	✔	
hsa-miR-181a-5p	mir-181	AKAP12				✔
hsa-miR-181a-5p	mir-181	BAI3	✔	✔		
hsa-miR-181a-5p	mir-181	CCDC141	✔	✔		
hsa-miR-181a-5p	mir-181	CDON	✔	✔		
hsa-miR-181a-5p	mir-181	CHIC1	✔	✔		
hsa-miR-181a-5p	mir-181	CNTNAP3	✔		✔	
hsa-miR-181a-5p	mir-181	CRISPLD1	✔	✔		
hsa-miR-181a-5p	mir-181	DCLK1	✔	✔		
hsa-miR-181a-5p	mir-181	EYA4				✔
hsa-miR-181a-5p	mir-181	FAM171A1	✔	✔		
hsa-miR-181a-5p	mir-181	FAM190B	✔		✔	
hsa-miR-181a-5p	mir-181	FAM46C	✔	✔		
hsa-miR-181a-5p	mir-181	ITSN1	✔	✔		
hsa-miR-181a-5p	mir-181	KCNH1	✔	✔		
hsa-miR-181a-5p	mir-181	KCNMA1	✔	✔		
hsa-miR-181a-5p	mir-181	LPPR4	✔	✔		
hsa-miR-181a-5p	mir-181	MAMDC2	✔	✔	✔	

To be continued...

miRNA	Rfam	Gene	miRanda	TargetScan	PicTar	mirTarBase
hsa-miR-181a-5p	mir-181	MAP1B	✔	✔	✔	
hsa-miR-181a-5p	mir-181	MYH10	✔	✔	✔	
hsa-miR-181a-5p	mir-181	NCALD	✔	✔	✔	
hsa-miR-181a-5p	mir-181	NEGR1	✔	✔		
hsa-miR-181a-5p	mir-181	NFIB	✔	✔		
hsa-miR-181a-5p	mir-181	OSBPL6	✔	✔		
hsa-miR-181a-5p	mir-181	PARM1	✔	✔		
hsa-miR-181a-5p	mir-181	PBX1	✔	✔		
hsa-miR-181a-5p	mir-181	PDK4	✔		✔	
hsa-miR-181a-5p	mir-181	PIK3C2A	✔	✔		
hsa-miR-181a-5p	mir-181	PPAP2B	✔	✔		
hsa-miR-181a-5p	mir-181	RECK	✔	✔	✔	
hsa-miR-181a-5p	mir-181	RFTN2	✔	✔		
hsa-miR-181a-5p	mir-181	RIMKLB	✔	✔		
hsa-miR-181a-5p	mir-181	RORB	✔	✔		
hsa-miR-181a-5p	mir-181	SLCO2A1				✔
hsa-miR-181a-5p	mir-181	TAF9B	✔	✔	✔	
hsa-miR-181a-5p	mir-181	TBCEL	✔	✔		
hsa-miR-181a-5p	mir-181	TCF21				✔
hsa-miR-181a-5p	mir-181	TGFBR2	✔	✔		
hsa-miR-181a-5p	mir-181	TGFBR3				✔
hsa-miR-181a-5p	mir-181	TIMP3	✔	✔		
hsa-miR-181a-5p	mir-181	TLL1	✔	✔		
hsa-miR-181a-5p	mir-181	TMEM47	✔	✔		
hsa-miR-181a-5p	mir-181	TNS1	✔	✔		
hsa-miR-181a-5p	mir-181	TRIM9	✔		✔	
hsa-miR-181a-5p	mir-181	UBE2J1	✔	✔		
hsa-miR-181a-5p	mir-181	ZNF280B	✔	✔		
hsa-miR-181b-5p	mir-181	ABI3BP	✔	✔	✔	
hsa-miR-181b-5p	mir-181	ADAMTS1	✔	✔	✔	
hsa-miR-181b-5p	mir-181	BAI3	✔	✔	✔	
hsa-miR-181b-5p	mir-181	CCDC141	✔	✔		
hsa-miR-181b-5p	mir-181	CDON	✔	✔		
hsa-miR-181b-5p	mir-181	CHIC1	✔	✔		
hsa-miR-181b-5p	mir-181	CRISPLD1	✔	✔		
hsa-miR-181b-5p	mir-181	DCLK1	✔	✔	✔	
hsa-miR-181b-5p	mir-181	FAM171A1	✔	✔		
hsa-miR-181b-5p	mir-181	FAM190B	✔		✔	
hsa-miR-181b-5p	mir-181	FAM46C	✔	✔		
hsa-miR-181b-5p	mir-181	FGFR1				✔
hsa-miR-181b-5p	mir-181	ITSN1	✔	✔		
hsa-miR-181b-5p	mir-181	KCNH1	✔	✔		
hsa-miR-181b-5p	mir-181	KCNMA1	✔	✔		
hsa-miR-181b-5p	mir-181	LPPR4	✔	✔		
hsa-miR-181b-5p	mir-181	MAMDC2	✔	✔	✔	
hsa-miR-181b-5p	mir-181	MAP1B	✔	✔	✔	
hsa-miR-181b-5p	mir-181	MYH10	✔	✔	✔	
hsa-miR-181b-5p	mir-181	NCALD	✔	✔	✔	
hsa-miR-181b-5p	mir-181	NEGR1	✔	✔		
hsa-miR-181b-5p	mir-181	NFIB	✔	✔		
hsa-miR-181b-5p	mir-181	NR2F1				✔
hsa-miR-181b-5p	mir-181	OSBPL6	✔	✔		
hsa-miR-181b-5p	mir-181	PARM1	✔	✔		
hsa-miR-181b-5p	mir-181	PBX1	✔	✔		

To be continued...

miRNA	Rfam	Gene	miRanda	TargetScan	PicTar	mirTarBase
hsa-miR-181b-5p	mir-181	PDK4	✓		✓	
hsa-miR-181b-5p	mir-181	PIK3C2A	✓	✓		
hsa-miR-181b-5p	mir-181	PLCXD3	✓	✓		
hsa-miR-181b-5p	mir-181	PRICKLE2	✓	✓		
hsa-miR-181b-5p	mir-181	RECK	✓	✓	✓	
hsa-miR-181b-5p	mir-181	RFTN2	✓	✓		
hsa-miR-181b-5p	mir-181	RIMKLB	✓	✓		
hsa-miR-181b-5p	mir-181	RORB	✓	✓		
hsa-miR-181b-5p	mir-181	TAF9B	✓	✓	✓	
hsa-miR-181b-5p	mir-181	TBCEL	✓	✓		
hsa-miR-181b-5p	mir-181	TGFBR2	✓	✓		
hsa-miR-181b-5p	mir-181	TGFBR3	✓	✓		
hsa-miR-181b-5p	mir-181	TIMP3	✓	✓	✓	✓
hsa-miR-181b-5p	mir-181	TLL1	✓	✓		
hsa-miR-181b-5p	mir-181	TMEM47	✓	✓		
hsa-miR-181b-5p	mir-181	TNS1	✓	✓		
hsa-miR-181b-5p	mir-181	TRIM9	✓		✓	
hsa-miR-181b-5p	mir-181	UBE2J1	✓	✓		
hsa-miR-181b-5p	mir-181	ZNF280B	✓	✓		
hsa-miR-188-5p	mir-188	EMP1	✓	✓		
hsa-miR-188-5p	mir-188	LPAR1	✓		✓	
hsa-miR-188-5p	mir-188	MEF2C	✓		✓	
hsa-miR-188-5p	mir-188	PCDH9	✓	✓	✓	
hsa-miR-192-3p	mir-192	LPHN3	✓		✓	
hsa-miR-200a-5p	mir-8	DLC1	✓		✓	
hsa-miR-200a-5p	mir-8	MYH10	✓		✓	
hsa-miR-200a-5p	mir-8	PCDH9	✓		✓	
hsa-miR-205-5p	mir-205	ADAMTS9	✓	✓	✓	
hsa-miR-205-5p	mir-205	ANK2		✓	✓	
hsa-miR-205-5p	mir-205	CALCRL	✓	✓	✓	
hsa-miR-205-5p	mir-205	CDON	✓		✓	
hsa-miR-205-5p	mir-205	CHIC1	✓	✓		
hsa-miR-205-5p	mir-205	ERRFI1	✓	✓		
hsa-miR-205-5p	mir-205	FERMT2	✓		✓	
hsa-miR-205-5p	mir-205	GCOM1	✓	✓		
hsa-miR-205-5p	mir-205	LPAR1	✓	✓		
hsa-miR-205-5p	mir-205	NFIB	✓	✓		
hsa-miR-205-5p	mir-205	PI16	✓	✓		
hsa-miR-205-5p	mir-205	PTP4A1	✓	✓		
hsa-miR-205-5p	mir-205	RBPMS2	✓	✓		
hsa-miR-205-5p	mir-205	SCD5	✓	✓		
hsa-miR-205-5p	mir-205	SORBS1	✓	✓	✓	
hsa-miR-205-5p	mir-205	VIP	✓	✓		
hsa-miR-205-5p	mir-205	ZEB1	✓	✓		✓
hsa-miR-210		ZNF462	✓	✓		
hsa-miR-214-5p	mir-214	PLCB4	✓		✓	
hsa-miR-214-5p	mir-214	TSPAN18	✓		✓	
hsa-miR-222-3p	mir-221	CACNB4	✓	✓		
hsa-miR-222-3p	mir-221	CDON	✓	✓		
hsa-miR-222-3p	mir-221	CXCL12	✓	✓		
hsa-miR-222-3p	mir-221	EBF3	✓		✓	
hsa-miR-222-3p	mir-221	EPB41L2				✓
hsa-miR-222-3p	mir-221	FERMT2	✓		✓	
hsa-miR-222-3p	mir-221	FOXP2	✓	✓		

To be continued...

miRNA	Rfam	Gene	miRanda	TargetScan	PicTar	mirTarBase
hsa-miR-222-3p	mir-221	IGF1	✔	✔		
hsa-miR-222-3p	mir-221	KDR	✔	✔		
hsa-miR-222-3p	mir-221	KIT	✔	✔		✔
hsa-miR-222-3p	mir-221	KSR1	✔	✔		
hsa-miR-222-3p	mir-221	MAP1B				✔
hsa-miR-222-3p	mir-221	MAT2A	✔		✔	
hsa-miR-222-3p	mir-221	NRK	✔	✔		
hsa-miR-222-3p	mir-221	PCDH9	✔	✔		
hsa-miR-222-3p	mir-221	PIK3R1	✔	✔		
hsa-miR-222-3p	mir-221	PLCXD3	✔	✔		
hsa-miR-222-3p	mir-221	RECK	✔	✔		✔
hsa-miR-222-3p	mir-221	SCD5	✔	✔		
hsa-miR-222-3p	mir-221	TIMP3	✔	✔		✔
hsa-miR-222-3p	mir-221	UBE2J1	✔	✔	✔	
hsa-miR-222-3p	mir-221	WEE1	✔	✔		
hsa-miR-222-3p	mir-221	ZNF518B	✔	✔		
hsa-miR-23b-3p	mir-23	ABI3BP	✔	✔		
hsa-miR-23b-3p	mir-23	AKAP12	✔	✔		
hsa-miR-23b-3p	mir-23	APOLD1	✔	✔		
hsa-miR-23b-3p	mir-23	B3GALT2	✔	✔	✔	
hsa-miR-23b-3p	mir-23	C5orf4	✔	✔		
hsa-miR-23b-3p	mir-23	CADM3	✔	✔		
hsa-miR-23b-3p	mir-23	CALCRL	✔	✔		
hsa-miR-23b-3p	mir-23	CAPN6	✔	✔	✔	
hsa-miR-23b-3p	mir-23	CFL2	✔	✔		
hsa-miR-23b-3p	mir-23	CRISPLD1	✔	✔		
hsa-miR-23b-3p	mir-23	CXCL12	✔	✔	✔	
hsa-miR-23b-3p	mir-23	CYBRD1	✔	✔		
hsa-miR-23b-3p	mir-23	EBF1	✔	✔		
hsa-miR-23b-3p	mir-23	EBF3	✔	✔	✔	
hsa-miR-23b-3p	mir-23	ECH1				✔
hsa-miR-23b-3p	mir-23	EPAS1	✔	✔		
hsa-miR-23b-3p	mir-23	ERO1LB	✔	✔		
hsa-miR-23b-3p	mir-23	FAM46A	✔	✔		
hsa-miR-23b-3p	mir-23	FAM46C	✔	✔		
hsa-miR-23b-3p	mir-23	FGF2	✔	✔		
hsa-miR-23b-3p	mir-23	FOSB	✔	✔	✔	
hsa-miR-23b-3p	mir-23	FOXP2	✔	✔		
hsa-miR-23b-3p	mir-23	FZD4	✔	✔		
hsa-miR-23b-3p	mir-23	GPR64	✔	✔	✔	
hsa-miR-23b-3p	mir-23	KCNH1	✔	✔		
hsa-miR-23b-3p	mir-23	LPAR1	✔	✔		
hsa-miR-23b-3p	mir-23	MAT2A	✔	✔	✔	
hsa-miR-23b-3p	mir-23	MEF2C	✔	✔	✔	
hsa-miR-23b-3p	mir-23	MFAP5	✔	✔		
hsa-miR-23b-3p	mir-23	MYCT1	✔	✔		
hsa-miR-23b-3p	mir-23	NEGR1	✔	✔		
hsa-miR-23b-3p	mir-23	NFIB	✔	✔		
hsa-miR-23b-3p	mir-23	NRK	✔	✔		
hsa-miR-23b-3p	mir-23	PBX1	✔	✔		
hsa-miR-23b-3p	mir-23	PIK3C2A	✔	✔		
hsa-miR-23b-3p	mir-23	PLAGL1	✔	✔	✔	
hsa-miR-23b-3p	mir-23	PLCB4	✔		✔	
hsa-miR-23b-3p	mir-23	PLCXD3	✔	✔	✔	

To be continued...

miRNA	Rfam	Gene	miRanda	TargetScan	PicTar	mirTarBase
hsa-miR-23b-3p	mir-23	PPP1R12A	✔	✔		
hsa-miR-23b-3p	mir-23	RBPMS2	✔	✔		✔
hsa-miR-23b-3p	mir-23	RDH10	✔	✔		
hsa-miR-23b-3p	mir-23	SFRP1	✔	✔		
hsa-miR-23b-3p	mir-23	SHB	✔	✔		
hsa-miR-23b-3p	mir-23	SYNPO2	✔	✔		
hsa-miR-23b-3p	mir-23	TEAD1	✔	✔		
hsa-miR-23b-3p	mir-23	TGFBR2	✔	✔		
hsa-miR-23b-3p	mir-23	TGFBR3	✔	✔	✔	
hsa-miR-23b-3p	mir-23	TMEM150C	✔	✔		
hsa-miR-23b-3p	mir-23	TMOD1	✔	✔	✔	
hsa-miR-23b-3p	mir-23	VEPH1	✔	✔		
hsa-miR-23b-3p	mir-23	WASF3	✔	✔		
hsa-miR-23b-3p	mir-23	ZEB1	✔	✔		
hsa-miR-29b-2-5p	mir-29	ADAMTS9	✔		✔	
hsa-miR-29b-2-5p	mir-29	ARRDC4	✔		✔	
hsa-miR-29b-2-5p	mir-29	EMP1	✔		✔	
hsa-miR-29b-2-5p	mir-29	FAM190B	✔		✔	
hsa-miR-29b-2-5p	mir-29	MAT2A	✔		✔	
hsa-miR-29b-2-5p	mir-29	PRICKLE2	✔		✔	
hsa-miR-29b-2-5p	mir-29	REV3L	✔		✔	
hsa-miR-29b-2-5p	mir-29	TLL1	✔		✔	
hsa-miR-30b-5p	mir-30	ABCC9	✔	✔		
hsa-miR-30b-5p	mir-30	ABCD2	✔	✔		
hsa-miR-30b-5p	mir-30	ABI3BP	✔	✔		
hsa-miR-30b-5p	mir-30	ACTC1	✔	✔	✔	
hsa-miR-30b-5p	mir-30	ADAMTS9	✔	✔		
hsa-miR-30b-5p	mir-30	AFF3	✔	✔		
hsa-miR-30b-5p	mir-30	ARID5B	✔	✔		
hsa-miR-30b-5p	mir-30	BNC2		✔	✔	
hsa-miR-30b-5p	mir-30	CACHD1	✔	✔		
hsa-miR-30b-5p	mir-30	CADM2	✔	✔	✔	
hsa-miR-30b-5p	mir-30	CFL2	✔	✔	✔	
hsa-miR-30b-5p	mir-30	CHIC1	✔	✔		
hsa-miR-30b-5p	mir-30	CYYR1	✔	✔		
hsa-miR-30b-5p	mir-30	DMD	✔	✔	✔	
hsa-miR-30b-5p	mir-30	EBF3	✔	✔	✔	
hsa-miR-30b-5p	mir-30	EDNRB	✔		✔	
hsa-miR-30b-5p	mir-30	ERG	✔	✔	✔	
hsa-miR-30b-5p	mir-30	ERRFI1	✔	✔		
hsa-miR-30b-5p	mir-30	FAM13C	✔	✔		
hsa-miR-30b-5p	mir-30	FAM46A	✔	✔		
hsa-miR-30b-5p	mir-30	FAM46C	✔	✔		
hsa-miR-30b-5p	mir-30	FERMT2				✔
hsa-miR-30b-5p	mir-30	FRZB	✔	✔		✔
hsa-miR-30b-5p	mir-30	GATM	✔	✔		
hsa-miR-30b-5p	mir-30	ITSN1	✔	✔		
hsa-miR-30b-5p	mir-30	KSR1	✔	✔		
hsa-miR-30b-5p	mir-30	LIFR	✔	✔		
hsa-miR-30b-5p	mir-30	LPHN3	✔	✔	✔	
hsa-miR-30b-5p	mir-30	LPPR4	✔	✔		
hsa-miR-30b-5p	mir-30	MAT2A	✔	✔	✔	
hsa-miR-30b-5p	mir-30	MEOX2	✔		✔	
hsa-miR-30b-5p	mir-30	MYH10	✔	✔		

To be continued...

miRNA	Rfam	Gene	miRanda	TargetScan	PicTar	mirTarBase
hsa-miR-30b-5p	mir-30	MYH11	✔	✔		
hsa-miR-30b-5p	mir-30	NEGR1	✔	✔	✔	
hsa-miR-30b-5p	mir-30	NFIB	✔	✔	✔	
hsa-miR-30b-5p	mir-30	NLGN1	✔		✔	
hsa-miR-30b-5p	mir-30	NR4A2	✔	✔	✔	
hsa-miR-30b-5p	mir-30	NRK	✔	✔	✔	
hsa-miR-30b-5p	mir-30	PLCXD3		✔	✔	
hsa-miR-30b-5p	mir-30	PPIL4	✔	✔		
hsa-miR-30b-5p	mir-30	PPP1R12A	✔	✔	✔	
hsa-miR-30b-5p	mir-30	PRUNE2	✔	✔		
hsa-miR-30b-5p	mir-30	PTP4A1	✔	✔		
hsa-miR-30b-5p	mir-30	RAVER2	✔	✔		
hsa-miR-30b-5p	mir-30	RECK	✔	✔		
hsa-miR-30b-5p	mir-30	RFTN2	✔	✔		
hsa-miR-30b-5p	mir-30	RNF122	✔	✔	✔	
hsa-miR-30b-5p	mir-30	SOBP	✔	✔		
hsa-miR-30b-5p	mir-30	SSBP2	✔	✔		
hsa-miR-30b-5p	mir-30	TACC1	✔	✔		
hsa-miR-30b-5p	mir-30	TIMP3	✔	✔	✔	
hsa-miR-30b-5p	mir-30	TMOD2		✔	✔	
hsa-miR-30b-5p	mir-30	TNXB	✔	✔	✔	
hsa-miR-30b-5p	mir-30	TRIM9		✔	✔	
hsa-miR-30b-5p	mir-30	TTLL7	✔	✔	✔	
hsa-miR-30b-5p	mir-30	UBE2J1	✔	✔	✔	
hsa-miR-30b-5p	mir-30	VIP	✔	✔	✔	
hsa-miR-30b-5p	mir-30	ZNF280B	✔	✔	✔	
hsa-miR-328		C5orf4	✔	✔		
hsa-miR-328		LMOD1		✔	✔	
hsa-miR-328		MN1	✔	✔		
hsa-miR-328		PGM5	✔	✔		
hsa-miR-330-3p	mir-330	ANKS1B	✔		✔	
hsa-miR-330-3p	mir-330	ANO6	✔	✔		
hsa-miR-330-3p	mir-330	APCDD1	✔	✔		
hsa-miR-330-3p	mir-330	APOLD1	✔	✔		
hsa-miR-330-3p	mir-330	ARID5B	✔	✔		
hsa-miR-330-3p	mir-330	CADM3	✔	✔	✔	
hsa-miR-330-3p	mir-330	CD44				✔
hsa-miR-330-3p	mir-330	COL19A1	✔	✔		
hsa-miR-330-3p	mir-330	CRISPLD1	✔	✔	✔	
hsa-miR-330-3p	mir-330	CRISPLD2	✔	✔		
hsa-miR-330-3p	mir-330	DCLK1	✔	✔	✔	
hsa-miR-330-3p	mir-330	DPYSL3	✔	✔	✔	
hsa-miR-330-3p	mir-330	EFEMP1	✔	✔		
hsa-miR-330-3p	mir-330	ERGIC2	✔	✔		
hsa-miR-330-3p	mir-330	FAM46C	✔	✔		
hsa-miR-330-3p	mir-330	FGFR1	✔	✔	✔	
hsa-miR-330-3p	mir-330	FOXP2	✔	✔		
hsa-miR-330-3p	mir-330	GPAM	✔	✔	✔	
hsa-miR-330-3p	mir-330	KANK2	✔	✔	✔	
hsa-miR-330-3p	mir-330	MAT2A	✔	✔		
hsa-miR-330-3p	mir-330	MEF2C	✔	✔	✔	
hsa-miR-330-3p	mir-330	MN1	✔	✔		
hsa-miR-330-3p	mir-330	MYH10	✔	✔	✔	
hsa-miR-330-3p	mir-330	PCDH9	✔	✔	✔	

To be continued...

miRNA	Rfam	Gene	miRanda	TargetScan	PicTar	mirTarBase
hsa-miR-330-3p	mir-330	PDE7B	✔	✔		
hsa-miR-330-3p	mir-330	PDGFD	✔		✔	
hsa-miR-330-3p	mir-330	PIK3R1	✔	✔		
hsa-miR-330-3p	mir-330	PRUNE2	✔	✔		
hsa-miR-330-3p	mir-330	PTGFR	✔	✔		
hsa-miR-330-3p	mir-330	PTP4A1	✔	✔		
hsa-miR-330-3p	mir-330	RAVER2	✔	✔		
hsa-miR-330-3p	mir-330	RCAN1	✔	✔		
hsa-miR-330-3p	mir-330	RNF180	✔	✔		
hsa-miR-330-3p	mir-330	SFRP1	✔	✔		
hsa-miR-330-3p	mir-330	SH3BGRL2	✔	✔		
hsa-miR-330-3p	mir-330	SLIT2				✔
hsa-miR-330-3p	mir-330	ST6GALNAC3	✔	✔		
hsa-miR-330-3p	mir-330	TBCEL	✔	✔		
hsa-miR-330-3p	mir-330	TEAD1	✔	✔		
hsa-miR-330-3p	mir-330	TGFBR3	✔	✔		
hsa-miR-330-3p	mir-330	TIPARP	✔		✔	
hsa-miR-330-3p	mir-330	UBE2J1	✔	✔		
hsa-miR-339-3p	mir-339	KCNMA1	✔		✔	
hsa-miR-342-3p	mir-342	ANK2	✔	✔		
hsa-miR-342-3p	mir-342	FLNC	✔	✔		
hsa-miR-342-3p	mir-342	LPPR4	✔	✔		
hsa-miR-342-3p	mir-342	PBX1	✔	✔		
hsa-miR-342-3p	mir-342	TACC1				✔
hsa-miR-342-3p	mir-342	TMTC2	✔	✔		
hsa-miR-342-3p	mir-342	ZEB1	✔	✔	✔	
hsa-miR-342-3p	mir-342	ZNF462	✔		✔	
hsa-miR-345-5p	mir-345	CAPN6	✔	✔		
hsa-miR-345-5p	mir-345	FOXP2	✔	✔		
hsa-miR-345-5p	mir-345	SORBS1	✔	✔		
hsa-miR-345-5p	mir-345	SSBP2	✔	✔	✔	
hsa-miR-378b	mir-378	KSR1	✔	✔		
hsa-miR-378b	mir-378	PARVA	✔	✔		
hsa-miR-378b	mir-378	RBMS3	✔	✔		
hsa-miR-455-3p	mir-455	CDON	✔	✔		
hsa-miR-455-3p	mir-455	CHRDL1	✔	✔		
hsa-miR-455-3p	mir-455	ERG	✔	✔		
hsa-miR-455-3p	mir-455	FABP4	✔	✔		
hsa-miR-455-3p	mir-455	FAM171B	✔	✔		
hsa-miR-455-3p	mir-455	FGF7	✔	✔		
hsa-miR-455-3p	mir-455	FOSB	✔	✔		
hsa-miR-455-3p	mir-455	MAP1B				✔
hsa-miR-455-3p	mir-455	NFIB	✔	✔		
hsa-miR-455-3p	mir-455	PCDH9	✔	✔		
hsa-miR-455-3p	mir-455	PIK3R1	✔	✔		
hsa-miR-455-3p	mir-455	RBPMS2	✔	✔		
hsa-miR-455-3p	mir-455	SPRY1	✔	✔		
hsa-miR-455-3p	mir-455	TSPAN18	✔	✔		
hsa-miR-455-3p	mir-455	ZEB1	✔	✔		
hsa-miR-455-3p	mir-455	ZNF462	✔	✔		
hsa-miR-489		CCRL1	✔	✔		
hsa-miR-489		CILP	✔	✔		
hsa-miR-489		KIAA1324L	✔	✔		
hsa-miR-489		PARM1	✔	✔		

To be continued...

miRNA	Rfam	Gene	miRanda	TargetScan	PicTar	mirTarBase
hsa-miR-489		RBMS3	✔	✔		
hsa-miR-492	mir-492	ADAMTS1	✔	✔		
hsa-miR-492	mir-492	CD44	✔	✔		
hsa-miR-492	mir-492	MAT2A	✔	✔		
hsa-miR-492	mir-492	PARVA	✔	✔		
hsa-miR-500a-5p	mir-500	CACHD1	✔	✔		
hsa-miR-500a-5p	mir-500	CALCRL	✔	✔		
hsa-miR-500a-5p	mir-500	DCLK1	✔	✔		
hsa-miR-500a-5p	mir-500	DLC1	✔	✔		
hsa-miR-500a-5p	mir-500	DMD	✔	✔		
hsa-miR-500a-5p	mir-500	ERRFI1	✔	✔		
hsa-miR-500a-5p	mir-500	FAM190B	✔	✔		
hsa-miR-500a-5p	mir-500	MFSD4	✔	✔		
hsa-miR-500a-5p	mir-500	PARVA	✔	✔		
hsa-miR-500a-5p	mir-500	PDK4	✔	✔		
hsa-miR-500a-5p	mir-500	PTP4A1	✔	✔		
hsa-miR-500a-5p	mir-500	PTPRD	✔	✔		
hsa-miR-500a-5p	mir-500	RBPMS2	✔	✔		
hsa-miR-500a-5p	mir-500	TMEM47	✔	✔		
hsa-miR-532-3p	mir-188	CAPN6	✔	✔		
hsa-miR-532-3p	mir-188	CHRDL1	✔	✔		
hsa-miR-532-3p	mir-188	DES	✔	✔		
hsa-miR-532-3p	mir-188	ITSN1	✔	✔		
hsa-miR-532-3p	mir-188	MSRB3	✔	✔		
hsa-miR-532-3p	mir-188	PTGER3	✔	✔		
hsa-miR-532-3p	mir-188	PTP4A1	✔	✔		
hsa-miR-532-3p	mir-188	ZEB1	✔	✔		
hsa-miR-532-5p	mir-188	CXCL12	✔	✔		
hsa-miR-532-5p	mir-188	FAM190B	✔	✔		
hsa-miR-532-5p	mir-188	NFIB	✔	✔		
hsa-miR-532-5p	mir-188	PCDH9	✔	✔		
hsa-miR-532-5p	mir-188	RAVER2	✔	✔		
hsa-miR-532-5p	mir-188	TTN	✔	✔		
hsa-miR-532-5p	mir-188	UST	✔	✔		
hsa-miR-874		AFF3	✔	✔		
hsa-miR-874		GPC6	✔	✔		
hsa-miR-874		HEG1	✔	✔		
hsa-miR-874		MSRB3	✔	✔		
hsa-miR-874		TRIM9	✔	✔		
hsa-miR-885-5p	mir-885	OSBPL6	✔	✔		
hsa-miR-885-5p	mir-885	PARVA	✔	✔		
hsa-miR-885-5p	mir-885	SEMA3D	✔	✔		
hsa-miR-885-5p	mir-885	SH2D1A	✔	✔		
hsa-miR-885-5p	mir-885	SH3BGRL2	✔	✔		

(END)
