# Supplementary material for: Downregulation of miR-181b-5p Inhibits the Viability, Migration, and Glycolysis of Gallbladder Cancer by Upregulating PDHX Under Hypoxia
Source: Front Oncol. 2021 Aug 16;11:683725. doi: 10.3389/fonc.2021.683725 (PMC8415503; doi:10.3389/fonc.2021.683725)
Supplement: Supplementary file 3 [file DataSheet_1.zip › RNA seq raw data/mirPath Analysis/A vs B_up miRNA dn Gene/README.pdf]

miRNA-Pathway Analysis

KC-TBY

December 27, 2013

1 Target Prediction

miRNA-target gene prediction is based on database querying. When a miRNA-gene pair can be found in 2 of the 3 databases (miRanda,TargetScan,PicTar) or can be found in the mirTarBase, the gene is predicted to be the target of the miRNA.

1.1 Database Version

miRanda Aug 2010

TargetScan V6.2, Jun 2012

PicTar 2006

mirTarBase V4.5, Nov 2013

2 Output

The miRNA-Pathway analysis will produce following output:

- File
  - Pathway Statistics.xls containing:
    1. The statistics from Fisher exact test
    2. The p-values and q-values from random sampling
    3. The predicted target genes (colored in cyan, red if this gene is also differentially expressed) of each miRNA and their belonging pathways
- Directory
  - **Heatmap** containing heatmaps with binned values from the pathway statistics:
    - **OrderedByFisherExact.pdf** Hierarchical clustering on the miRNAs and the pathways are sorted by Fisher exact p-values.
    - **OrderedByRandomSampling.pdf** Hierarchical clustering on the miRNAs and the pathways are sorted by random sampling p-values.
    - **Biclustering.pdf** Hierarchical clustering on both the miRNAs and the pathways
  - **PathNet** containing the associated miRNA-pathway connection for Network analysis. Both gephi and cytoscape are supported.
  - **GeneNet** containing the associated miRNA-gene connection for Network analysis. Both gephi and cytoscape are supported.
  - **DEG Target (optional)** The detailed statistics of database hit for each differentially miRNA-gene pairs.

3 Detailed Settings

Fisher exact p-value The p-value is the exact two-tailed hypergeometric p-value whenever possible. Or the Yates's correction for continuity is used to calculate the approximated Chi-squared p-value.

Random Sampling $n = 10^5$, where n is the repeat times of the sampling procedure

Hierarchical clustering Euclidean distance, average-linkage
