# Supplementary material for: Downregulation of miR-181b-5p Inhibits the Viability, Migration, and Glycolysis of Gallbladder Cancer by Upregulating PDHX Under Hypoxia
Source: Front Oncol. 2021 Aug 16;11:683725. doi: 10.3389/fonc.2021.683725 (PMC8415503; doi:10.3389/fonc.2021.683725)
Supplement: Supplementary file 3 [file diffCoding.pdf › RNA seq raw data/Heatmap/NoRowNormalization/diffCoding.pdf]

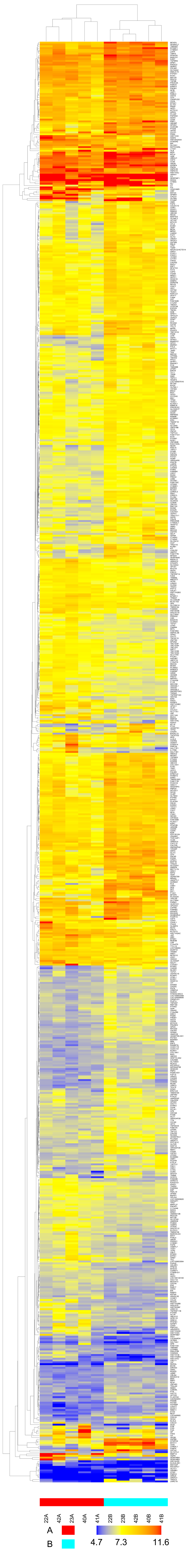
22A
42A
23A
40A
41A
22B
23B
42B
40B
41B
A
B
4.7
7.3
11.6
