# Supplementary material for: Downregulation of miR-181b-5p Inhibits the Viability, Migration, and Glycolysis of Gallbladder Cancer by Upregulating PDHX Under Hypoxia
Source: Front Oncol. 2021 Aug 16;11:683725. doi: 10.3389/fonc.2021.683725 (PMC8415503; doi:10.3389/fonc.2021.683725)
Supplement: Supplementary file 3 [file varTop2000LncRNA.pdf › RNA seq raw data/Heatmap/NoRowNormalization/varTop2000LncRNA.pdf]

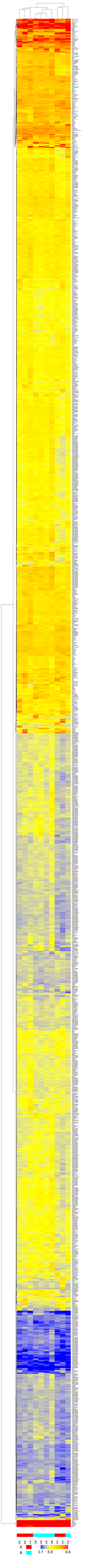
22A
40A
41A
22B
42B
23B
40B
23A
42A
41B
A
B
3.7
5.8
9.8
